# Programmed S → N Remote Cyano Migration: A Thiocyanate-Based Entry to Cyanamides and Selective Ferroptosis Inducers

**DOI:** 10.1021/acscentsci.6c00645

**Published:** 2026-08-06

**Authors:** Xiyan Duan, Wanjing Feng, Fengjiao Du, Can Liu, Ning Liu, Yihao Zhao, Kun Liu, Pu Liu, Yixuan Zhang, Jingze Li, Yahui Ding, Quan Zhang

**Affiliations:** † School of Chemistry & Chemical Engineering, 74623 Henan University of Science and Technology, Luoyang 471003, Henan, China; ‡ College of Pharmacy, State Key Laboratory of Medicinal Chemical Biology and Tianjin Key Laboratory of Molecular Drug Research, 12538 Nankai University, Tianjin 300350, China; § College of Chemistry, State Key Laboratory of Medicinal Chemical Biology, 12538 Nankai University, Tianjin 300071, China; ∥ School of Nursing, 74623 Henan University of Science and Technology, Luoyang 471003, Henan, China; ⊥ College of Food & Bioengineering, 74623 Henan University of Science and Technology, Luoyang 471003, Henan, China

## Abstract

Ferroptosis represents a promising strategy to overcome apoptosis-associated drug resistance, yet access to diverse and drug-like ferroptosis-inducing chemical space remains limited. Among potential scaffolds for populating this high-value space, cyanamide-bearing molecules are attractive but synthetically challenging due to the long-standing difficulty of constructing the N–CN bond. Here we disclose a thiocyanate-enabled cascade cyclization that leverages inexpensive, low-toxicity inorganic thiocyanate as an unconventional electrophilic CN surrogate. This transformation proceeds through a cascade sequence involving SCN installation, remote S → N cyano migration, and subsequent intramolecular thiazine annulation under mild, metal-free conditions. The resulting cyanamide 3,4-dihydro-2H-1,4-thiazines exhibited nanomolar ferroptosis-inducing potency; lead compounds **3b** and **3c** achieved IC_50_ values of 6.8 nM and 14 nM, respectively; and significantly suppressed tumor growth in a lung cancer PDX model.

## Graphical abstract

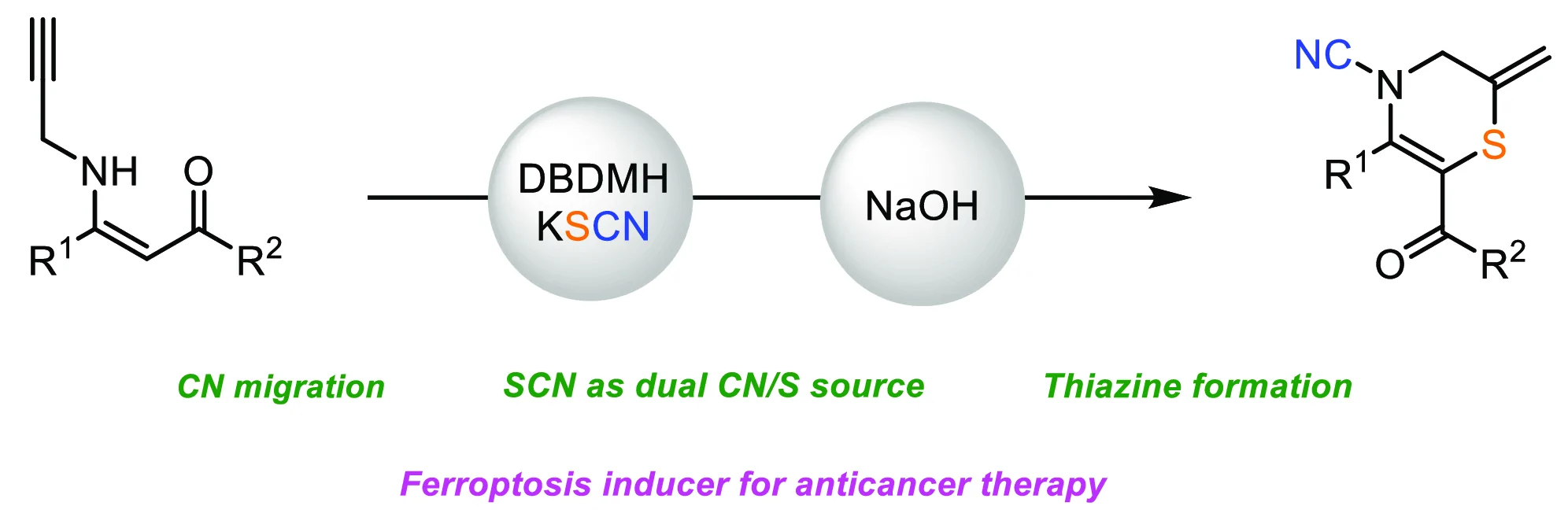

Cancer cells frequently acquire multidrug resistance through gene mutations, overexpression of antiapoptotic proteins, and upregulation of drug efflux pumps, ultimately causing relapses and metastatic progression. In nonsmall cell lung cancer, approximately 50–60% of patients acquire resistance within one year of first-generation epidermal growth factor receptor (EGFR) tyrosine kinase inhibitor therapy,^1^ leading to treatment failure and often promoting tumor dedifferentiation and metastasis. Ferroptosis-a programmed cell death driven by iron accumulation, glutathione depletion, and GPX4 inactivation, ultimately leading to catastrophic lipid peroxidation of polyunsaturated fatty acids (PUFAs)-has emerged as a promising alternative strategy for targeting aggressive and poorly differentiated cancers.^2^ However, despite rapid advances in ferroptosis research, clinical translation remains limited owing to the poor solubility, low stability, and insufficient selectivity of widely used inducers like erastin and RSL3 (Figure 1a).^3^ To date, no selective ferroptosis inducer has been approved, underscoring the urgent need to develop novel therapeutic agents with improved efficacy, selectivity, and superior pharmaceutical properties.

**Figure 1:**
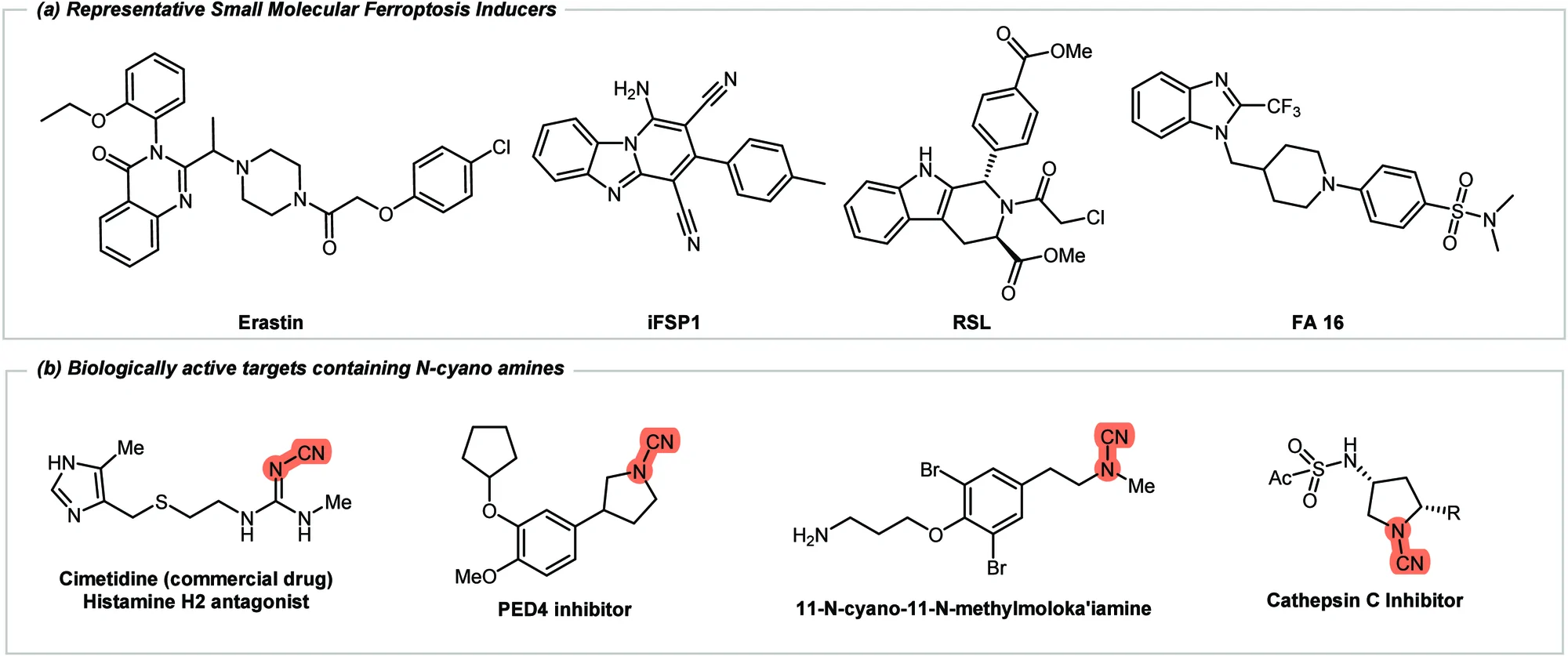
(a) Representative small-molecule ferroptosis inducers. (b) Biologically active molecules containing N-cyanoamines.

Cyanamides serve as versatile intermediates for assembling nitrogen-containing heterocycles. In medicinal chemistry, the cyanamide moiety is not only a useful structural motif but also a functionally relevant pharmacophore in a variety of bioactive molecules (Figure 1b).^4^ Previous studies have shown that the cyanamide moiety can contribute to biological activity by tuning electrophilic reactivity and by participating in specific interactions with biological targets. For example, in cyanamide-based covalent JAK3 inhibitors, the cyanamide group serves as a distinctive electrophilic moiety that enables productive engagement with the target cysteine residue, thereby contributing substantially to inhibitory potency and selectivity.^5^ Despite their value, practical access to cyanamides remains limited by the intrinsic difficulty of forming the N–CN bond and the reliance on hazardous cyanide reagents.^6,7^ Historically, cyanamide synthesis relied on highly toxic cyanogen halides,^8^ while more recent electrophilic N-cyanation approaches still require preprepared CN-transfer reagents derived from toxic sources such as BrCN or trimethylsilyl cyanide (TMSCN) (Figure 2a, b).^9–18^ Additional limitations-including instability, high cost, harsh conditions, and low atom economy-further hinder broad adoption.^19,20^

**Figure 2:**
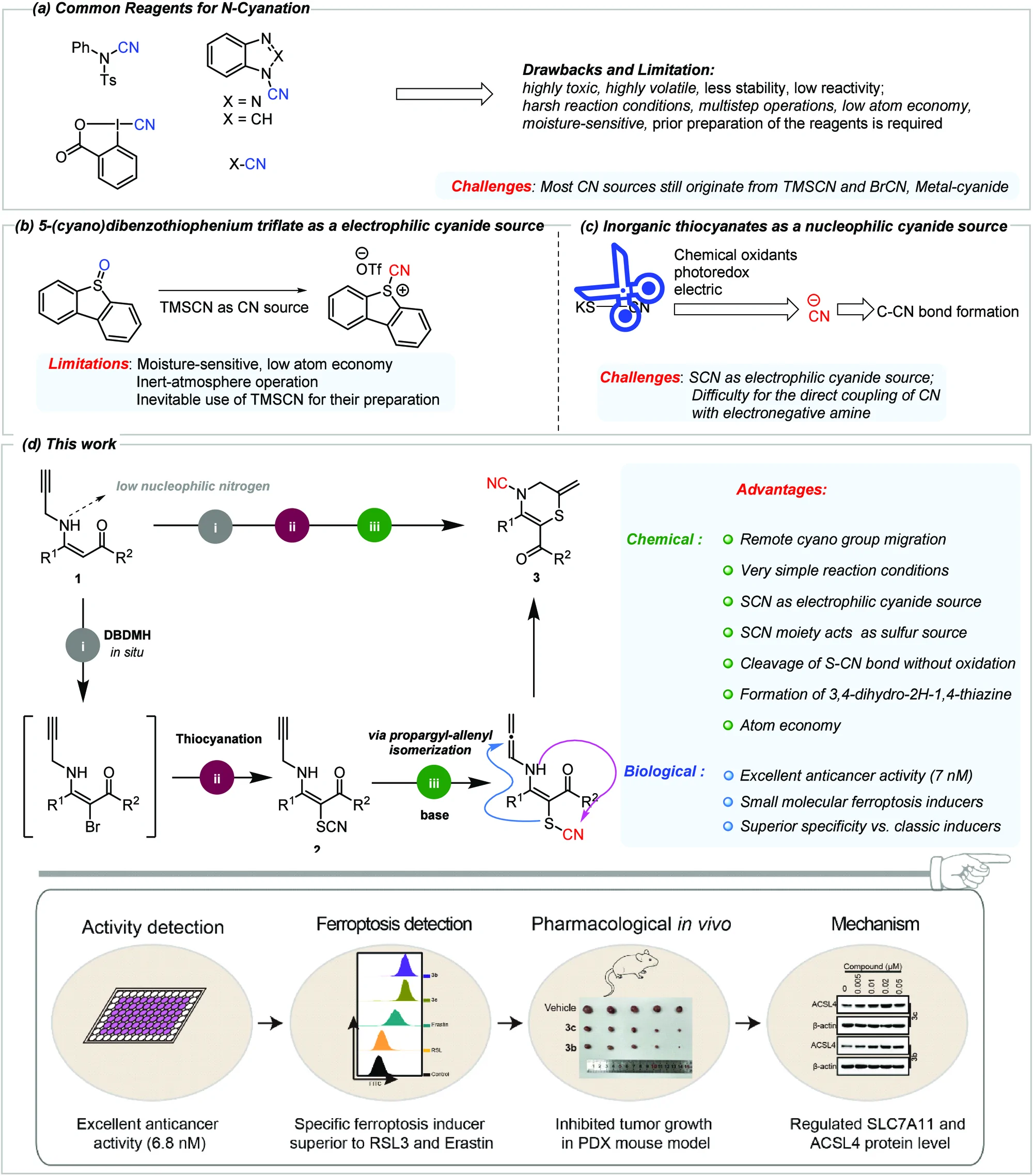
(a) Existing N-cyanation methods. (b) 5-(cyano)­dibenzothiophenium triflate as an electrophilic cyanide source. (c) Inorganic thiocyanates as a nucleophilic cyanide source. (d) This work.

Inorganic thiocyanates (e.g., KSCN) are inexpensive, stable, and easy to handle, and thus represent ideal “benign CN equivalents” (Figure 2c).^21–23^ Under oxidative conditions, thiocyanates can generate nucleophilic cyanide in situ and have enabled numerous C–CN bond-forming reactions.^21–29^ In sharp contrast, strategies that leverage thiocyanates for electrophilic cyanation, particularly for direct N–CN bond formation, remain largely unexplored. We sought to address this gap by designing a cascade process in which SCN is first installed, and the cyano group is then unlocked and relayed from sulfur to nitrogen under basic conditions, thereby avoiding preformed toxic cyanide reagents.

The precise control of chemical structure, particularly of reactive centers, is central to the development of functional systems with desirable properties in both molecular and materials chemistry.^30–36^ In this context, N-propargylic β-enaminones have emerged as versatile building blocks owing to their well-defined and tunable reaction centers, which enable their facile conversion into pyridines, pyrrolines, pyrroles, and medium-ring heterocycles.^37–39^ Under basic conditions, they can undergo propargyl–allenyl isomerization to form a highly electrophilic allenyl-enaminone intermediate, enabling diverse cascade transformations.^40–43^ Building on our continuing interest in enamine chemistry,^44–47^ we envisioned that an SCN-functionalized N-propargylic β-enaminone could be programmed to cyclize into a 3,4-dihydro-2*H*-1,4-thiazine framework, followed by selective S–CN cleavage and a remote sulfur to nitrogen cyano migration to forge the cyanamide motif. Here we report a highly chemoselective, one-pot cascade that converts N-propargylic β-enaminones into cyanamide-bearing 3,4-dihydro-2*H*-1,4-thiazines (Figure 2d). This transformation integrates multiple elementary events—SCN installation, chemoselective S–CN bond cleavage enabling remote S → N cyano relay, and subsequent propargyl–allenyl isomerization/intramolecular cyclization—thereby providing a direct and practical entry to cyanamides from a benign thiocyanate feedstock. Importantly, biological evaluation revealed this scaffold class to be exceptionally potent anticancer agents, with IC_50_ value as low as 6.8 nM. Compounds **3b** and **3c** further exhibited excellent potency in inducing ferroptosis, and their *in vivo* efficacy was validated in a lung cancer patient-derived xenograft (PDX) model, where both tumor volume and tumor weight were significantly reduced.

## Synthesis of 3,4-Dihydro-2*H*-1,4-thiazine Analogues

After systematic evaluation of temperature, bases, additives, and other parameters (see Supporting Information, Table S1), a two-stage, one-pot protocol was identified (Scheme 1). In stage 1, substrate **1** was first treated with DBDMH in DMF at 60 °C, followed by addition of KSCN to install the SCN unit and generate the crude intermediate. In stage 2, the residue was directly subjected to NaOH and 15-crown-5 in DMF at room temperature to promote cascade cyclization and remote cyano relay, affording the desired 3,4-dihydro-2*H*-1,4-thiazines **3** in moderate yields (42%–75%). The absolute configuration of **3a** was established by X-ray crystallography (CCDC 2497077, Figure S4, Table S3), and other products were assigned by analogy. The substrate scope for this reaction is very broad. Variation of R^1^ on the enaminone aryl group showed that electron-neutral (H), electron-donating (MeO, Me, *t*-Bu, propargyl), and electron-withdrawing (OCF_3_, F, Cl, Br) substituents at the *para* or *meta* positions were well tolerated (**3a**–**3m**). For example, *para*-substituted OMe- and Me-substituted substrates afforded the corresponding products (**3b**–**c**) in 64% and 75% yields, respectively, while *para*-substituted F, Cl, Br, and OCF_3_ derivatives (**3d**–**g**) gave comparable yields (60–63%), suggesting that the transformation is not highly sensitive to the electronic characteristics of the aryl substituents. In contrast, steric and positional effects had a more pronounced influence on the reaction efficiency. The presence of bulky substituents such as *t*-Bu (**3h**) and propargyl (**3i**) led to a decrease in the isolated yields. Naphthyl substrates also reacted smoothly (**3n**–**3o**), and heteroaryl enaminones (furan- and thiophene-derived) gave **3p** and **3q** in moderate yields, indicating strong tolerance toward heterocycles. Replacement of the carbonyl with an ester group furnished **3r** in 60% yield. We next examined substitution at R^2^ on the enaminone aryl ring. Methoxy-, alkyl-, propargyl-, and halogen-substituted substrates were efficiently converted to **3s**–**3ac**, and thiophene/naphthyl variants (**1ad**, **1ae**) were also compatible. Disubstituted aryl substrates (**1af**–**1an**) provided **3af**–**3an** in moderate to good yields, highlighting the practicality of the cascade in generating structurally diverse analogues (Scheme 1).

**Scheme 1:**
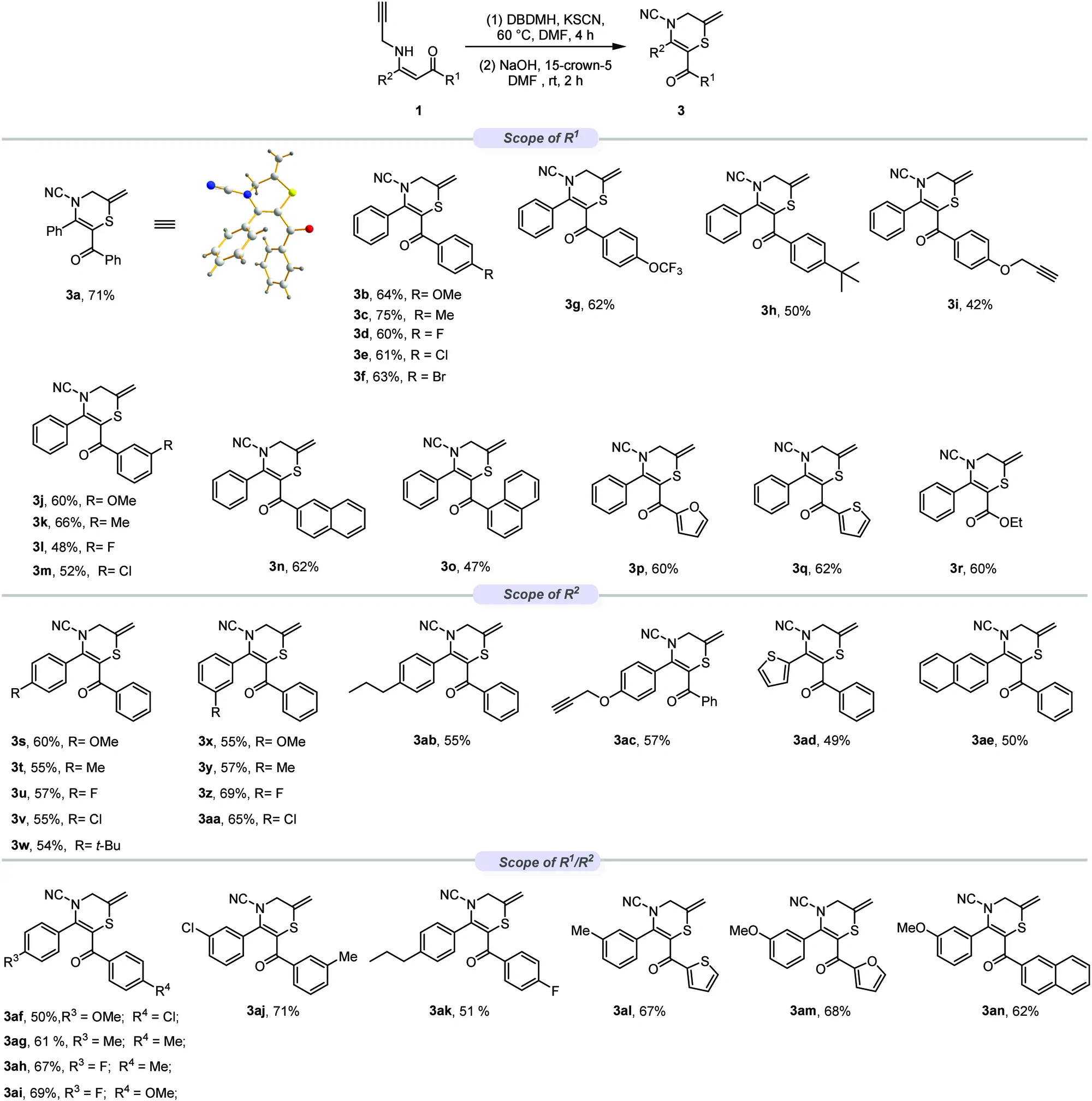
Substrates Scope^a^,^b^

a Reaction conditions: Step 1: **1** (2 mmol, 1.0 equiv), DBDMH (1 mmol, 0.5 equiv), DMF (2 mL), 60 °C, 2 h; then, KSCN (2 mmol, 1.0 equiv), DMF (4 mL), 2 h, workup. Step 2: Reaction residue of step 1, NaOH (2 mmol, 1.0 equiv), 15-crown-5 (2 mmol, 1.0 equiv), DMF (8 mL, 0.25 M), rt, 2 h.
b Isolated yields for two steps.

Some control experiments were conducted next to gain insight into the reaction mechanism. When intermediate **2a** was subjected to the standard conditions of the second step, product **3a** was readily obtained (Scheme 2A). This result provides direct experimental support for compound **2** as the intermediate of the reaction and is consistent with the proposed stepwise sequence involving initial thiocyanation followed by base-promoted cyano migration and cyclization. The addition of 3 equivalents of TEMPO under the standard reaction conditions afforded product **3a** in 59% yield (Scheme 2B), indicating that the reaction is not significantly inhibited by the radical scavenger and thus is unlikely to proceed predominantly through a radical mechanism. We next considered an alternative possibility that this transformation might proceed not through an intermolecular cyano-transfer process, but rather via free cyanide species generated in situ. To probe this issue, we designed and conducted a control experiment involving cyanide-species trapping to determine whether any cyanating species were present in the reaction system (Scheme 2C). When aniline was added under the standard reaction conditions, no corresponding cyanamide product was detected, whereas product **3a** was still obtained in 65% yield. These results indicate that the transformation most likely proceeds through an intramolecular cyano-transfer process (Scheme 2C). A deuterium-labeling experiment was also performed to probe the reaction mechanism (Scheme 2D). When D_2_O was added under the standard conditions, ^1^H NMR analysis revealed the formation of a mixture of partially deuterated products, in which deuterium was incorporated independently at either the C6 or the C7 position, affording **3a-D**, **3a′-D**, and **3a″-D** (Scheme S1). This result indicates that both sites can independently undergo proton/deuterium exchange during the reaction. The incomplete deuterium incorporation, together with the observed product distribution, suggests the presence of a reversible proton-transfer equilibrium during the interconversion process. To demonstrate the practical utility of this protocol, a gram-scale synthesis of **3c** was carried out under the standard conditions, affording **3c** in 70% isolated yield (Scheme 2E).

**Scheme 2:**
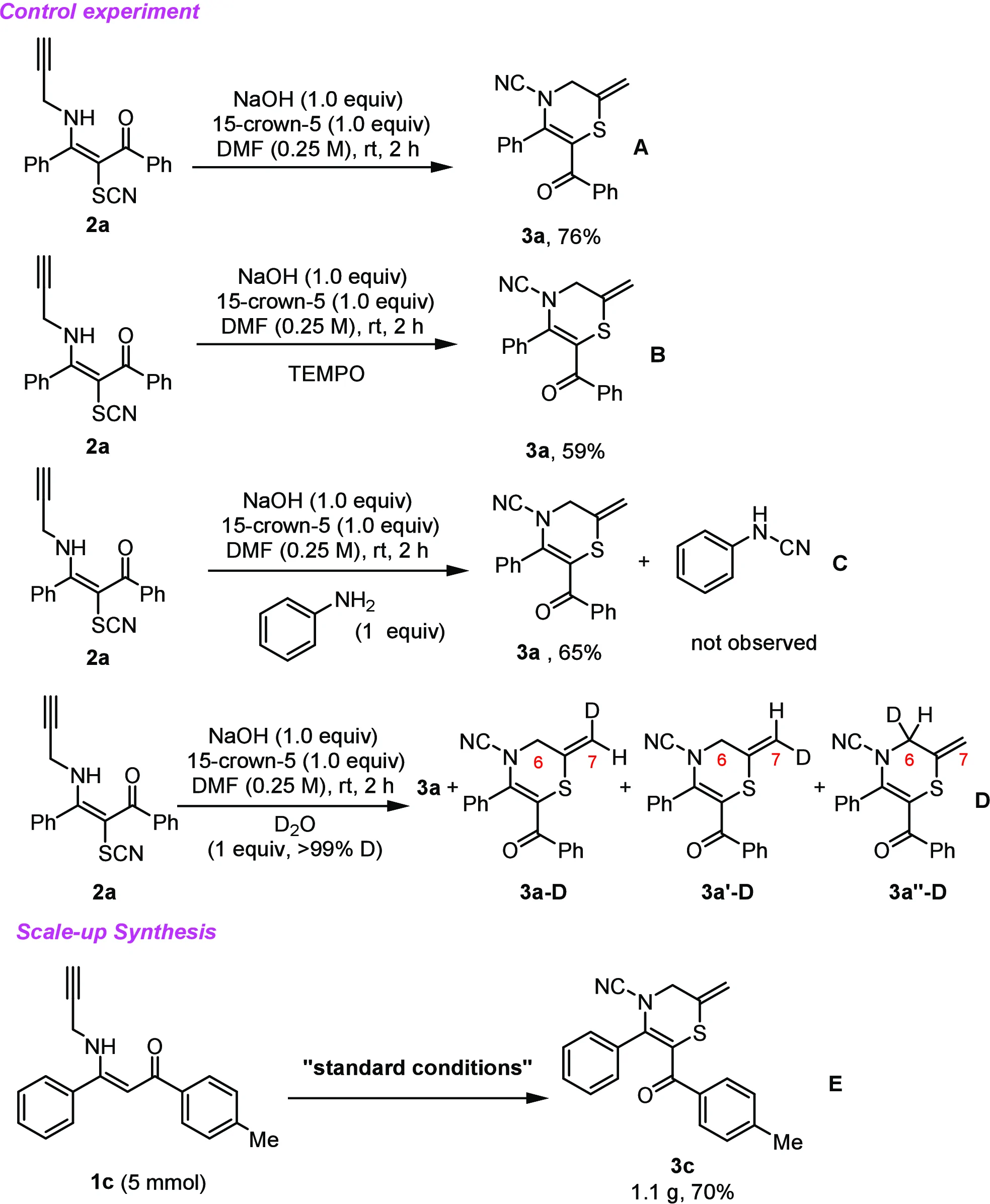
Control Experiment and Scale-up Synthesis

To gain deeper insight into the reaction mechanism, DFT calculations were performed using **2a** as a model substrate (Scheme 3, Figure S5, Table S4). Under basic conditions, the enamine NH unit of **2a** is readily deprotonated to form intermediate **INT1** in a highly exothermic process. **INT1** subsequently undergoes dehydration to generate an imine–carbanion intermediate, which is in resonance with the N-anionic enamine form denoted as **INT2**. Propargylic deprotonation is also feasible, but the resulting **INT2′** is 10.3 kcal/mol higher in energy than **INT2**, indicating that N–H deprotonation is energetically preferred. Subsequent rotation about the C–C single bond proceeds via **TS1** with an energy barrier of only 5.2 kcal/mol, leading to **INT3**. Once the N-anion and the SCN group are properly aligned, intramolecular nucleophilic attack of the N-anion at the cyano carbon of the SCN moiety occurs via **TS2**, requiring only 8.3 kcal/mol to form the five-membered-ring **INT4**. Cleavage of the C–S bond then takes place via **TS3** with a negligible barrier of 1.7 kcal/mol, yielding thiolate **INT5** and thereby completing the stepwise cyano migration. Subsequently, direct intramolecular nucleophilic addition of the thiolate to the alkyne moiety proceeds through **TS4** with an activation energy of 25.0 kcal/mol, affording the six-membered-ring **INT6**. Finally, a proton transfer process occurs via **TS5** with an energy barrier of only 6.1 kcal/mol, affording the final product **3a**. Alternatively, **INT5** may undergo propargyl–allene isomerization under basic conditions. The subsequent intramolecular nucleophilic addition of the thiolate to the allene moiety proceeds through **TS4′** with an activation energy of only 18.3 kcal/mol, affording the six-membered-ring intermediate **INT6′**. Finally, proton transfer via **TS5′** requires an energy barrier of 11.6 kcal/mol, affording product **3a**. Therefore, allene cyclization constitutes the major pathway, whereas alkyne cyclization is less favorable. Overall, analysis of the computed potential energy surface indicates that the cascade reaction proceeds through sequential conformational rotation, stepwise cyano migration, and allene cyclization, in excellent agreement with the experimental observations.

**Scheme 3:**
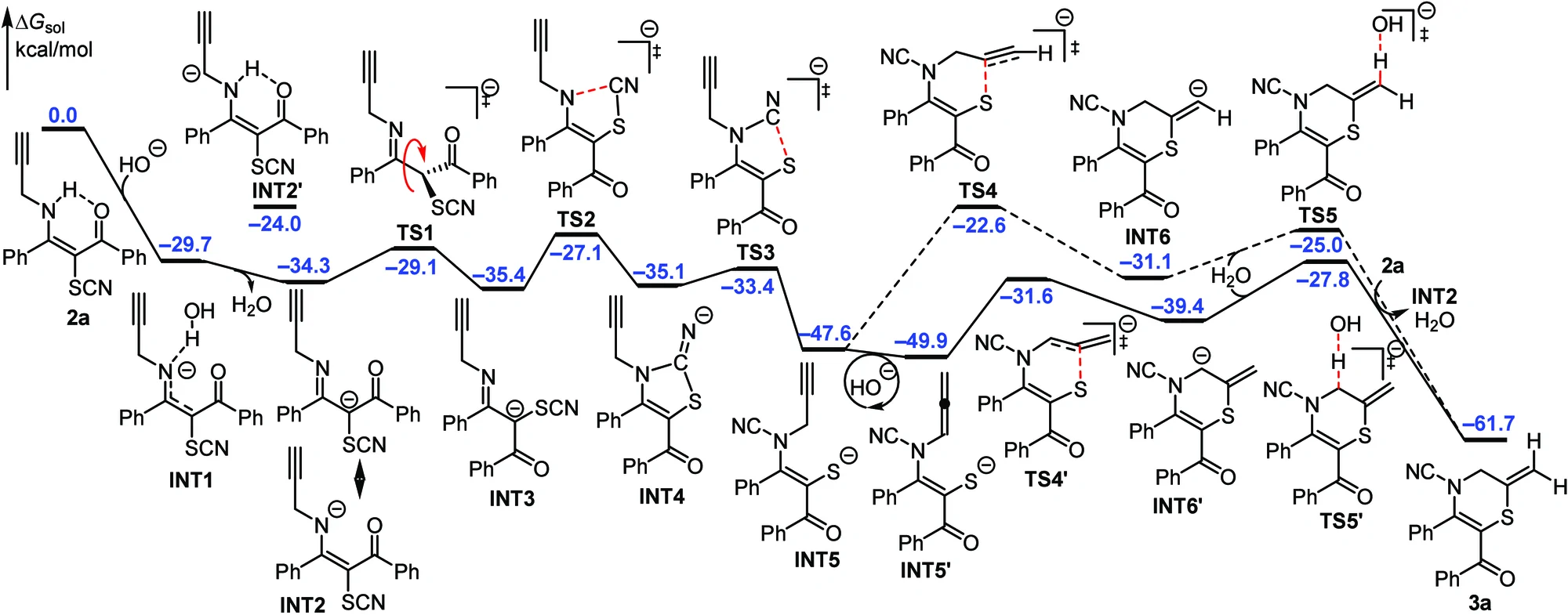
Proposed Reaction Mechanism

## Biological Assessment of 3,4-Dihydro-2*H*-1,4-thiazine Analogues

We next evaluated the *in vitro* antiproliferative activity of 3,4-dihydro-2*H*-1,4-thiazine derivatives **3a**–**3an** in the human nonsmall cell lung cancer cell line NCI-H820 using an MTT assay, with cisplatin as a positive control (Table 1). Across the series, the compounds exhibited moderate to potent activity, highlighting the pharmaceutical potential of this scaffold. Substitution on the R^1^ aryl ring (**3b**–**3r**) generally enhanced antiproliferative potency relative to R^2^-substituted analogues (**3s**–**3ae**), with *para*-substituted derivatives more active than their *meta*-substituted counterparts. Compounds **3b** and **3c** were the most potent, with **3b** (*para*-methoxy) exhibiting an IC_50_ of 6.8 nM, representing a 19-fold improvement over **3a** (IC_50_ = 0.13 μM).

**Table 1:** Cytotoxic Activities of Compounds **3a**–**3an** *in Vitro* (IC_50_, μM)^a^,^b^

Compd	NCI-H820^c^	Compd	NCI-H820^c^
**positive control** ^d^	3.112 ± 0.495	**3u**	0.16 ± 0.058
**3a**	0.13 ± 0.010	**3v**	0.66 ± 0.21
**3b**	0.0068 ± 0.0004	**3w**	>20
**3c**	0.014 ± 0.0006	**3x**	0.18 ± 0.068
**3d**	0.042 ± 0.012	**3y**	0.42 ± 0.14
**3e**	0.31 ± 0.035	**3z**	0.10 ± 0.039
**3f**	0.016 ± 0.0078	**3aa**	0.078 ± 0.016
**3g**	0.093 ± 0.035	**3ab**	15.98 ± 0.67
**3h**	8.66 ± 1.91	**3ac**	1.41 ± 0.09
**3i**	0.054 ± 0.0075	**3ad**	0.081 ± 0.041
**3j**	0.32 ± 0.071	**3ae**	>20
**3k**	0.47 ± 0.17	**3af**	1.13 ± 0.33
**3l**	0.034 ± 0.0092	**3ag**	0.50 ± 0.17
**3m**	0.33 ± 0.053	**3ah**	0.13 ± 0.003
**3n**	0.12 ± 0.0067	**3ai**	0.013 ± 0.002
**3o**	0.75 ± 0.15	**3aj**	0.18 ± 0.069
**3p**	0.13 ± 0.0012	**3ak**	12.06 ± 4.37
**3q**	0.080 ± 0.025	**3al**	0.34 ± 0.11
**3r**	1.90 ± 0.84	**3am**	0.53 ± 0.044
**3s**	2.79 ± 0.85	**3an**	0.34 ± 0.072
**3t**	2.84 ± 1.18		

a All values were the mean of three independent experiments.

b IC_50_ represented 50% cytotoxic concentration.

c NCI-H820 was the human lung cancer cell line.

d Cisplatin was used as the positive control.

Halogen substitution (**3d**–**3g**) typically increased potency (IC_50_ = 0.016–0.31 μM), whereas a bulky *tert*-butyl group (**3h**) markedly reduced activity. *Meta*-substituted analogues (**3j**, **3k**, **3m**) showed decreased potency (IC_50_ = 0.32–0.47 μM), consistent with steric sensitivity. Analogues bearing naphthyl, furan, or thiophene groups (**3n**–**3q**) retained strong activity, while ester **3r** exhibited low cytotoxicity (IC_50_ = 1.90 μM). Among R^2^-substituted compounds, representative examples (e.g., **3u**, **3x**, **3z**, **3aa**, **3ad**) maintained submicromolar activity. Most disubstituted aryl analogues (**3af**–**3an**) remained potent (IC_50_ = 0.013–1.13 μM), with the exception of **3ak** (IC_50_ = 12.06 μM), further illustrating the detrimental effect of excessive steric bulk on activity.

Because lipid ROS accumulation is a hallmark of ferroptosis, intracellular lipid ROS levels were quantified by flow cytometry. Compounds **3b** and **3c** induced robust, dose-dependent lipid ROS accumulation (Figure 3A, B). Moreover, **3b** and **3c** exhibited excellent potency for ferroptosis induction compared with the classic ferroptosis inducers RSL3 and erastin (Figure 3C), and their effects were abrogated by the ferroptosis inhibitor liproxstatin-1 (Figure 3D). Consistently, IC_50_ comparisons indicated that **3b** and **3c** were approximately 500–700-fold more potent than RSL3 and erastin (Figure 3E), establishing these compounds as highly potent and selective ferroptosis inducers. Moreover, the cytotoxicity of **3b**/**3c** against 3T3 cells showed the IC_50_ values of the two compounds in 3T3 cells were 0.0285 ± 0.0090 μM (**3b**) and 0.0653 ± 0.0099 μM (**3c**) respectively, which suggested that **3b**/**3c** exhibited 4-fold selectivity to cancer cells (Table S2). Taken together, these results indicated **3b**/**3c** were specific ferroptosis inducers with relatively high safety profile.

**Figure 3:**
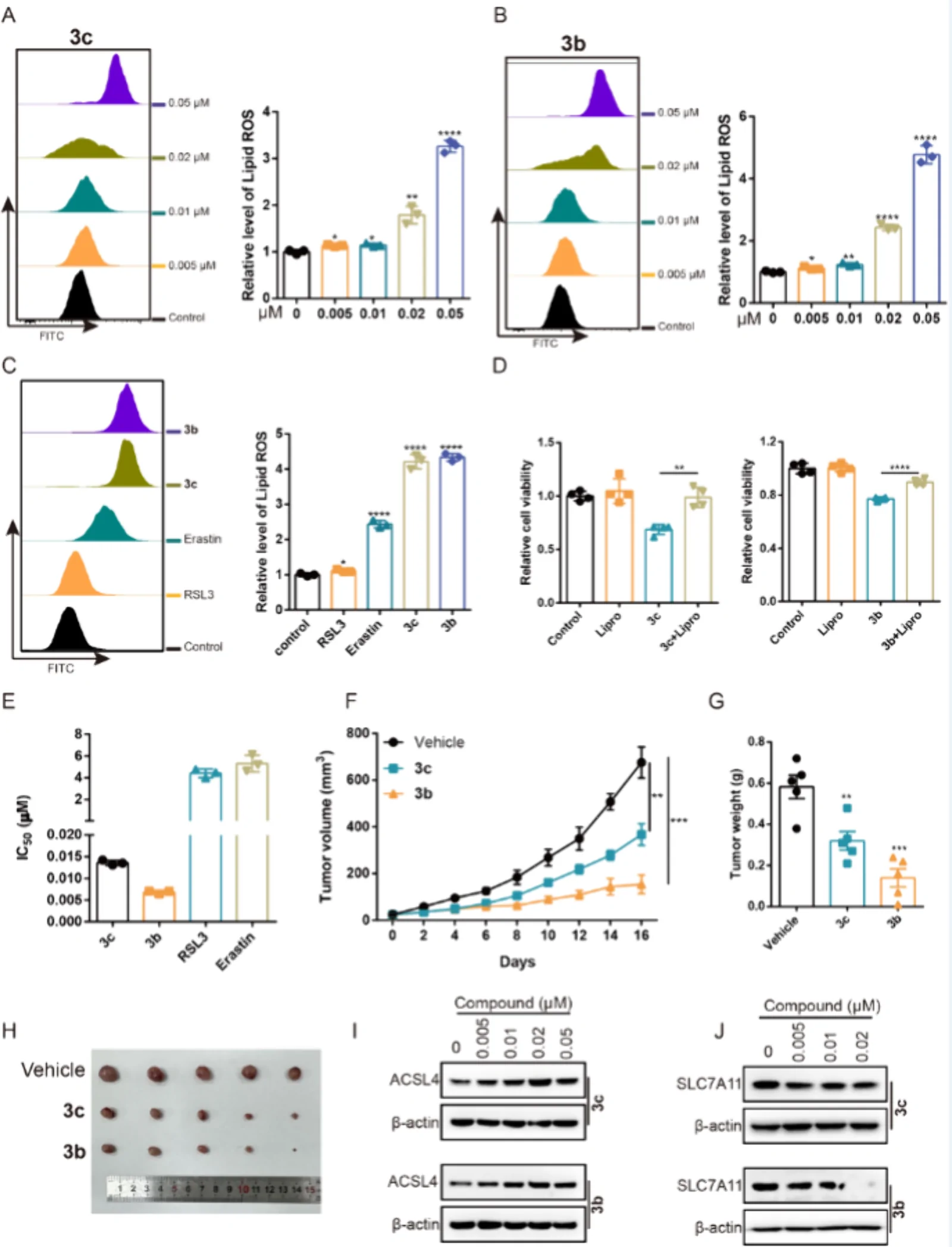
(A) Representative images and statistical results of intracellular lipid ROS accumulation after the treatment of **3c** for 48 h in NCI-H820 cells. (B) Representative images and statistical results of intracellular lipid ROS accumulation after the treatment of **3b** for 48 h in NCI-H820 cells. (C) Representative images and statistical results of intracellular lipid ROS accumulation after the treatment of RSL3 (2 μM), Erastin (20 μM), **3c** (0.05 μM) and **3b** (0.05 μM), respectively, for 48 h in NCI-H820 cells. (D) The percentage of cell viability after the coincubation with Lipro and subsequent treatment with **3b** and **3c**. (E) The IC_50_ comparison of **3b**, **3c**, RSL3 and Erastin. (F) The tumor volume after administrated with **3b** and **3c** respectively in lung adenocarcinoma PDX tumor. (G, H) The tumor pictures and tumor weight after administrated with **3b** and **3c** respectively in lung adenocarcinoma PDX tumor. (I, J) The protein level of ACSL4 and SLC7A11 after the treatment of **3b** and **3c** for 48 h in NCI-H820 cells.

To identify the direct target of **3b** and **3c**, we performed a systematic target prediction analysis using multiple online target prediction platforms, including AmIActive (AIA), SwissTargetPrediction and bSDTNBI. From each database, we selected the top 100 predicted targets ranked by prediction score. Subsequently, we identified the overlapping targets among the three prediction lists to determine consensus targets (Figure S3A,C). Based on these results, PARP1 protein was predicted by all databases and is closely associated with ferroptosis, suggesting that PARP1 might be the direct target of **3b** and **3c**. To further investigate the interaction between **3b**/**3c** and PARP1, molecular docking studies were performed. The docking results showed that **3b** formed three hydrogen bonds with PARP1 residues, including Ser-983 and His-937 (via different side-chain atoms), as well as hydrophobic interactions with Ala-755 and Met-890. These results suggested that **3b** exhibited strong binding affinity for PARP1 (binding energy: – 5.86 kcal/mol, Figure S3B). Similarly, the docking results showed that **3c** formed four hydrogen bonds with PARP1 residues, including Ser-983, Ala-935, and His-937 (via different side-chain atoms), as well as hydrophobic interactions with Ala-755 and Met-890. These results suggested that **3c** exhibited strong binding affinity for PARP1 (binding energy: −6.75 kcal/mol, Figure S3C). Collectively, the prediction results suggest that compounds **3b** and **3c** may bind directly to PARP1 through noncovalent interactions. Moreover, we will synthesize molecular probes for further target identification in future studies.

In addition, to evaluate the *in vivo* toxicity of **3b** and **3c**, Bal b/c mice were intraperitoneally administered **3b** or **3c** at a dose of 25 mg/kg. At this dose, no significant changes in body weight, behavioral abnormalities, or significant alterations in the organ coefficients of the heart, liver, spleen, lungs, kidneys, or brain were observed after 72 h (Figure S2A,B). Furthermore, no significant changes were observed in serum aspartate aminotransferase (AST) or creatinine (CR) levels, indicating that **3b** and **3c** did not cause severe hepatic or renal toxicity at 25 mg/kg (Figure S2C,D). These results suggested that **3b** and **3c** exhibited no significant *in vivo* toxicity. To assess *in vivo* efficacy, a lung cancer PDX mouse model was established, and compounds **3b** or **3c** were administered intraperitoneally every 3 days. Treatment with **3b** (5 mg/kg) or **3c** (10 mg/kg) significantly reduced tumor volume and tumor weight (Figure 3F–H). Notably, **3b** produced superior tumor suppression relative to **3c**, positioning **3b** as a particularly promising lead for further development. Preliminary mechanism investigations revealed that compounds **3b** and **3c** induced ferroptosis through upregulation of ACSL4 and concomitant downregulation of SLC7A11 (Figure 3I,J).

In summary, we developed a programmed cascade cyclization featuring remote cyano group migration to access cyanamide-bearing 3,4-dihydro-2*H*-1,4-thiazines as selective ferroptosis inducers. This strategy employs nontoxic, inexpensive KSCN as a practical CN equivalent to construct cyanamide-bearing 1,4-thiazines through a cascade sequence involving SCN installation, remote S → N cyano migration, and intramolecular cyclization, while featuring operational simplicity, broad substrate scope, and excellent functional group tolerance. The resulting scaffold class exhibits outstanding anticancer potency by inducing ferroptosis, and lead compounds **3b** and **3c** show remarkable efficacy in a lung cancer PDX model. Collectively, these findings provide both a chemically enabling approach to cyanamides from benign feedstocks and a high-value entry to next-generation ferroptosis-based anticancer leads.

## Supplementary Material



## Notes

Experimental details, characterization data, copies of NMR spectra, and X-ray crystallographic data collection and structural parameters of compound **3a** (PDF)

## Notes

CCDC 2497077 contain the supplementary crystallographic data for this paper. These data can be obtained free of charge via www.ccdc.cam.ac.uk/data_request/cif, or by emailing data_request@ccdc.cam.ac.uk, or by contacting The Cambridge Crystallographic Data Centre, 12 Union Road, Cambridge CB2 1EZ, UK; fax: +44 1223 336033.
