# Supplementary material for: Programmed S → N Remote Cyano Migration: A Thiocyanate-Based Entry to Cyanamides and Selective Ferroptosis Inducers
Source: ACS Cent Sci. 2026 Aug 6;12(8):1237–45. doi: 10.1021/acscentsci.6c00645 (PMC13523506; doi:10.1021/acscentsci.6c00645)

Supporting Information

Programmed S→N Remote Cyano Migration: A Thiocyanate-Based Entry to Cyanamides and Selective Ferroptosis Inducers

Xiyan Duan,[1*‡] Wanjing Feng,[2‡] Fengjiao Du,[1‡] Can Liu,[2] Ning Liu,[4] Yihao Zhao,[1] Kun Liu,[1] Pu Liu,[5] Yixuan Zhang,[1] Jingze Li,[1] Yahui Ding,[3*] and Quan Zhang[2*]

[1]School of Chemistry & Chemical Engineering, Henan University of Science and Technology, Luoyang 471003, Henan, China

[2]College of Pharmacy, State Key Laboratory of Medicinal Chemical Biology and Tianjin Key Laboratory of Molecular Drug Research, Nankai University, Tianjin 300350, China

[3]College of Chemistry, State Key Laboratory of Medicinal Chemical Biology, Nankai University, 94 Weijin Road, Tianjin 300071, China

[4]School of Nursing, Henan University of Science and Technology, Luoyang 471003, Henan, People's Republic of China

[5]College of Food & Bioengineering, Henan University of Science and Technology, Luoyang 471003, Henan, People's Republic of China

E-mail: 017095@nankai.edu.cn; E-mail: duanxiyan@tju.edu.cn ; E-mail: zhangquan@nankai.edu.cn

Contents

General Information 2

(a) Optimization of the Reaction Conditions 3

(b) KIE Experiment 4

(c) General Experimental Procedure A for the synthesis of N-propargyl enaminones 5

(d) Procedure B for the synthesis of thiocyanated enaminones 2 33

(e) General procedure C for the synthesis of 3,4-dihydro-2H-1,4-thiazines 3a-3an 34

(f) Biological Assessment of 3,4-Dihydro-2H-1,4-thiazines 56

(g) X-Ray crystallographic studies 61

(h) Details of Calculation and Discussion 63

(i) Reference 84

(j) NMR Spectra 86

General Information

All reagents and solvent were commercially available with analytical grade and used as received. Yields refer to chromatographically and spectroscopically (^{1}H NMR) homogeneous materials, unless otherwise stated. The used solvents were purified and dried according to common procedures. High-resolution mass spectra (HRMS) were obtained with a FTICR-MS (Ionspec 7.0T) spectrometer. The ^{1}H, ^{13}C and ^{19}F NMR NMR spectra were recorded in $CDCl_3$ or DMSO-d_6 solution on a Bruker AV 400 MHz spectrometer. Chemical shifts are reported in parts per million (δ) relative to $CDCl_3$ (7.26 ppm) for ^{1}H NMR data and $CDCl_3$ (77.0 ppm) for ^{13}C NMR data or the peak of DMSO-d_6, defined at δ = 2.50 (^{1}H NMR) or δ = 39.5 (^{13}C NMR). The following abbreviations were used to explain multiplicities: *s* = singlet, *d* = doublet, *t* = triplet, *q* = quartet, *m* = multiplet, *br* = broad). Flash column chromatography was performed over silica gel 200−300 mesh, and the eluent was a mixture of ethyl acetate (EA) and petroleum ether (PE). Procedures involving air- or moisture-sensitive materials were conducted with anhydrous solvents under an inert atmosphere of nitrogen or argon using standard Schlenk techniques. The synthesis of ynones substrates was according to reference[1] and detailed characterization data was also included.

(a) Optimization of the Reaction Conditions

We started the optimization of conditions using thiocyanated enaminone **2a** as model substrates (Table 1). After a systematic investigation, **3a** was obtained in 76% isolated yield by using NaOH as a base (entry 1). Other bases displayed inferior performance (entries 2−10). The reaction solvent had a profound impact on efficiency, as exemplified by the drastically decreased yields or no reaction when DMF was replaced with other solvents such as DMSO or DMA (entries 11 and 12). When the reaction was carried out at higher temperature (60, or 100 °C), the efficiency of this transformation decreased significantly (entries 13−14). Control experiments showed base was critical to afford the 3,4-dihydro-2H-1,4-thiazine **3a** (entry 15). However, a 65% yield of **3a** was obtained without 15-crown-5 (entry 16), indicating that although the 15-crown-5 was not indispensable, it was crucial for achieving a high yield. We envisioned that a one-pot cascade reaction of the in-situ-formed thiocyanated enaminones would provide an alternative approach for the efficient construction of 3,4-dihydro-2H-1,4-thiazines. To assess the feasibility of the one-pot process, we selected **1a** as the model substrate (entry 17). After careful optimization of the reaction conditions, the desired 3,4-Dihydro-2H-1,4-thiazine **3a** was obtained through the following one-pot procedure with a 71% isolated yield (entry 17).

Table S1. Optimization of the Reaction Conditions

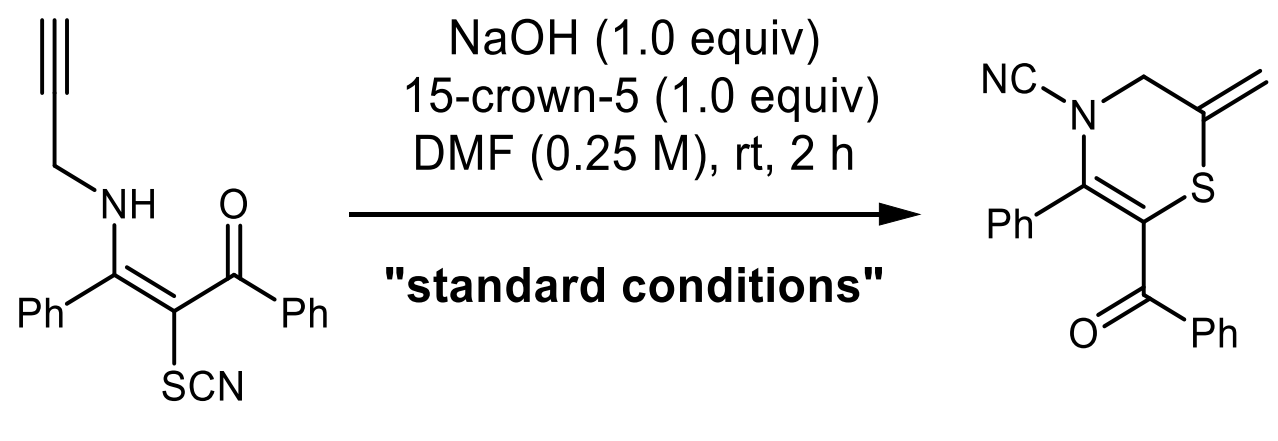

2a 3a

entry	variation from "Standard conditions"[a]	yield **3a** (%)[b]
1	none	83 (76[c])
2[d]	NaH instead of NaOH	51
3[d]	LiOH instead of NaOH	55
4[d]	KOH instead of NaOH	61

5 [d]	tBuOK instead of NaOH	60
6 [d]	NaHMDS instead of NaOH	32
7 [d]	LiHMDS instead of NaOH	0
8 [d]	KHMDS instead of NaOH	49
9 [d]	Na_2CO_3 instead of NaOH	0
10 [d]	Cs_2CO_3 instead of NaOH	0
11	DMSO instead of DMF	31
12	DMA instead of DMF	0
13	60 °C	45
14	100 °C	Trace
15	w/o NaOH	n.d
16	w/o 15-crown-5	65
17 [e]	One-pot procedure	76 (71 [f])

[a] *Standard conditions*: compound **2a** (0.2 mmol, 1.0 equiv), NaOH (0.2 mmol, 1.0 equiv), 15-crown-5 (0.2 mmol, 1.0 equiv), DMF (0.8 mL, 0.25 M), rt, 2 h. [b] ^{1}H NMR yield using 1,3,5-trimethoxybenzene as the internal standard. [c] Isolated yield. [d] without 15-crown-5. [e] Reaction conditions: Step 1: **1a** (0.2 mmol, 1.0 equiv), DBDMH (0.1 mmol, 0.5 equiv), DMF (0.2 mL), 60 °C, 2h; then, KSCN (0.2 mmol, 1.0 equiv), DMF (0.4 mL), 2h, work-up. Step 2: Reaction residue of step 1, NaOH (0.2 mmol, 1.0 equiv), 15-crown-5 (0.2 mmol, 1.0 equiv), DMF (0.8 mL, 0.25 M), rt, 2 h. [f] Isolated yields for two steps.

(b) KIE Experiment

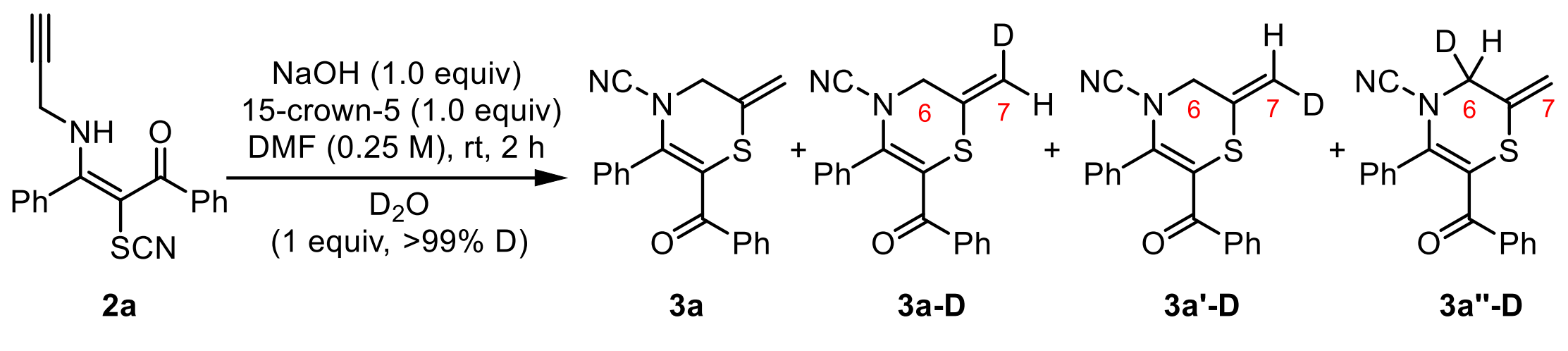

Scheme S1

Compound **2a** (1 mmol) was dissolved in DMF (2 mL), NaOH (1 mmol), 15-crown-5 (1 mmol) and deuterated water (D_2O, 99.96% *d*-content) were subsequently added. The reaction mixture was stirred at room temperature for 2 hours. Upon completion, ethyl acetate (20 mL) was added, and the mixture was washed three times with saturated brine. The organic phase was separated, dried over anhydrous sodium

sulfate, filtered, and concentrated to yield the crude product. The crude product was further purified by flash column chromatography to afford the inseparable compounds **3a**, **3a-D**, **3a′-D** and **3a″-D**. ^{1}H NMR (400 MHz, $CDCl_3$): δ = 7.58–7.54 (m, 2H), 7.33–7.27 (m, 1H), 7.25–7.22 (m, 2H), 7.20–7.12 (m, 5H), 5.56 (d, J = 1.2 Hz, 0.61 H), 5.55 (s, 0.20H), 5.39 (d, J = 1.1 Hz, 0.58H), 5.38 (d, J = 1.1 Hz, 0.20H), 4.39 (s, 1.42H), 4.38 (s, 0.34H). HRMS (ESI): m/z $[M + H]^+$ of **3a-D** calcd for: $C_{19}H_{14}DN_2OS$: 321.0968; found: 320.0974.

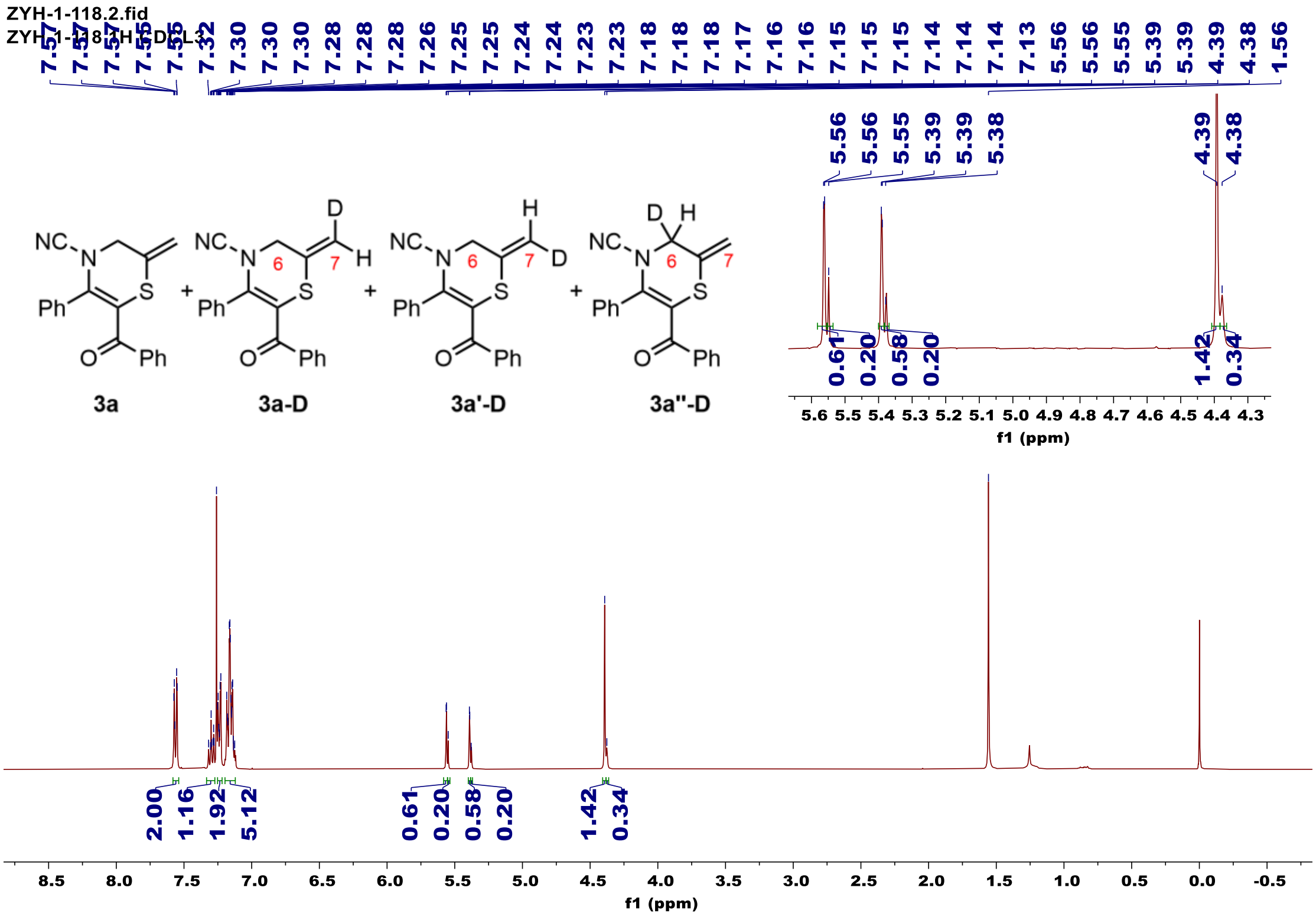

Figure S1 ^{1}H NMR spectrum of compounds 3a, 3a-D, 3a′-D and 3a″-D (400 MHz, $CDCl_3$)

(c) General Experimental Procedure A for the synthesis of N-propargyl enaminones

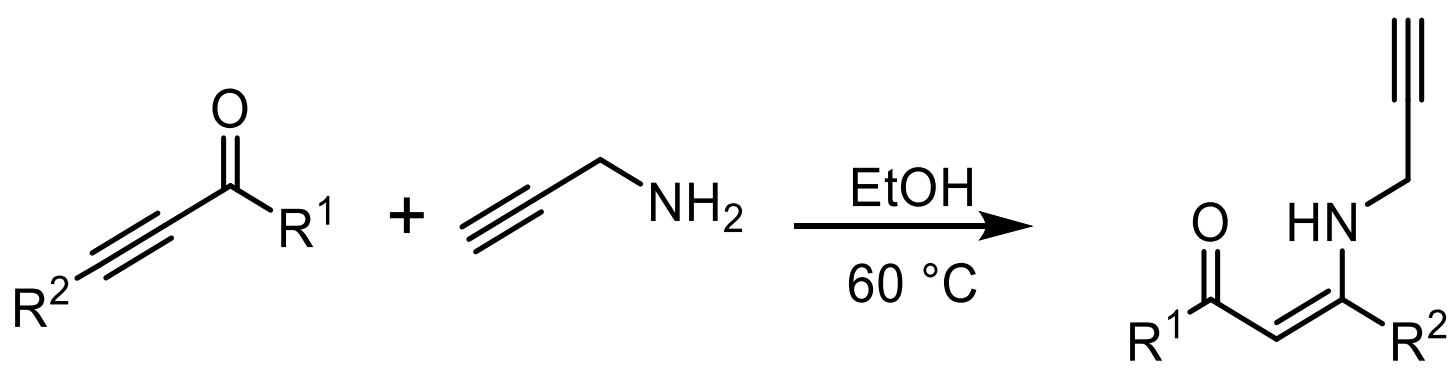

A mixture of *prop-2-yn-1-amine* (12 mmol), *propynones* (10 mmol), and anhydrous EtOH (10 mL) was stirred at 60 °C in air for 24 hours. Once the reaction was complete (monitored by TLC), the solvent was removed under reduced pressure, and the residue was purified by column chromatography (silica gel, eluted with a petroleum ether/ethyl acetate mixture) to yield N-propargyl enaminones.

Synthesis of 1a

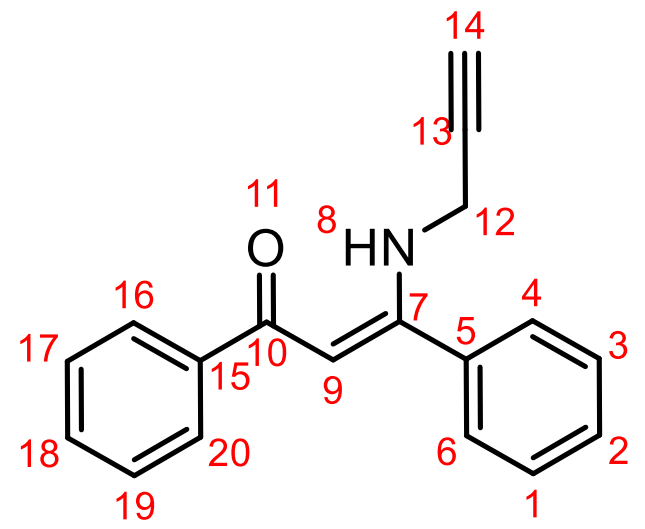

(Z)-1,3-diphenyl-3-(prop-2-yn-1-ylamino)prop-2-en-1-one[2] (**1a**). General procedure A was followed and purification by flash column chromatography afforded **1a** as a yellow solid (PE: EA = 7:1). Yield: 2.3 g, 86%; mp: 77–79 ºC. ^{1}H NMR (400 MHz, $CDCl_3$): δ = 11.33 (s, 1H), 7.92–7.90 (m, 2H), 7.49–7.40 (m, 8H), 5.85 (s, 1H), 3.94 (dd, J = 6.3, 2.5 Hz, 2H), 2.31 (t, J = 2.5 Hz, 1H). ^{13}C NMR (101 MHz, $CDCl_3$): δ = 189.0, 165.7, 139.8, 134.7, 130.9, 129.7, 128.6, 128.1, 127.7, 127.0, 94.5, 79.7, 72.4, 34.1.

Synthesis of 1b

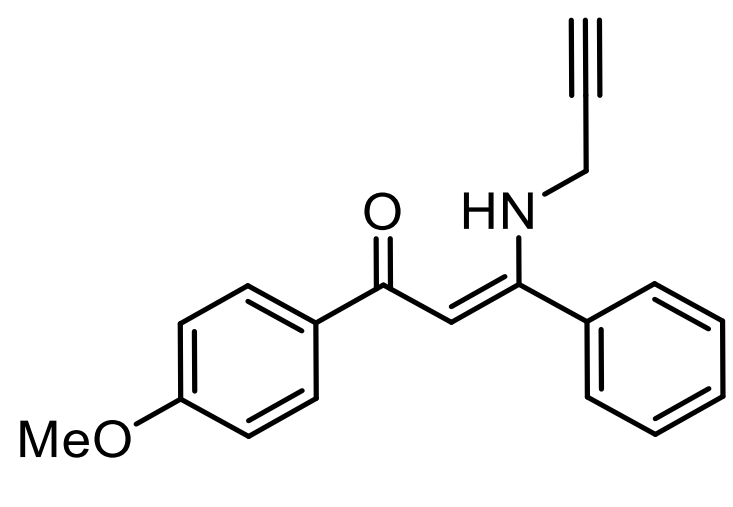

(Z)-1-(4-methoxyphenyl)-3-phenyl-3-(prop-2-yn-1-ylamino)prop-2-en-1-one[2] (**1b**). General procedure A was followed and purification by flash column chromatography afforded **320** as a brown solid (PE: EA = 40:1 to 7:1). Yield: 2.3 g, 80%. mp: 80–83 ºC. ^{1}H NMR (400 MHz, $CDCl_3$): δ = 11.21 (s, 1H), 7.92–7.86 (m, 2H), 7.48 (q, J = 3.0 Hz, 5H), 6.90 (d, J = 8.9 Hz, 2H), 5.81 (s, 1H), 3.92 (dd, J = 6.3, 2.5 Hz, 2H), 3.84 (s, 3H),

2.29 (s, 1H). ^{13}C NMR (101 MHz, $CDCl_3$): δ = 188.3, 165.3, 162.0, 135.1, 132.6, 129.7, 129.1, 128.6, 127.8, 113.4, 94.3, 79.9, 72.3, 55.3, 34.2.

Synthesis of 1c

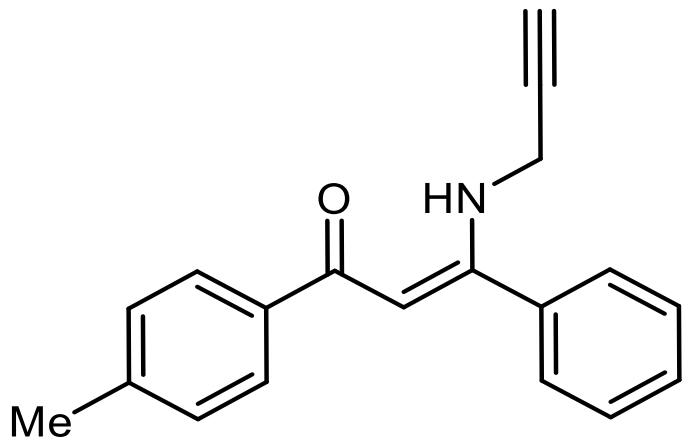

(Z)-3-phenyl-3-(prop-2-yn-1-ylamino)-1-(p-tolyl)prop-2-en-1-one[3] (**1c**). General procedure A was followed and purification by flash column chromatography afforded **1c** as a yellow solid (PE: EA = 7:1). Yield: 2.2 g，81%; mp: 80–81 ºC. ^{1}H NMR (400 MHz, $CDCl_3$): δ = 11.33 (s, 1H), 7.82 (d, J = 8.2 Hz, 2H), 7.52–7.39 (m, 5H), 7.19 (d, J = 7.9 Hz, 2H), 5.84 (s, 1H), 3.90 (dd, J = 6.3, 2.5 Hz, 2H), 2.35 (s, 3H), 2.31 (t, J = 2.5 Hz, 1H). ^{13}C NMR (101 MHz, $CDCl_3$): δ = 188.6, 165.3, 141.1, 136.9, 134.7, 129.5, 128.7, 128.4, 127.6, 127.0, 94.3, 79.6, 72.2, 33.9, 21.2.

Synthesis of 1d

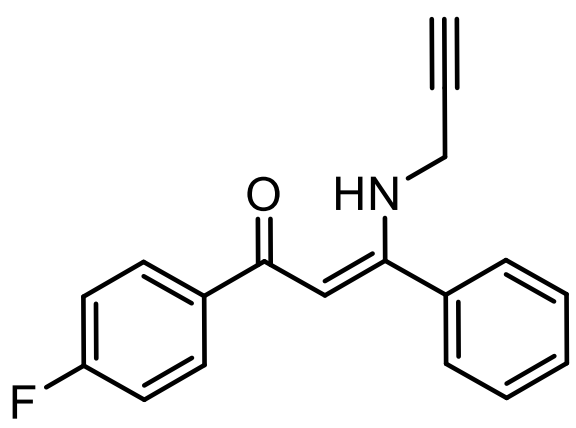

(Z)-1-(4-fluorophenyl)-3-phenyl-3-(prop-2-yn-1-ylamino)prop-2-en-1-one[3] (**1d**). General procedure A was followed and purification by flash column chromatography afforded **1d** as a brown solid (PE: EA = 7:1). Yield: 2.2 g, 80%; mp: 107–109 ºC. ^{1}H NMR (400 MHz, $CDCl_3$): δ = 11.28 (s, 1H), 8.11–7.70 (m, 2H), 7.48 (d, J = 2.2 Hz, 5H), 7.07 (t, J = 8.7 Hz, 2H), 5.78 (s, 1H), 3.94 (dd, J = 6.3, 2.5 Hz, 2H), 2.31 (s, 1H). ^{13}C NMR (101 MHz, $CDCl_3$): δ = 187.6, 166.0, 164.6 (d, J_{C-F} = 250 Hz), 136.1, 134.8, 129.9, 129.4 (d, J_{C-F} = 9 Hz), 128.7, 127.8, 115.1 (d, J_{C-F} = 21 Hz), 94.2, 79.7, 72.5, 34.21.

Synthesis of 1e

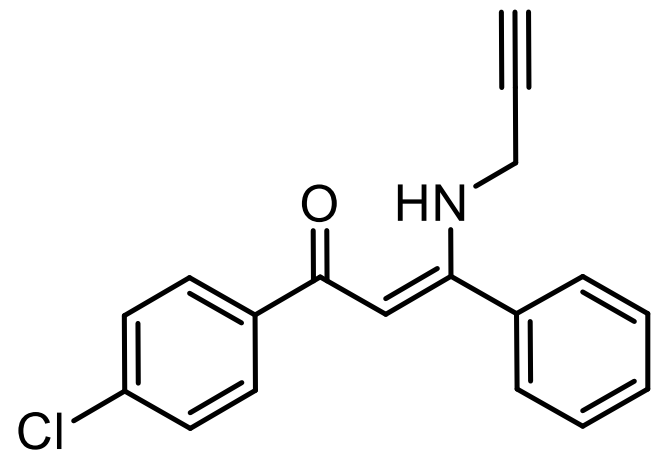

(Z)-1-(4-chlorophenyl)-3-phenyl-3-(prop-2-yn-1-ylamino)prop-2-en-1-one[2] (**1e**). General procedure A was followed and purification by flash column chromatography afforded **1e** as a yellow solid (PE: EA = 40:1 to 7:1). Yield: 2.5 g, 85%; mp: 125–126 ºC. ^{1}H NMR (400 MHz, $CDCl_3$): δ = 11.33 (s, 1H), 7.86–7.81 (m, 2H), 7.48 (s, 5H), 7.37 (d, J = 8.6 Hz, 2H), 5.78 (s, 1H), 3.95 (dd, J = 6.3, 2.5 Hz, 2H), 2.31 (t, J = 4 Hz, 1H). ^{13}C NMR (101 MHz, $CDCl_3$): δ = 187.6, 166.2, 138.3, 137.1, 134.7, 130.0, 128.8, 128.6, 128.4, 127.8, 94.2, 79.6, 72.6, 34.3.

Synthesis of 1f

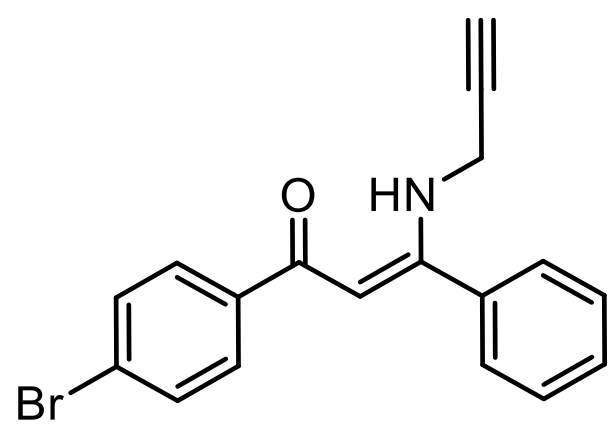

(Z)-1-(4-bromophenyl)-3-phenyl-3-(prop-2-yn-1-ylamino)prop-2-en-1-one[4] (**1f**). General procedure A was followed and purification by flash column chromatography afforded **1f** as a yellow solid (PE: EA = 7:1). Yield: 2.2 g, 65%; mp: 135–136 ºC. ^{1}H NMR (400 MHz, $CDCl_3$): δ = 11.33 (s, 1H), 7.76 (d, J = 8.6 Hz, 2H), 7.53 (d, J = 8.6 Hz, 2H), 7.48 (s, 5H), 5.78 (s, 1H), 3.95 (dd, J = 6.3, 2.5 Hz, 2H), 2.31 (t, J = 2.5 Hz, 1H). ^{13}C NMR (101 MHz, $CDCl_3$): δ = 187.7, 166.3, 138.7, 134.7, 131.4, 130.0, 128.8, 127.8, 125.7, 94.2, 79.5, 77.2, 72.6, 34.3.

Synthesis of 1g

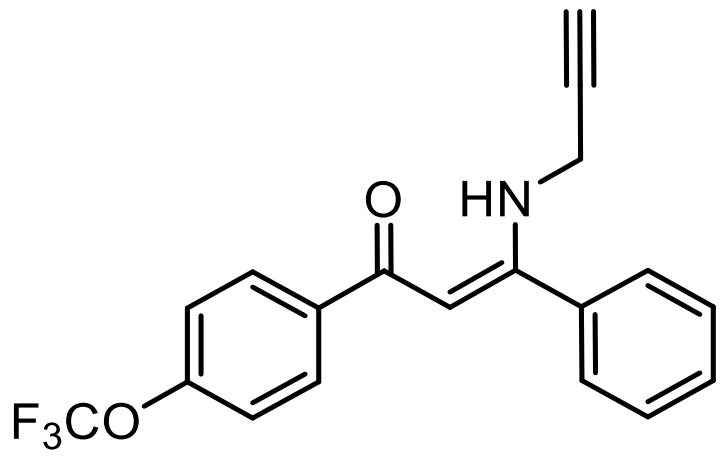

(Z)-3-phenyl-3-(prop-2-yn-1-ylamino)-1-(4-(trifluoromethoxy)phenyl)prop-2-en-1-one (**1g**). General procedure A was followed and purification by flash column chromatography afforded **1g** as a brown solid (PE: EA = 7:1). Yield: 3.07 g, 89%; Mp: 100–103 ºC. ^{1}H NMR (400 MHz, $CDCl_3$): δ = 11.33 (d, J = 6.9 Hz, 1H), 7.96–7.91 (m, 2H), 7.48 (s, 5H), 7.23 (dd, J = 9.0, 1.1 Hz, 2H), 5.79 (s, 1H), 3.95 (dd, J = 6.3, 2.6 Hz, 2H), 2.32 (t, J = 2.5 Hz, 1H).^{13}C NMR (101 MHz, $CDCl_3$): δ = 87.3, 166.3, 151.1, 138.3, 134.6, 123.0, 128.9, 128.7, 127.7, 120.2, 94.2, 79.5, 72.6, 34.2. HRMS (ESI): m/z $[M + H]^+$ calcd for: $C_{19}H_{15}NO_2F_3$: 346.1055; found: 346.1058.

Synthesis of 1h

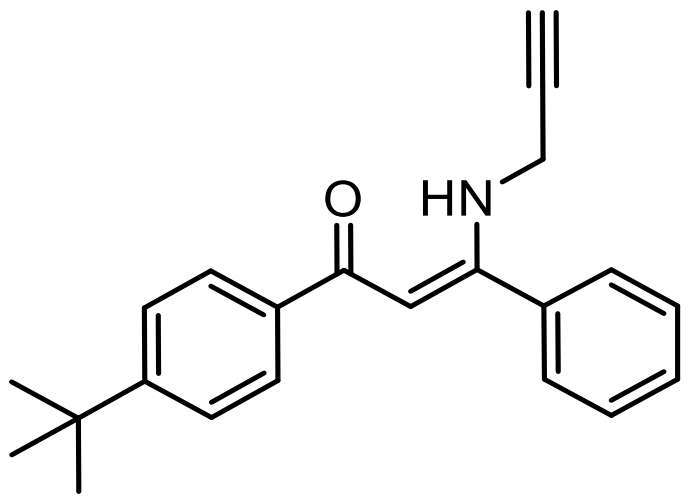

(Z)-1-(4-(tert-butyl)phenyl)-3-phenyl-3-(prop-2-yn-1-ylamino)prop-2-en-1-one[3] (**1h**). General procedure A was followed and purification by flash column chromatography afforded **1h** as a brown oil (PE: EA = 7:1). Yield: 2.6 g，82%. ^{1}H NMR (400 MHz, $CDCl_3$): δ = 11.28 (s, 1H), 7.84 (d, J = 8.6 Hz, 2H), 7.48 (t, J = 2.4 Hz, 5H), 7.42 (d, J = 8.6 Hz, 2H), 5.84 (s, 1H), 3.93 (dd, J = 6.3, 2.5 Hz, 2H), 2.30 (s, 1H), 1.33 (s, 9H). ^{13}C NMR (101 MHz, $CDCl_3$): δ = 189.1, 165.5, 154.4, 137.2, 135.0, 129.7, 128.6, 127.9, 127.0, 125.2, 94.7, 79.8, 72.3, 34.8, 34.2, 31.2.

Synthesis of 1j

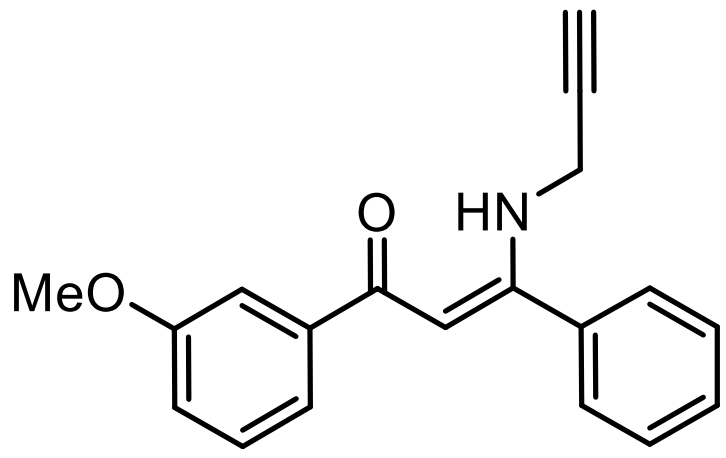

(Z)-1-(3-methoxyphenyl)-3-phenyl-3-(prop-2-yn-1-ylamino)prop-2-en-1-one[3] (**1j**). General procedure A was followed and purification by flash column chromatography afforded **1j** as a yellow oil (PE: EA = 8:1). Yield: 2.8 g, 97%. ^{1}H NMR (400 MHz, $CDCl_3$): δ = 11.32 (s, 1H), 7.53–7.43 (m, 7H), 7.30 (t, J = 7.9 Hz, 1H), 7.03–6.96 (m, 1H), 5.83 (s, 1H), 3.94 (dd, J = 6.3, 2.5 Hz, 2H), 3.85 (s, 3H), 2.31 (t, J = 2.5 Hz, 1H). ^{13}C NMR (101 MHz, $CDCl_3$): δ = 188.7, 165.8, 159.6, 141.4, 134.8, 129.8, 129.1, 128.7, 127.8, 119.6, 117.4, 111.7, 94.7, 79.7, 72.5, 55.3, 34.2.

Synthesis of 1k

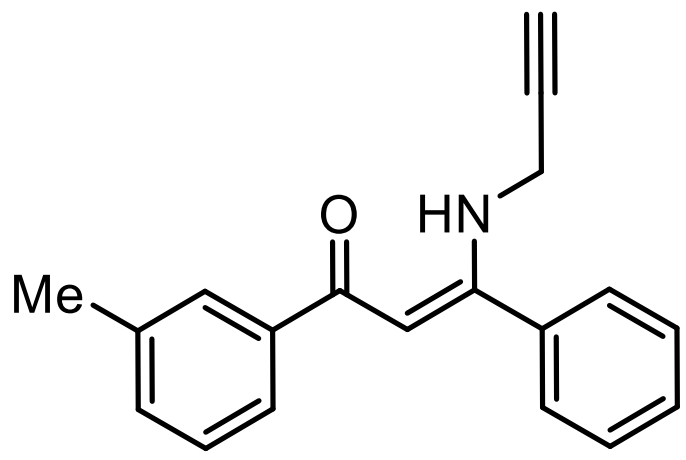

(Z)-3-phenyl-3-(prop-2-yn-1-ylamino)-1-(m-tolyl)prop-2-en-1-one (**1k**)[5]. General procedure A was followed and purification by flash column chromatography afforded **1k** as brown oil (PE: EA = 7:1). Yield: 2.1 g, 75%. ^{1}H NMR (400 MHz, $CDCl_3$): δ = 11.30 (s, 1H), 7.73–7.67 (m, 2H), 7.51–7.45 (m, 5H), 7.27-7.25 (m, 2H), 5.83 (s, 1H), 3.94 (dd, J = 6.3, 2.5 Hz, 2H), 2.39 (s, 3H), 2.30 (t, J = 2.5 Hz, 1H). ^{13}C NMR (101 MHz, $CDCl_3$): δ = 189.4, 165.7, 139.9, 137.9, 134.9, 131.8, 129.8, 128.7, 128.1, 127.9, 127.8, 124.3, 94.8, 79.8, 72.4, 34.2, 21.4.

Synthesis of 1l

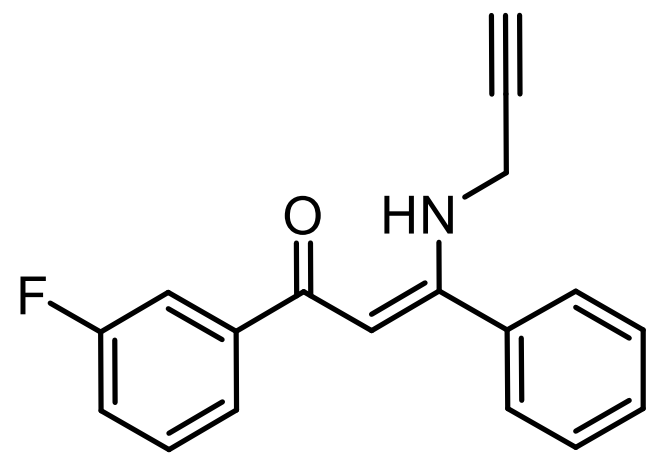

(Z)-1-(3-fluorophenyl)-3-phenyl-3-(prop-2-yn-1-ylamino)prop-2-en-1-one[3] (**1l**). General procedure A was followed and purification by flash column chromatography afforded **1l** as a white solid (PE: EA = 10:1). Yield: 2.5 g, 89%. Mp: 106–108 ºC. ^{1}H NMR (400 MHz, $CDCl_3$): δ = 11.34 (s, 1H), 7.66 (d, J = 7.8 Hz, 1H), 7.62–7.56 (m, 1H), 7.55–7.43 (m, 5H), 7.40–7.33 (m, 1H), 7.19–7.08 (m, 1H), 5.78 (s, 1H), 3.96 (dd, J = 6.3, 2.5 Hz, 2H), 2.32 (t, J = 4 Hz 1H). ^{13}C NMR (101 MHz, $CDCl_3$): δ = 187.3, 166.4, 162.8 (d, $J_{C\text{-}F}$ = 245 Hz), 142.3, 134.6, 129.9, 129.7 (d, $J_{C\text{-}F}$ = 7 Hz), 128.7, 127.8, 122.8, 117.7, 114.0 (d, $J_{C\text{-}F}$ = 22 Hz), 94.4, 79.5, 72.6, 34.3.

Synthesis of 1m

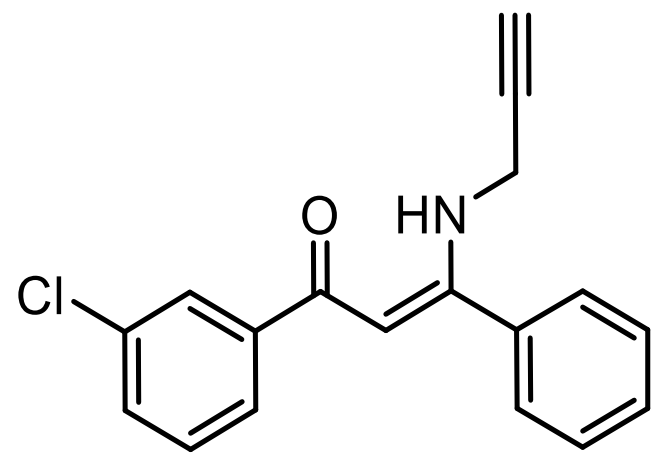

(Z)-1-(3-chlorophenyl)-3-phenyl-3-(prop-2-yn-1-ylamino)prop-2-en-1-one[3] (**1m**). General procedure A was followed and purification by flash column chromatography afforded **1m** as a yellow solid (PE: EA = 7:1). Yield: 2.5 g, 84%; mp：100–103 ºC. ^{1}H NMR (400 MHz, $CDCl_3$): δ = 11.34 (s, 1H), 7.87 (t, J = 1.7 Hz, 1H), 7.80–7.73 (m, 1H), 7.48 (s, 5H), 7.43–7.38 (m, 1H), 7.33 (t, J = 7.8 Hz, 1H), 5.77 (s, 1H), 3.96 (dd, J = 6.3, 2.5 Hz, 2H), 2.32 (t, J = 2.5 Hz, 1H). ^{13}C NMR (101 MHz, $CDCl_3$): δ = 187.3, 166.5, 141.7, 134.6, 134.4, 130.9, 130.0, 129.6, 128.8, 127.8, 127.4, 125.3, 94.4, 79.6, 72.7, 34.3.

Synthesis of 1n

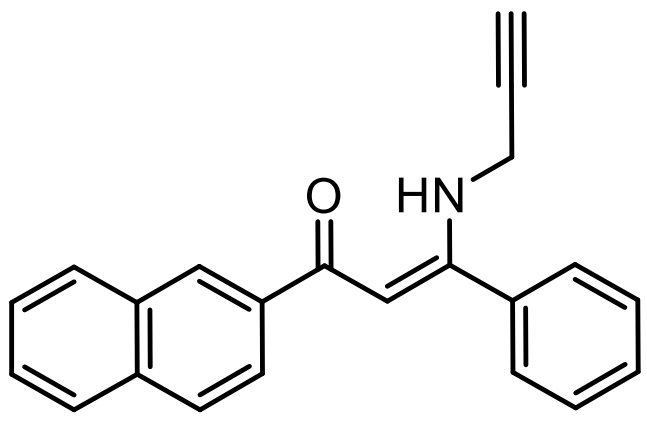

(Z)-1-(naphthalen-2-yl)-3-phenyl-3-(prop-2-yn-1-ylamino)prop-2-en-1-one (**1n**)[6]. General procedure A was followed and purification by flash column chromatography afforded **1n** as a brown solid (PE: EA = 40:1to 7:1). Yield: 88%; mp: 63–66 ºC. ^{1}H NMR (400 MHz, $CDCl_3$): δ = 11.40 (s, 1H), 8.41 (s, 1H), 8.02 (dd, J = 8.6, 1.7 Hz, 1H), 7.93–7.83 (m, 3H), 7.63–7.41 (m, 7H), 6.01 (s, 1H), 3.98 (dd, J = 6.3, 2.5 Hz, 2H), 2.33 (t, J = 4 Hz,1H).^{13}C NMR (101 MHz, $CDCl_3$): δ = 188.9, 165.9, 137.2, 135.0, 134.7, 132.8, 129.8, 129.2, 128.7, 128.0, 127.9, 127.7, 127.6, 127.3, 126.3, 124.1, 94.8, 79.7, 72.5, 34.3.

Synthesis of 1o

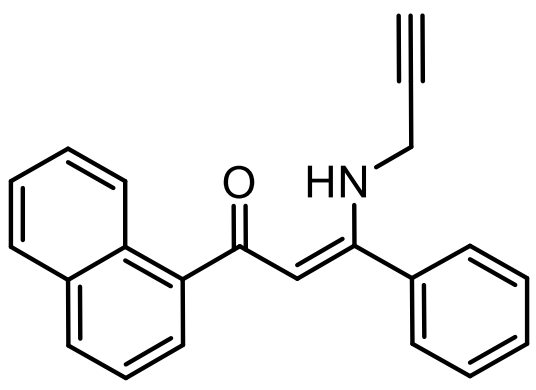

(Z)-1-(naphthalen-1-yl)-3-phenyl-3-(prop-2-yn-1-ylamino)prop-2-en-1-one[7] (**1o**). General procedure A was followed and purification by flash column chromatography afforded **1o** as brown oil (PE: EA = 20:1). Yield: 2.9 g, 93%. ^{1}H NMR (400 MHz, $CDCl_3$): δ = 11.34 (s, 1H), 8.54 (d, J = 8.3 Hz, 1H), 7.89-7.83 (m, 2H), 7.69 (dd, J = 7.1, 1.1 Hz, 1H), 7.56–7.50 (m, 4H), 7.48–7.44 (m, 4H), 5.66 (s, 1H), 4.01 (dd, J = 6.4, 2.5 Hz, 2H), 2.36 (t, J = 4 Hz, 1H). ^{13}C NMR (101 MHz, $CDCl_3$): δ = 193.4, 165.6, 139.8, 134.6, 133.8, 130.2, 130.0, 129.9, 128.7, 128.2, 127.9, 126.6, 126.1, 125.9, 125.6, 124.8, 99.2, 79.8, 72.6, 34.3.

Synthesis of 1p

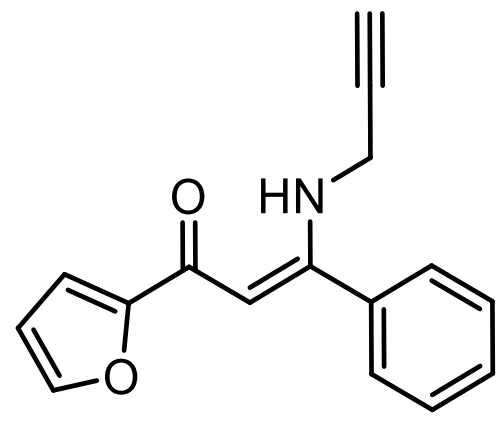

(Z)-1-(furan-2-yl)-3-phenyl-3-(prop-2-yn-1-ylamino)prop-2-en-1-one[3] (**1p**). General procedure A was followed and purification by flash column chromatography afforded **1p** as a yellow solid (PE: EA = 7:1). Yield: 1.38 g, 55%; mp: 90–91 ºC. ^{1}H NMR (400 MHz, $CDCl_3$): δ = 10.98 (s, 1H), 7.47 (s, 6H), 7.04 (dd, J = 3.5, 0.7 Hz, 1H), 6.47 (dd, J = 3.5, 1.7 Hz, 1H), 5.77 (s, 1H), 3.92 (dd, J = 6.3, 2.5 Hz, 2H), 2.29 (t, J = 2.5 Hz, 1H). ^{13}C NMR (101 MHz, $CDCl_3$) : δ = 178.4, 166.0, 154.2, 144.4, 134.7, 129.9, 128.7, 127.8, 113.2, 111.9, 94.2, 79.7, 72.4, 34.3.

Synthesis of 1q

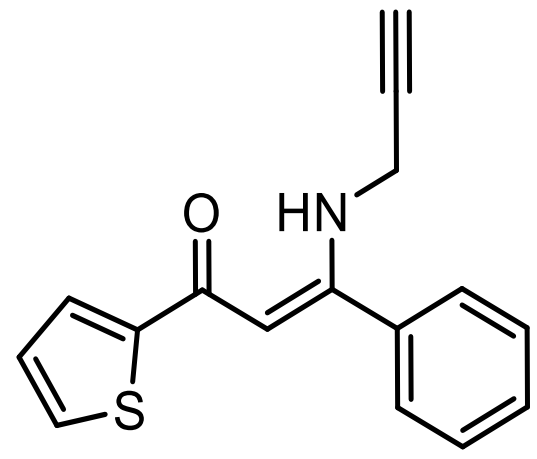

(Z)-3-phenyl-3-(prop-2-yn-1-ylamino)-1-(thiophen-2-yl)prop-2-en-1-one[3] (**1q**). General procedure A was followed and purification by flash column chromatography afforded **1q** as a yellow oil (PE: EA = 5:1). Yield: 2.6 g, 98%. Mp: 87–88 ºC. ^{1}H NMR (400 MHz, $CDCl_3$): δ = 10.96 (s, 1H), 7.58–7.55 (m, 1H), 7.48 (s, 6H), 7.06 (dd, J = 4.9, 3.8 Hz, 1H), 5.71 (s, 1H), 3.91 (dd, J = 6.3, 2.5 Hz, 2H), 2.29 (t, J = 2.5 Hz, 1H). ^{13}C NMR (101 MHz, $CDCl_3$): δ = 182.1, 165.5, 146.7, 134.7, 130.6, 129.9, 128.7, 128.1, 127.8, 127.7, 94.4, 79.6, 72.4, 34.2.

Synthesis of 1r

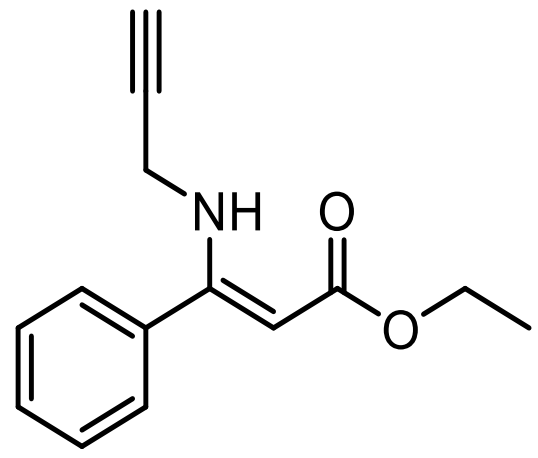

ethyl (Z)-3-phenyl-3-(prop-2-yn-1-ylamino)acrylate[8] General procedure A was followed and purification by flash column chromatography afforded **1r** as a brown oil (PE:EA = 15:1). Yield: 1.9 g, 85%. ^{1}H NMR (400 MHz, $CDCl_3$): δ = 8.58 (s, 1H), 7.40 (s, 5H), 4.71 (s, 1H), 4.15 (q, J = 7.1 Hz, 2H), 3.79 (dd, J = 6.4, 2.5 Hz, 2H), 2.23 (s, 1H), 1.27 (t, J = 7.1 Hz, 3H). ^{13}C NMR (101 MHz, $CDCl_3$): δ = 170.1, 163.7, 135.3, 129.5, 128.5, 127.9, 88.0, 80.4, 71.8, 58.9, 34.1, 14.5.

Synthesis of 1s

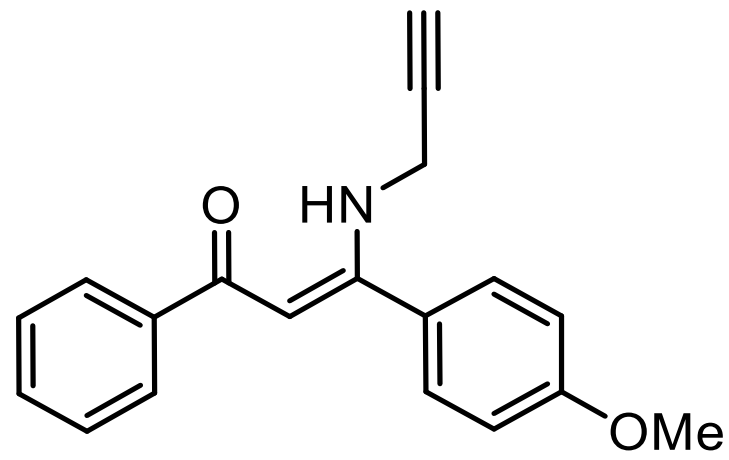

(Z)-3-(4-methoxyphenyl)-1-phenyl-3-(prop-2-yn-1-ylamino)prop-2-en-1-one[9] (**1s**). General procedure A was followed and purification by flash column chromatography afforded **1s** as a yellow oil (PE: EA = 7:1). Yield: 2.4 g, 82%. ^{1}H NMR (400 MHz, $CDCl_3$): δ = 11.35 (s, 1H), 7.90 (dd, J = 8.0, 1.5 Hz, 2H), 7.49–7.36 (m, 5H), 6.98 (d, J = 8.7 Hz, 2H), 5.83 (s, 1H), 3.98 (dd, J = 6.3, 2.5 Hz, 2H), 3.86 (s, 3H), 2.31 (s, 1H). ^{13}C NMR (101 MHz, $CDCl_3$): δ = 188.9, 166.0, 160.8, 140.0, 130.9, 129.4, 128.2, 127.1, 114.0, 94.6, 79.9, 77.2, 72.4, 55.3, 34.2.

Synthesis of 1t

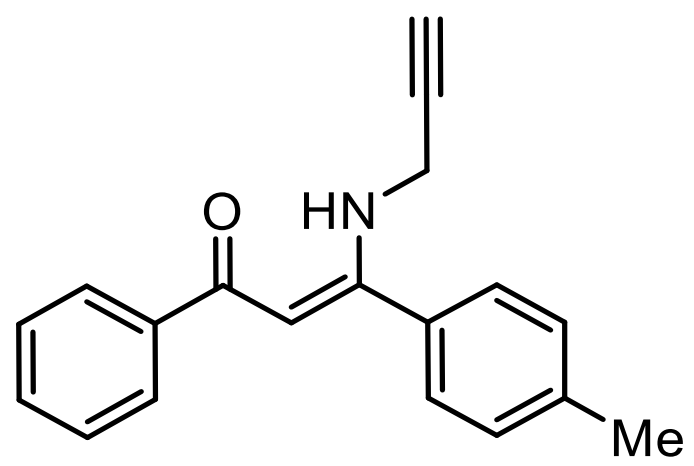

(Z)-1-phenyl-3-(prop-2-yn-1-ylamino)-3-(p-tolyl)prop-2-en-1-one[10] (**1t**). General procedure A was followed and purification by flash column chromatography afforded **1t** as a yellow oil (PE: EA = 30:1 to 7:1). Yield: 2.6 g, 95%. ^{1}H NMR (400 MHz, $CDCl_3$): δ = 11.33 (s, 1H), 7.90 (d, J = 6.6 Hz, 2H), 7.45–7.37 (m, 5H), 7.28 (d, J = 7.9 Hz, 2H), 5.84 (s, 1H), 3.96 (dd, J = 6.3, 2.5 Hz, 2H), 2.42 (s, 3H), 2.31 (t, J = 4 Hz, 1H). ^{13}C NMR (101 MHz, $CDCl_3$): δ = 189.0, 166.1, 140.1, 140.0, 131.9, 130.9, 129.3, 128.2, 127.8, 127.1, 94.6, 79.9, 72.4, 34.2, 21.4.

Synthesis of 1u

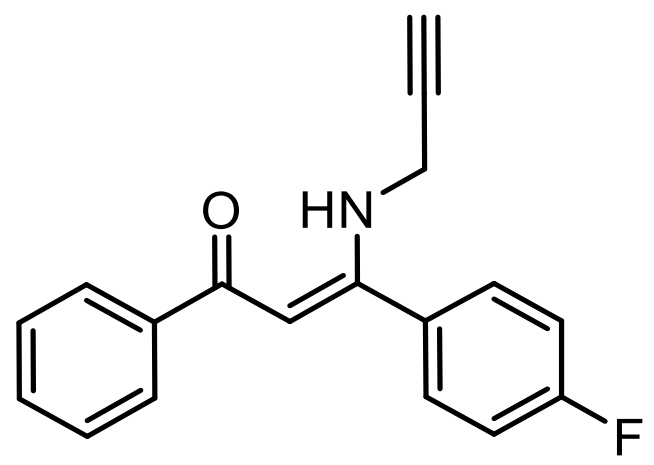

(Z)-3-(4-fluorophenyl)-1-phenyl-3-(prop-2-yn-1-ylamino)prop-2-en-1-one[4] (**1u**). General procedure A was followed and purification by flash column chromatography afforded **1u** as a yellow solid (PE: EA = 15:1). Yield: 2.4 g, 85%. Mp: 63–64 ºC. ^{1}H NMR (400 MHz, $CDCl_3$): δ = 11.29 (s, 1H), 8.04–7.86 (m, 2H), 7.53–7.36 (m, 5H), 7.15 (t, J = 8.6 Hz, 2H), 5.82 (s, 1H), 3.91 (dd, J = 6.4, 2.5 Hz, 2H), 2.31 (t, J = 2.5 Hz, 1H). ^{13}C NMR (101 MHz, $CDCl_3$): δ = 189.1, 164.6, 162.1, 139.7, 131.0, 130.9 (d, $J_{C\text{-}F}$ = 4 Hz), 129.9, 129.8, 127.1, 115.7 (d, $J_{C\text{-}F}$ = 21 Hz), 94.7, 79.6, 72.5, 34.1.

Synthesis of 1v

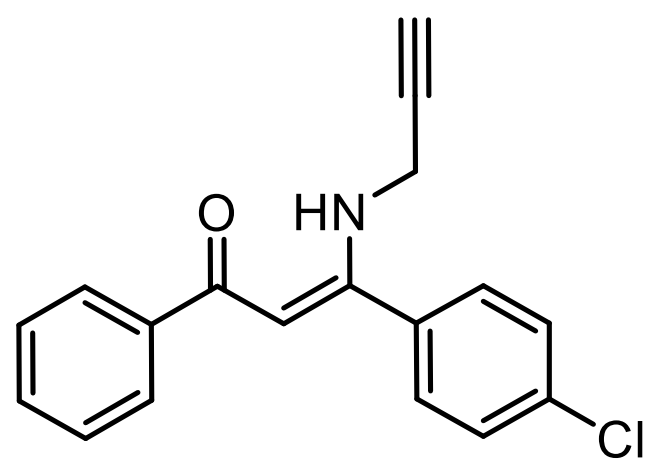

(Z)-3-(4-chlorophenyl)-1-phenyl-3-(prop-2-yn-1-ylamino)prop-2-en-1-one[4] (**1v**). General procedure A was followed and purification by flash column chromatography afforded **1v** as a yellow solid (PE: EA = 15:1). Yield: 2.3 g, 79%. mp: 90–92 ºC. ^{1}H NMR (400 MHz, $CDCl_3$): δ = 11.25 (s, 1H), 7.93–7.86 (m, 2H), 7.46–7.37 (m, 7H),

5.81 (s, 1H), 3.91 (dd, *J* = 6.4, 2.5 Hz, 2H), 2.31 (t, *J* = 2.5 Hz, 1H). ^{13}C NMR (101 MHz, $CDCl_3$): δ = 189.2, 164.4, 139.7, 135.9, 133.2, 131.1, 129.2, 128.9, 128.2, 127.1, 94.7, 79.6, 72.6, 34.1.

Synthesis of 1w

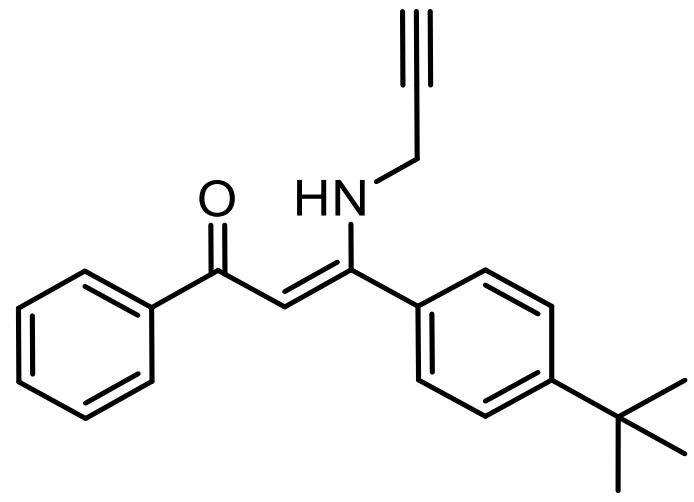

(Z)-3-(4-(tert-butyl)phenyl)-1-phenyl-3-(prop-2-yn-1-ylamino)prop-2-en-1-one[11] (**1w**). General procedure A was followed and purification by flash column chromatography afforded **1w** as a yellow solid (PE: EA = 10:1). Yield: 3.0 g, 96%. Mp: 99–101 ºC. ^{1}H NMR (400 MHz, $CDCl_3$): δ = 11.38 (s, 1H), 7.91 (dd, *J* = 8.0, 1.5 Hz, 2H), 7.52–7.37 (m, 7H), 5.86 (s, 1H), 3.98 (dd, *J* = 6.3, 2.5 Hz, 2H), 2.33 (s, 1H), 1.37 (s, 9H). ^{13}C NMR (101 MHz, $CDCl_3$): δ = 189.9, 165.9, 153.1, 139.9, 131.9, 130.8, 128.1, 127.6, 127.1, 125.5, 94.5, 79.8, 72.4, 34.7, 34.2, 31.2.

Synthesis of 1x

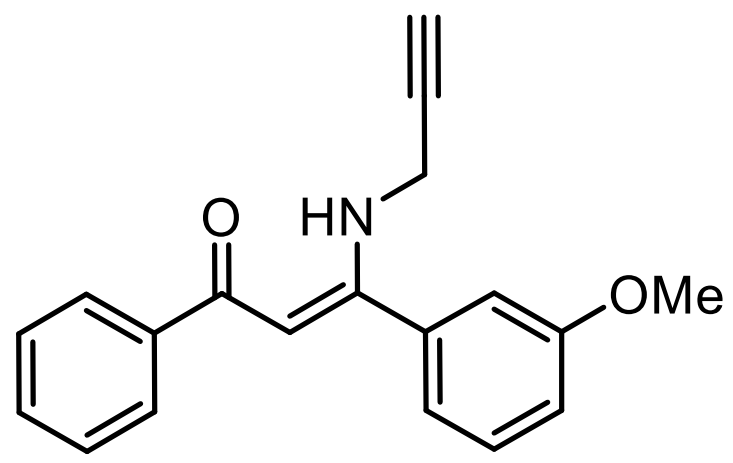

(Z)-3-(3-methoxyphenyl)-1-phenyl-3-(prop-2-yn-1-ylamino)prop-2-en-1-one (**1x**). General procedure A was followed and purification by flash column chromatography afforded **1x** as brown oil (PE: EA = 15:1). Yield: 2.7 g, 95%. ^{1}H NMR (400 MHz, $CDCl_3$): δ = 11.31 (s, 1H), 7.93–7.87 (m, 2H), 7.45–7.35 (m, 4H), 7.07–6.99 (m, 3H), 5.86 (s, 1H), 3.95 (dd, *J* = 6.4, 2.6 Hz, 2H), 3.85 (s, 3H), 2.32 (t, *J* = 2.5 Hz, 1H). ^{13}C NMR (101 MHz, $CDCl_3$): δ = 189.1, 165.6, 159.6, 139.9, 136.1, 131.0, 129.8, 128.2, 127.1, 120.0, 115.6, 113.1, 94.4, 79.9, 72.4, 55.3, 34.2. HRMS (ESI): m/z $[M + H]^+$ calcd for: $C_{19}H_{18}NO_2$: 292.1338; found: 292.1342.

Synthesis of 1y

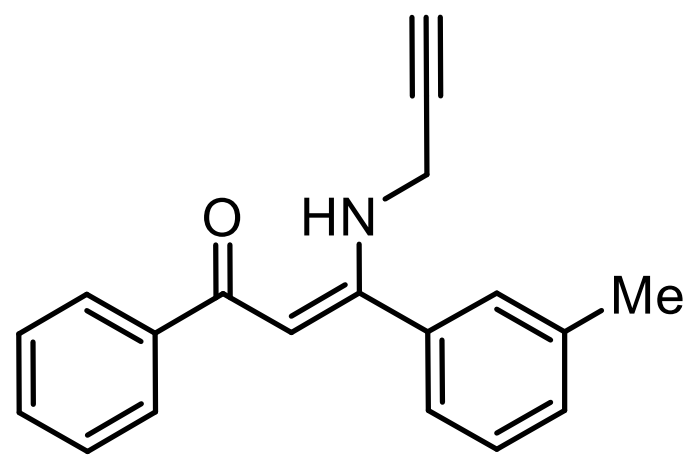

(Z)-1-phenyl-3-(prop-2-yn-1-ylamino)-3-(m-tolyl)prop-2-en-1-one (**1y**) [3]. General procedure A was followed and purification by flash column chromatography afforded **1y** as brown oil (PE: EA = 15:1). Yield: 2.6 g, 96%. ^{1}H NMR (400 MHz, $CDCl_3$): δ = ^{1}H NMR (400 MHz, $CDCl_3$): δ = 11.35 (s, 1H), 7.93–7.89 (m, 2H), 7.45–7.38 (m, 3H), 7.3 –7.33 (m, 1H), 7.31–7.27 (m, 3H), 5.85 (s, 1H), 3.94 (dd, *J* = 6.3, 2.5 Hz, 2H), 2.42 (s, 3H), 2.32 (t, *J* = 2.5 Hz, 1H). ^{13}C NMR (101 MHz, $CDCl_3$): δ = 189.0, 166.2, 139.9, 138.5, 134.8, 130.9, 130.5, 128.5, 128.4, 128.2, 127.1, 124.8, 94.5, 79.8, 72.4, 34.2, 21.4.

Synthesis of 1z

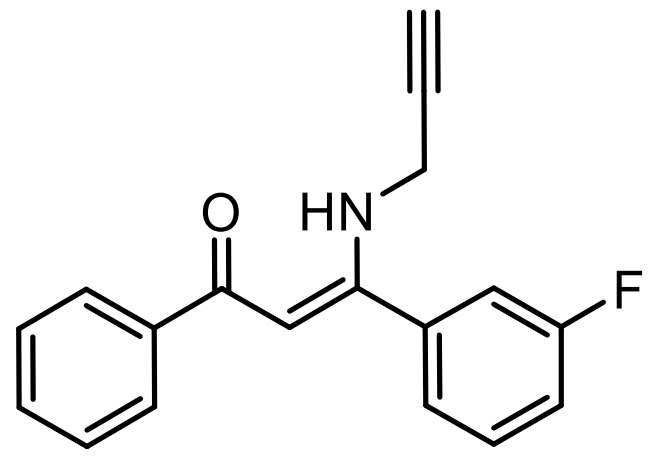

(Z)-3-(3-fluorophenyl)-1-phenyl-3-(prop-2-yn-1-ylamino)prop-2-en-1-one (**1z**)[10]. General procedure A was followed and purification by flash column chromatography afforded **1z** as a brown solid (PE: EA = 7:1). Yield: 1.16 g, 83%; Mp: 78–80 ºC. ^{1}H NMR (400 MHz, $CDCl_3$): δ = 11.24 (t, *J* = 6.2 Hz, 1H), 7.94–7.86 (m, 2H), 7.48–7.37 (m, 4H), 7.32–7.26 (m, 1H), 7.25–7.14 (m, 2H), 5.84 (s, 1H), 3.92 (dd, *J* = 6.4, 2.5 Hz, 2H), 2.32 (t, *J* = 2.5 Hz, 1H).^{13}C NMR (101 MHz, $CDCl_3$): δ = 189.3, 164.1, 162.5 (d, J_{C-F} = 247 Hz), 139.6, 136.8 (d, J_{C-F} = 8 Hz), 131.1, 130.4 (d, J_{C-F} = 8 Hz), 1282, 127.1, 123.6, 123.6, 116.9, 116.7, 115.1(d, J_{C-F} = 22 Hz), 94.6, 79.5, 72.6, 34.1.

Synthesis of 1aa

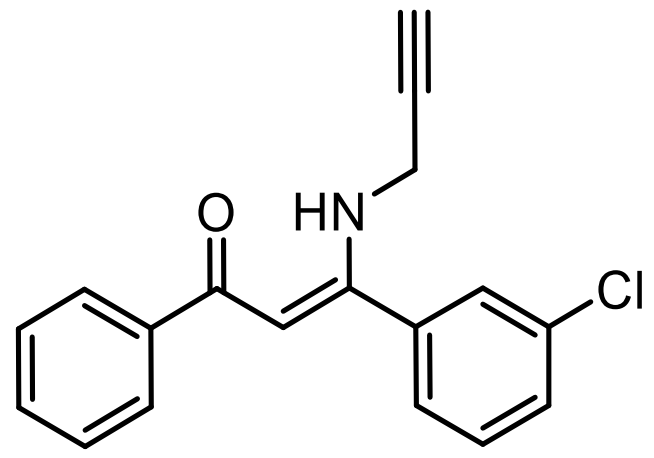

(Z)-3-(3-chlorophenyl)-1-phenyl-3-(prop-2-yn-1-ylamino)prop-2-en-1-one (**1aa**). General procedure A was followed and purification by flash column chromatography afforded **1aa** as a brown solid (PE: EA = 7:1). Yield: 1.2 g, 82%; Mp: 76–78 ºC. ^{1}H NMR (400 MHz, $CDCl_3$): δ = 11.23 (s, 1H), 7.94–7.85 (m, 2H), 7.51–7.34 (m, 7H), 5.81 (s, 1H), 3.93–3.86 (m, 2H), 2.33 (t, J = 2.5 Hz, 1H). ^{13}C NMR (101 MHz, $CDCl_3$): δ = 189.2, 163.8, 139.5, 136.4, 134.5, 131.1, 129.9, 129.8, 128.1, 127.8, 127.0, 125.9, 94.6, 79.5, 72.6, 34.0. HRMS (ESI): m/z $[M + H]^+$ calcd for: $C_{18}H_{15}NOCl$: 296.0842; found: 296.0847.

Synthesis of 1ab

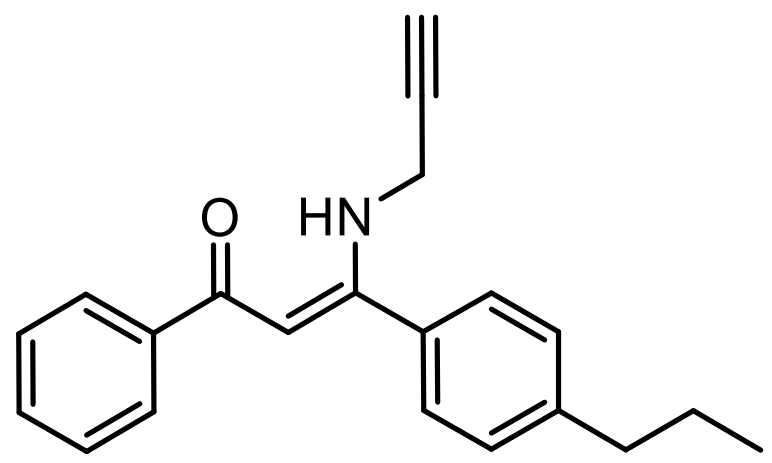

(Z)-1-phenyl-3-(prop-2-yn-1-ylamino)-3-(4-propylphenyl)prop-2-en-1-one (**1ab**). General procedure A was followed and purification by flash column chromatography afforded **1ab** as a brown oil (PE: EA = 20:1). Yield: 2.7 g, 92%. ^{1}H NMR (400 MHz, $CDCl_3$): δ = 11.35 (s, 1H), 7.93–7.87 (m, 2H), 7.45–7.39 (m, 5H), 7.29–7.26 (m, 2H), 5.85 (s, 1H), 3.97 (dd, J = 6.3, 2.5 Hz, 2H), 2.65 (t, J = 8 Hz, 2H), 2.31 (s, 1H), 1.73–1.64 (m, 2H), 0.98 (t, J = 7.3 Hz, 3H). ^{13}C NMR (101 MHz, $CDCl_3$): δ = 188.9, 166.1, 144.8, 140.0, 132.2, 130.9, 128.7, 128.2, 127.8, 127.1, 94.5, 79.8, 72.4, 37.8, 34.2, 24.3, 13.8. HRMS (ESI): m/z $[M + H]^+$ calcd for: $C_{21}H_{22}NO$: 304.1701; found: 304.1704.

Synthesis of 1ad

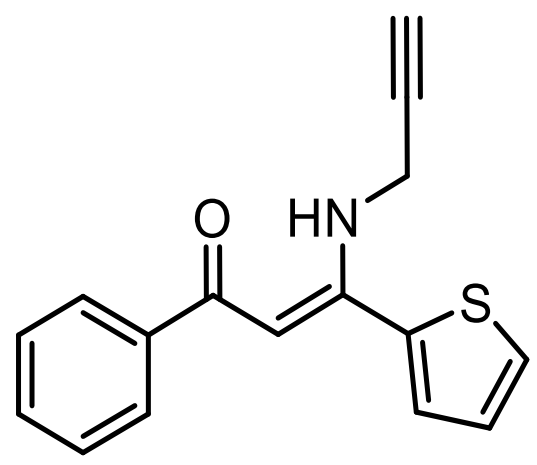

(Z)-1-phenyl-3-(prop-2-yn-1-ylamino)-3-(thiophen-2-yl)prop-2-en-1-one[7] (**1ad**). General procedure A was followed and purification by flash column chromatography afforded **1ad** as a yellow solid (PE: EA = 7:1). Yield: 1.2 g, 46%; mp: 80–81 ºC. ^{1}H NMR (400 MHz, $CDCl_3$): δ =11.40 (s, 1H), 8.02–7.80 (m, 2H), 7.54–7.35 (m, 5H), 7.15 (dd, J = 4.8, 3.9 Hz, 1H), 6.05 (s, 1H), 4.16 (dd, J = 6.3, 2.5 Hz, 2H), 2.36 (t, J = 2.5 Hz, 1H).^{13}C NMR (101 MHz, $CDCl_3$) : δ = 189.1, 158.1, 139.8, 135.5, 131.1, 129.1, 128.3, 128.1, 127.7, 127.2, 95.2, 79.9, 72.7, 34.4.

Synthesis of 1ae

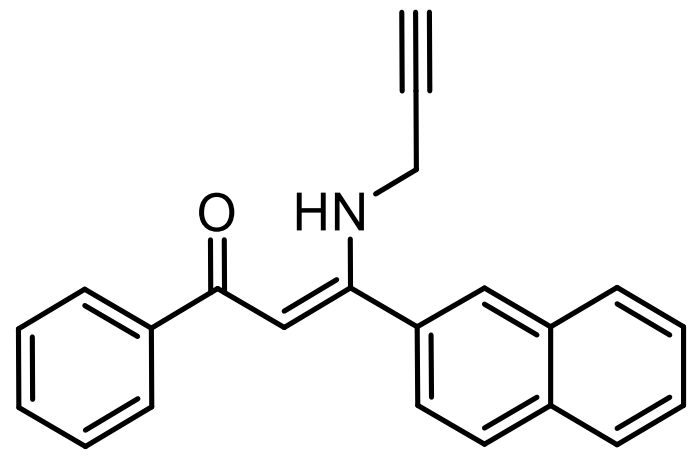

(Z)-3-(naphthalen-2-yl)-1-phenyl-3-(prop-2-yn-1-ylamino)prop-2-en-1-one (**1ae**). General procedure A was followed and purification by flash column chromatography afforded **1ae** as a yellow solid (PE: EA = 20:1). Yield: 2.3 g, 75%. Mp: 80–81 ºC. ^{1}H NMR (400 MHz, $CDCl_3$): δ = 11.38 (s, 1H), 8.01 (s, 1H), 7.96–7.90 (m, 5H), 7.59–7.56 (m, 3H), 7.46–7.40 (m, 3H), 5.96 (s, 1H), 3.99 (dd, J = 6.3, 2.5 Hz, 2H), 2.32 (t, J = 4 Hz, 1H). ^{13}C NMR (101 MHz, $CDCl_3$): δ = 189.1, 165.8, 139.9, 133.7, 132.9, 132.3, 131.0, 128.5, 128.4, 128.3, 127.8, 127.6, 127.3, 127.2, 126.9, 125.0, 95.0, 79.8, 72.5, 34.4. HRMS (ESI): m/z $[M + H]^+$ calcd for: $C_{21}H_{22}NO$: 304.1701; found: 304.1704.

Synthesis of 1af

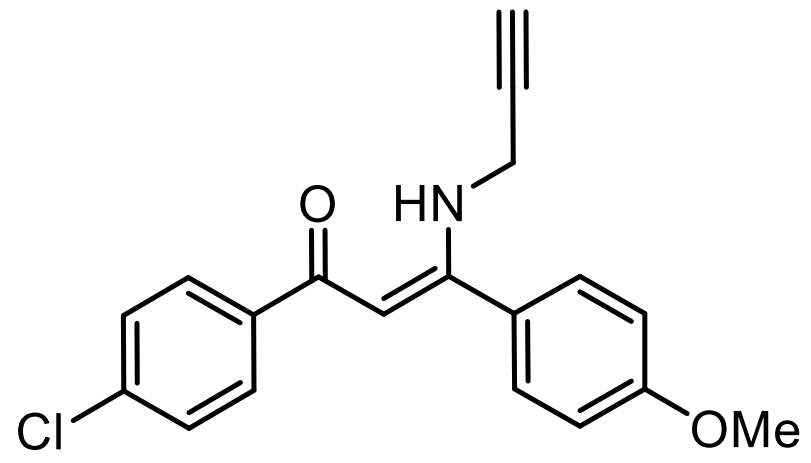

(Z)-1-(4-chlorophenyl)-3-(4-methoxyphenyl)-3-(prop-2-yn-1-ylamino)prop-2-en-1-one (**1af**) [5]. General procedure A was followed and purification by flash column chromatography afforded **1af** as a brown solid (PE: EA = 20:1). Yield: 2.9 g, 90%. mp 105–106 ºC. ^{1}H NMR (400 MHz, $CDCl_3$): δ = 11.35 (s, 1H), 7.83 (d, J = 8.6 Hz, 2H), 7.45–7.42 (m, 2H), 7.38–7.34 (m, 2H), 7.01–6.96 (m, 2H), 5.77 (s, 1H), 3.98 (dd, J = 6.3, 2.5 Hz, 2H), 3.86 (s, 3H), 2.32 (t, J = 2.5 Hz, 1H). ^{13}C NMR (101 MHz, $CDCl_3$): δ = 187.3, 166.3, 160.9, 138.4, 137.0, 134.0, 129.4, 128.5, 128.4, 126.9, 114.1, 94.1, 79.7, 72.5, 55.4, 34.3.

Synthesis of 1ag

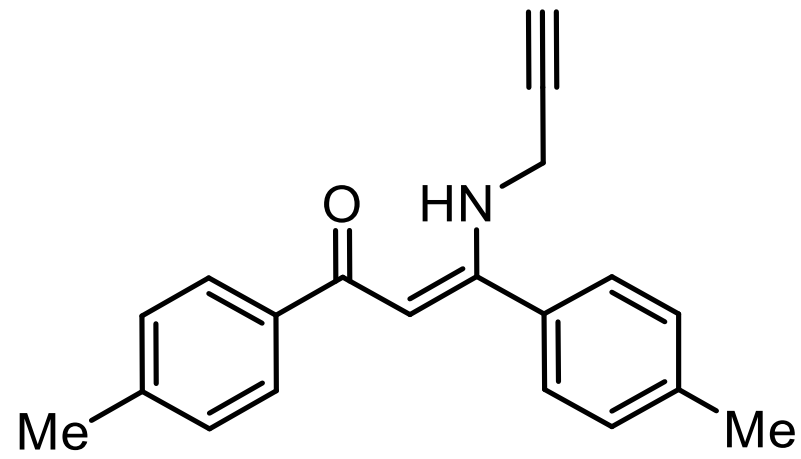

(Z)-3-(prop-2-yn-1-ylamino)-1,3-di-p-tolylprop-2-en-1-one[4] (**1ag**). General procedure A was followed and purification by flash column chromatography afforded **1ag** as a brown oil (PE: EA = 8:1). Yield: 85%. ^{1}H NMR (400 MHz, $CDCl_3$): δ = 11.31 (s, 1H), 7.81 (d, J = 8.2 Hz, 2H), 7.39 (d, J = 8.1 Hz, 2H), 7.27 (d, J = 7.7 Hz, 3H), 7.20 (d, J = 7.9 Hz, 2H), 5.83 (s, 1H), 3.95 (dd, J = 6.3, 2.6 Hz, 2H), 2.42 (s, 3H), 2.38 (s, 3H). ^{13}C NMR (101 MHz, $CDCl_3$): δ = 188.8, 165.8, 141.2, 139.9, 137.2, 132.0, 129.2, 128.8, 127.7, 127.1, 94.4, 79.9, 72.3, 34.1, 21.4, 21.3.

Synthesis of 1ah

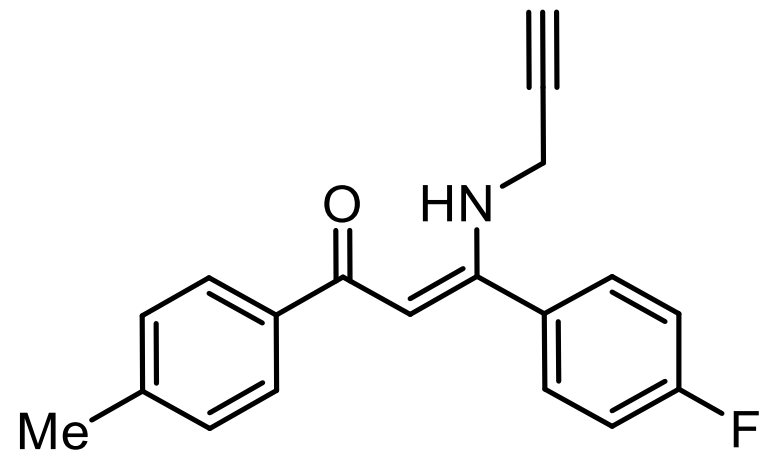

(Z)-3-(4-fluorophenyl)-3-(prop-2-yn-1-ylamino)-1-(p-tolyl)prop-2-en-1-one (**1ah**). General procedure A was followed and purification by flash column chromatography afforded **1ah** as a brown oil (PE: EA = 20:1). Yield: 2.5 g, 86%. ^{1}H NMR (400 MHz, $CDCl_3$): δ = 11.25 (s, 1H), 7.82–7.78 (m, 2H), 7.51–7.46 (m, 2H), 7.23–7.13 (m, 4H), 5.80 (s, 1H), 3.90 (dd, J = 6.4, 2.5 Hz, 2H), 2.37 (s, 3H), 2.30 (t, J = 2.5 Hz, 1H). ^{13}C NMR (101 MHz, $CDCl_3$): δ = 189.0, 164.4, 163.4 (d, $J_{C\text{-}F}$ = 249 Hz), 141.5, 137.0, 130.9 (d, $J_{C\text{-}F}$ = 3 Hz), 129.8 (d, $J_{C\text{-}F}$ = 9 Hz), 128.9, 127.2, 115.7 (d, $J_{C\text{-}F}$ = 21 Hz), 94.7, 79.7, 72.4, 34.0, 21.4. HRMS (ESI): m/z [M + H]$^+$ calcd for: $C_{19}H_{17}NOF$: 294.1294; found: 294.1297.

Synthesis of 1ai

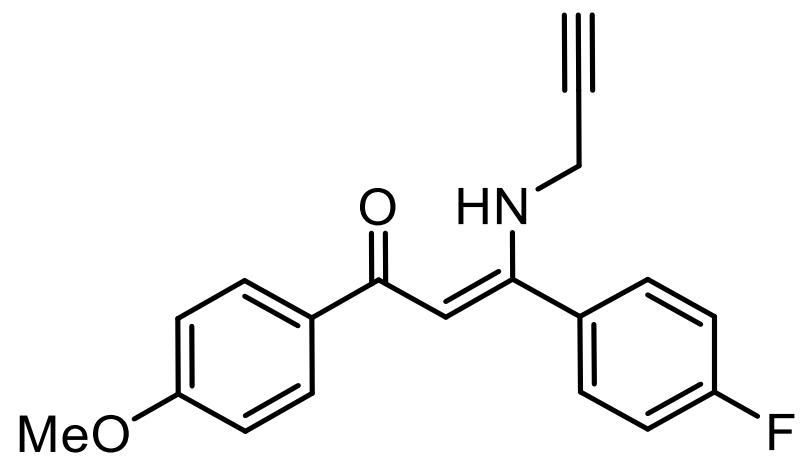

(Z)-3-(4-fluorophenyl)-1-(4-methoxyphenyl)-3-(prop-2-yn-1-ylamino)prop-2-en-1-one (**1ai**). General procedure A was followed and purification by flash column chromatography afforded **1ai** as a brown solid (PE: EA = 20:1). Yield: 2.7 g, 90%. mp 92–94 °C. ^{1}H NMR (400 MHz, $CDCl_3$): δ = 11.17 (s, 1H), 7.88 (d, J = 8.9 Hz, 2H), 7.49 (dd, J = 8.6, 5.4 Hz, 2H), 7.15 (t, J = 8.6 Hz, 2H), 6.90 (d, J = 8.8 Hz, 2H), 5.78 (s, 1H), 3.90 (dd, J = 6.4, 2.5 Hz, 2H), 3.84 (s, 3H), 2.29 (s, 1H). ^{13}C NMR (101 MHz, $CDCl_3$): δ = 188.3, 164.2, 162.1, 132.5, 132.1, 130.0, 129.9 (d, $J_{C\text{-}F}$ = 9 Hz), 129.1, 115.8 (d, $J_{C\text{-}F}$ = 21 Hz), 113.5, 94.5, 79.8, 72.4, 55.3, 34.1. HRMS (ESI): m/z [M + H]$^+$ calcd for: $C_{19}H_{17}NO_2F$: 310.1243; found: 310.1247.

Synthesis of 1aj

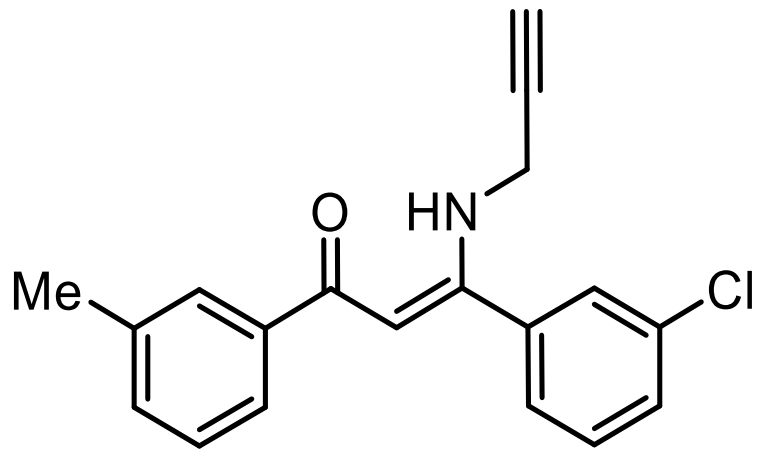

(Z)-3-(3-chlorophenyl)-3-(prop-2-yn-1-ylamino)-1-(m-tolyl) prop-2-en-1-one (**1aj**). General procedure A was followed and purification by flash column chromatography afforded **1aj** as a brown oil (PE: EA = 7:1). Yield: 1.4g, 90%. ^{1}H NMR (400 MHz, $CDCl_3$): δ = 11.20 (t, J = 6.4 Hz, 1H), 7.73–7.66 (m, 2H), 7.51–7.48 (m, 1H), 7.47–7.38 (m, 3H), 7.32–7.26 (m, 2H), 5.81 (s, 1H), 3.91 (dd, J = 6.4, 2.6 Hz, 2H), 2.39 (s, 3H), 2.32 (t, J = 4 Hz, 1H).^{13}C NMR (101 MHz, $CDCl_3$): δ =189.7, 163.9, 139.6, 137.9, 136.6, 134.7, 131.9, 123.0, 129.9, 128.1, 128.0, 127.8, 126.0, 124.3, 94.9, 79.5, 72.6, 34.2, 21.4. HRMS (ESI): m/z [M + H]$^+$ calcd for: $C_{19}H_{17}NOCl$: 310.0999; found: 310.1001.

Synthesis of 1ak

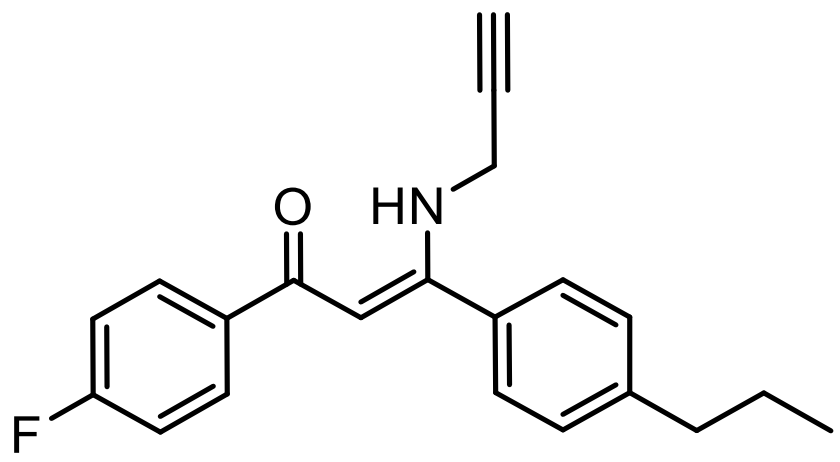

(Z)-1-(4-fluorophenyl)-3-(prop-2-yn-1-ylamino)-3-(4-propylphenyl)prop-2-en-1-one (**1ak**). General procedure A was followed and purification by flash column chromatography afforded **1ak** as a brown oil (PE: EA = 40:1). Yield: 2.6 g, 82%. ^{1}H NMR (400 MHz, $CDCl_3$): δ = 11.31 (s, 1H), 7.96–7.84 (m, 2H), 7.43–7.36 (m, 2H), 7.27 (d, J = 8.3 Hz, 2H), 7.09–7.01 (t, J = 8.7 Hz, 2H), 5.78 (s, 1H), 3.96 (dd, J = 6.3, 2.5 Hz, 2H), 2.64 (t, J = 8 Hz, 2H), 2.31 (t, J = 2.5 Hz, 1H), 1.73–1.64 (m, 2H), 0.97 (t, J = 7.3 Hz, 3H). ^{13}C NMR (101 MHz, $CDCl_3$): δ = 187.4, 166.3, 164.5 (d, $J_{C\text{-}F}$ = 250 Hz), 165.7, 163.2, 144.9, 136.2, 132.1, 129.4 (d, $J_{C\text{-}F}$ = 9 Hz), 128.7, 127.7, 115.1 (d, $J_{C\text{-}F}$ = 21 Hz), 94.1, 79.7, 72.4, 37.8, 34.2, 24.3, 13.8. HRMS (ESI): m/z [M + H]$^+$ calcd for: $C_{21}H_{21}NOF$: 322.1607; found: 322.1609.

Synthesis of 1al

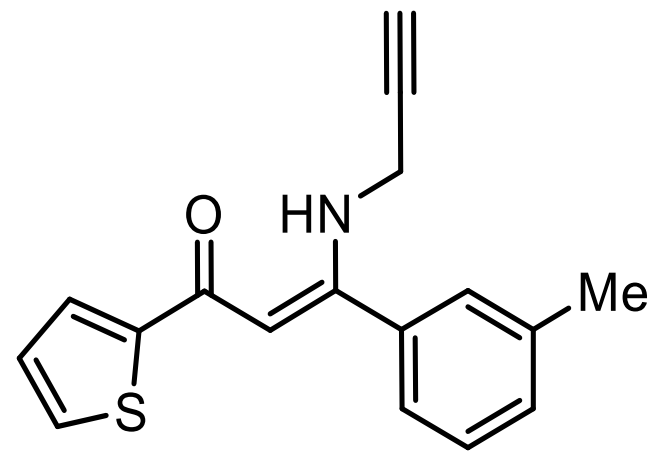

(Z)-3-(prop-2-yn-1-ylamino)-1-(thiophen-2-yl)-3-(m-tolyl)prop-2-en-1-one (**1al**). General procedure A was followed and purification by flash column chromatography afforded **1al** as a brown oil (PE: EA = 30:1). Yield: 2.7 g, 95%. ^{1}H NMR (400 MHz, $CDCl_3$): δ = 10.96 (s, 1H), 7.56 (dd, J = 3.7, 1.2 Hz, 1H), 7.47 (dd, J = 4.9, 1.2 Hz, 1H), 7.38–7.33 (m, 1H), 7.31–7.26 (m, 3H), 7.06 (dd, J = 5.0, 3.7 Hz, 1H), 5.70 (s, 1H), 3.91 (dd, J = 6.3, 2.5 Hz, 2H), 2.42 (s, 3H), 2.29 (t, J = 2.5 Hz, 1H).^{13}C NMR (101 MHz, $CDCl_3$) : δ = 181.9, 165.8, 146.8, 138.6, 134.6, 130.6, 130.5, 128.6, 128.3, 128.1, 127.7, 124.8, 94.2, 79.7, 72.4, 34.2, 21.4. HRMS (ESI): m/z [M + H]$^+$ calcd for: $C_{17}H_{16}NOS$: 282.0953; found: 282.0957.

Synthesis of 1am

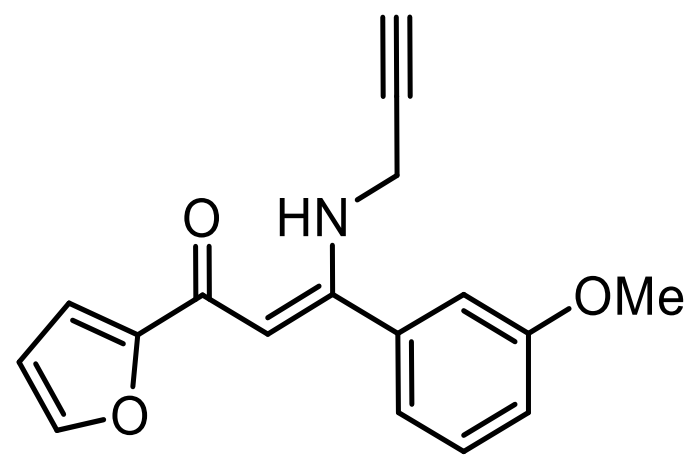

(Z)-1-(furan-2-yl)-3-(3-methoxyphenyl)-3-(prop-2-yn-1-ylamino)prop-2-en-1-one (**1am**). General procedure A was followed and purification by flash column chromatography afforded **1am** as brown oil (PE: EA = 10:1). Yield: 2.6 g, 92%. ^{1}H NMR (400 MHz, $CDCl_3$): δ = 10.96 (s, 1H), 7.46 (dd, J = 1.8, 0.8 Hz, 1H), 7.35 (t, J = 7.8 Hz, 1H), 7.05–6.97 (m, 4H), 6.46 (dd, J = 3.5, 1.8 Hz, 1H), 5.77 (s, 1H), 3.92 (dd, J = 6.4, 2.5 Hz, 2H), 3.84 (s, 3H), 2.30 (t, J = 4 Hz, 1H).^{13}C NMR (101 MHz, $CDCl_3$) : δ = 178.3, 165.75, 159.6, 154.1, 144.4, 135.8, 129.8, 120.0, 115.7, 113.2, 113.1, 111.9, 93.9, 79.8, 72.4, 55.3, 34.3. HRMS (ESI): m/z [M + H]$^+$ calcd for: $C_{17}H_{14}NO_3$: 280.0974; found: 280.0975.

Synthesis of 1an

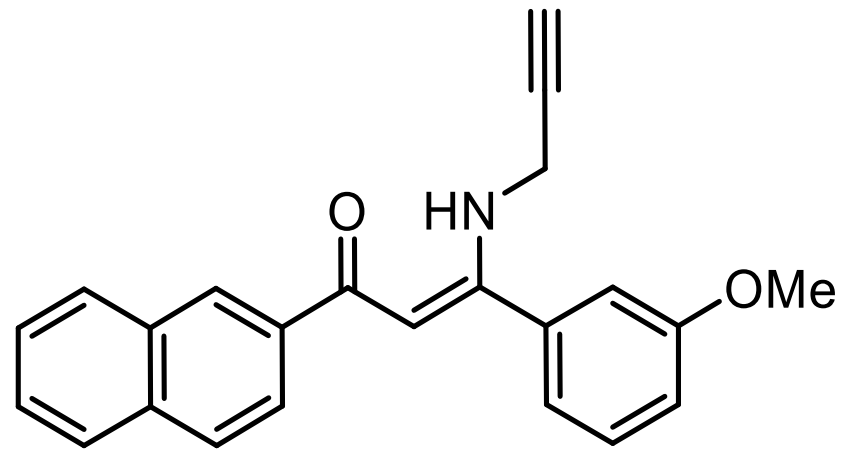

(Z)-3-(3-methoxyphenyl)-1-(naphthalen-2-yl)-3-(prop-2-yn-1-ylamino)prop-2-en-1-one (**1an**). General procedure A was followed and purification by flash column chromatography afforded **1an** as brown oil (PE: EA = 15:1). Yield: 2.6 g, 75%. ^{1}H NMR (400 MHz, $CDCl_3$): δ = 11.42 (s, 1H), 8.42 (d, J = 1.7 Hz, 1H), 8.05–8.01 (m, 1H), 7.93-7.90 (m, 1H), 7.88-7.83 (m, 2H), 7.55-7.45 (m, 3H), 7.40 (t, J = 7.9 Hz, 1H), 7.12–7.08 (m, 2H), 7.04-7.0 (m, 1H), 6.04 (s, 1H), 3.99 (dd, J = 6.4, 2.5 Hz, 2H), 3.86 (s, 3H), 2.36 (t, J = 2.5 Hz, 1H). ^{13}C NMR (101 MHz, $CDCl_3$): δ = 188.8, 165.7, 159.5, 137.1, 136.0, 134.6, 132.7, 129.8, 129.1, 127.9, 127.6, 127.5, 127.2, 126.2, 124.0, 120.0, 115.5, 113.2, 94.5, 79.8, 72.4, 55.3, 34.2. HRMS (ESI): m/z $[M + H]^+$ calcd for: $C_{23}H_{20}NO_2$: 342.1494; found: 342.1497.

Synthesis of 1i

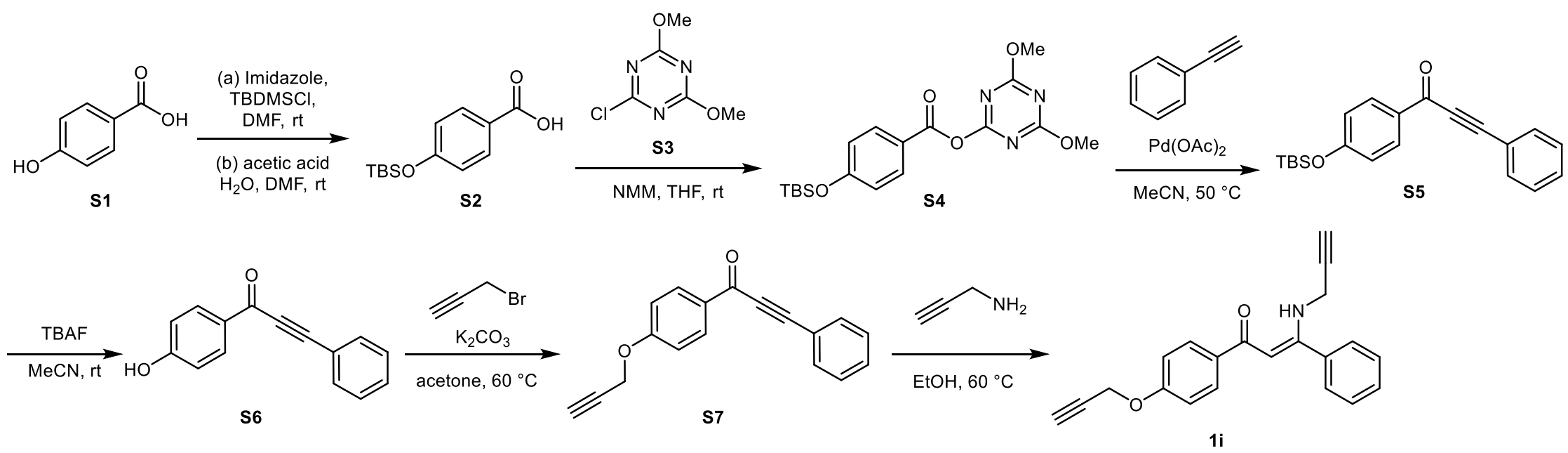

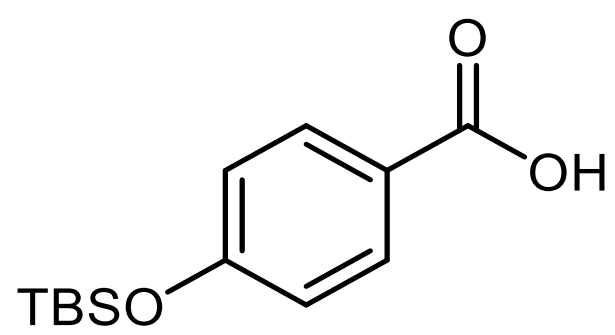

S2

4-((tert-butyldimethylsilyl)oxy)benzoic acid[13] (**S2**)

Para-hydroxybenzoic acid **S1** (690 mg, 5.0 mmol) was dissolved in dry DMF (50.0 mL) under a nitrogen atmosphere. Subsequently, TBDMSCl (1.5 g, 10.0 mmol) and imidazole (680 mg, 10.0 mmol) were added sequentially. The reaction mixture was

stirred at room temperature for 4 hours, after which it was poured into water (50 mL) and extracted with ethyl acetate (50 mL × 3). The combined organic phases were dried, filtered, and concentrated under reduced pressure to obtain the residue. The residue was then dissolved in THF (15 mL) and water (3 mL), followed by the addition of acetic acid (12 mL). The reaction mixture was stirred at room temperature for 20 hours, after which THF was removed under reduced pressure. Water (30 mL) was added to the reaction mixture, and the reaction mixture was extracted with ethyl acetate (30 mL × 3). The combined organic phases were dried, filtered, and concentrated under reduced pressure. The residue was purified by silica gel column chromatography (PE: EA = 20:1) to yield compound **S2** as a colorless solid. mp: 98–100 °C. ^{1}H NMR (400 MHz, $CDCl_3$): δ = 8.03 (d, J = 8.7 Hz, 2H), 6.89 (d, J = 8.7 Hz, 2H), 1.00 (s, 9H), 0.25 (s, 6H). ^{13}C NMR (101 MHz, $CDCl_3$): δ = 172.0, 160.9, 132.3, 122.3, 119.9, 25.6, 18.2, -4.39.

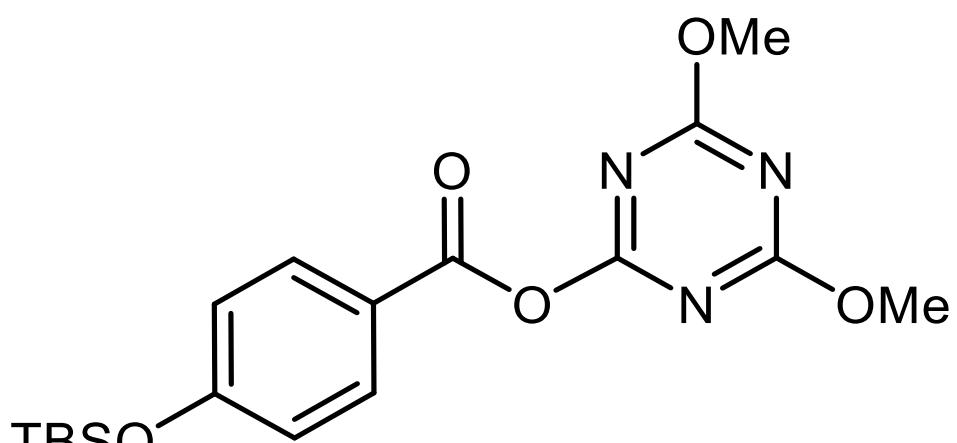

S4

4,6-dimethoxy-1,3,5-triazin-2-yl 4-((tert-butyldimethylsilyl)oxy)benzoate (**S4**)

To a solution of 2-chloro-4,6-dimethoxy-1,3,5-triazine (CDMT, 2 mmol, 350 mg) and 4-methylmorpholine (NMM, 2 mmol, 202 mg) in THF (4 mL), carboxylic acid **S2** (2 mmol, 504 mg) in THF (2 mL) was added dropwise. The reaction mixture was stirred at room temperature for 1 hour. The mixture was then filtered and concentrated under reduced pressure. The resulting residue was dissolved in ethyl acetate (30 mL) and washed sequentially with 10% citric acid solution (20 mL × 3), water (20 mL × 3), and 0.5 M $NaHCO_3$ (20 mL × 3). The combined organic layer was dried over Na_2SO_4 and concentrated under reduced pressure to obtain **S4** as a colorless oil, which was used directly in the next step without further purification.

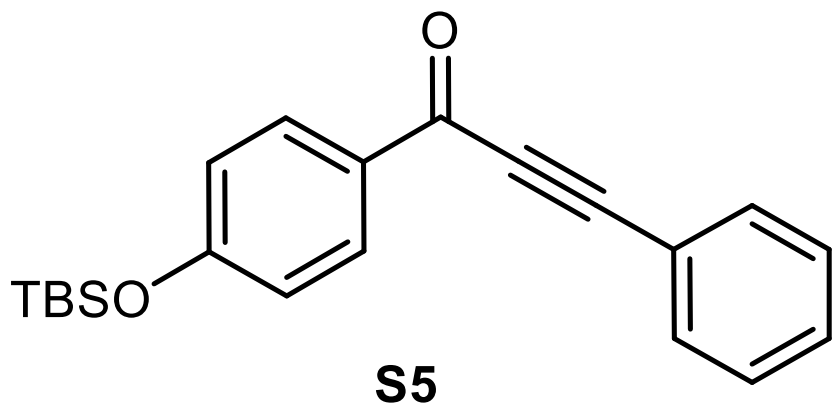

S5

1-(4-((tert-butyldimethylsilyl)oxy)phenyl)-3-phenylprop-2-yn-1-one (**S5**)

To a solution of $Pd(OAc)_2$ (0.06 mmol, 13 mg) in 15 mL CH_3CN, compound **S4** (6 mmol, 2.34 g) was added under a nitrogen atmosphere. Phenylacetylene (7.2 mmol, 734 mg) was added dropwise at room temperature. The reaction mixture was stirred at 50 °C for 10 hours. After the reaction cooled to room temperature, water (30 mL) was added, and the mixture was extracted with ethyl acetate (30 mL × 3). The combined organic phases were filtered, dried, and concentrated to yield the crude product **S5**, which was directly used in the next step.

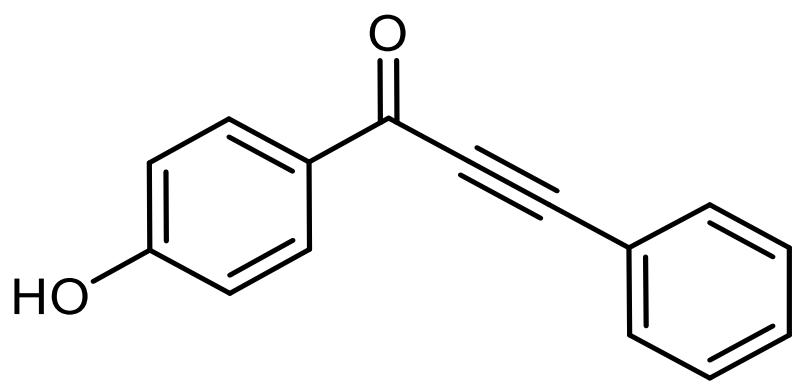

S6

1-(4-hydroxyphenyl)-3-phenylprop-2-yn-1-one (**S6**)

A solution of compound **S5** (4 mmol, 1.34 g) in methanol (20 mL) was treated with 4 mL of TBAF (1 M in THF) solution. The reaction was carried out at room temperature and monitored by TLC until complete. The reaction mixture was then concentrated under reduced pressure. The residue was dissolved in ethyl acetate (30 mL), and the organic layer was washed with water (30 mL). The aqueous phase was extracted with ethyl acetate (30 mL × 2). The combined organic layers were dried over Na_2SO_4, filtered, and concentrated to yield the crude product **S6**, which was used directly in the next step.

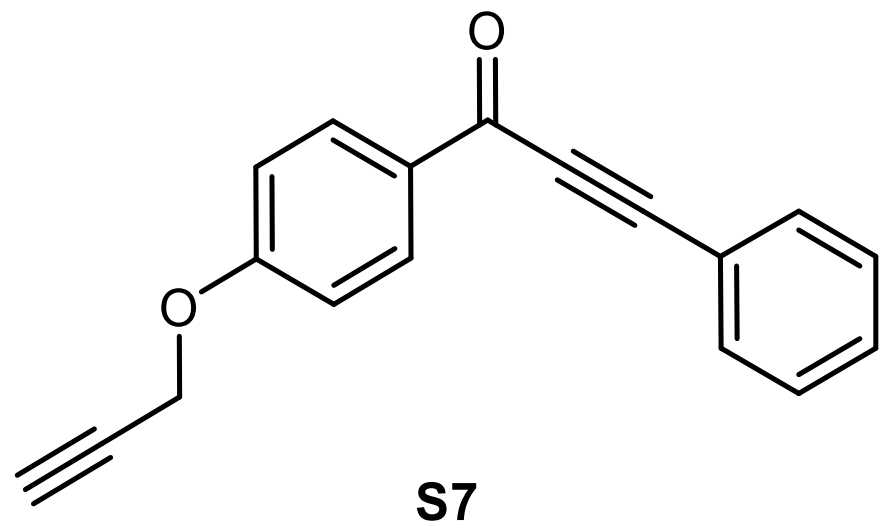

S7

3-phenyl-1-(4-(prop-2-yn-1-yloxy)phenyl)prop-2-yn-1-one (**S7**)

Compound **S6** (0.88 g, 4 mmol) and K_2CO_3 (1.66 g, 12 mmol) were placed in a 50 mL flask, followed by 20 mL of acetone and 3-bromopropyne (714 mg, 6 mmol). The reaction was stirred at 60 °C for 6 hours, and the reaction was monitored by TLC until the starting material was completely consumed. The reaction mixture was concentrated under reduced pressure to remove the solvent. The residue was re-dissolved in ethyl acetate (30 mL) and washed with water (30 mL). The aqueous phase was extracted three times with ethyl acetate (30 mL × 3). The combined organic layers were dried, filtered, and concentrated under reduced pressure. The residue was purified by silica gel column chromatography (PE: EA = 8:1) to obtain compound **S7** as a yellow solid (Yield: 884 mg, 85%). mp: 80–84 °C. ^{1}H NMR (400 MHz, $CDCl_3$): δ = 8.21 (d, J = 8.9 Hz, 2H), 7.69–7.65 (m, 2H), 7.50–7.45 (m, 1H), 7.44–7.39 (m, 2H), 7.11–7.03 (m, 2H), 4.79 (d, J = 2.4 Hz, 2H), 2.57 (s, 1H). ^{13}C NMR (101 MHz, $CDCl_3$): δ = 176.6, 162.2, 133.0, 131.9, 131.0, 130.6, 128.6, 120.3, 114.7, 92.5, 86.8, 77.6, 76.3, 55.9. HRMS (ESI): m/z $[M + H]^+$ calcd for: $C_{18}H_{13}O_2$: 261.0916; found: 261.0918.

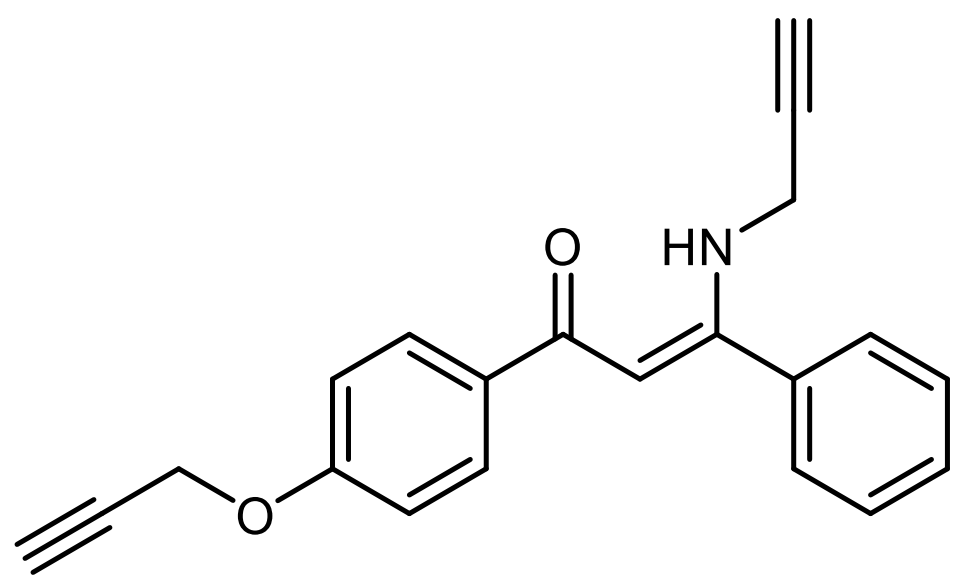

1i

(Z)-3-phenyl-3-(prop-2-yn-1-ylamino)-1-(4-(prop-2-yn-1-yloxy)phenyl)prop-2-en-1-one (**1i**)

Prop-2-yn-1-amine (6 mmol, 330 mg) and compound **S7** (5 mmol, 1300 mg) were stirred in anhydrous ethanol (5 mL) at 60 °C for 24 h. Upon completion of the reaction, the solvent was removed under reduced pressure, and the residue was purified by silica gel column chromatography (petroleum ether/ethyl acetate, PE: EA = 7:1) to afford the N-propargyl enaminone **1i** as a brown solid. (Yield: 1.34 g, 85%); mp: 63–65 °C. ^{1}H NMR (400 MHz, $CDCl_3$): δ = 11.22 (t, J = 6.3 Hz, 1H), 7.93–7.85 (m, 2H), 7.51–7.44 (m, 5H), 7.00–6.95 (m, 2H), 5.80 (s, 1H), 4.72 (d, J = 2.4 Hz, 2H), 3.92 (dd, J = 6.3, 2.5 Hz, 2H), 2.53 (t, J = 2.4 Hz, 1H), 2.29 (t, J = 2.5 Hz, 1H).^{13}C NMR (101 MHz, $CDCl_3$): δ = 188.1, 165.5, 159.8, 135.0, 133.4, 129.7, 129.0, 128.6, 127.8, 114.3, 94.3, 79.9, 78.1, 75.8, 72.3, 55.7, 34.1. HRMS (ESI): m/z [M + H]$^+$ calcd for: $C_{21}H_{18}NO_2$: 316.1338; found: 316.1342.

Synthesis of 1ac

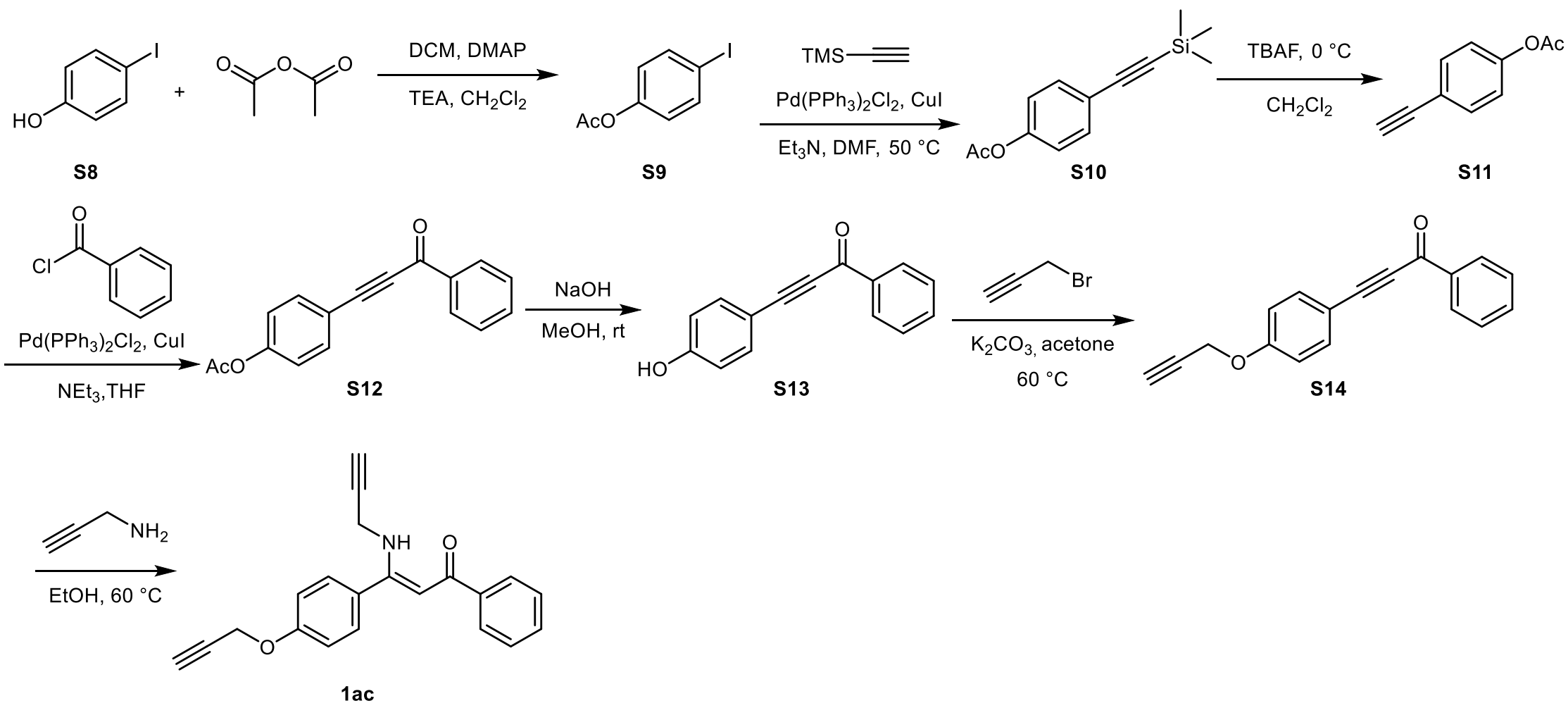

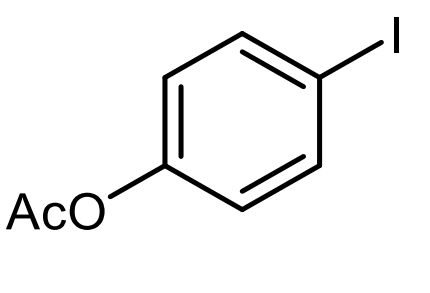

S9

4-Iodophenyl acetate[14] (**S9**)

4-Iodophenol (2.2 g, 10 mmol, 1 equiv.), DMAP (67 mg, 0.5 mmol, 0.05 equiv.), and triethylamine (0.81 g, 8 mmol, 0.8 equiv.) were dissolved in dry CH_2Cl_2 (30 mL). The resulting solution was stirred at room temperature, and a solution of acetic anhydride

(1.63 g, 16 mmol, 1.6 equiv.) in CH_2Cl_2 (10 mL) was added dropwise. After TLC indicated complete consumption of the starting material (1 h), the reaction mixture was washed sequentially with water and brine. The organic layer was dried over anhydrous Na_2SO_4 and concentrated under reduced pressure to afford a clear pink solid (2.26 g, 87%), which was used directly in the next step without further purification.

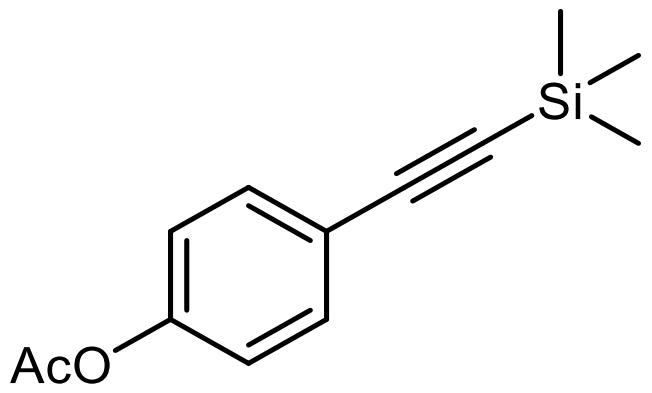

S10

4-((trimethylsilyl)ethynyl)phenyl acetate[14] (**S10**)

$Pd(PPh_3)_2Cl_2$ (210 mg, 0.3 mmol), CuI (114 mg, 0.6 mmol), and compound **S9** (2.6 g, 10 mmol) were sequentially added to a reaction flask and purged with nitrogen. Then, DMF (25 mL) was added as the solvent, followed by the slow addition of Et_3N (4 g, 40 mmol) and trimethylsilyl acetylene (1.2 g, 12 mmol). After stirring the reaction mixture at 50 °C for 1 hour, the mixture was cooled to room temperature and diluted with ethyl acetate. The organic phase was washed successively with 1M HCl aqueous solution (15 mL), deionized water (15 mL), and saturated brine (15 mL). Finally, the organic phase was dried over Na_2SO_4, filtered, and concentrated under vacuum. The crude product was purified by silica gel column chromatography to yield compound **S10** as yellow solid (PE/EA = 9:1). Yield: 2.08 g, 90%. Mp：68–69 ºC. ^{1}H NMR (400 MHz, $CDCl_3$): δ = 7.47 (d, J = 8.7 Hz, 2H), 7.03 (d, J = 8.7 Hz, 2H), 2.29 (s, 3H), 0.24 (s, 9H). ^{13}C NMR (101 MHz, $CDCl_3$): δ = 169.0, 150.6, 133.1, 121.5, 120.8, 104.2, 94.3, 21.1, -0.08.

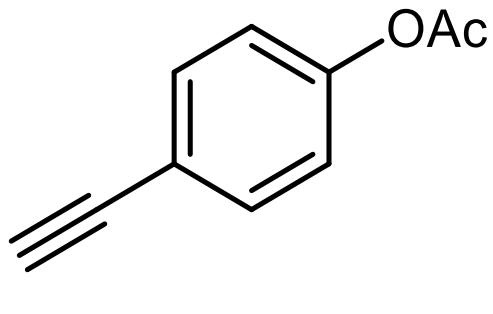

S11

4-ethynylphenyl acetate[14] (**S11**)

A solution of tetrabutylammonium fluoride (TBAF) (1 M in THF, 1.1 mL, 1.1 mmol, 1.1 equiv.) was slowly added to a solution of compound **S5** (232 mg, 1 mmol, 1 equiv.) in CH_2Cl_2 (5 mL) at 0 °C. The reaction mixture was stirred at room temperature for 1 hour, then washed with saturated NH_4Cl solution, deionized water, and brine. The organic layer was separated, dried over anhydrous $MgSO_4$, and concentrated under reduced pressure. The crude product was purified by silica gel column chromatography (PE/EA = 10:1) to give compound **S11** as yellow solid. Yield: 152 mg, 95%. Mp：55–58 ºC. ^{1}H NMR (400 MHz, $CDCl_3$): δ = 7.52–7.48 (m, 2H), 7.08–7.04 (m, 2H), 3.06 (s, 1H), 2.30 (s, 3H). ^{13}C NMR (101 MHz, $CDCl_3$): δ = 169.1, 150.8, 133.3, 121.7, 119.8, 82.8, 77.3, 21.0.

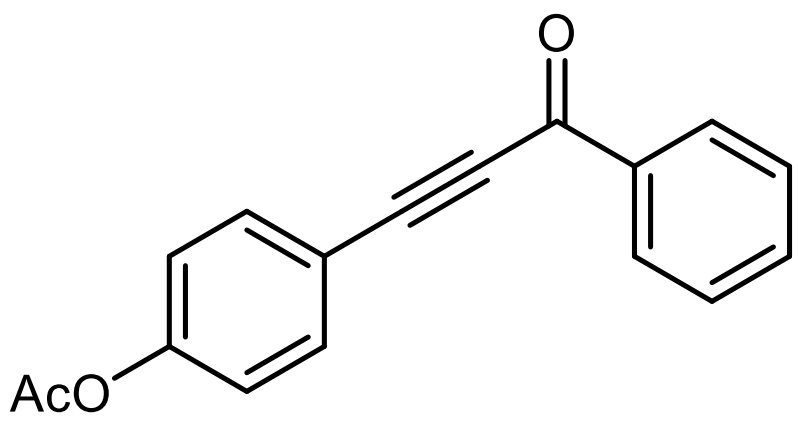

S12

4-(3-oxo-3-phenylprop-1-yn-1-yl)phenyl acetate (**S12**)

Using the standard Schlenk apparatus, alkyne (160 mg, 1 mmol, 1.0 equiv), $Pd(PPh_3)_2Cl_2$ (14 mg, 0.02 mmol, 2.0 mol%), CuI (8 mg, 0.40 mmol, 4.0 mol%) were added to a flask under nitrogen atmosphere, followed by the addition of dry THF (25 mL). Et_3N (140 μL, 1.0 mmol, 1.0 equiv) and benzoyl chloride (140 μL, 1.2 mmol, 1.2 equiv) were then added dropwise. The reaction mixture was stirred at room temperature and allowed to react under nitrogen atmosphere, monitored by TLC until the starting materials were completely consumed (1 hour). The reaction was quenched with a saturated ammonium chloride solution (20 mL). The aqueous layer was extracted with EtOAc (3 × 20 mL). The combined organic layers were washed with brine (20 mL), dried over anhydrous $MgSO_4$, filtered, and concentrated under reduced pressure. The resulting crude solid was purified by silica gel flash column chromatography, yielding compound **S12** as a yellow oil (240 mg, 91%). ^{1}H NMR (400 MHz, $CDCl_3$): δ = 8.25–8.16 (m, 2H), 7.76–7.67 (m, 2H), 7.66–7.61 (m, 1H), 7.54–7.48 (m, 2H), 7.20–7.15 (m, 2H), 2.32 (s, 3H). ^{13}C NMR (101 MHz, $CDCl_3$): δ = 177.9, 168.8, 152.4, 136.8, 134.4,

134.1, 129.5, 128.6, 122.2, 117.6, 92.2, 86.9, 21.1. HRMS (ESI): m/z $[M + H]^+$ calcd for: $C_{17}H_{13}O_3$: 265.0865; found: 265.0867.

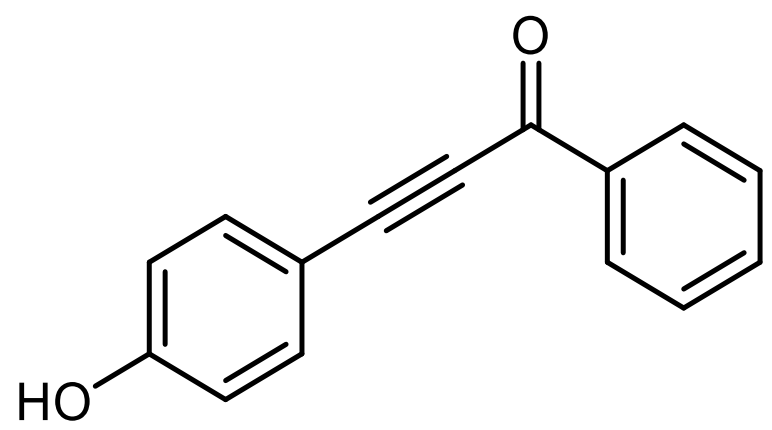

S13

3-(4-hydroxyphenyl)-1-phenylprop-2-yn-1-one (**S13**)

Sodium hydroxide (400 mg, 10 mmol) was added to a 50 mL round-bottom flask and dissolved by adding 20 mL of anhydrous ethanol. Then, **S12** (10 mmol) was added to the reaction mixture, which was stirred at room temperature for 4 hours. Afterward, the reaction mixture was concentrated under reduced pressure to remove ethanol, and 30 mL of distilled water was added. The aqueous phase was then extracted with ethyl acetate (3 × 20 mL). The combined organic layer was dried over Na_2SO_4 and filtered. Finally, the reaction mixture was concentrated under reduced pressure. The resulting crude product was used directly in the next step without further purification.

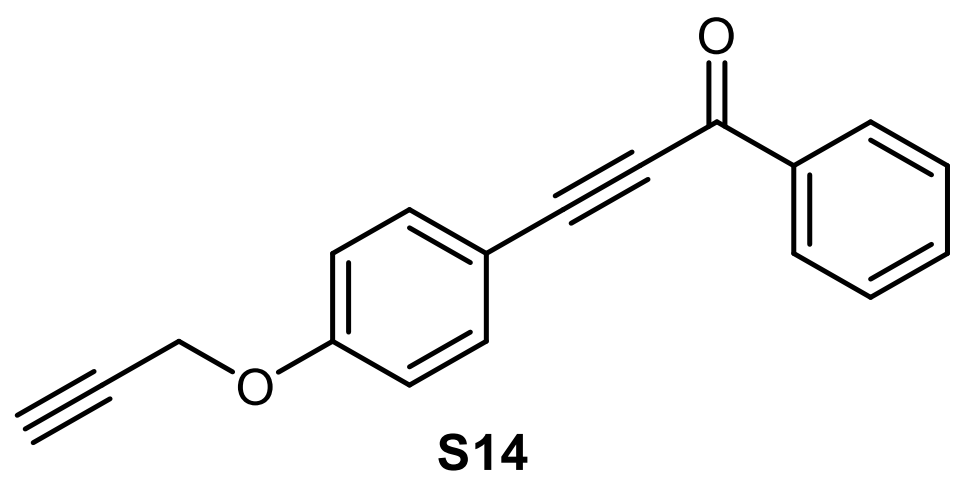

S14

1-phenyl-3-(4-(prop-2-yn-1-yloxy)phenyl)prop-2-yn-1-one (**S14**)

Compound **S13** (1.77 g, 8 mmol) and K_2CO_3 (2.21 g, 16 mmol) were placed in a 50 mL flask, followed by the addition of 30 mL of acetonitrile and 3-bromopropyne (1.43 g, 12 mmol). The mixture was stirred at room temperature for 6 hours, and the reaction was monitored by TLC until completion. Afterward, 30 mL of water was added to the reaction mixture, and the aqueous phase was extracted with dichloromethane (30 mL×3). The combined organic layers were dried over anhydrous Na_2SO_4, filtered, and concentrated under reduced pressure. The resulting crude solid was purified by silica

gel flash column chromatography, yielding **S14** as a brown solid (2.0 g, 95% yield). Mp: 68-71 ºC. ^{1}H NMR (400 MHz, $CDCl_3$): δ =8.26–8.18 (m, 2H), 7.70–7.59 (m, 3H), 7.51 (t, J = 7.6 Hz, 2H), 7.06–6.98 (m, 2H), 4.75 (d, J = 2.4 Hz, 2H), 2.57 (t, J = 2.4 Hz, 1H). ^{13}C NMR (101 MHz, $CDCl_3$): δ = 178.0, 159.5, 137.0, 135.0, 133.9, 129.5, 128.6, 115.3, 112.9, 93.8, 86.9, 76.2, 55.9, 29.7. HRMS (ESI): m/z $[M + H]^+$ calcd for: $C_{18}H_{13}O_2$: 261.0916; found: 261.0919.

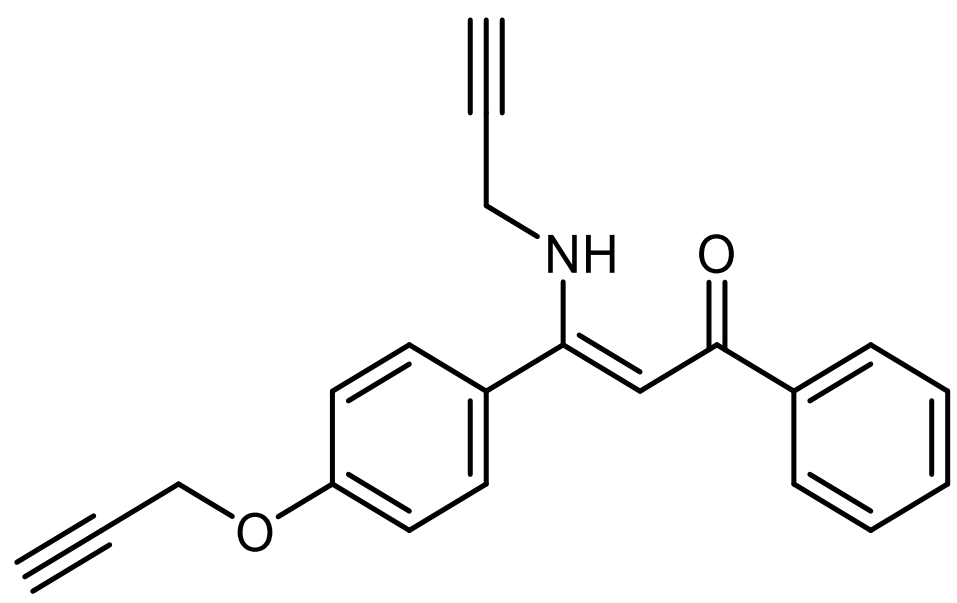

1ac

(Z)-1-phenyl-3-(prop-2-yn-1-ylamino)-3-(4-(prop-2-yn-1-yloxy)phenyl)prop-2-en-1-one (**1ac**) A mixture of prop-2-yn-1-amine (12 mmol), **S14** (10 mmol), and anhydrous ethanol (10 mL) was stirred at 60 °C under air for 24 hours. Once the reaction was complete (monitored by TLC), the solvent was removed under reduced pressure. The residue was purified by silica gel flash column chromatography (PE: EA = 8:1), yielding **1ac** as a brown solid (2.8 g, 90% yield). Mp: 105–108 ºC. ^{1}H NMR (400 MHz, $CDCl_3$): δ = 11.33 (s, 1H), 7.93–7.86 (m, 2H), 7.50–7.36 (m, 5H), 7.10–7.03 (m, 2H), 5.83 (s, 1H), 4.75 (d, J = 2.4 Hz, 2H), 3.97 (dd, J = 6.4, 2.5 Hz, 2H), 2.57 (t, J = 2.4 Hz, 1H), 2.32 (t, J = 2.5 Hz, 1H). ^{13}C NMR (101 MHz, $CDCl_3$): δ = 188.9, 165.5, 158.7, 140.0, 130.9, 129.4, 128.2, 128.0, 127.1, 114.9, 94.6, 79.8, 78.0, 76.0, 72.4, 55.8, 34.2. HRMS (ESI): m/z $[M + H]^+$ calcd for: $C_{21}H_{18}NO_2$: 316.1338; found: 316.1340.

(d) Procedure B for the synthesis of thiocyanated enaminones 2

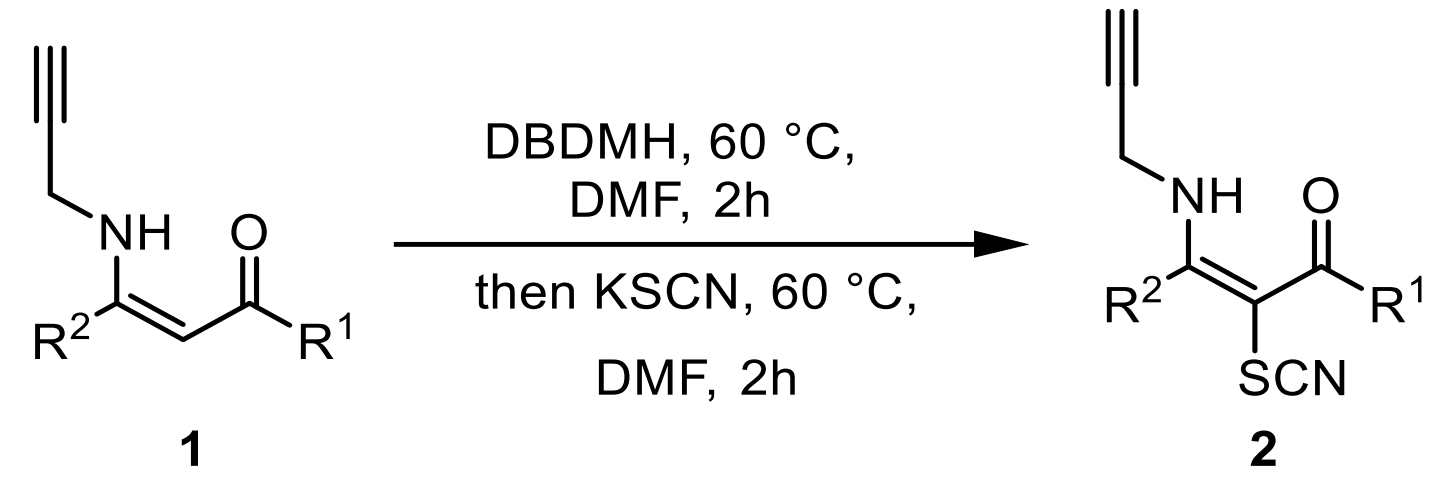

To a solution of N-propargyl-β-enaminone **1** (2 mmol) in DMF (1 mL) was added dropwise a solution of DBDMH (1 mmol) in DMF (1 mL). The reaction mixture was stirred at room temperature for 30 min and then heated at 60 °C for 2 h. Subsequently, a solution of KSCN (2 mmol) in DMF (4 mL) was added dropwise, and the reaction mixture was stirred at 60 °C for an additional 2 h. After completion of the reaction, the mixture was cooled to room temperature, and saturated aqueous sodium thiosulfate (30 mL) and ethyl acetate (30 mL) were added. The organic phase was washed with saturated brine (30 mL), dried over anhydrous Na_2SO_4, filtered, and concentrated under reduced pressure. The residue was purified by column chromatography to give compound **2**.

Synthesis of 2a

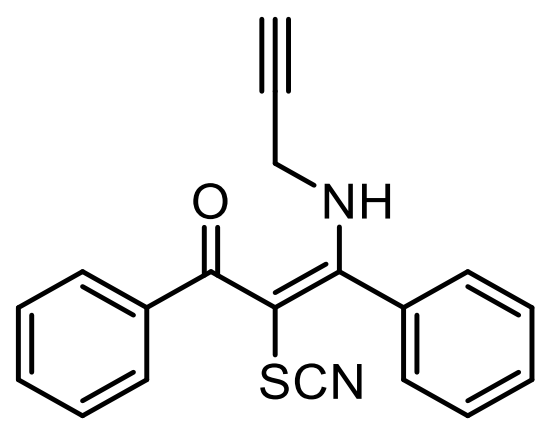

(E)-1,3-diphenyl-3-(prop-2-yn-1-ylamino)-2-thiocyanatoprop-2-en-1-one (**2a**). General procedure B was followed and purification by flash column chromatography afforded **2a** as a brown solid (PE: EA = 6:1 to 3:1). Yield: 566 mg, 89%. mp: 128–130 °C. ^1H NMR (400 MHz, $CDCl_3$): δ = 7.28–7.21 (m, 5H), 7.20–7.13 (m, 3H), 7.04-6.95 (m, 2H), 4.32 (d, J = 2.4 Hz, 2H), 2.30 (t, J = 2.4 Hz, 1H). ^{13}C NMR (101 MHz, $CDCl_3$): δ = 187.7, 162.0, 147.4, 138.0, 130.9, 130.1, 130.0, 129.2, 128.4, 128.1, 127.5, 114.9, 77.9, 72.2, 34.7. HRMS (ESI): m/z $[M + H]^+$ calcd for: $C_{19}H_{15}N_2OS$: 319.0905; found: 319.0909.

(e) General procedure C for the synthesis of 3,4-dihydro-2H-1,4-thiazines 3a-3an

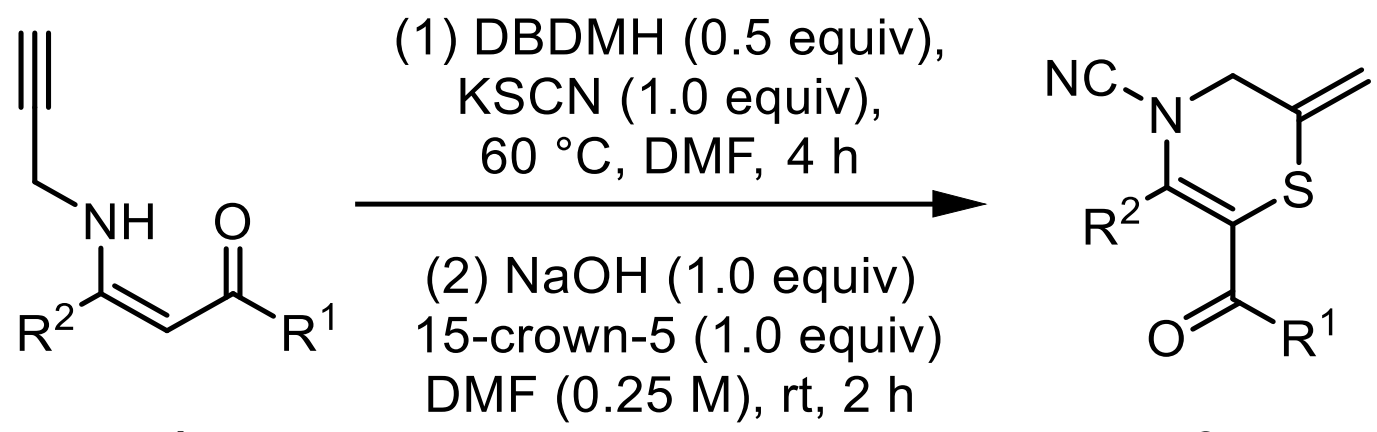

To a solution of N-propargyl-*β*-enaminone **1** (2 mmol) in DMF (1 mL) was added dropwise a solution of DBDMH (1 mmol) in DMF (1 mL). The reaction mixture was stirred at room temperature for 30 min and then heated at 60 °C for 2 h. Subsequently, a solution of KSCN (2 mmol) in DMF (4 mL) was added dropwise, and the reaction mixture was stirred at 60 °C for an additional 2 h. After completion of the reaction, the mixture was cooled to room temperature, and ethyl acetate (30 mL) were added. The organic phase was washed with saturated brine (30 mL), dried over anhydrous Na_2SO_4, filtered, and concentrated under reduced pressure to yield a brown liquid that was carried forward without further purification.

The residue of the step 1 was dissolved in DMF (4 mL), and NaOH (2 mmol) and 15-crown-5 (2 mmol) were subsequently added. The reaction mixture was stirred at room temperature for 2 hours. Upon completion, ethyl acetate (20 mL) was added, and the mixture was washed three times with saturated brine. The organic phase was separated, dried over anhydrous sodium sulfate, filtered, and concentrated to yield the crude product. The crude product was further purified by flash column chromatography to obtain compound **3**.

Synthesis of 3a

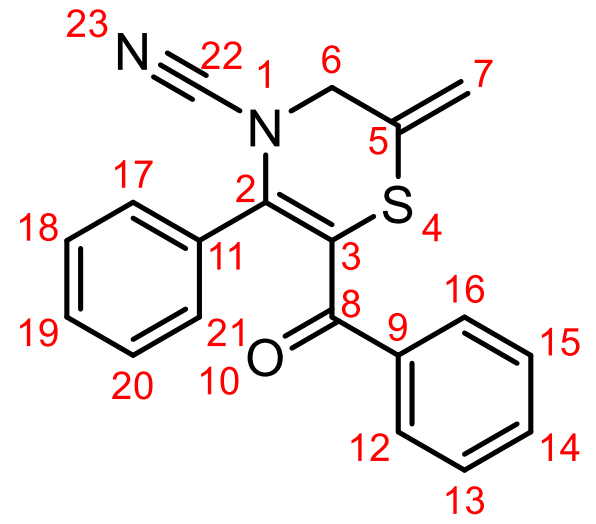

6-benzoyl-2-methylene-5-phenyl-2,3-dihydro-4H-1,4-thiazine-4-carbonitrile (**3a**). General procedure C was followed and purification by flash column chromatography afforded **3a** as a yellow solid (PE: EA = 8:1 to 6:1). Yield: 451 mg, 71%; mp: 163–165 ºC. ^{1}H NMR (400 MHz, $CDCl_3$): δ = 7.59–7.53 (m, 2H), 7.31–7.27 (m, 1H), 7.25–7.20 (m, 2H), 7.18–7.11 (m, 5H), 5.55 (d, J = 1.1 Hz, 1H), 5.38 (d, J = 1.4 Hz, 1H), 4.38 (s, 2H). ^{13}C NMR (101 MHz, $CDCl_3$): δ= 192.8, 140.6, 136.6, 132.8, 132.7, 132.0, 130.8, 130.0, 129.3, 128.6, 128.0, 117.3, 113.0, 112.6, 54.9. HRMS (ESI): m/z $[M+H]^+$ calcd for: $C_{19}H_{15}N_2OS$: 319.0905; found: 319.0908.

Synthesis of 3b

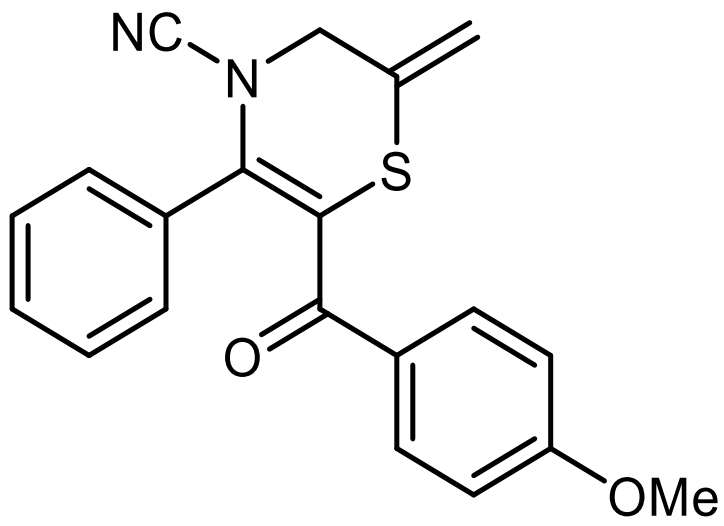

6-(4-methoxybenzoyl)-2-methylene-5-phenyl-2,3-dihydro-4H-1,4-thiazine-4-carbonitrile (**3b**). General procedure C was followed and purification by flash column chromatography afforded **3b** as a yellow solid (PE: EA = 8:1 to 6:1). Yield: 445 mg, 64%; mp:149–151 ºC. ^{1}H NMR (400 MHz, $CDCl_3$): δ = 7.66–7.61 (m, 2H), 7.29–7.26 (m, 2H), 7.21–7.16 (m, 3H), 6.71–6.67 (m, 2H), 5.53 (d, J = 1.2 Hz, 1H), 5.37 (d, J = 1.1 Hz, 1H), 4.36 (s, 2H), 3.76 (s, 3H).^{13}C NMR (101 MHz, $CDCl_3$) : δ = 191.0, 163.5, 138.5, 132.8, 132.1, 131.9, 130.6, 129.7, 129.0, 128.7, 117.5, 113.4, 112.9, 112.8, 55.4, 54.7. HRMS (ESI): m/z $[M + H]^+$ calcd for: $C_{20}H_{17}N_2O_2S$: 349.1011; found: 349.1013.

Synthesis of 3c

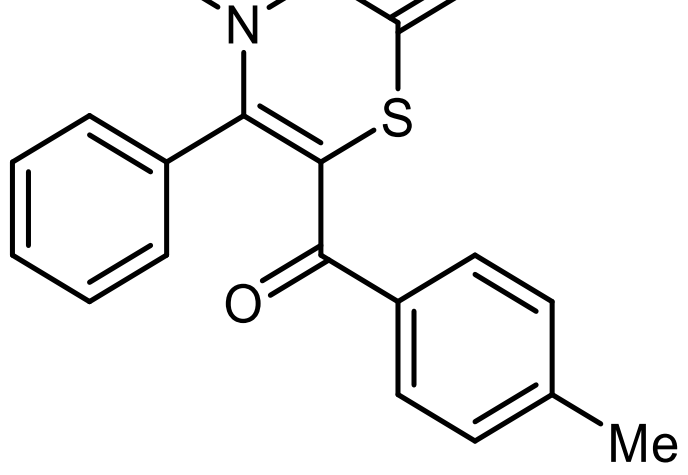

6-(4-methylbenzoyl)-2-methylene-5-phenyl-2,3-dihydro-4H-1,4-thiazine-4-

carbonitrile (**3c**). General procedure C was followed and purification by flash column chromatography afforded **3c** as a yellow solid (PE: EA = 8: 1 to 6:1). Yield: 498 mg, 75%; mp 160–162 ºC. ^{1}H NMR (400 MHz, $CDCl_3$): δ = 7.51 (d, J = 8.2 Hz, 2H), 7.26–7.23 (m, 2H), 7.20–7.13 (m, 3H), 6.99 (d, J = 7.9 Hz, 2H), 5.54 (d, J = 1.2 Hz, 1H), 5.37 (d, J = 1.2 Hz, 1H), 4.37 (s, 2H), 2.26 (s, 3H). ^{13}C NMR (101 MHz, $CDCl_3$): δ = 192.3, 143.9, 139.5, 133.8, 132.8, 132.1, 130.7, 129.8, 129.6, 128.8, 128.6, 117.5, 112.8, 112.7, 54.8, 21.6. HRMS (ESI): m/z $[M + H]^+$ calcd for: $C_{20}H_{17}N_2OS$:333.1062; found: 333.1065.

Synthesis of 3d

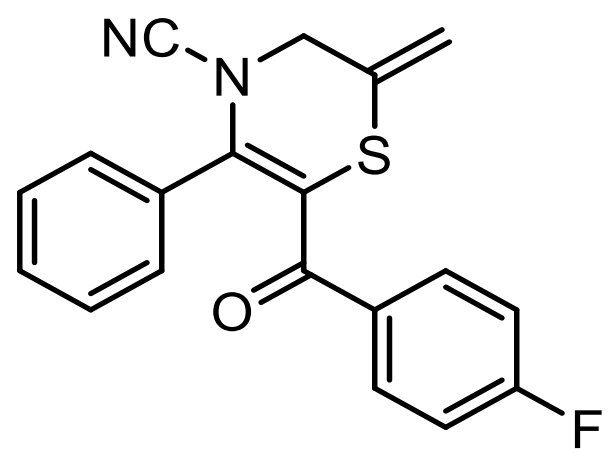

6-(4-fluorobenzoyl)-2-methylene-5-phenyl-2,3-dihydro-4H-1,4-thiazine-4-carbonitrile (**3d**). General procedure C was followed and purification by flash column chromatography afforded **3d** as a yellow solid (PE: EA = 8:1 to 6:1). Yield: 403 mg, 60%; mp: 112–114 ºC. ^{1}H NMR (400 MHz, $CDCl_3$): δ = 7.62–7.55 (m, 2H), 7.25–7.14 (m, 5H), 6.83 (t, J = 8.6 Hz, 2H), 5.56 (s, 1H), 5.39 (s, 1H), 4.38 (s, 2H). ^{13}C NMR (101 MHz, $CDCl_3$): δ = 191.3, 165.2 (d, $J_{C\text{-}F}$ = 254 Hz), 140.6, 132.9 (d, $J_{C\text{-}F}$ = 3 Hz), 132.6, 131.9, 131.8 (d, $J_{C\text{-}F}$ = 9 Hz), 131.0, 129.9, 128.8, 117.1, 115.2 (d, $J_{C\text{-}F}$ = 22 Hz), 113.1, 112.5, 54.9. ^{19}F NMR (376 MHz, $CDCl_3$): δ = -104.67 (s, F). HRMS (ESI): m/z $[M + H]^+$ calcd for: $C_{19}H_{14}N_2OSF$:337.0811; found: 337.0816.

Synthesis of 3e

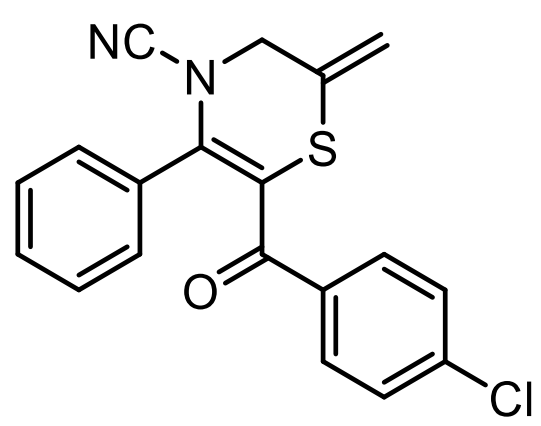

6-(4-chlorobenzoyl)-2-methylene-5-phenyl-2,3-dihydro-4H-1,4-thiazine-4-carbonitrile(**3e**). General procedure C was followed and purification by flash column chromatography afforded **3e** as a yellow solid (PE: EA = 8:1 to 6:1). Yield: 429 mg, 61%; mp: 178 ºC. ^{1}H NMR (400 MHz, $CDCl_3$): δ =7.51–7.47 (m, 2H), 7.25–7.21 (m, 3H), 7.20–7.16 (m, 2H), 7.15–7.11 (m, 2H), 5.57 (d, J = 1.1 Hz, 1H), 5.40 (d, J = 1.2 Hz, 1H), 4.39 (s, 2H). ^{13}C NMR (101 MHz, $CDCl_3$): δ = 191.6, 141.0, 139.0, 135.0, 132.5, 131.9, 131.2, 130.6, 130.0, 128.8, 128.3, 116.9, 113.2, 112.5, 54.9. HRMS (ESI): m/z $[M + H]^+$ calcd for:$C_{19}H_{14}N_2OSCl$: 353.0515; found: 353.0518.

Synthesis of 3f

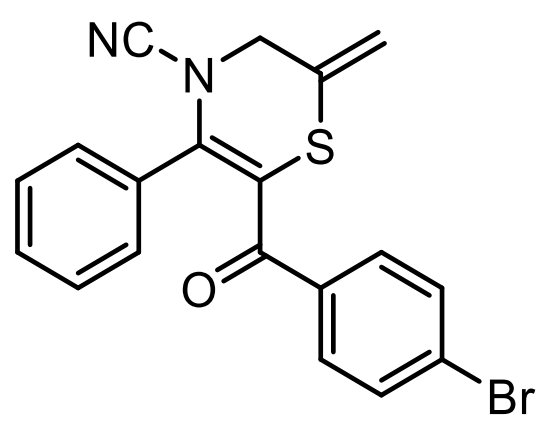

6-(4-bromobenzoyl)-2-methylene-5-phenyl-2,3-dihydro-4H-1,4-thiazine-4-carbonitrile(**3f**). General procedure C was followed and purification by flash column chromatography afforded **3f** as a yellow solid (PE: EA = 8:1). Yield: 498 mg, 63%; mp: 174–175 ºC. ^{1}H NMR (400 MHz, $CDCl_3$): δ = 7.42–7.38 (m, 2H), 7.31–7.26 (m, 2H), 7.24–7.14 (m, 5H), 5.56 (d, J = 1.2 Hz, 1H), 5.39 (d, J = 1.1 Hz, 1H), 4.38 (s, 2H). ^{13}C NMR (101 MHz, $CDCl_3$): δ = 191.7, 141.1, 135.4, 132.5, 131.8, 131.3, 131.1, 130.6, 130.0, 128.8, 127.7, 116.9, 113.2, 112.4, 54.8. HRMS (ESI): m/z $[M + H]^+$ calcd for: $C_{19}H_{14}BrN_2OS$: 397.0010; found: 397.0008.

Synthesis of 3g

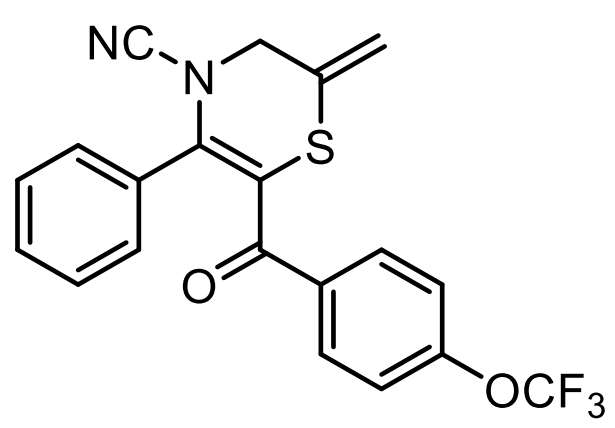

2-methylene-5-phenyl-6-(4-(trifluoromethoxy)benzoyl)-2,3-dihydro-4H-1,4-thiazine-4-

carbonitrile (**3g**). General procedure C was followed and purification by flash column chromatography afforded **3g** as a yellow solid (PE: EA = 8:1). Yield: 498 mg, 62%; mp: 136–139 ºC. ^{1}H NMR (400 MHz, $CDCl_3$): δ = 7.57–7.53 (m, 2H), 7.23–7.12 (m, 5H), 6.97–6.93 (m, 2H), 5.58 (d, J = 1.3 Hz, 1H), 5.41 (d, J = 1.2 Hz, 1H), 4.40 (d, J = 0.9 Hz, 2H). ^{13}C NMR (101 MHz, $CDCl_3$): δ = 191.4, 151.8, 141.8, 135.1, 132.5, 132.2, 131.9, 131.2, 131.1, 130.2, 128.8, 119.9, 117.1, 113.3, 112.4, 54.9. ^{19}F NMR (376 MHz, $CDCl_3$): δ = -57.72 (s, 3F). HRMS (ESI): m/z $[M + H]^+$ calcd for: $C_{20}H_{14}N_2O_2SF_3$: 403.0728; found: 403.0728.

Synthesis of 3h

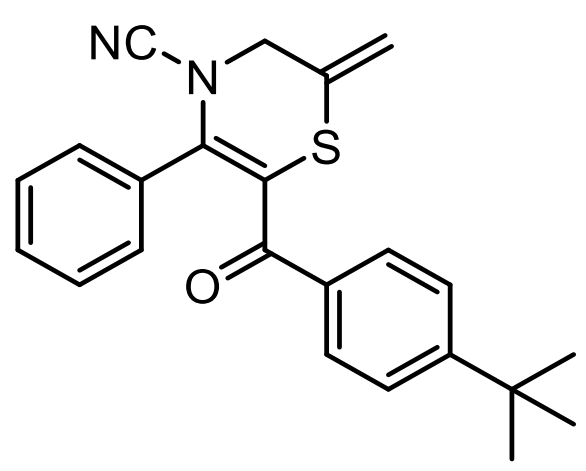

6-(4-(tert-butyl)benzoyl)-2-methylene-5-phenyl-2,3-dihydro-4H-1,4-thiazine-4-carbonitrile (**3h**). General procedure C was followed and purification by flash column chromatography afforded **3h** as a yellow solid (PE: EA = 8:1 to 6:1). Yield: 374 mg, 50%; mp: 175–176 ºC. ^{1}H NMR (400 MHz, $CDCl_3$): δ = 7.55–7.49 (m, 2H), 7.25–7.20 (m, 2H), 7.20–7.11 (m, 5H), 5.53 (d, J = 1.0 Hz, 1H), 5.37 (d, J = 4.0 Hz, 1H), 4.37 (s, 2H), 1.21 (s, 9H). ^{13}C NMR (101 MHz, $CDCl_3$): δ = 192.3, 156.5, 140.0, 133.7, 132.9, 132.1, 130.6, 129.9, 129.4, 128.5, 124.9, 117.6, 112.7, 54.8, 34.9, 30.8. HRMS (ESI): m/z $[M + H]^+$ calcd for: $C_{23}H_{23}N_2OS$: 375.1531; found: 375.1535.

Synthesis of 3i

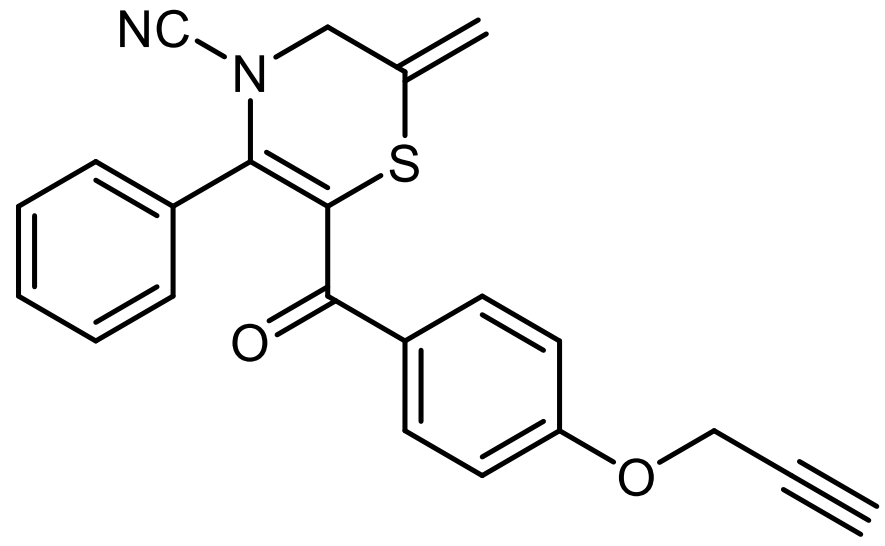

3i

2-methylene-5-phenyl-6-(4-(prop-2-yn-1-yloxy)benzoyl)-2,3-dihydro-4H-1,4-thiazine-4-carbonitrile (**3i**). General procedure C was followed and purification by flash column chromatography afforded **3i** as a yellow solid. Yield: 312 mg, 42%; mp: 104–107 ºC. ^{1}H NMR (400 MHz, $CDCl_3$): δ = 7.66–7.60 (m, 2H), 7.29–7.24 (m, 2H), 7.22–7.14 (m, 3H), 6.76 (d, J = 8.9 Hz, 2H), 5.54 (d, J = 1.2 Hz, 1H), 5.37 (d, J = 1.2 Hz, 1H), 4.64 (d, J = 2.4 Hz, 2H), 4.37 (s, 2H), 2.52 (t, J = 2.4 Hz, 1H). ^{13}C NMR (101 MHz, $CDCl_3$): δ = 191.0, 161.2, 139.0, 132.7, 132.1, 131.7, 130.7, 129.8, 129.7, 128.6, 117.4, 114.2, 112.8, 112.7, 77.5, 76.1, 55.8, 54.7. HRMS (ESI): m/z $[M + H]^+$ calcd for: $C_{22}H_{17}N_2O_2S$: 373.1011; found: 373.1015.

Synthesis of 3j

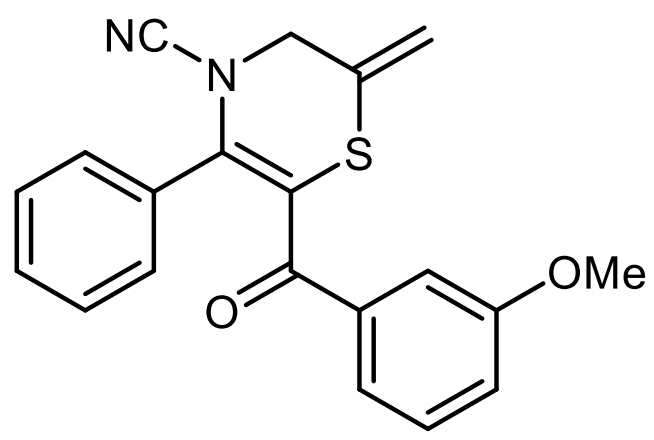

6-(3-methoxybenzoyl)-2-methylene-5-phenyl-2,3-dihydro-4H-1,4-thiazine-4-carbonitrile (**3j**). General procedure C was followed and purification by flash column chromatography afforded **3j** as a yellow solid (PE: EA = 8:1 to 6:1). Yield: 417 mg, 60%; mp: 143–146 ºC. ^{1}H NMR (400 MHz, $CDCl_3$): δ = 7.26–7.23 (m, 2H), 7.22–7.13 (m, 4H), 7.10–7.04 (m, 2H), 6.87–6.82 (m, 1H), 5.55 (d, J = 1.0 Hz, 1H), 5.38 (d, J = 0.8 Hz, 1H), 4.38 (s, 2H), 3.74 (s, 3H). ^{13}C NMR (101 MHz, $CDCl_3$): δ = 192.5, 159.1, 140.5, 137.8, 132.8, 132.1, 130.8, 130.0, 129.0, 128.6, 122.2, 119.2, 117.4, 113.7, 112.9, 112.6, 55.4, 54.9. HRMS (ESI): m/z $[M + H]^+$ calcd for: $C_{20}H_{17}N_2O_2S$:349.1011; found: 349.1011.

Synthesis of 3k

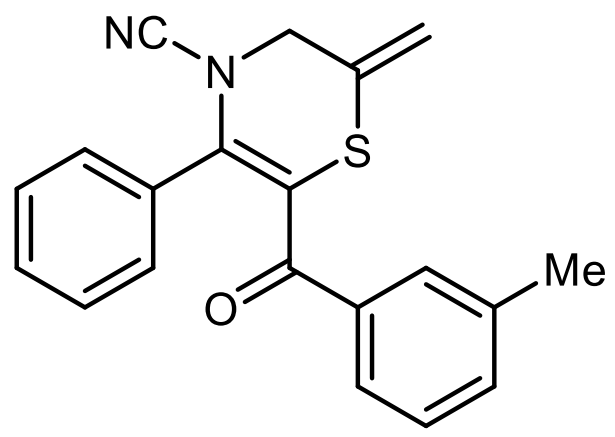

6-(3-methylbenzoyl)-2-methylene-5-phenyl-2,3-dihydro-4H-1,4-thiazine-4-carbonitrile (**3k**). General procedure C was followed and purification by flash column chromatography afforded **3k** as a yellow solid (PE: EA = 8:1 to 6:1). Yield: 438 mg, 66%; mp: 144–146 °C. ^{1}H NMR (400 MHz, $CDCl_3$): δ = 7.40–7.33 (m, 2H), 7.25–7.22 (m, 2H), 7.17– 7.03 (m, 5H), 5.54 (d, J = 0.8 Hz, 1H), 5.37 (d, J = 0.7 Hz, 1H), 4.38 (s, 2H), 2.23 (s, 3H). ^{13}C NMR (101 MHz, $CDCl_3$): δ = 192.8, 140.5, 137.7, 136.4, 133.5, 132.9, 132.1, 130.8, 129.9, 129.8, 128.6, 127.9, 126.6, 117.5, 112.8, 112.7, 54.9, 21.1. HRMS (ESI): m/z $[M + H]^+$ calcd for: $C_{20}H_{17}N_2OS$: 333.1062; found: 333.1065.

Synthesis of 3l

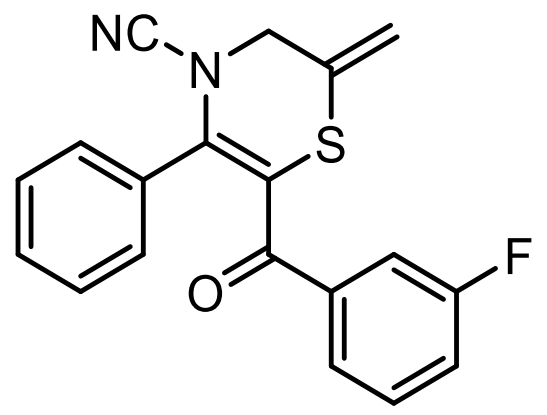

6-(3-fluorobenzoyl)-2-methylene-5-phenyl-2,3-dihydro-4H-1,4-thiazine-4-carbonitrile (**3l**). General procedure C was followed and purification by flash column chromatography afforded **3l** as a yellow solid (PE: EA = 8:1 to 6:1). Yield: 322 mg, 48%; mp: 154–157 °C. ^{1}H NMR (400 MHz, $CDCl_3$): δ = 7.33–7.28 (m, 1H), 7.25–7.08 (m, 7H), 6.99-6.93 (m, 1H), 5.57 (s, 1H), 5.40 (s, 1H), 4.40 (s, 2H). ^{13}C NMR (101 MHz, $CDCl_3$): δ = 191.6, 161.9 (d, J_{C-F} = 246 Hz), 141.7, 138.8 (d, J_{C-F} = 6 Hz), 132.6, 131.9, 131.2, 130.1, 129.6 (d, J_{C-F} = 8 Hz), 128.7, 124.9 (d, J_{C-F} = 3 Hz), 119.5 (d, J_{C-F} = 21Hz), 116.9, 115.8 (d, J_{C-F} = 22Hz), 113.2, 112.4, 54.9. ^{19}F NMR (376 MHz, $CDCl_3$): δ = -112.48 (s, F). HRMS (ESI): m/z $[M + H]^+$ calcd for: $C_{19}H_{14}FN_2OS$: 337.0811; found: 337.0813.

Synthesis of 3m

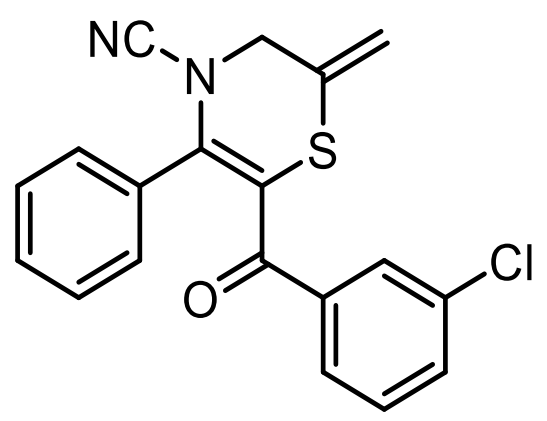

6-(3-chlorobenzoyl)-2-methylene-5-phenyl-2,3-dihydro-4H-1,4-thiazine-4-carbonitrile(**3m**). The general procedure C was followed and purification by flash column chromatography afforded **3m** as a yellow solid (PE: EA = 8:1 to 6:1). Yield: 366 mg, 52%; mp:182–184 ºC. ^{1}H NMR (400 MHz, CDCl$_3$): δ = 7.43–7.38 (m, 2H), 7.25–7.16 (m, 6H), 7.08 (t, J = 7.8 Hz, 1H), 5.58 (d, J = 1.0 Hz, 1H), 5.40 (d, J = 0.9 Hz, 1H), 4.41 (s, 2H). ^{13}C NMR (101 MHz, CDCl$_3$): δ = 191.5, 142.2, 138.3, 133.9, 132.6, 132.3, 131.9, 131.3, 130.2, 129.3, 129.2, 128.8, 127.1, 116.9, 113.2, 112.3, 54.9. HRMS (ESI): m/z [M + H]$^+$ calcd for: $C_{19}H_{14}N_2OSCl$: 353.0515; found: 353.0509.

Synthesis of 3n

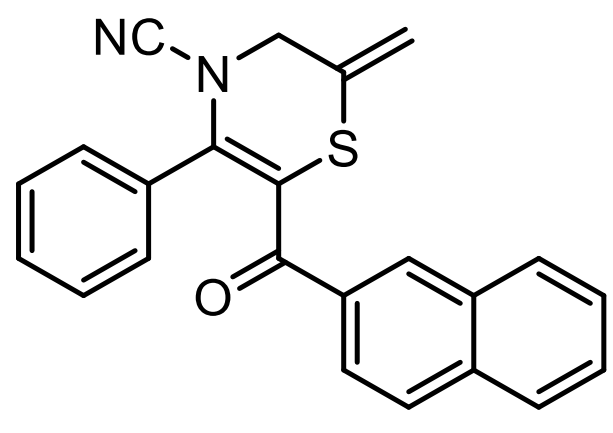

6-(2-naphthoyl)-2-methylene-5-phenyl-2,3-dihydro-4H-1,4-thiazine-4-carbonitrile (**3n**). General procedure C was followed and purification by flash column chromatography afforded **3n** as a yellow solid (PE: EA = 8:1 to 6:1). Yield: 456 mg, 62%; mp: 150–151 ºC. ^{1}H NMR (400 MHz, CDCl$_3$): δ = 7.68–7.64 (m, 2H), 7.51–7.47 (m, 2H), 7.46–7.37 (m, 5H), 7.29–7.27 (m, 1H), 7.17–7.15 (m, 2H), 5.57 (d, J = 1.2 Hz, 1H), 5.40 (d, J = 1.1 Hz, 1H), 4.41 (s, 2H). ^{13}C NMR (101 MHz, CDCl$_3$): δ = 192.3, 145.4, 140.4, 139.6, 135.2, 132.8, 132.1, 130.8, 130.0, 129.9, 128.9, 128.7, 128.2, 127.1, 126.6, 117.4, 112.9, 112.7, 54.9. HRMS (ESI): m/z [M + H]$^+$ calcd for: $C_{23}H_{17}N_2OS$: 369.1062; found: 369.1066.

Synthesis of 3o

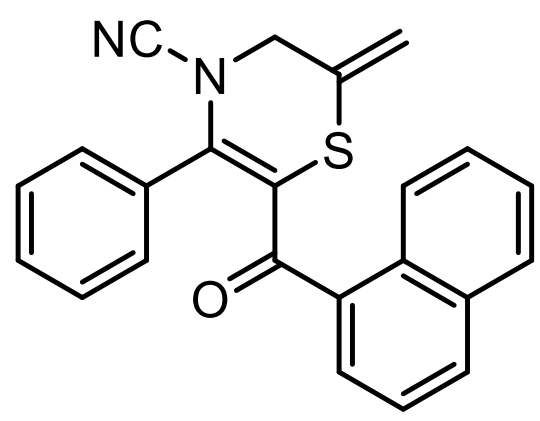

6-(1-naphthoyl)-2-methylene-5-phenyl-2,3-dihydro-4H-1,4-thiazine-4-carbonitrile (**3o**). General procedure C was followed and purification by flash column chromatography afforded **3o** as a yellow solid (PE: EA = 8:1 to 6:1). Yield: 345 mg, 47%; mp: 150–152 ºC. ^{1}H NMR (400 MHz, $CDCl_3$): δ = 8.23 (dd, J = 8.6, 1.1 Hz, 1H), 7.68–7.60 (m, 2H), 7.56–7.41 (m, 3H), 7.16–7.01 (m, 3H), 6.87–6.75 (m, 3H), 5.61 (d, J = 1.0 Hz, 1H), 5.40 (d, J = 1.1 Hz, 1H), 4.42 (s, 2H). ^{13}C NMR (101 MHz, $CDCl_3$): δ = 193.8, 144.2, 135.8, 133.2, 133.0, 132.1, 132.0, 130.5, 130.3, 129.8, 128.3, 128.0, 127.5, 126.2, 125.1, 123.7, 119.7, 113.1, 112.2, 55.0. HRMS (ESI): m/z $[M + H]^+$ calcd for: $C_{23}H_{17}N_2OS$: 369.1062; found: 369.1059.

Synthesis of 3p

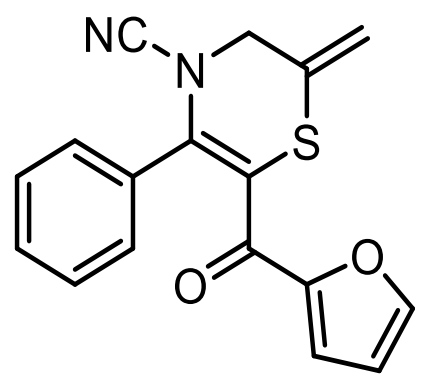

6-(furan-2-carbonyl)-2-methylene-5-phenyl-2,3-dihydro-4H-1,4-thiazine-4-carbonitrile (**3p**). General procedure C was followed and purification by flash column chromatography afforded **3p** as a yellow solid (PE: EA = 8:1 to 6:1). Yield: 396 mg, 60%; mp:128–130 ºC. ^{1}H NMR (400 MHz, $CDCl_3$): δ = 7.37–7.33 (m, 2H), 7.29–7.26 (m, 3H), 7.26–7.22 (m, 1H), 6.89 (dd, J = 3.6, 0.6 Hz, 1H), 6.24 (dd, J = 3.6, 1.7 Hz, 1H), 5.54 (d, J = 1.0 Hz, 1H), 5.37 (d, J = 0.8 Hz, 1H), 4.35 (s, 2H). ^{13}C NMR (101 MHz, $CDCl_3$): δ = 179.4, 151.1, 146.7, 140.9, 132.9, 132.3, 130.8, 129.6, 128.7, 119.7, 116.6, 112.9, 112.6, 112.3, 54.9. HRMS (ESI): m/z $[M+Na]^+$ calcd for:$C_{17}H_{12}N_2O_2NaS$: 331.0517; found: 331.0522.

Synthesis of 3q

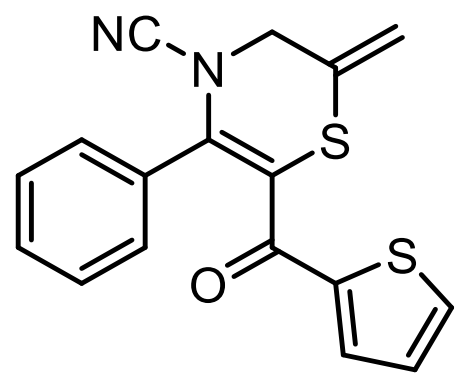

2-methylene-5-phenyl-6-(thiophene-2-carbonyl)-2,3-dihydro-4H-1,4-thiazine-4-carbonitrile (**3q**). General procedure C was followed and purification by flash column chromatography afforded **3q** as a yellow solid (PE: EA = 8:1 to 6:1). Yield: 401 mg, 62%; mp: 163–166 ºC. ^{1}H NMR (400 MHz, $CDCl_3$): δ = 7.48–7.45 (m, 1H), 7.40–7.37 (m, 1H), 7.37–7.33 (m, 2H), 7.25–7.22 (m, 2H), 6.84 (dd, J = 4.9, 3.9 Hz, 1H), 5.55 (d, J = 1.1 Hz, 1H), 5.39 (d, J = 0.9 Hz, 1H), 4.36 (s, 2H). ^{13}C NMR (101 MHz, $CDCl_3$): δ = 184.2, 142.4, 138.8, 134.9, 134.6, 132.6, 132.2, 130.8, 129.7, 128.8, 127.7, 117.6, 113.1, 112.7, 54.8. HRMS (ESI): m/z $[M + H]^+$ calcd for:$C_{17}H_{13}N_2OS_2$: 325.0469; found: 325.0470.

Synthesis of 3r

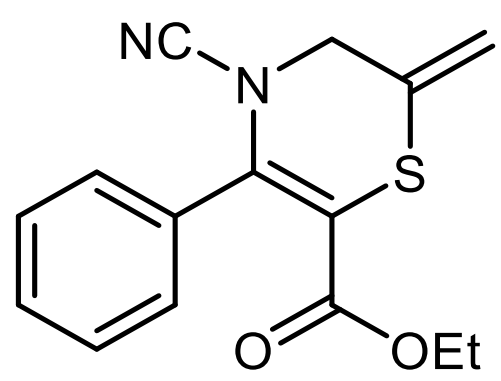

ethyl 4-cyano-2-methylene-5-phenyl-3,4-dihydro-2H-1,4-thiazine-6-carboxylate (**3r**). General procedure C was followed and purification by flash column chromatography afforded **3r** as a yellow solid (PE: EA = 8:1 to 6:1). Yield: 343 mg, 60%; mp: 112–113 ºC. ^{1}H NMR (400 MHz, $CDCl_3$): δ = 7.47–7.40 (m, 3H), 7.37–7.33 (m, 2H), 5.53 (s, 1H), 5.33 (s, 1H), 4.28 (s, 2H), 3.97 (q, J = 7.1 Hz, 2H), 0.92 (t, J = 7.1 Hz, 3H). ^{13}C NMR (101 MHz, $CDCl_3$): δ = 163.9, 142.6, 133.2, 132.1, 130.5, 129.2, 128.6, 113.1, 112.2, 108.6, 61.6, 54.6, 13.4. HRMS (ESI): m/z $[M + H]^+$ calcd for: $C_{15}H_{15}N_2O_2S$: 287.0854; found: 287.0856.

Synthesis of 3s

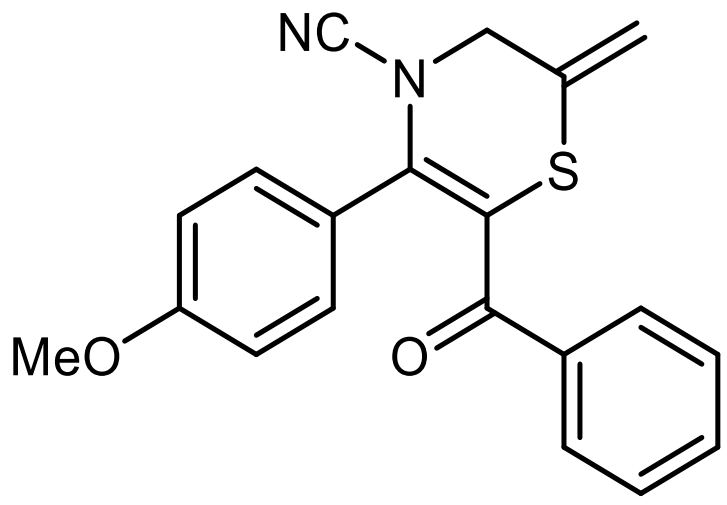

6-benzoyl-5-(4-methoxyphenyl)-2-methylene-2,3-dihydro-4H-1,4-thiazine-4-carbonitrile (**3s**). General procedure C was followed and purification by flash column chromatography afforded **3s** as a yellow solid (PE: EA = 8:1 to 6:1). Yield: 417 mg, 60%; mp: 67–70 ºC. ^{1}H NMR (400 MHz, $CDCl_3$): δ = 7.60–7.49 (m, 2H), 7.31–7.26 (m, 1H), 7.19–7.13 (m, 4H), 6.67–6.61 (m, 2H), 5.53 (d, J = 0.8 Hz, 1H), 5.36 (d, J = 0.7 Hz, 1H), 4.37 (s, 2H), 3.67 (s, 3H). ^{13}C NMR (101 MHz, $CDCl_3$): δ = 193.0, 161.5, 141.5, 136.8, 133.4, 132.5, 131.7, 129.2, 127.9, 124.4, 115.9, 114.1, 112.8, 112.5, 55.3, 55.1. HRMS (ESI): m/z $[M + H]^+$ calcd for: $C_{20}H_{17}N_2O_2S$: 349.1011; found: 349.1015.

Synthesis of 3t

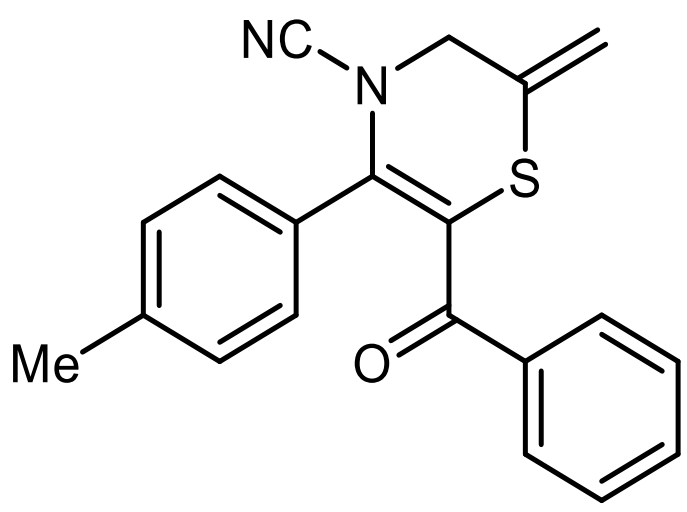

6-benzoyl-2-methylene-5-(p-tolyl)-2,3-dihydro-4H-1,4-thiazine-4-carbonitrile (**3t**). General procedure C was followed and purification by flash column chromatography afforded **3t** as a yellow solid (PE: EA = 8:1 to 6:1). Yield: 365 mg, 55%; mp: 129–132 ºC. ^{1}H NMR (400 MHz, $CDCl_3$): δ = 7.59–7.53 (m, 2H), 7.33–7.27 (m, 1H), 7.20–7.08 (m, 4H), 6.93 (d, J = 7.9 Hz, 2H), 5.53 (d, J = 0.8 Hz, 1H), 5.36 (d, J = 0.6 Hz, 1H), 4.37 (s, 2H), 2.17 (s, 3H). ^{13}C NMR (101 MHz, $CDCl_3$): δ = 192.9, 141.3, 141.1, 136.7, 133.1, 132.6, 129.9, 129.4, 129.3, 129.2, 127.9, 116.5, 112.7, 112.6, 54.9, 21.3. HRMS (ESI): m/z $[M + H]^+$ calcd for: $C_{20}H_{17}N_2OS$: 333.1062; found: 333.1063.

Synthesis of 3u

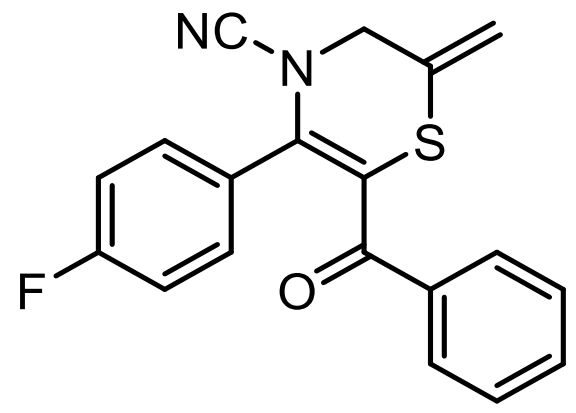

6-benzoyl-5-(4-fluorophenyl)-2-methylene-2,3-dihydro-4H-1,4-thiazine-4-carbonitrile (**3u**). General procedure C was followed and purification by flash column chromatography afforded **3u** as a yellow solid (PE: EA = 8:1 to 6:1). Yield: 383 mg, 57%; Mp: 165–168 °C. ^{1}H NMR (400 MHz, $CDCl_3$): δ = 7.58–7.53 (m, 2H), 7.37–7.31 (m, 1H), 7.25–7.17 (m, 4H), 6.88–6.80 (m, 2H), 5.57 (d, J = 1.1 Hz, 1H), 5.40 (d, J = 0.9 Hz, 1H), 4.38 (s, 2H). ^{13}C NMR (101 MHz, $CDCl_3$): δ = 192.6, 163.9 (d, $J_{C\text{-}F}$ = 252 Hz), 139.3, 136.5, 133.0, 132.6, 132.1(d, $J_{C\text{-}F}$ = 9 Hz), 129.3, 128.2 (d, $J_{C\text{-}F}$ = 3 Hz), 128.1, 117.6, 115.9 (d, $J_{C\text{-}F}$ = 22 Hz), 113.2, 112.5, 54.9. ^{19}F NMR (376 MHz, $CDCl_3$): δ = -107.92 (s, F). HRMS (ESI): m/z [M + H]$^+$ calcd for: $C_{19}H_{14}N_2OSF$: 337.0811; found: 337.0812.

Synthesis of 3v

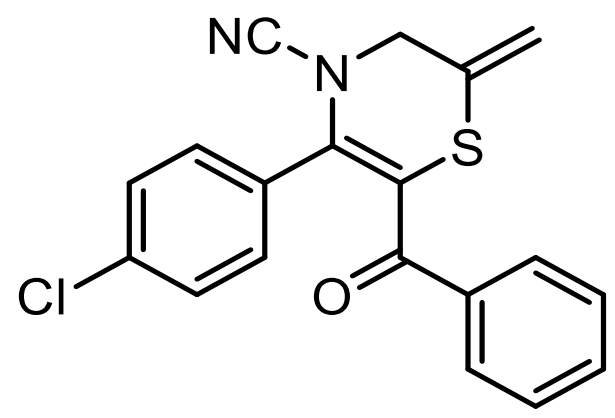

6-benzoyl-5-(4-chlorophenyl)-2-methylene-2,3-dihydro-4H-1,4-thiazine-4-carbonitrile (**3v**). General procedure C was followed and purification by flash column chromatography afforded **3v** as a yellow solid (PE: EA = 8:1 to 6:1). Yield: 387 mg, 55%; mp: 173–176 °C. ^{1}H NMR (400 MHz, $CDCl_3$): δ = 7.61–7.55 (m, 2H), 7.37 (t, J = 7.4 Hz, 1H), 7.24–7.10 (m, 6H), 5.57 (d, J = 0.8 Hz, 1H), 5.40 (s, 1H), 4.37 (s, 2H). ^{13}C NMR (101 MHz, $CDCl_3$): δ = 192.5, 138.8, 137.0, 136.3, 133.1, 132.4, 131.1, 130.5, 129.3, 129.0, 128.2, 118.2, 113.4, 112.5, 54.7. HRMS (ESI): m/z [M + H]$^+$ calcd for:

$C_{19}H_{14}N_2OSCl$: 353.0515; found: 353.0519.

Synthesis of 3w

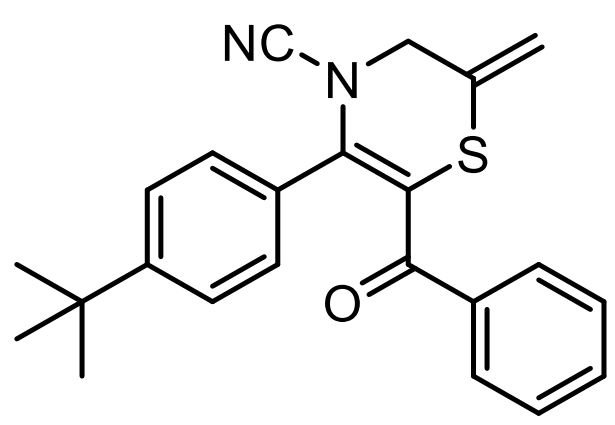

6-benzoyl-5-(4-(tert-butyl)phenyl)-2-methylene-2,3-dihydro-4H-1,4-thiazine-4-carbonitrile (**3w**). General procedure C was followed and purification by flash column chromatography afforded **3w** as a yellow solid (PE: EA = 8:1 to 6:1). Yield: 403 mg, 54%; mp: 173–177 ºC. ^{1}H NMR (400 MHz, $CDCl_3$): δ = 7.49–7.44 (m, 2H), 7.21 (t, J = 7.4 Hz, 1H), 7.15–7.06 (m, 6H), 5.54 (s, 1H), 5.37 (s, 1H), 4.39 (s, 2H), 1.13 (s, 9H). ^{13}C NMR (101 MHz, $CDCl_3$): δ = 193.1, 154.3, 141.9, 137.0, 133.2, 132.1, 130.0, 129.1, 129.0, 127.7, 125.5, 116.8, 112.7, 112.6, 55.0, 34.7, 30.8. HRMS (ESI): m/z $[M + H]^+$ calcd for: $C_{23}H_{23}N_2OS$: 375.1531; found: 375.1534.

Synthesis of 3x

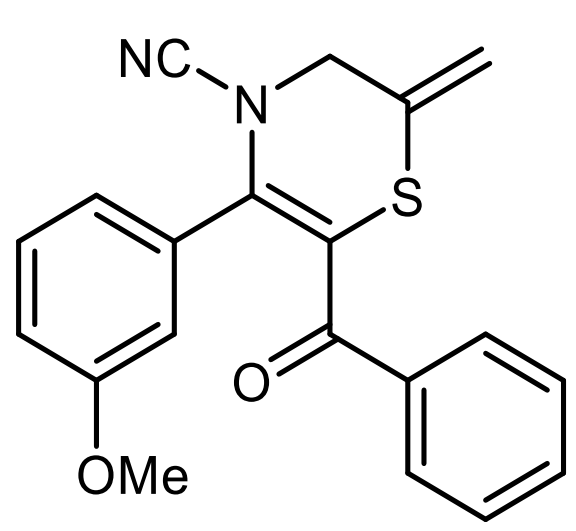

6-benzoyl-5-(3-methoxyphenyl)-2-methylene-2,3-dihydro-4H-1,4-thiazine-4-carbonitrile (**3x**). General procedure C was followed and purification by flash column chromatography afforded **3x** as a yellow solid (PE: EA = 8:1). Yield: 382 mg, 55%; mp: 79–81 ºC. ^{1}H NMR (400 MHz, $CDCl_3$): δ = 7.60–7.55 (m, 2H), 7.34–7.29 (m, 1H), 7.21–7.15 (m, 2H), 7.05 (t, J = 7.9 Hz, 1H), 6.88–6.83 (m, 1H), 6.74–6.67 (m, 2H), 5.55 (d, J = 1.2 Hz, 1H), 5.38 (d, J = 1.2 Hz, 1H), 4.37 (s, 2H), 3.65 (s, 3H). ^{13}C NMR

(101 MHz, $CDCl_3$) : δ = 192.8, 159.3, 140.3, 136.5, 133.2, 132.7, 129.7, 129.1, 128.0, 122.4, 117.4, 117.1, 115.1, 113.0, 112.6, 55.2, 54.8. HRMS (ESI): m/z $[M + H]^+$ calcd for: $C_{20}H_{17}N_2O_2S$: 349.1011; found: 349.1012.

Synthesis of 3y

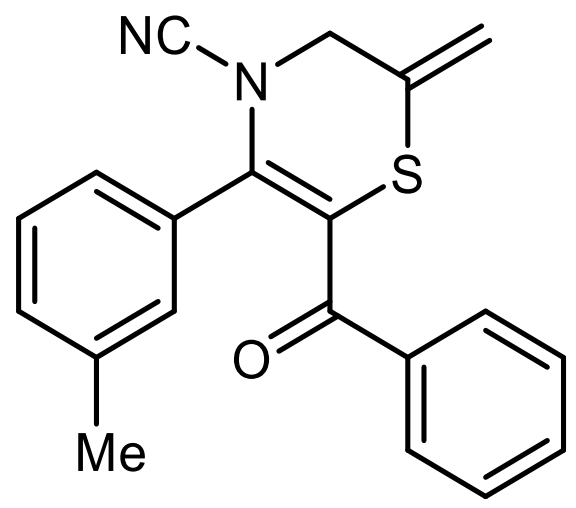

6-benzoyl-2-methylene-5-(m-tolyl)-2,3-dihydro-4H-1,4-thiazine-4-carbonitrile (**3y**). General procedure C was followed and purification by flash column chromatography afforded **3y** as a yellow solid (PE: EA = 9:1). Yield: 378 mg, 57%; mp: 127–129 ºC. ^1H NMR (400 MHz, $CDCl_3$): δ = 7.55–7.50 (m, 2H), 7.31–7.26 (m, 1H), 7.15 (t, J = 7.7 Hz, 2H), 7.07–6.98 (m, 3H), 6.97–6.92 (m, 1H), 5.54 (d, J = 1.1 Hz, 1H), 5.37 (d, J = 1.2 Hz, 1H), 4.38 (s, 2H), 2.15 (s, 3H). ^{13}C NMR (101 MHz, $CDCl_3$): δ = 193.0, 141.3, 138.3, 136.7, 133.0, 132.5, 131.9, 131.6, 131.0, 129.0, 128.5, 127.8, 127.0, 117.1, 112.7, 112.6, 54.9, 21.0. HRMS (ESI): m/z $[M + H]^+$ calcd for: $C_{20}H_{17}N_2OS$: 333.1062; found: 333.1067.

Synthesis of 3z

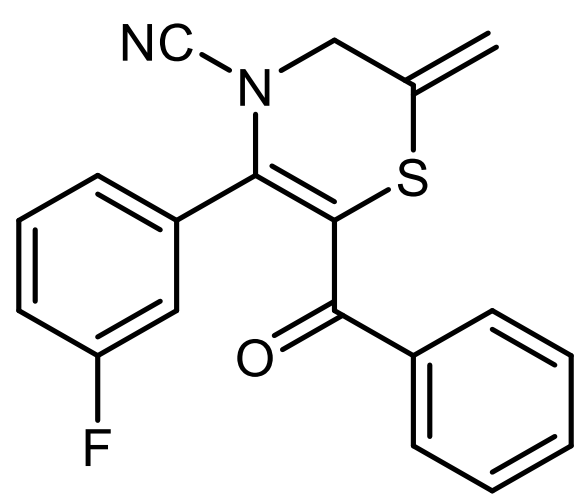

6-benzoyl-5-(3-fluorophenyl)-2-methylene-2,3-dihydro-4H-1,4-thiazine-4-carbonitrile(**3z**). General procedure C was followed and purification by flash column chromatography afforded **3z** as a yellow solid (PE: EA = 8:1). Yield: 463 mg, 69%; Mp: 140–143 ºC. ^1H NMR (400 MHz, $CDCl_3$): δ = 7.62–7.55 (m, 2H), 7.38–7.32 (m, 1H),

7.22 (t, J = 7.8 Hz, 2H), 7.15–7.09 (m, 1H), 7.05–7.02 (m, 1H), 6.98–6.93 (m, 1H), 6.91–6.85 (m, 1H), 5.58 (d, J = 1.2 Hz, 1H), 5.41 (d, J = 1.2 Hz, 1H), 4.37 (s, 2H). ^{13}C NMR (101 MHz, $CDCl_3$): δ = 192.4, 162.3 (d, $J_{C\text{-}F}$ = 247 Hz), 136.3, 134.0, 133.1, 132.3, 130.4 (d, $J_{C\text{-}F}$ = 8 Hz), 129.3, 128.2, 125.8 (d, $J_{C\text{-}F}$ = 3 Hz), 118.7, 117.8 (d, $J_{C\text{-}F}$ = 21 Hz), 116.9, 116.7, 113.5, 112.4, 54.7. ^{19}F NMR (376 MHz, $CDCl_3$): δ = -111.46 (s, F). HRMS (ESI): m/z $[M + H]^+$ calcd for: $C_{19}H_{14}N_2OSF$:337.0811; found: 337.0815.

Synthesis of 3aa

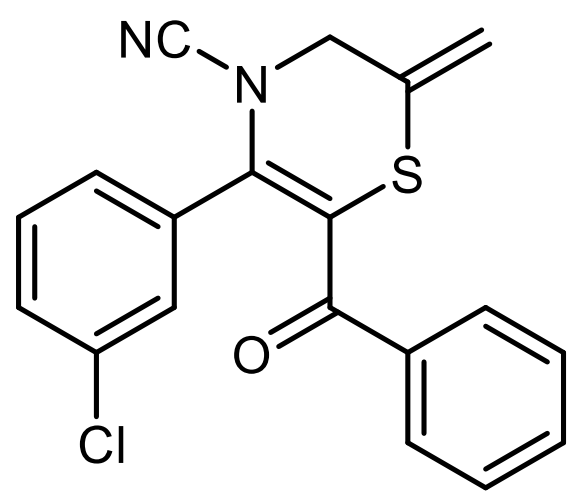

6-benzoyl-5-(3-chlorophenyl)-2-methylene-2,3-dihydro-4H-1,4-thiazine-4-carbonitrile(**3aa**). General procedure C was followed and purification by flash column chromatography afforded **3aa** as a yellow solid (PE: EA = 8:1). Yield: 457 mg, 65%; mp: 166–169 ºC. ^{1}H NMR (400 MHz, $CDCl_3$): δ = 7.60–7.52 (m, 2H), 7.38–7.31 (m, 1H), 7.25–7.18 (m, 3H), 7.16–7.11 (m, 2H), 7.10–7.04 (m, 1H), 5.58 (d, J = 1.2 Hz, 1H), 5.41 (d, J = 1.4 Hz, 1H), 4.37 (s, 2H). ^{13}C NMR (101 MHz, $CDCl_3$): δ = 192.5, 138.5, 136.4, 134.6, 133.7, 133.0, 132.3, 130.7, 130.0, 129.9, 129.1, 128.1, 128.0, 118.9, 113.5, 112.3, 54.7. HRMS (ESI): m/z $[M + H]^+$ calcd for: $C_{19}H_{14}N_2OSCl$: 353.0515; found: 353.0515.

Synthesis of 3ab

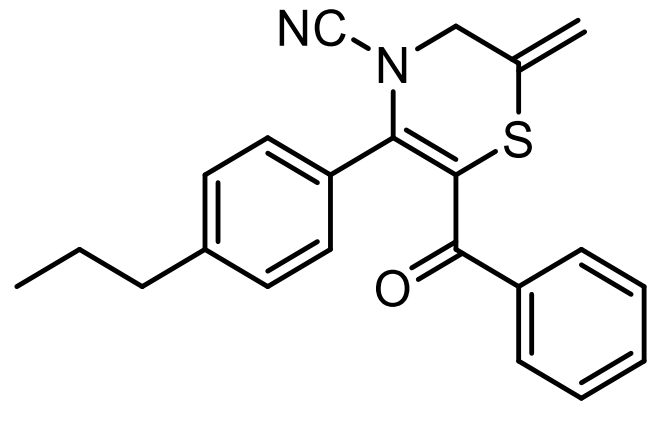

6-benzoyl-2-methylene-5-(4-propylphenyl)-2,3-dihydro-4H-1,4-thiazine-4-carbonitrile (**3ab**). General procedure C was followed and purification by flash column

chromatography afforded **3ab** as a yellow solid (PE: EA = 8:1 to 6:1). Yield: 396 mg, 55%; mp: 122–124 ºC. ^{1}H NMR (400 MHz, $CDCl_3$): δ = 7.54–7.47 (m, 2H), 7.25–7.21 (m, 1H), 7.15–7.70 (m, 4H), 6.91 (d, J = 8.1 Hz, 2H), 5.54 (d, J = 1.2 Hz, 1H), 5.36 (d, J = 1.2 Hz, 1H), 4.38 (s, 2H), 2.40 (t, J = 7.5 Hz, 2H), 1.44 (q, J = 7.4 Hz, 2H), 0.74 (t, J = 7.3 Hz, 3H). ^{13}C NMR (101 MHz, $CDCl_3$): δ = 193.1, 146.0, 141.7, 136.9, 133.2, 132.3, 130.1, 129.4, 129.2, 128.7, 127.8, 116.6, 112.7, 112.6, 55.0, 37.6, 23.9, 13.4. HRMS (ESI): m/z $[M + H]^+$ calcd for: $C_{22}H_{21}N_2OS$: 361.1375; found: 361.1376.

Synthesis of 3ac

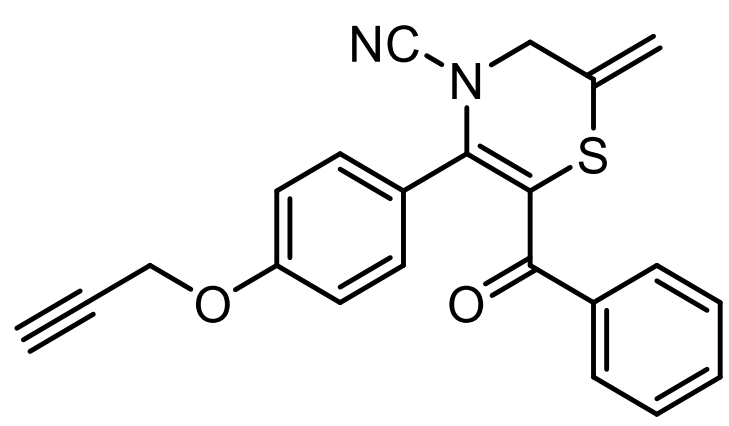

6-benzoyl-2-methylene-5-(4-(prop-2-yn-1-yloxy)phenyl)-2,3-dihydro-4H-1,4-thiazine-4-carbonitrile (**3ac**). General procedure C was followed and purification by flash column chromatography afforded **3ac** as a yellow solid (PE: EA = 8:1 to 6:1). Yield: 424 mg, 57%; mp: 122–125 ºC. ^{1}H NMR (400 MHz, $CDCl_3$): δ = 7.58–7.47 (m, 2H), 7.32–7.26 (m, 1H), 7.20–7.12 (m, 4H), 6.75–6.65 (m, 2H), 5.53 (d, J = 0.6 Hz, 1H), 5.36 (s, 1H), 4.55 (d, J = 2.4 Hz, 2H), 4.37 (s, 2H), 2.48 (s, 1H). ^{13}C NMR (101 MHz, $CDCl_3$) : δ = 192.9, 159.4, 141.1, 136.8, 133.2, 132.6, 131.6, 129.2, 128.0, 125.3, 116.4, 115.0, 112.7, 112.6, 77.7, 75.9, 55.7, 55.0. HRMS (ESI): m/z $[M + H]^+$ calcd for: $C_{22}H_{17}N_2O_2S$: 373.1011; found: 373.1006.

Synthesis of 3ad

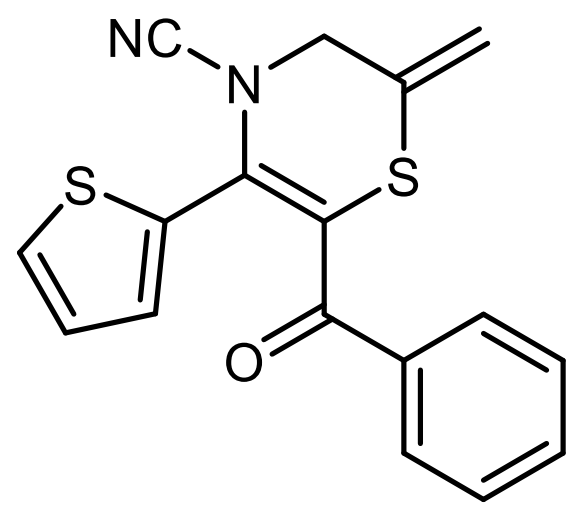

6-benzoyl-2-methylene-5-(thiophen-2-yl)-2,3-dihydro-4H-1,4-thiazine-4-carbonitrile

(**3ad**). General procedure C was followed and purification by flash column chromatography afforded **3ad** as a yellow solid (PE: EA = 2:1). Yield: 317 mg, 49%; mp: 158–161 °C. ^{1}H NMR (400 MHz, $CDCl_3$): δ = 7.66–7.60 (m, 2H), 7.40–7.34 (m, 1H), 7.28 (dd, J = 5.1, 1.2 Hz, 1H), 7.25–7.20 (m, 2H), 6.99 (dd, J = 3.7, 1.2 Hz, 1H), 6.73 (dd, J = 5.1, 3.7 Hz, 1H), 5.56 (d, J = 1.1 Hz, 1H), 5.40 (d, J = 0.8 Hz, 1H), 4.36 (s, 2H). ^{13}C NMR (101 MHz, $CDCl_3$): δ = 192.3, 136.2, 133.6, 133.3, 132.9, 132.8, 130.9, 129.1, 128.2, 127.6, 118.3, 113.3, 112.4, 55.0. HRMS (ESI): m/z $[M + H]^+$ calcd for: $C_{17}H_{13}N_2OS_2$: 325.0469; found:325.0470.

Synthesis of 3ae

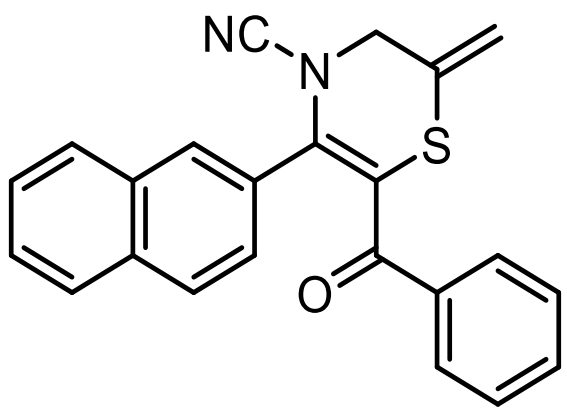

6-benzoyl-2-methylene-5-(naphthalen-2-yl)-2,3-dihydro-4H-1,4-thiazine-4-carbonitrile (**3ae**). General procedure C was followed and purification by flash column chromatography afforded **3ae** as a yellow solid (PE: EA = 8:1 to 6:1). Yield: 368 mg, 50%; mp: 164–167 °C. ^{1}H NMR (400 MHz, $CDCl_3$): δ = 7.60 (d, J = 7.3 Hz, 2H), 7.50–7.32 (m, 7H), 7.22 (t, J = 8 Hz, 3H), 5.59 (s, 1H), 5.43 (s, 1H), 4.39 (s, 2H). ^{13}C NMR (101 MHz, $CDCl_3$): δ = 192.2, 137.7, 136.2, 135.4, 133.3, 132.4, 132.1, 131.9, 130.2, 130.1, 129.4, 128.4, 128.3, 125.7, 125.6, 124.7, 121.9, 119.7, 113.8, 112.3, 54.6. HRMS (ESI): m/z $[M + H]^+$ calcd for: $C_{23}H_{17}N_2OS$: 369.1062; found: 369.1058.

Synthesis of 3af

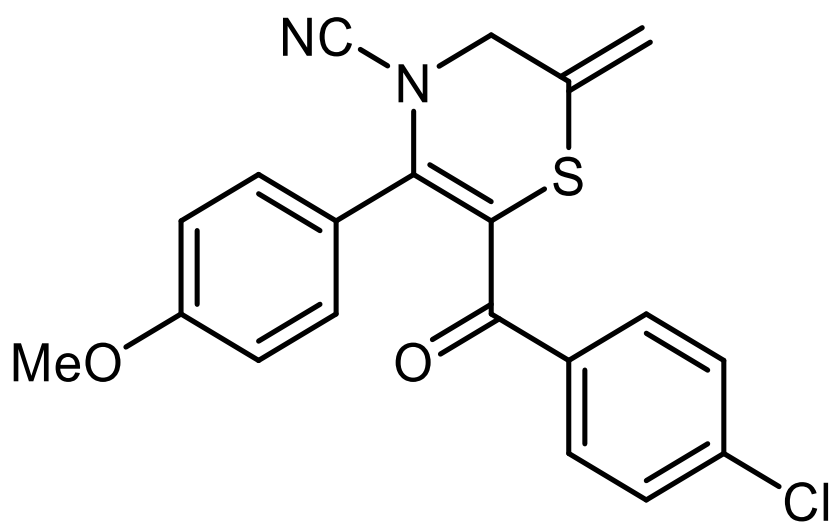

6-(4-chlorobenzoyl)-5-(4-methoxyphenyl)-2-methylene-2,3-dihydro-4H-1,4-thiazine-4-carbonitrile (**3af**). General procedure C was followed and purification by flash column chromatography afforded **3af** as a yellow solid (PE: EA = 9:1). Yield: 382 mg, 50%; mp: 118–120 °C. ^{1}H NMR (400 MHz, $CDCl_3$): δ = 7.49–7.44 (m, 2H), 7.17–7.10 (m, 4H), 6.68–6.64 (m, 2H), 5.53 (s, 1H), 5.36 (s, 1H), 4.37 (s, 2H), 3.70 (s, 3H). ^{13}C NMR (101 MHz, $CDCl_3$) : δ = 191.7, 161.8, 141.9, 138.7, 135.2, 133.1, 131.7, 130.5, 128.3, 124.2, 115.5, 114.3, 112.7, 112.6, 55.3, 55.1. HRMS (ESI): m/z $[M + H]^+$ calcd for: $C_{20}H_{16}N_2O_2SCl$: 383.0621; found: 383.0625.

Synthesis of 3ag

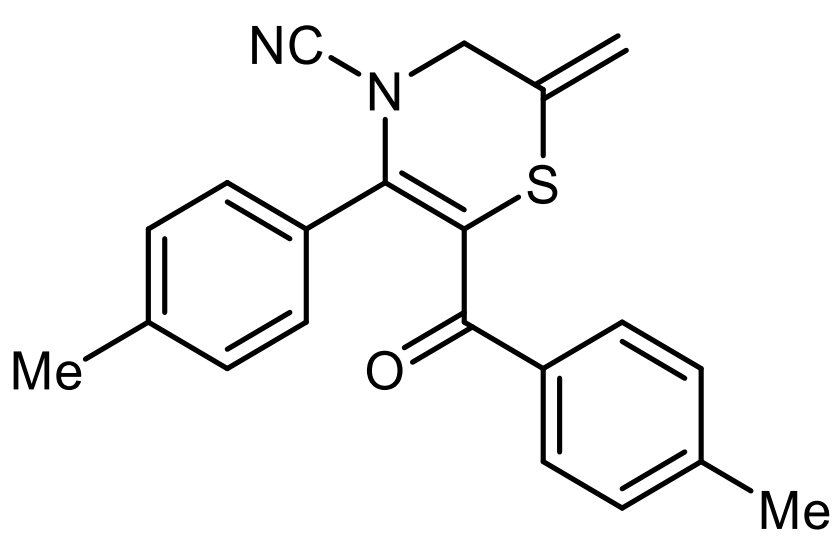

6-(4-methylbenzoyl)-2-methylene-5-(p-tolyl)-2,3-dihydro-4H-1,4-thiazine-4-carbonitrile (**3ag**). General procedure C was followed and purification by flash column chromatography afforded **3ag** as a yellow solid (PE: EA = 8:1). Yield: 422 mg, 61%; mp: 145–146 °C. ^{1}H NMR (400 MHz, $CDCl_3$): δ = 7.52 (d, J = 8.3 Hz, 2H), 7.16–7.11 (m, 2H), 6.98 (dd, J = 14.9, 8.0 Hz, 4H), 5.51 (d, J = 1.1 Hz, 1H), 5.35 (d, J = 1.1 Hz, 1H), 4.36 (s, 2H), 2.27 (s, 3H), 2.20 (s, 3H). ^{13}C NMR (101 MHz, $CDCl_3$): δ = 192.3, 143.8, 141.1, 139.9, 133.8, 133.1, 129.7, 129.6, 129.4, 129.3, 128.8, 116.7, 112.9, 112.5, 54.8, 21.6, 21.3. HRMS (ESI): m/z $[M + H]^+$ calcd for: $C_{21}H_{19}N_2OS$: 347.1218; found: 347.1222.

Synthesis of 3ah

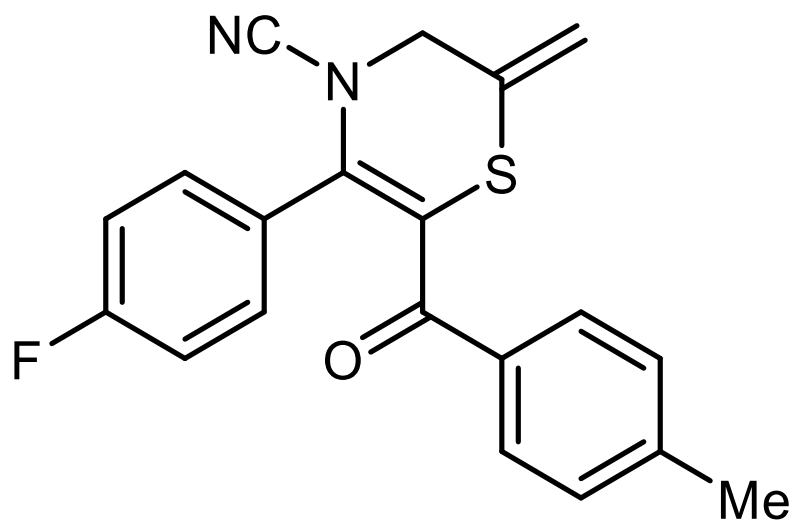

5-(4-fluorophenyl)-6-(4-methylbenzoyl)-2-methylene-2,3-dihydro-4H-1,4-thiazine-4-carbonitrile (**3ah**). General procedure C was followed and purification by flash column chromatography afforded **3ah** as a yellow solid (PE: EA = 9:1). Yield: 469 mg, 67%; mp: 85–88 °C. ^{1}H NMR (400 MHz, $CDCl_3$): δ = 7.58–7.45 (m, 2H), 7.28–7.21 (m, 2H), 7.01 (d, J = 7.9 Hz, 2H), 6.90–6.80 (m, 2H), 5.53(s, 1H), 5.37 (s, 1H), 4.36 (s, 2H), 2.28 (s, 3H).^{13}C NMR (101 MHz, $CDCl_3$): δ = 192.1, 163.7 (d, $J_{C\text{-}F}$ = 251 Hz), 144.2, 138.2, 133.6, 132.6, 131.8 (d, $J_{C\text{-}F}$ = 9 Hz), 129.5, 128.9, 128.3 (d, $J_{C\text{-}F}$ = 2 Hz), 117.7, 115.9 (d, $J_{C\text{-}F}$ = 22 Hz), 113.0, 112.6, 54.7, 21.5. ^{19}F NMR (376 MHz, $CDCl_3$): δ = -108.47 (s, F). HRMS (ESI): m/z $[M + H]^+$ calcd for: $C_{20}H_{16}FN_2OS$: 351.0967; found: 351.0969.

Synthesis of 3ai

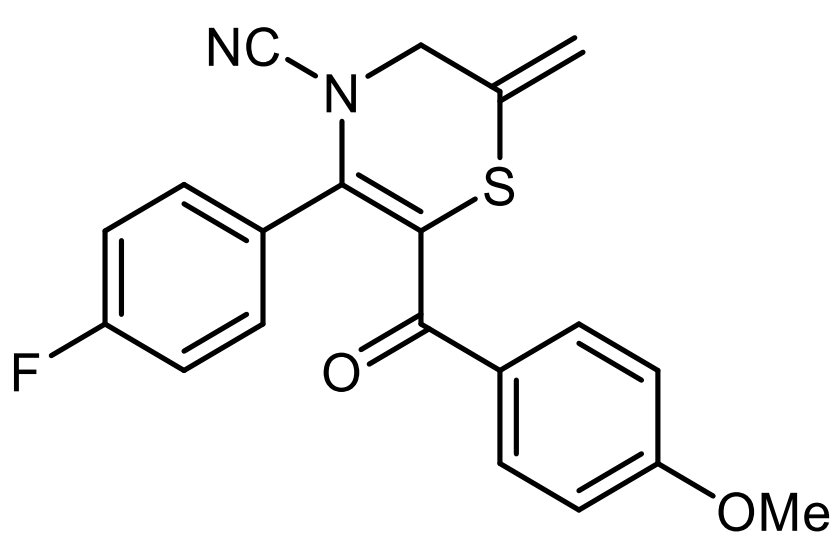

5-(4-fluorophenyl)-6-(4-methoxybenzoyl)-2-methylene-2,3-dihydro-4H-1,4-thiazine-4-carbonitrile (**3ai**). General procedure C was followed and purification by flash column chromatography afforded **3ai** as a yellow solid (PE: EA = 8:1). Yield: 505 mg, 69%; mp: 117–120 °C. ^{1}H NMR (400 MHz, $CDCl_3$): δ = 7.68–7.59 (m, 2H), 7.29–7.23 (m, 2H), 6.92–6.84 (m, 2H), 6.74–6.68 (m, 2H), 5.54 (d, J = 1.2 Hz, 1H), 5.38 (d, J = 1.2 Hz, 1H), 4.36 (s, 2H), 3.79 (s, 3H).^{13}C NMR (101 MHz, $CDCl_3$) : δ = 190.8, 163.8 (d,

J_{C-F} = 251 Hz), 163.7, 137.1, 132.5, 131.9, 131.7 (d, J_{C-F} = 9 Hz), 128.8, 128.3 (d, J_{C-F} = 3 Hz), 117.7, 116.0 (d, J_{C-F} = 22 Hz), 113.6, 113.1, 112.7, 55.5, 54.7. ^{19}F NMR (376 MHz, $CDCl_3$): δ = -108.43 (s, F). HRMS (ESI): m/z $[M + H]^+$ calcd for: $C_{20}H_{16}N_2O_2FS$: 367.0917; found: 367.0922.

Synthesis of 3aj

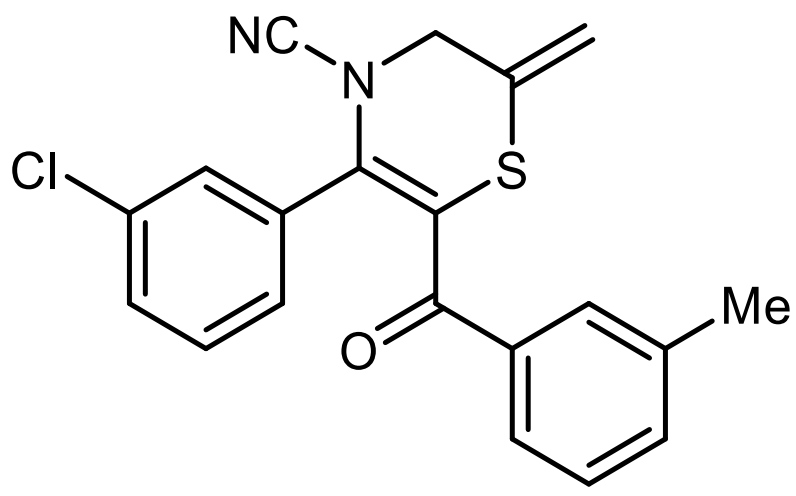

5-(3-chlorophenyl)-6-(3-methylbenzoyl)-2-methylene-2,3-dihydro-4H-1,4-thiazine-4-carbonitrile (**3aj**). General procedure C was followed and purification by flash column chromatography afforded **3aj** as a yellow solid (PE: EA = 7:1). Yield: 519 mg, 71%; mp: 104–105 °C. ^{1}H NMR (400 MHz, $CDCl_3$): δ = 7.38–7.33 (m, 2H), 7.22 (t, J = 1.9 Hz, 1H), 7.17–7.05 (m, 5H), 5.57 (d, J = 1.2 Hz, 1H), 5.40 (d, J = 1.3 Hz, 1H), 4.37 (s, 2H), 2.26 (s, 3H). ^{13}C NMR (101 MHz, $CDCl_3$): δ = 192.5, 138.6, 138.0, 136.3, 134.6, 133.8, 132.4, 130.7, 129.9, 129.8, 129.7, 128.0, 127.9, 126.5, 119.1, 113.4, 112.4, 54.7, 21.1. HRMS (ESI): m/z $[M + H]^+$ calcd for: $C_{20}H_{16}N_2OSCl$: 367.0672; found: 367.0676.

Synthesis of 3ak

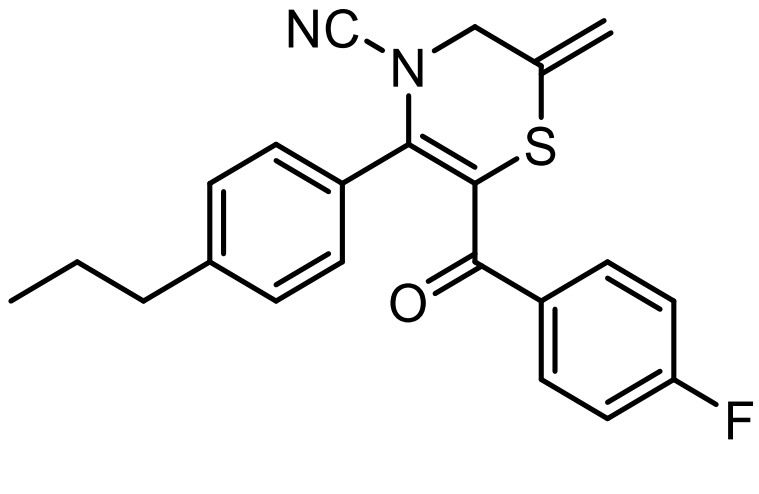

5-(4-butylphenyl)-6-(4-fluorobenzoyl)-2-methylene-2,3-dihydro-4H-1,4-thiazine-4-carbonitrile (**3ak**). General procedure C was followed and purification by flash column chromatography afforded **3ak** as a yellow solid (PE: EA = 9:1). Yield: 386 mg, 51%; mp: 114–116 °C. ^{1}H NMR (400 MHz, $CDCl_3$): δ = 7.58–7.48 (m, 2H), 7.12 (d, J = 8.2 Hz, 2H), 6.94 (d, J = 8.1 Hz, 2H), 6.79 (t, J = 8.6 Hz, 2H), 5.55 (s, 1H), 5.38 (s, 1H),

4.38 (s, 2H), 2.43 (t, J = 7.4 Hz, 2H), 1.46 (q, J = 7.4 Hz, 2H), 0.76 (t, J = 7.3 Hz, 3H). ^{13}C NMR (101 MHz, $CDCl_3$): δ = 191.5, 164.9 (d, $J_{C\text{-}F}$ = 253 Hz), 146.4, 141.8, 133.2 (d, $J_{C\text{-}F}$ = 3 Hz), 133.0, 131.7 (d, $J_{C\text{-}F}$ = 9 Hz), 130.1, 129.3, 128.9, 116.4, 115.0 (d, $J_{C\text{-}F}$ = 22 Hz), 112.8, 112.6, 55.0, 37.6, 24.0, 13.3. ^{19}F NMR (376 MHz, $CDCl_3$): δ = -105.45 (s, F). HRMS (ESI): m/z [M + H]$^+$ calcd for: $C_{22}H_{20}FN_2OS$:379.1280; found: 379.1279.

Synthesis of 3al

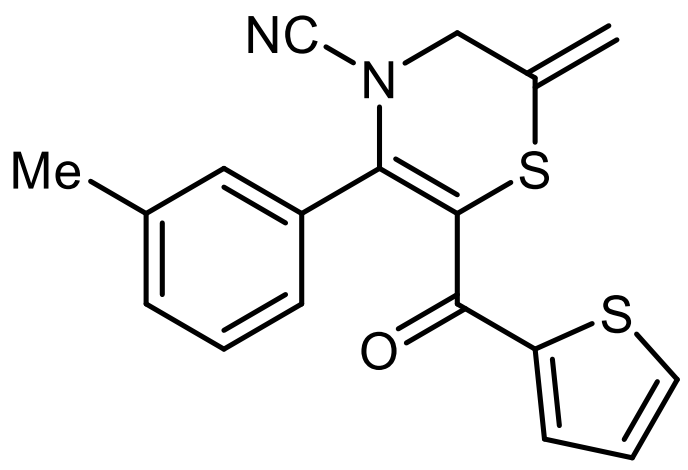

2-methylene-6-(thiophene-2-carbonyl)-5-(m-tolyl)-2,3-dihydro-4H-1,4-thiazine-4-carbonitrile (**3al**). General procedure C was followed and purification by flash column chromatography afforded **3al** as a yellow solid (PE: EA = 8:1). Yield: 452 mg, 67%; mp: 143–146 ºC. ^{1}H NMR (400 MHz, $CDCl_3$): δ = 7.46 (dd, J = 4.9, 1.2 Hz, 1H), 7.36 (dd, J = 3.8, 1.2 Hz, 1H), 7.17–7.04 (m, 4H), 6.82 (dd, J = 4.9, 3.8 Hz, 1H), 5.53 (d, J = 1.2 Hz, 1H), 5.37 (q, J = 1.1 Hz, 1H), 4.34 (s, 2H), 2.22 (s, 3H). ^{13}C NMR (101 MHz, $CDCl_3$): δ = 184.4, 142.4, 139.3, 138.6, 134.7, 134.3, 132.7, 132.0, 131.6, 130.4, 128.7, 127.6, 126.7, 117.3, 112.8, 112.7, 54.8, 21.1. HRMS (ESI): m/z [M + H]$^+$ calcd for: $C_{18}H_{15}N_2OS_2$:339.0626; found: 339.0631.

Synthesis of 3am

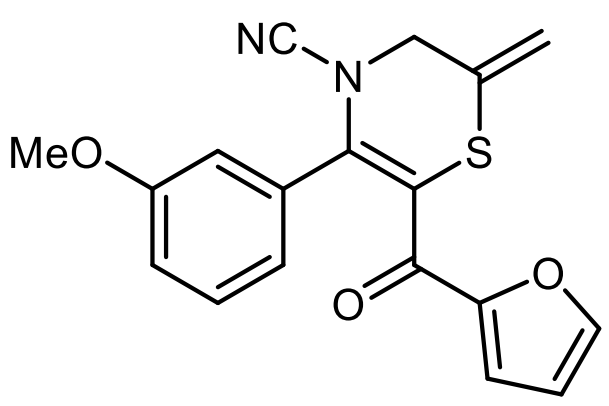

6-(furan-2-carbonyl)-5-(3-methoxyphenyl)-2-methylene-2,3-dihydro-4H-1,4-thiazine-4-carbonitrile (**3am**). General procedure C was followed and purification by flash column chromatography afforded **3am** as a yellow solid (PE: EA = 5:1). Yield: 459 mg, 68%; mp: 155–157 ºC. ^{1}H NMR (400 MHz, $CDCl_3$): δ = 7.28–7.26 (m, 1H), 7.16 (t, J

= 7.9 Hz, 1H), 6.96–6.89 (m, 2H), 6.85–6.79 (m, 2H), 6.26 (dd, *J* = 3.6, 1.7 Hz, 1H), 5.54 (d, *J* = 1.2 Hz, 1H), 5.37 (d, *J* = 1.1 Hz, 1H), 4.33 (s, 2H), 3.72 (s, 3H). ^{13}C NMR (101 MHz, $CDCl_3$) : δ = 179.4, 159.5, 151.1, 146.8, 140.5, 133.4, 132.8, 129.8, 122.1, 119.6, 117.1, 116.7, 114.5, 112.9, 112.6, 112.3, 55.4, 54.8. HRMS (ESI): m/z $[M + H]^+$ calcd for: $C_{18}H_{15}N_2O_3S$: 339.0803; found: 339.0808.

Synthesis of 3an

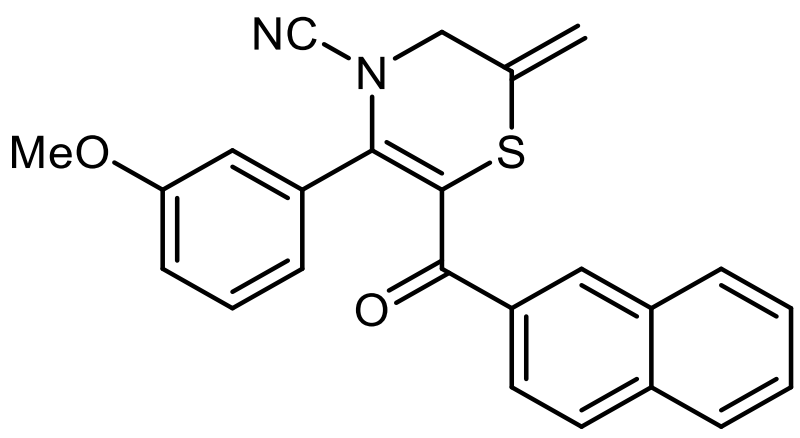

6-(2-naphthoyl)-5-(3-methoxyphenyl)-2-methylene-2,3-dihydro-4H-1,4-thiazine-4-carbonitrile (**3an**). General procedure C was followed and purification by flash column chromatography afforded **3an** as a yellow solid (PE: EA = 8:1). Yield: 493 mg, 62%; mp: 128–131 ºC. ^{1}H NMR (400 MHz, $CDCl_3$): δ = 8.10 (d, *J* = 1.6 Hz, 1H), 7.82–7.73 (m, 2H), 7.69–7.63 (m, 2H), 7.55–7.46 (m, 2H), 6.98–6.85 (m, 2H), 6.75 (dd, *J* = 2.5, 1.7 Hz, 1H), 6.56–6.52 (m, 1H), 5.58 (d, *J* = 1.2 Hz, 1H), 5.41 (d, *J* = 1.2 Hz, 1H), 4.44 (s, 2H), 3.56 (s, 3H). ^{13}C NMR (101 MHz, $CDCl_3$) : δ = 192.5, 159.3, 140.2, 135.2, 133.8, 133.4, 132.7, 131.8, 131.3, 129.7, 129.2, 128.5, 128.1, 127.7, 126.7, 124.5, 122.2, 117.5, 116.9, 115.0, 113.1, 112.7, 55.2, 54.9. HRMS (ESI): m/z $[M + H]^+$ calcd for: $C_{24}H_{19}N_2O_2S$: 399.1167; found: 399.1169.

(f) Biological Assessment of 3, 4-Dihydro-2H-1, 4-thiazines

1. Cell culture. NCI-H820 cells were cultured in 1640 medium supplement with 10% fetal bovine serum (FBS). 3T3 cells were cultured in DMEM medium supplement with 10% fetal bovine serum. And the cells were incubated in 37 °C with 5% CO_2.

2. MTT assay. Cells were collected, counted and seeded into 96 well plate with 2000 cells each well. After the cell adherence, compounds with different concentrations were added and incubated for 72 hours. Then 20 μL MTT solution (5 mg/mL) was added into each well and incubated for about 4 hours. After discarding the supernatant, the precipitate was dissolved with DMSO and the absorption value of 570 nm was determined.

3. Lipid ROS assay. NCI-H820 cells were counted and seeded into 12 well plate with 80,000 cells each well. After the cell adherence, compounds with different concentrations were added and incubated for 48 hours. Then the cells were digested, collected, washed with PBS buffer and then incubated with BODIPY 581/591 C11 (5 μM) for 40 minutes at 37 °C. After that the cells were washed and analyzed with flow cytometry.

4. Western blot assay. NCI-H820 cells treated with compounds were collected and lysed using RIPA. The proteins were separated with SDS-PAGE and transferred to PVDF membranes. Then the membranes were blocked with 5% non-fat milk in PBST and incubated with specific primary antibodies overnight at 4 °C. After washing five times with PBST, the membranes were incubated with corresponding secondary antibodies for 2 hours at room temperature and developed with electrochemiluminescence.

5. Animal Assay. The animal experiment protocol was approved by the Experimental Animal Ethics Committee of Nankai University (approval No. 2025-SYDWLL-000523), and all procedures were performed in strict accordance with the guidelines

outlined in the Guide for the Care and Use of Laboratory Animals. The animals were housed in a specific-pathogen-free (SPF) environment. Patient-derived xenograft (PDX) models, established from human lung carcinoma tissues, were obtained from Beijing IDMO Co., Ltd. The human lung cancer tumor tissue excised from PDX model mice were cut into 5-8 mm^3 small pieces and subcutaneously inoculated on flank of the BAL B/c nude mice. After the average volume of the transplanted tumors reached approximately 25 mm^3 (approximately 7 days after inoculation), the tumor-bearing mice were divided randomly into solvent control group, **3b** treatment group (5 mg/kg) and **3c** treatment group (10 mg/kg), with five mice in each group. Compounds **3b** and **3c** dissolved in corn oil containing 5% DMSO were administered via intraperitoneal injection every three days for a total of six administrations, whereas the control cohort received an equivalent volume of vehicle alone. The tumor growth rate was monitored every two days by measuring both the long and short diameters using an electronic vernier caliper, with tumor volumes calculated according to the formula: volume = 1/2 × (long diameter) × (short diameter) ^{2}; concurrently, body weights were recorded to evaluate drug tolerability.

6. Acute toxicity assay. BAL B/c mice were randomly divided randomly into vehicle control group, **3b** treatment group and **3c** treatment group, with five mice in each group. Compounds **3b** and **3c** dissolved in corn oil containing 5% DMSO were administered via intraperitoneal injection at doses of 25 mg/kg. Then the behaviors, body and organ weights of mice were recorded and the levels of AST and CR was examined.

7. Target predication. The possible target of **3c** and **3b** was predicted in SwissTargetPrediction (https://swisstargetprediction.ch/), AmIActive (https://amiactive.ccen.ufpb.br/) and bSDTNBI (https://lmmd.ecust.edu.cn/netinfer/). The top 100 predicted targets were ranked by prediction score. Subsequently, the intersection of the predications from the four lists were selected.

8. Molecular Docking. Molecular docking between **3b**/**3c** and poly (ADP-ribose) polymerase 1 (PARP1) were performed using AutoDock software. The crystal structure

of PARP1 (PDB code: 7ONT) was retrieved from the RCSB Protein Data Bank, and all solvent molecules were removed. The three-dimensional structures of **3b** and **3c** were sketched using ChemDraw and subsequently optimized to their lowest-energy conformations. The ligand poses with the highest docking scores were selected for subsequent analysis.

9. Ethics Statement. The PDX animal were purchased from BEIJING IDMO Co., Ltd. The study was approved by the Ethics Committee of Nankai University (approval No. NKUIRB2024072) and conducted in accordance with the Declaration of Helsinki. The animal experiments were approved by Nankai University Ethical Committee (approval No. 2025-SYDWLL-000523) and conformed to the legal mandates and national guidelines for the care and maintenance of laboratory animals.

10. Statistical analysis. Statistical analysis was carried out with GraphPad Prism. Comparisons between two groups were analyzed using Student's t-test, and comparisons among multiple groups were analyzed using one-way ANOVA. Statistical significance was set at $P < 0.05$ and denoted as *$P < 0.05$, ** $P < 0.01$, *** $P < 0.001$, and **** $P < 0.001$.

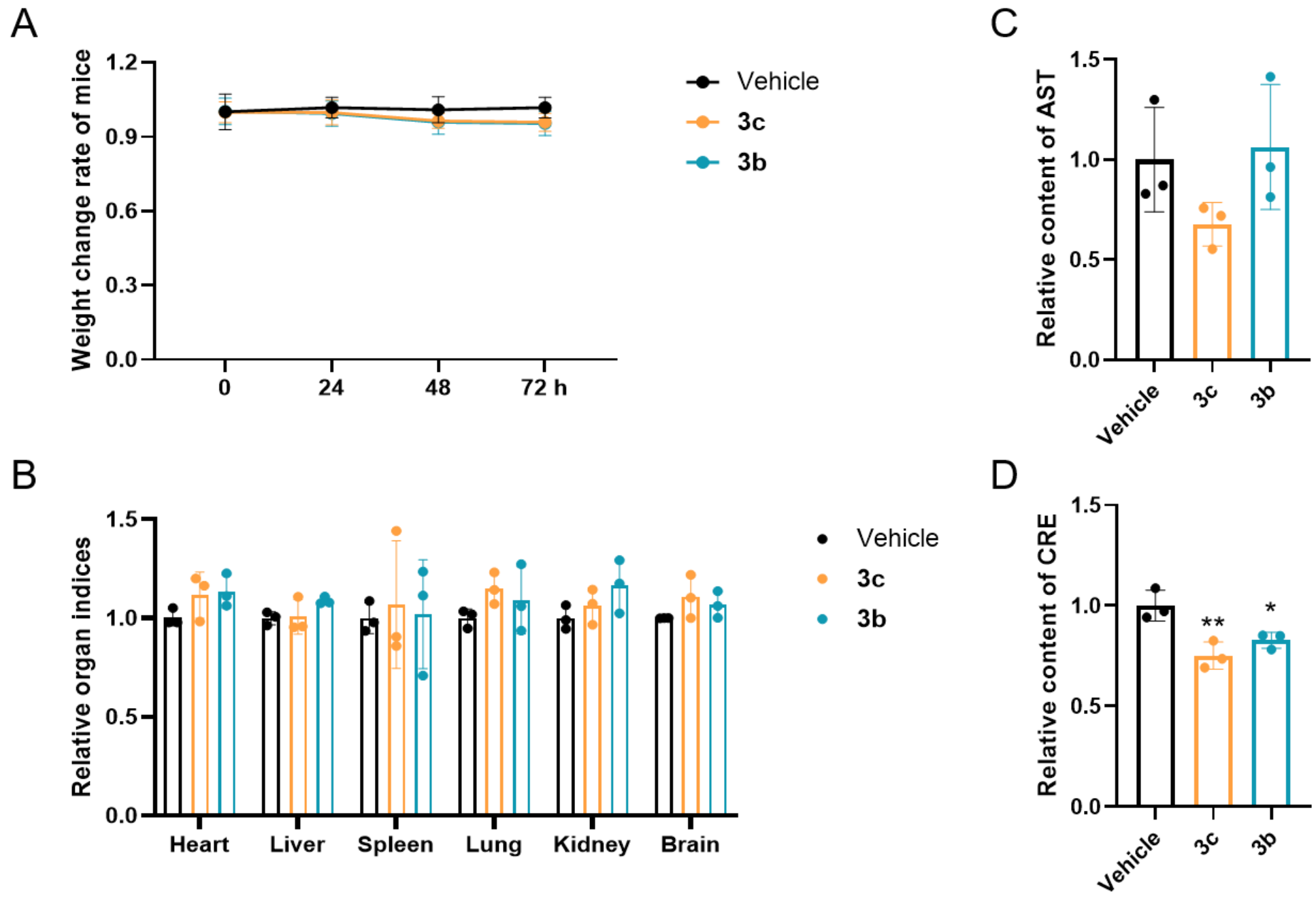

Figure S2. The toxicity assessment of compounds **3b** and **3c** *in vivo*. (A) The body weight change of BAL B/c mice after administrated with **3b** and **3c** at the dose of 25 mg/kg by intraperitoneal injections. (B) The organ indices (organ weight/body weight) were determined after administrated with **3b** and **3c** at the dose of 25 mg/kg by intraperitoneal injections for 72 hours. (C, D) Serum AST and CRE levels were measured after administrated with **3b** and **3c** at the dose of 25 mg/kg by intraperitoneal injections for 72 hours using commercial kits (Beyotime Biotechnology). All data are mean ± SD (n = 3 per group). **P* < 0.05; ***P* < 0.01.

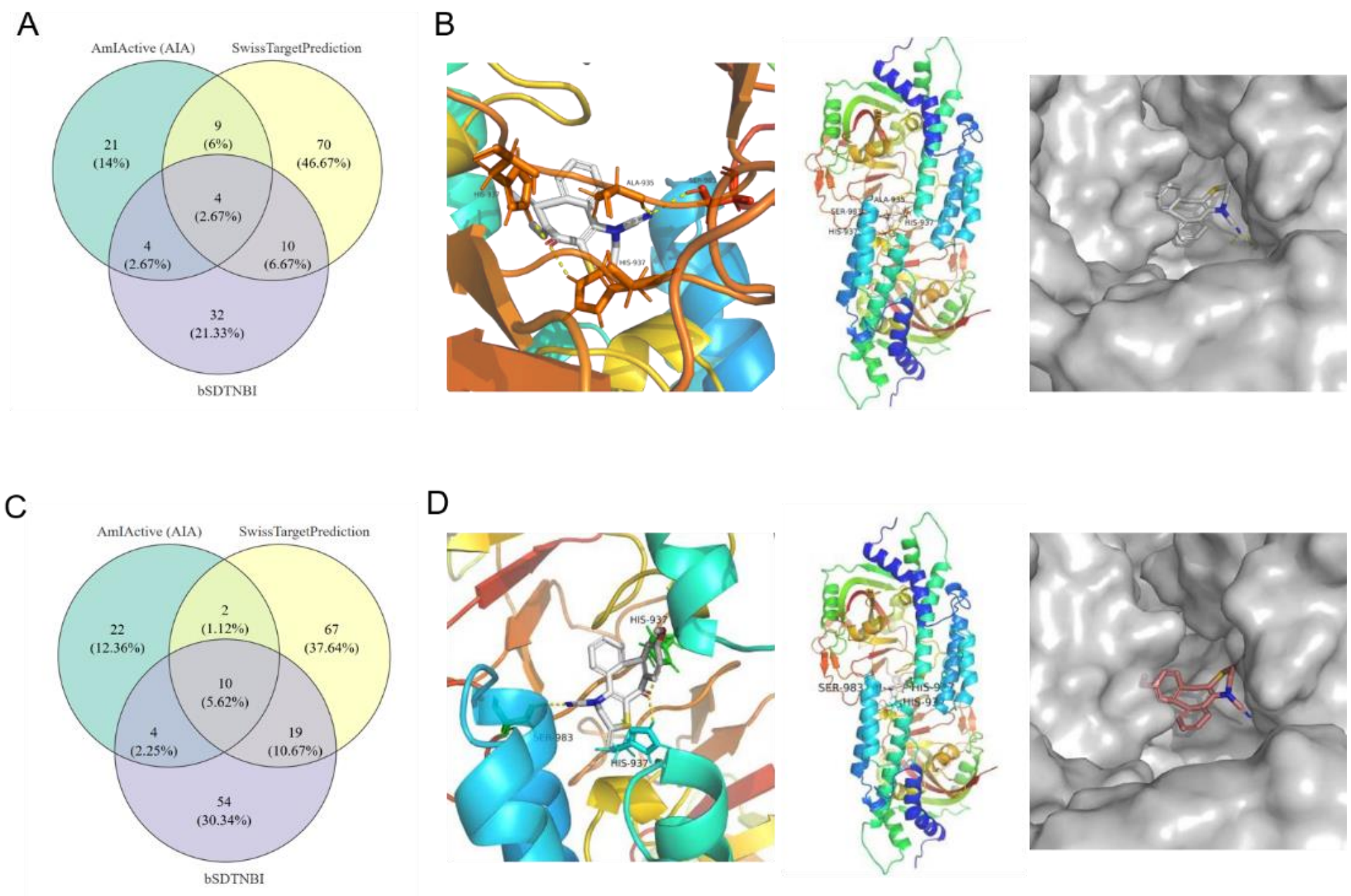

Figure S3. The target prediction and molecular docking of compounds **3b** and **3c**. (A, C) Venn diagrams showing predicted targets for **3c** (A) and **3b** (C) from SwissTargetPrediction, AmIActive and bSDTNBI. Numbers and percentages indicated target counts and proportions. (B) Molecular docking of **3c** with PARP1 (PDB: 7ONT). (D) Molecular docking of **3b** with PARP1 (PDB: 7ONT). Docking performed using AutoDock after ligand energy minimization.

Table S2. The selectivity of **3b**/**3c** against normal cells and cancer cells

Compounds	NCI-H820 (μM)	3T3 (μM)	Selectivity
**3b**	0.0068±0.0004	0.0285±0.0090	4.19
**3c**	0.014±0.0006	0.0653±0.0099	4.66

(g) X-Ray crystallographic studies

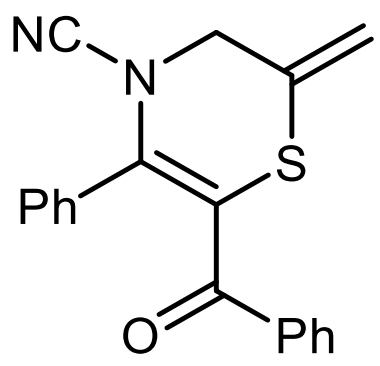

3a

Method of crystallization: A solution of **3a** in CH_2Cl_2 was left in the hood, and the solvent was allowed to evaporate slowly. The crystal structures have been deposited at the Cambridge Crystallographic Data Centre. CCDC 2497077 (**3a**), which contains the supplementary crystallographic data for this paper. These data can be obtained free of charge via the internet at https://www.ccdc.cam.ac.uk/structures/.

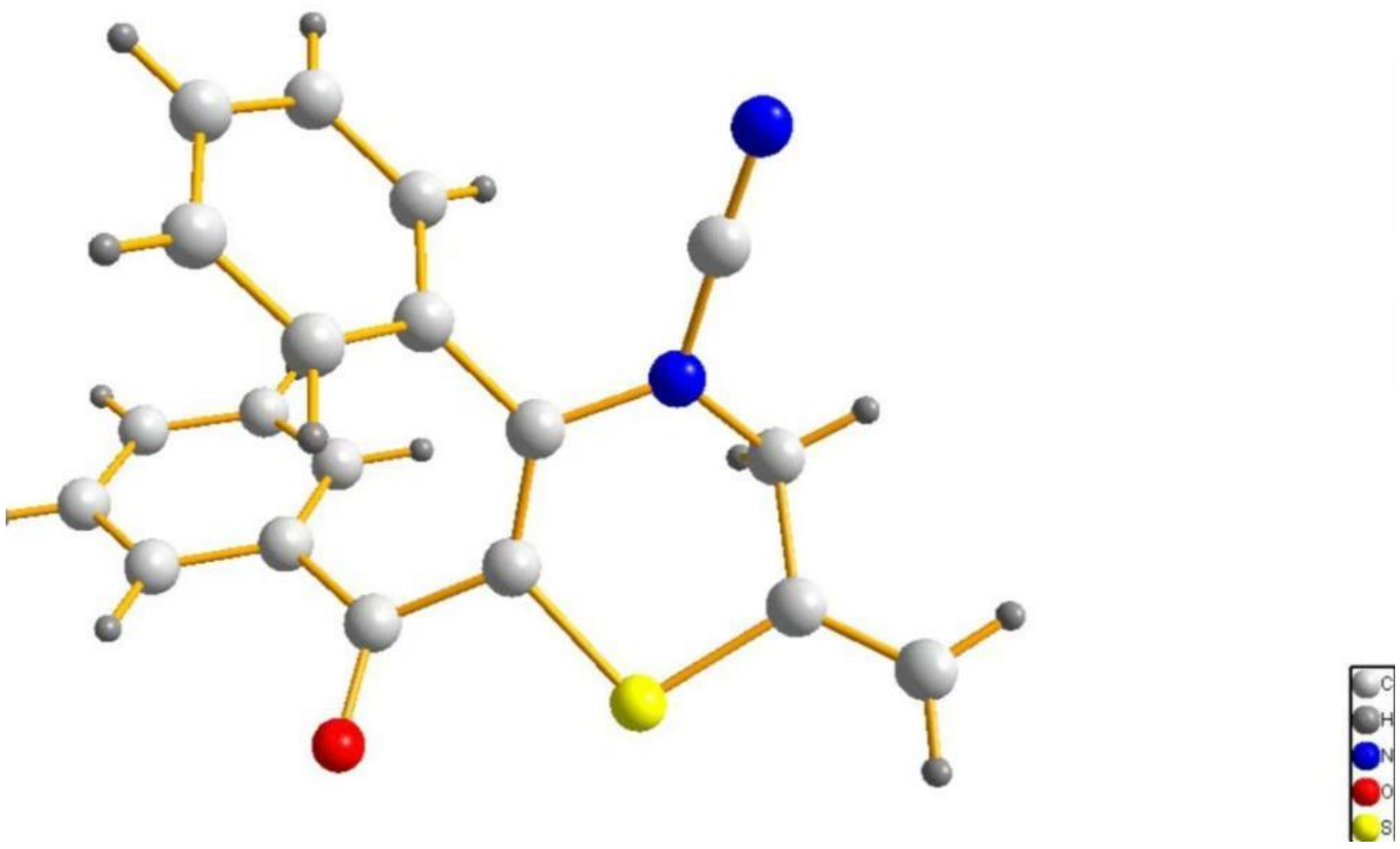

Figure S4. ORTEP X-ray structure of **3a**

Table S3. Crystal data and structure refinement for **3a**

Empirical formula	$C_{19}H_{14}N_2OS$
Formula weight	318.38
Temperature	296.15 K
Wavelength	0.71073 Å
Crystal system, space group	triclinic, P-1
Unit cell dimensions	a= 6.294 (4) Å alpha = 95.792 (15)°<br>b= 11.763 (6) Å beta = 104.526 (17)°<br>c= 11.928 (7) Å gamma = 105.242 (15)°
Volume	811.5(8) Å^3
Z, Calculated density	2, 1.303 g/cm^3
Absorption coefficient	0.205 mm^{-1}

F(000)	332.0
Crystal size	0.22×0.2×0.18 mm^3
Theta range for data collection	5.554 to 50.05°
Index ranges	-7≤h≤7, -13≤k≤13, -13≤l≤14
Reflections collected	10321
Independent reflections	2807 [R_{int} = 0.0867, R_{sigma} = 0.1078]
Completeness to theta = 25.025°	0.975 %
Absorption correction	multiscan
Refinement method	Full-matrix least-squares on F^2
Data / restraints / parameters	2807/0/209
Goodness-of-fit on F^2	0.992
Final R indices [I>2sigma(I)]	R1 = 0.0633, wR_2 = 0.1474
R indices (all data)	R1 = 0.1695, wR_2 = 0.2065
Largest diff. peak and hole	0.23 and -0.31 e. $Å^{-3}$

(h) Details of Calculation and Discussion

All of the DFT calculations were performed with the Gaussian 09 program package.[15] The geometry optimization of all the minima involved were performed at the M06-2X level of theory[16] with def2-SVP basis set[17] for all atoms. The structures of the reactants, intermediates, transition states, and products were fully optimized without any restriction. The vibrational frequencies were computed at the same level to check whether each optimized structure is an energy minimum or a transition state and to evaluate its zero-point vibrational energy (ZPVE) and thermal corrections at 298 K. IRC calculations[18] were used to confirm that the transition states found from the optimization calculations connect the related reactants and products. Single-point solvent calculations were performed with def2-TZVP basis set[17] at the optimized geometries for all the intermediates and transition states. Solvation effects were taken into account by applying the SMD[19] solvation model with DMF solvent in both geometry optimization and single-point energy calculations. The reported energies are Gibbs free energies in DMF solution (ΔG_{sol}). The 3D structures were presented using the CYLview visualization software.[20]

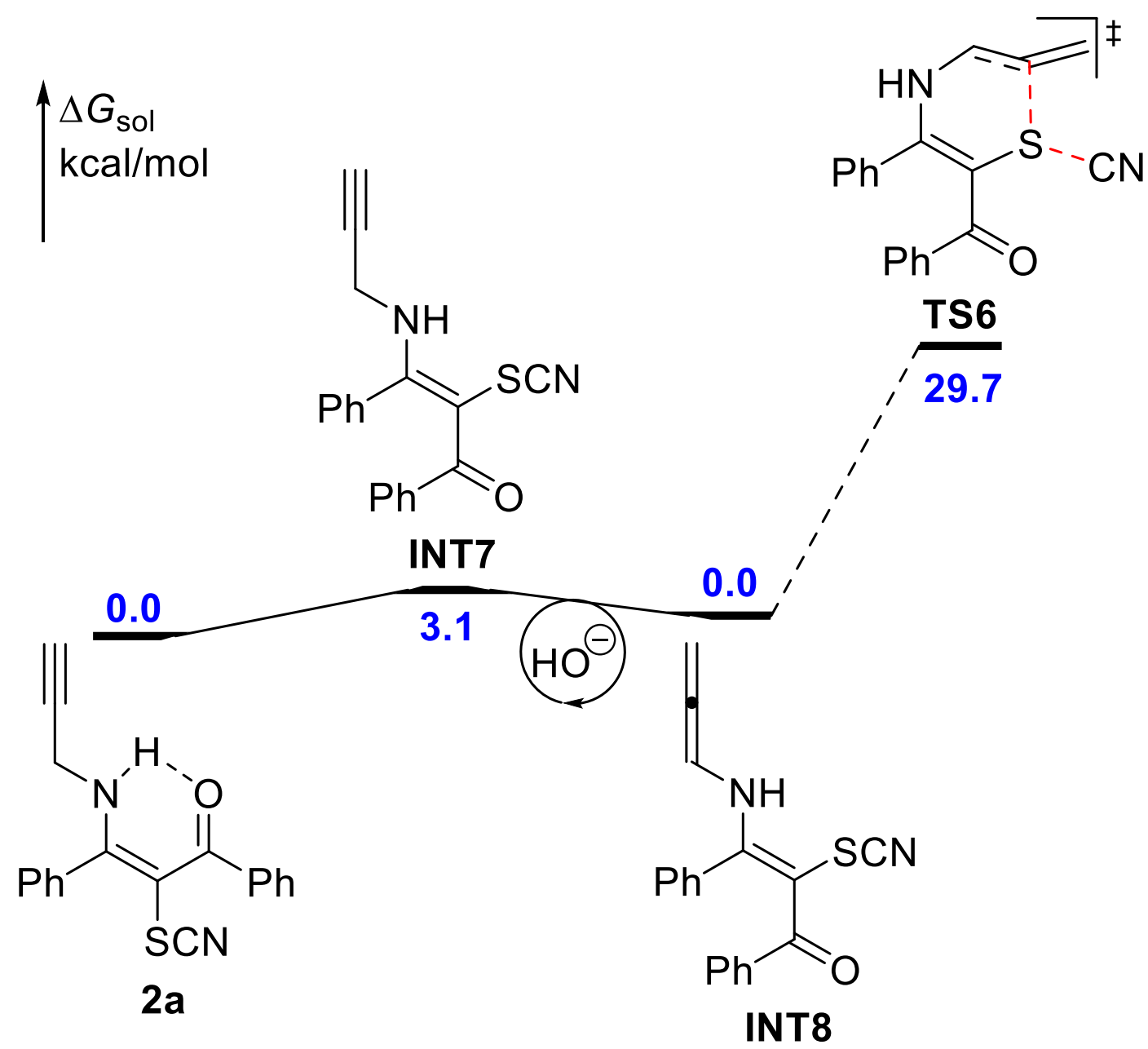

Figure S5 Calculated free-energy profiles of the direct cyclization pathway of **2a** at the M06-2X/def2-TZVP/SMD (SMD, DMF) // M06-2X / def2-SVP/(SMD, DMF) level of theory.

The computational results indicate that in the pathway involving cyclization prior to cyano migration (Figure S5), the cyclization transition state **TS6** is relatively high in energy and is also accompanied by cyano departure. Thus, this process is kinetically disfavored and is therefore ruled out.

DFT-Computed Energies of All Stationary Points

Table S4. Sum of electronic and thermal free energies (*G*, in Hartree), thermal correction to Gibbs free energy (*CGFE*, in Hartree), electronic energy in DMF (E_{sol}, in Hartree), and free energies in DMF (G_{sol}, in Hartree). For transition state structures, one imaginary frequency (Ifreq) was observed and given below. For all minimum structures, no imaginary frequency was observed.

Structure	*G*	*CGFE*	E_{sol}	G_{sol}	Ifreq
**2a**	-1313.873236	0.229729	-1315.256922	-1315.027193	
**HO$^-$**	-75.753618	-0.008076	-75.89156018	-75.89963618	
**INT1**	-1389.715279	0.239018	-1391.213107	-1390.974089	
**INT2**	-1313.38848	0.212468	-1314.763618	-1314.55115	
**INT2’**	-1313.378578	0.212945	-1314.747555	-1314.53461	
**H_2O**	-76.326898	0.003671	-76.43407288	-76.43040188	
**TS1**	-1313.378302	0.216166	-1314.758911	-1314.542745	-28.5147
**INT3**	-1313.390747	0.216719	-1314.769584	-1314.552865	
**TS2**	-1313.380309	0.219167	-1314.758782	-1314.539615	-307.134
**INT4**	-1313.394063	0.222086	-1314.774455	-1314.552369	
**TS3**	-1313.3937	0.220399	-1314.770025	-1314.549626	-91.8783
**INT5**	-1313.417252	0.217276	-1314.789587	-1314.572311	
**TS4**	-1313.377256	0.218397	-1314.750848	-1314.532451	-384.139
**INT6**	-1313.388574	0.221025	-1314.767012	-1314.545987	
**TS5**	-1389.709372	0.239159	-1391.205863	-1390.966704	-1056.57
**3a**	-1313.920973	0.23503	-1315.30586	-1315.07083	
**INT5’**	-1313.421166	0.217432	-1314.793392	-1314.57596	
**TS4’**	-1313.394732	0.215269	-1314.762052	-1314.546783	-320.44
**INT6’**	-1313.405816	0.217241	-1314.776444	-1314.559203	
**TS5’**	-1389.711846	0.239877	-1391.210959	-1390.971082	-467.49
**INT7**	-1313.868112	0.229466	-1315.251758	-1315.022292	
**INT8**	-1313.873871	0.230297	-1315.257495	-1315.027198	
**TS6**	-1313.82357	0.232396	-1315.212216	-1314.97982	-166.37

The coordinates of all stationary points (Å)

2a

C	-0.91296700	0.51719100	-0.45972500
C	0.39942600	0.06535100	-0.24368000
C	-3.18634900	2.84535400	0.24136000
C	-2.50893800	2.34535700	-0.96557800
H	-3.13540700	1.58669900	-1.45273800
H	-2.38277200	3.17133000	-1.67901300
C	-3.73882200	3.25608800	1.23507000
S	0.63490800	-1.64345800	0.18734100
N	-1.19318100	1.79570700	-0.68396600
C	1.53696700	0.97917400	-0.26551000
O	1.38654700	2.19806300	-0.34547200
C	-2.06540600	-0.43412100	-0.42182400
C	-2.86164800	-0.49534900	0.72590400
C	-2.35392800	-1.24191800	-1.52610700
C	-3.94671600	-1.37138600	0.76763800
H	-2.62504600	0.13400800	1.58693500
C	-3.44408300	-2.10886000	-1.47959600
H	-1.72326100	-1.19128200	-2.41640800
C	-4.23981800	-2.17457700	-0.33417300
H	-4.56519900	-1.42224500	1.66527500
H	-3.67331900	-2.73696300	-2.34212900
H	-5.09183300	-2.85587700	-0.30130400
C	2.94265200	0.44842900	-0.20574600
C	3.38114700	-0.57777000	-1.05082000
C	3.85310400	1.07721100	0.64984500
C	4.71579100	-0.97837200	-1.02886800
H	2.68429100	-1.05059800	-1.74707500
C	5.18206500	0.65843400	0.68819100
H	3.51085800	1.89263000	1.28985700
C	5.61456800	-0.36854500	-0.15197100
H	5.05580700	-1.76908800	-1.69979500
H	5.88442900	1.13890200	1.37163200
H	6.65714700	-0.69120600	-0.12794600
C	0.22879900	-1.59842500	1.84697700
N	-0.06266700	-1.59824200	2.96804200
H	-0.39826900	2.43827700	-0.61794100
H	-4.23244800	3.62439100	2.12010800

HO^-

O	0.00000000	0.00000000	0.10854100
H	0.00000000	0.00000000	-0.86833100

INT1

C	-1.17438600	0.45703400	-0.23099600

C	0.20666300	-0.03372100	-0.21417900
C	-2.67050200	3.56912000	0.48737600
C	-2.72711700	2.27789300	-0.21874200
H	-3.43731700	1.62122000	0.31256600
H	-3.15689500	2.46005000	-1.21933700
C	-2.62398900	4.62773200	1.07122100
S	0.55343600	-1.44720500	-1.20479000
N	-1.39516500	1.71883700	-0.34908300
C	1.24854400	0.63212900	0.49445100
O	1.08894200	1.58759600	1.26605800
C	-2.26481000	-0.56956000	-0.09978800
C	-2.23898700	-1.45638600	0.98377600
C	-3.29548000	-0.67151100	-1.04190800
C	-3.23195300	-2.42363900	1.12997200
H	-1.42834900	-1.38410300	1.71305200
C	-4.28089700	-1.64940900	-0.90420100
H	-3.31580900	0.00964200	-1.89589200
C	-4.25243100	-2.52452700	0.18291700
H	-3.20545300	-3.10512300	1.98228500
H	-5.07349400	-1.73052600	-1.65044000
H	-5.02520500	-3.28797500	0.29096200
C	2.67912200	0.17607400	0.28069600
C	3.49990400	1.00584600	-0.49195900
C	3.22223500	-0.95977400	0.88771100
C	4.84731600	0.69301500	-0.66995200
H	3.05495100	1.89338700	-0.95128300
C	4.57334000	-1.26696900	0.71620600
H	2.58810500	-1.60873500	1.49794700
C	5.38692700	-0.44404400	-0.06471400
H	5.48046800	1.33932500	-1.28158900
H	4.99346300	-2.15462400	1.19371500
H	6.44239200	-0.68779100	-0.20042100
C	0.51625100	-2.76591200	-0.09936200
N	0.51285800	-3.66187800	0.63727700
H	-2.58310600	5.56977300	1.59290500
O	0.91906700	3.38465500	-0.83672300
H	1.30312200	3.15892800	0.02209400
H	0.10173900	2.84475900	-0.77311000

INT2

C	-1.13145000	0.57808200	-0.34539000
C	0.26867000	0.15890200	-0.33770000
C	-2.92294900	3.58603700	0.19634500
C	-2.83135000	2.23710800	-0.39168500

H	-3.46995800	1.56187700	0.20581500
H	-3.29168700	2.28358200	-1.39547900
C	-3.00374800	4.69167800	0.68174300
S	0.62453000	-1.48341500	-0.87138200
N	-1.44725700	1.81787300	-0.49265200
C	1.32184900	0.96931600	0.21935400
O	1.15096400	1.99396100	0.87799900
C	-2.17398100	-0.49777900	-0.16993200
C	-2.27116700	-1.18571600	1.04539500
C	-3.07289900	-0.80023800	-1.19871900
C	-3.25256100	-2.16014100	1.22915800
H	-1.56808900	-0.95637400	1.85029200
C	-4.04654600	-1.78332500	-1.02010700
H	-3.00407600	-0.26446000	-2.14883100
C	-4.13934800	-2.46389300	0.19499100
H	-3.32215900	-2.68753000	2.18265000
H	-4.73671000	-2.01772600	-1.83287600
H	-4.90307600	-3.23102900	0.33610600
C	2.76693900	0.53459500	0.02545700
C	3.30546800	0.26487100	-1.23801100
C	3.61463400	0.53189100	1.13815300
C	4.66486200	-0.01061200	-1.38390000
H	2.65473200	0.28304100	-2.11550700
C	4.96990300	0.23180900	0.99904200
H	3.19927400	0.77334500	2.11896800
C	5.49881100	-0.03786400	-0.26398500
H	5.07644100	-0.20426400	-2.37666000
H	5.61772400	0.21767100	1.87793400
H	6.56122700	-0.26215600	-0.37769800
C	0.71172100	-2.38033400	0.59559900
N	0.79033300	-2.98587900	1.58206300
H	-3.07652000	5.67668000	1.11268400

INT2‘

C	-1.05597500	0.45266600	-0.19305400
C	0.30954000	-0.04970800	-0.23284800
C	-2.42902400	3.84356500	0.08129600
C	-2.46090000	2.45485500	-0.05516800
C	-2.40866200	5.06639600	0.20142000
S	0.52670300	-1.80008100	-0.14220200
N	-1.27936800	1.74580800	-0.07452000
C	1.41687600	0.83416400	-0.15309800
O	1.26929500	2.07890400	-0.04343600
C	-2.23355300	-0.45770200	-0.26357200

C	-2.97835700	-0.71994900	0.89370200
C	-2.61232200	-1.05166000	-1.47296000
C	-4.07712700	-1.57724700	0.84377600
H	-2.68599900	-0.25327500	1.83785100
C	-3.71394300	-1.90608300	-1.52236300
H	-2.03263000	-0.84693700	-2.37617200
C	-4.44583400	-2.17163800	-0.36421100
H	-4.64587300	-1.78498500	1.75211600
H	-4.00066800	-2.36822200	-2.46893700
H	-5.30574800	-2.84303100	-0.40212300
C	2.83672400	0.33431300	-0.19344100
C	3.30379100	-0.56623400	-1.15907600
C	3.74570800	0.87851400	0.72302400
C	4.64913800	-0.93298000	-1.19081400
H	2.61544700	-0.96642100	-1.90652100
C	5.08673200	0.49988300	0.70303100
H	3.38502200	1.60495200	1.45367100
C	5.54167300	-0.40864900	-0.25428900
H	5.00363300	-1.62776800	-1.95463600
H	5.78203000	0.91900200	1.43310500
H	6.59337700	-0.70079300	-0.27678500
C	0.20582700	-2.09224600	1.51890000
N	-0.03661000	-2.29600000	2.63414300
H	-0.37331500	2.26831000	-0.00814700
H	-2.39285200	6.13580300	0.31291900
H	-3.39406100	1.90320300	-0.15251200

H_2O

O	0.00000000	0.00000000	0.12051700
H	0.00000000	-0.75422000	-0.48206700
H	0.00000000	0.75422000	-0.48206700

TS1

C	-1.08004900	0.20757800	-0.48619400
C	0.32701800	0.06076500	-0.01792600
C	-3.49385300	2.43238100	-0.83396200
C	-2.76563300	1.43415100	-1.63106400
H	-3.35925700	0.50475200	-1.66371200
H	-2.69370000	1.81115000	-2.66381700
C	-4.08083700	3.24501300	-0.15629500
S	0.56155100	0.49904600	1.65619800
N	-1.41052400	1.26402000	-1.12313100
C	1.35826700	-0.23025400	-0.93767400
O	1.15009300	-0.48245200	-2.13768900

C	-2.01044000	-0.91350100	-0.11927000
C	-3.16040600	-0.70008200	0.64905800
C	-1.68291600	-2.21606300	-0.51998800
C	-3.97463900	-1.77577300	1.00948100
H	-3.41313800	0.31210100	0.97523500
C	-2.50872400	-3.28508900	-0.17930700
H	-0.77457000	-2.37408500	-1.10753600
C	-3.65408300	-3.06691300	0.59092100
H	-4.86333200	-1.60163100	1.61913200
H	-2.25430000	-4.29494000	-0.50691500
H	-4.29372000	-3.90692600	0.86842400
C	2.79825000	-0.29062100	-0.46398600
C	3.21240400	-1.06989700	0.62271200
C	3.76609200	0.38904700	-1.21374400
C	4.56323200	-1.15227300	0.96417700
H	2.47346100	-1.63322800	1.19682200
C	5.11402800	0.32419100	-0.86354300
H	3.44939700	0.97166400	-2.08146200
C	5.51684100	-0.44787600	0.22806200
H	4.87160100	-1.77272000	1.80818700
H	5.85566100	0.87237600	-1.44828600
H	6.57286300	-0.50673900	0.49892300
C	0.45511600	2.22419800	1.60704400
N	0.36070000	3.37973700	1.57682700
H	-4.60576300	3.97291700	0.44047600

INT3

C	0.99513000	0.16175700	-0.40081700
C	0.66286800	-1.24978100	-0.36543700
C	4.01529500	1.99886600	-0.25811600
C	2.59438800	1.92491700	-0.64799100
H	2.01475600	2.52280900	0.07861200
H	2.48961200	2.44518600	-1.61804900
C	5.17846900	2.05424800	0.07209500
S	2.00798800	-2.37263000	-0.65750500
N	2.18155400	0.54342800	-0.75622900
C	-0.56079900	-1.92270100	-0.00476900
O	-0.61167300	-3.09026700	0.39052700
C	-0.03268200	1.14475100	0.08944000
C	-0.54464300	1.05026300	1.38709000
C	-0.46860500	2.18343000	-0.74212300
C	-1.50259900	1.96000200	1.83606900
H	-0.20270500	0.24566700	2.04254800
C	-1.43118600	3.08791100	-0.29837600

H	-0.06691000	2.26610000	-1.75511200
C	-1.95523300	2.97426000	0.99127900
H	-1.90193600	1.87188400	2.84843700
H	-1.77736300	3.88265200	-0.96199600
H	-2.71203400	3.67957900	1.33995000
C	-1.89971700	-1.23179600	-0.18223900
C	-2.21778300	-0.47445900	-1.31292500
C	-2.89114300	-1.46906800	0.77805100
C	-3.49602300	0.06699200	-1.46422100
H	-1.45872700	-0.30800500	-2.08045600
C	-4.16220100	-0.91649400	0.63874600
H	-2.64493000	-2.09042900	1.64137700
C	-4.46703400	-0.14309200	-0.48454700
H	-3.73485200	0.65529300	-2.35276900
H	-4.92083500	-1.09018400	1.40464100
H	-5.46361300	0.28777500	-0.60016000
C	3.04913000	-2.03200000	0.65065700
N	3.78087900	-1.80014000	1.52092100
H	6.21429100	2.10484400	0.36478800

TS2

C	1.01976900	-0.05157000	0.09433200
C	0.64511200	-1.40920000	-0.09633200
C	3.32520400	2.31397400	-0.60845600
C	2.92847900	1.41072300	0.48800100
H	3.85303300	1.09969500	1.01491500
H	2.31991700	1.98165600	1.21198300
C	3.64523200	3.05234000	-1.51281000
S	2.01425600	-2.56917800	-0.13745000
N	2.29732200	0.21001500	0.02382400
C	-0.61129400	-2.08157300	-0.22123600
O	-0.69527400	-3.31633700	-0.21002300
C	0.01758700	1.00770900	0.43099900
C	-0.87045900	0.85720600	1.50215800
C	-0.03444200	2.17251500	-0.34429600
C	-1.82410200	1.83971800	1.76700000
H	-0.82735600	-0.04657700	2.11363300
C	-0.98805200	3.15388600	-0.07996900
H	0.65986500	2.29419900	-1.17876700
C	-1.89024300	2.98578000	0.97164500
H	-2.52172700	1.70857300	2.59650000
H	-1.03025600	4.05041600	-0.70131700
H	-2.64279400	3.75016700	1.17441800
C	-1.89074700	-1.30083100	-0.39770900

C	-2.03124300	-0.28787900	-1.35132900
C	-2.99070500	-1.65446400	0.39179600
C	-3.24008000	0.39703500	-1.47798900
H	-1.18347900	-0.02637900	-1.98801900
C	-4.19479300	-0.96206100	0.27685900
H	-2.88430300	-2.46722800	1.11315100
C	-4.31938500	0.07166100	-0.65399900
H	-3.34004700	1.19035000	-2.22183300
H	-5.04032200	-1.22856300	0.91427800
H	-5.26128400	0.61632100	-0.74466800
C	3.33293000	-1.56415400	0.37917400
N	4.43517700	-1.45268200	0.76842000
H	3.93273600	3.70868200	-2.31764100

INT4

C	0.96922100	-0.19818000	0.29828200
C	0.57937400	-1.50365000	0.07170100
C	3.44482100	1.99451300	-0.50867400
C	2.97233600	1.25086500	0.67273400
H	3.84988600	1.00126600	1.28741300
H	2.30009700	1.88604400	1.26424800
C	3.82803900	2.61270800	-1.47579900
S	1.96085400	-2.59501000	-0.03480700
N	2.31567200	0.00354200	0.34149000
C	-0.76024700	-2.09185600	-0.00931000
O	-0.93077600	-3.28880000	0.17289000
C	0.04968200	0.94620900	0.53018400
C	-0.90236100	0.87934900	1.55360000
C	0.08676800	2.07522300	-0.30041500
C	-1.83450100	1.90357800	1.71632400
H	-0.92382700	0.00637900	2.20903500
C	-0.83945800	3.10181400	-0.13061200
H	0.82469000	2.13360500	-1.10381400
C	-1.80866600	3.01362100	0.87140500
H	-2.58342000	1.83231300	2.50735800
H	-0.81338400	3.96974800	-0.79200100
H	-2.54150700	3.81296400	0.99622900
C	-1.96008000	-1.24738200	-0.35341300
C	-1.93185600	-0.29258800	-1.37399400
C	-3.15229600	-1.47746600	0.34199100
C	-3.07210600	0.45546600	-1.66552500
H	-1.00816200	-0.12573300	-1.93184900
C	-4.28806300	-0.72029900	0.06133700
H	-3.17316300	-2.24327100	1.12006500

C	-4.24736800	0.25237200	-0.93986500
H	-3.04127200	1.20209700	-2.46173400
H	-5.20879900	-0.88886100	0.62312900
H	-5.13551900	0.84714100	-1.16225000
C	3.22341400	-1.11545100	0.07619100
N	4.42928100	-1.11729100	-0.00731300
H	4.17233100	3.16003400	-2.33813100

TS3

C	-0.97850000	-0.26918200	-0.41117500
C	-0.49792000	-1.54133800	-0.27532900
C	-3.47857200	1.91843200	0.56585600
C	-3.05973500	1.12490300	-0.60288300
H	-3.95991600	0.84791800	-1.17240500
H	-2.42804500	1.73964500	-1.25656700
C	-3.82378500	2.57281500	1.52330400
S	-1.69595000	-2.77226900	0.03863800
N	-2.34925600	-0.09513500	-0.24958800
C	0.93364100	-1.93575600	-0.38424900
O	1.26394800	-2.94655100	-0.97433700
C	-0.12752200	0.90737300	-0.70382000
C	0.77696400	0.86586300	-1.77236700
C	-0.14148000	2.03652200	0.13189200
C	1.68015400	1.90950600	-1.97768000
H	0.77723400	0.00187300	-2.44043500
C	0.75638700	3.07900400	-0.07764900
H	-0.83673800	2.07743200	0.97370300
C	1.67710300	3.01446700	-1.12790700
H	2.38778000	1.85723800	-2.80724300
H	0.74884200	3.94260500	0.59005700
H	2.38699500	3.82865300	-1.28458300
C	1.99414400	-1.11706300	0.30982700
C	1.72311700	-0.35896900	1.45358100
C	3.30379100	-1.17606100	-0.18216700
C	2.74566200	0.35067000	2.08354300
H	0.70700800	-0.32480400	1.85155800
C	4.32177100	-0.45529200	0.43694600
H	3.50827300	-1.78617000	-1.06390400
C	4.04289700	0.31167600	1.57109000
H	2.52629000	0.93759100	2.97748400
H	5.33706800	-0.49133300	0.03757900
H	4.84026300	0.87505200	2.05982100
C	-3.19191000	-1.12664200	0.19336400
N	-4.33315800	-1.24727800	0.50805800

H	-4.13177200	3.15433100	2.37707400

INT5

C	-1.11350500	-0.32116100	-0.49920100
C	-0.15326800	-1.08533600	-1.10705100
C	-1.37758500	-1.34715500	2.35717000
C	-2.04036200	-1.95009400	1.19412300
H	-1.42591500	-2.78200300	0.81192300
H	-3.01845300	-2.35527300	1.48660700
C	-0.79643900	-0.85844400	3.29941400
S	-0.14599200	-2.78654200	-1.37699400
N	-2.23885000	-0.98727500	0.10480100
C	1.17065800	-0.49341600	-1.51871200
O	1.43028400	-0.19950400	-2.66693000
C	-1.06622700	1.14518000	-0.30833800
C	-0.43954100	2.00103800	-1.23544300
C	-1.67926900	1.73904600	0.81365700
C	-0.37635300	3.37634000	-1.01684500
H	-0.02618700	1.59037800	-2.15563800
C	-1.62322100	3.11455100	1.02297400
H	-2.19712600	1.10956300	1.53881600
C	-0.96087700	3.94336900	0.11564200
H	0.11800600	4.01250300	-1.75396600
H	-2.09964600	3.54274400	1.90729300
H	-0.91646600	5.02115100	0.28134600
C	2.19808200	-0.32532200	-0.43211000
C	2.01495600	-0.84870600	0.85374700
C	3.35924300	0.39924400	-0.72622500
C	2.98400500	-0.64449600	1.83476000
H	1.12123600	-1.43319900	1.07881300
C	4.32410600	0.60768900	0.25644300
H	3.48574400	0.79899000	-1.73391800
C	4.13669800	0.08584800	1.53862900
H	2.83963900	-1.05940600	2.83434900
H	5.22469000	1.17923800	0.02445200
H	4.89308400	0.24662100	2.30934100
H	-0.28820600	-0.41760300	4.14175800
C	-3.46238500	-0.71997400	-0.32051800
N	-4.53387600	-0.46732700	-0.69573200

TS4

C	1.17931000	0.14921800	0.38823800
C	0.56686700	-0.91588600	0.96279500
C	1.86368600	-2.24440400	-1.12822500

C	2.89655100	-1.18614200	-0.99049000
H	3.81013500	-1.61217100	-0.55022100
H	3.12901800	-0.82146400	-2.00268800
C	1.30462100	-2.92882600	-2.04872000
S	1.30169000	-2.49357900	1.04052600
N	2.48899900	-0.02981200	-0.15662700
C	-0.86303100	-0.87355700	1.43271200
O	-1.13424000	-0.96206700	2.61037000
C	0.55530600	1.47444400	0.18179500
C	-0.26394600	2.05900400	1.16094700
C	0.75416400	2.16210300	-1.02809400
C	-0.89849500	3.27695100	0.91958200
H	-0.38833000	1.56656000	2.12698000
C	0.12475700	3.38187800	-1.26240000
H	1.39290700	1.72240700	-1.79759400
C	-0.70956000	3.94173300	-0.29216800
H	-1.53362000	3.71572500	1.69141700
H	0.28205100	3.89623800	-2.21238200
H	-1.20306000	4.89753600	-0.47710700
C	-1.94542600	-0.78643400	0.39434300
C	-1.67345600	-0.86844600	-0.97689400
C	-3.26621800	-0.62748200	0.83278100
C	-2.71721400	-0.78830900	-1.89834600
H	-0.64773500	-1.00925200	-1.32479000
C	-4.30504400	-0.54013600	-0.08903900
H	-3.45839800	-0.57087800	1.90547300
C	-4.03024300	-0.62026400	-1.45690200
H	-2.50367100	-0.85779900	-2.96632900
H	-5.33226300	-0.40987400	0.25614400
H	-4.84427200	-0.55203900	-2.18119500
H	0.58757100	-3.74148100	-1.91690700
C	3.41503300	0.88738300	0.08352200
N	4.22965500	1.69457500	0.27165500

INT6

C	-0.93455200	0.40904100	-0.14383100
C	-0.90398600	-0.92612400	-0.39131200
C	-3.61022800	-0.97779800	0.45629100
C	-2.98259700	0.13649100	1.24637300
H	-3.77145500	0.77073900	1.66800400
H	-2.34538700	-0.24191200	2.06271200
C	-4.92023100	-1.24902600	0.38688300
H	-5.08068600	-2.14132700	-0.26191700
S	-2.31018300	-1.99774800	-0.33769900

N	-2.10588700	0.98353500	0.40648000
C	0.34231000	-1.66924900	-0.78336700
O	0.30563400	-2.47291800	-1.69049800
C	0.21113700	1.32723600	-0.33512700
C	1.01715100	1.25484400	-1.47903900
C	0.51678100	2.26904000	0.66004200
C	2.13494200	2.07889300	-1.60437600
H	0.76156700	0.55590100	-2.27877700
C	1.63007500	3.09317200	0.52951100
H	-0.10795700	2.32920300	1.55411600
C	2.44664100	2.99524300	-0.59992600
H	2.75892200	2.01272900	-2.49743500
H	1.86732600	3.81098800	1.31666800
H	3.32066000	3.64133300	-0.69969900
C	1.60704400	-1.46833200	0.00165900
C	1.61508100	-0.95183200	1.30243300
C	2.81208300	-1.85542000	-0.59760400
C	2.82087800	-0.80564200	1.98678200
H	0.67780000	-0.66499900	1.78301500
C	4.01632500	-1.69771700	0.08247500
H	2.78879000	-2.26864200	-1.60740000
C	4.02154900	-1.16882400	1.37518900
H	2.82279300	-0.40561800	3.00209500
H	4.95421100	-1.98578400	-0.39563400
H	4.96545600	-1.04358900	1.90913700
C	-2.52574900	2.17968400	0.01294200
N	-2.89155800	3.23592200	-0.30129400

TS5

C	-0.61663700	0.30133200	-0.23165300
C	-0.48966500	-1.04070000	-0.41228600
C	-3.19759400	-1.20559700	0.14062200
C	-2.76670600	-0.02811900	0.97093600
H	-3.69037100	0.52388300	1.22693300
H	-2.22221000	-0.34107800	1.87651000
C	-4.49091500	-1.46831900	-0.07921000
H	-4.73164800	-2.33970300	-0.70730100
S	-1.83494300	-2.19842500	-0.46030000
N	-1.84920100	0.83352300	0.20294000
C	0.81235800	-1.73549500	-0.68020300
O	0.85453000	-2.63411000	-1.49447400
C	0.48628100	1.27454200	-0.40616600
C	1.35605300	1.19252800	-1.50089800
C	0.68505000	2.27584900	0.55725900

C	2.43362100	2.07059400	-1.60788900
H	1.18470500	0.44148100	-2.27549100
C	1.75856900	3.15388700	0.44454400
H	0.01175400	2.34212300	1.41481600
C	2.63974000	3.04846000	-0.63436000
H	3.11014700	1.99606700	-2.46097000
H	1.91419600	3.91947000	1.20658700
H	3.48308200	3.73601400	-0.71966100
C	2.03620100	-1.38277300	0.11513800
C	1.97230100	-0.80241200	1.38691500
C	3.28101600	-1.71023400	-0.43594500
C	3.14643800	-0.53450900	2.08954900
H	1.00409300	-0.56474400	1.83205800
C	4.45257500	-1.43048900	0.26141900
H	3.31575100	-2.17665800	-1.42199500
C	4.38591200	-0.83936800	1.52524500
H	3.09175000	-0.08634500	3.08314700
H	5.42097300	-1.67250300	-0.17996500
H	5.30344800	-0.61904200	2.07419500
C	-2.27978300	2.01447800	-0.23015900
N	-2.66959000	3.04957100	-0.58132100
O	-5.71744900	0.55428300	1.07606800
H	-5.25029300	-0.59106500	0.46543300
H	-5.68027700	1.14192100	0.30927800

3a

C	-0.90594700	0.40930300	-0.08113400
C	-0.89430100	-0.93221700	-0.29341700
C	-3.55949600	-0.90154600	0.36223200
C	-3.00803900	0.20803000	1.21204500
H	-3.81575000	0.85323600	1.57427900
H	-2.45300400	-0.19839200	2.07146700
C	-4.86248500	-1.11829800	0.17017000
H	-5.59724700	-0.48233800	0.66960100
H	-5.22639800	-1.92954200	-0.46481200
S	-2.33508600	-1.97287000	-0.33088100
N	-2.06884400	1.02192400	0.42728800
C	0.32494100	-1.73219000	-0.64469900
O	0.22577500	-2.63763700	-1.44625400
C	0.25204100	1.29783000	-0.32689700
C	0.99840400	1.17628600	-1.50524900
C	0.62409500	2.24743400	0.63619700
C	2.12886100	1.96727300	-1.70074600
H	0.68654200	0.46355700	-2.27212400

C	1.75295200	3.03595700	0.43610300
H	0.04561500	2.34093700	1.55820700
C	2.51066600	2.89207100	-0.72885200
H	2.70896200	1.86593700	-2.61944500
H	2.05011600	3.76028900	1.19624100
H	3.39741000	3.50970900	-0.88234900
C	1.62534100	-1.47281000	0.05507300
C	1.69146100	-0.89940900	1.33009100
C	2.80141900	-1.88433300	-0.58408700
C	2.92737200	-0.72237700	1.94991800
H	0.77665800	-0.59635000	1.84302700
C	4.03558000	-1.69356800	0.03095200
H	2.73335500	-2.34388900	-1.57168800
C	4.09904800	-1.11001700	1.29843700
H	2.97605600	-0.28001900	2.94635400
H	4.95117300	-1.99993000	-0.47779800
H	5.06622200	-0.96156900	1.78254700
C	-2.41339400	2.25728500	0.06415800
N	-2.71910400	3.33767000	-0.22316700

INT5’

C	-1.15700400	-0.30298500	-0.39329700
C	-0.13658300	-1.12784700	-0.79583000
C	-1.48401800	-0.78217200	2.45510100
C	-2.34743300	-1.24985900	1.58128400
H	-3.19055400	-1.87171300	1.89711300
C	-0.64529200	-0.32085200	3.34763200
S	-0.06864800	-2.83623600	-0.67145000
N	-2.30031200	-0.96742100	0.19546500
C	1.14740400	-0.62566300	-1.39713500
O	1.28528700	-0.48760700	-2.59509000
C	-1.21729400	1.17093900	-0.38308100
C	-0.29561100	1.99737100	-1.06159500
C	-2.24017700	1.82133800	0.34199000
C	-0.37787300	3.38613100	-0.98932900
H	0.48302300	1.56988300	-1.68885100
C	-2.32010500	3.21014500	0.40700600
H	-2.98798700	1.22802700	0.86790400
C	-1.38516800	4.00834500	-0.25158300
H	0.35376000	3.98757600	-1.53282000
H	-3.12633700	3.67146800	0.98132100
H	-1.44675200	5.09664500	-0.20112000
C	2.27927900	-0.34748900	-0.44786100
C	2.14874900	-0.53506600	0.93351800

C	3.47756300	0.15239700	-0.97073700
C	3.20955100	-0.22374000	1.78251500
H	1.21948600	-0.94191300	1.33587200
C	4.53683400	0.46251400	-0.12146100
H	3.55969200	0.29491000	-2.04961900
C	4.40275200	0.27541200	1.25642300
H	3.10626000	-0.37310800	2.85897500
H	5.46996100	0.85329300	-0.53148600
H	5.23245400	0.51972000	1.92265600
H	-0.81347600	0.64264800	3.84341300
C	-3.17674900	-1.55322500	-0.61350300
N	-3.95806400	-2.03377900	-1.32586900
H	0.25222100	-0.88256900	3.63303000

TS4'

C	1.10563600	0.44302400	-0.39034100
C	-0.09970200	0.54619300	-1.01628200
C	-0.28434900	3.01318700	0.38995000
C	1.03572100	2.78188800	0.57801100
H	1.68605900	3.50839500	1.06483400
C	-1.32686700	3.57353700	1.03814200
S	-0.80883100	2.05116200	-1.51626500
N	1.72866400	1.63186500	0.07779400
C	-1.01044200	-0.62794800	-1.25381800
O	-0.87837300	-1.37551400	-2.19978800
C	1.75886000	-0.82933100	0.00511100
C	1.86802600	-1.90266300	-0.89228500
C	2.29103500	-0.97375400	1.29849000
C	2.47634500	-3.09431500	-0.49946500
H	1.47710600	-1.79128400	-1.90390300
C	2.90297500	-2.16327800	1.68547200
H	2.20960600	-0.14836700	2.00976600
C	2.99614200	-3.22911500	0.78829000
H	2.55730600	-3.91823900	-1.21101500
H	3.30526000	-2.26116400	2.69560800
H	3.47887000	-4.16016700	1.09117500
C	-2.15824100	-0.79977400	-0.29711800
C	-2.21925700	-0.09407900	0.91030700
C	-3.16690800	-1.71686200	-0.61577400
C	-3.28117800	-0.30280100	1.78808300
H	-1.43456500	0.62292700	1.15926300
C	-4.23070800	-1.92062500	0.25988700
H	-3.10267700	-2.26316700	-1.55837900
C	-4.28832500	-1.21341700	1.46309000

H	-3.32415100	0.25031900	2.72802100
H	-5.01847600	-2.63231700	0.00621400
H	-5.12099300	-1.37323700	2.15081600
H	-2.15988500	4.03743700	0.50395100
C	3.06139800	1.69498400	0.05798900
N	4.22026300	1.75278800	0.06192900
H	-1.36809000	3.55981200	2.13115200

INT6'

C	1.12846200	0.36800500	-0.32633000
C	-0.03476900	0.58978200	-0.97276100
C	-0.19010500	2.98237400	0.39422800
C	0.99079400	2.48653300	0.96750100
H	1.05862300	2.37123500	2.05281500
C	-1.08844700	3.91850300	0.84411700
S	-0.61451800	2.24363700	-1.21964300
N	1.76636900	1.52195700	0.20959900
C	-1.03964800	-0.46624200	-1.34727000
O	-0.98657100	-1.06014700	-2.40094300
C	1.70732500	-0.95693800	-0.00123900
C	1.69024200	-2.01110300	-0.92648000
C	2.29943900	-1.15960200	1.25609300
C	2.23427000	-3.24962500	-0.58894700
H	1.26458200	-1.84915800	-1.91746200
C	2.83982200	-2.39878300	1.58849000
H	2.32136000	-0.34040600	1.97802200
C	2.80697000	-3.44801400	0.66772200
H	2.22108500	-4.06166200	-1.31820200
H	3.28929900	-2.54668700	2.57218400
H	3.23579600	-4.41767700	0.92721100
C	-2.14823400	-0.70100300	-0.36550900
C	-2.16360900	-0.08355000	0.89253700
C	-3.18282200	-1.57336500	-0.72717700
C	-3.20788800	-0.34196200	1.77862100
H	-1.35858200	0.59675900	1.18266700
C	-4.22573400	-1.82595200	0.15883600
H	-3.15364500	-2.04494900	-1.71084900
C	-4.23796800	-1.20973700	1.41264600
H	-3.21872400	0.13839600	2.75825400
H	-5.03285500	-2.50301700	-0.12595500
H	-5.05559500	-1.40695500	2.10863300
H	-2.06772900	4.03475700	0.37897600
C	3.05243600	1.74040400	-0.02846900
N	4.18830200	1.92406900	-0.20094500

H	-0.84466400	4.53182200	1.71530700

TS5'

C	0.64640100	0.26820400	0.27701000
C	0.51359100	-1.08236900	0.38770800
C	3.22822400	-1.21946200	-0.09719100
C	2.87075800	0.03137500	-0.84094300
H	2.36377200	-0.23027200	-1.78644900
C	4.50117400	-1.59473700	0.07423700
S	1.87637500	-2.22096100	0.48258100
N	1.89995200	0.81351900	-0.05414500
C	-0.78027000	-1.80897700	0.57040300
O	-0.81474800	-2.79082900	1.28511500
C	-0.46801600	1.23120200	0.44209000
C	-1.37618200	1.11129900	1.50094600
C	-0.63597300	2.26015600	-0.49722300
C	-2.46383600	1.97853100	1.59285400
H	-1.22956300	0.33516600	2.25569300
C	-1.71988000	3.12720800	-0.40010500
H	0.06946400	2.35705300	-1.32562100
C	-2.64115900	2.98230900	0.64015200
H	-3.17267200	1.87298300	2.41589500
H	-1.85330000	3.91370500	-1.14479500
H	-3.49402600	3.65963400	0.71195900
C	-2.01194000	-1.38761700	-0.17805500
C	-1.96117100	-0.73359600	-1.41382900
C	-3.25159900	-1.72366100	0.37877700
C	-3.14345100	-0.39909500	-2.07291700
H	-0.99711900	-0.48861400	-1.86413900
C	-4.43108800	-1.37607700	-0.27346100
H	-3.27603100	-2.24963200	1.33473600
C	-4.37763200	-0.71034800	-1.50016100
H	-3.10002500	0.10841000	-3.03818100
H	-5.39518700	-1.62394900	0.17407400
H	-5.30104200	-0.43770800	-2.01464400
H	4.78193000	-2.51172300	0.59986900
C	2.26455700	2.00510000	0.41409700
N	2.58222300	3.05064100	0.80471000
H	5.24456800	-0.89561200	-0.33772200
O	5.32225300	1.00160600	-1.25977500
H	3.93834300	0.62209100	-1.04281300
H	5.38858200	0.61185000	-2.14216700

INT7

C	-0.98876700	-0.36561800	0.25941000
C	-0.91826800	0.91988700	-0.27538800
C	-3.61558700	-2.42171900	-1.12392700
C	-3.16303800	-1.07688700	-0.74925200
H	-2.81274600	-0.54866100	-1.65022500
H	-4.01014100	-0.50496500	-0.34006500
C	-3.99064500	-3.53095900	-1.42232800
S	-2.39779500	1.89409500	-0.46473200
N	-2.05935600	-1.16734800	0.19369000
C	0.32481200	1.68720000	-0.49732500
O	0.34383900	2.90120900	-0.39279500
C	0.16446400	-0.94522300	1.00220200
C	0.87761500	-0.15894500	1.91515500
C	0.53226400	-2.28131700	0.79548700
C	1.96508300	-0.69932100	2.59763400
H	0.57157300	0.87440900	2.09559700
C	1.62451400	-2.81501100	1.47402400
H	-0.02003700	-2.89184800	0.07734200
C	2.34354300	-2.02362100	2.37225400
H	2.51794000	-0.08502000	3.31005600
H	1.92031700	-3.85021300	1.29654100
H	3.20076200	-2.44368100	2.90154700
C	1.57561400	0.97108500	-0.93580800
C	1.54688900	-0.19792000	-1.70433700
C	2.80653100	1.55395600	-0.61180600
C	2.73901700	-0.78668900	-2.12576100
H	0.59090500	-0.64687900	-1.98279800
C	3.99642100	0.95871600	-1.02174000
H	2.81476100	2.47483000	-0.02601700
C	3.96349300	-0.21516900	-1.77842400
H	2.71167600	-1.69512700	-2.73015900
H	4.95303500	1.41035900	-0.75290800
H	4.89516600	-0.68230900	-2.10337700
C	-2.41209100	2.66146500	1.06171500
N	-2.45024500	3.19253400	2.09033400
H	-2.05736200	-1.98638500	0.79699000
H	-4.32581400	-4.51947400	-1.69333500

INT8

C	-0.87906600	-0.49011700	0.09519100
C	-0.83644000	0.86558000	-0.21281100
C	-4.10248000	-1.68154600	-1.08852200
C	-2.93895500	-1.06907300	-1.10538700
H	-2.67013400	-0.39035800	-1.92224100

C	-5.25604600	-2.29416600	-1.09563200
S	-2.34230100	1.81825700	-0.29473400
N	-1.92932300	-1.29564400	-0.15506800
C	0.39227500	1.68605900	-0.27104700
O	0.37813100	2.86686700	0.02856900
C	0.25922600	-1.15893700	0.77754200
C	0.90327100	-0.52239400	1.84635300
C	0.68035600	-2.43083200	0.36657400
C	1.97305300	-1.14575300	2.48436900
H	0.55420100	0.45607600	2.18488500
C	1.75679200	-3.04550200	1.00085600
H	0.18508200	-2.92299700	-0.47343000
C	2.40496100	-2.40268500	2.05769600
H	2.46991200	-0.64968500	3.31962000
H	2.09616500	-4.02674400	0.66524000
H	3.24853100	-2.88626400	2.55358500
C	1.66946200	1.07262900	-0.78151100
C	1.68438400	0.05876300	-1.74551800
C	2.87959800	1.59991200	-0.31451000
C	2.89877300	-0.43442600	-2.22244500
H	0.74525800	-0.34081100	-2.13452100
C	4.09144300	1.09523300	-0.77852600
H	2.85415000	2.40436000	0.42289300
C	4.10195600	0.07567600	-1.73333000
H	2.90510200	-1.22051700	-2.97969800
H	5.03169700	1.49821800	-0.39773900
H	5.05124800	-0.31817400	-2.10158100
C	-2.36480700	2.40291700	1.30944100
N	-2.42009200	2.80755300	2.39331300
H	-5.37670500	-3.27639900	-1.56668700
H	-6.13940500	-1.84343400	-0.62919600
H	-1.96397500	-2.18171300	0.34566200

TS6

C	-0.58214700	0.94823100	0.25381600
C	-0.94321800	-0.41108700	0.07258800
C	-3.37780100	0.26672500	1.10485000
C	-2.68984700	1.48516900	1.32061400
H	-3.21719100	2.31233300	1.79329600
C	-4.63668700	-0.07788800	1.47589700
S	-2.56518500	-0.92609400	0.04760100
N	-1.45303500	1.75920300	0.87313800
C	-0.05445000	-1.51225900	-0.35777500
O	-0.46724700	-2.39292000	-1.09223300

C	0.68922700	1.52718400	-0.22144000
C	1.22429700	1.13523700	-1.45858200
C	1.39678300	2.45265400	0.56193900
C	2.44489500	1.64482800	-1.89288000
H	0.67337900	0.43377300	-2.08971000
C	2.61283500	2.96858200	0.11758600
H	1.01242300	2.74806800	1.54164300
C	3.14478300	2.56232300	-1.10672500
H	2.84791400	1.32854200	-2.85670500
H	3.15624200	3.68013400	0.74185900
H	4.10259800	2.95866200	-1.44811100
C	1.34118800	-1.56006900	0.18781700
C	1.69096400	-0.96520000	1.40566800
C	2.30667000	-2.25657100	-0.54863600
C	3.00134600	-1.05199500	1.87180100
H	0.93695900	-0.43884600	1.99427900
C	3.61823300	-2.33212400	-0.08672200
H	2.01648800	-2.72664400	-1.49000600
C	3.96678200	-1.72665100	1.12246200
H	3.27001400	-0.59068000	2.82364700
H	4.37241000	-2.86324400	-0.66993900
H	4.99475400	-1.78515800	1.48517400
C	-3.60047700	-0.45683600	-1.49372300
N	-3.86700200	-0.22194700	-2.59819500
H	-5.05808400	-1.05126200	1.22090900
H	-5.23728500	0.61638500	2.06657600
H	-1.16966800	2.73649200	0.96740700

(i) Reference

(1) Qi, H.; Chi, D.; Chen, S. Pd-Catalyzed C–H Functionalization of Indole-Containing Alkene-Tethered Aryl Halides with Alkynes To Construct Indole Alkaloid Scaffolds. *Org. Lett.* **2022**, *24* (15), 2910–2914.

(2) Cacchi, S.; Fabrizi, G.; Filisti, E. *N* -Propargylic β-Enaminones: Common Intermediates for the Synthesis of Polysubstituted Pyrroles and Pyridines. *Org. Lett.* **2008**, *10* (13), 2629–2632.

(3) Weng, Y.; Kuai, C.; Lv, W.; Cheng, G. Synthesis of 2-Aminopyridines via a Base-Promoted Cascade Reaction of *N* -Propargylic β-Enaminones with Formamides. *J. Org. Chem.* **2018**, *83* (9), 5002–5008.

(4) Cheng, G.; Lv, W.; Xue, L. Base-Promoted Ring-Closing Carbonyl–Allene Metathesis for the Synthesis of 2,4-Disubstituted Pyrroles. *Green Chem.* **2018**, *20* (19), 4414–4417.

(5) Yang, X.; Hu, F.; Wang, Y.; Yang, C.; Zou, X.; Liu, J.; Zhang, Q. Base-Catalyzed Cascade Synthesis of 2,3-Dihydrofuro[2,3-b]Pyridines and 2,3-Dihydro-1H-Pyrrolo[2,3-b]Pyridines from N-Propargylic β-Enaminones. *Chem. Commun.* **2017**, *53* (54), 7497–7500.

(6) Goutham, K.; Mangina, N. S. V. M. R.; Suresh, S.; Raghavaiah, P.; Karunakar, G. V. Gold-Catalysed Cyclisation of N-Propargylic β-Enaminones to Form 3-Methylene-1-Pyrroline Derivatives. *Org Biomol Chem* **2014**, *12* (18), 2869–2873.

(7) Cheng, G.; Xue, L.; Weng, Y.; Cui, X. Transition-Metal-Free Cascade Approach toward 2-Alkoxy/2-Sulfenylpyridines and Dihydrofuro[2,3- *b*]Pyridines by Trapping In Situ Generated 1,4-Oxazepine. *J. Org. Chem.* **2017**, *82* (18), 9515–9524.

(8) Toh, K. K.; Wang, Y.-F.; Ng, E. P. J.; Chiba, S. Copper-Mediated Aerobic Synthesis of 3-Azabicyclo[3.1.0]Hex-2-Enes and 4-Carbonylpyrroles from *N* -Allyl/Propargyl Enamine Carboxylates. *J. Am. Chem. Soc.* **2011**, *133* (35), 13942–13945.

(9) Korkmaz, E.; Zora, M. Synthesis of 3-[(4-Nitrophenyl)Thio]-Substituted 4-Methylene-1-Pyrrolines from *N* -Propargylic β-Enaminones. *J. Org. Chem.* **2020**, *85* (7), 4937–4950.

(10) Kelgokmen, Y.; Cayan, Y.; Zora, M. Zinc Chloride Mediated Synthesis of 1,4-Oxazepines from *N* -Propargylic β-Enaminones. *Eur. J. Org. Chem.* **2017**, *2017* (47), 7167–7178.

(11) Kanova, N.; Dundar, B. A.; Kelgokmen, Y.; Zora, M. One-Pot Synthesis of 2-Acetyl-1 *H* -Pyrroles from *N* -Propargylic β-Enaminones via Intermediacy of 1,4-Oxazepines. *J. Org. Chem.* **2021**, *86* (9), 6289–6304.

(13) Voigt, T.; Gerding-Reimers, C.; Ngoc Tran, T. T.; Bergmann, S.; Lachance, H.; Schölermann, B.; Brockmeyer, A.; Janning, P.; Ziegler, S.; Waldmann, H. A Natural Product Inspired Tetrahydropyran Collection Yields Mitosis Modulators That Synergistically Target CSE1L and Tubulin. *Angew. Chem. Int. Ed.* **2013**, *52* (1), 410–414.

(14) Boehm, P.; Kehl, N.; Morandi, B. Rhodium-Catalyzed Anti-Markovnikov Transfer Hydroiodination of Terminal Alkynes. *Angew. Chem. Int. Ed.* **2023**, *62* (4), e202214071.

(15) Frisch, M. J.; Trucks, G. W.; Schlegel, H. B.; Scuseria, G. E.; Robb, M. A.; Cheeseman, J. R.; Scalmani, G.; Barone, V.; Mennucci, B.; Petersson, G. A.; Nakatsuji, H.; Caricato, M.; Li, X.; Hratchian, H. P.; Izmaylov, A. F.; Bloino, J.; Zheng, G.; Sonnenberg, J. L.; Hada, M.; Ehara, M.; Toyota, K.; Fukuda, R.; Hasegawa, J.; Ishida, M.; Nakajima, T.; Honda, Y.;

Kitao, O.; Nakai, H.; Vreven, T.; Montgomery, J. A., Jr.; Peralta, J. E.; Ogliaro, F.; Bearpark, M.; Heyd, J. J.; Brothers, E.; Kudin, K. N.; Staroverov, V. N.; Kobayashi, R.; Normand, J.; Raghavachari, K.; Rendell, A.; Burant, J. C.; Iyengar, S. S.; Tomasi, J.; Cossi, M.; Rega, N.; Millam, J. M.; Klene, M.; Knox, J. E.; Cross, J. B.; Bakken, V.; Adamo, C.; Jaramillo, J.; Gomperts, R.; Stratmann, R. E.; Yazyev, O.; Austin, A. J.; Cammi, R.; Pomelli, C.; Ochterski, J. W.; Martin, R. L.; Morokuma, K.; Zakrzewski, V. G.; Voth, G. A.; Salvador, P.; Dannenberg, J. J.; Dapprich, S.; Daniels, A. D.; Farkas, Ö.; Foresman, J. B.; Ortiz, J. V.; Cioslowski, J.; Fox, D. J., Gaussian 09, Revision A.01, Gaussian, Inc., Wallingford CT, 2009.

(16) Zhao, Y.; Truhlar, D. G. The M06 suite of density functionals for main group thermochemistry, thermochemical kinetics, noncovalent interactions, excited states, and transition elements: two new functionals and systematic testing of four M06-class functionals and 12 other functionals. *Theor. Chem. Acc.* **2008**, *120*, 215–241.

(17) Weigend, F.; Ahlrichs, R. Balanced basis sets of split valence, triple zeta valence and quadruple zeta valence quality for H to Rn: Design and assessment of accuracy. *Phys. Chem. Chem. Phys.* **2005**, *7*, 3297–3305.

(18) a) Fukui, K. Formulation of the reaction coordinate. *J. Phys. Chem.* **1970**, *74*, 4161–4163; b) Gonzalez, C.; Schlegel, H. B. An improved algorithm for reaction path following. *J. Chem. Phys.* **1989**, *90*, 2154–2161; c) Gonzalez, C.; Schlegel, H. B. Reaction path following in mass-weighted internal coordinates. *J. Phys. Chem.* **1990**, *94*, 5523–5527.

(19) Marenich, A. V.; Cramer, C. J.; Truhlar, D. G. Universal Solvation Model Based on Solute Electron Density and on a Continuum Model of the Solvent Defined by the Bulk Dielectric Constant and Atomic Surface Tensions. *J. Phys. Chem. B* **2009**, *113*, 6378–6396.

(20) Legault, C. Y. CYL view, 1.0b, Université de Sherbrooke, Sherbrooke, Quebec, Canada, 2009, https://www.cylview.org.

(j) NMR Spectra

^{1}H NMR spectrum of compound 1a (400 MHz, $CDCl_3$)

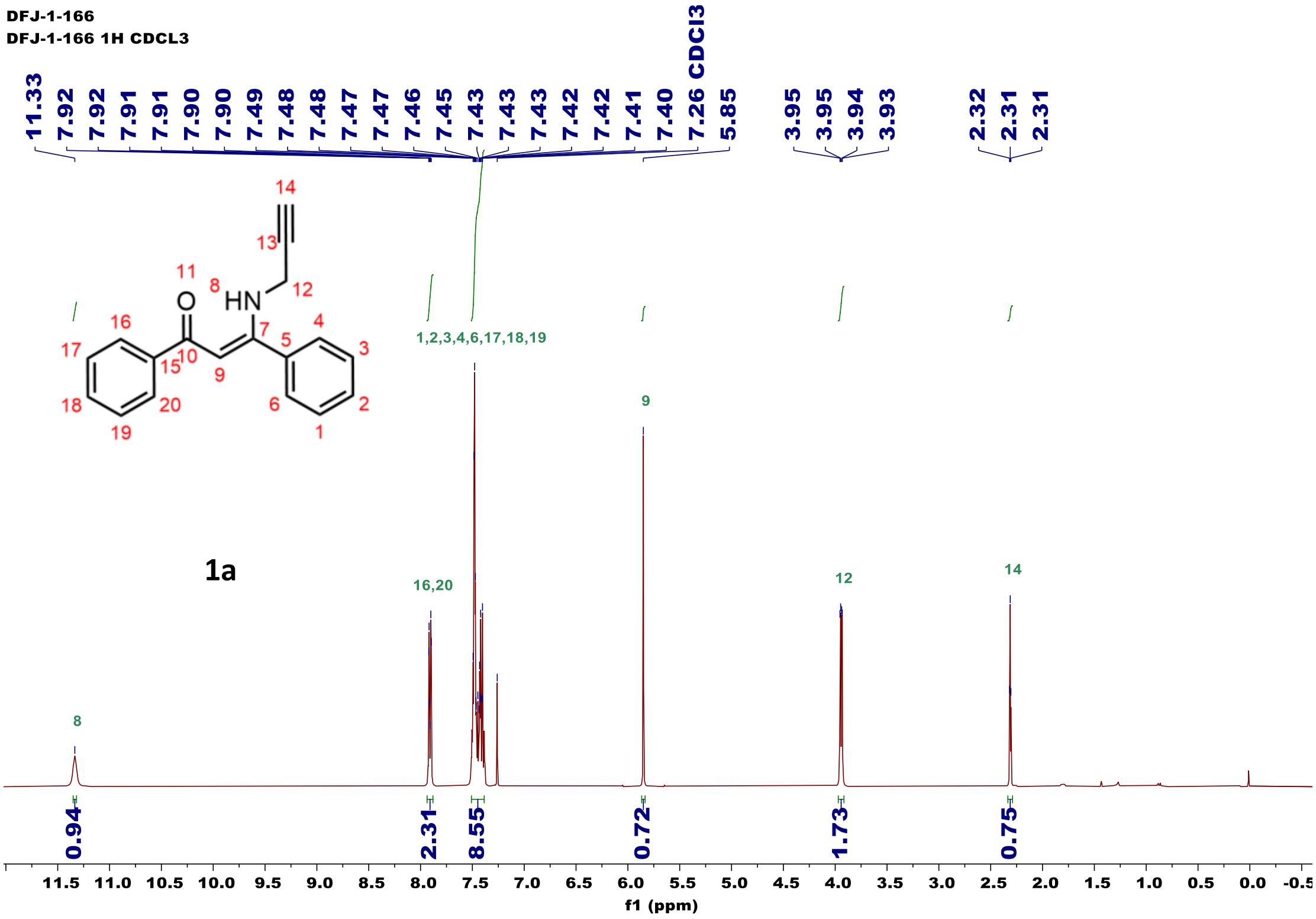

^{13}C NMR spectrum of compound 1a (101 MHz, $CDCl_3$)

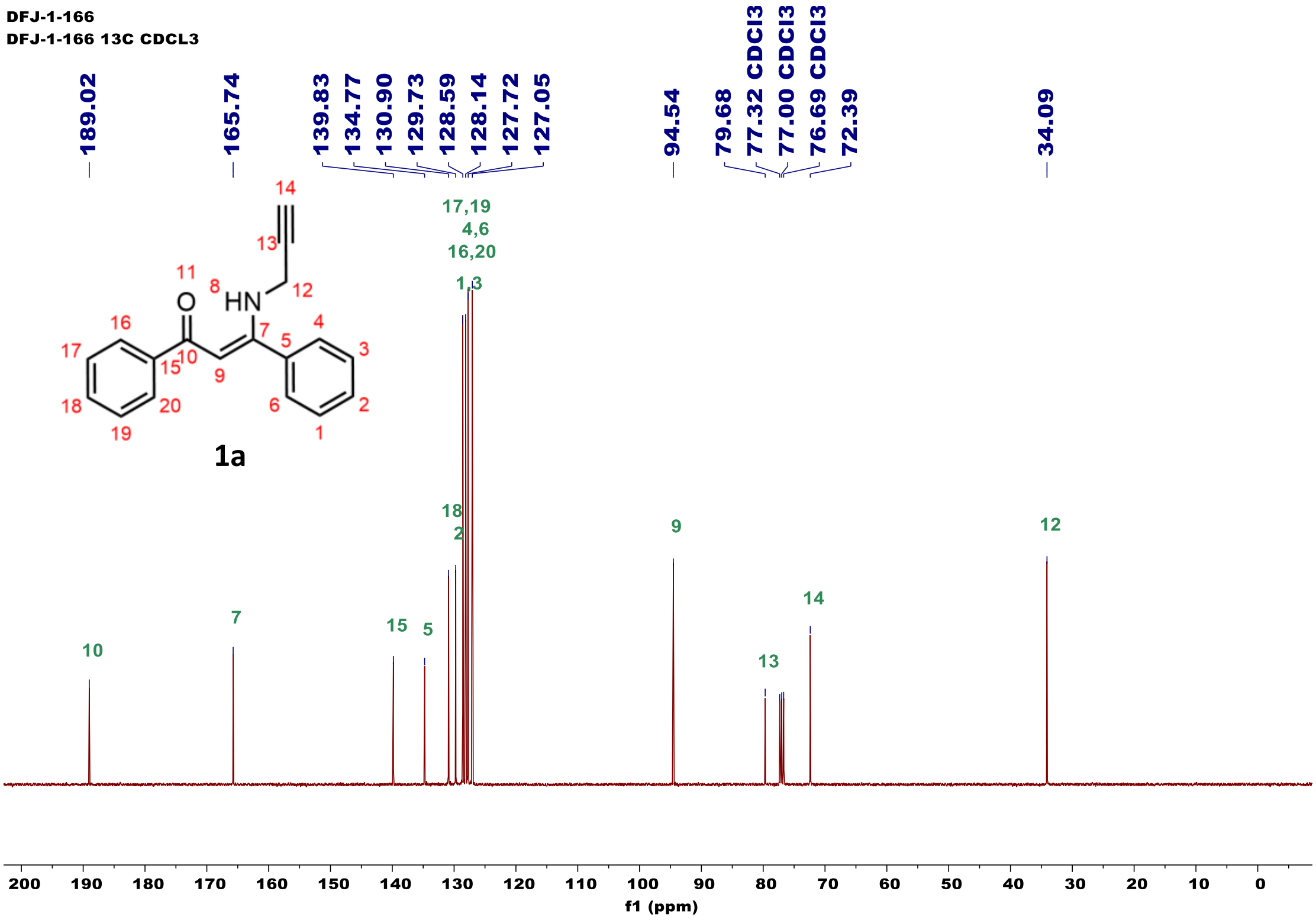

^{1}H NMR spectrum of compound 1b (400 MHz, $CDCl_3$)

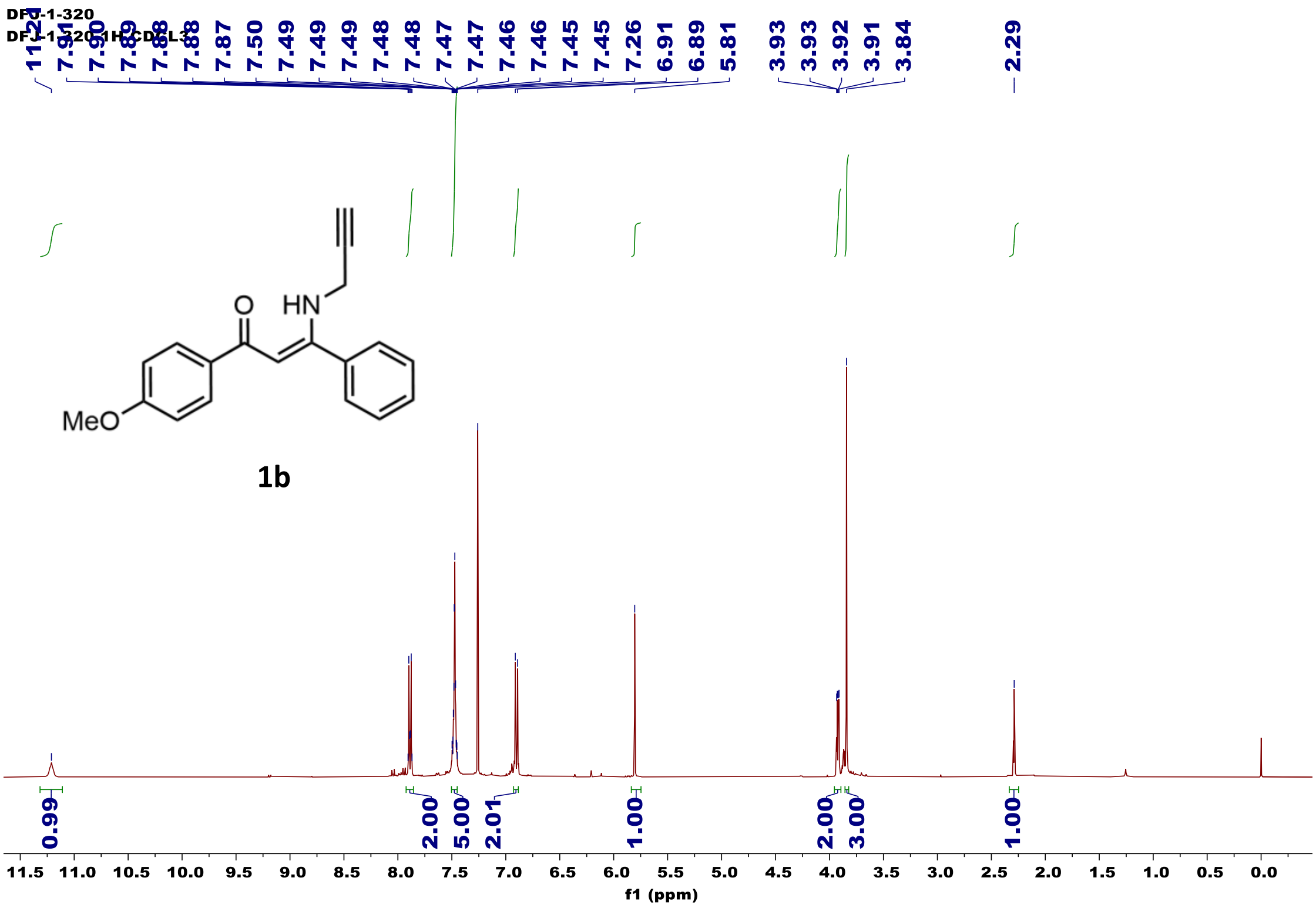

^{13}C NMR spectrum of compound 1b (101 MHz, $CDCl_3$)

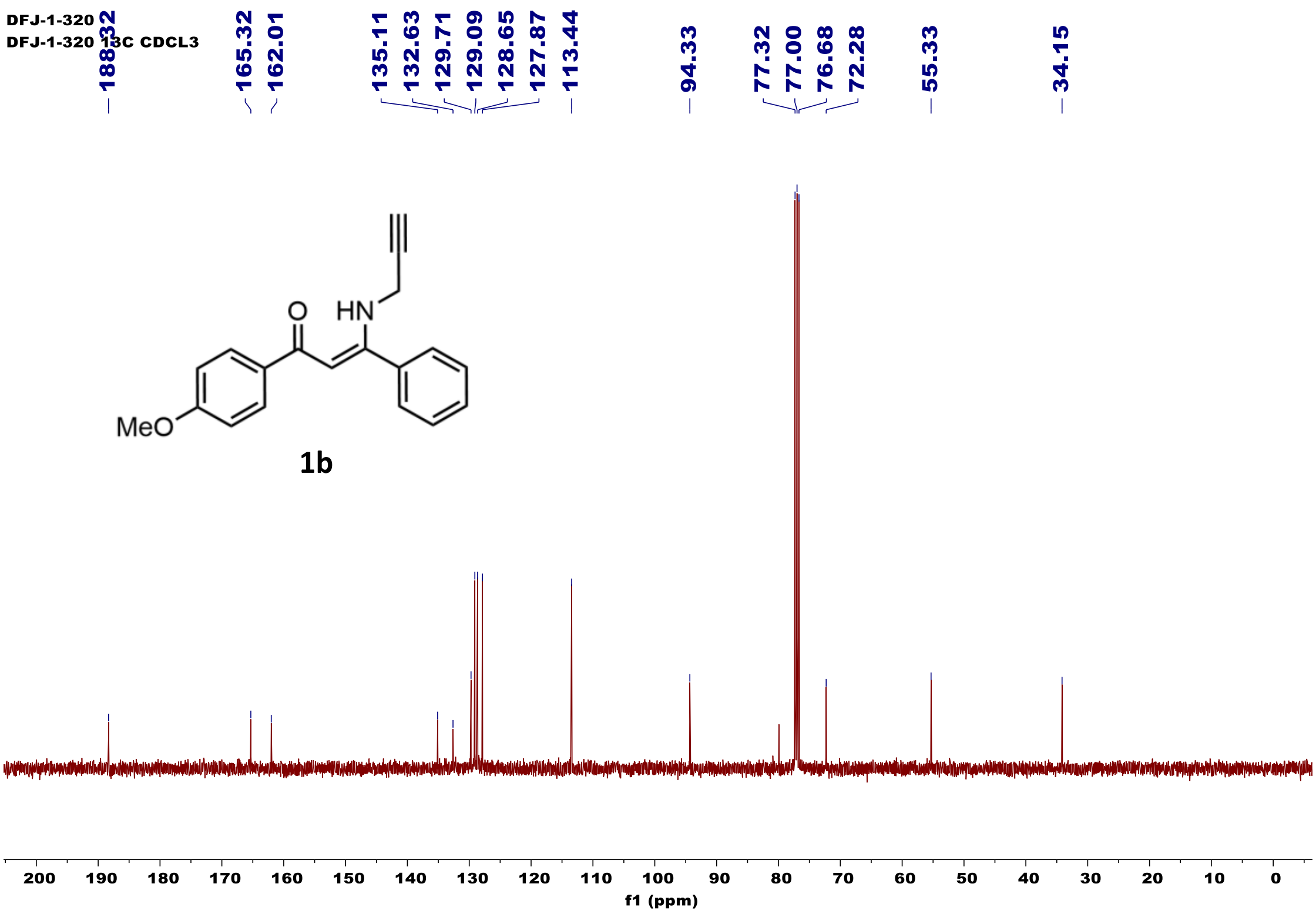

^{1}H NMR spectrum of compound 1c (400 MHz, $CDCl_3$)

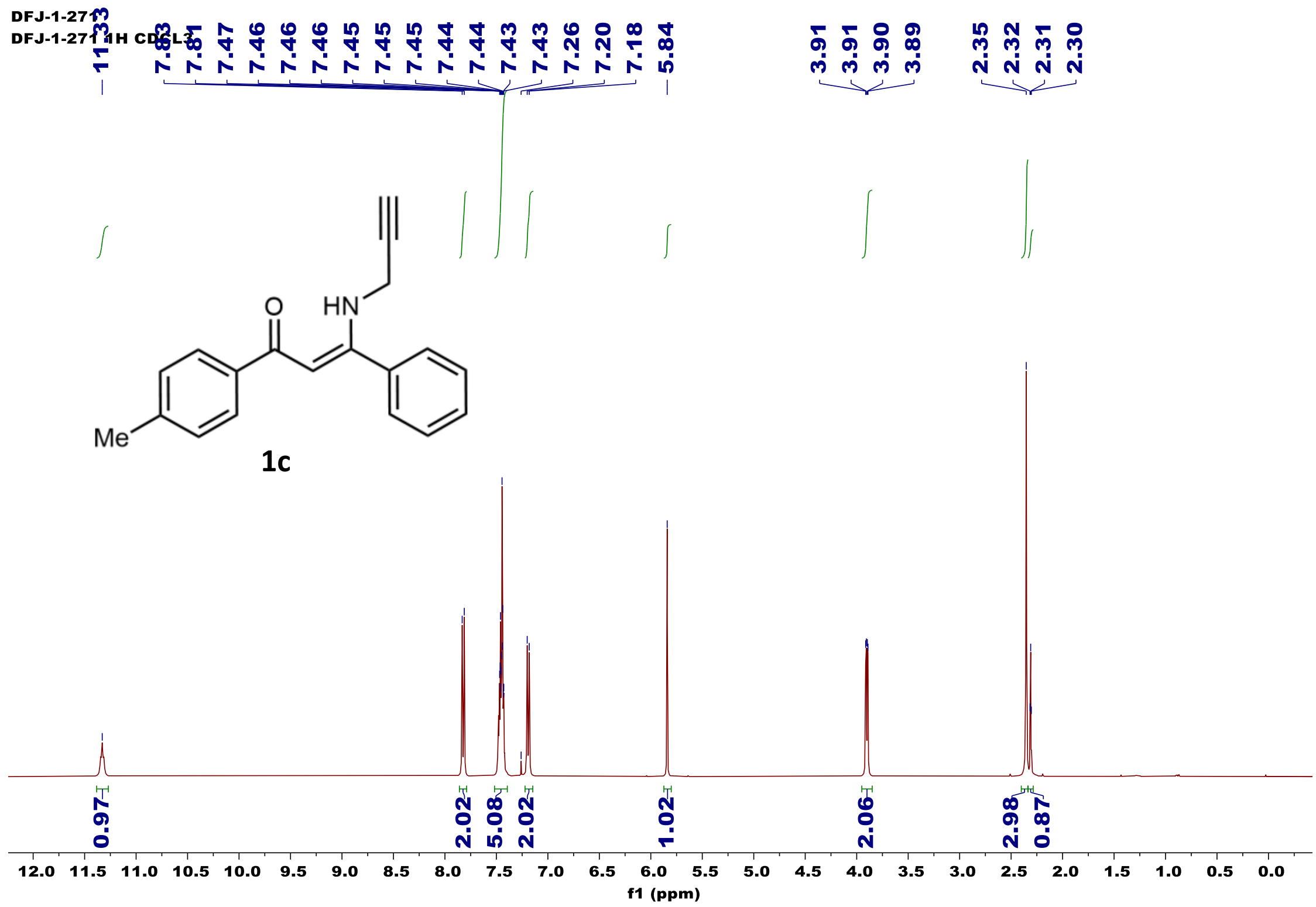

^{13}C NMR spectrum of compound 1c (101 MHz, $CDCl_3$)

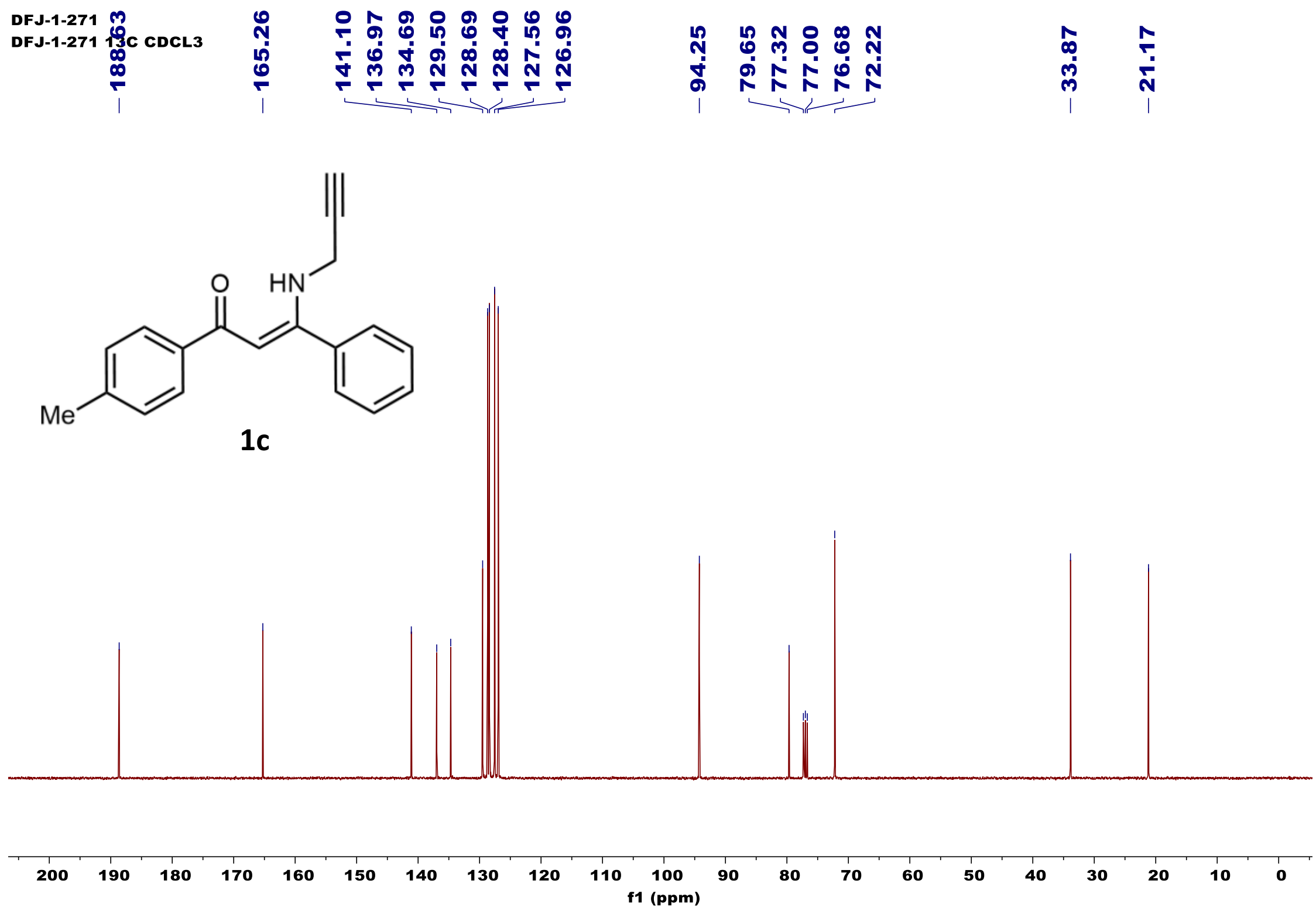

^{1}H NMR spectrum of compound 1d (400 MHz, $CDCl_3$)

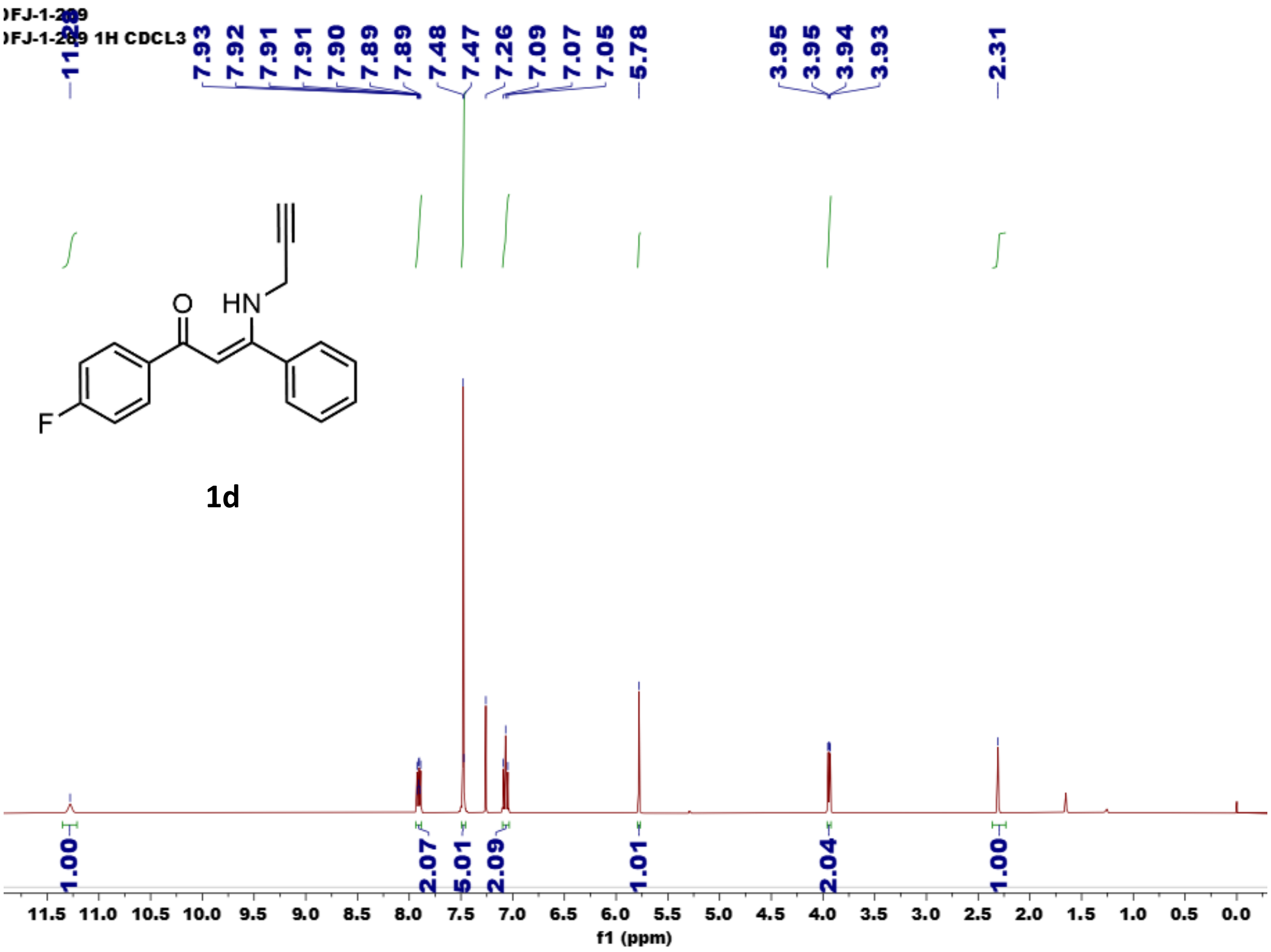

^{13}C NMR spectrum of compound 1d (101 MHz, $CDCl_3$)

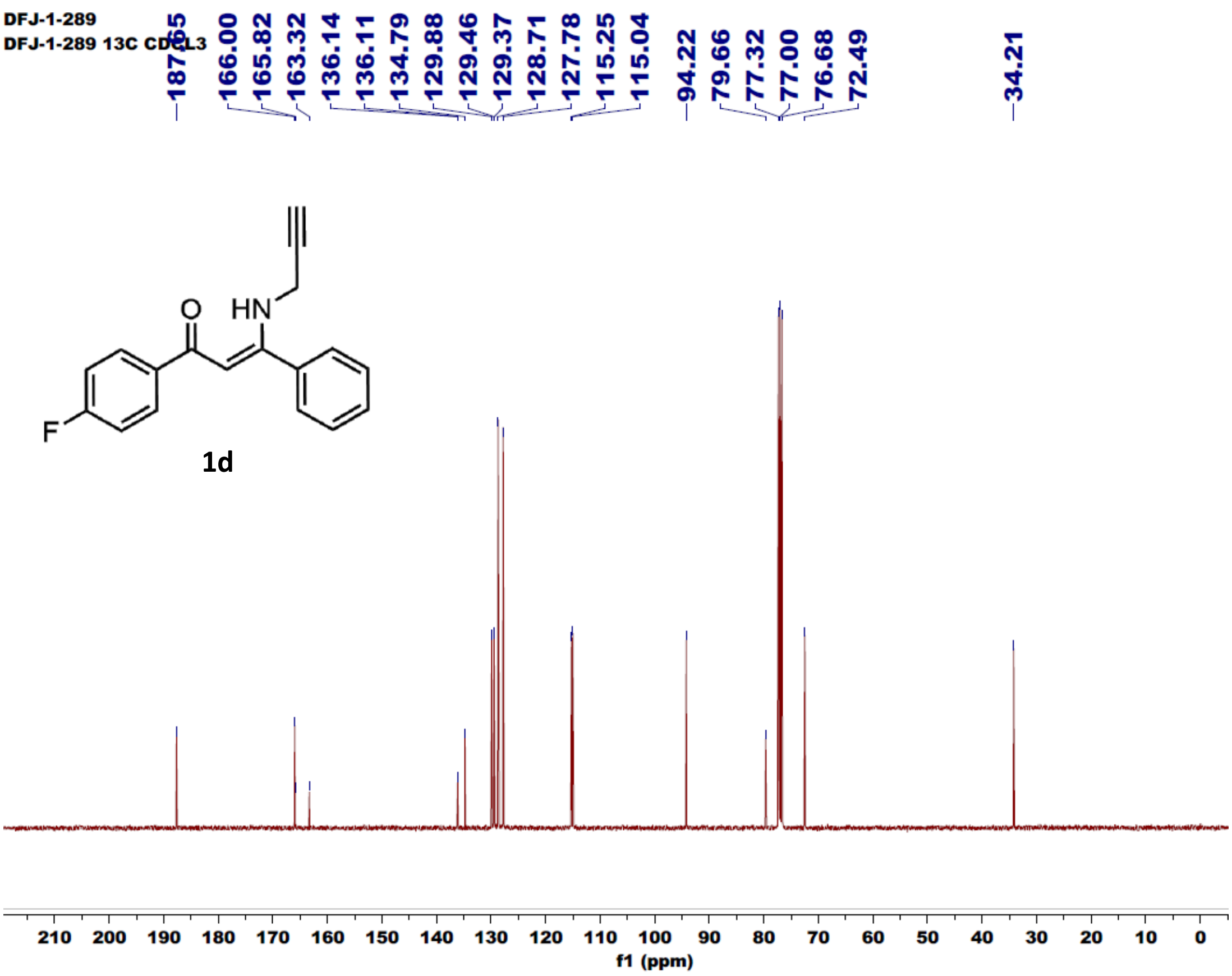

^{1}H NMR spectrum of compound 1e (400 MHz, $CDCl_3$)

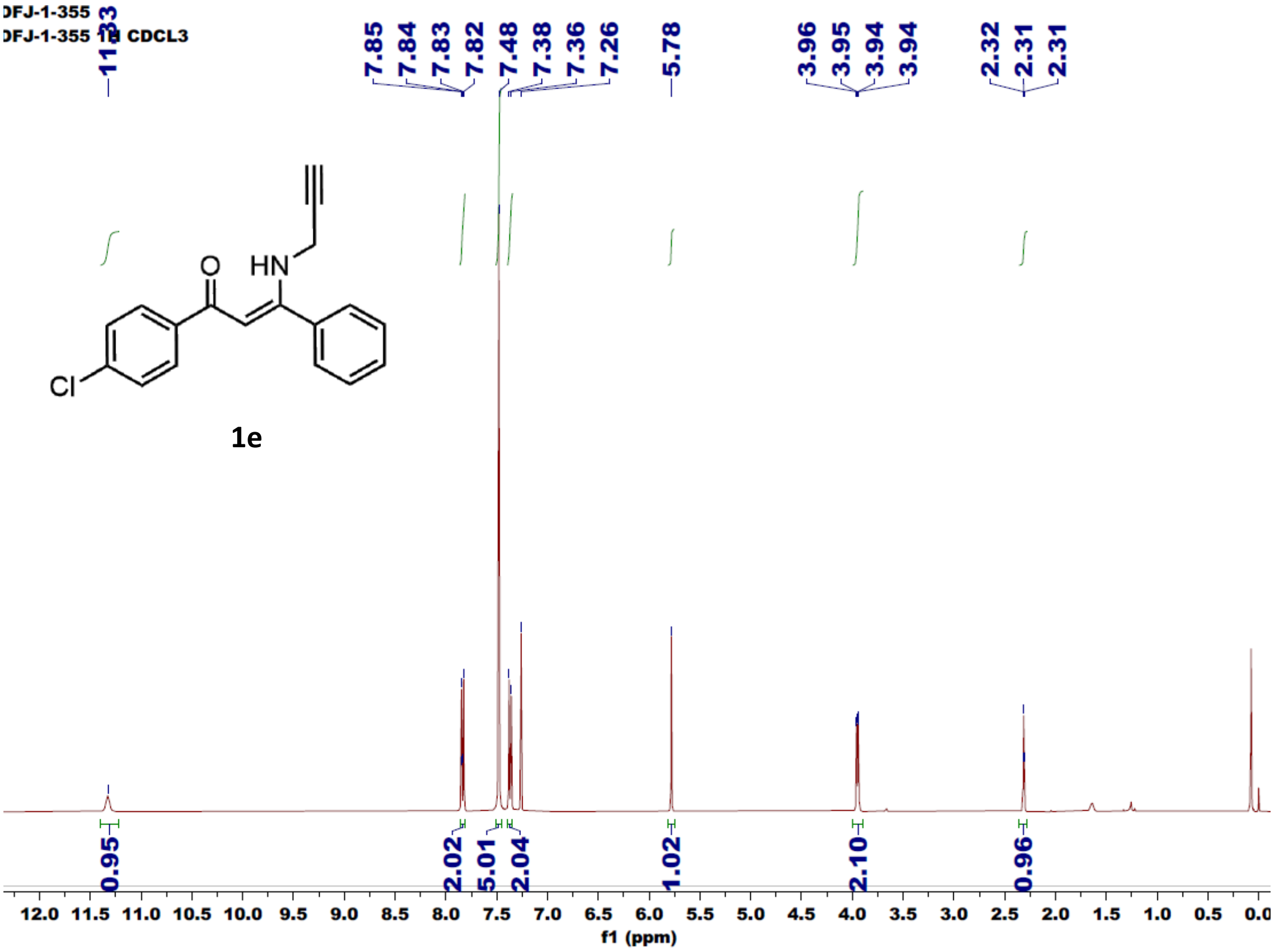

^{13}C NMR spectrum of compound 1e (101 MHz, $CDCl_3$)

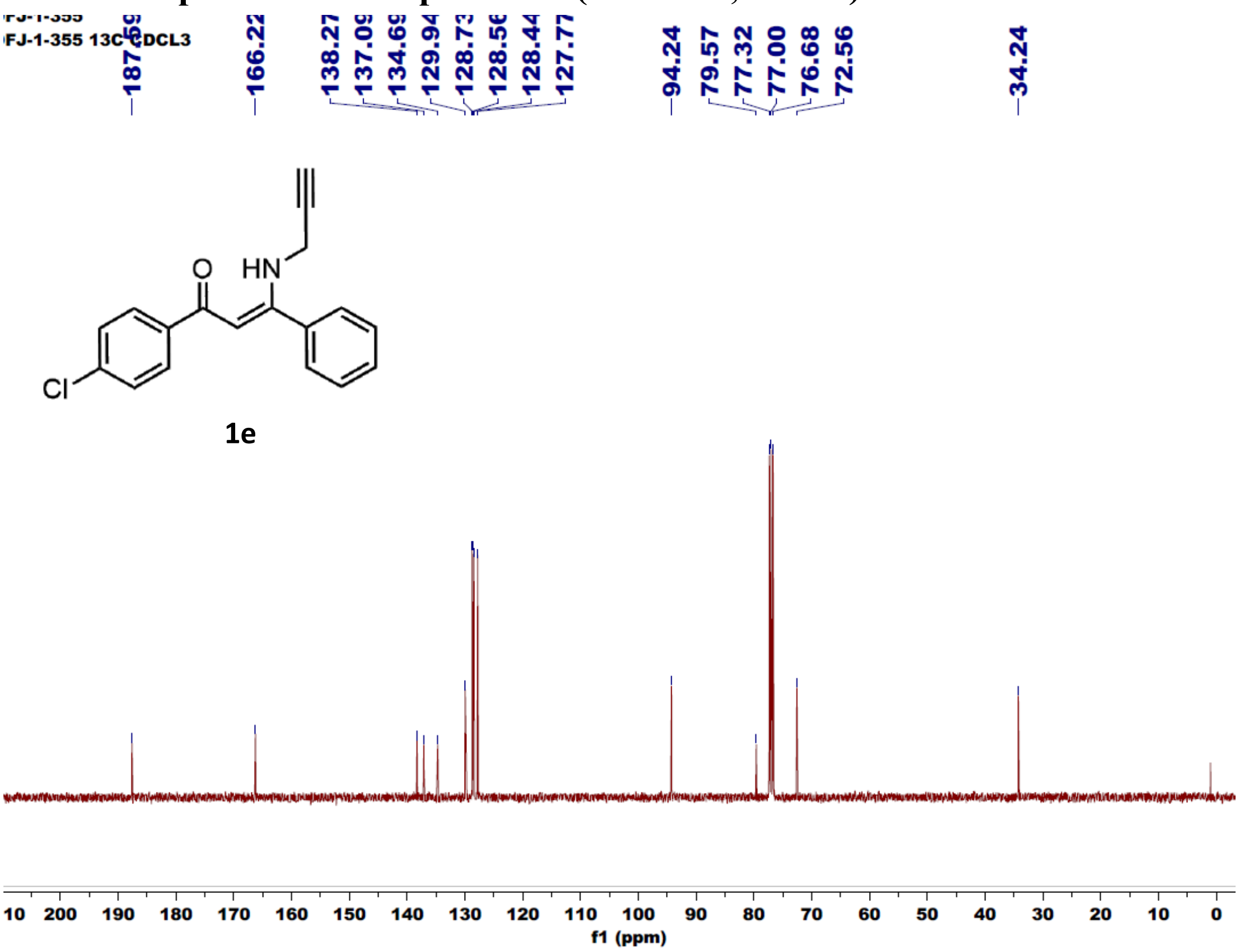

^{1}H NMR spectrum of compound 1f (400 MHz, $CDCl_3$)

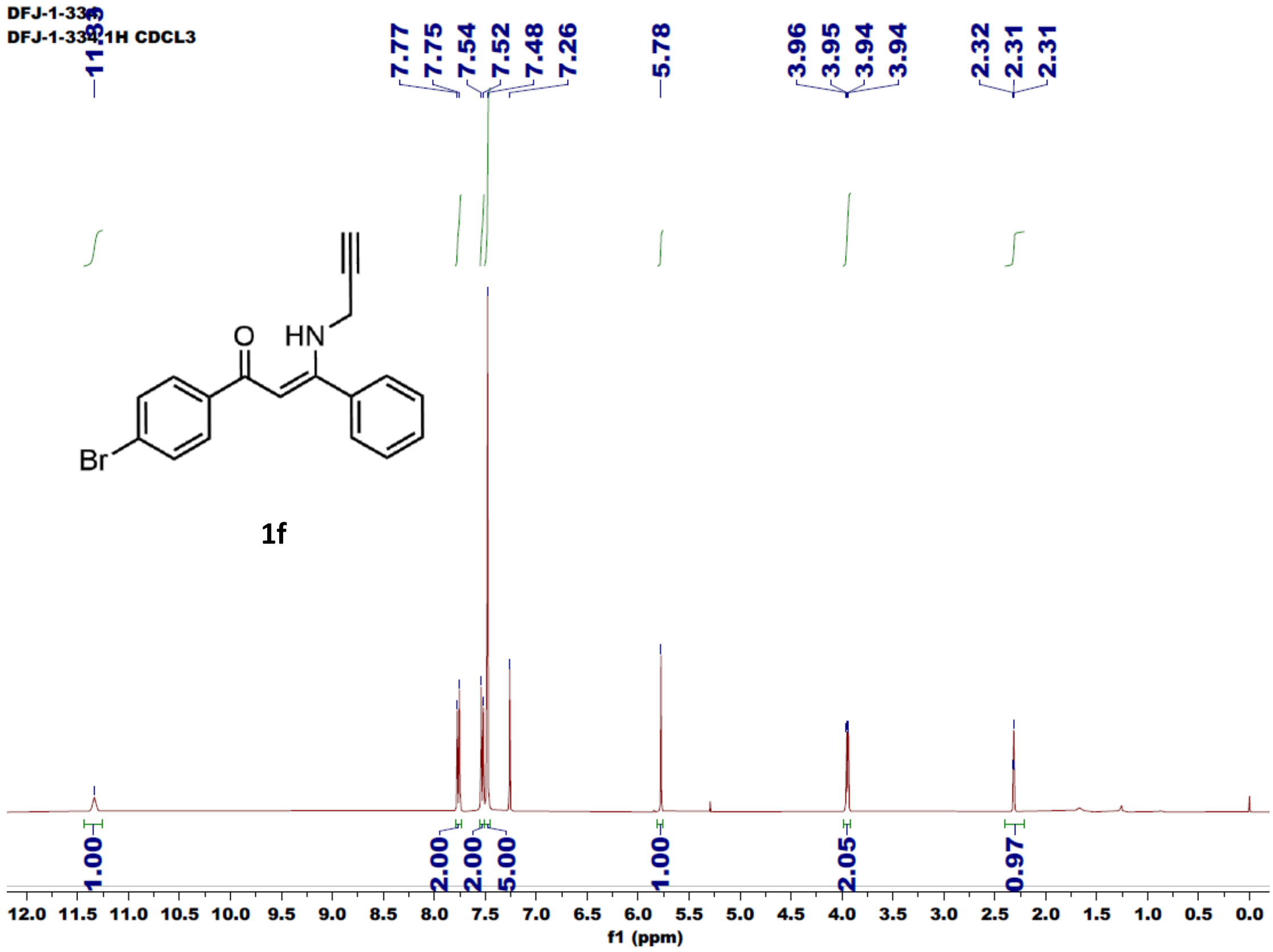

^{13}C NMR spectrum of compound 1f (101 MHz, $CDCl_3$)

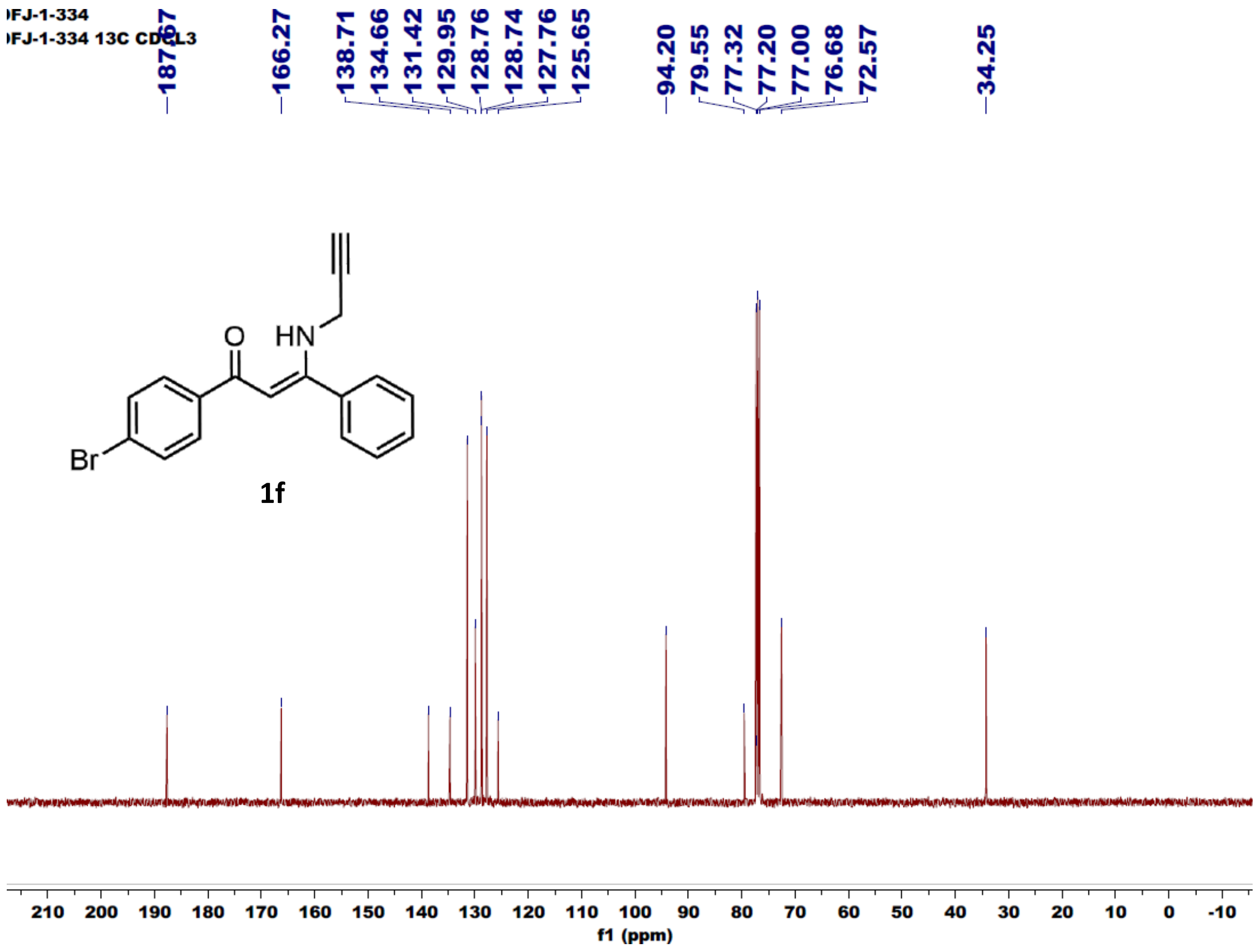

^{1}H NMR spectrum of compound 1g (400 MHz, $CDCl_3$)

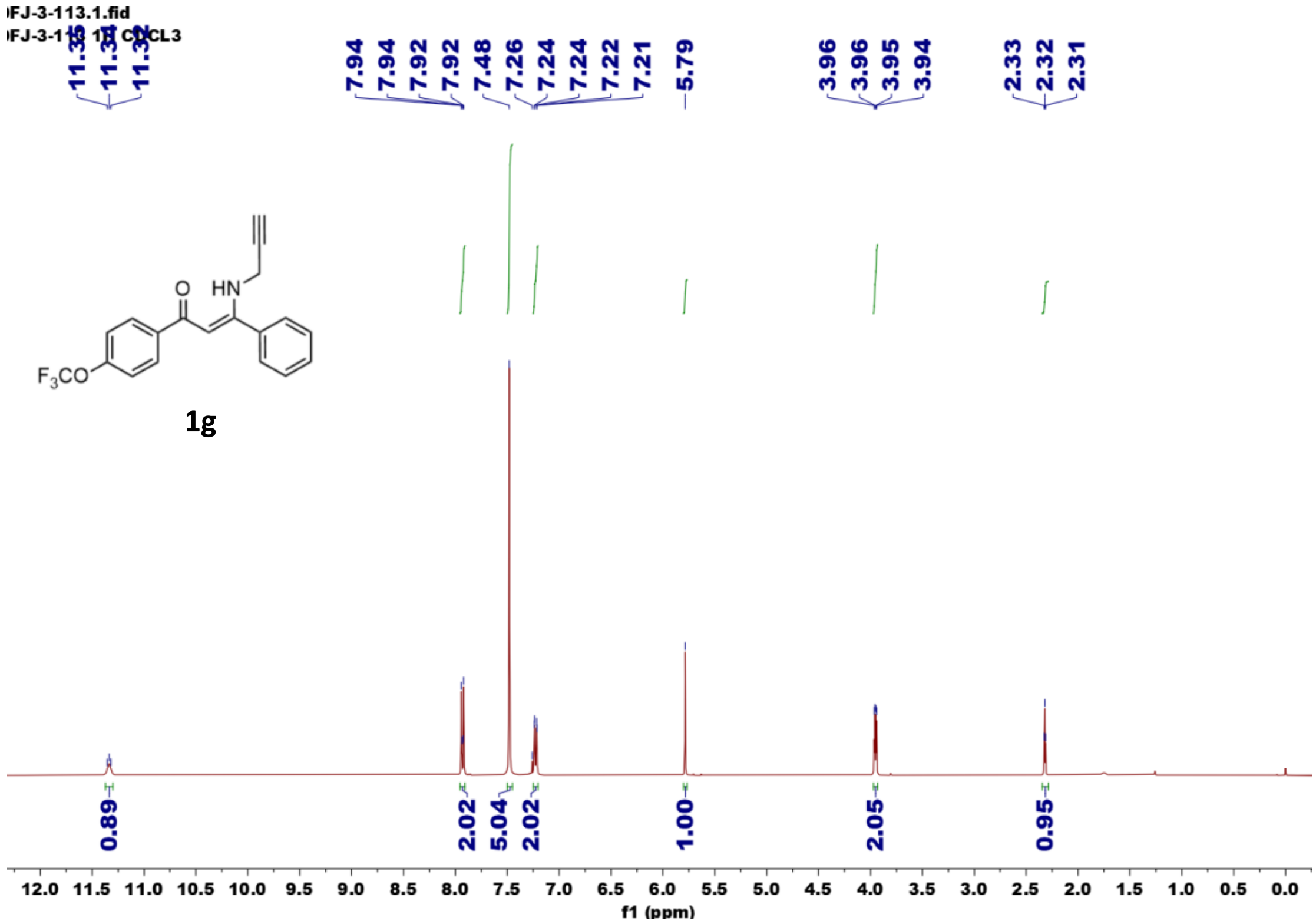

^{13}C NMR spectrum of compound 1g (101 MHz, $CDCl_3$)

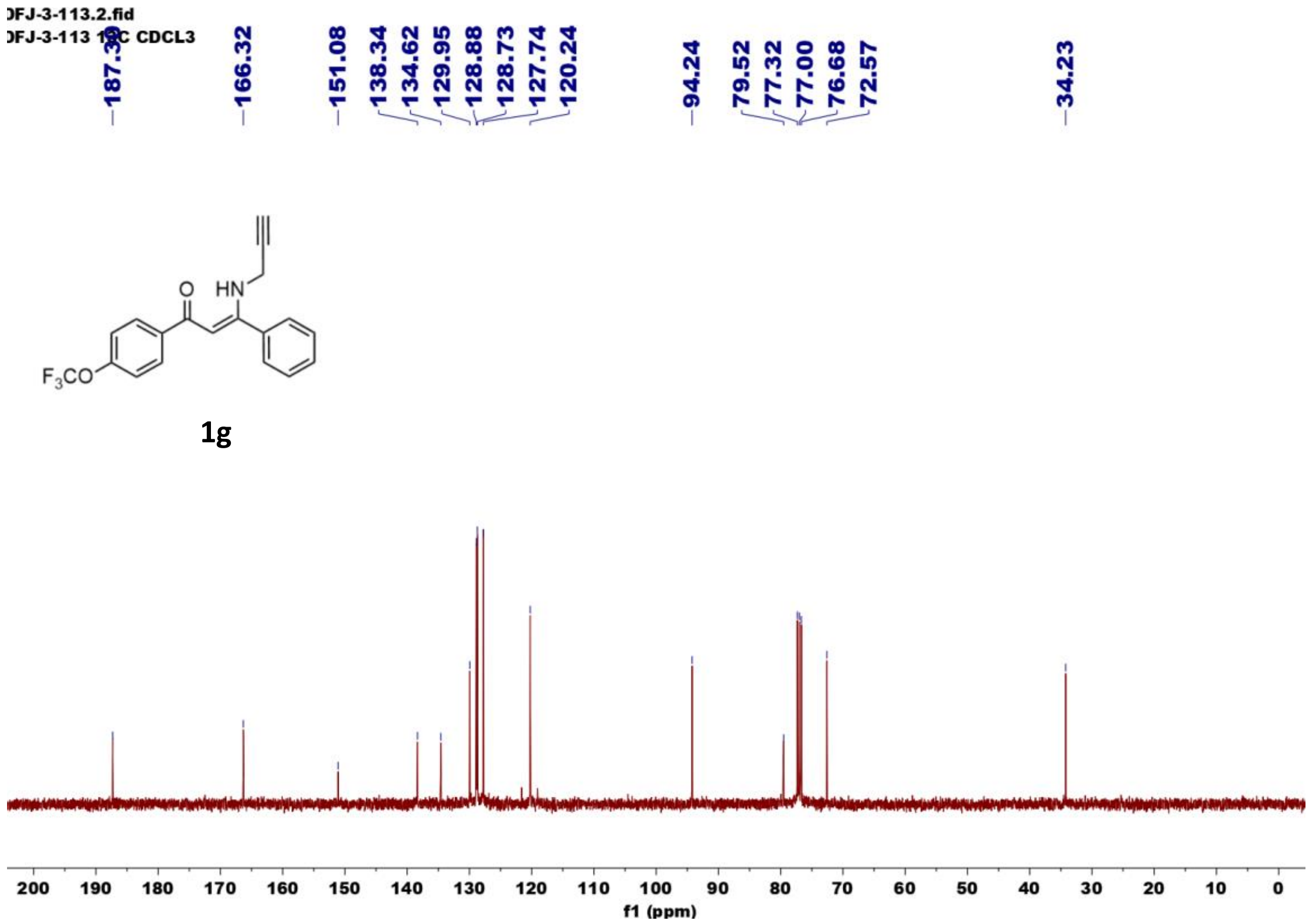

^{1}H NMR spectrum of compound 1h (400 MHz, $CDCl_3$)

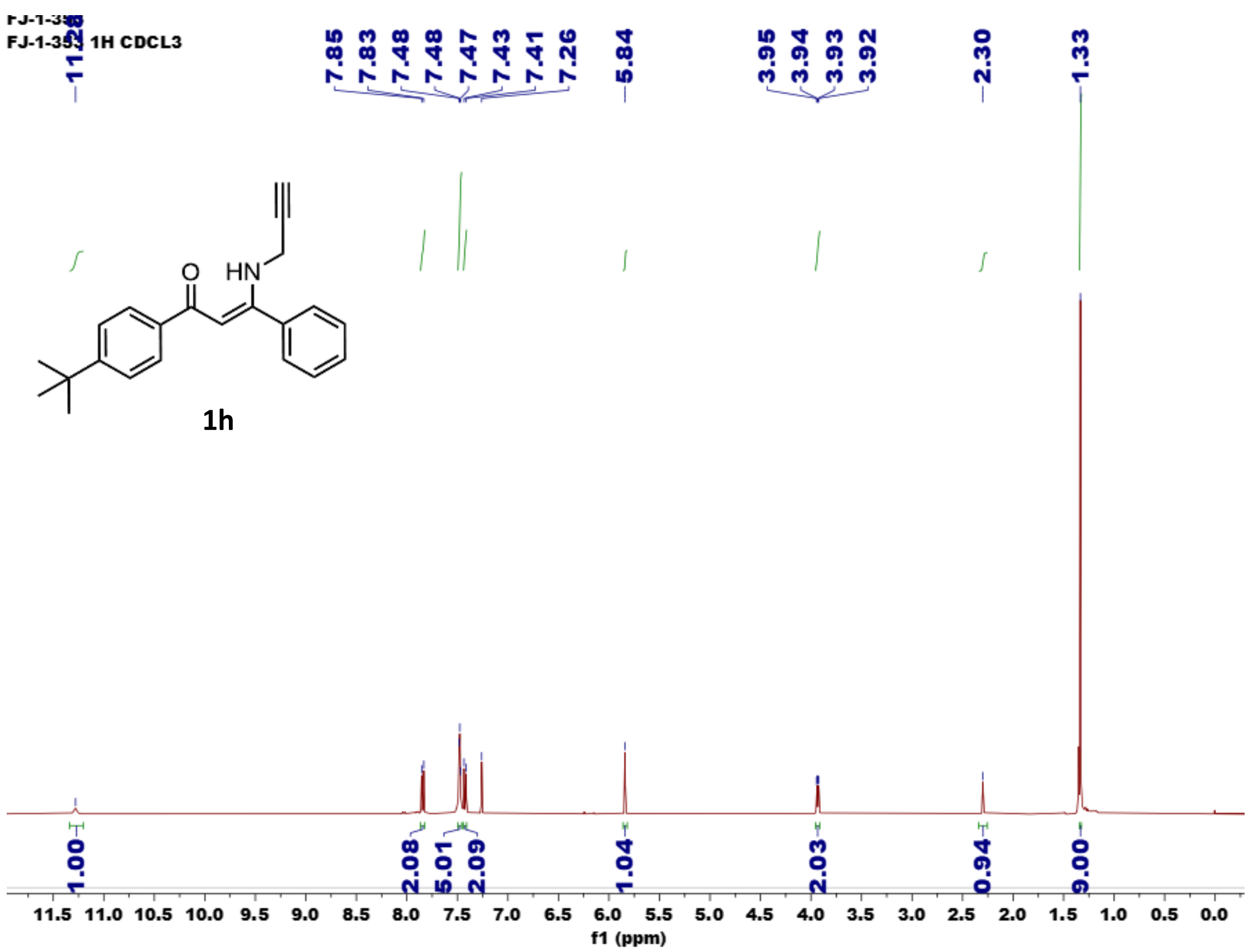

^{13}C NMR spectrum of compound 1h (101 MHz, $CDCl_3$)

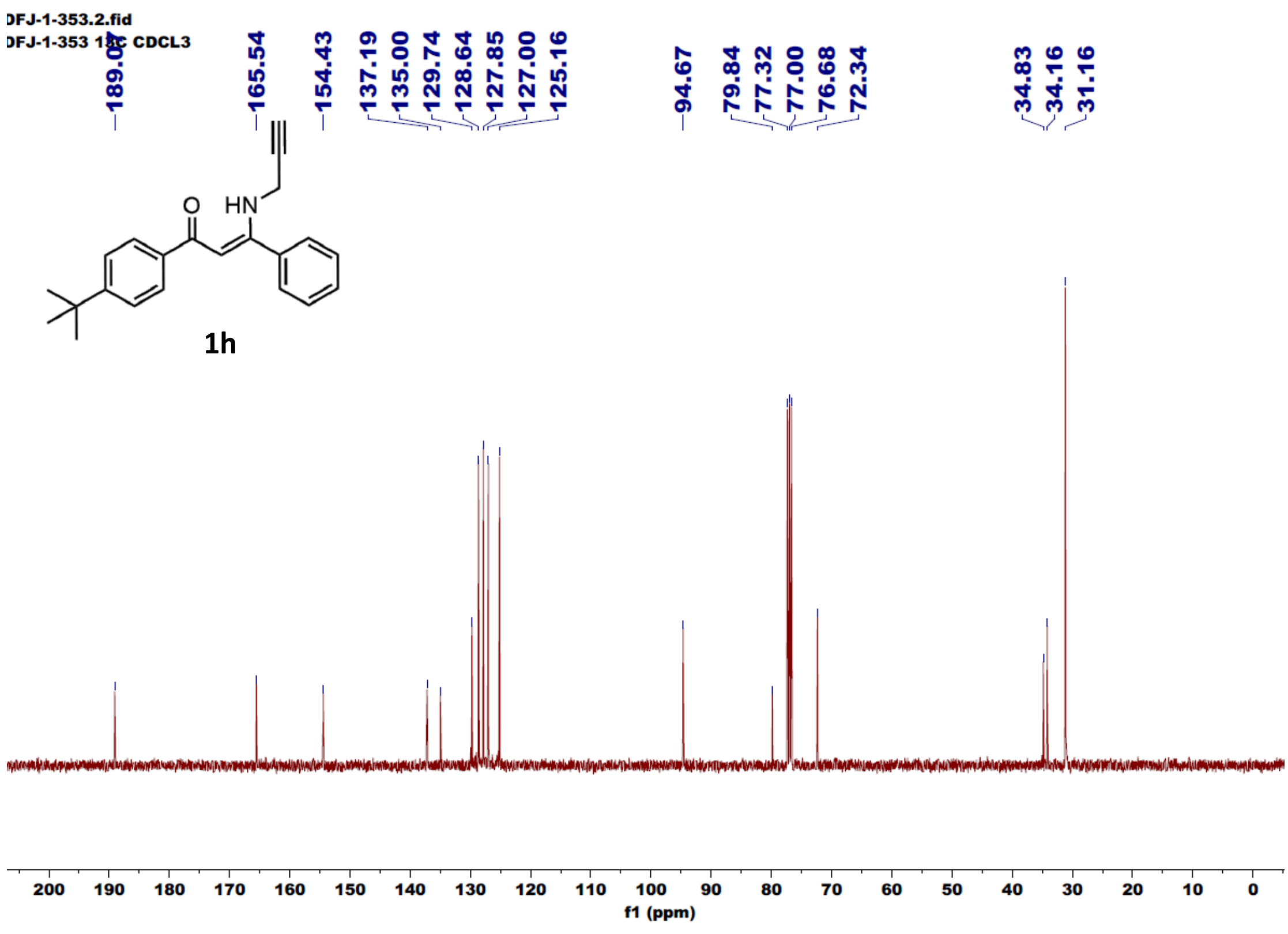

^{1}H NMR spectrum of compound 1j (400 MHz, $CDCl_3$)

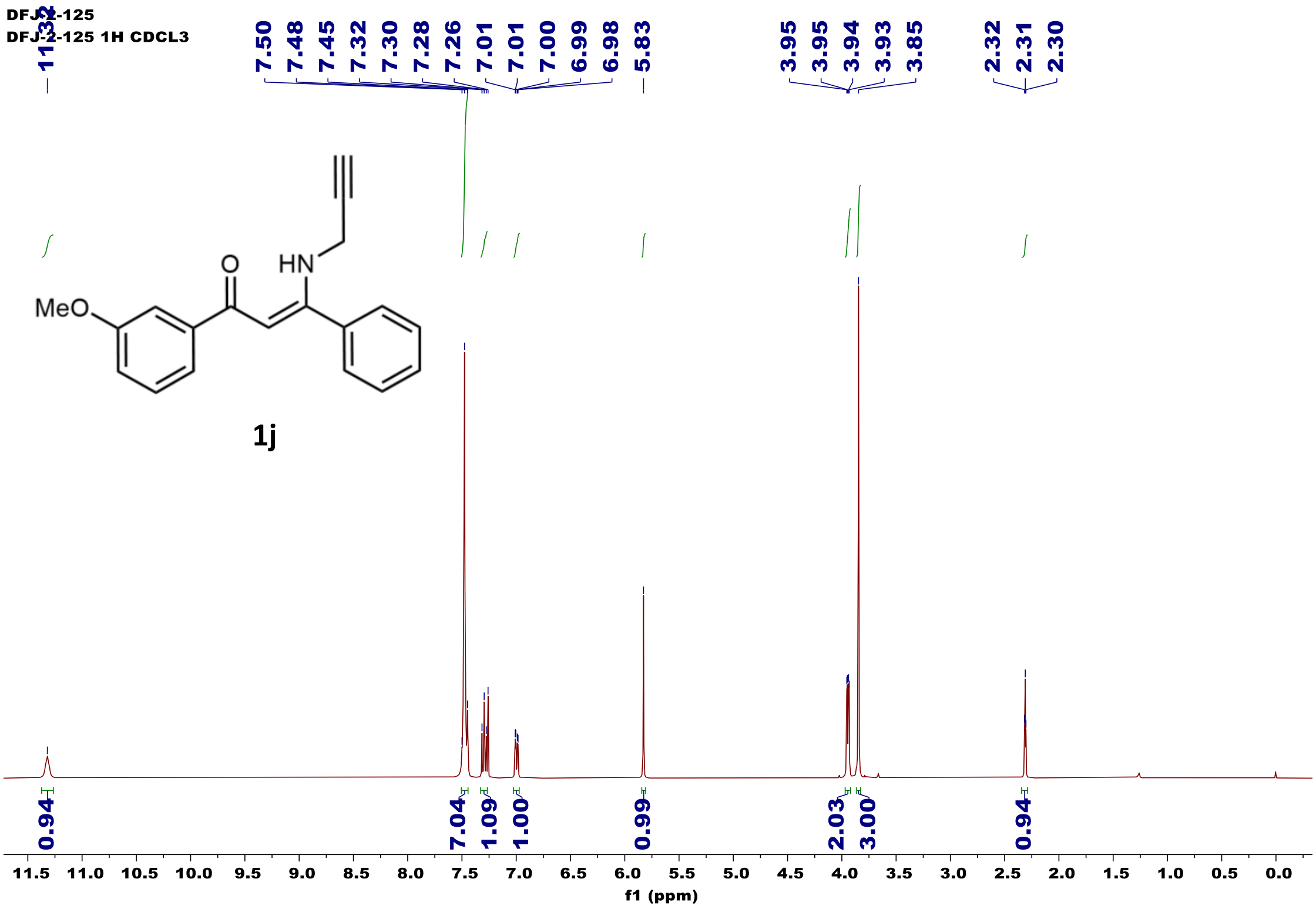

^{13}C NMR spectrum of compound 1j (101 MHz, $CDCl_3$)

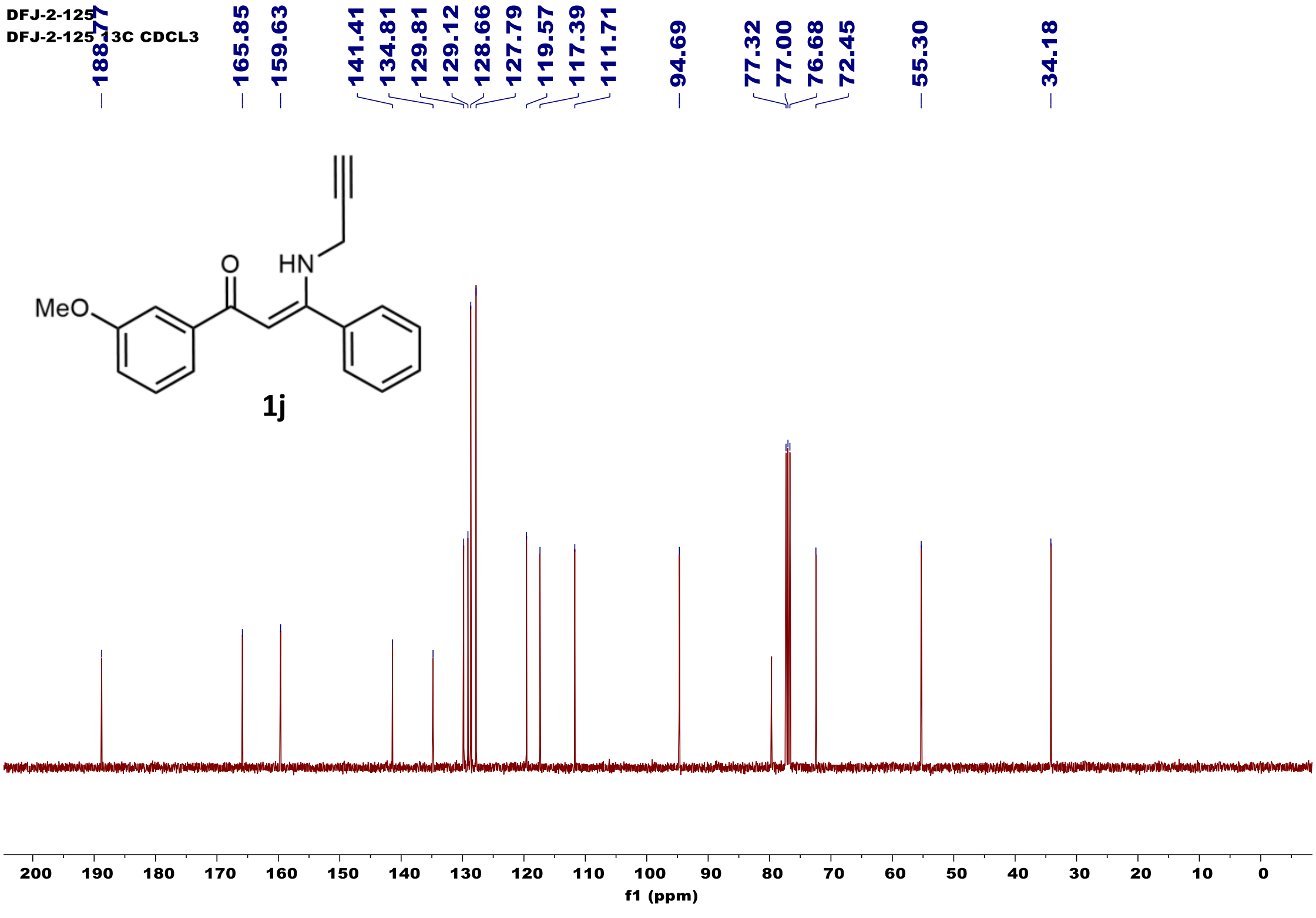

^{1}H NMR spectrum of compound 1k (400 MHz, $CDCl_3$)

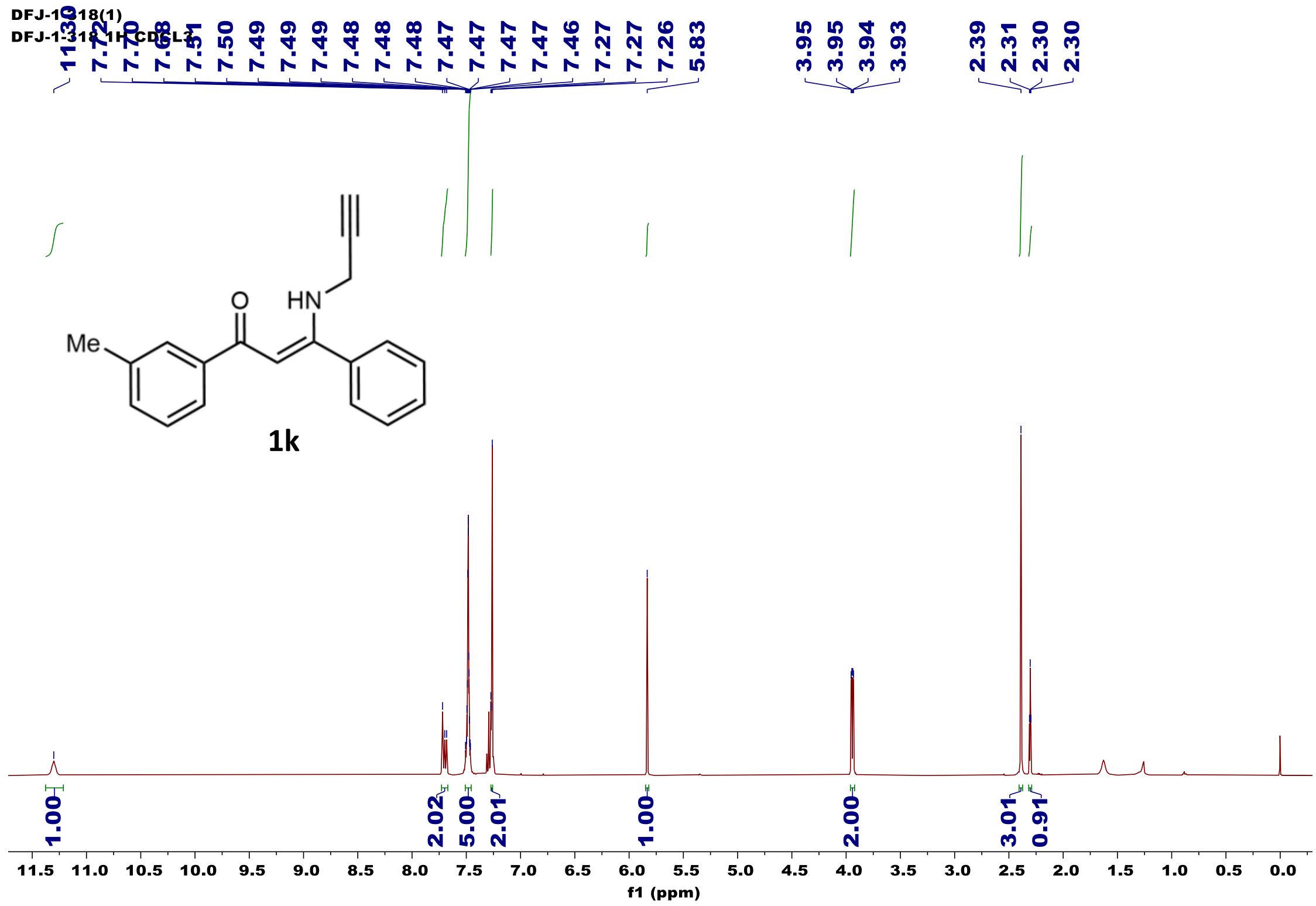

^{13}C NMR spectrum of compound 1k (101 MHz, $CDCl_3$)

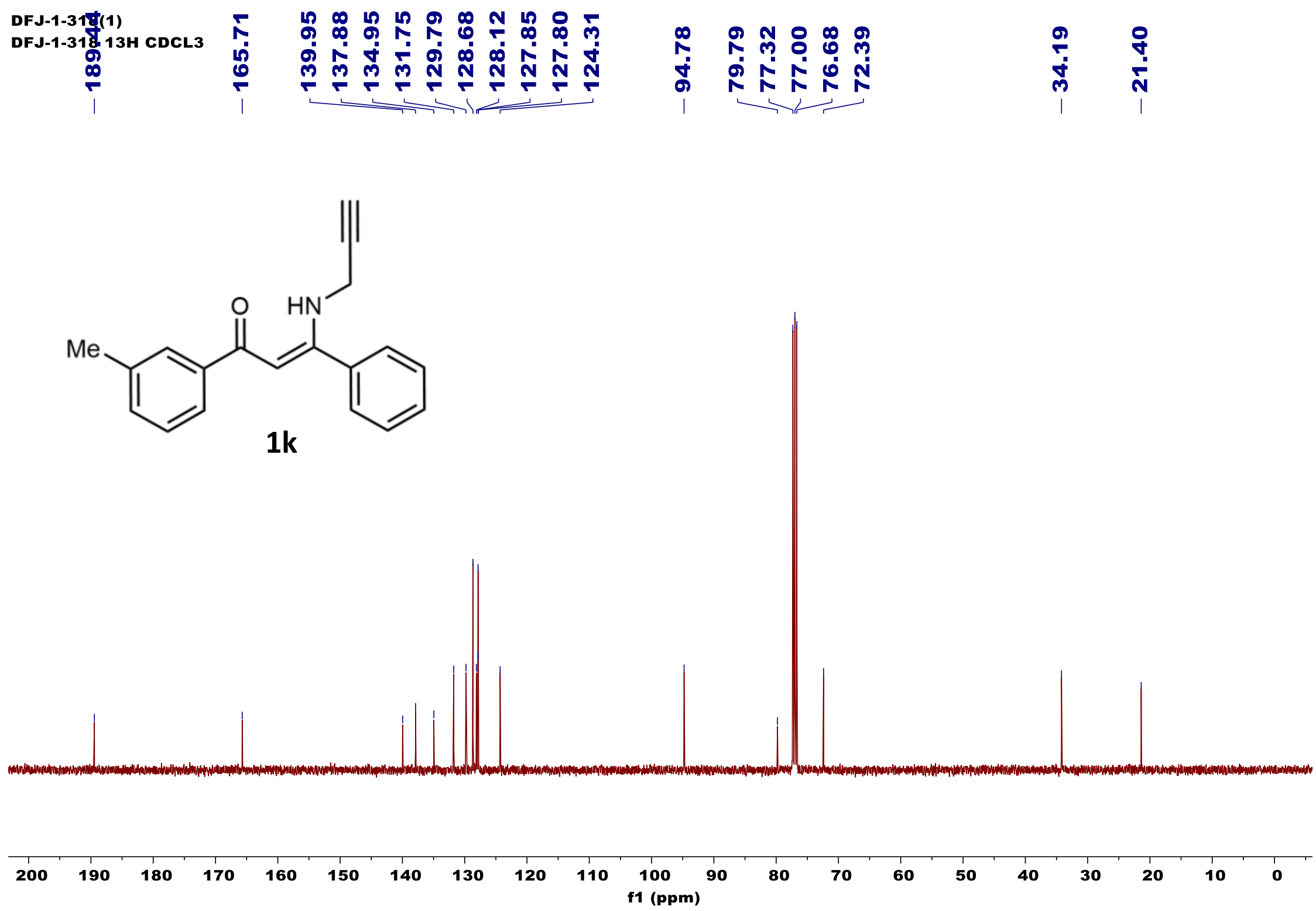

^{1}H NMR spectrum of compound 1l (400 MHz, $CDCl_3$)

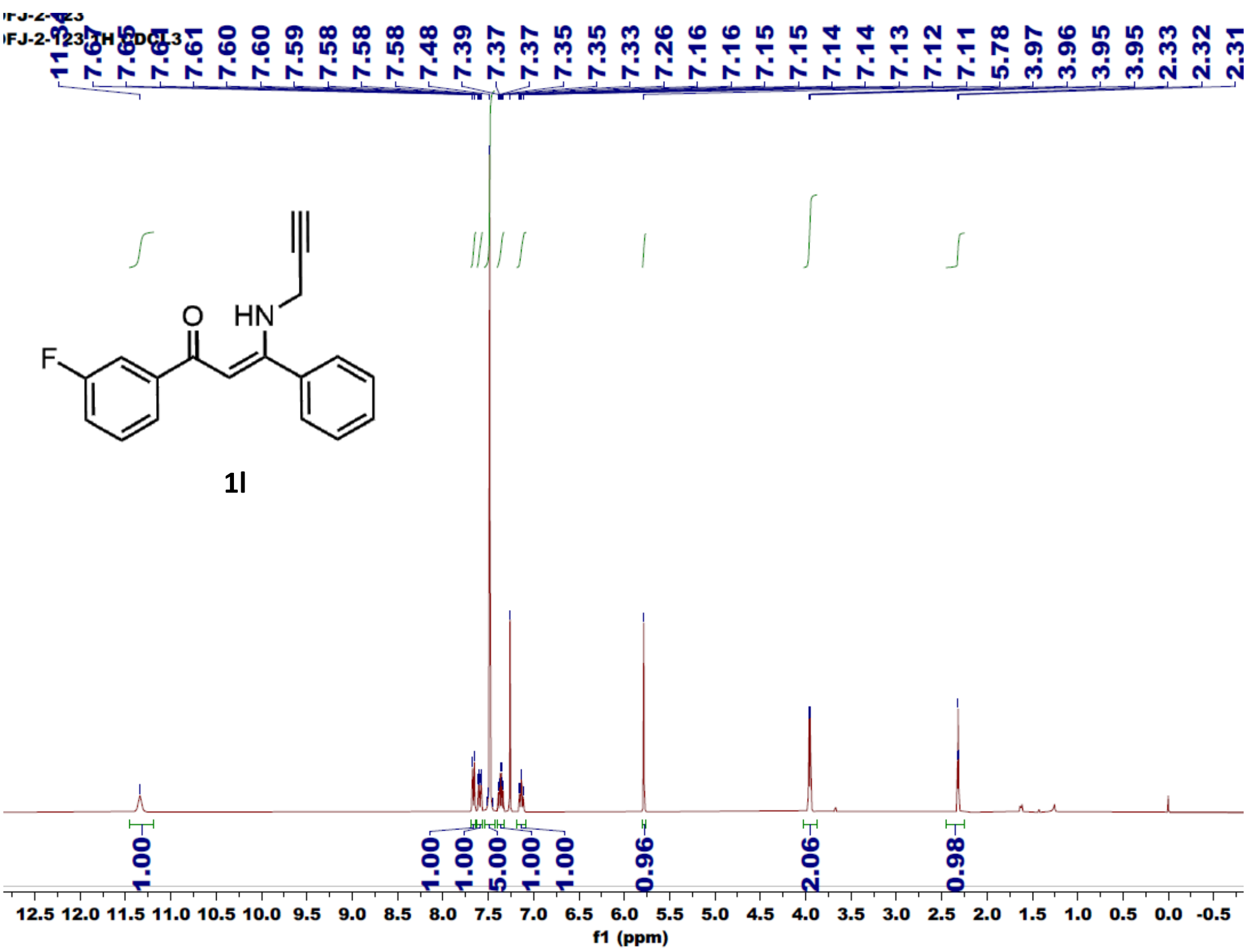

^{13}C NMR spectrum of compound 1l (101 MHz, $CDCl_3$)

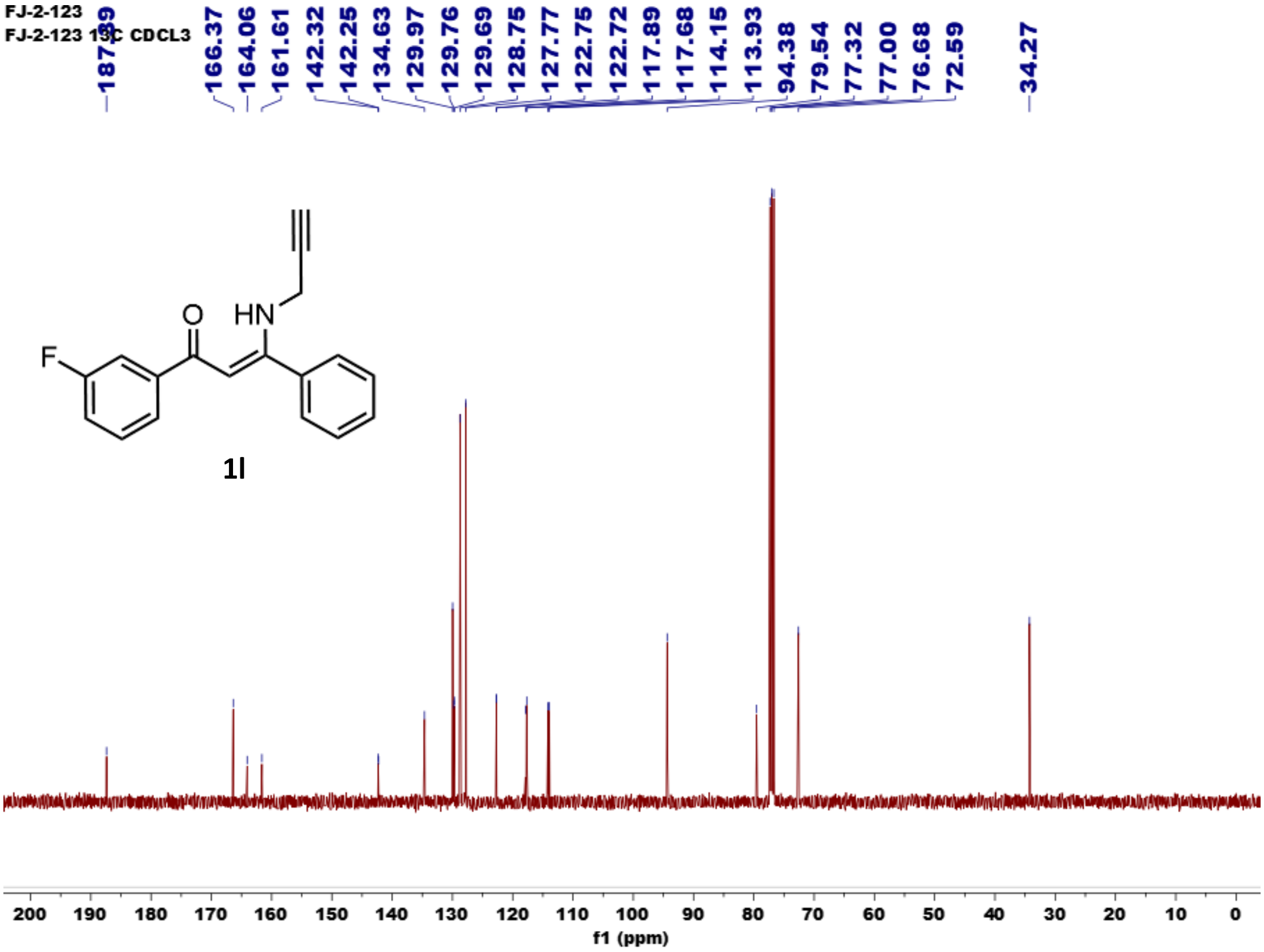

^{1}H NMR spectrum of compound 1m (400 MHz, $CDCl_3$)

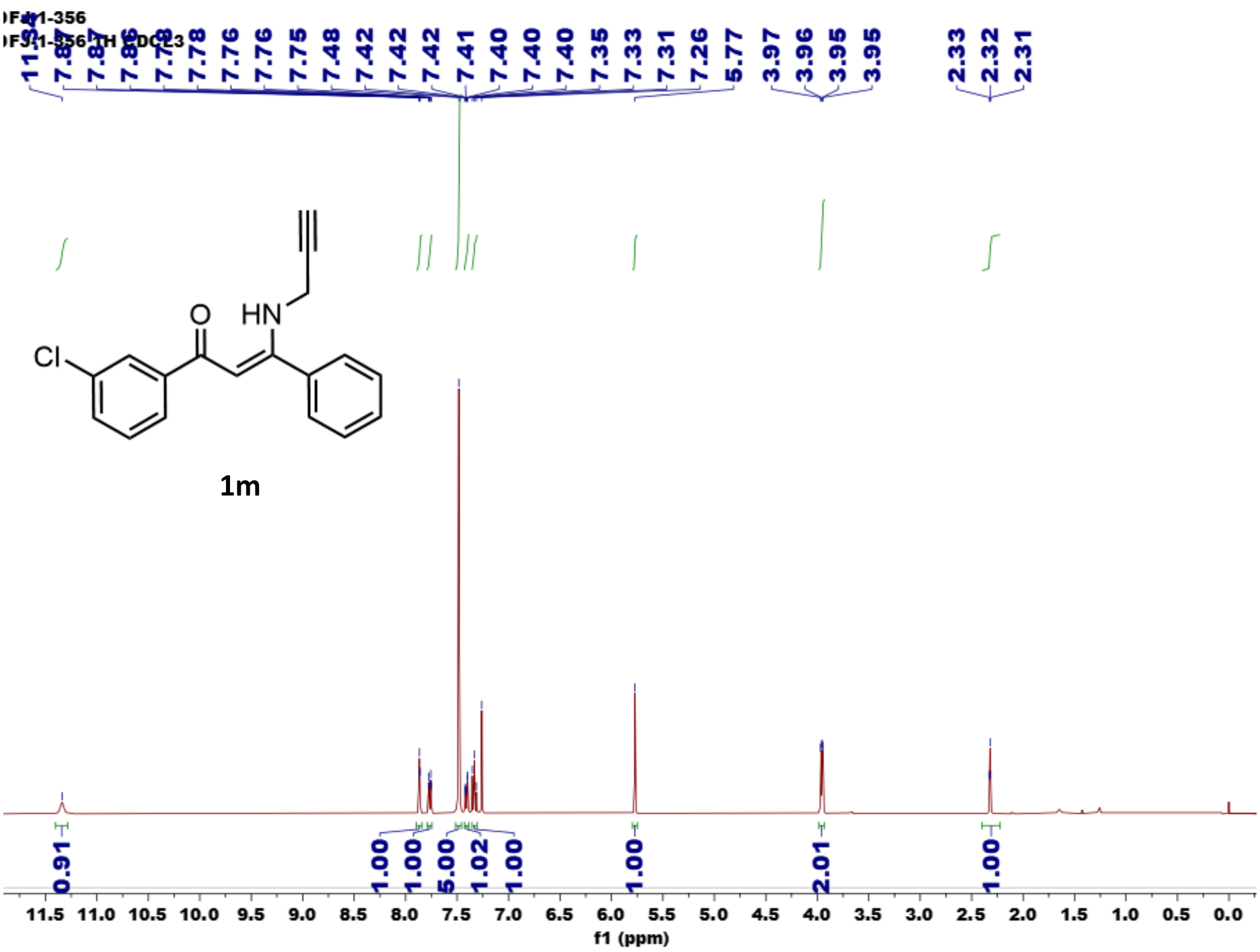

^{13}C NMR spectrum of compound 1m (101 MHz, $CDCl_3$)

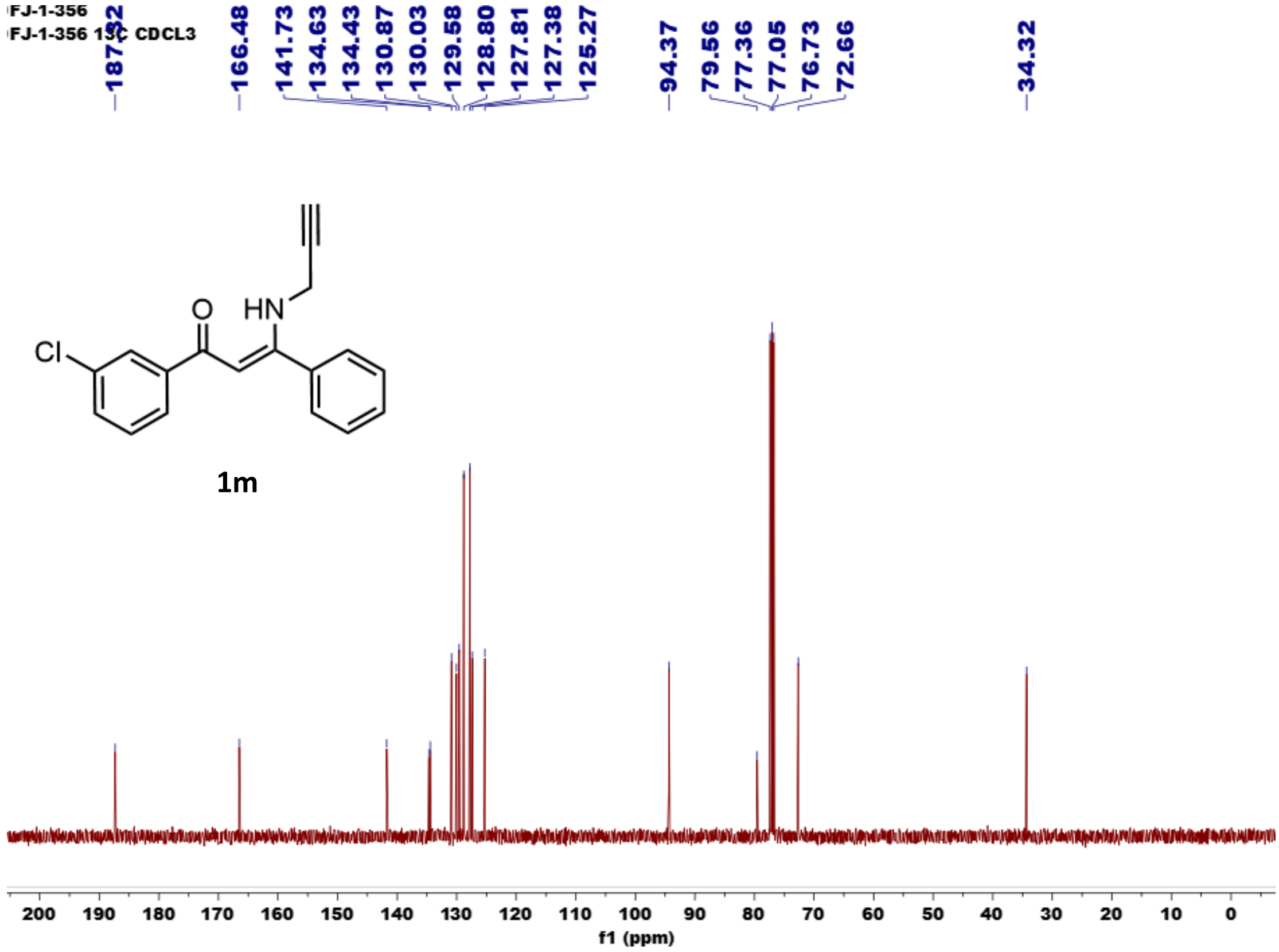

^{1}H NMR spectrum of compound 1n (400 MHz, $CDCl_3$)

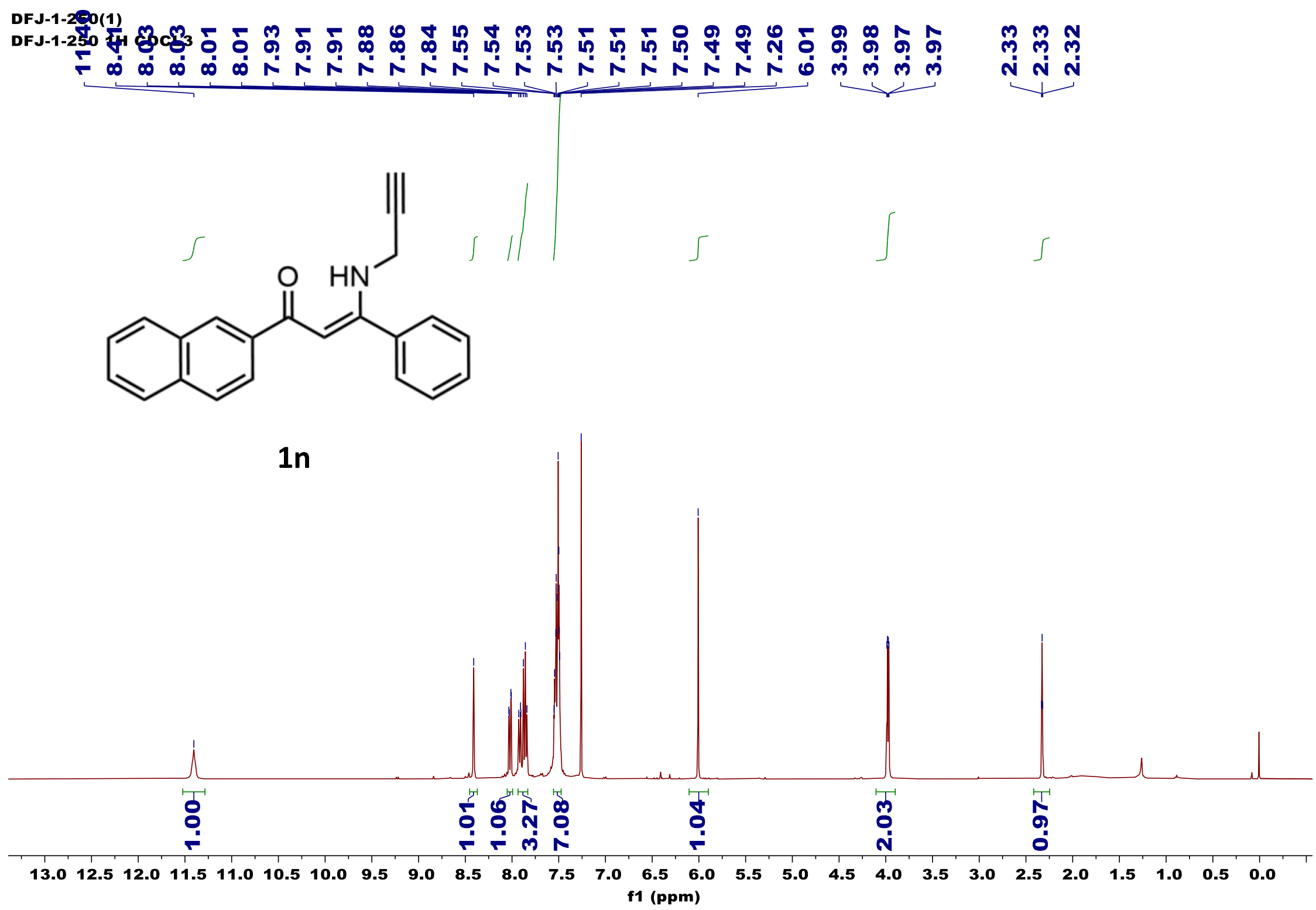

^{13}C NMR spectrum of compound 1n (101 MHz, $CDCl_3$)

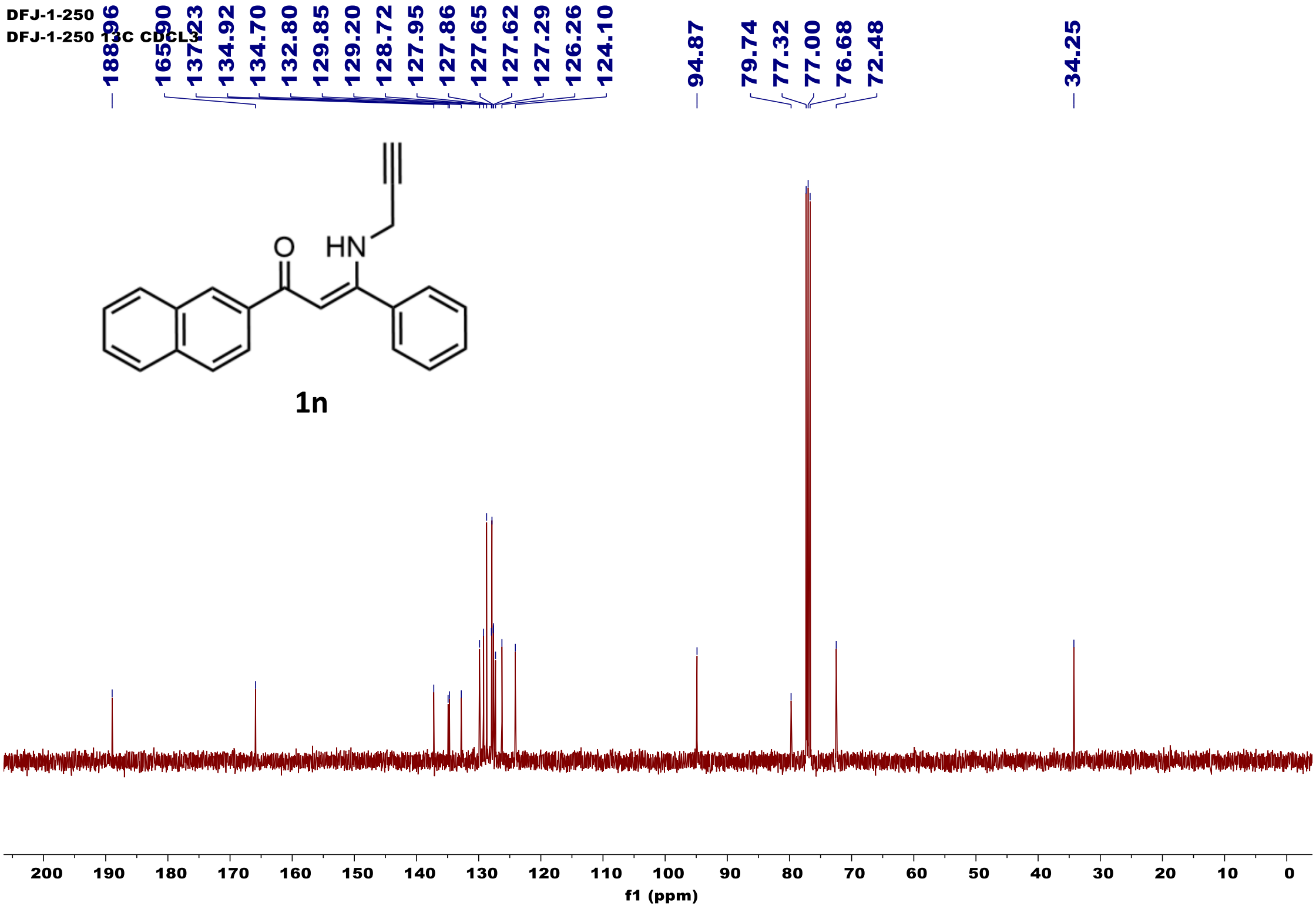

^{1}H NMR spectrum of compound 1o (400 MHz, $CDCl_3$)

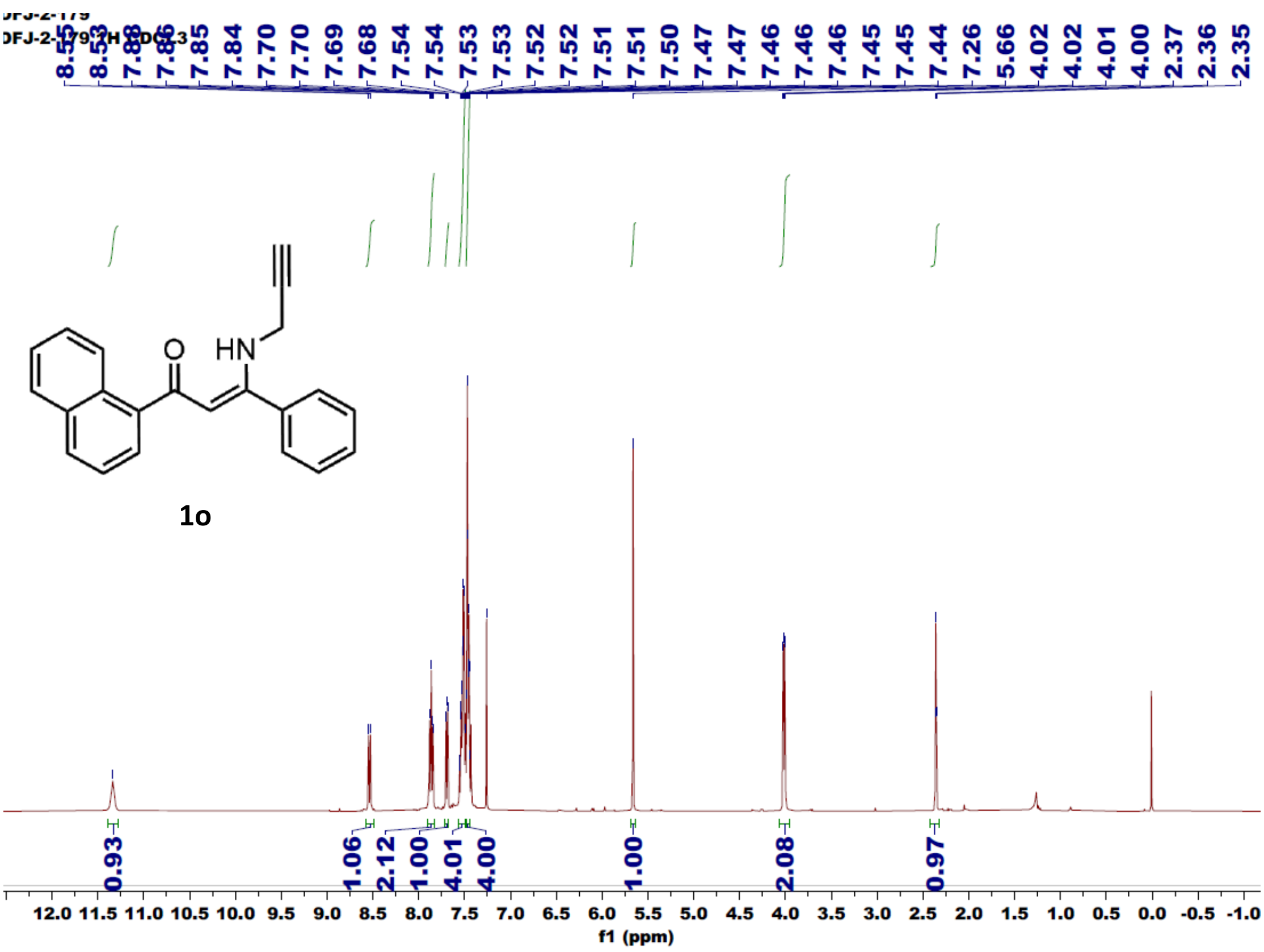

^{13}C NMR spectrum of compound 1o (101 MHz, $CDCl_3$)

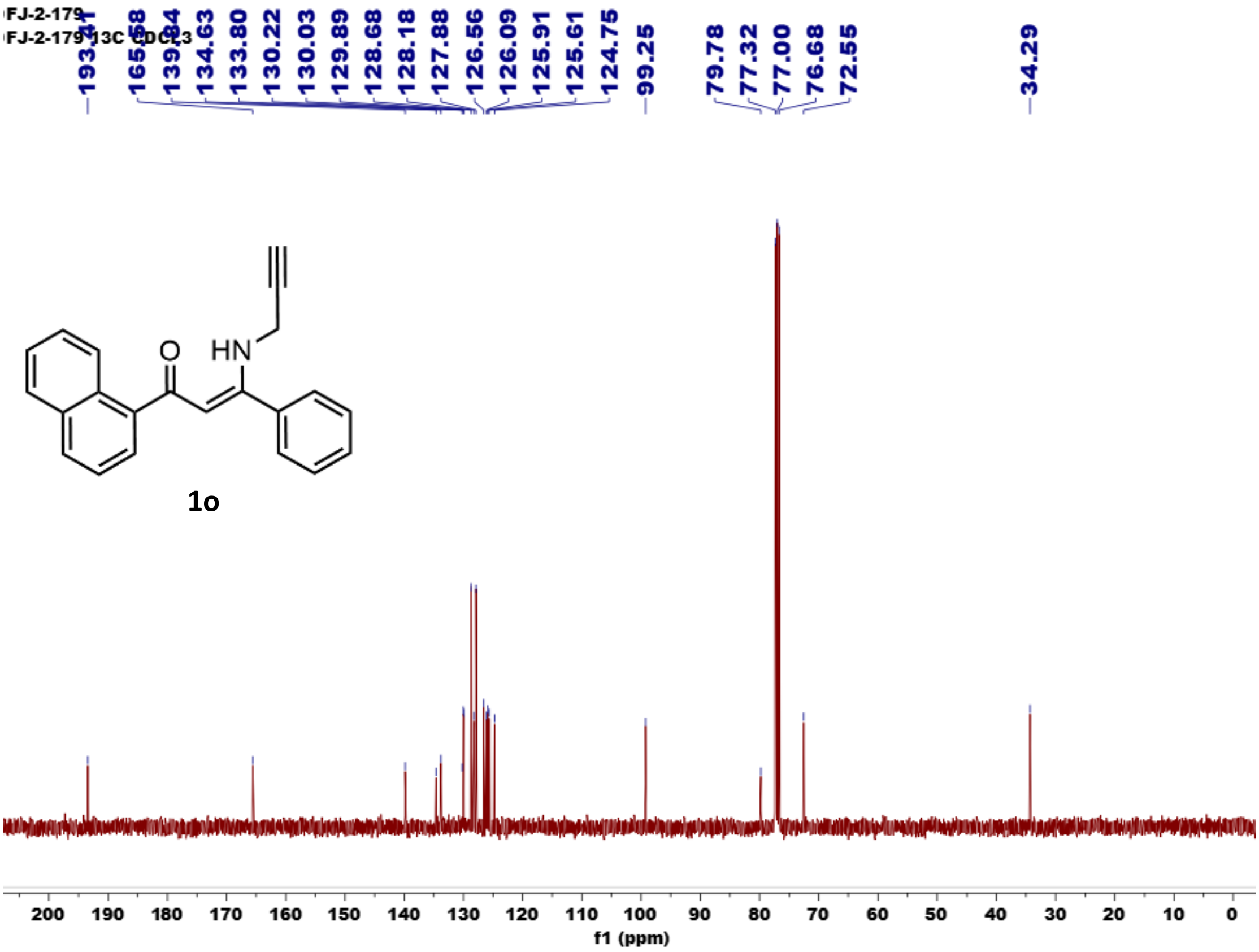

^{1}H NMR spectrum of compound 1p (400 MHz, $CDCl_3$)

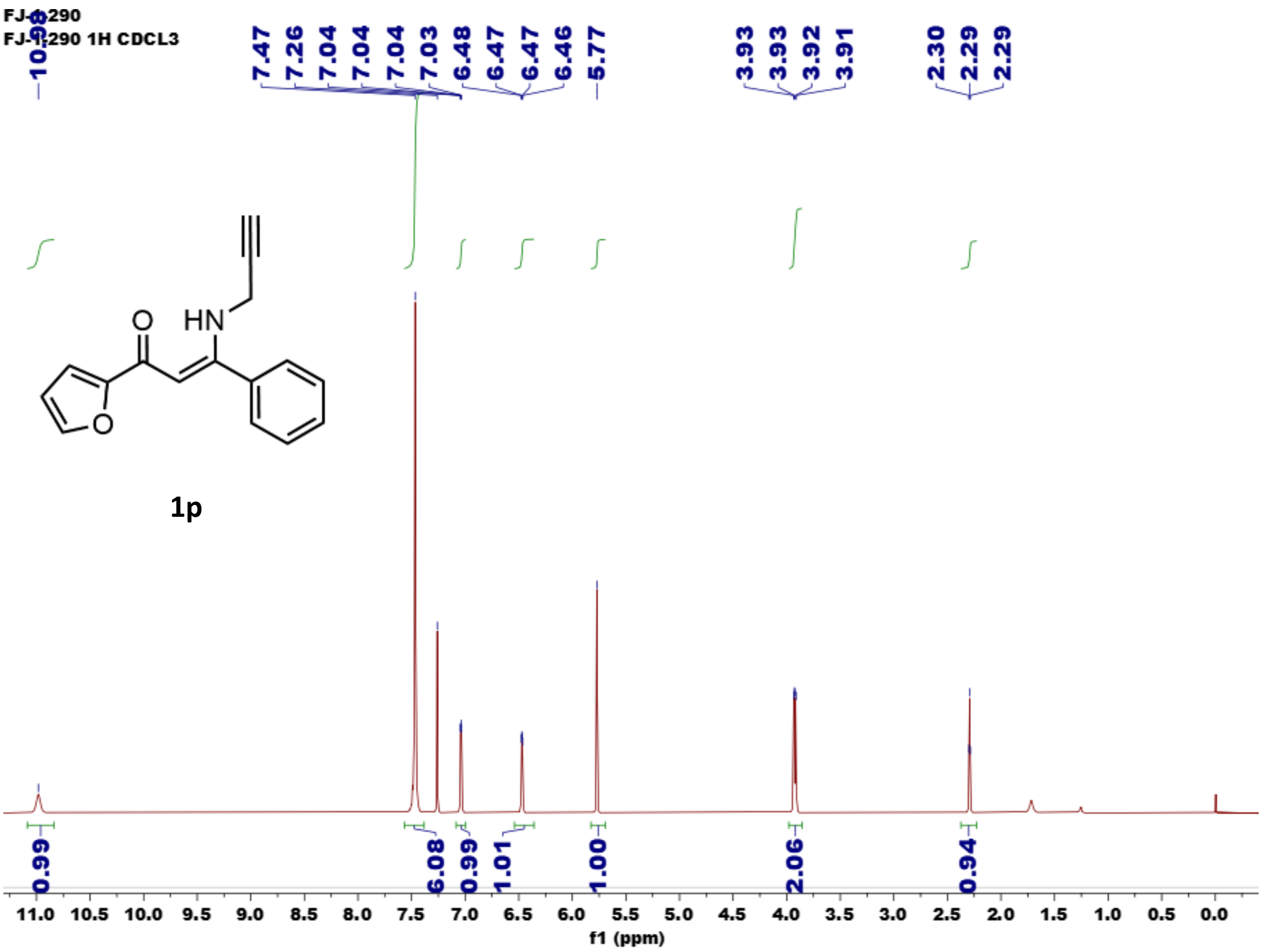

^{13}C NMR spectrum of compound 1p (101 MHz, $CDCl_3$)

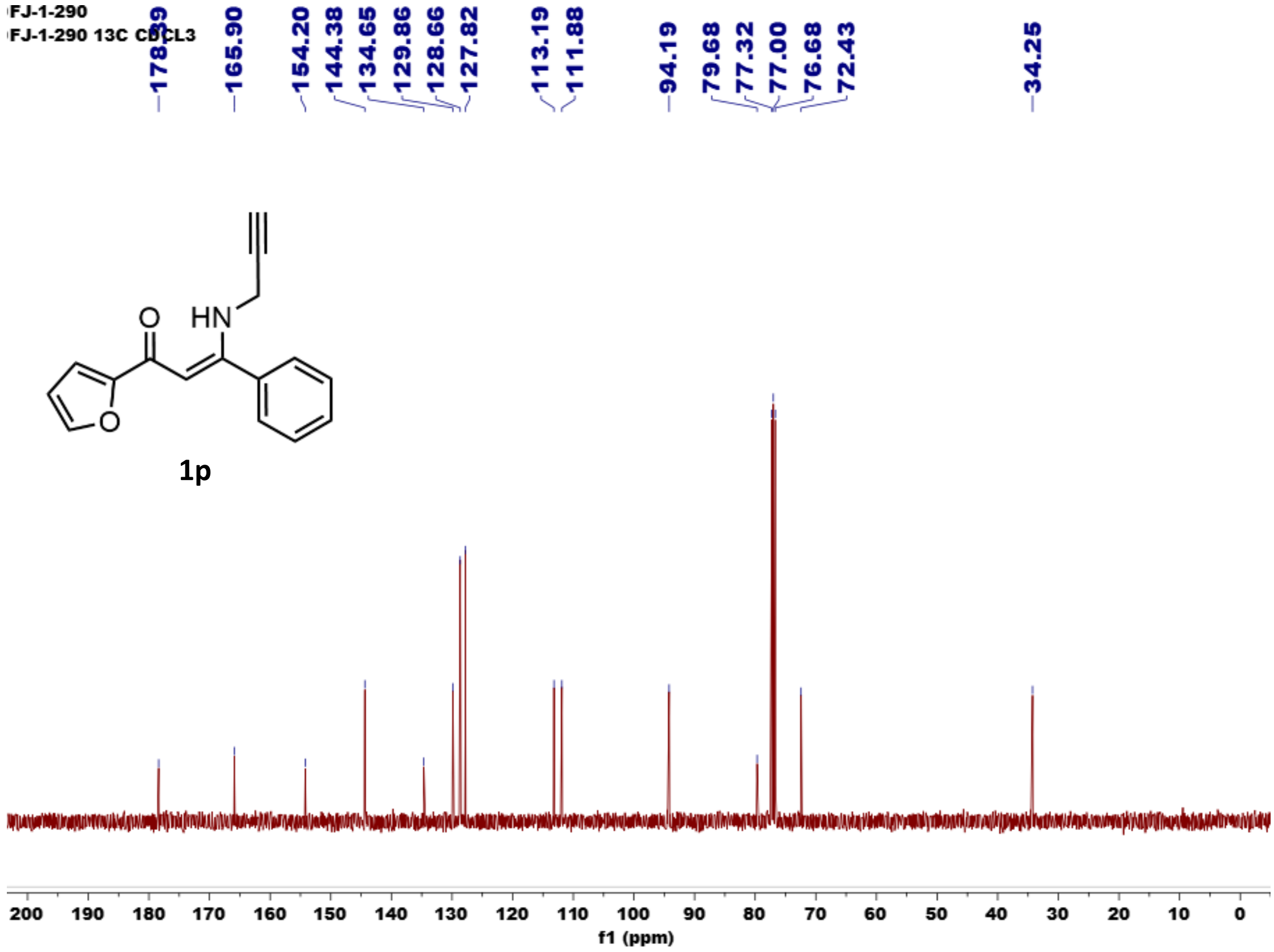

^{1}H NMR spectrum of compound 1q (400 MHz, $CDCl_3$)

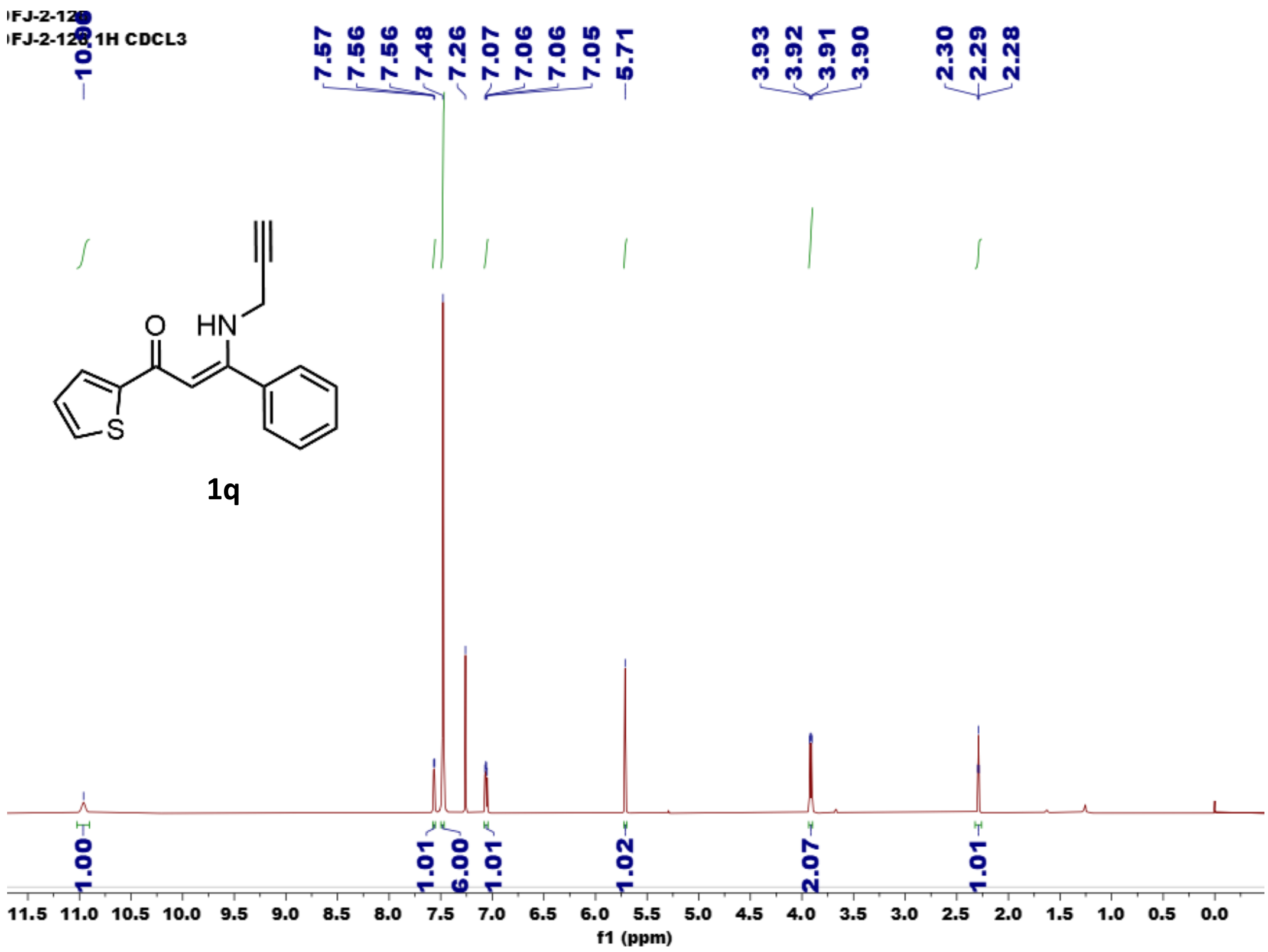

^{13}C NMR spectrum of compound 1q (101 MHz, $CDCl_3$)

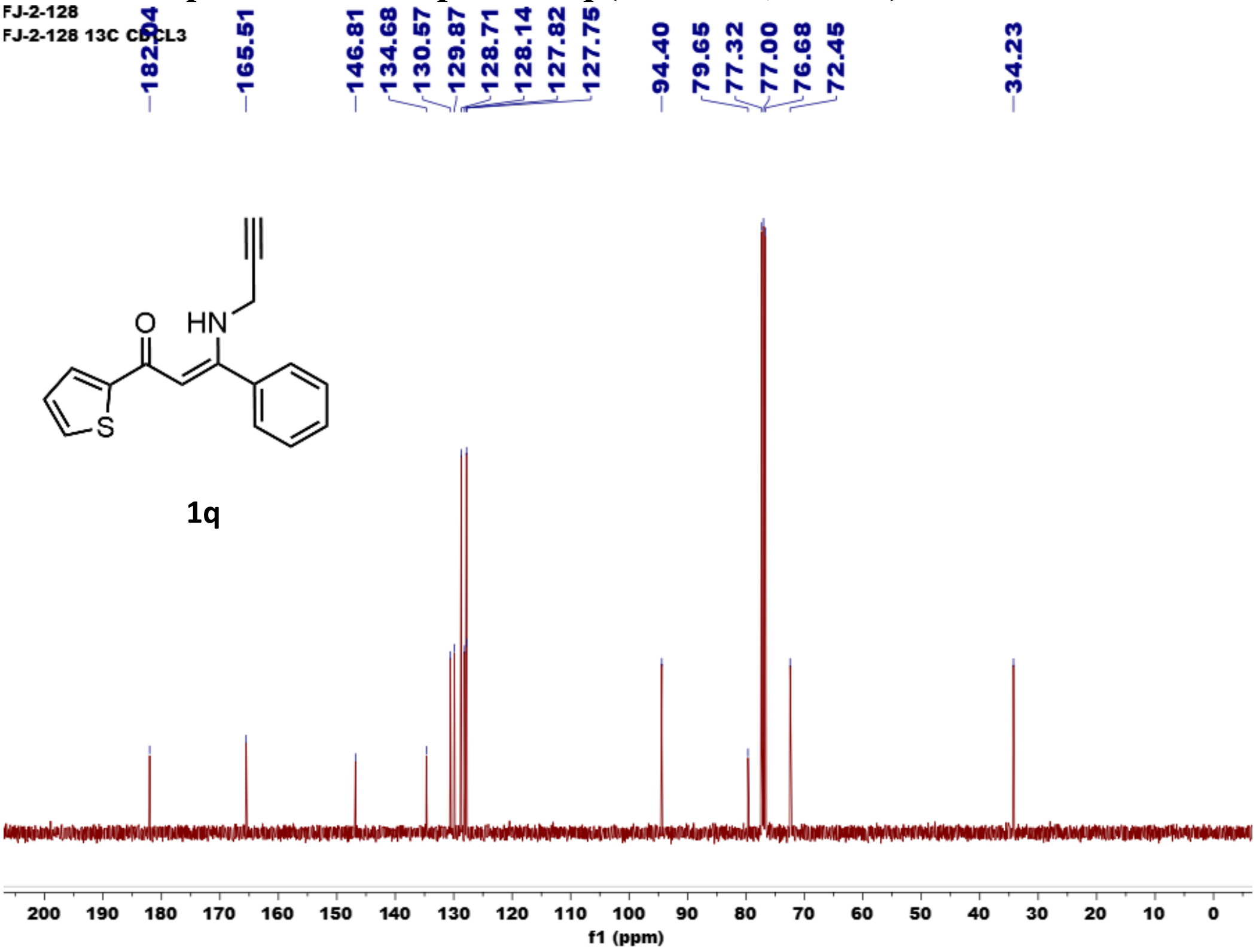

^{1}H NMR spectrum of compound 1r (400 MHz, $CDCl_3$)

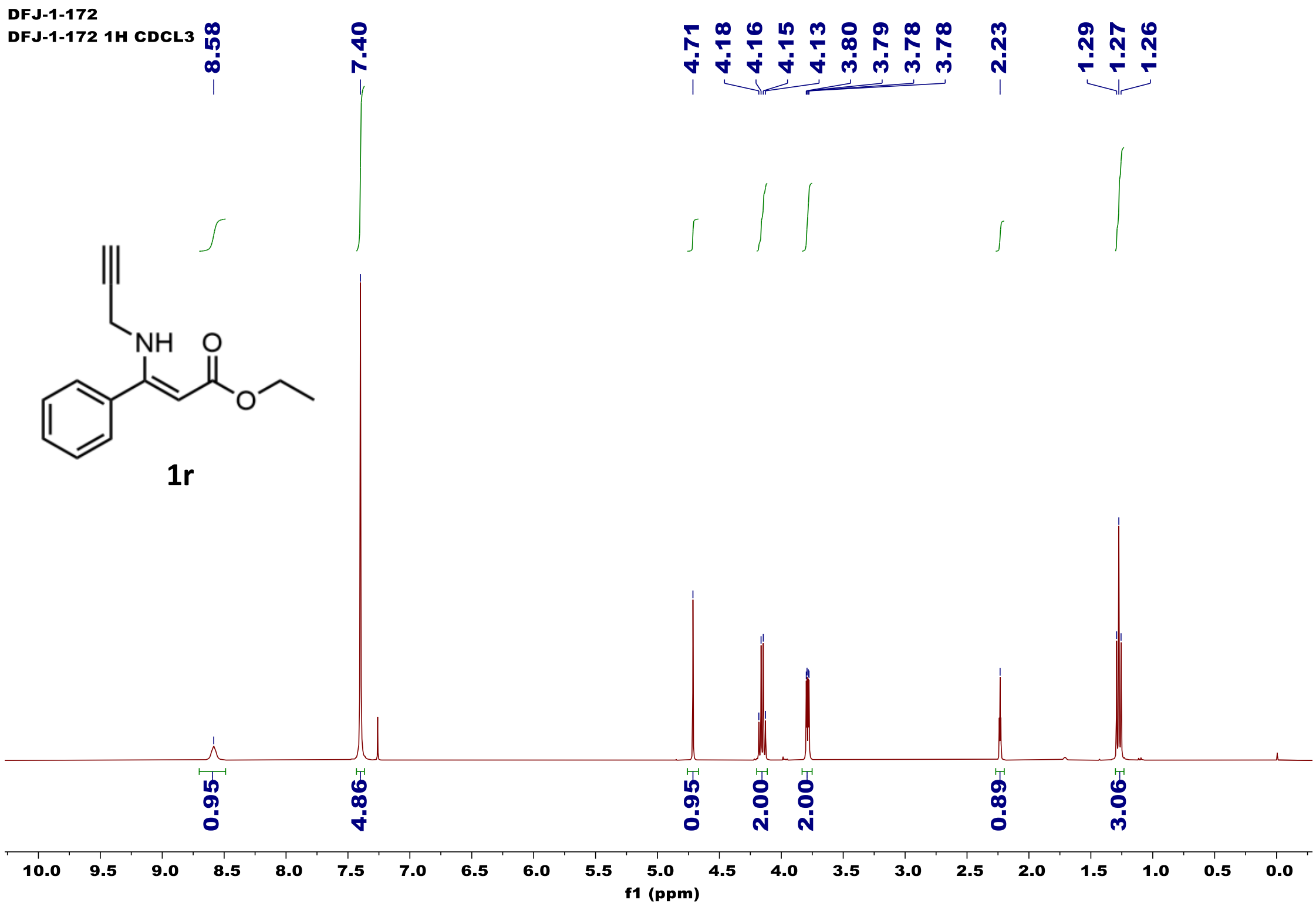

^{13}C NMR spectrum of compound 1r (101 MHz, $CDCl_3$)

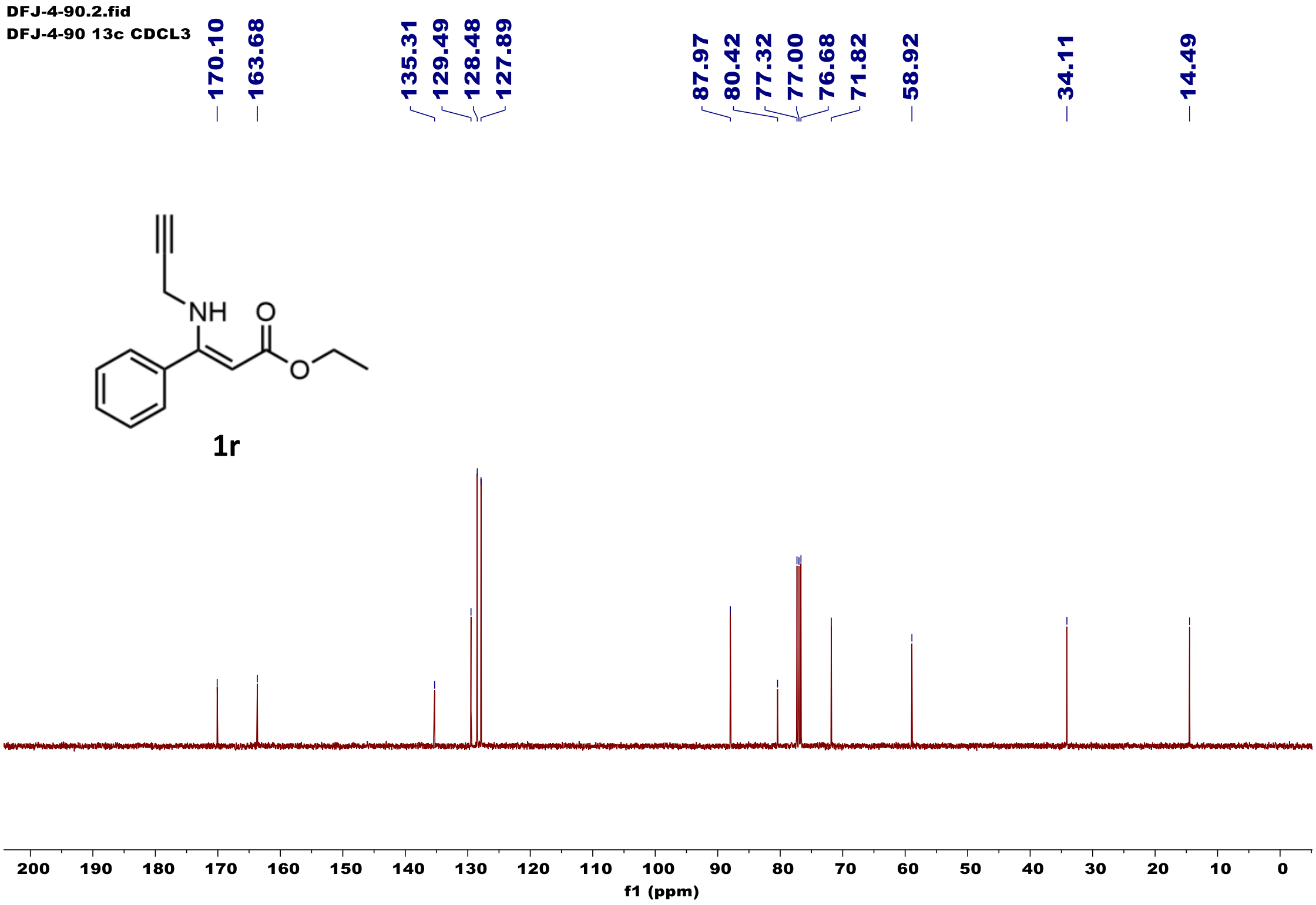

^{1}H NMR spectrum of compound 1s (400 MHz, $CDCl_3$)

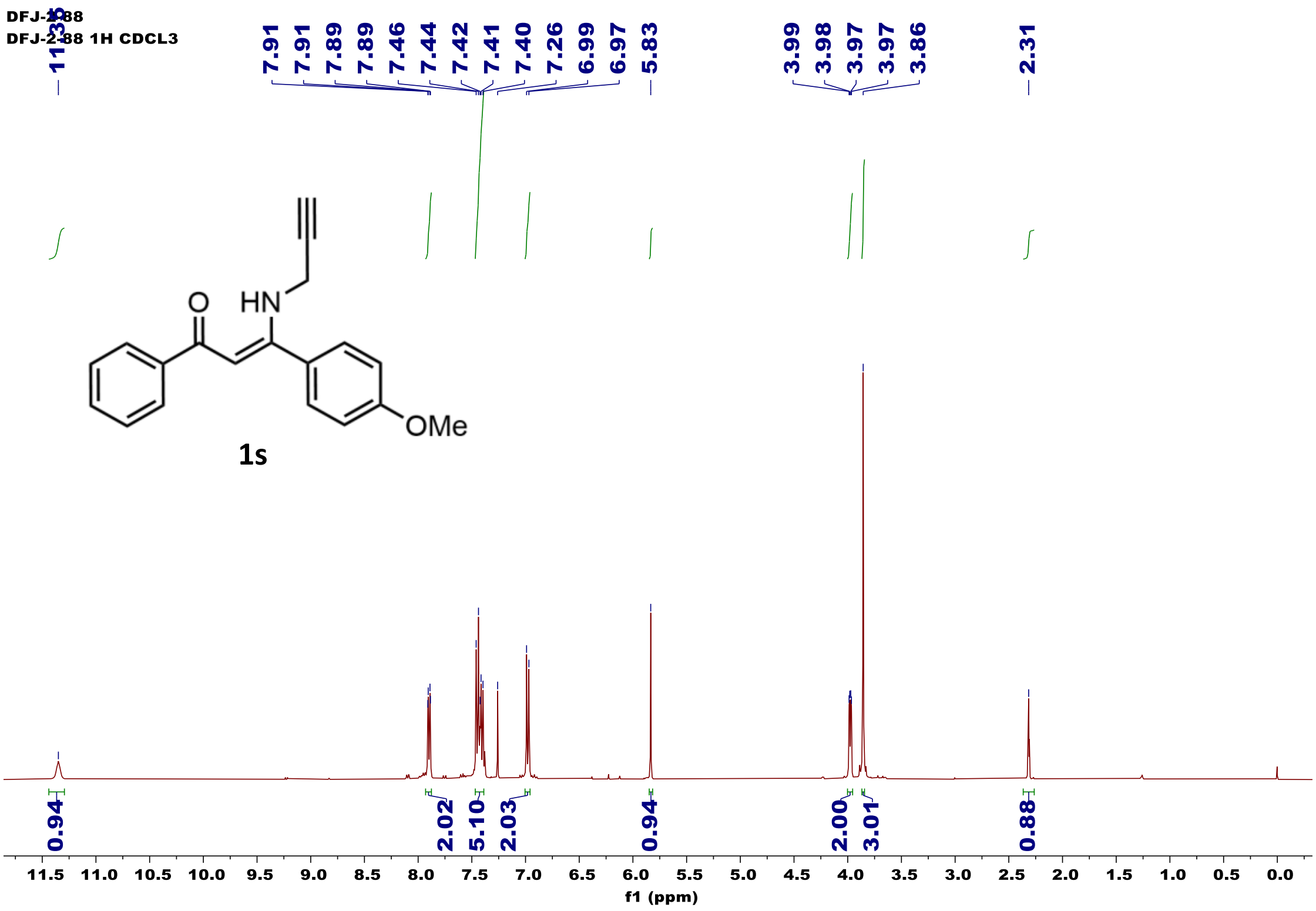

^{13}C NMR spectrum of compound 1s (101 MHz, $CDCl_3$)

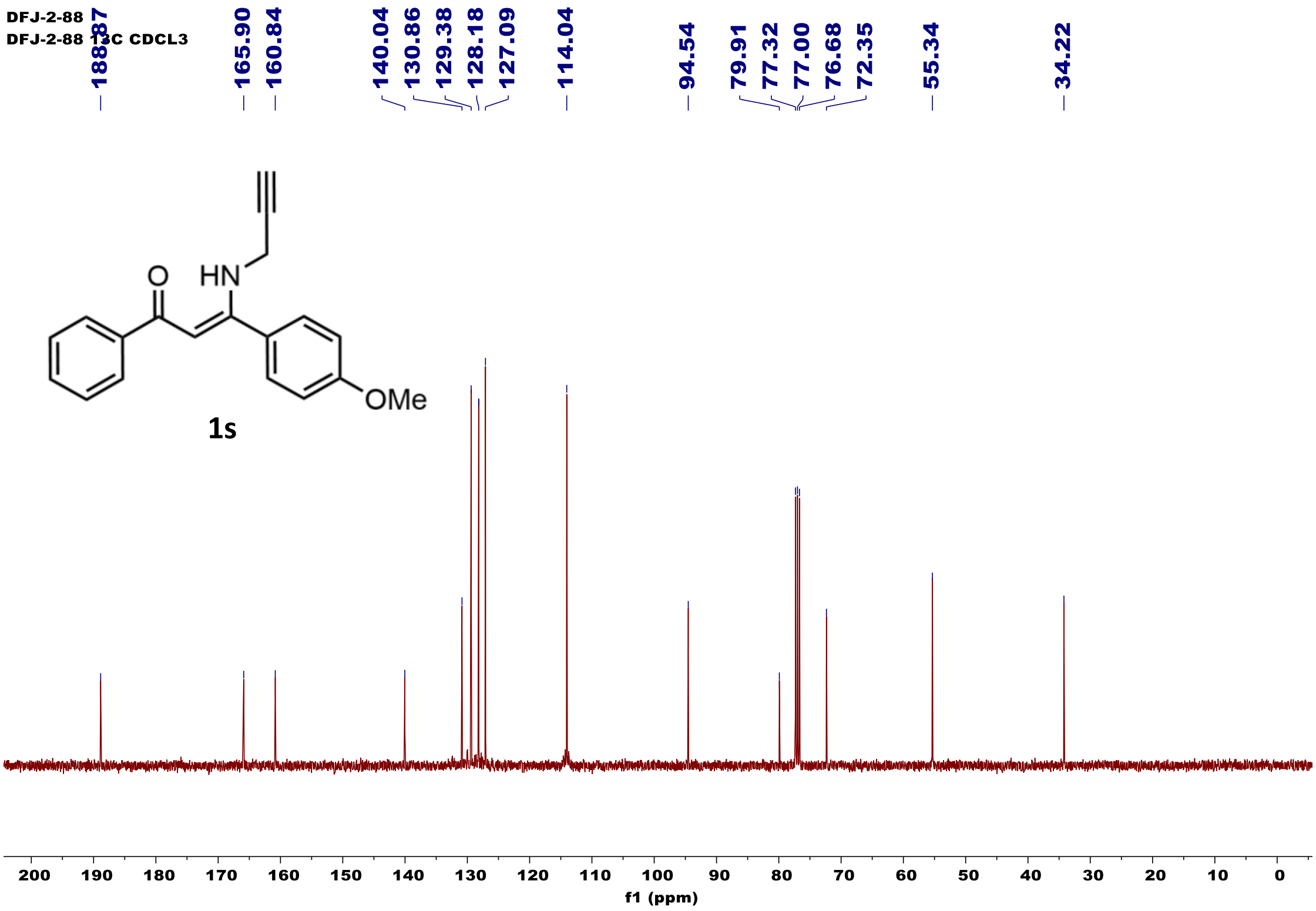

^{1}H NMR spectrum of compound 1t (400 MHz, $CDCl_3$)

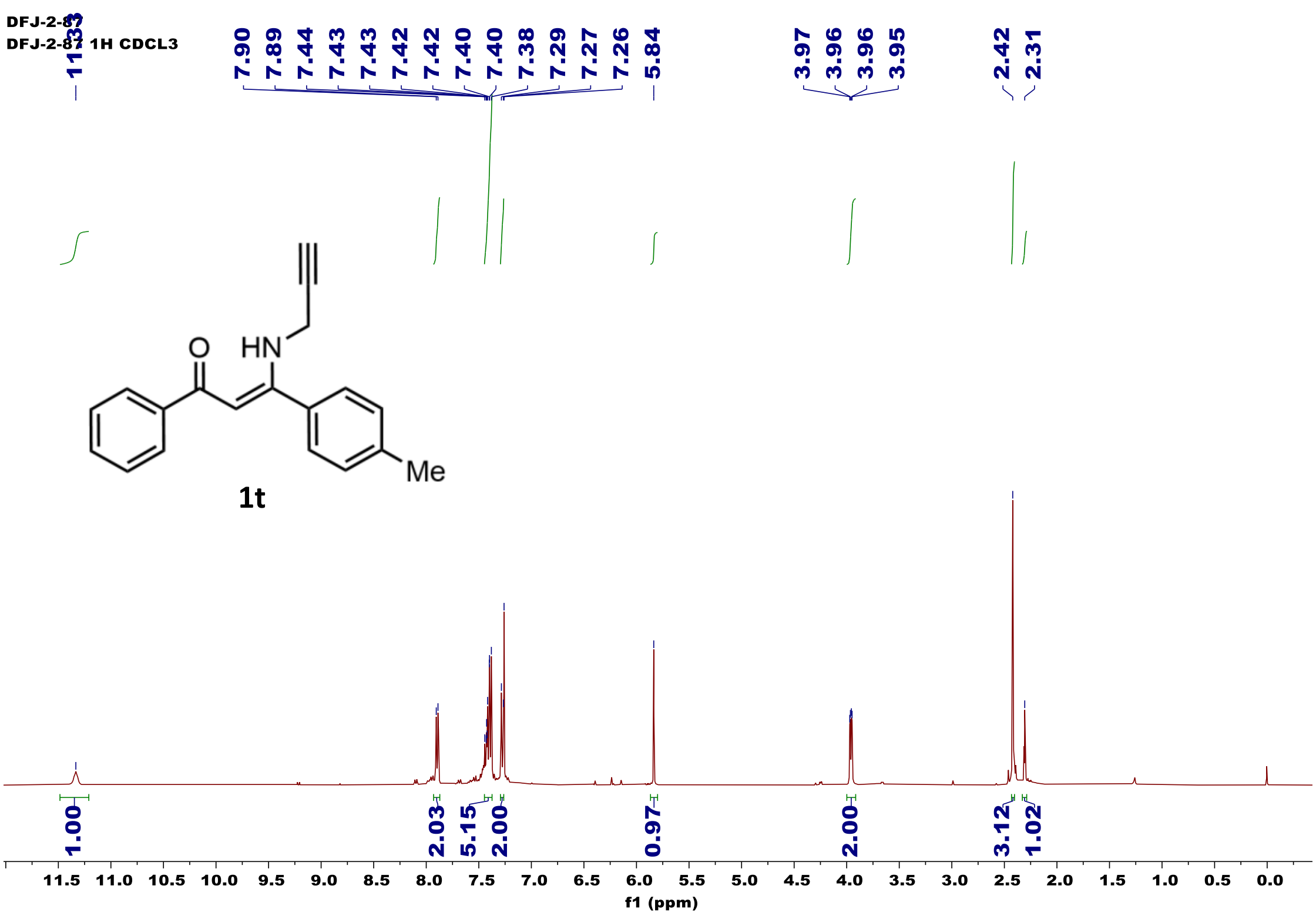

^{13}C NMR spectrum of compound 1t (101 MHz, $CDCl_3$)

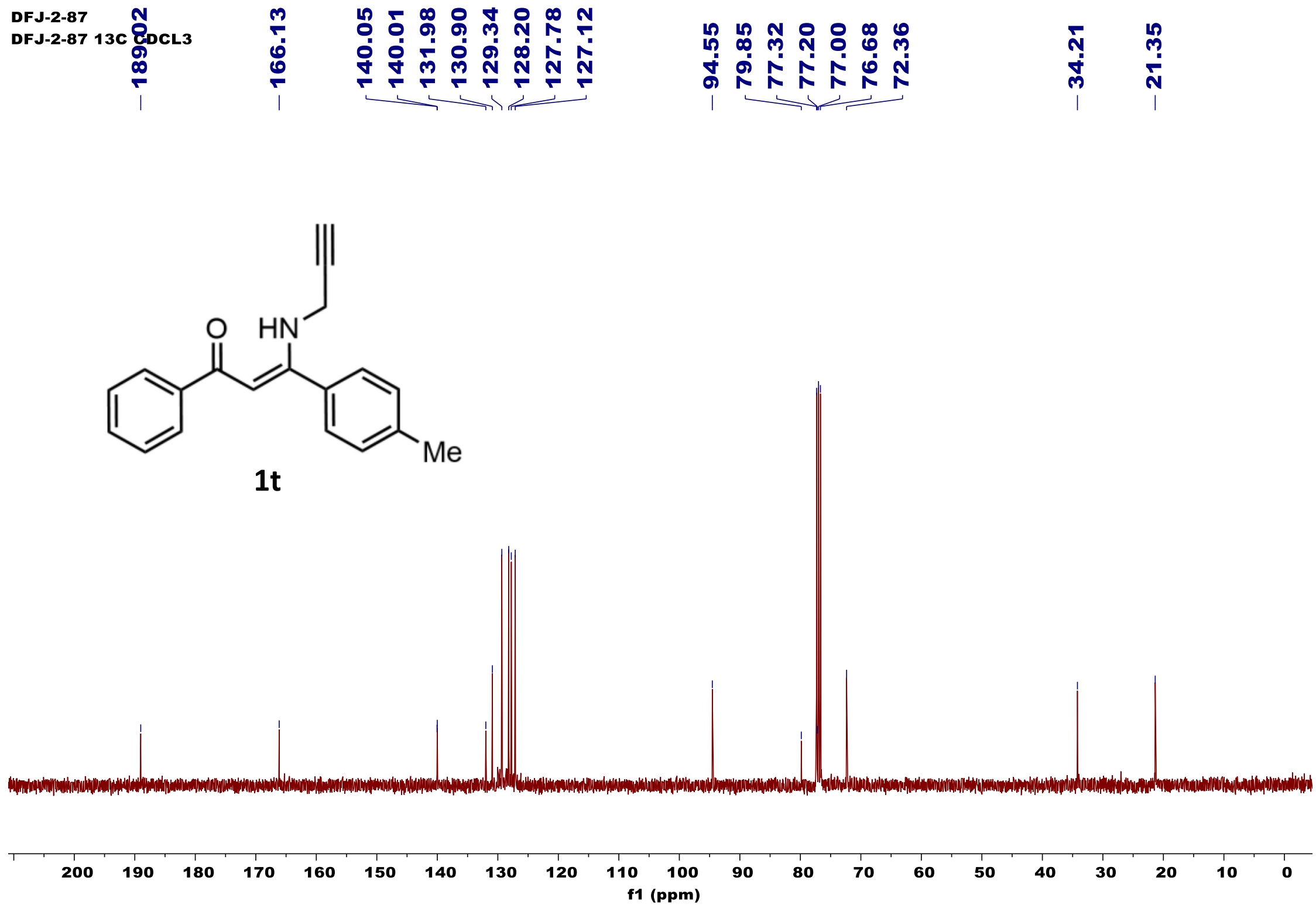

^{1}H NMR spectrum of compound 1u (400 MHz, $CDCl_3$)

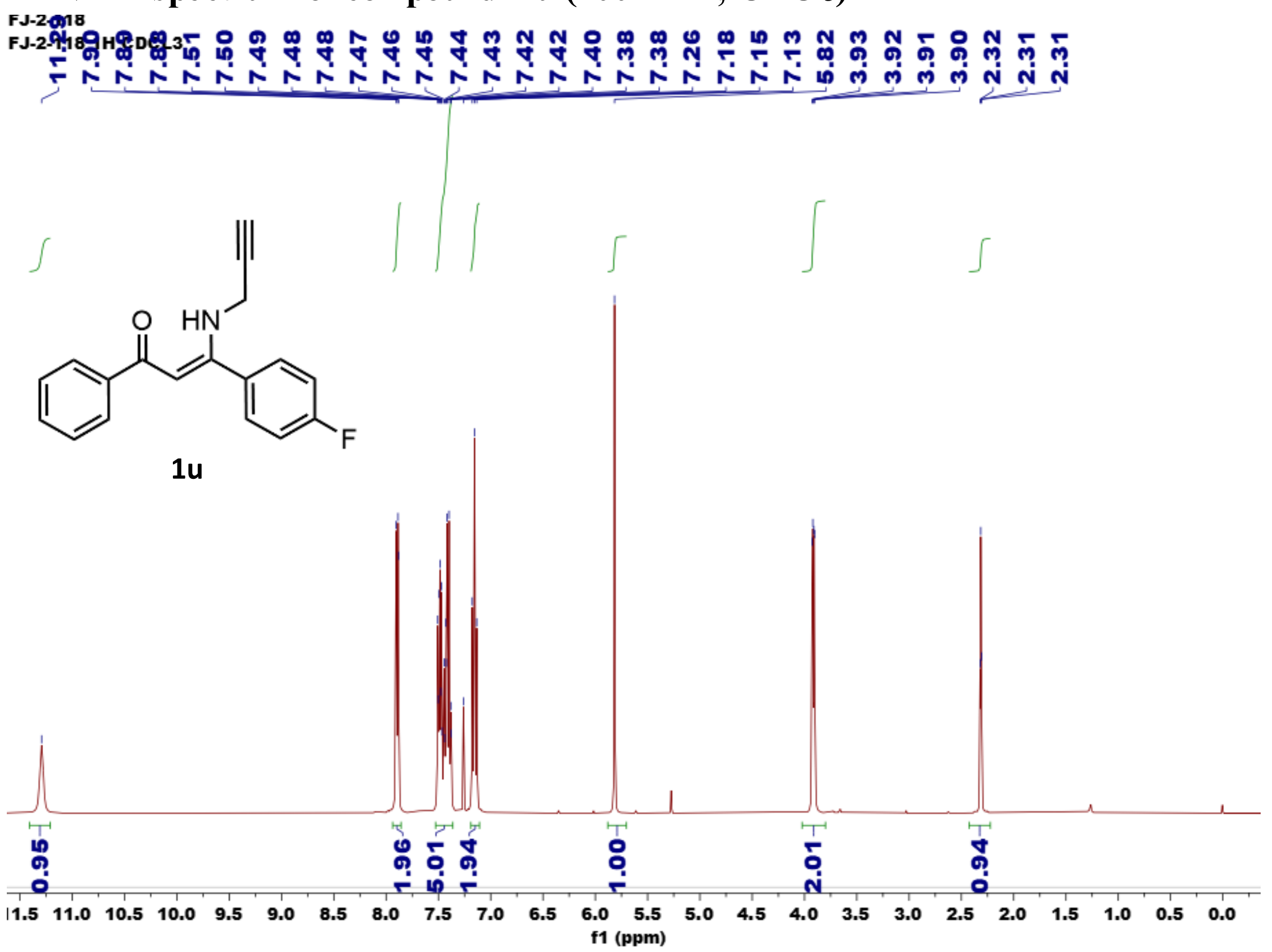

^{13}C NMR spectrum of compound 1u (101 MHz, $CDCl_3$)

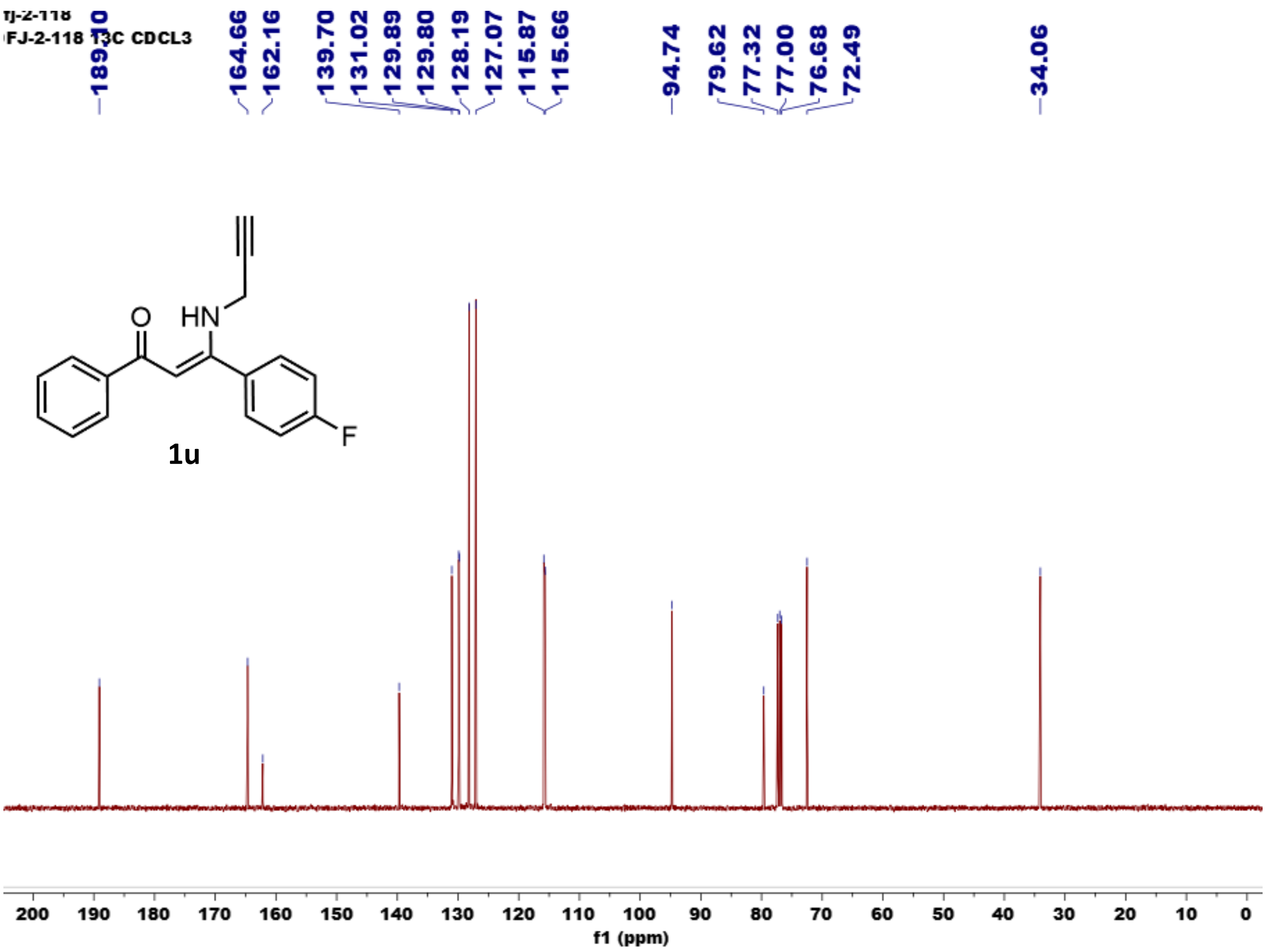

^{1}H NMR spectrum of compound 1v (400 MHz, $CDCl_3$)

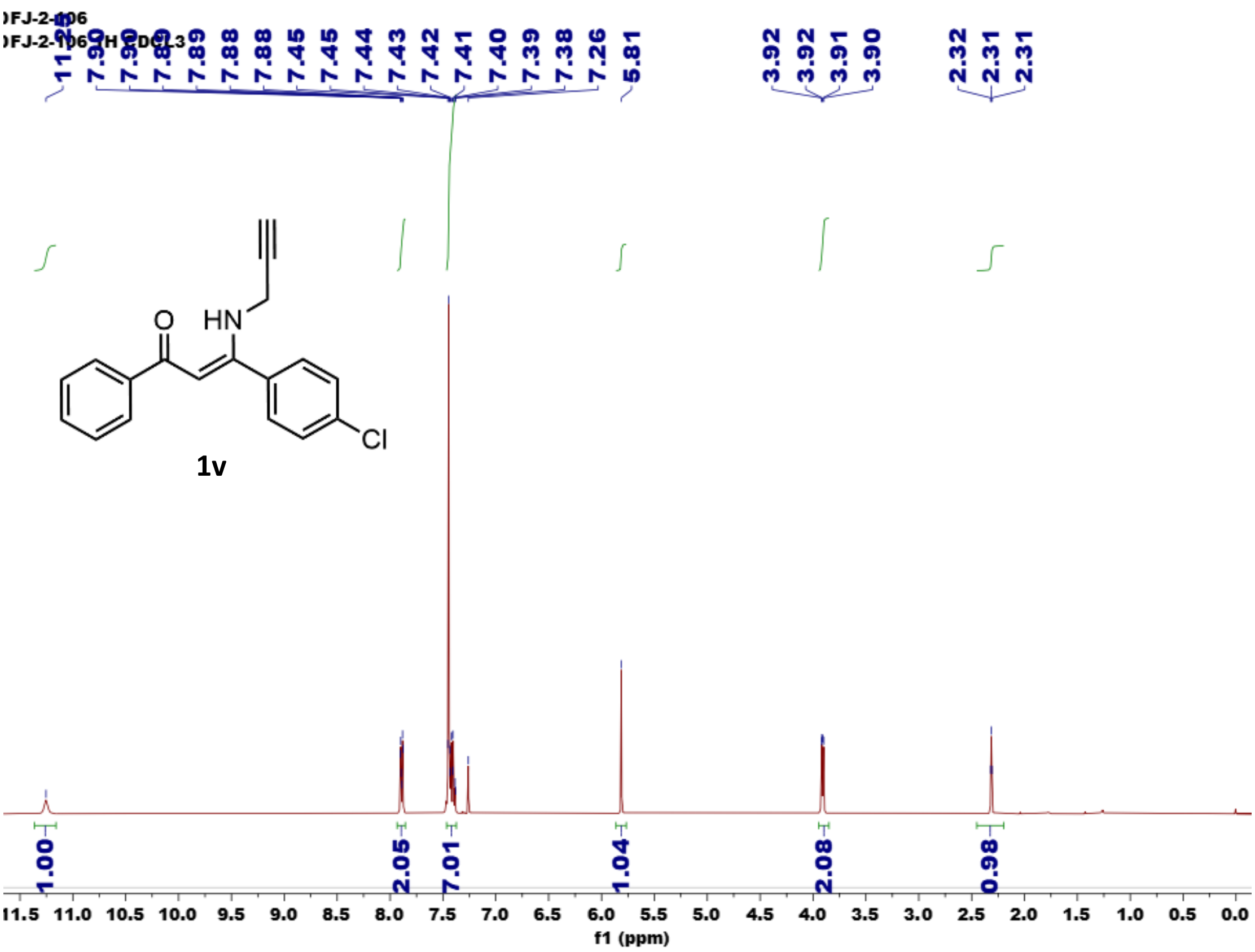

^{13}C NMR spectrum of compound 1v (101 MHz, $CDCl_3$)

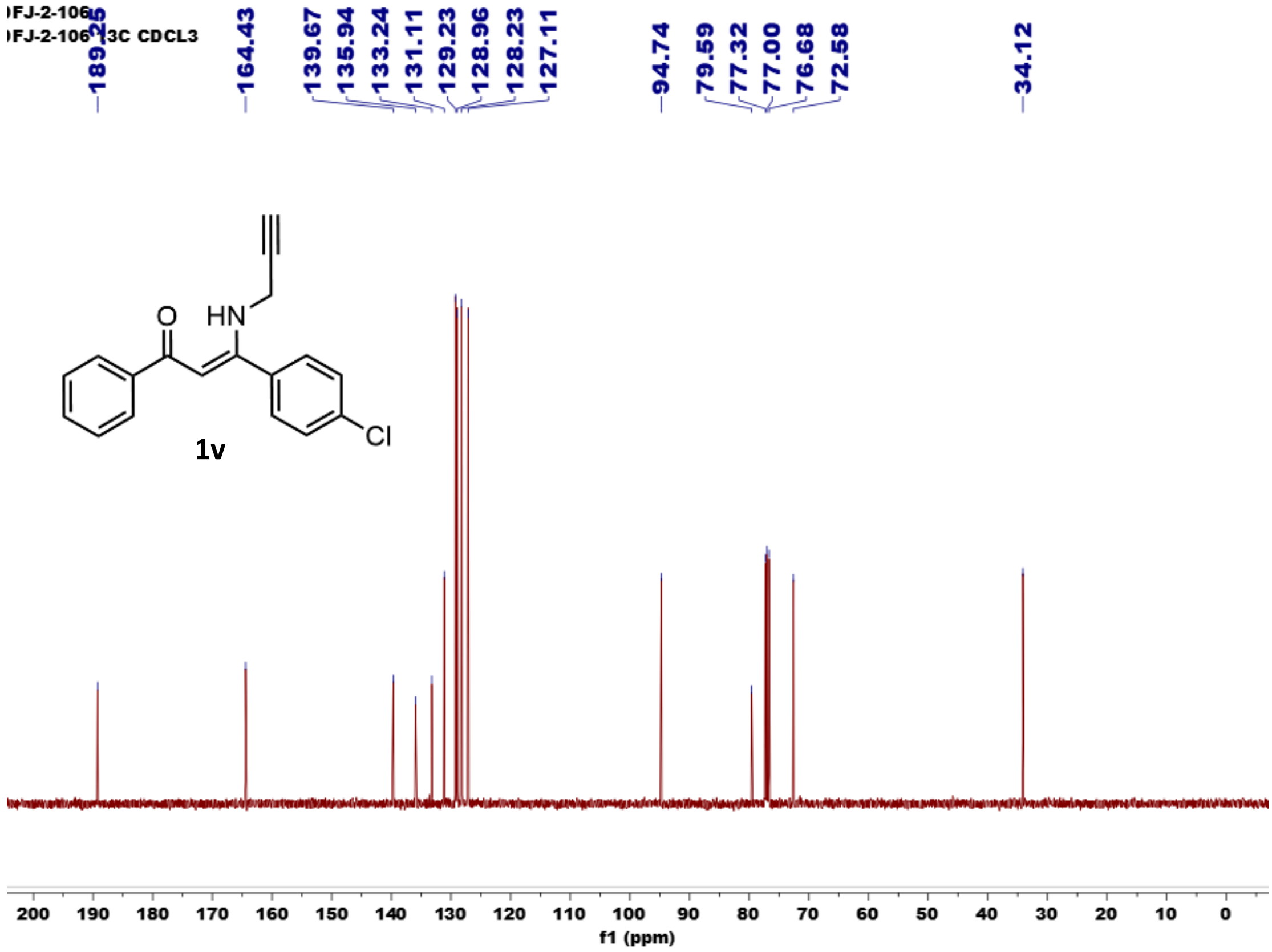

^{1}H NMR spectrum of compound 1w (400 MHz, $CDCl_3$)

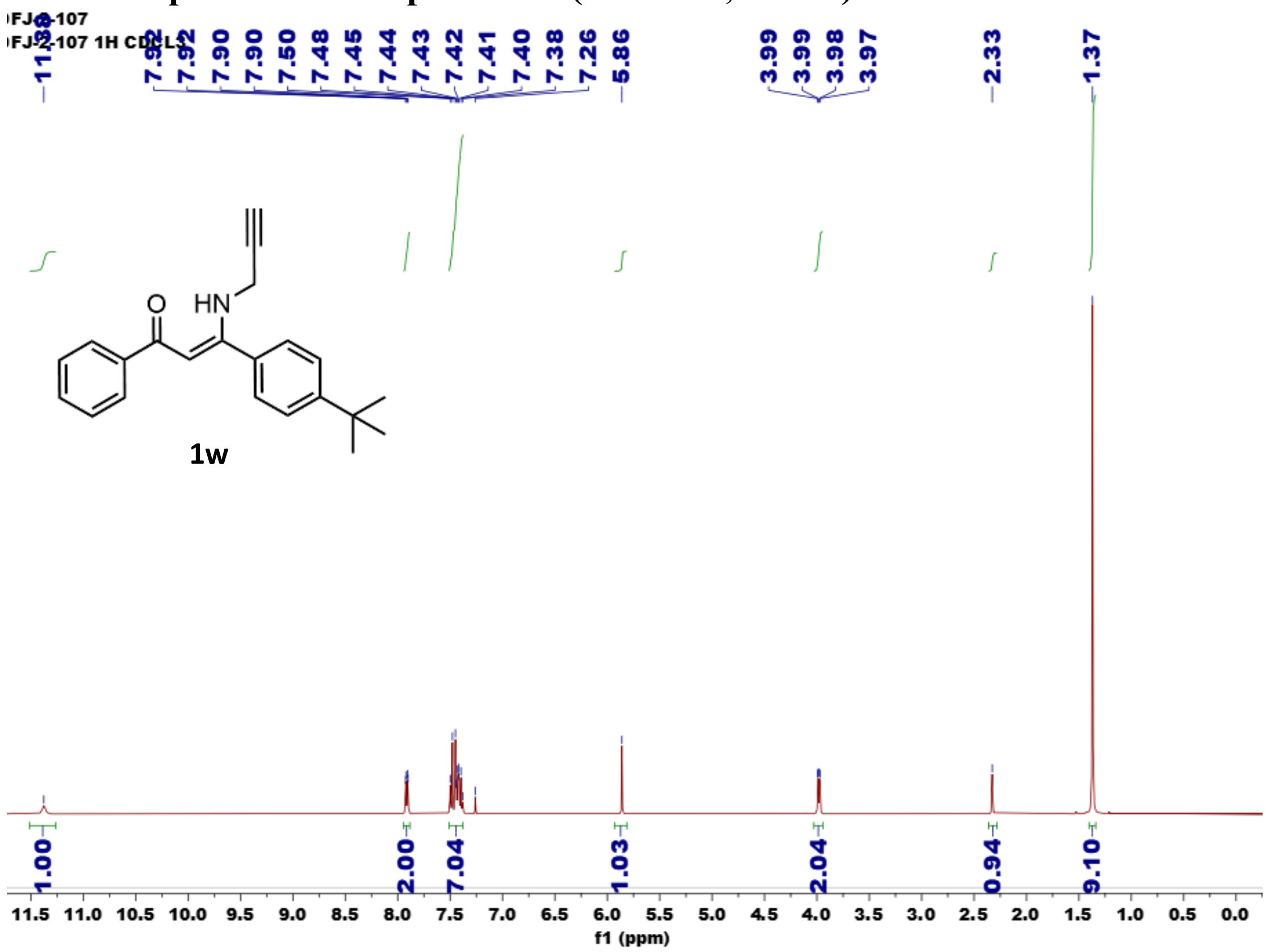

^{13}C NMR spectrum of compound 1w (101 MHz, $CDCl_3$)

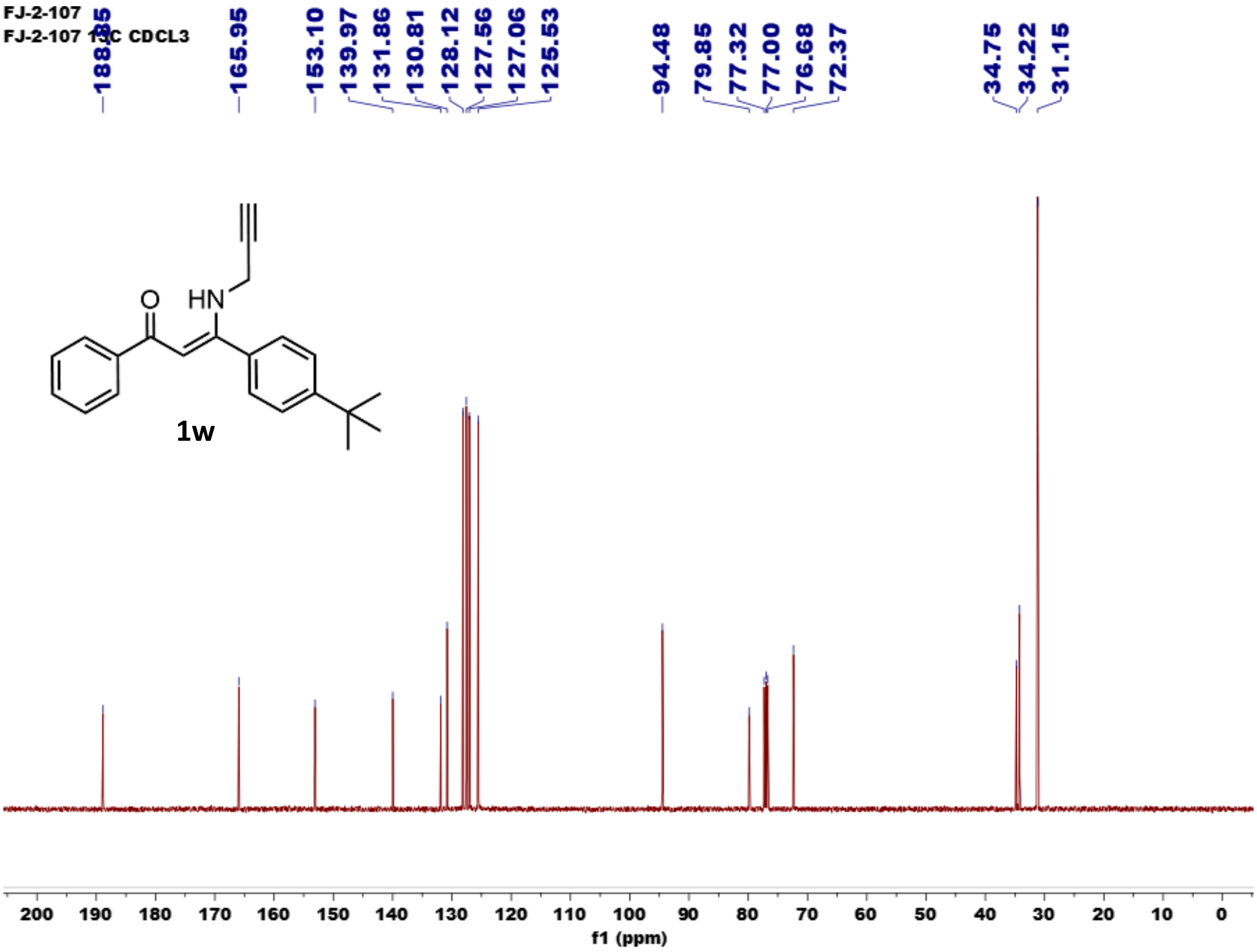

^{1}H NMR spectrum of compound 1x (400 MHz, $CDCl_3$)

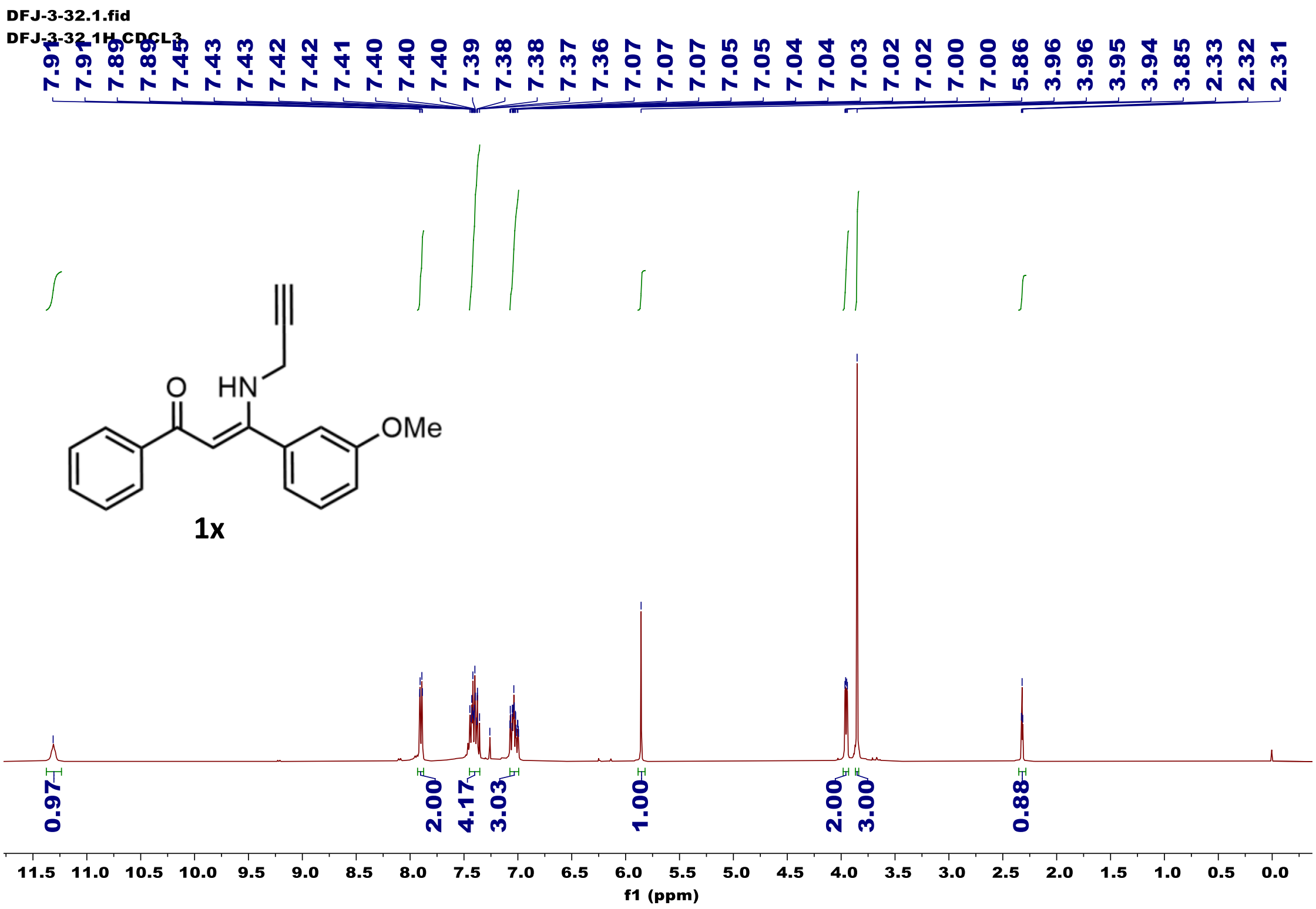

^{13}C NMR spectrum of compound 1x (101 MHz, $CDCl_3$)

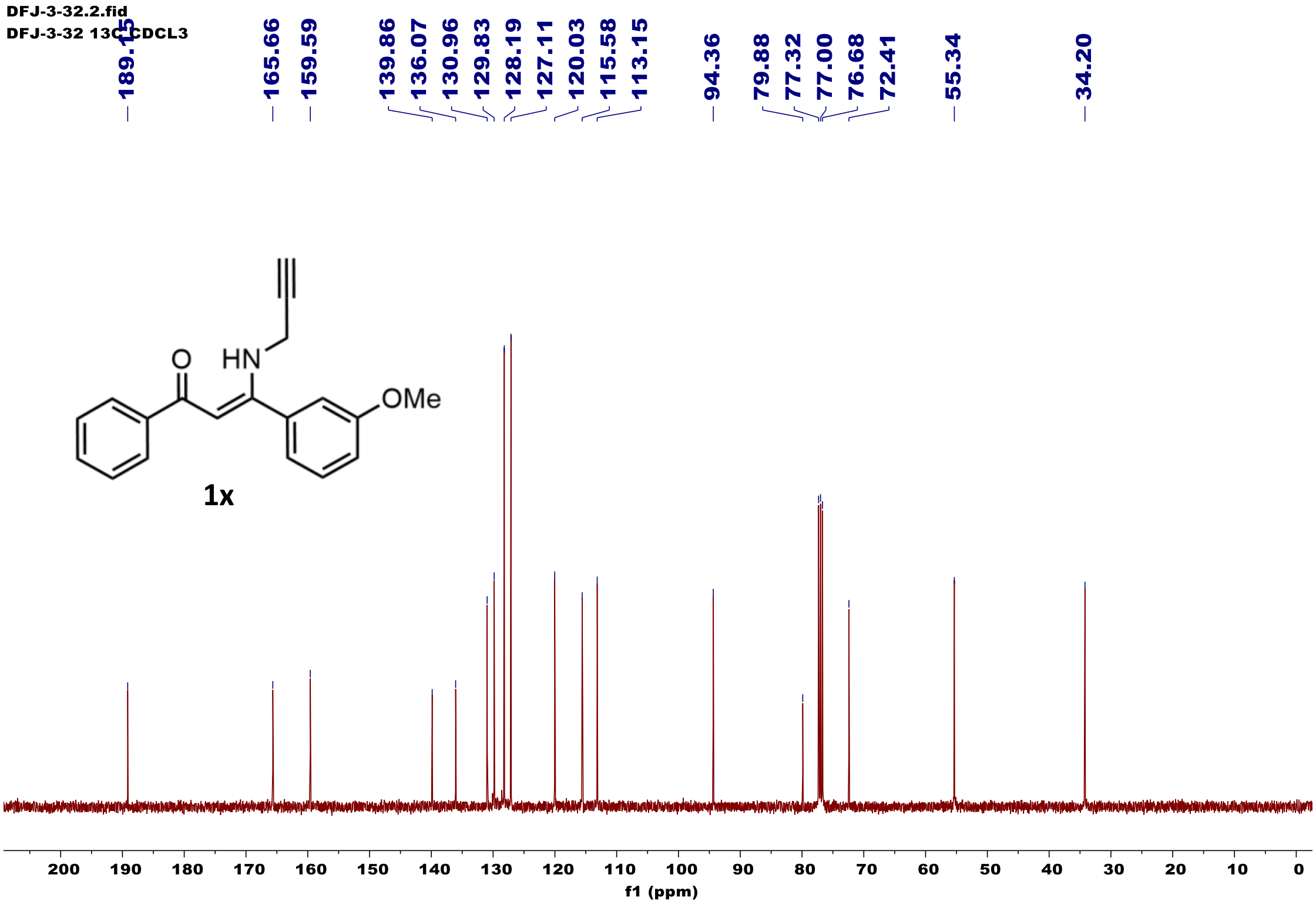

^{1}H NMR spectrum of compound 1y (400 MHz, $CDCl_3$)

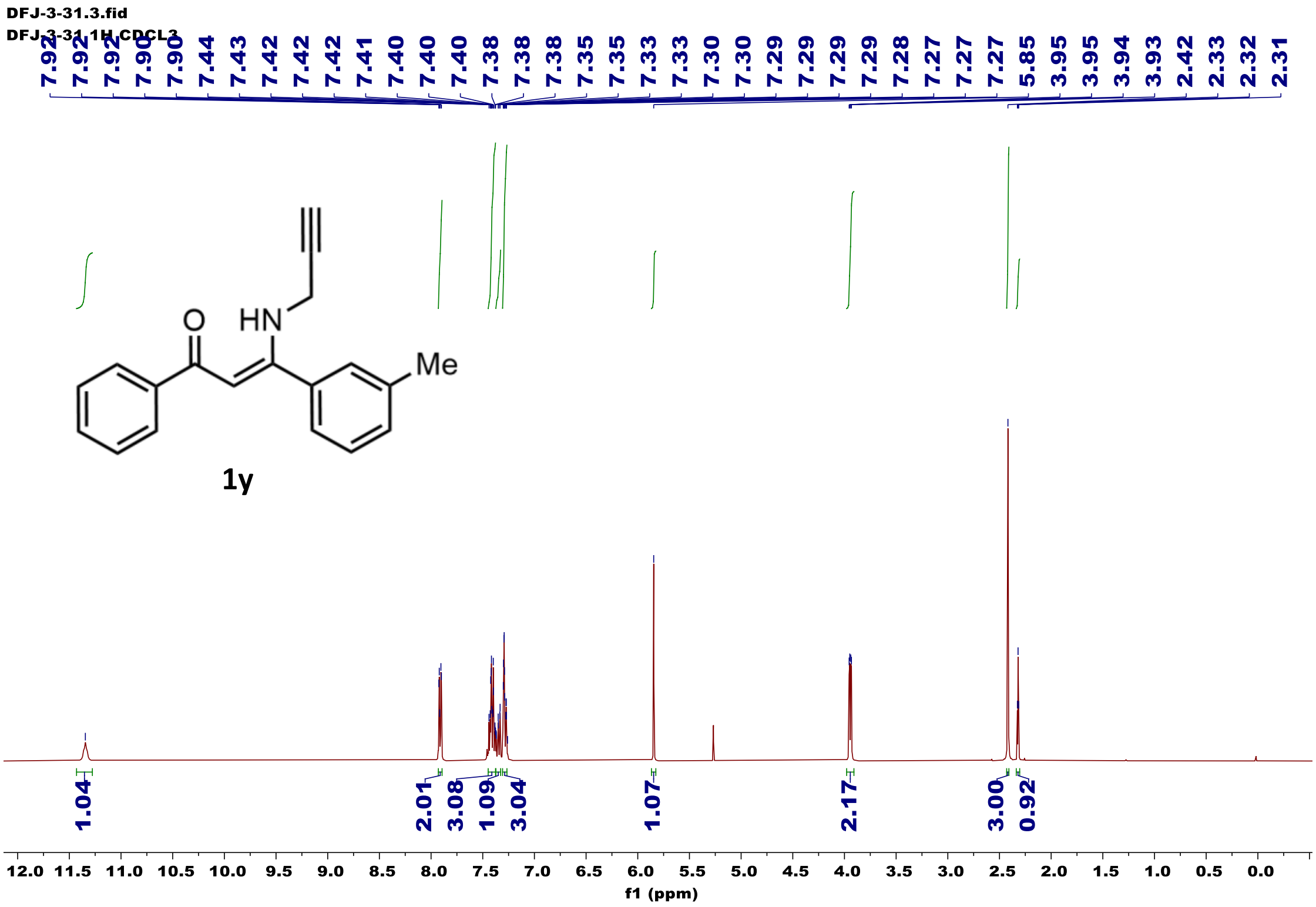

^{13}C NMR spectrum of compound 1y (101 MHz, $CDCl_3$)

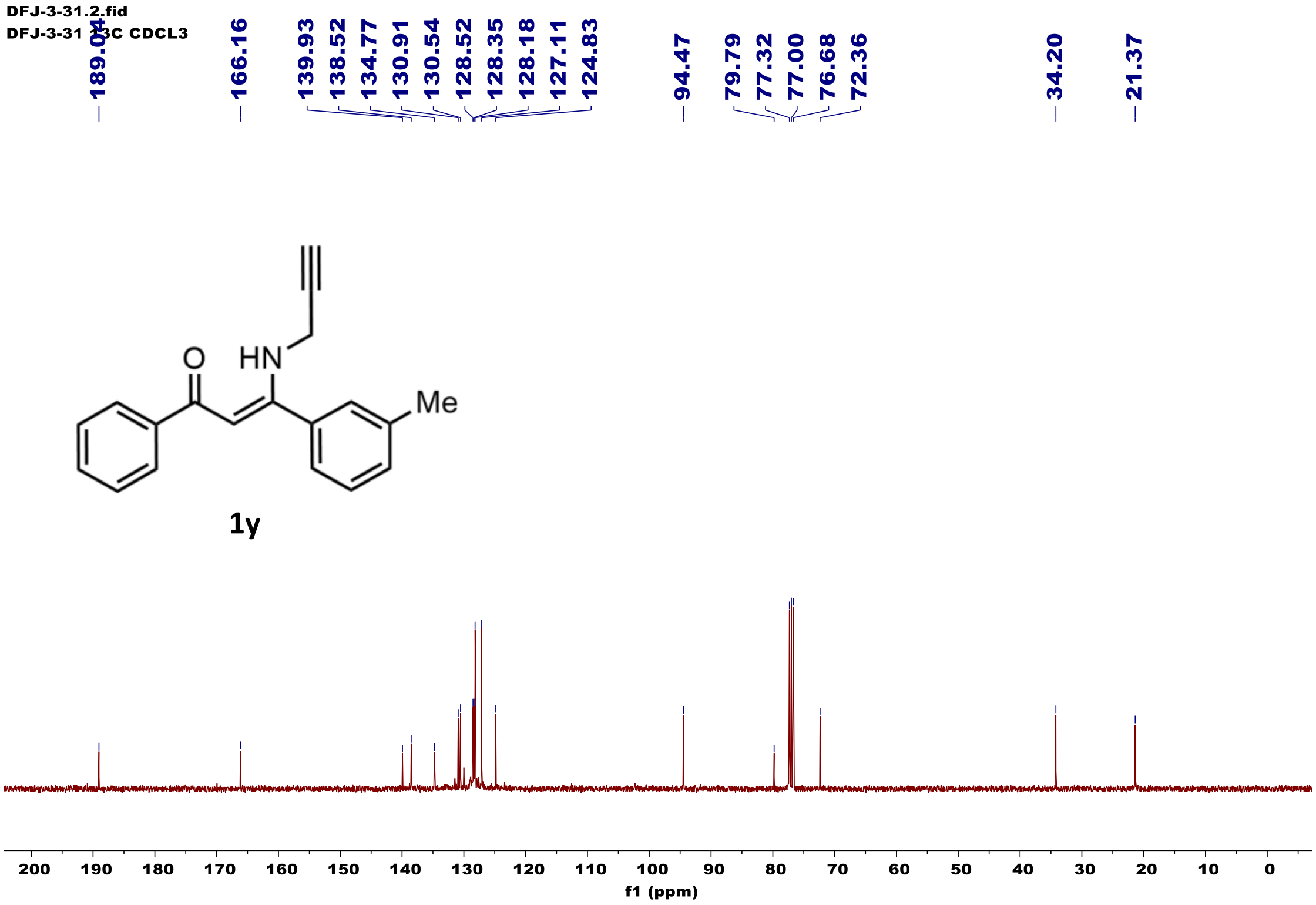

^{1}H NMR spectrum of compound 1z (400 MHz, $CDCl_3$)

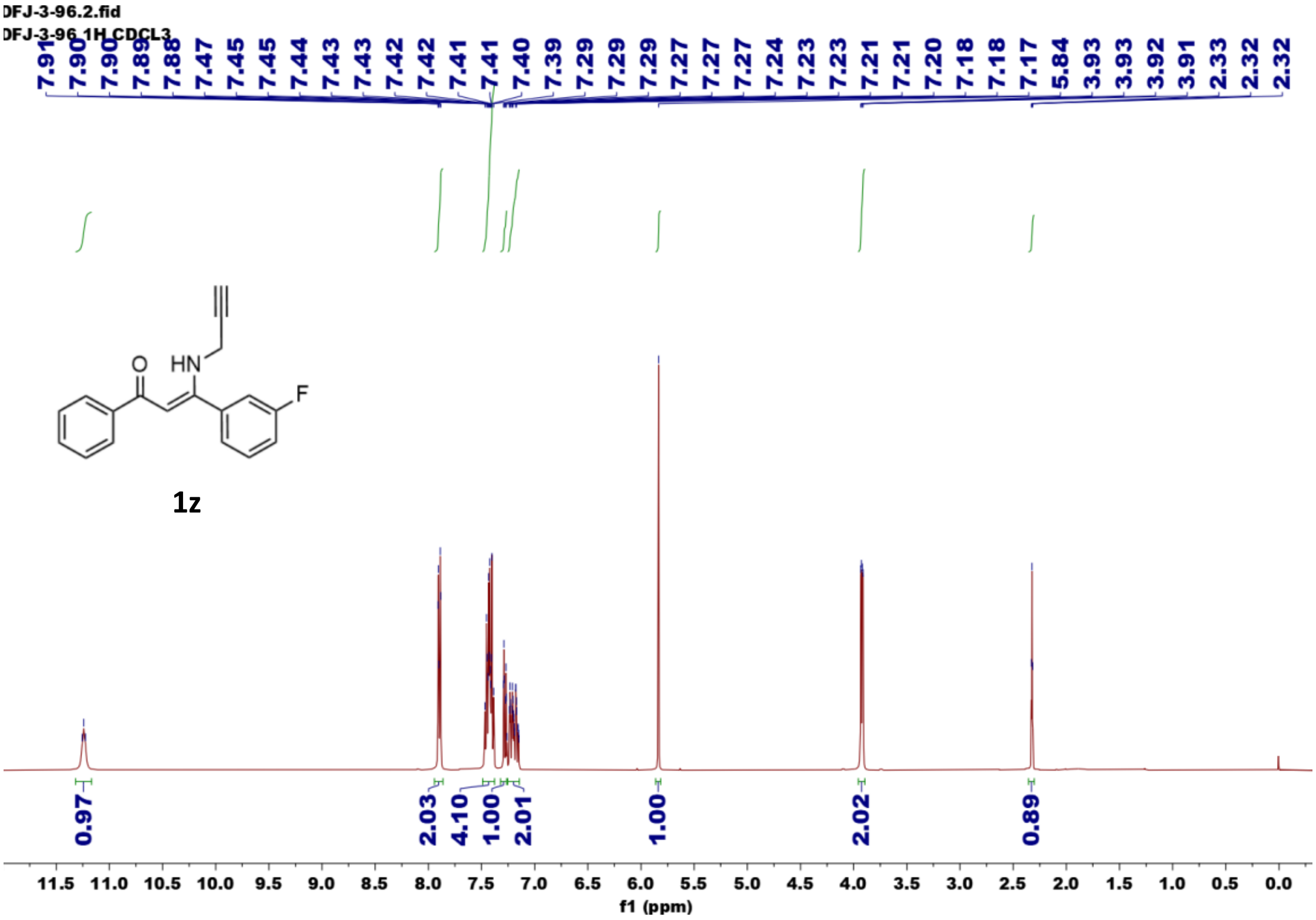

^{13}C NMR spectrum of compound 1z (101 MHz, $CDCl_3$)

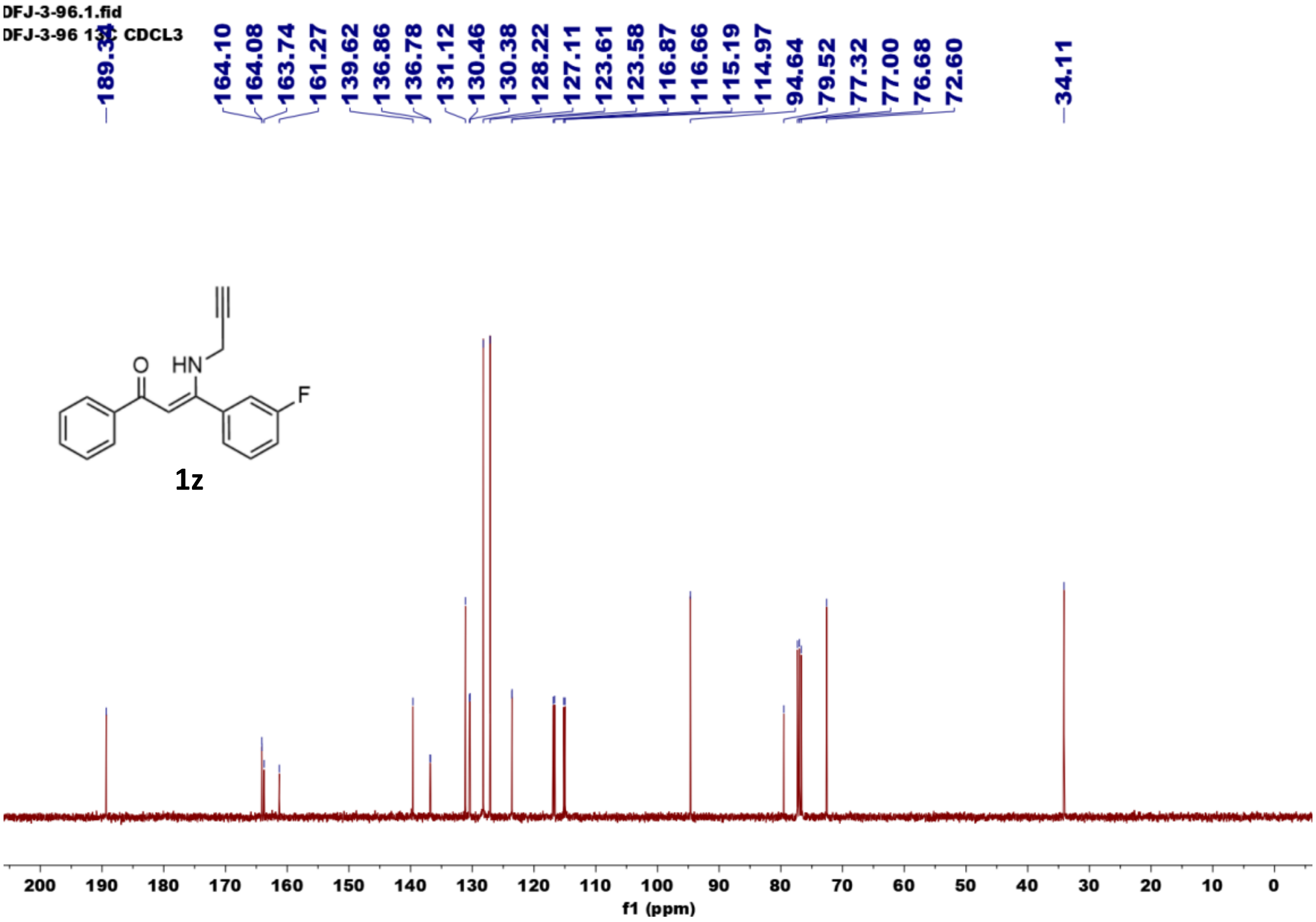

^{1}H NMR spectrum of compound 1aa (400 MHz, $CDCl_3$)

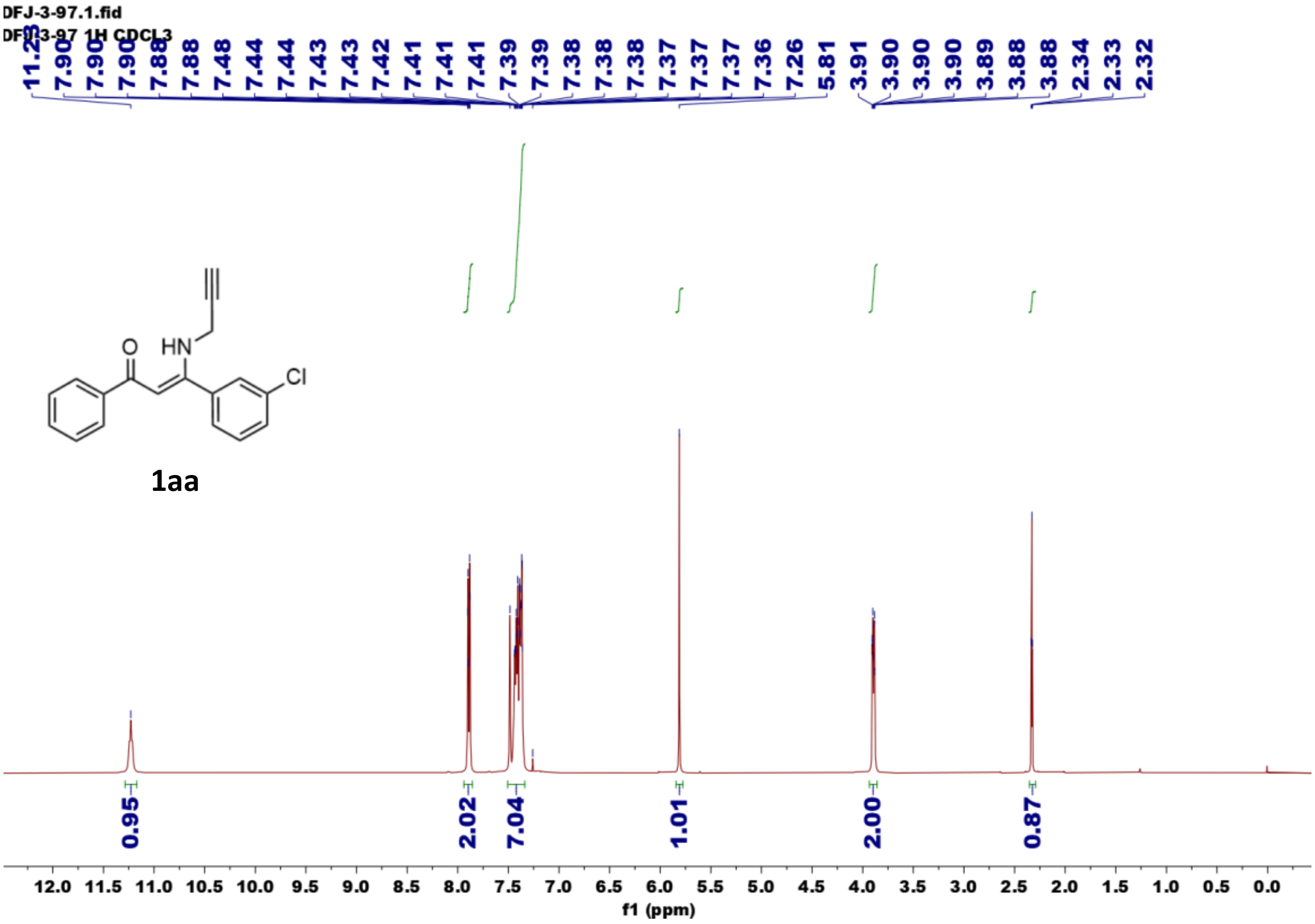

^{13}C NMR spectrum of compound 1aa (101 MHz, $CDCl_3$)

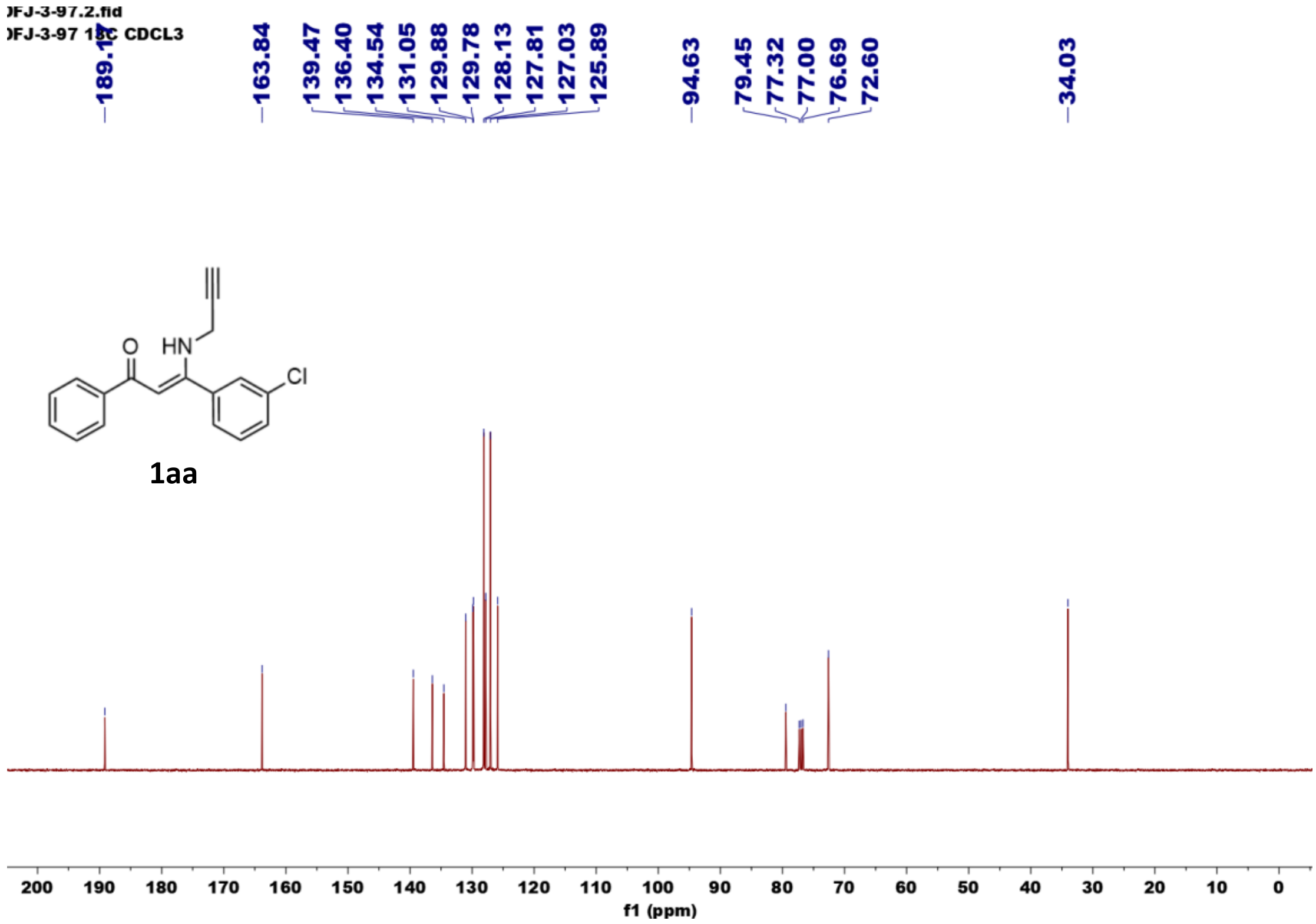

^{1}H NMR spectrum of compound 1ab (400 MHz, $CDCl_3$)

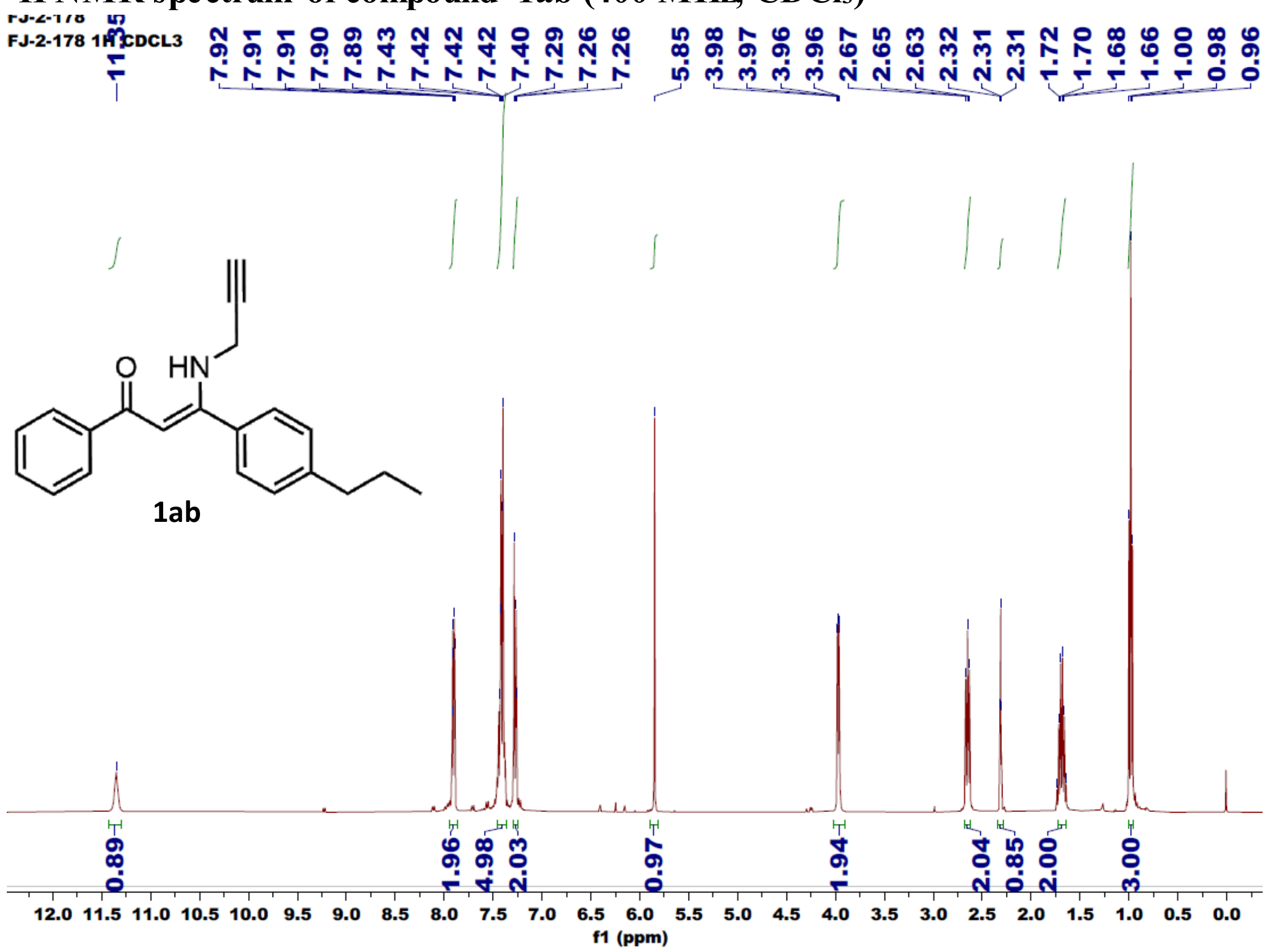

^{13}C NMR spectrum of compound 1ab (101 MHz, $CDCl_3$)

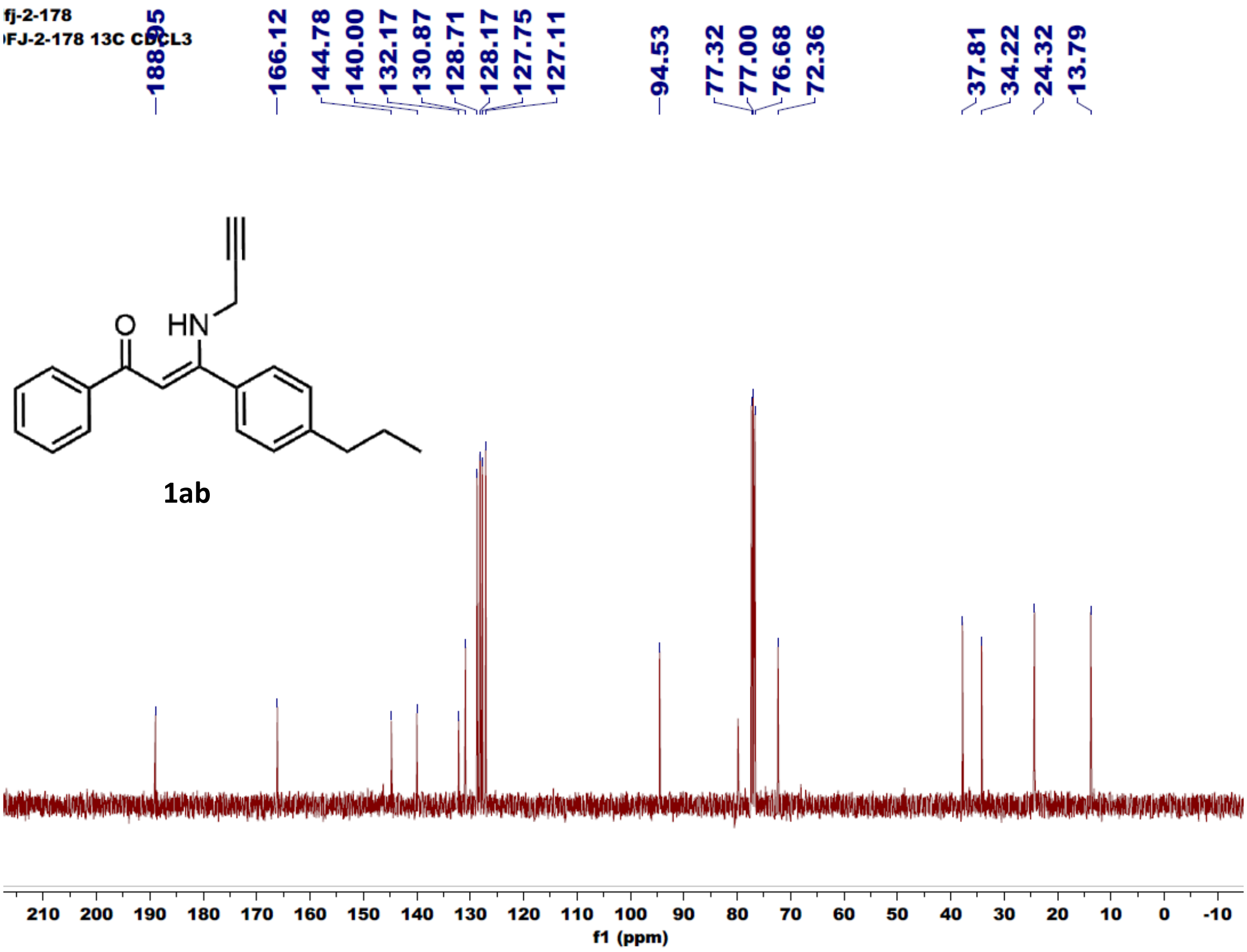

^{1}H NMR spectrum of compound 1ad (400 MHz, $CDCl_3$)

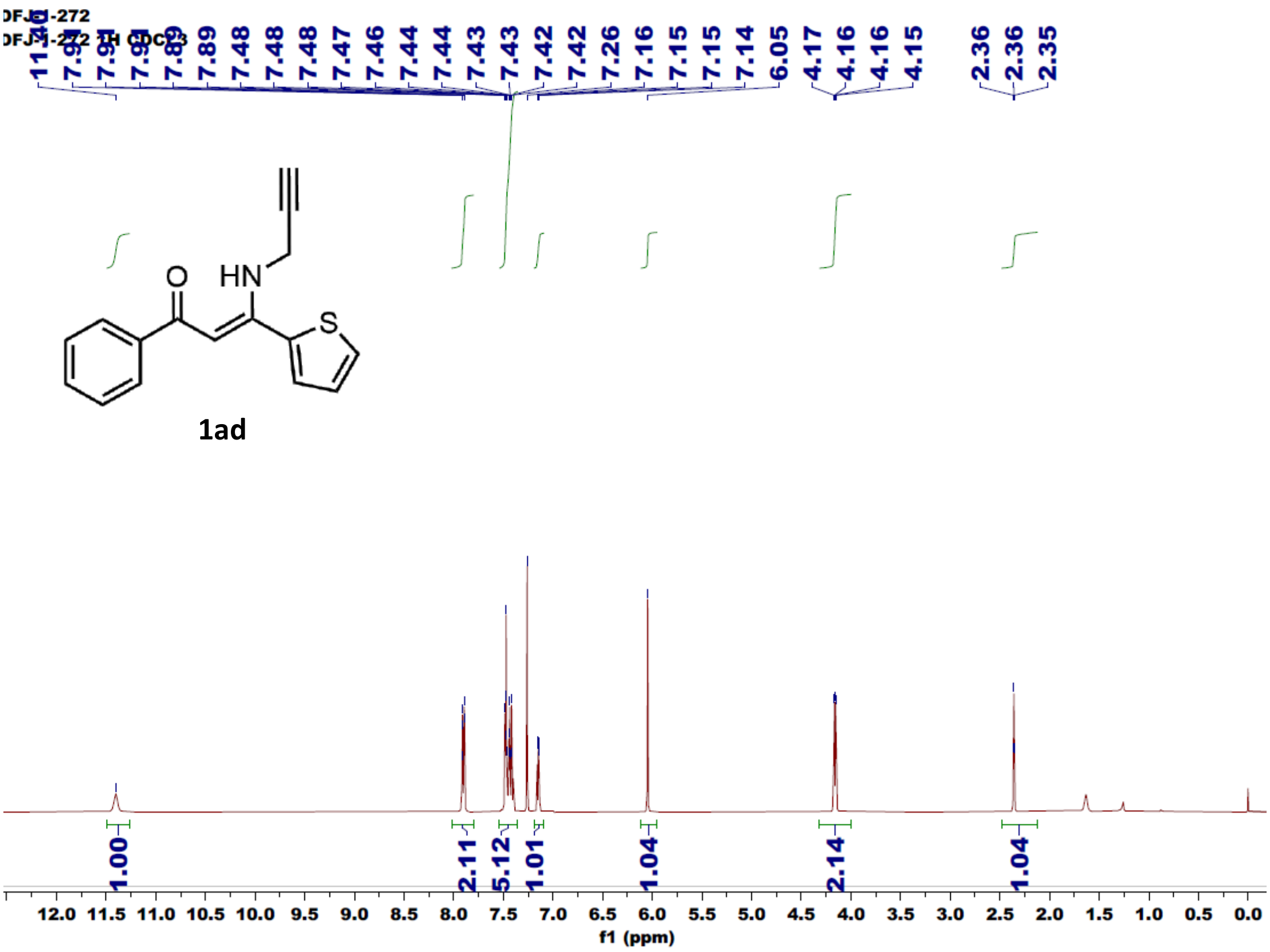

^{13}C NMR spectrum of compound 1ad (101 MHz, $CDCl_3$)

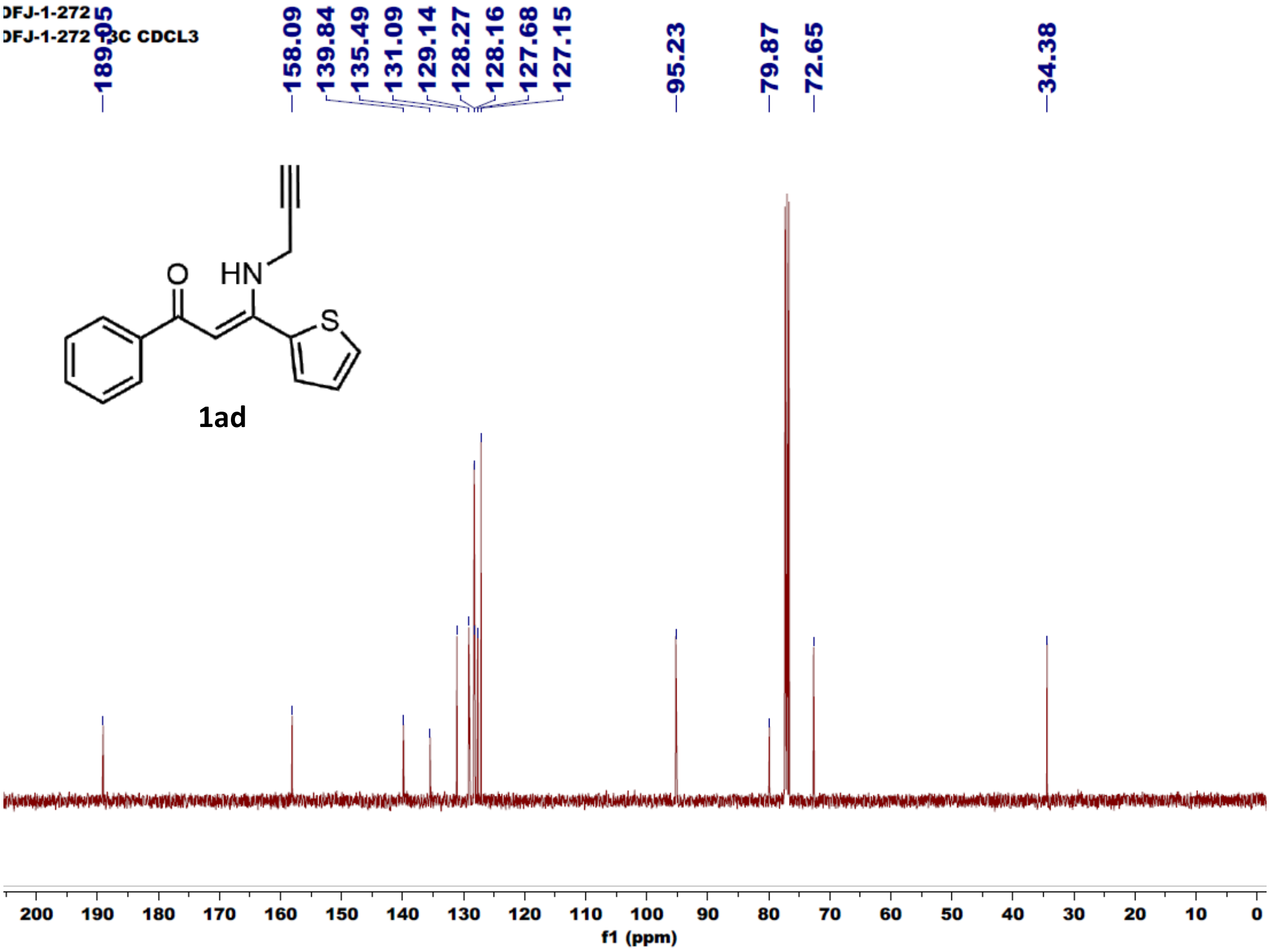

^{1}H NMR spectrum of compound 1ae (400 MHz, $CDCl_3$)

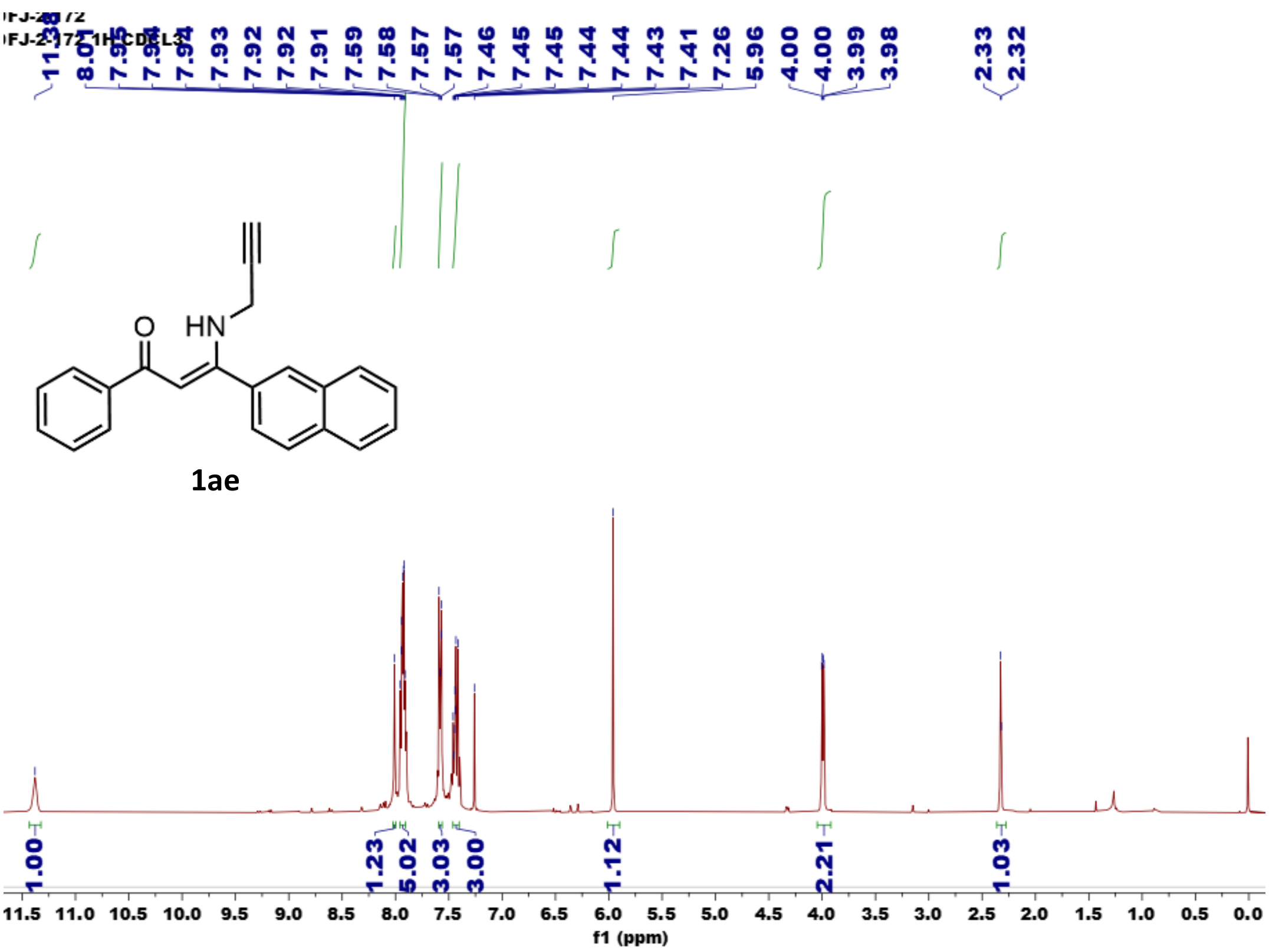

^{13}C NMR spectrum of compound 1ae (101 MHz, $CDCl_3$)

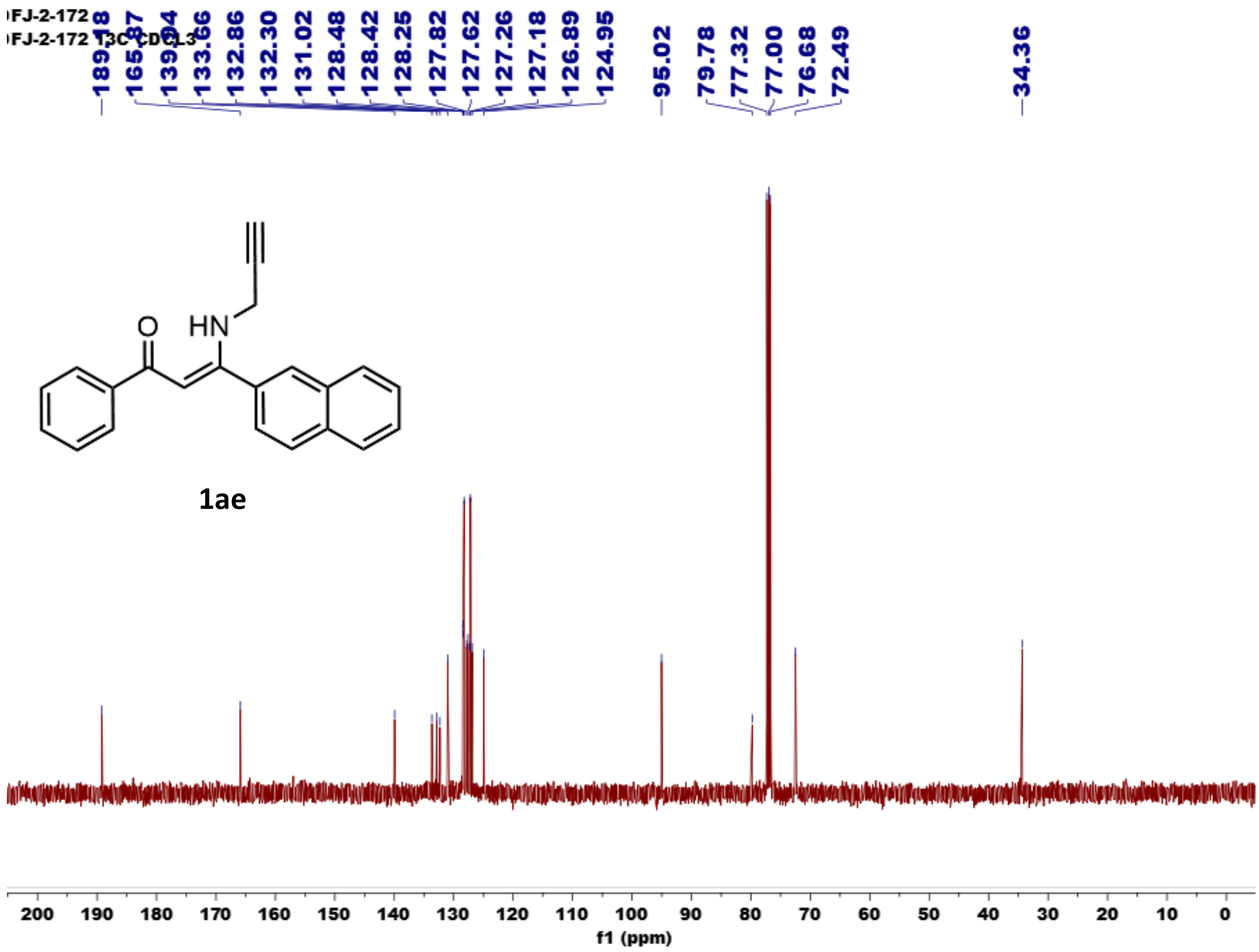

^{1}H NMR spectrum of compound 1af (400 MHz, $CDCl_3$)

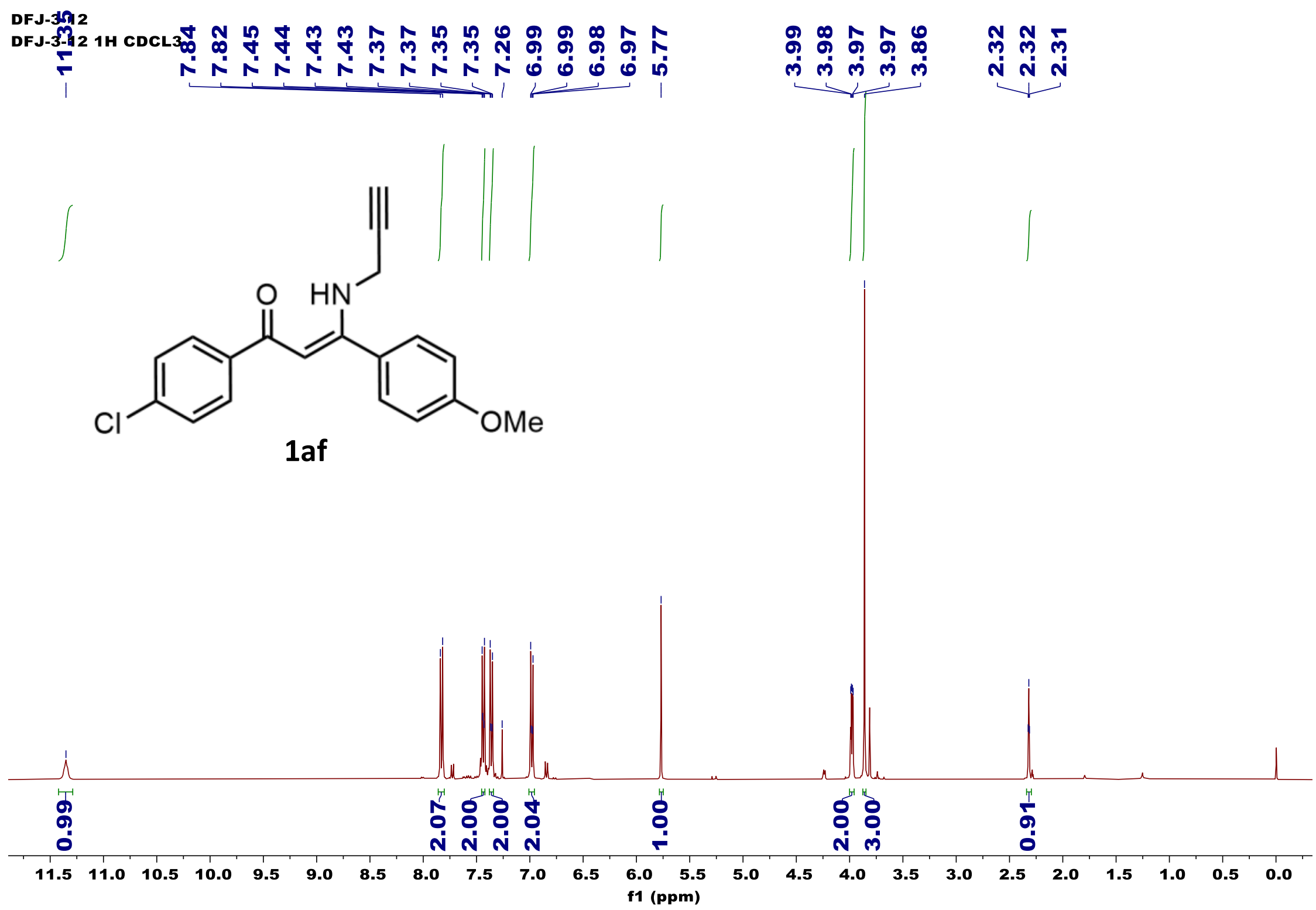

^{13}C NMR spectrum of compound 1af (101 MHz, $CDCl_3$)

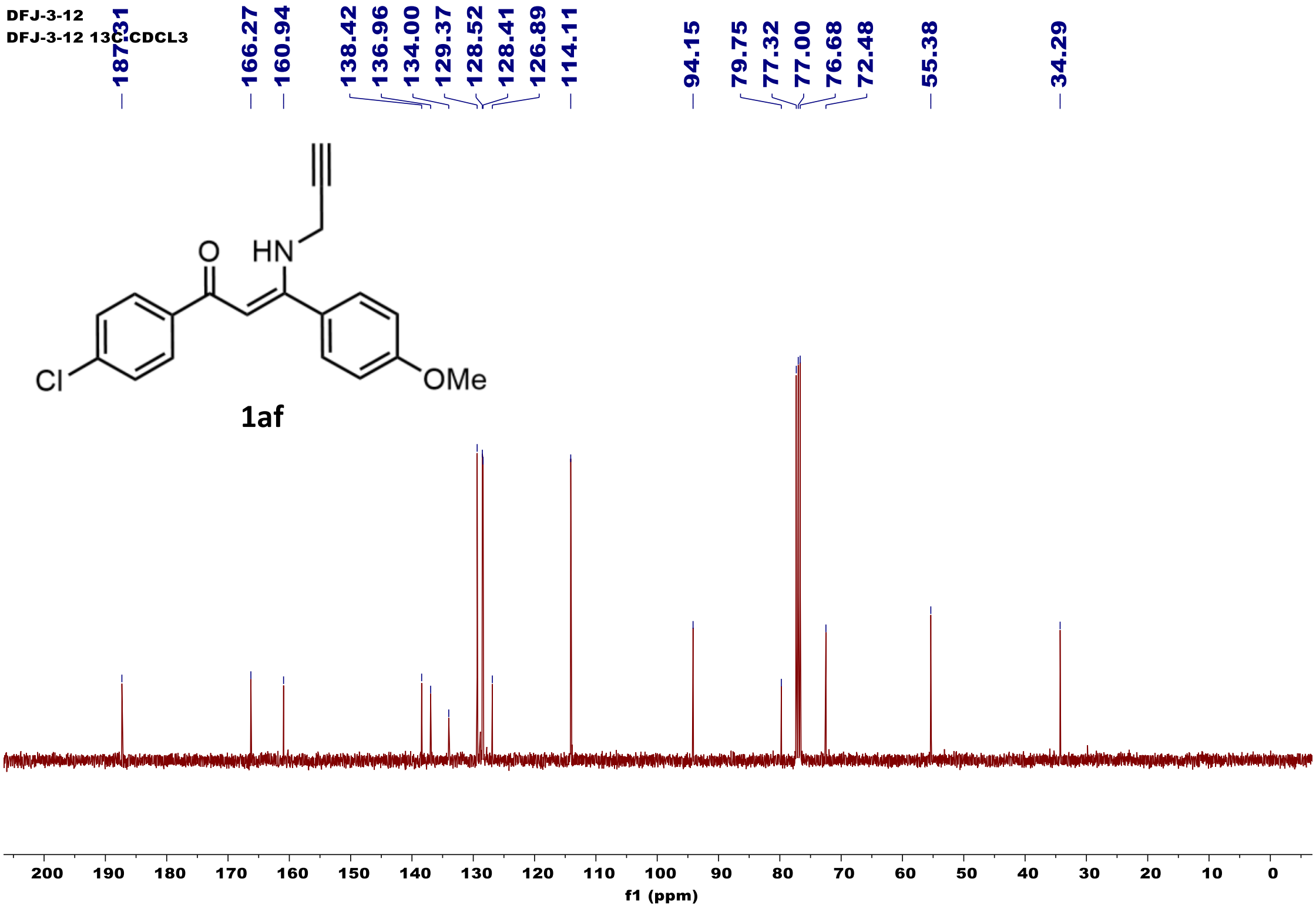

^{1}H NMR spectrum of compound 1ag (400 MHz, $CDCl_3$)

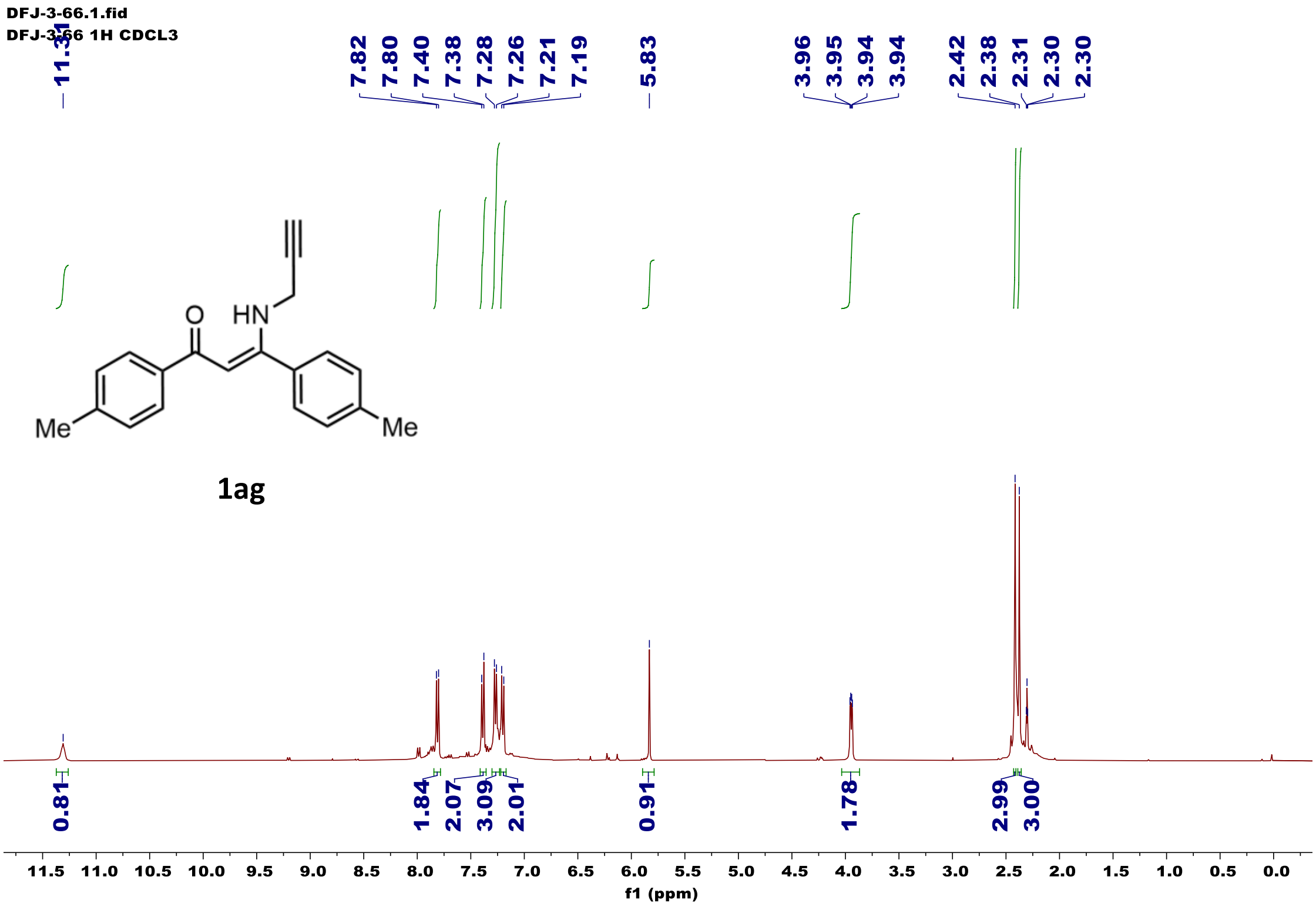

^{13}C NMR spectrum of compound 1ag (101 MHz, $CDCl_3$)

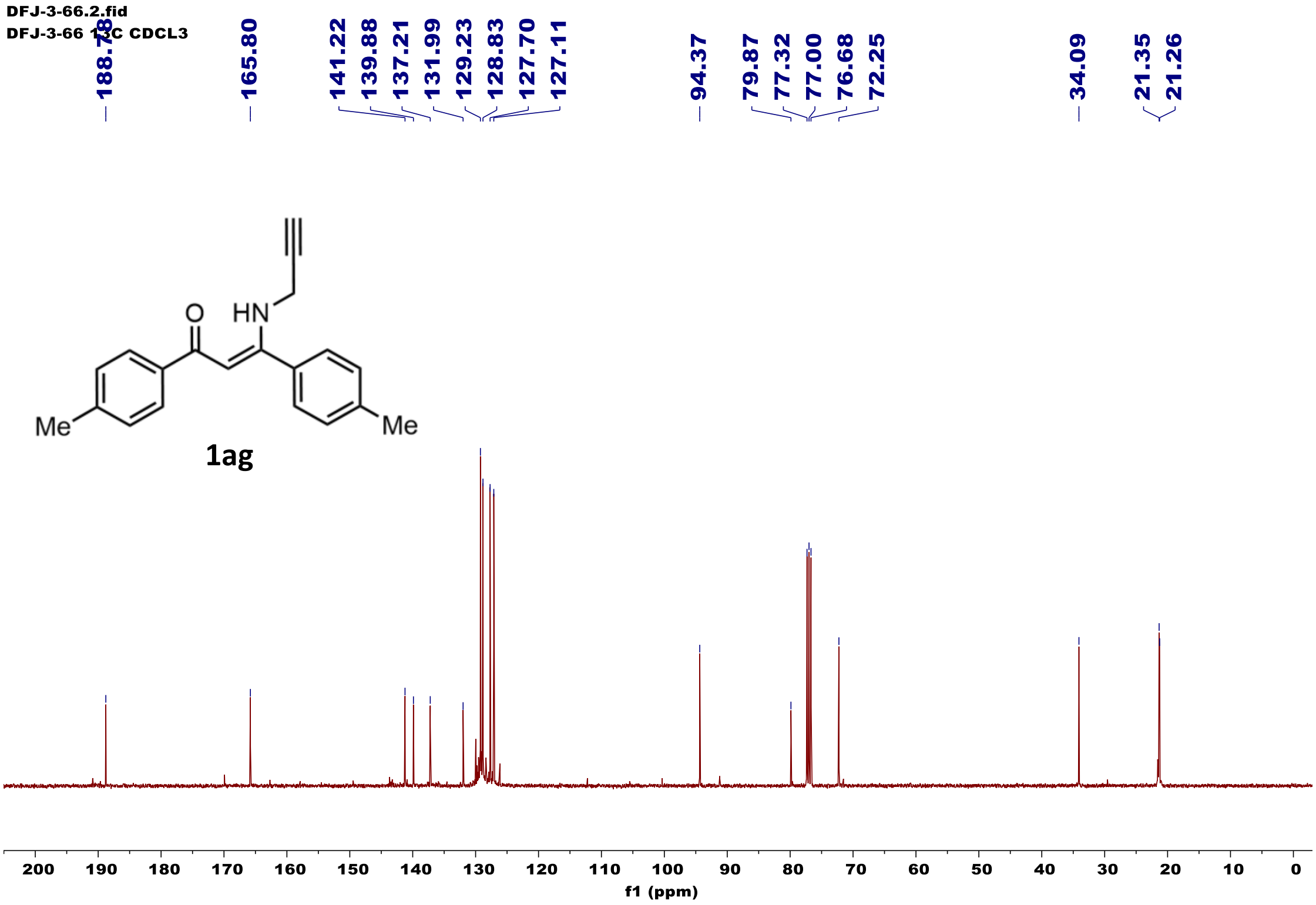

^{1}H NMR spectrum of compound 1ah (400 MHz, $CDCl_3$)

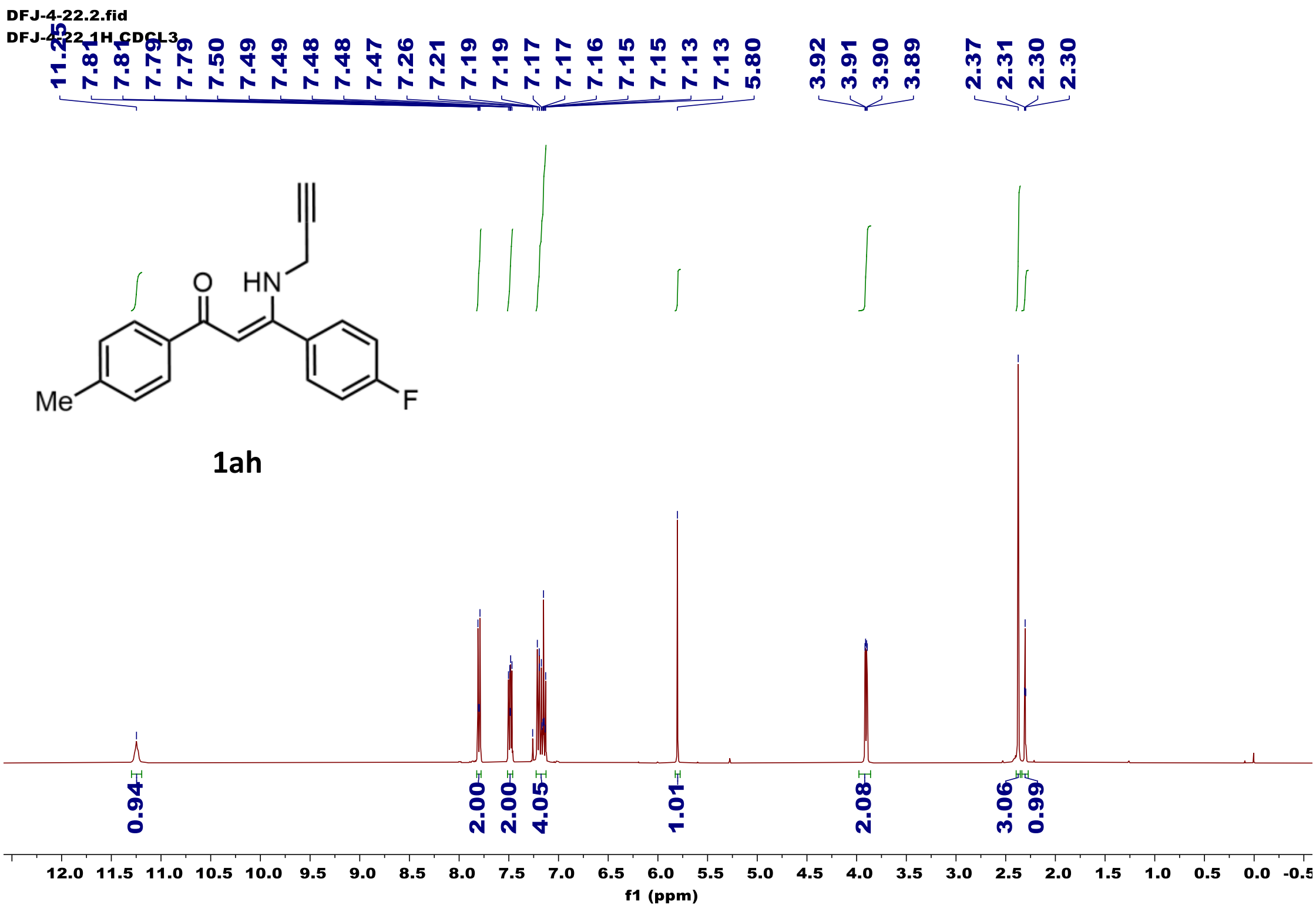

^{13}C NMR spectrum of compound 1ah (101 MHz, $CDCl_3$)

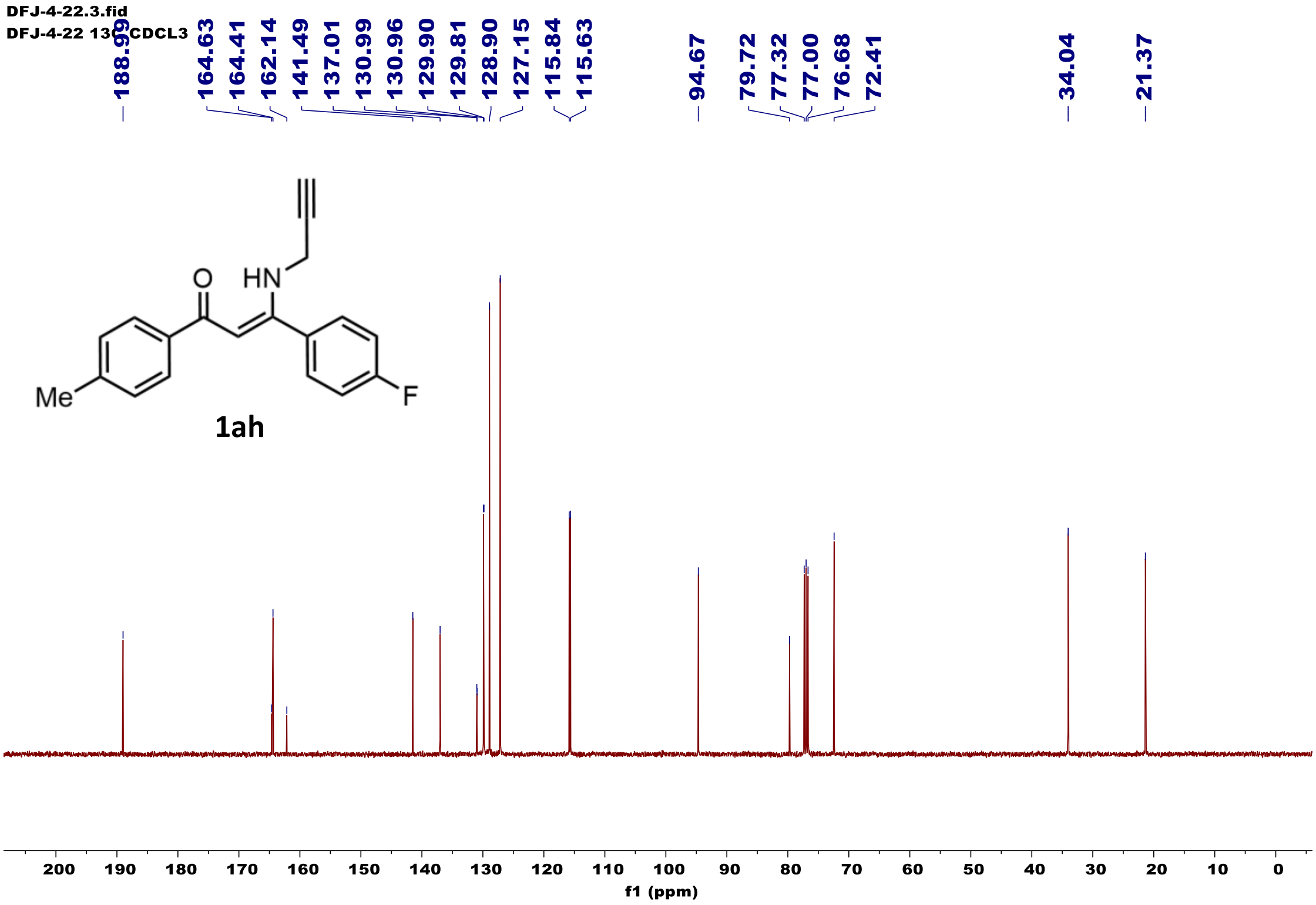

^{1}H NMR spectrum of compound 1ai (400 MHz, $CDCl_3$)

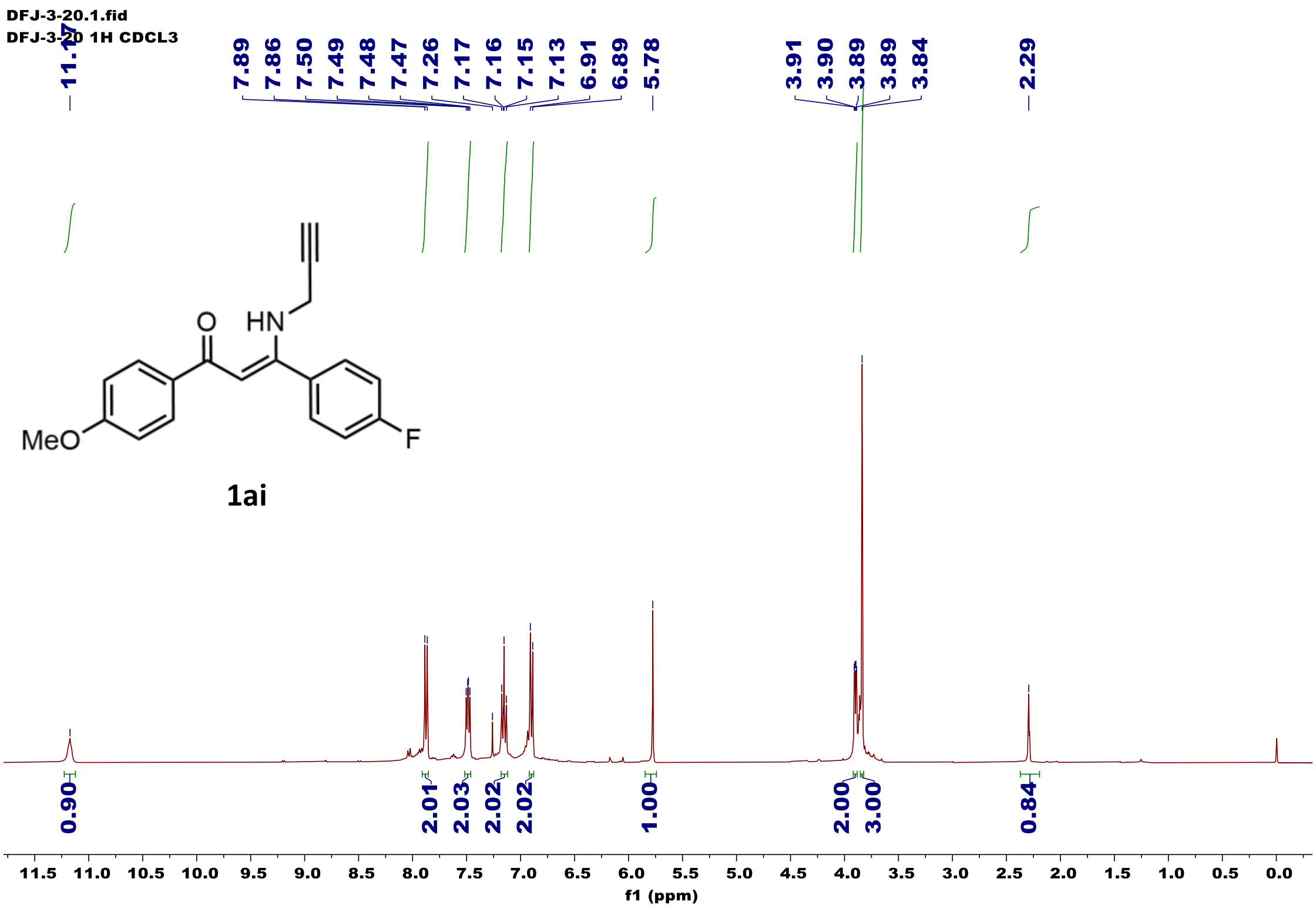

^{13}C NMR spectrum of compound 1ai (101 MHz, $CDCl_3$)

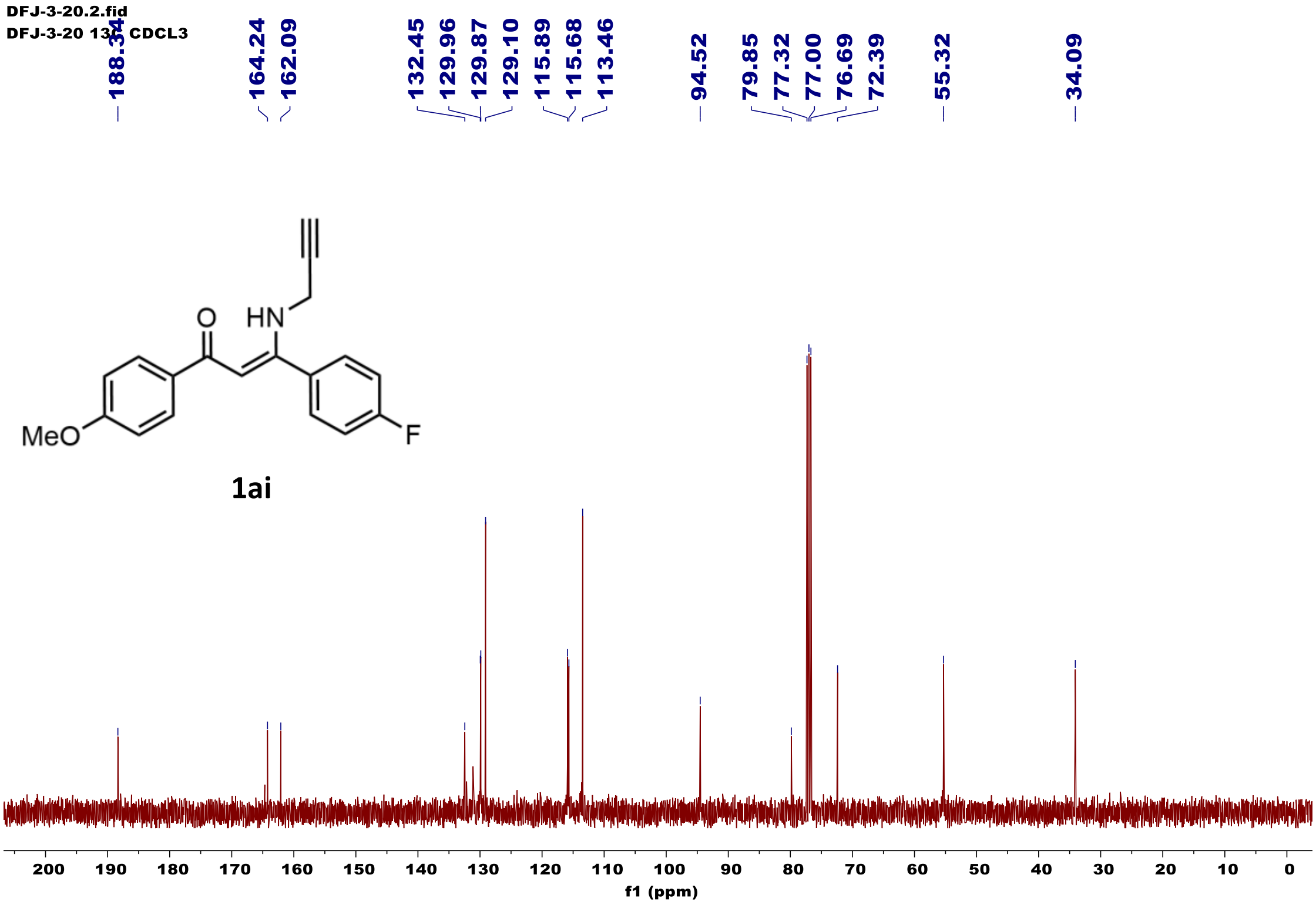

^{1}H NMR spectrum of compound 1aj (400 MHz, $CDCl_3$)

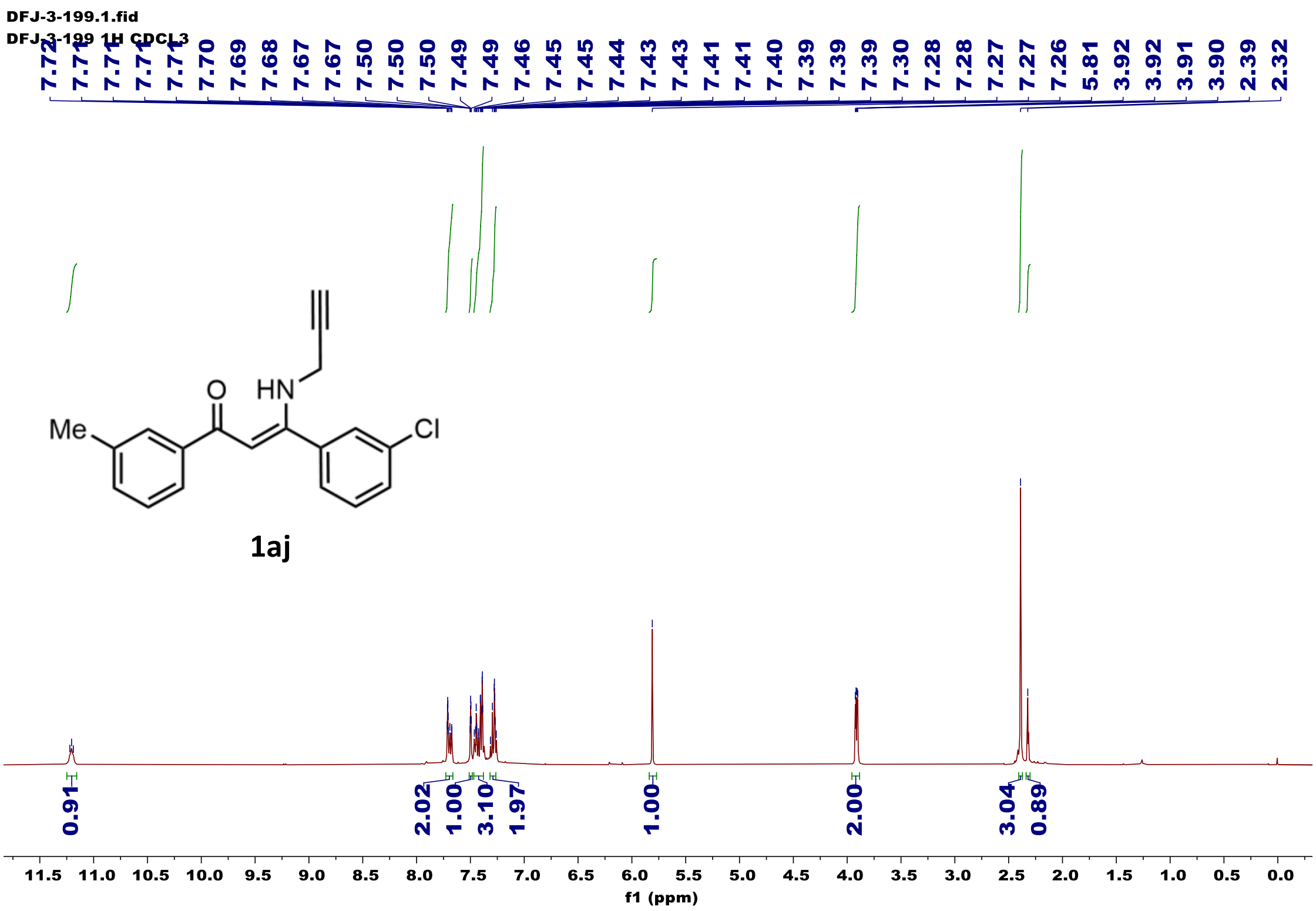

^{13}C NMR spectrum of compound 1aj (101 MHz, $CDCl_3$)

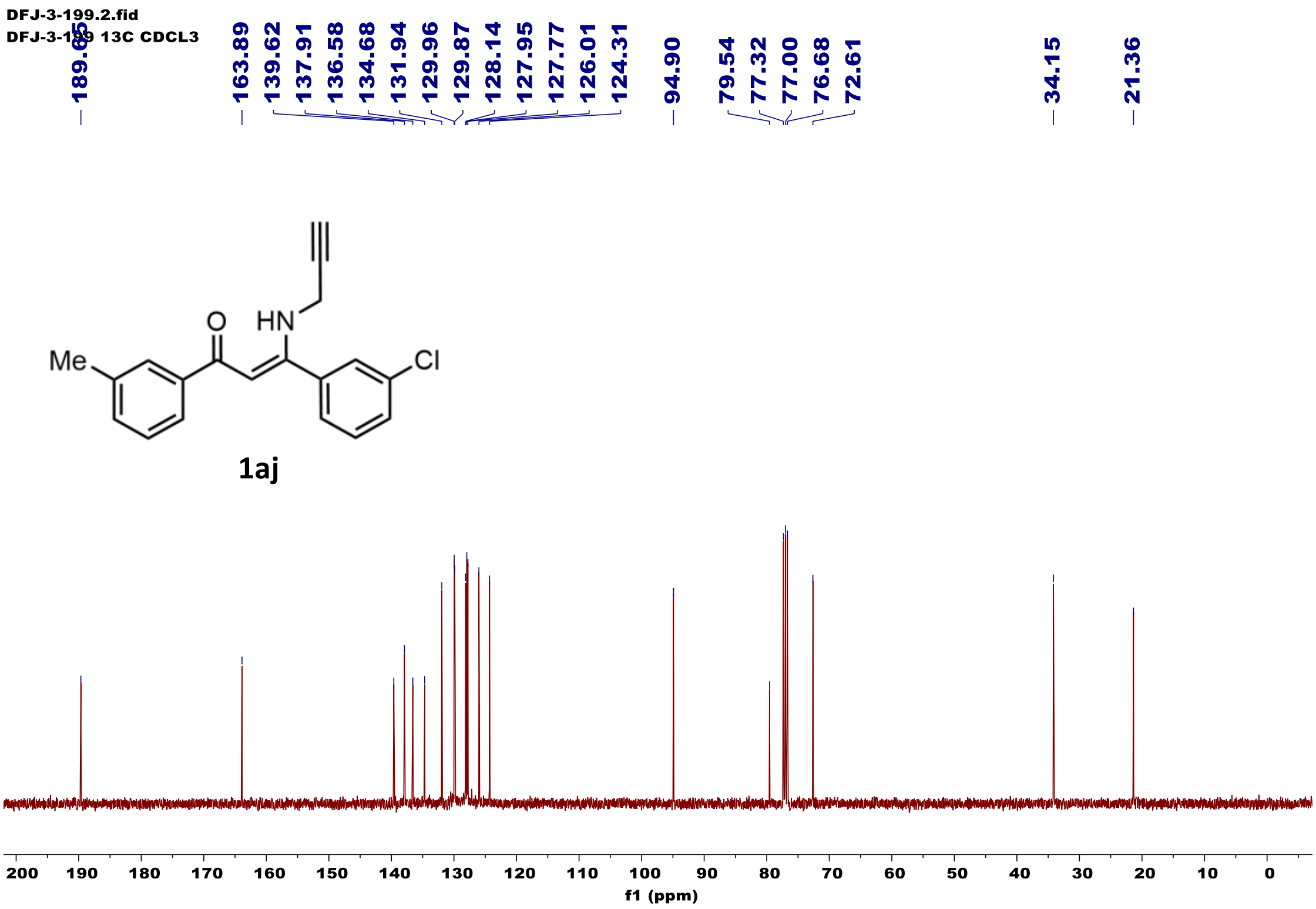

^{1}H NMR spectrum of compound 1ak (400 MHz, $CDCl_3$)

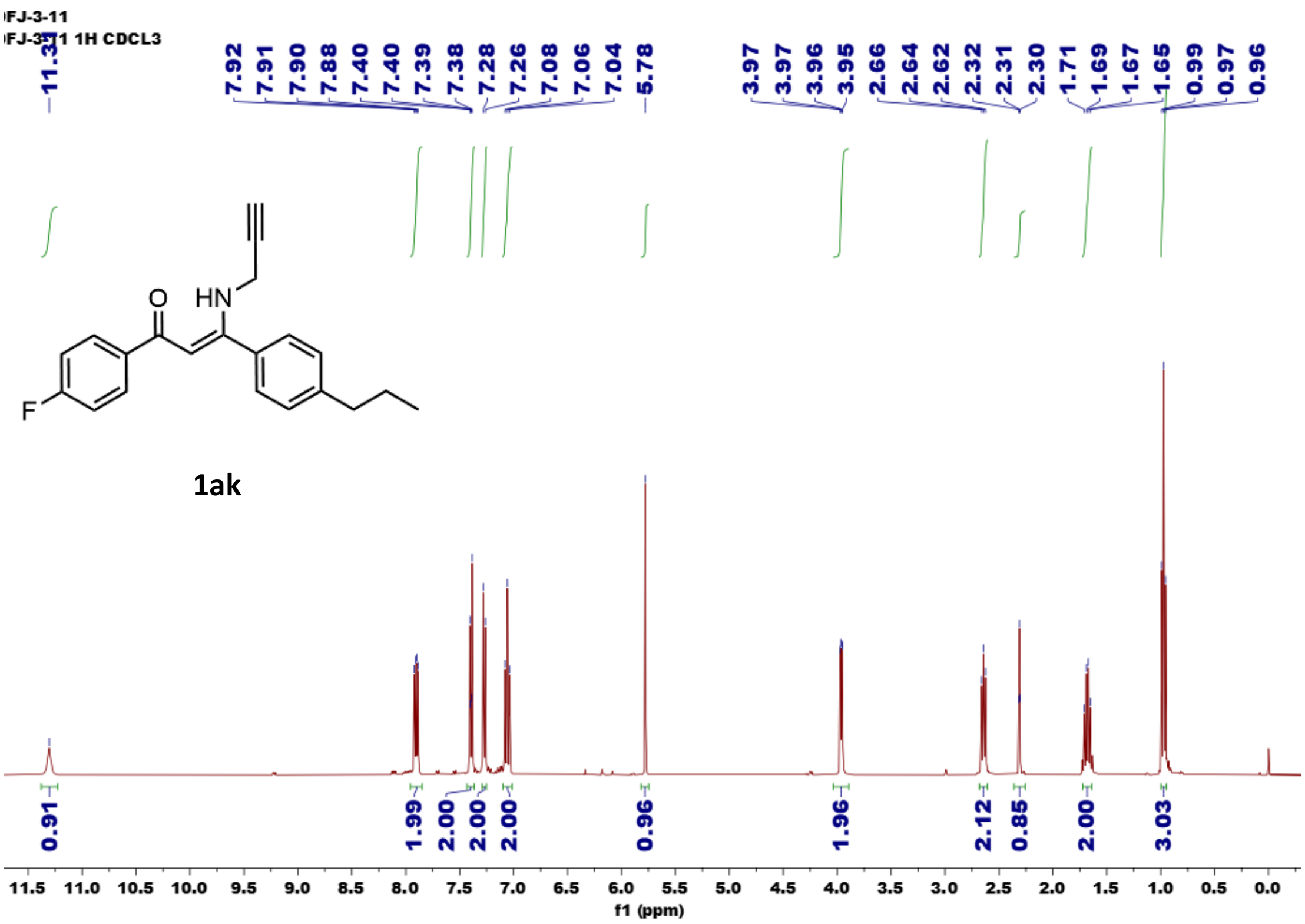

^{13}C NMR spectrum of compound 1ak (101 MHz, $CDCl_3$)

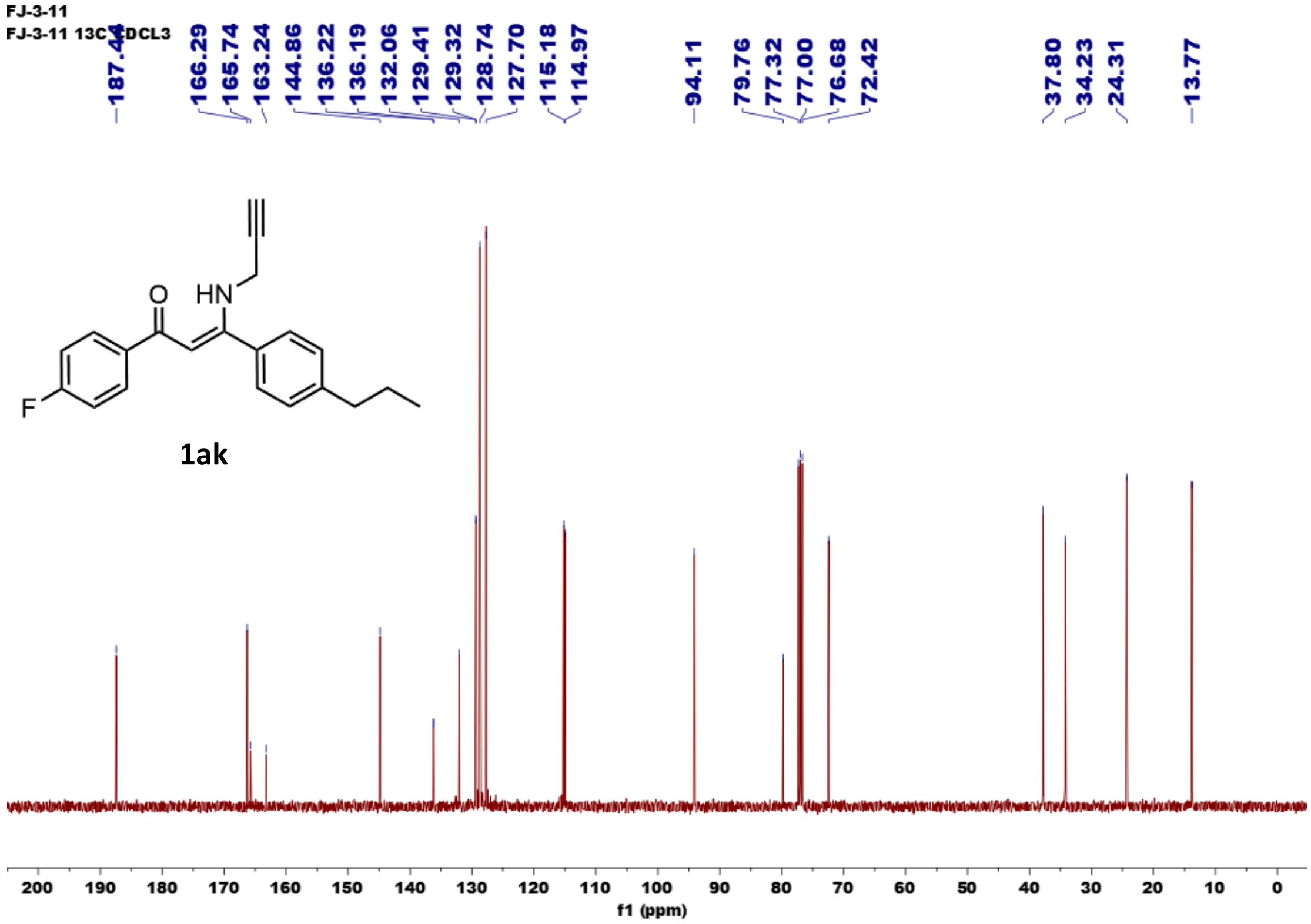

^{1}H NMR spectrum of compound 1al (400 MHz, $CDCl_3$)

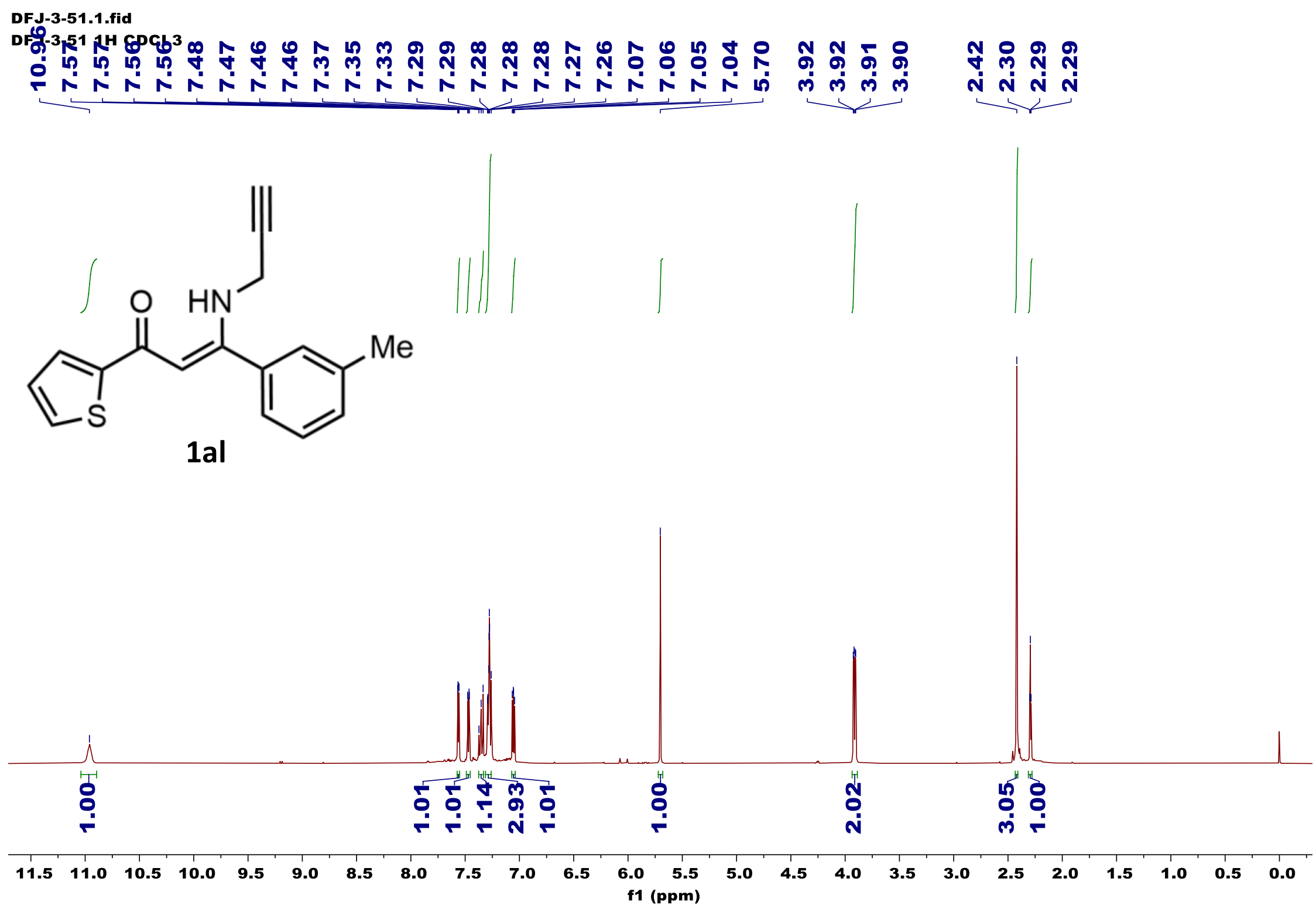

^{13}C NMR spectrum of compound 1al (101 MHz, $CDCl_3$)

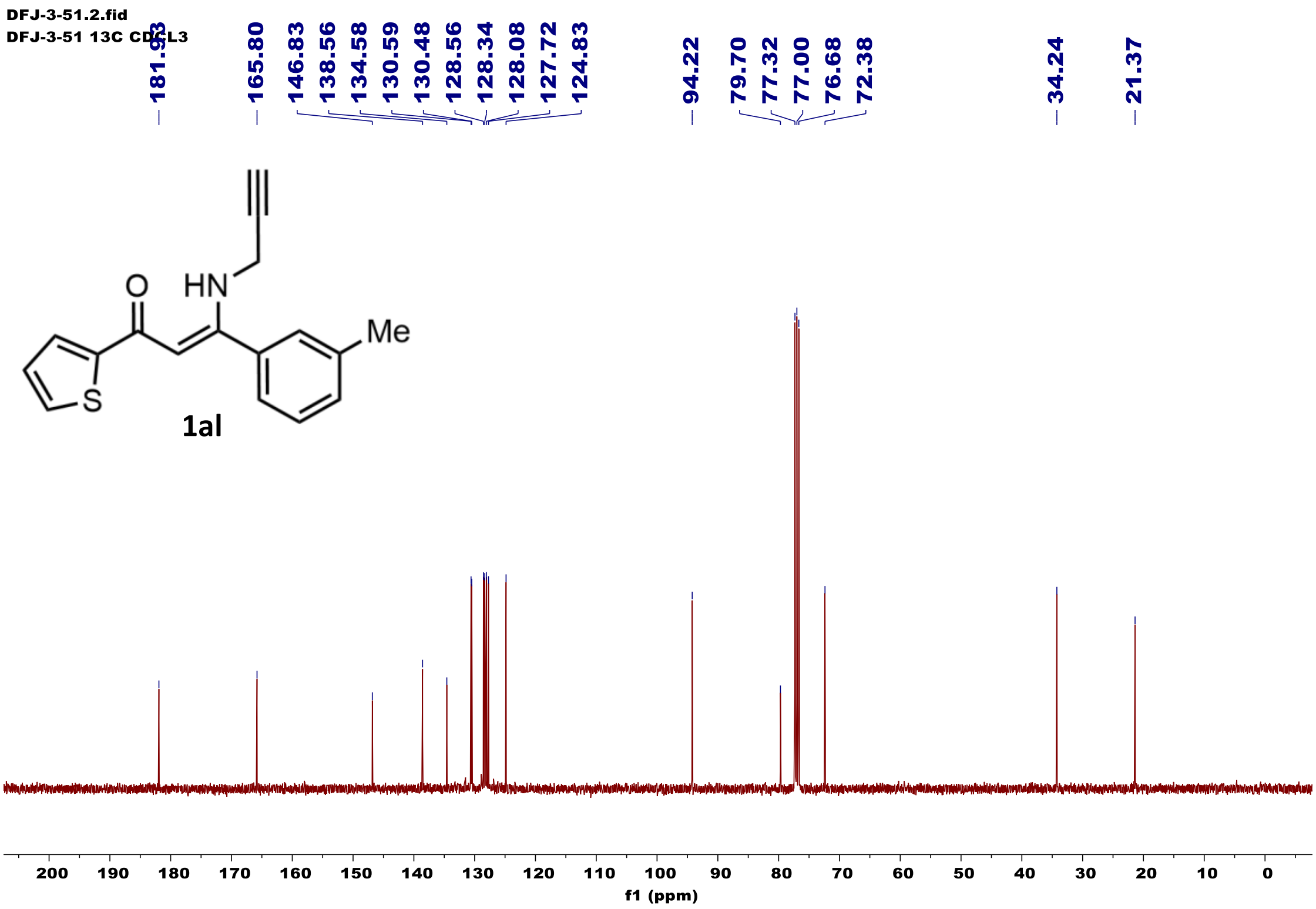

^{1}H NMR spectrum of compound 1am (400 MHz, $CDCl_3$)

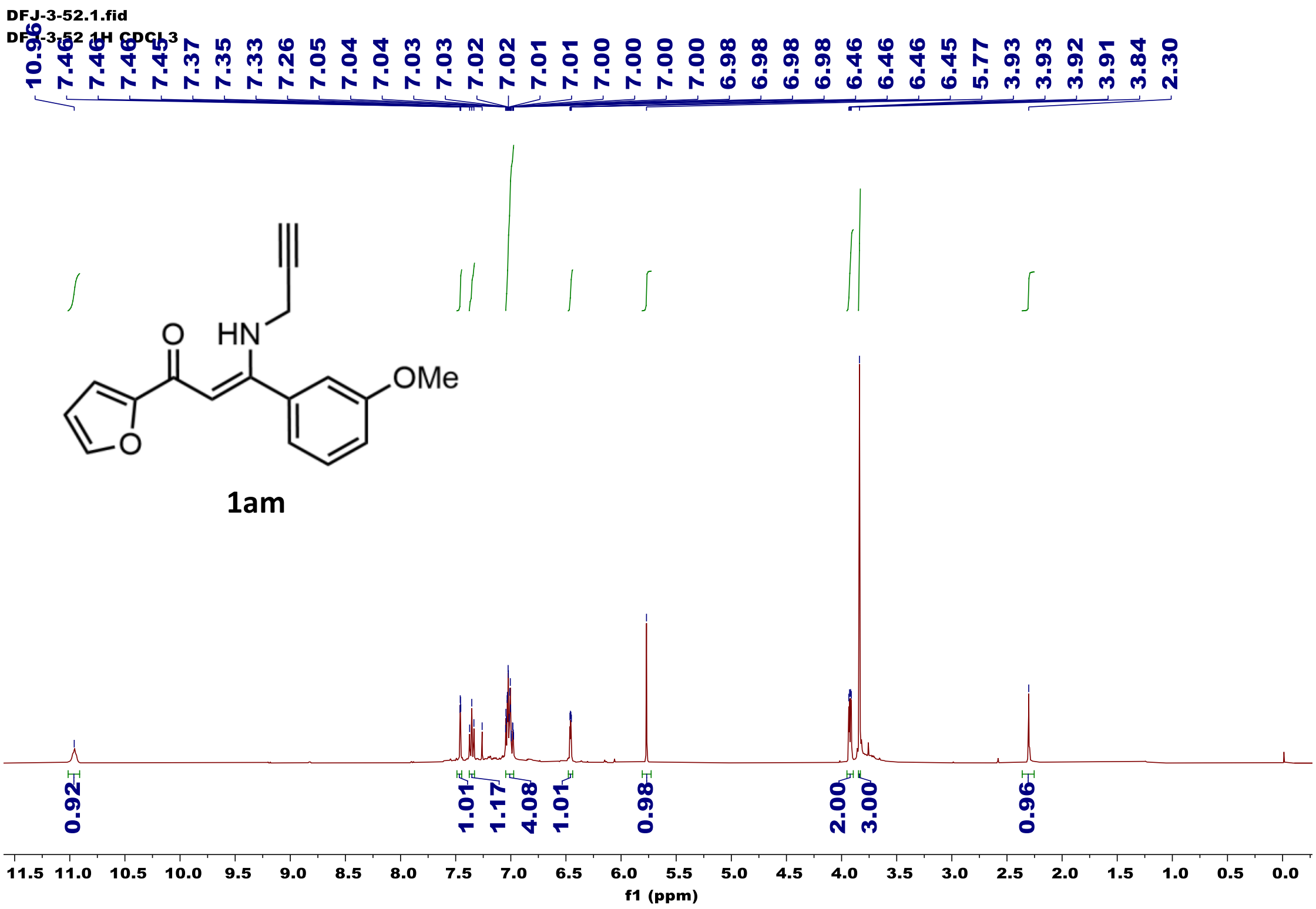

^{13}C NMR spectrum of compound 1am (101 MHz, $CDCl_3$)

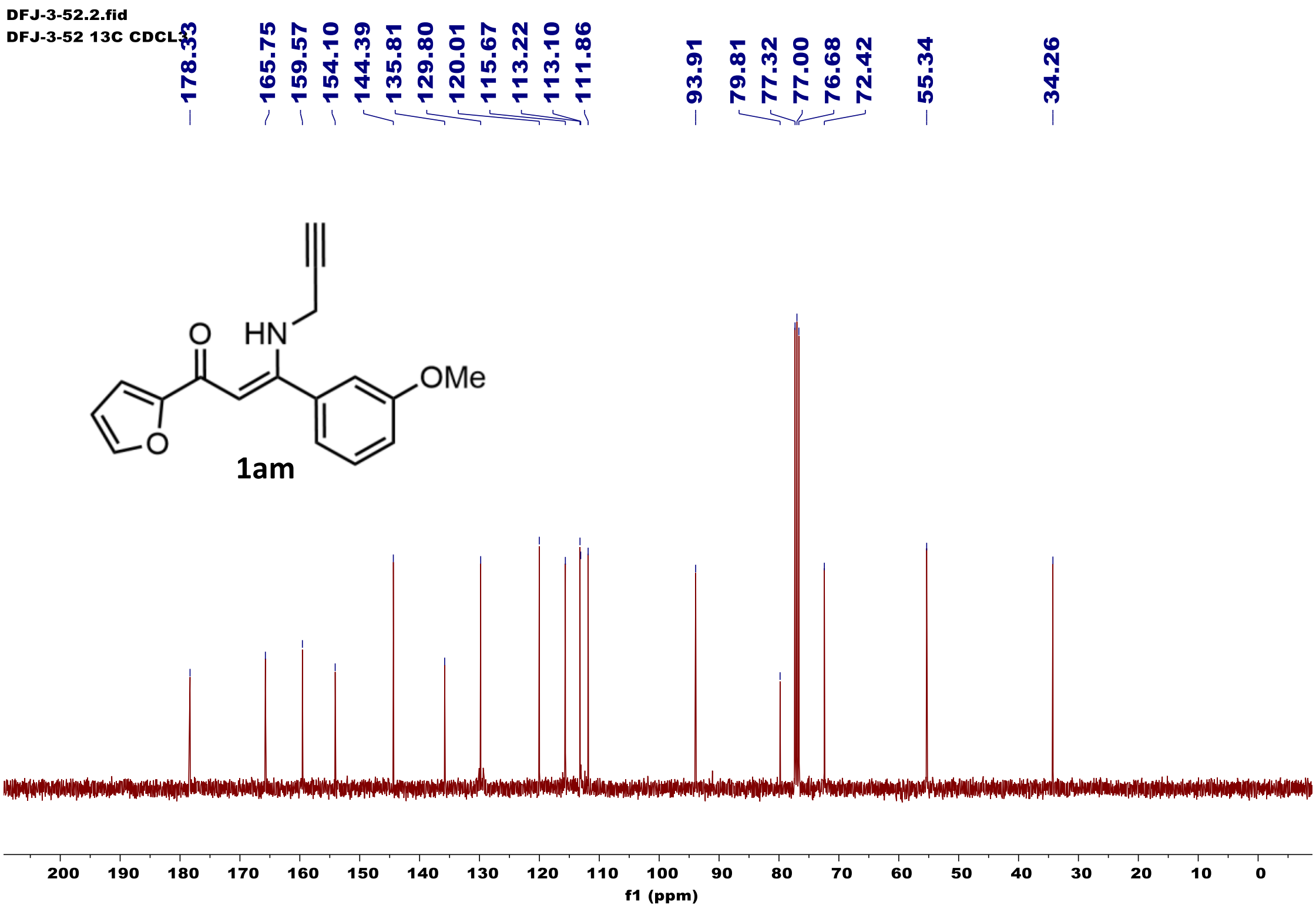

^{1}H NMR spectrum of compound 1an (400 MHz, $CDCl_3$)

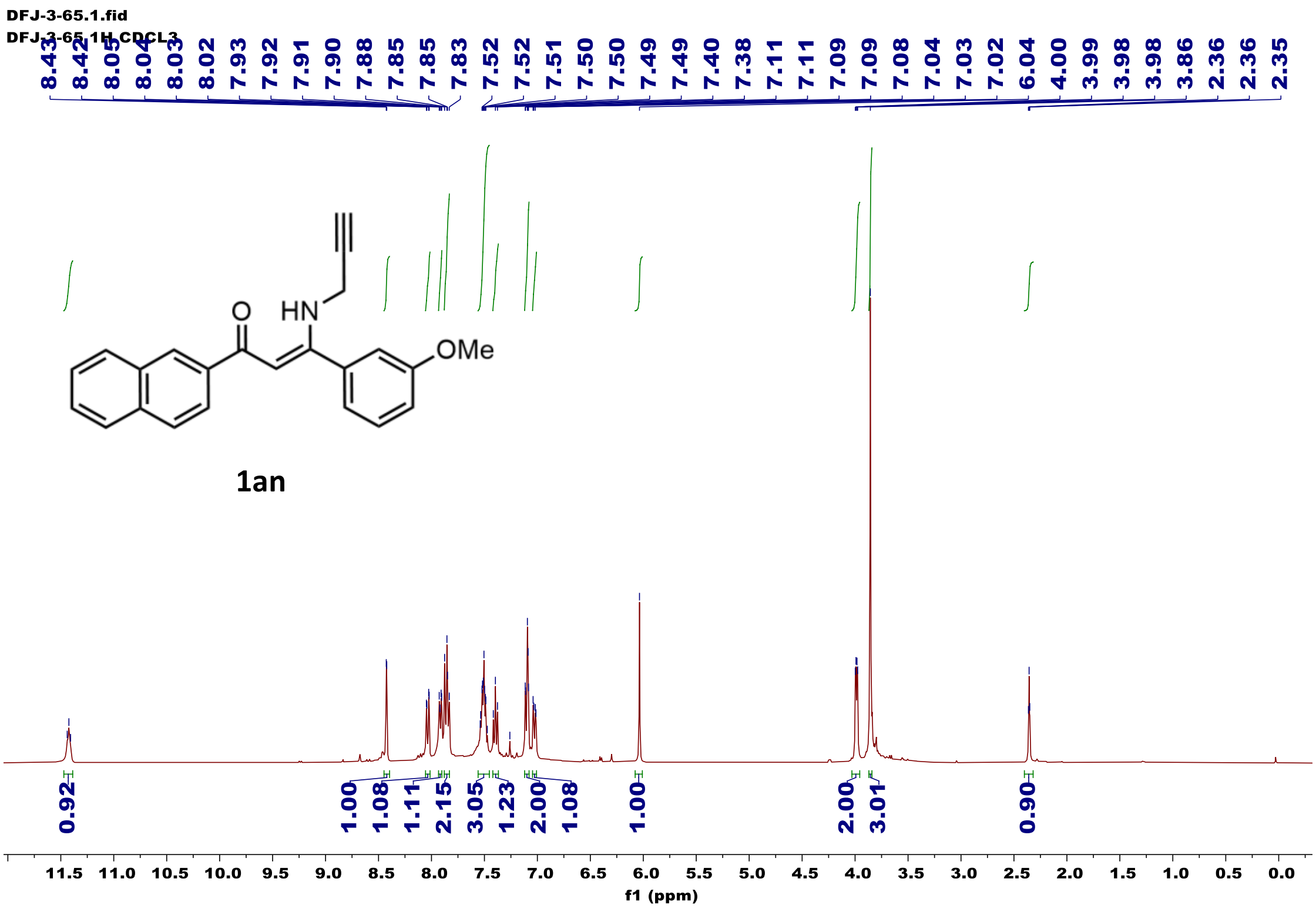

^{13}C NMR spectrum of compound 1an (101 MHz, $CDCl_3$)

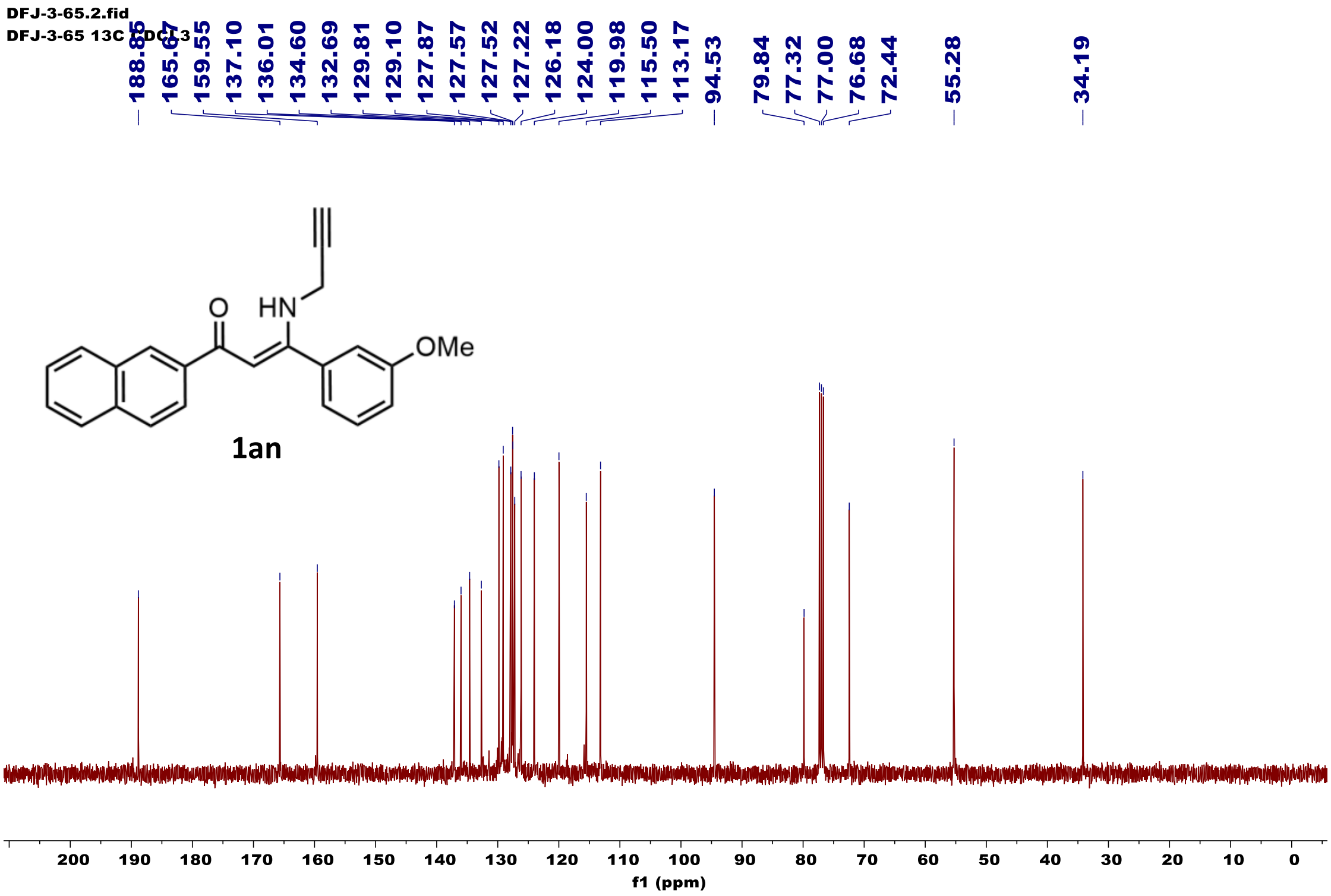

^{1}H NMR spectrum of compound S7 (400 MHz, $CDCl_3$)

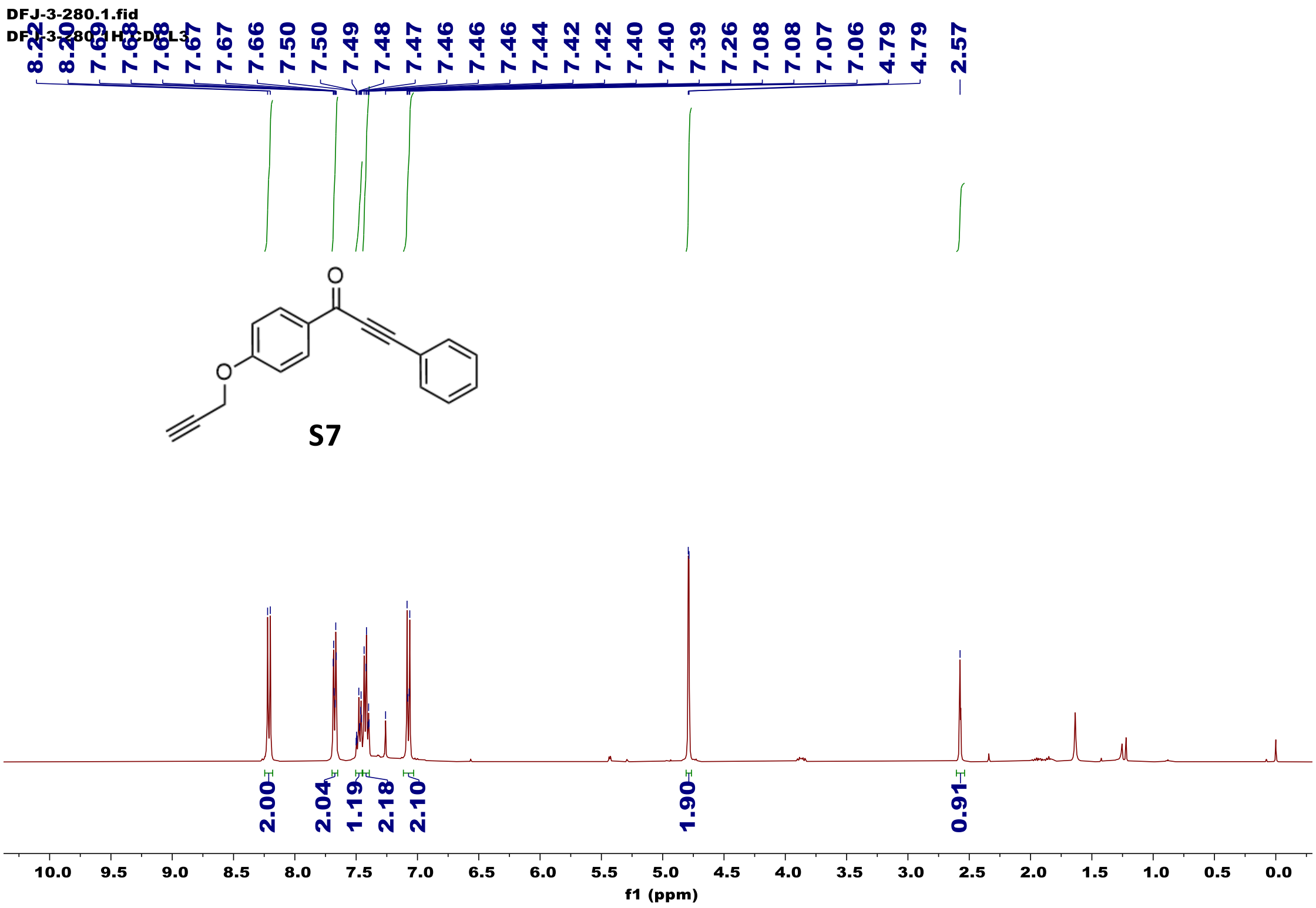

^{13}C NMR spectrum of compound S7 (101 MHz, $CDCl_3$)

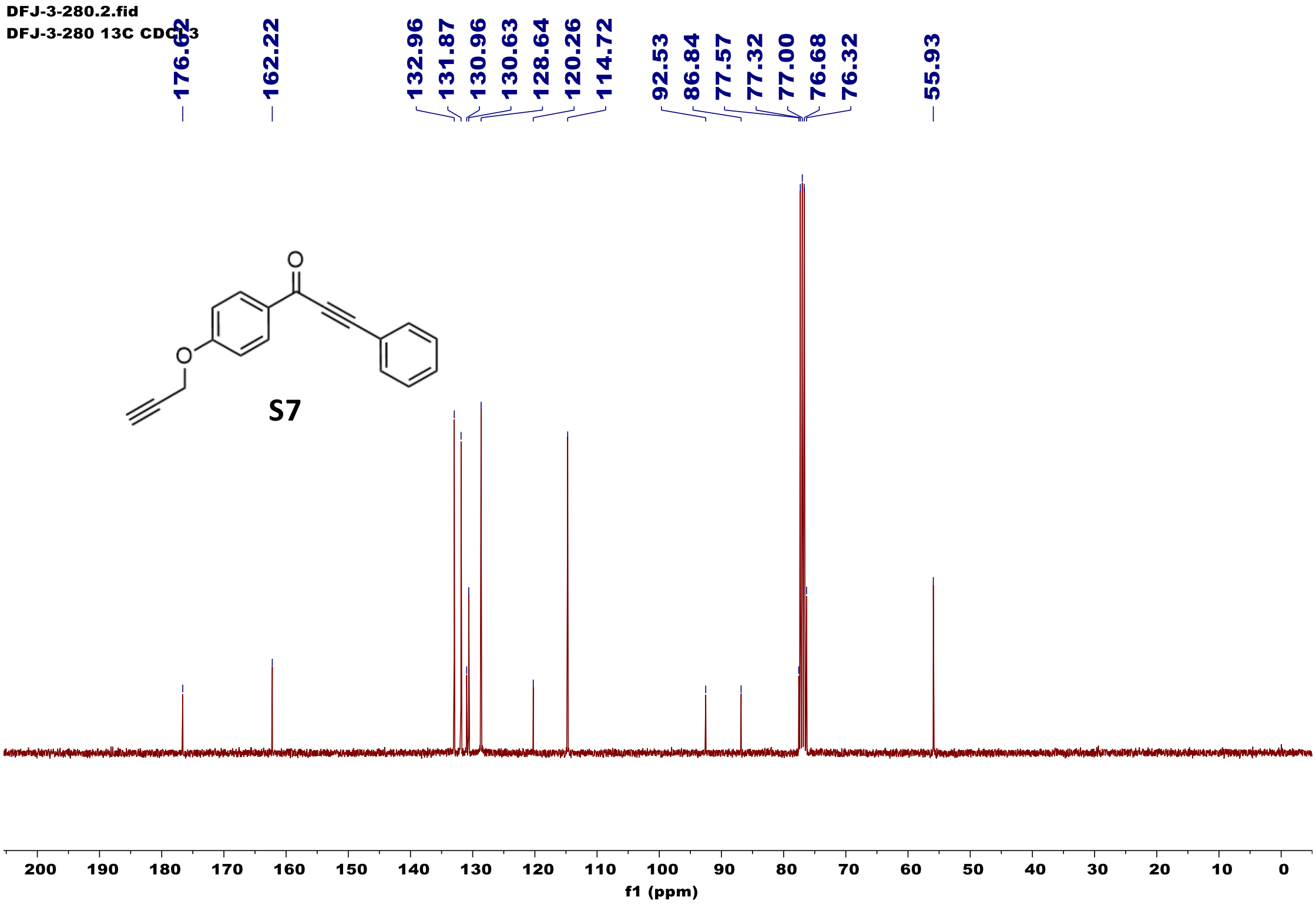

^{1}H NMR spectrum of compound 1i (400 MHz, $CDCl_3$)

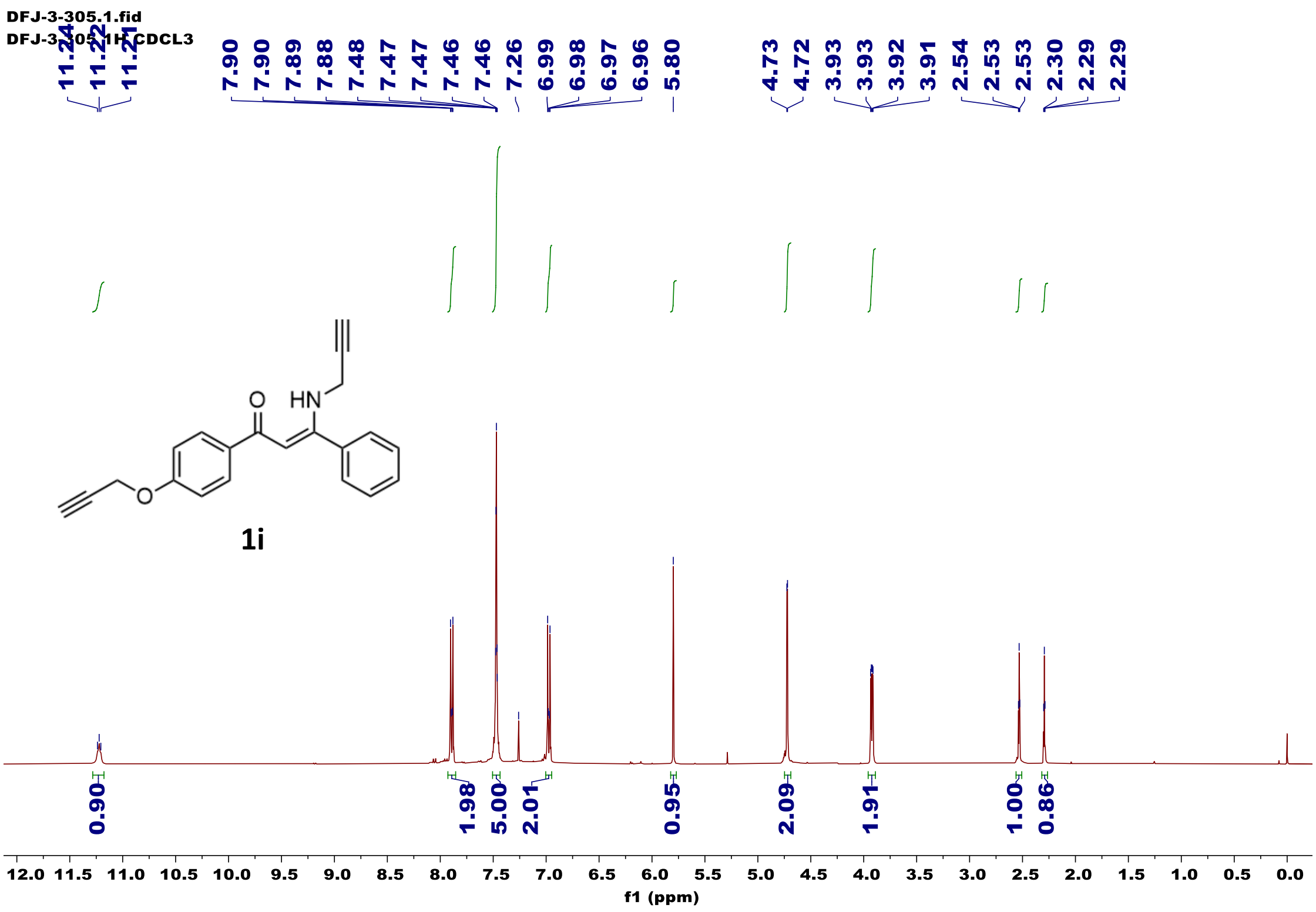

^{13}C NMR spectrum of compound 1i (101 MHz, $CDCl_3$)

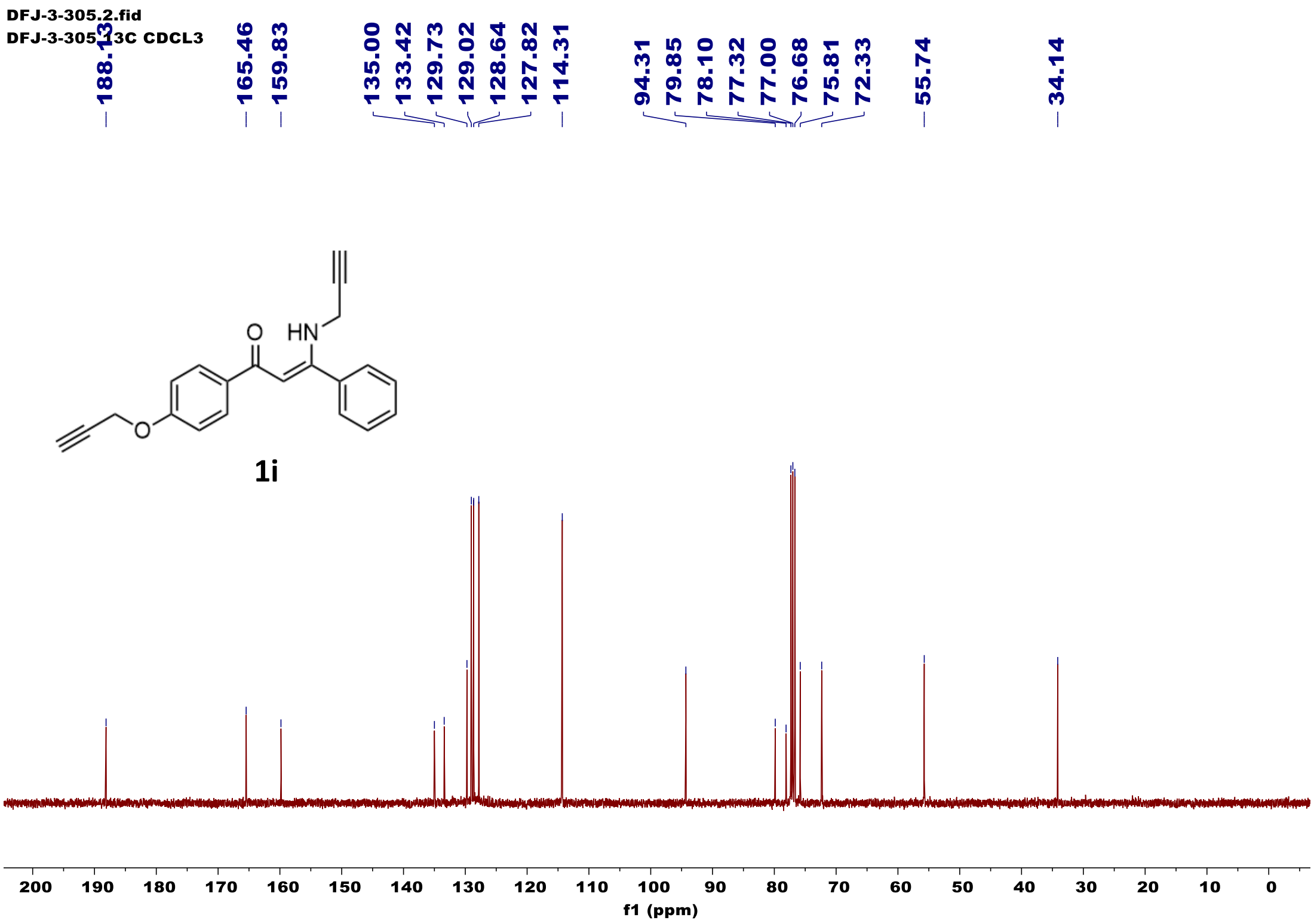

^{1}H NMR spectrum of compound S10 (400 MHz, $CDCl_3$)

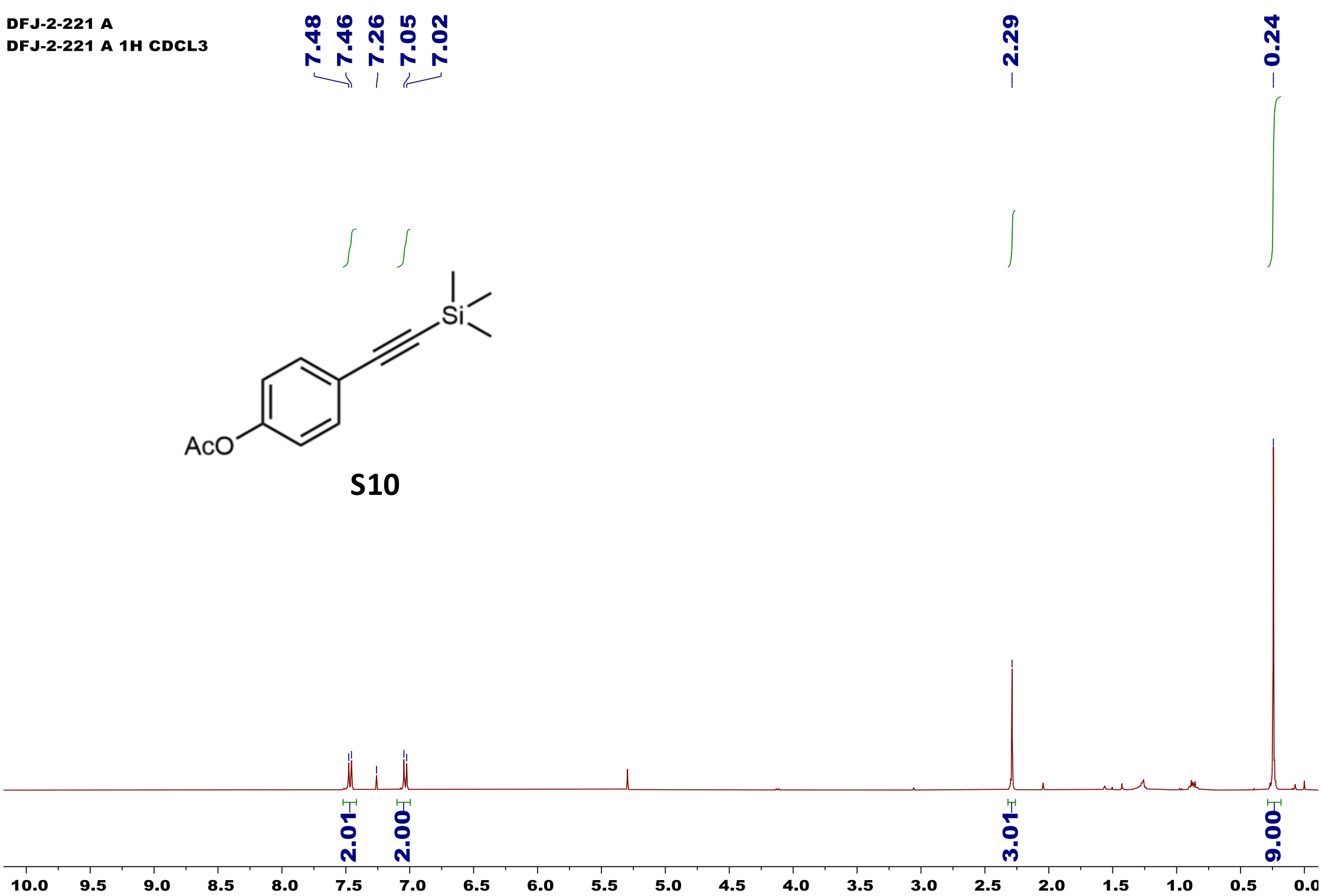

^{13}C NMR spectrum of compound S10 (101 MHz, $CDCl_3$)

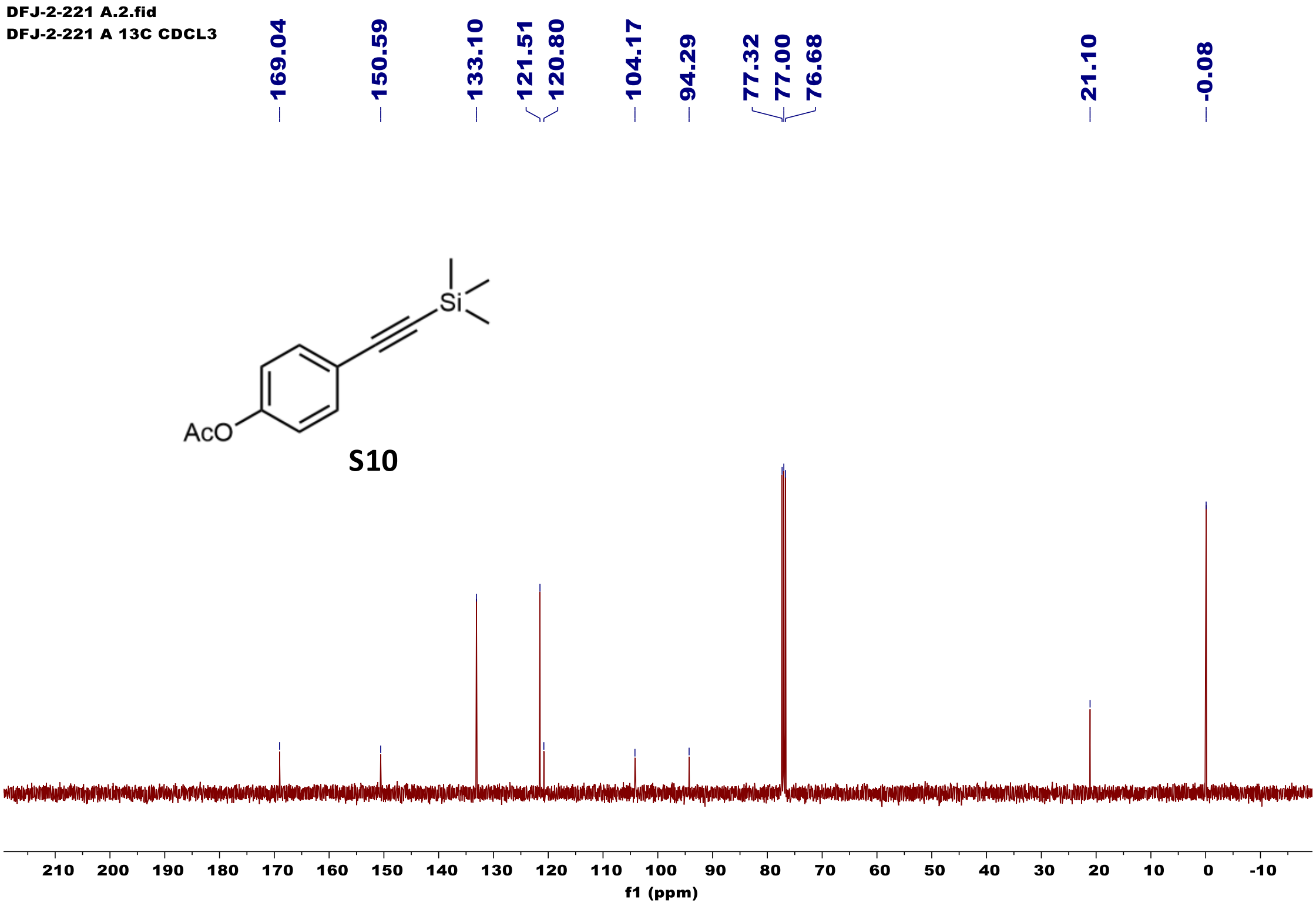

^{1}H NMR spectrum of compound S11 (400 MHz, $CDCl_3$)

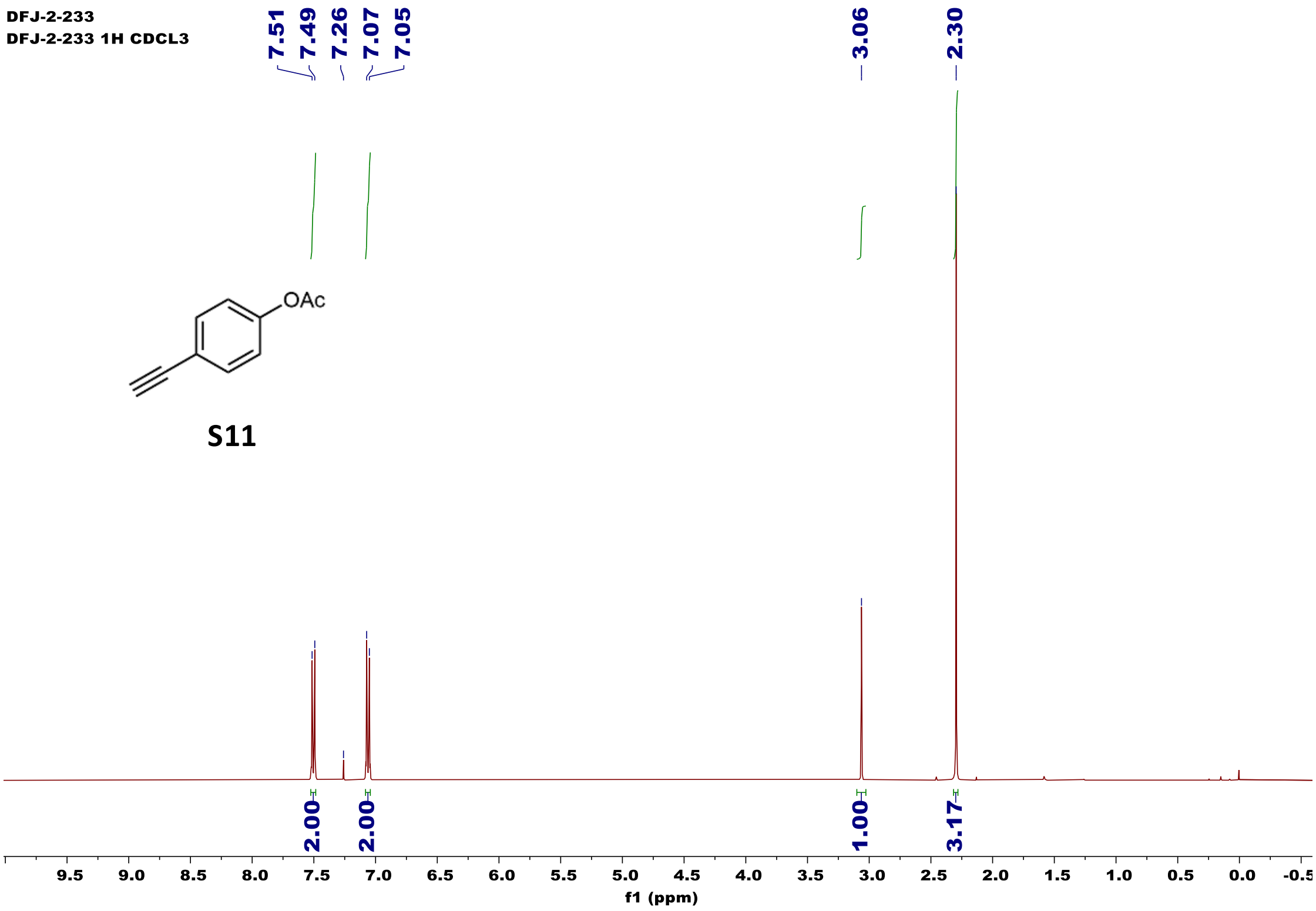

^{13}C NMR spectrum of compound S11 (101 MHz, $CDCl_3$)

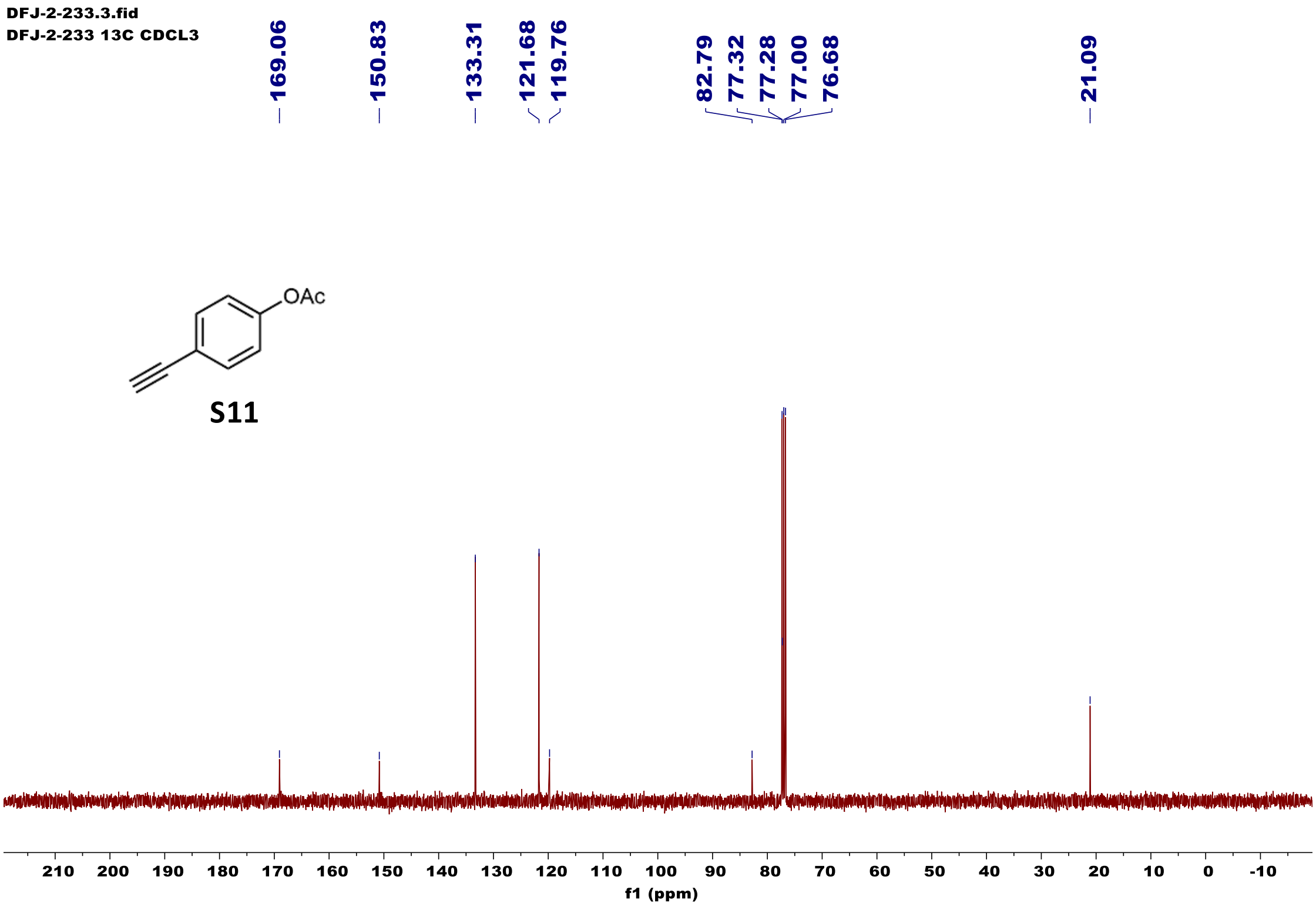

^{1}H NMR spectrum of compound S12 (400 MHz, $CDCl_3$)

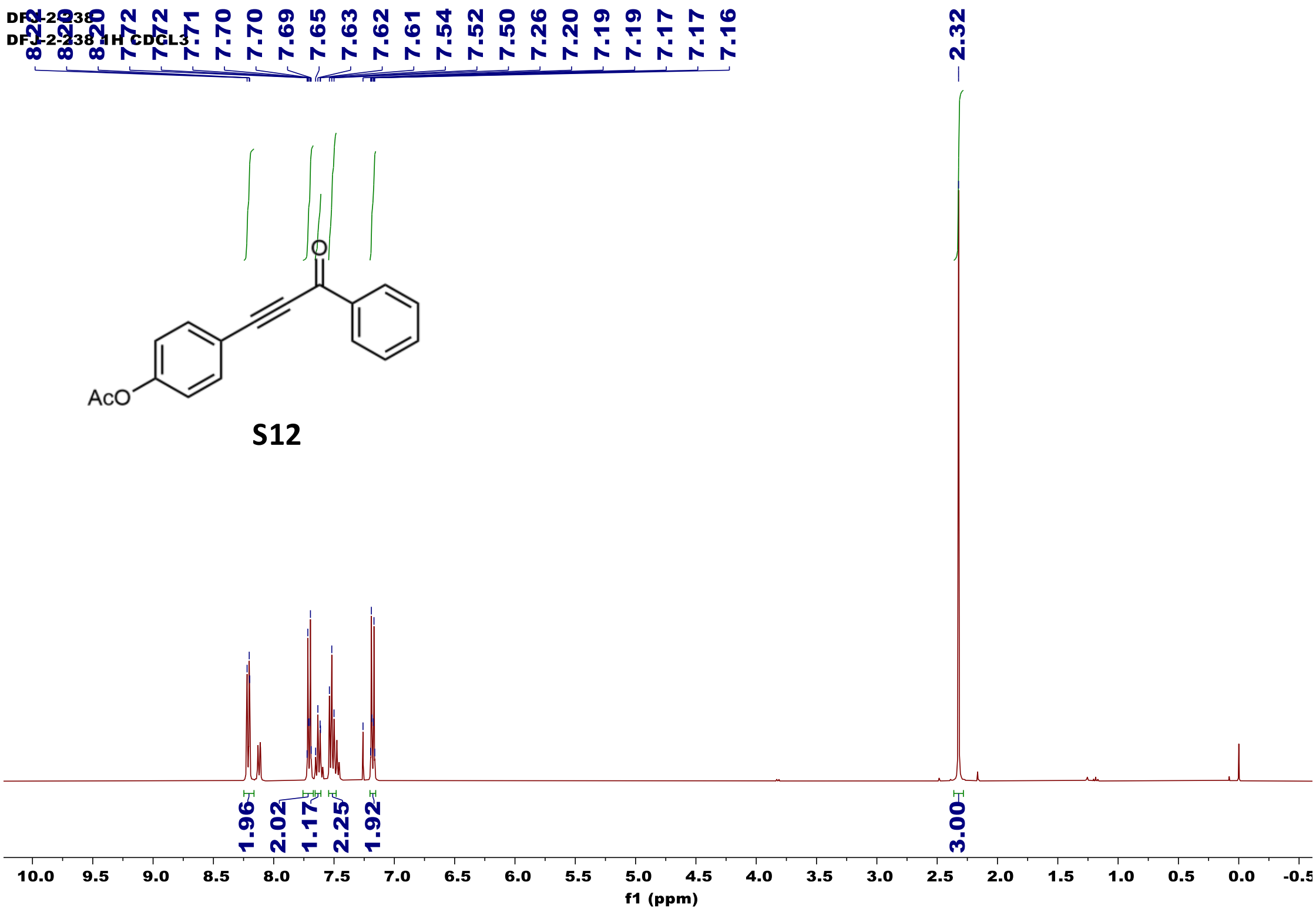

^{13}C NMR spectrum of compound S12 (101 MHz, $CDCl_3$)

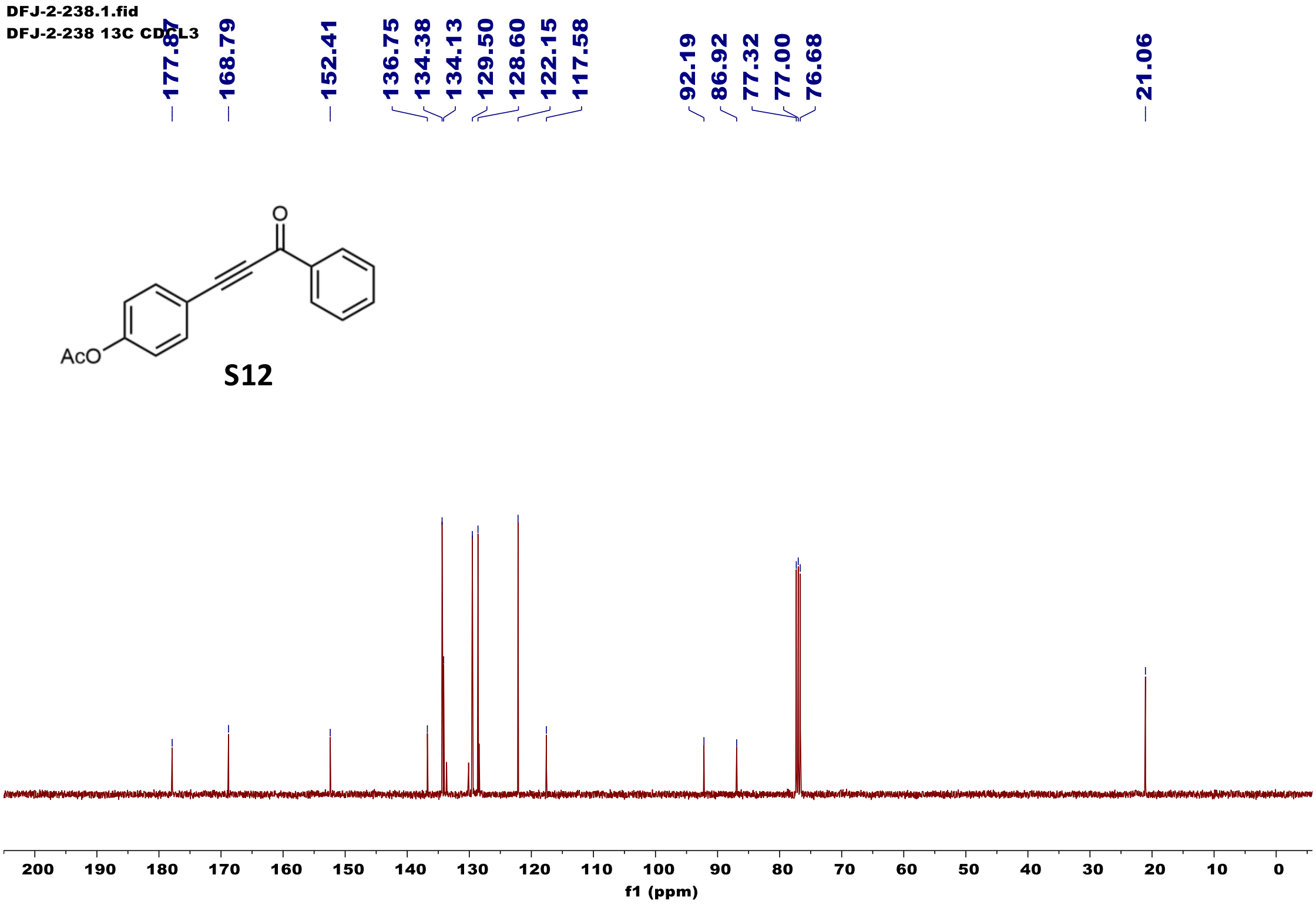

^{1}H NMR spectrum of compound S14 (400 MHz, $CDCl_3$)

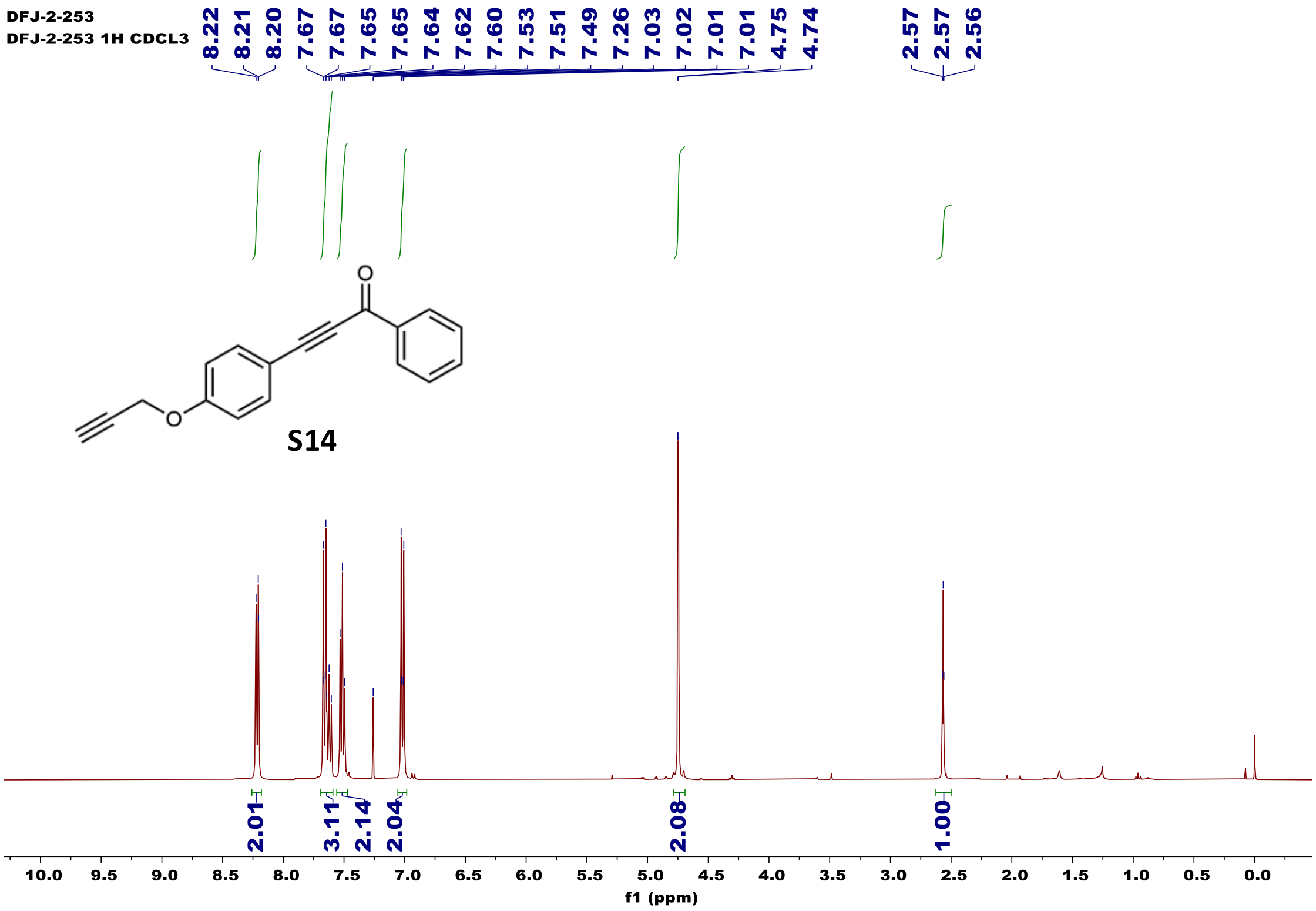

^{13}C NMR spectrum of compound S14 (101 MHz, $CDCl_3$)

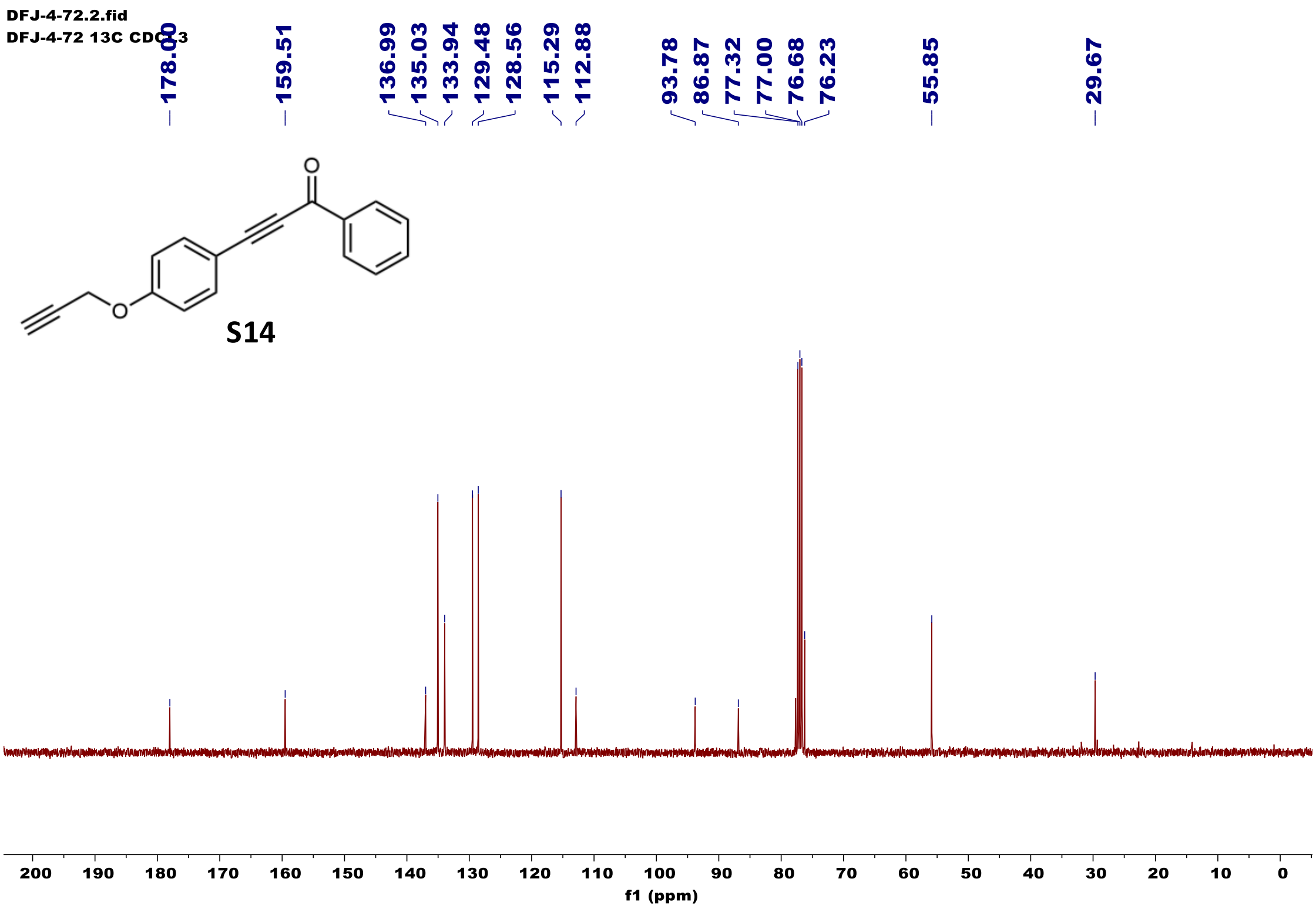

^{1}H NMR spectrum of compound 1ac (400 MHz, $CDCl_3$)

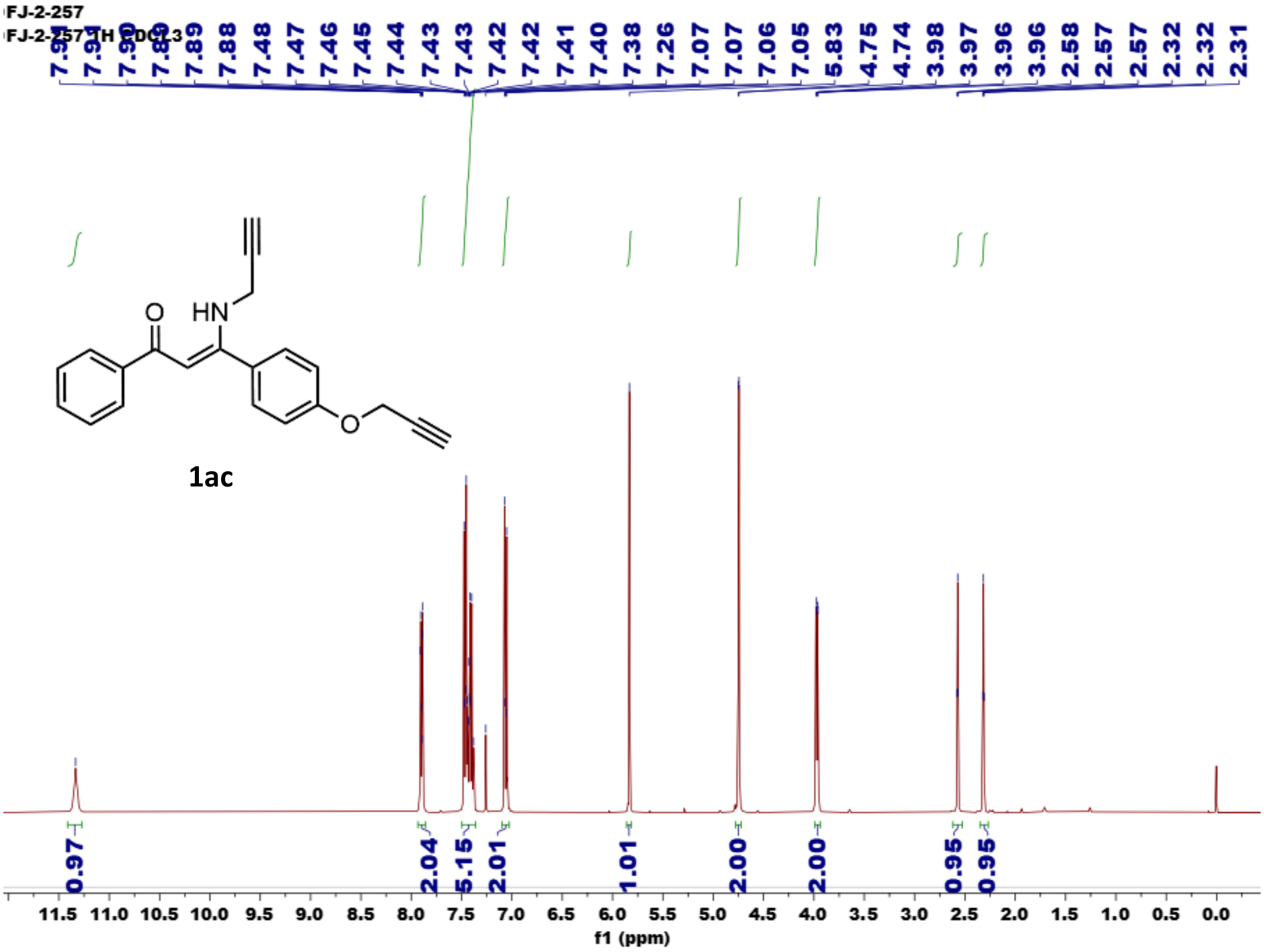

^{13}C NMR spectrum of compound 1ac (101 MHz, $CDCl_3$)

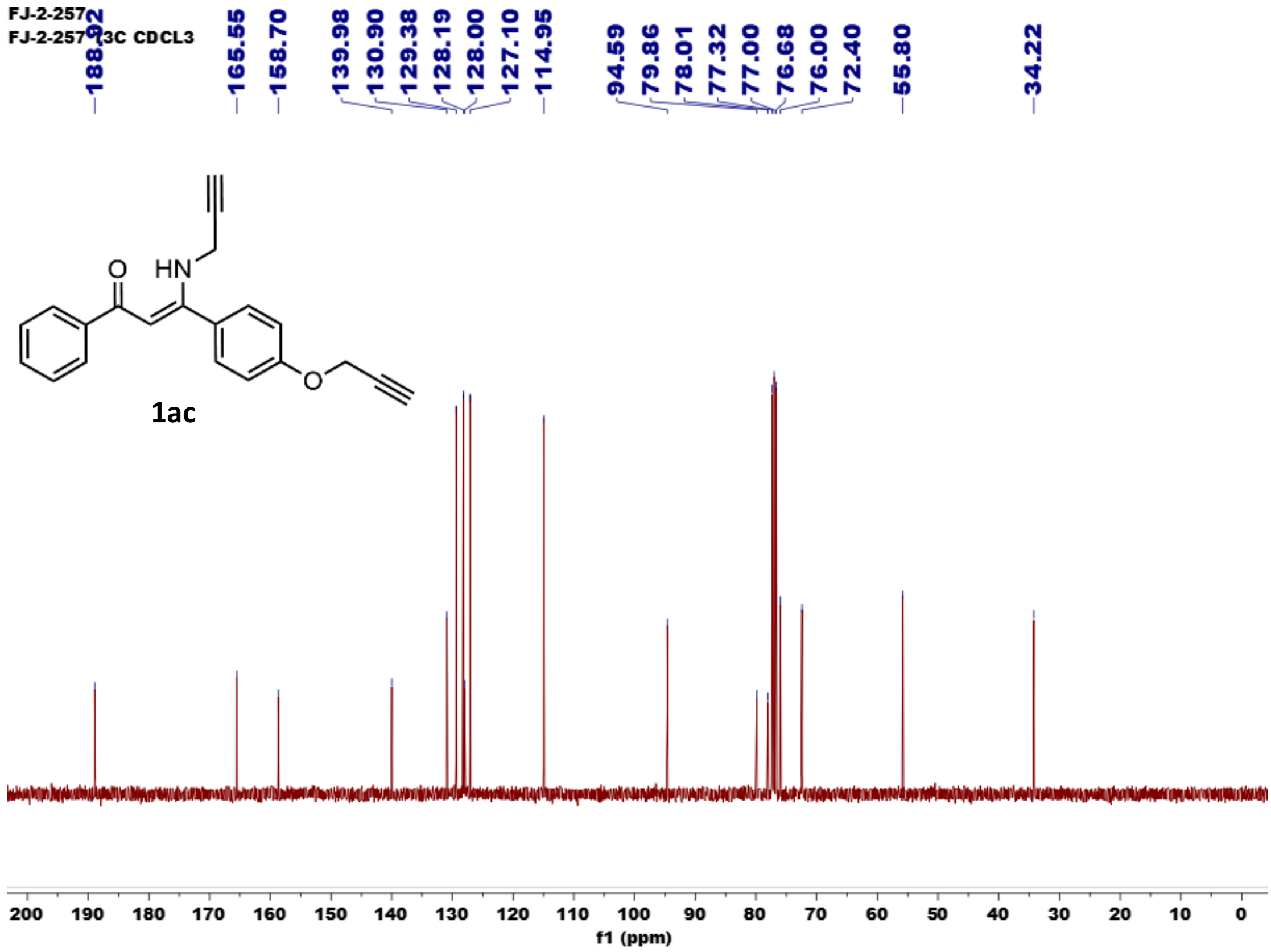

^{1}H NMR spectrum of compound 2a (400 MHz, $CDCl_3$)

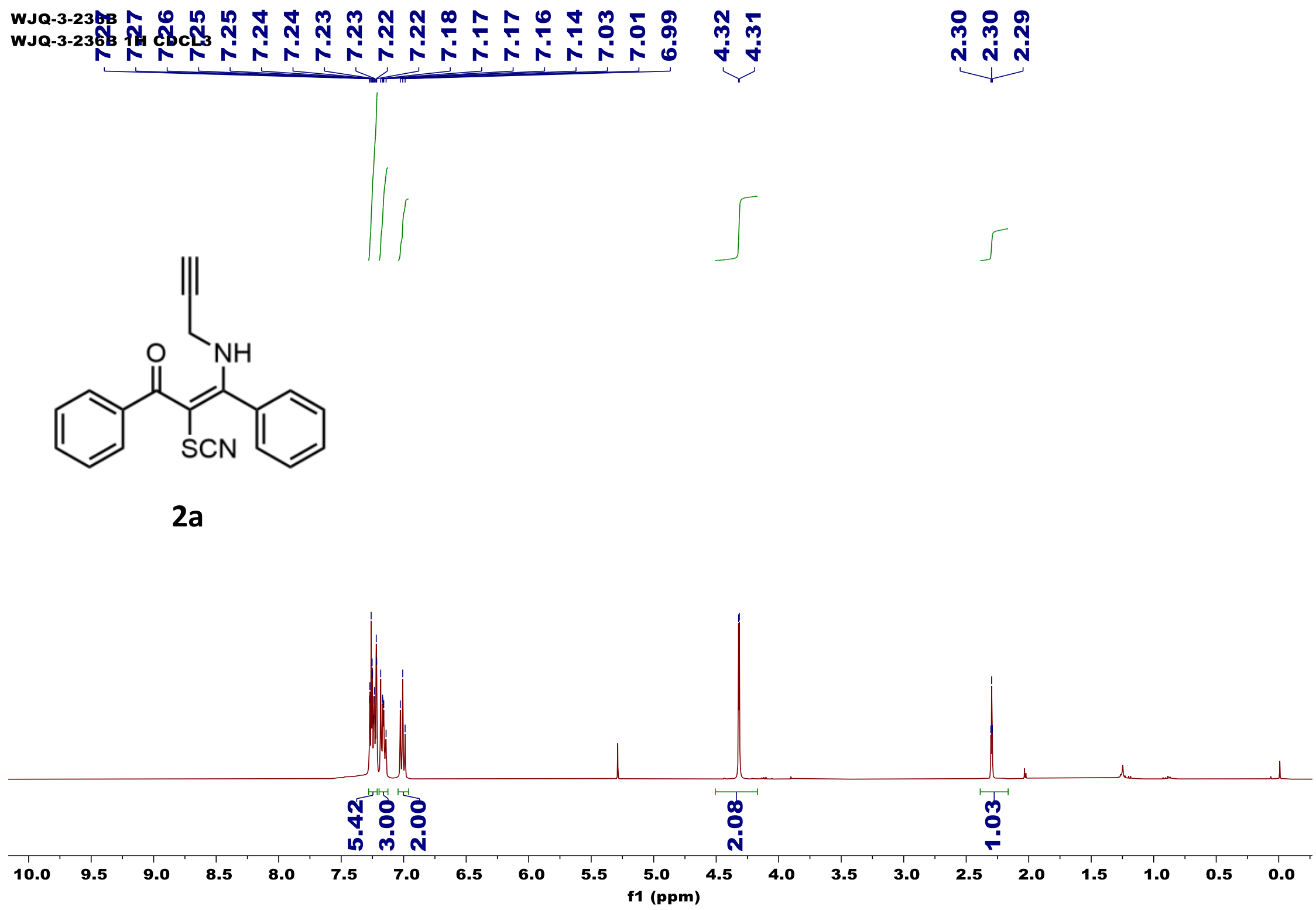

^{13}C NMR spectrum of compound 2a (101 MHz, $CDCl_3$)

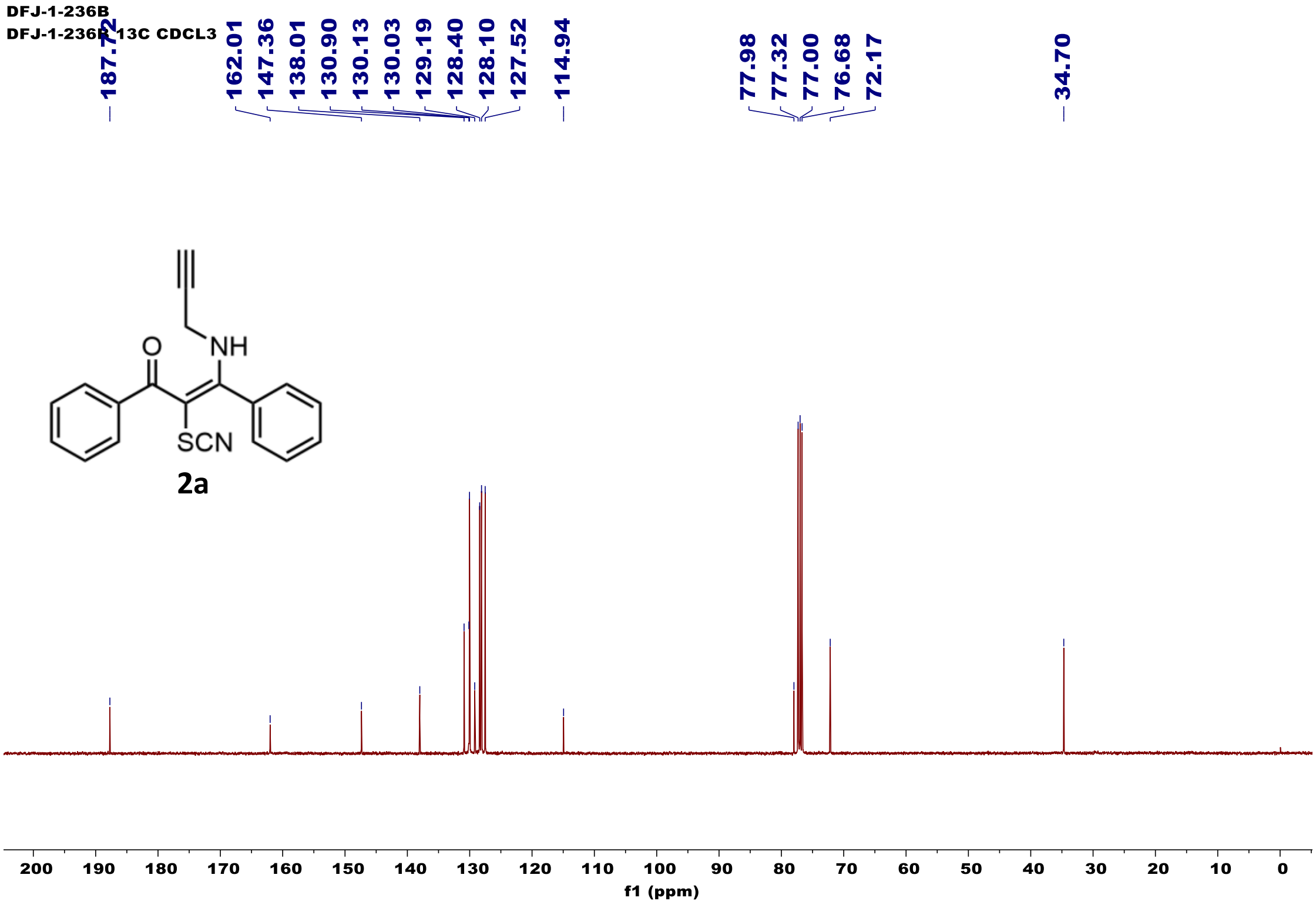

^{1}H NMR spectrum of compound 3a (400 MHz, $CDCl_3$)

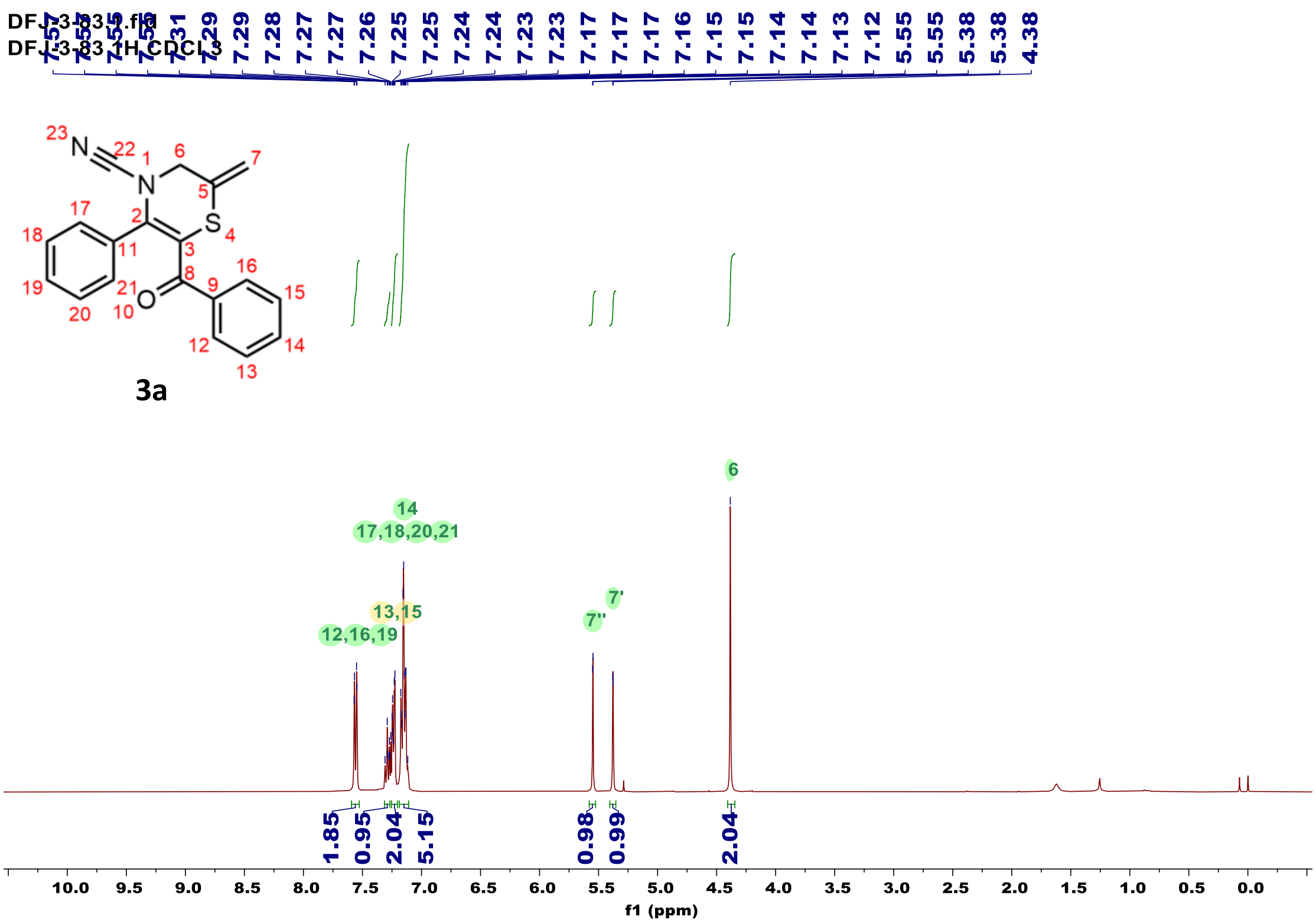

^{13}C NMR spectrum of compound 3a (101 MHz, $CDCl_3$)

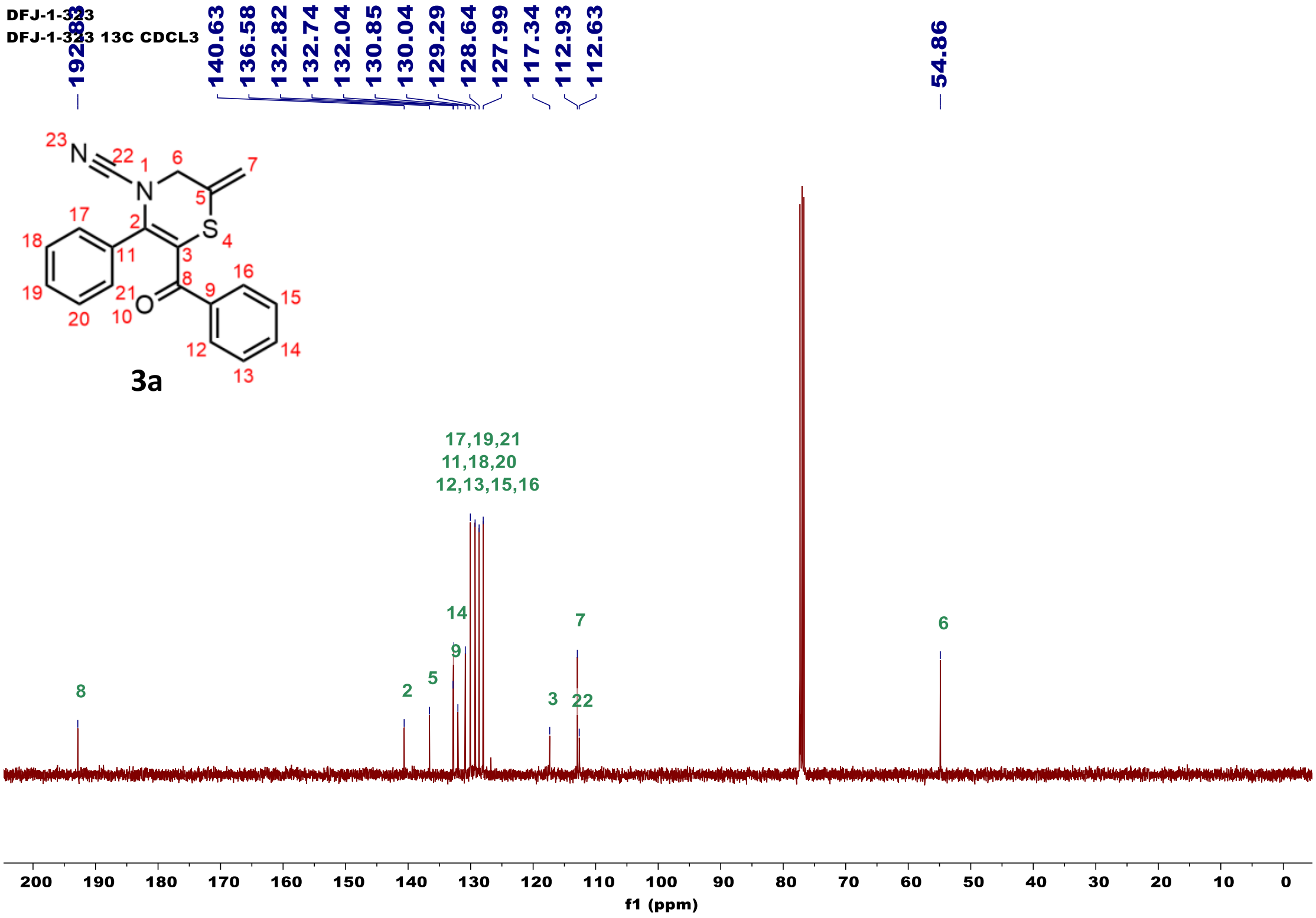

^{1}H NMR spectrum of compound 3b (400 MHz, $CDCl_3$)

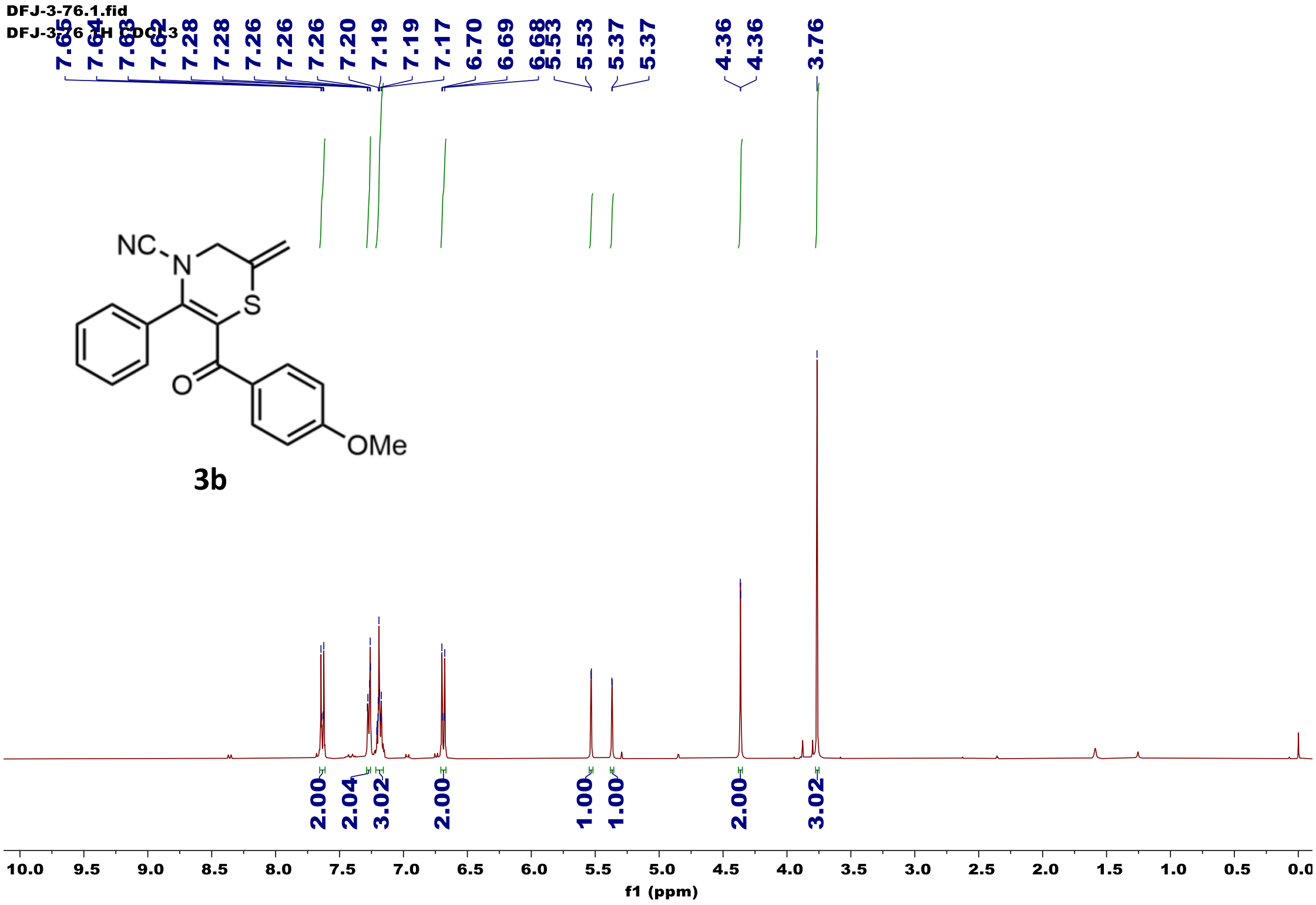

^{13}C NMR spectrum of compound 3b (101 MHz, $CDCl_3$)

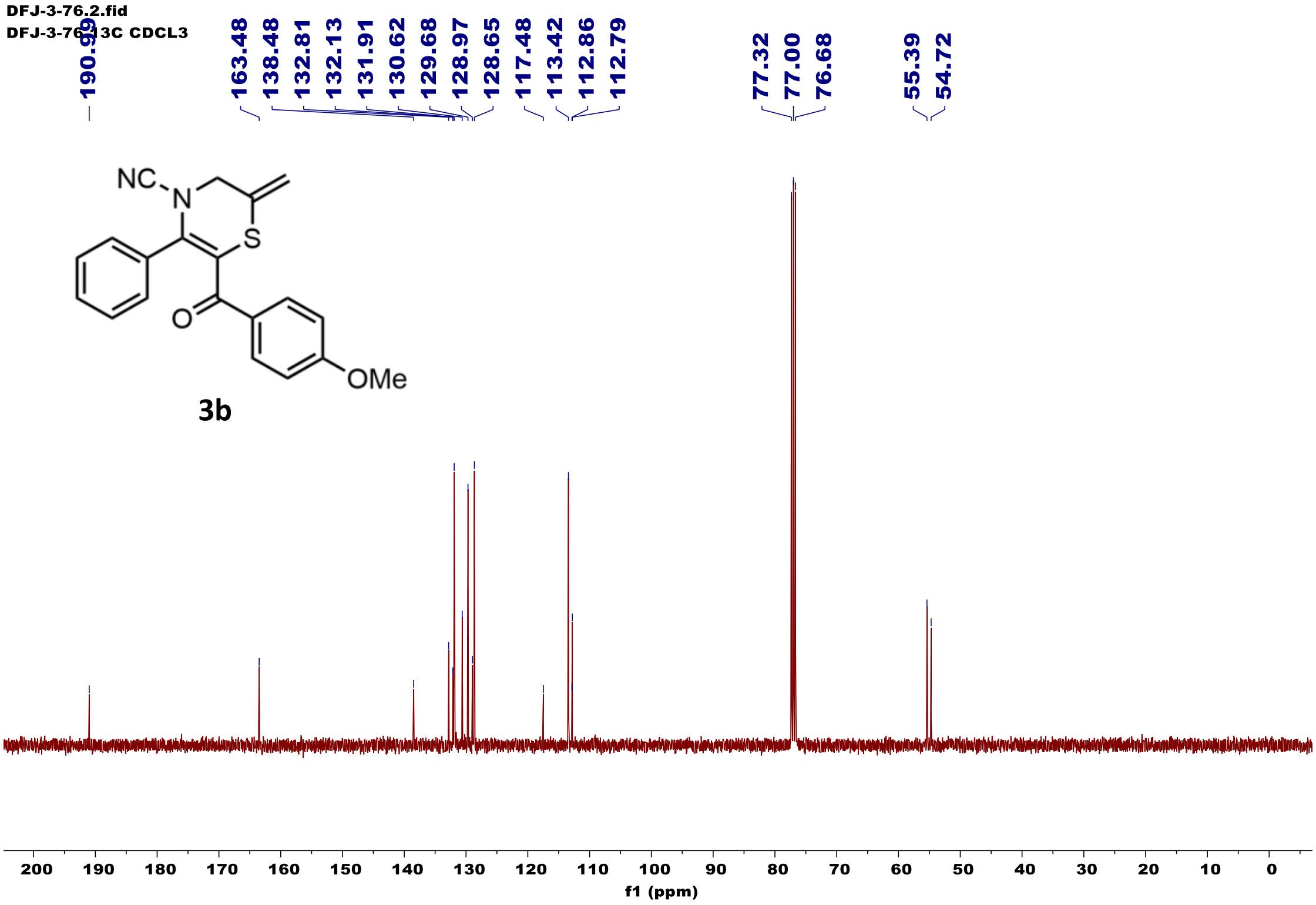

¹H NMR spectrum of compound 3c (400 MHz, $CDCl_3$)

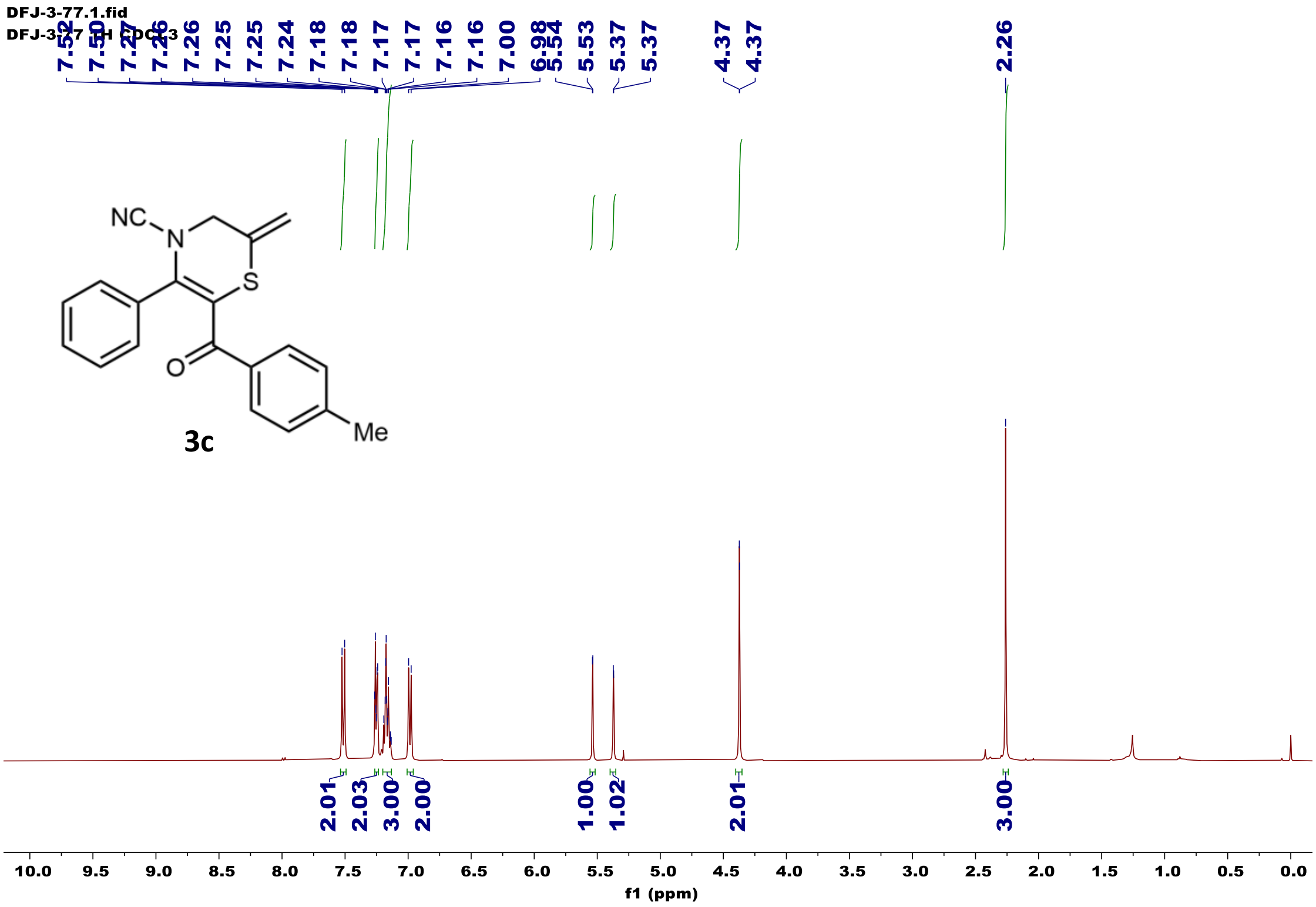

^{13}C NMR spectrum of compound 3c (101 MHz, CDCl$_3$)

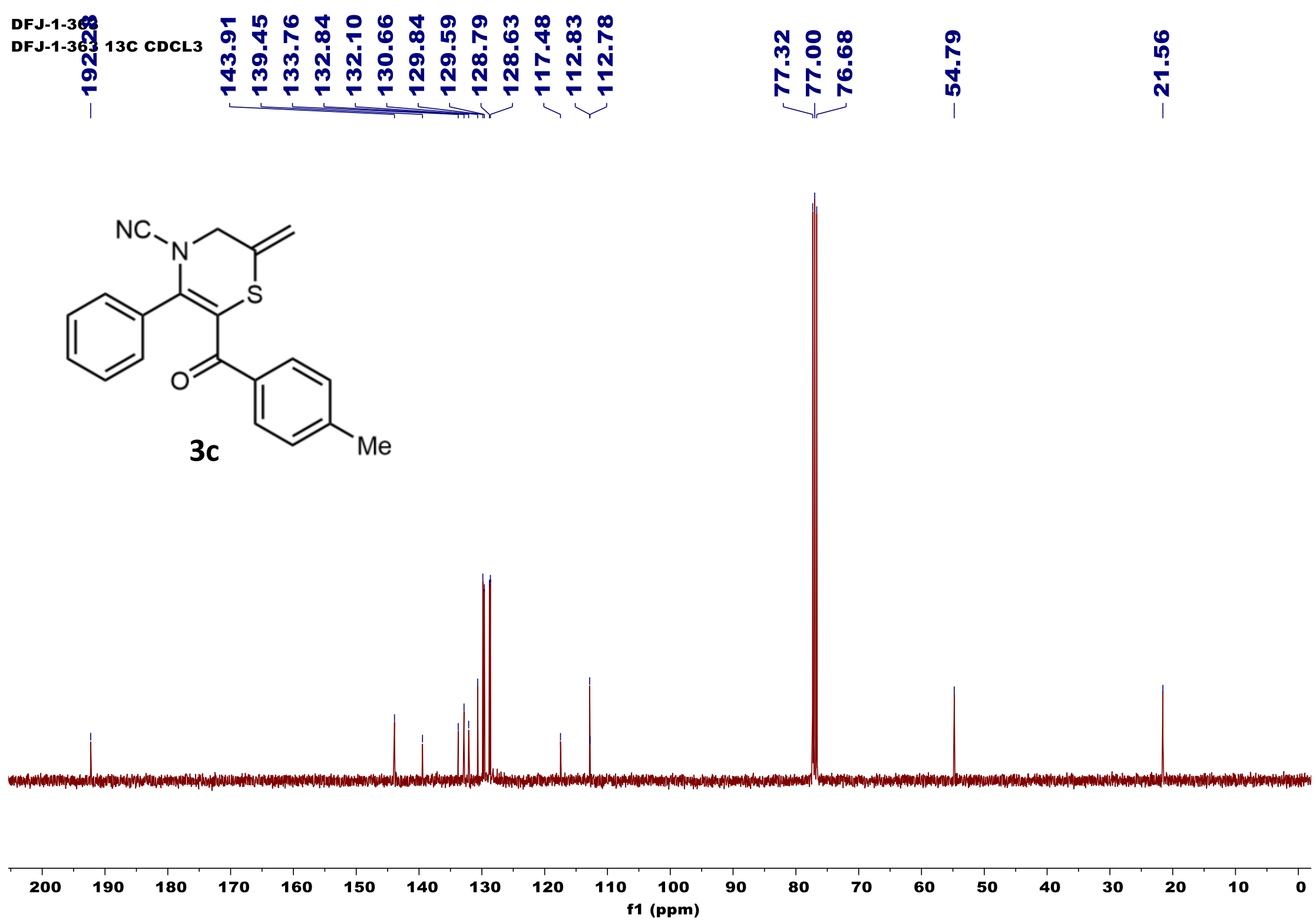

^{1}H NMR spectrum of compound 3d (400 MHz, CDCl$_3$)

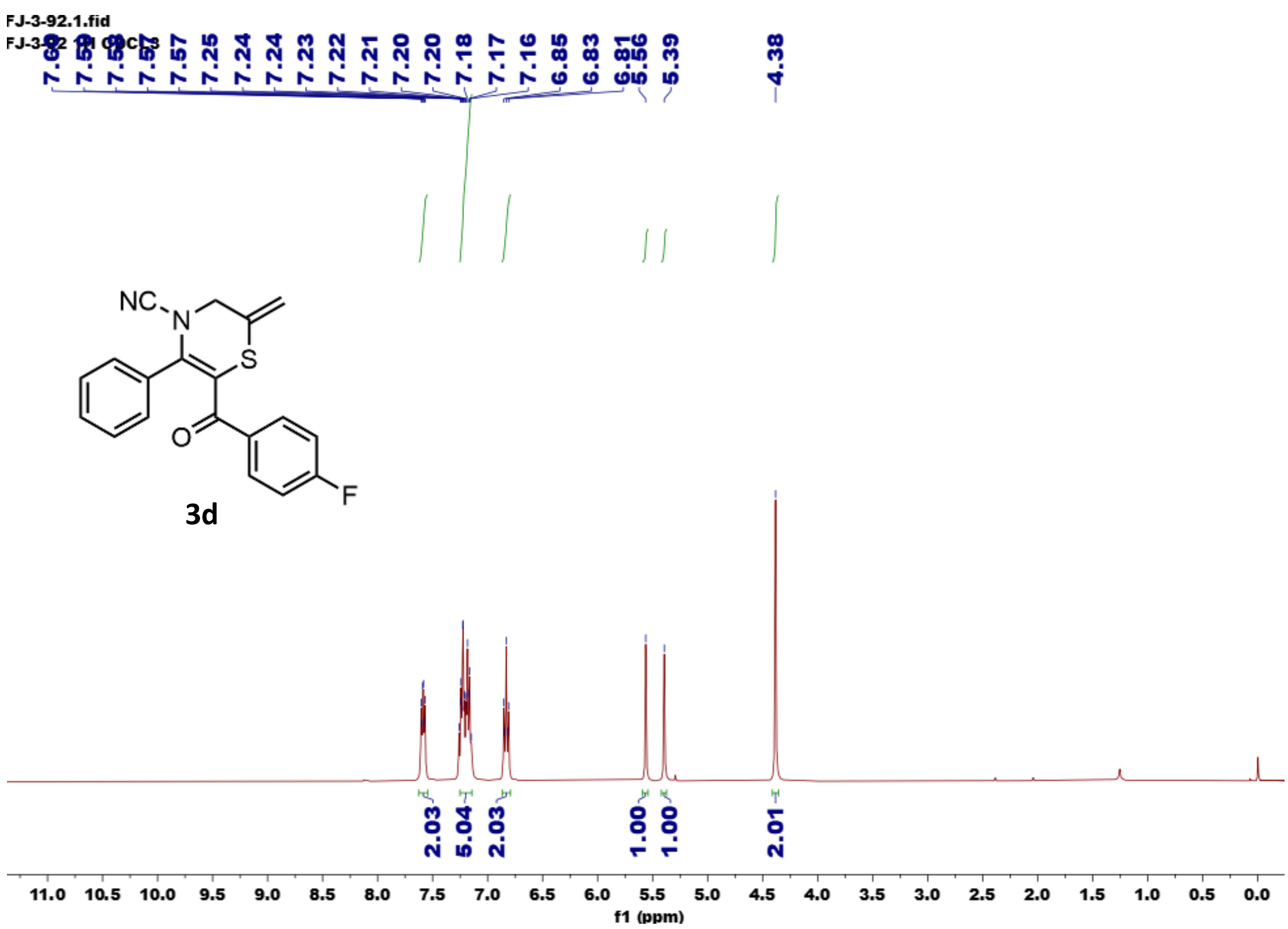

^{13}C NMR spectrum of compound 3d (101 MHz, $CDCl_3$)

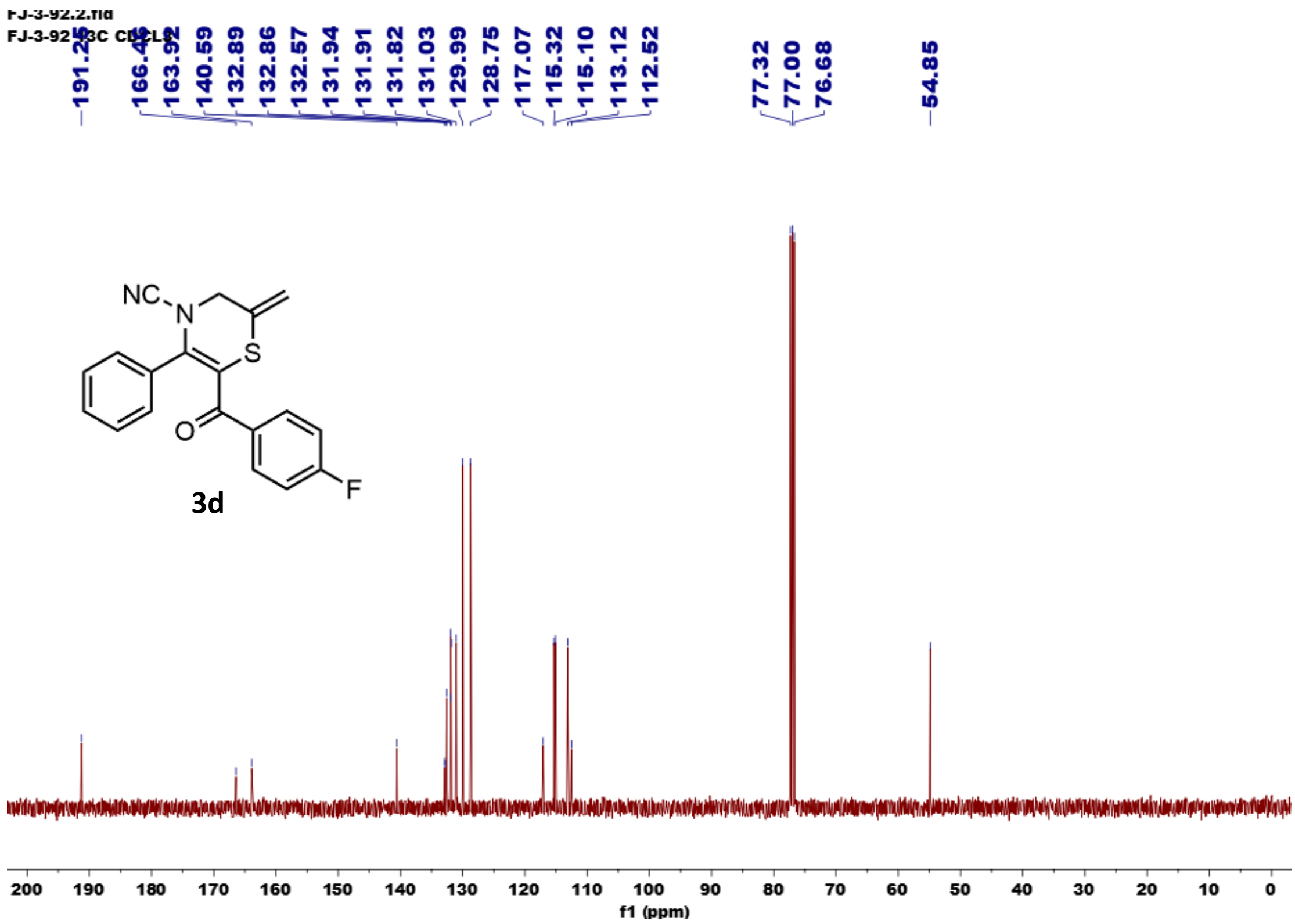

^{19}F NMR spectrum of compound 3d (376 MHz, $CDCl_3$)

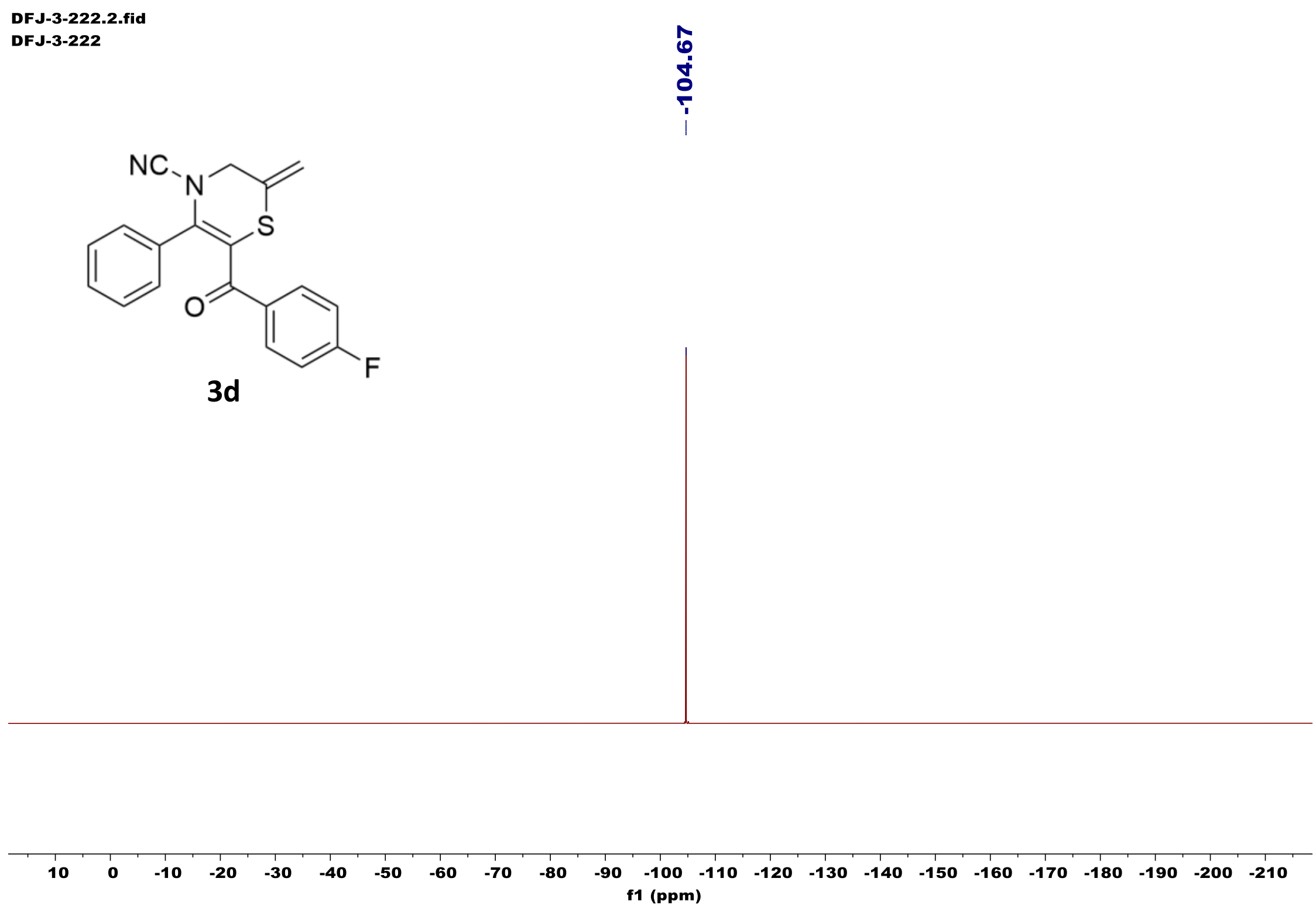

¹H NMR spectrum of compound 3e (400 MHz, CDCl₃)

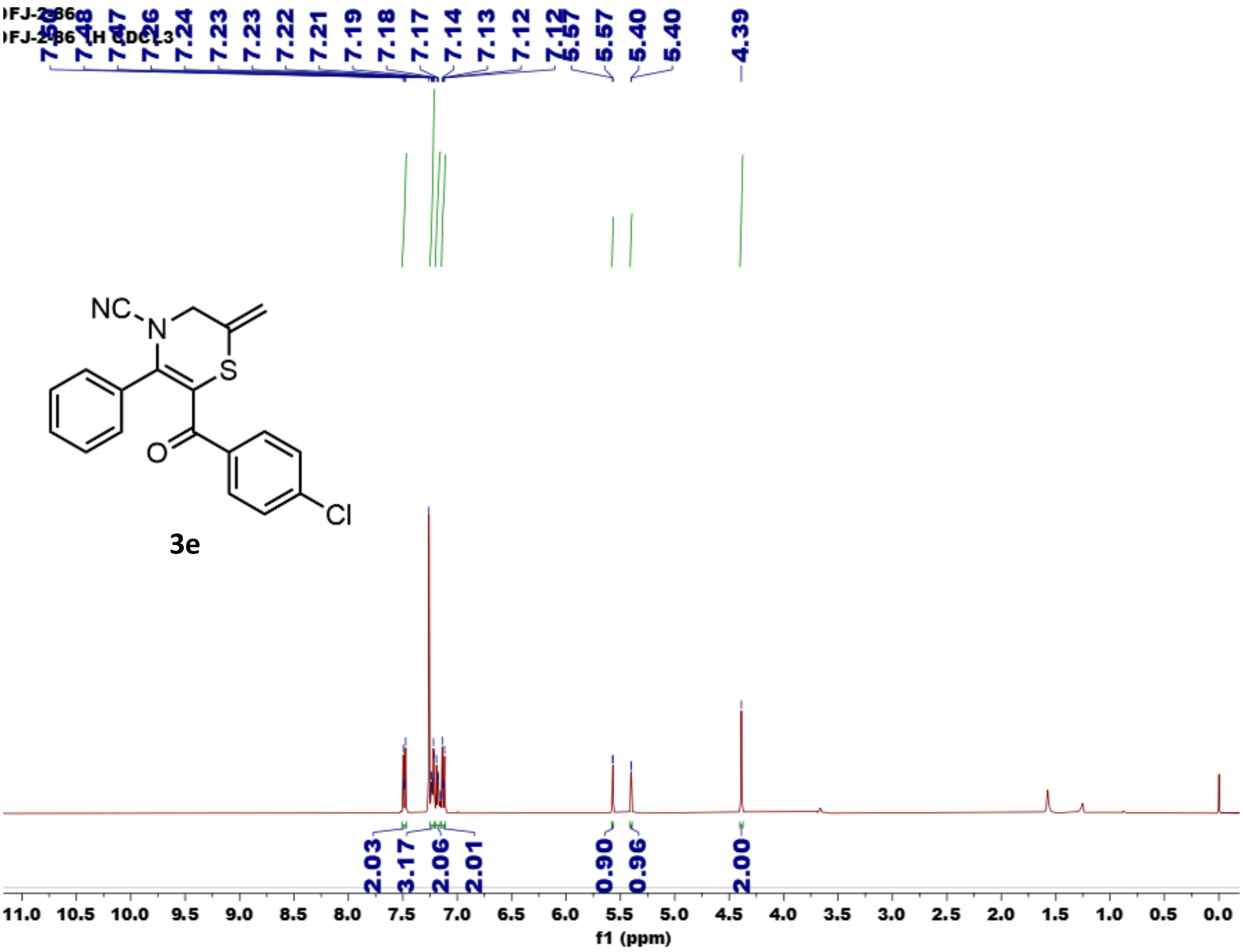

^{13}C NMR spectrum of compound 3e (101 MHz, CDCl₃)

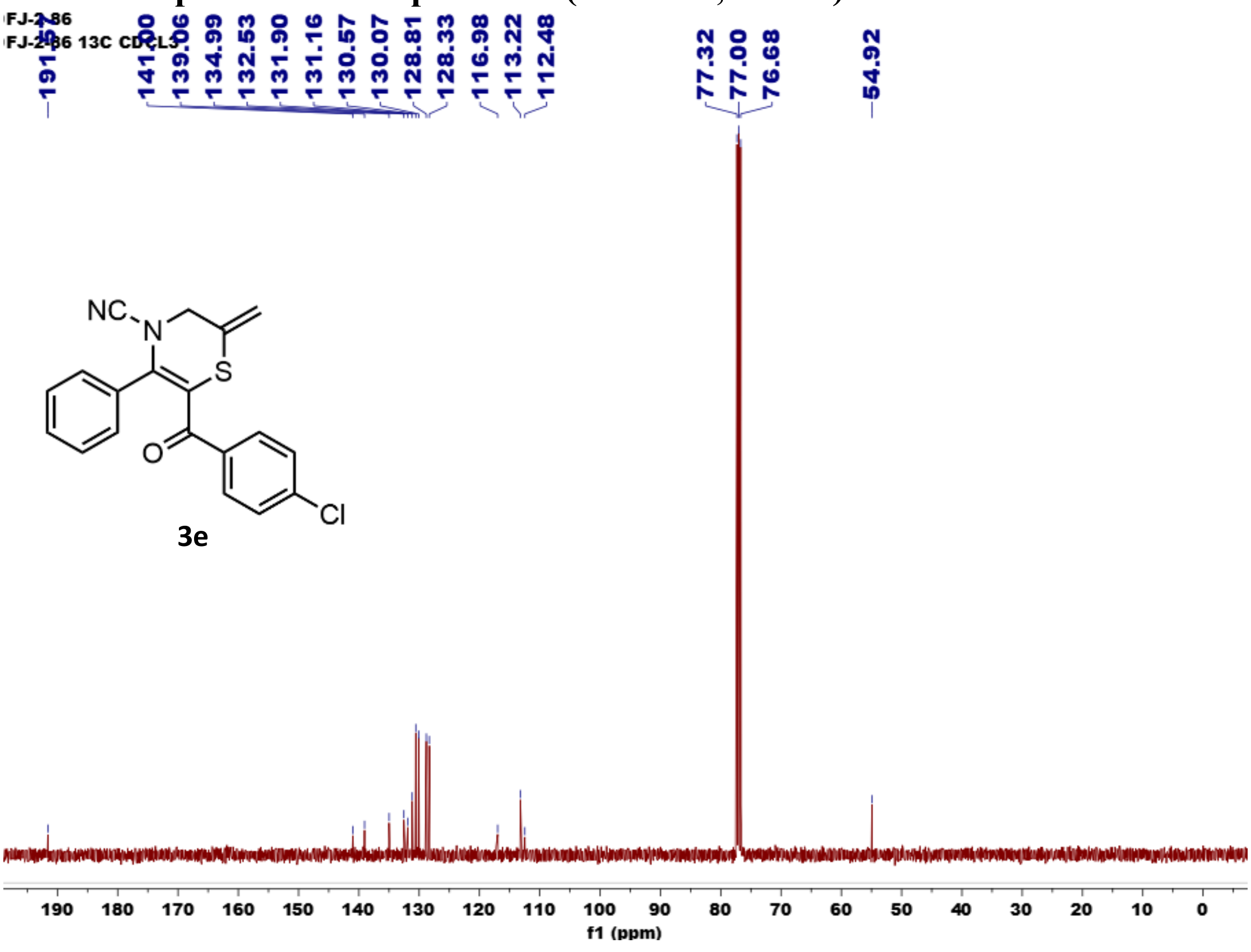

^{1}H NMR spectrum of compound 3f (400 MHz, $CDCl_3$)

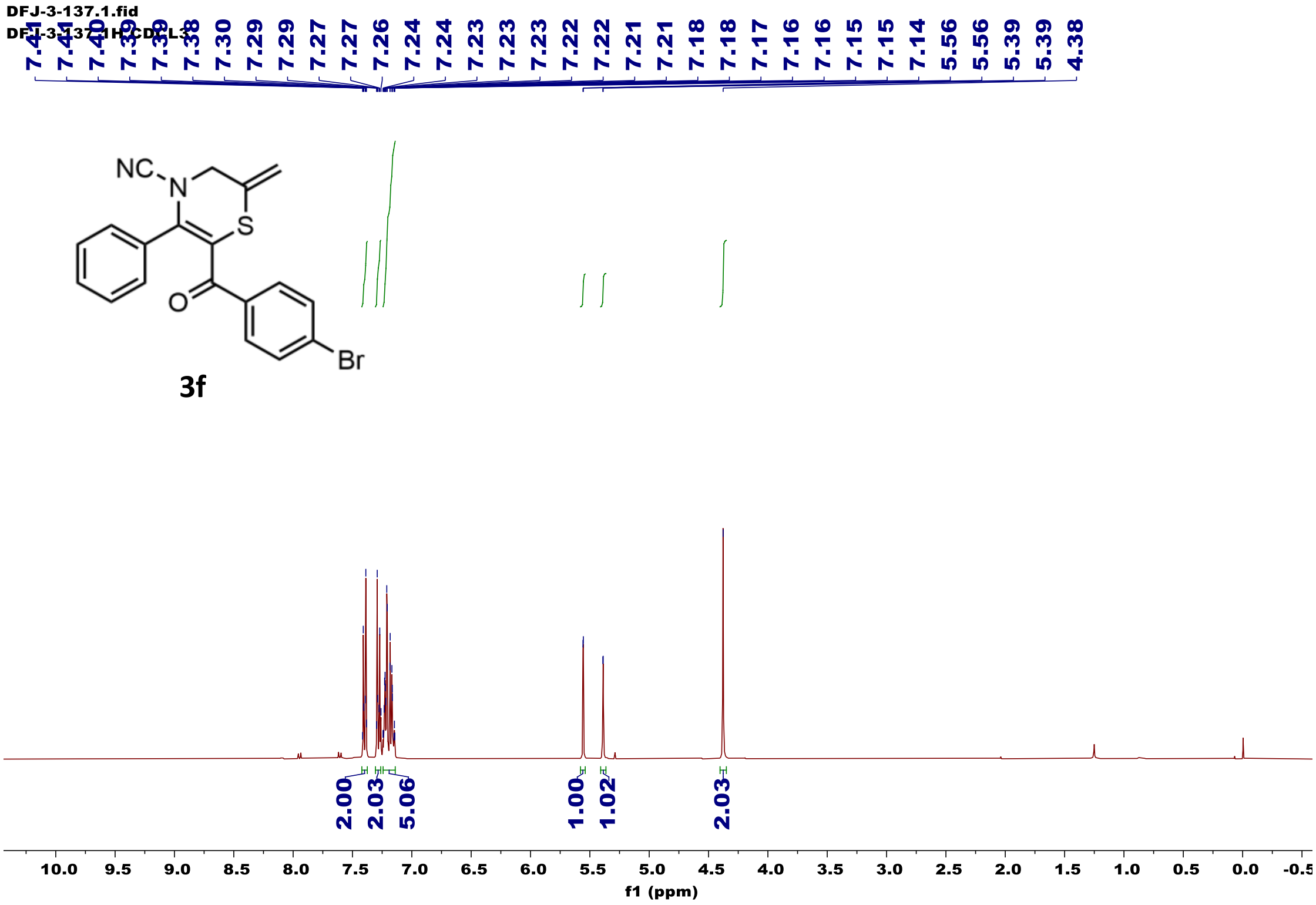

^{13}C NMR spectrum of compound 3f (101 MHz, $CDCl_3$)

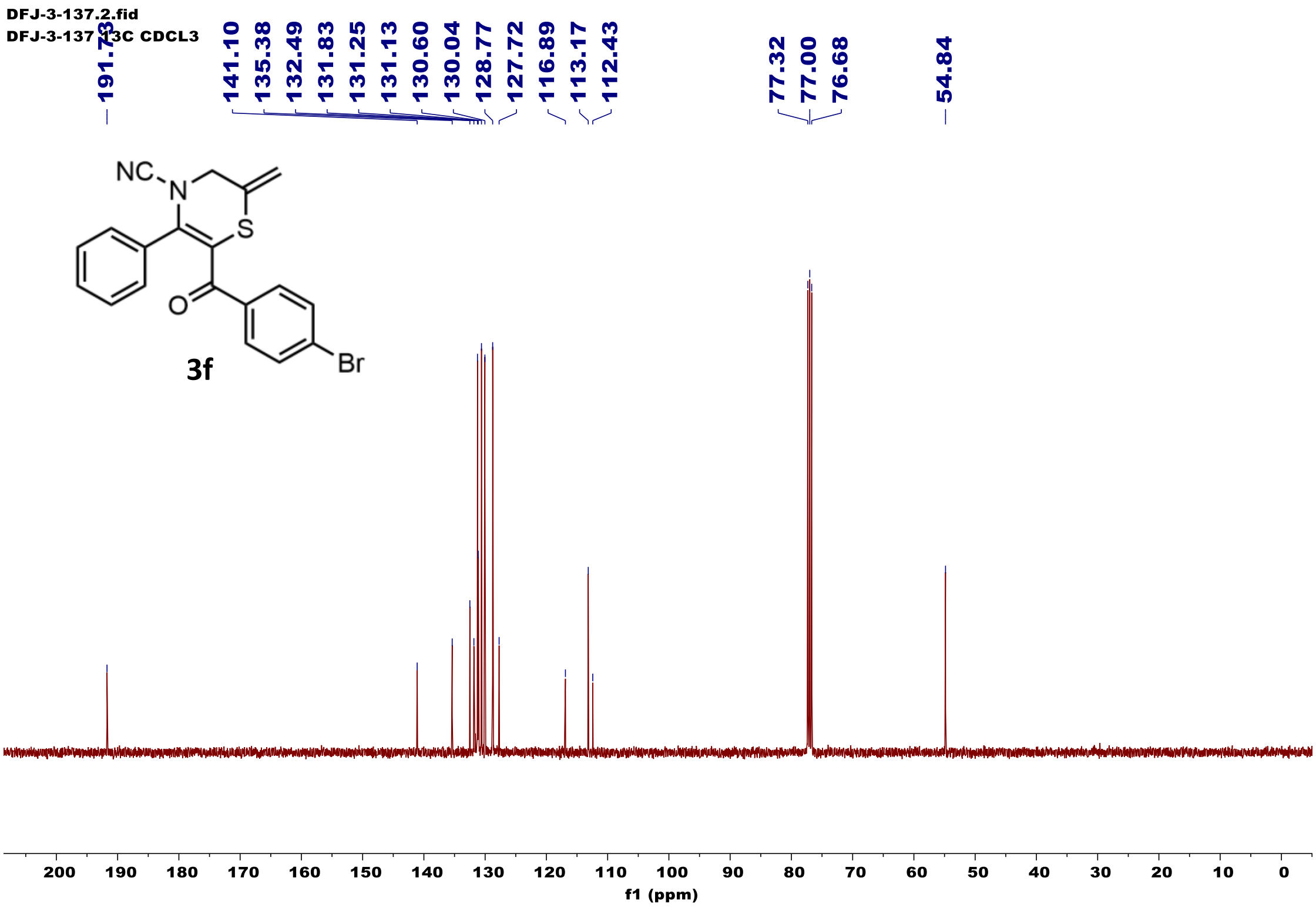

^{1}H NMR spectrum of compound 3g (400 MHz, $CDCl_3$)

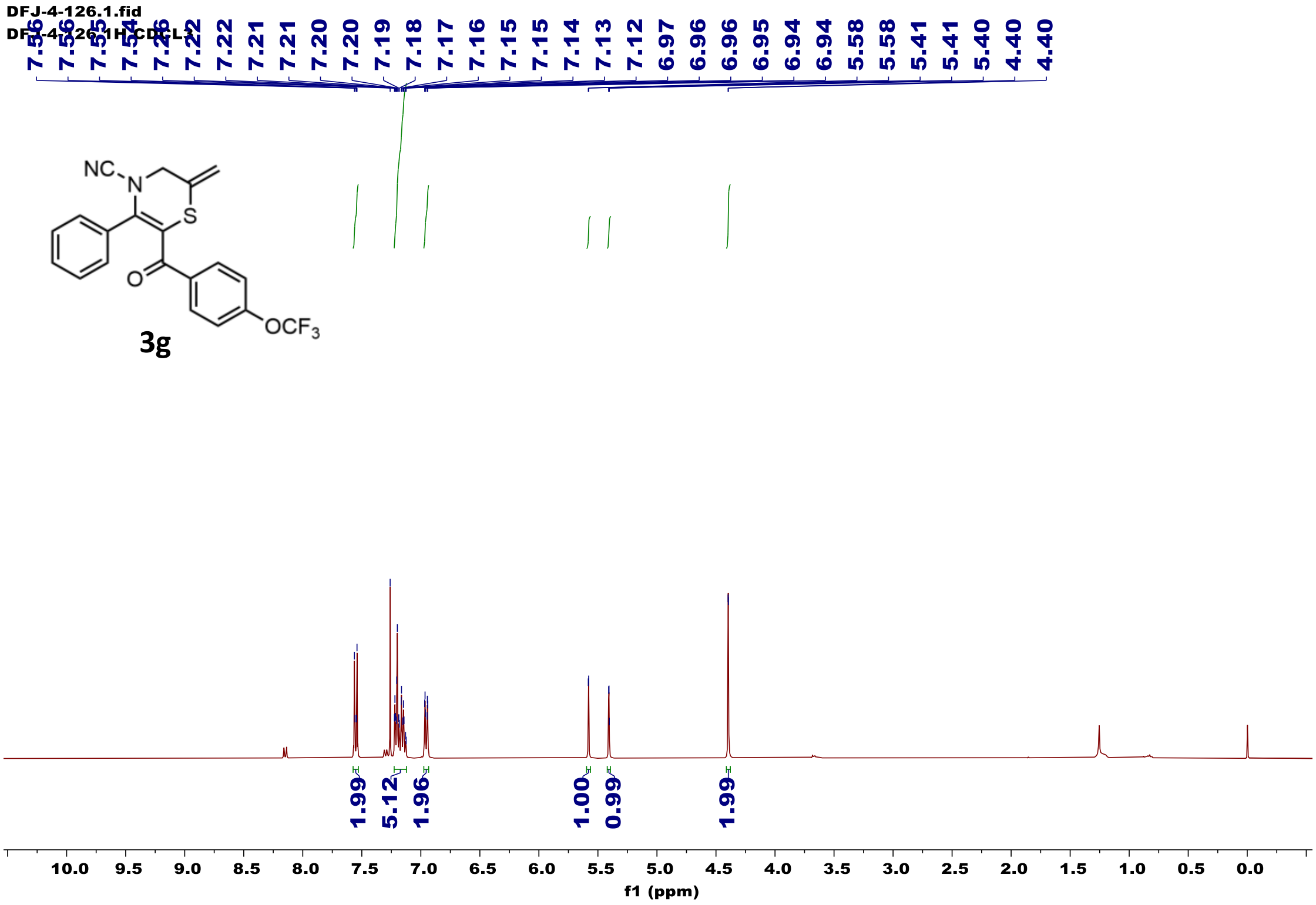

^{13}C NMR spectrum of compound 3g (101 MHz, $CDCl_3$)

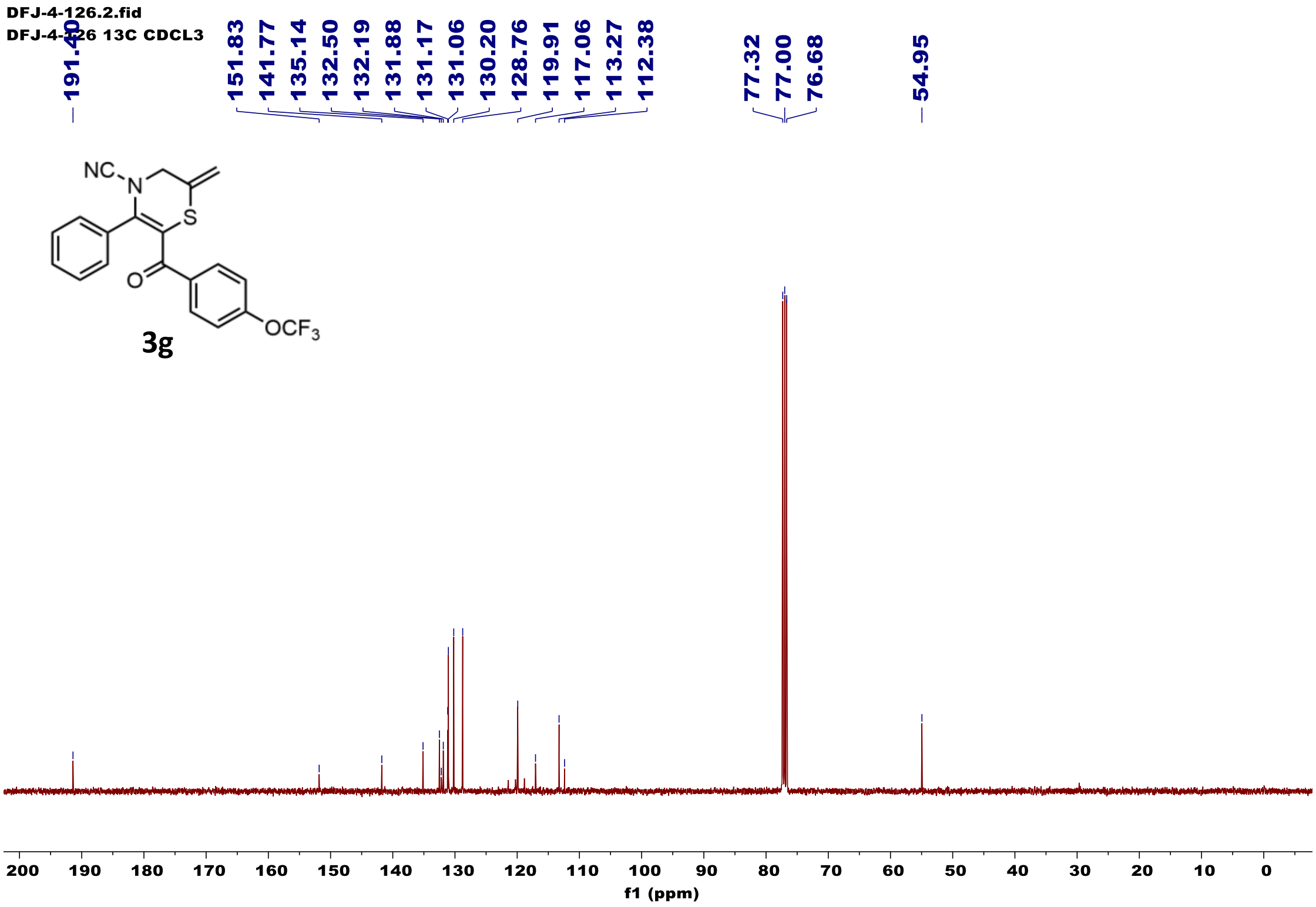

^{19}F NMR spectrum of compound 3g (376 MHz, $CDCl_3$)

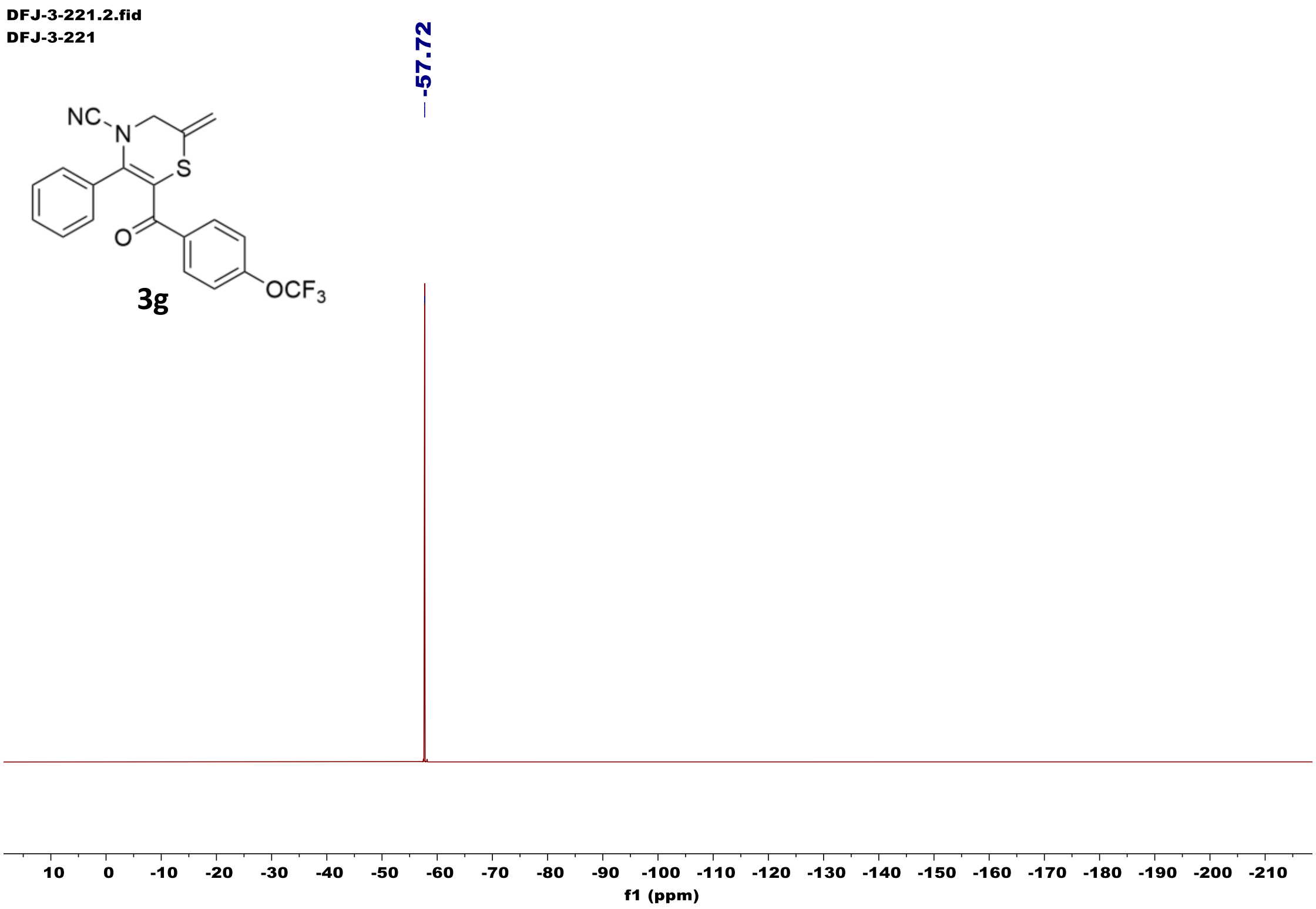

^{1}H NMR spectrum of compound 3h (400 MHz, $CDCl_3$)

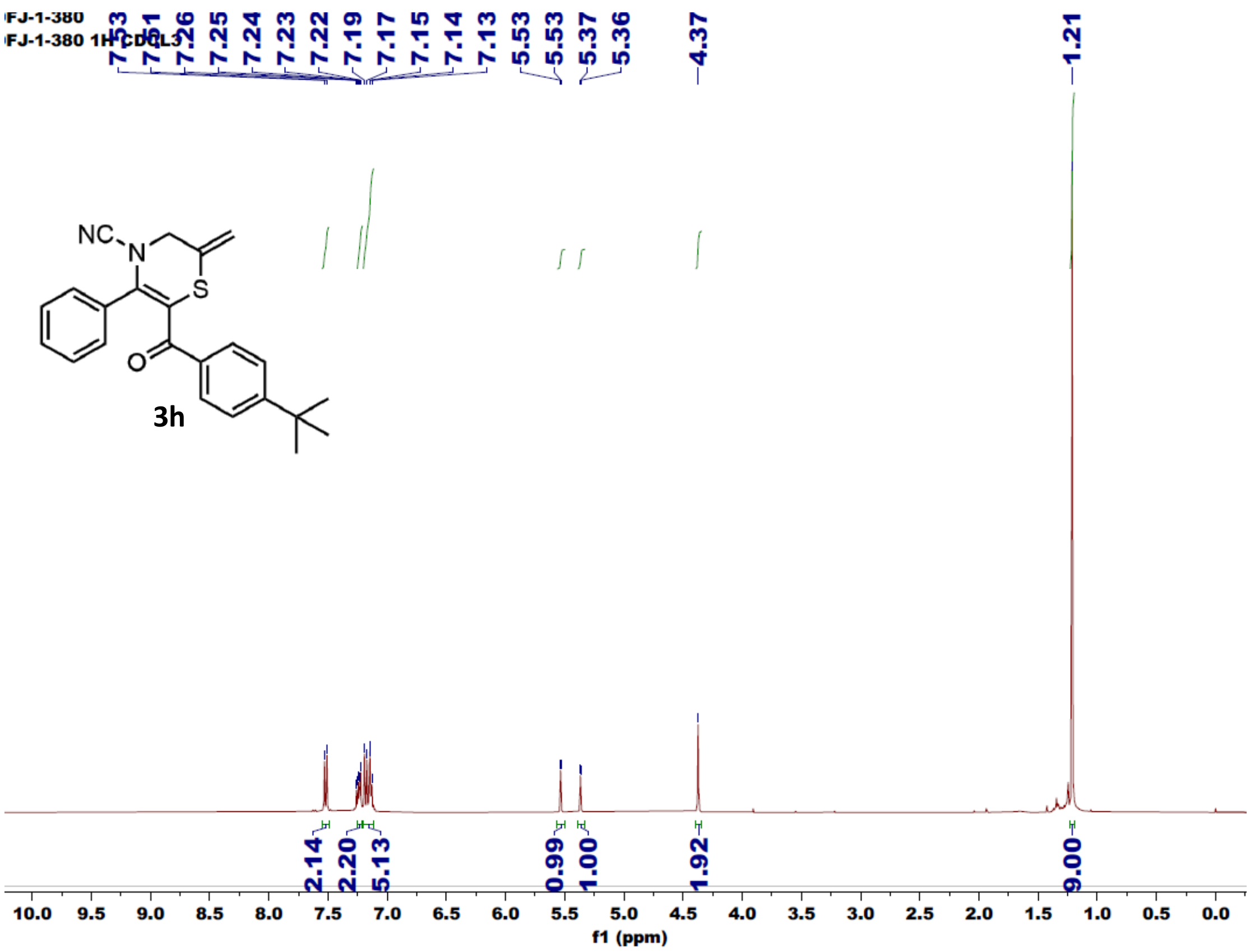

^{13}C NMR spectrum of compound 3h (101 MHz, $CDCl_3$)

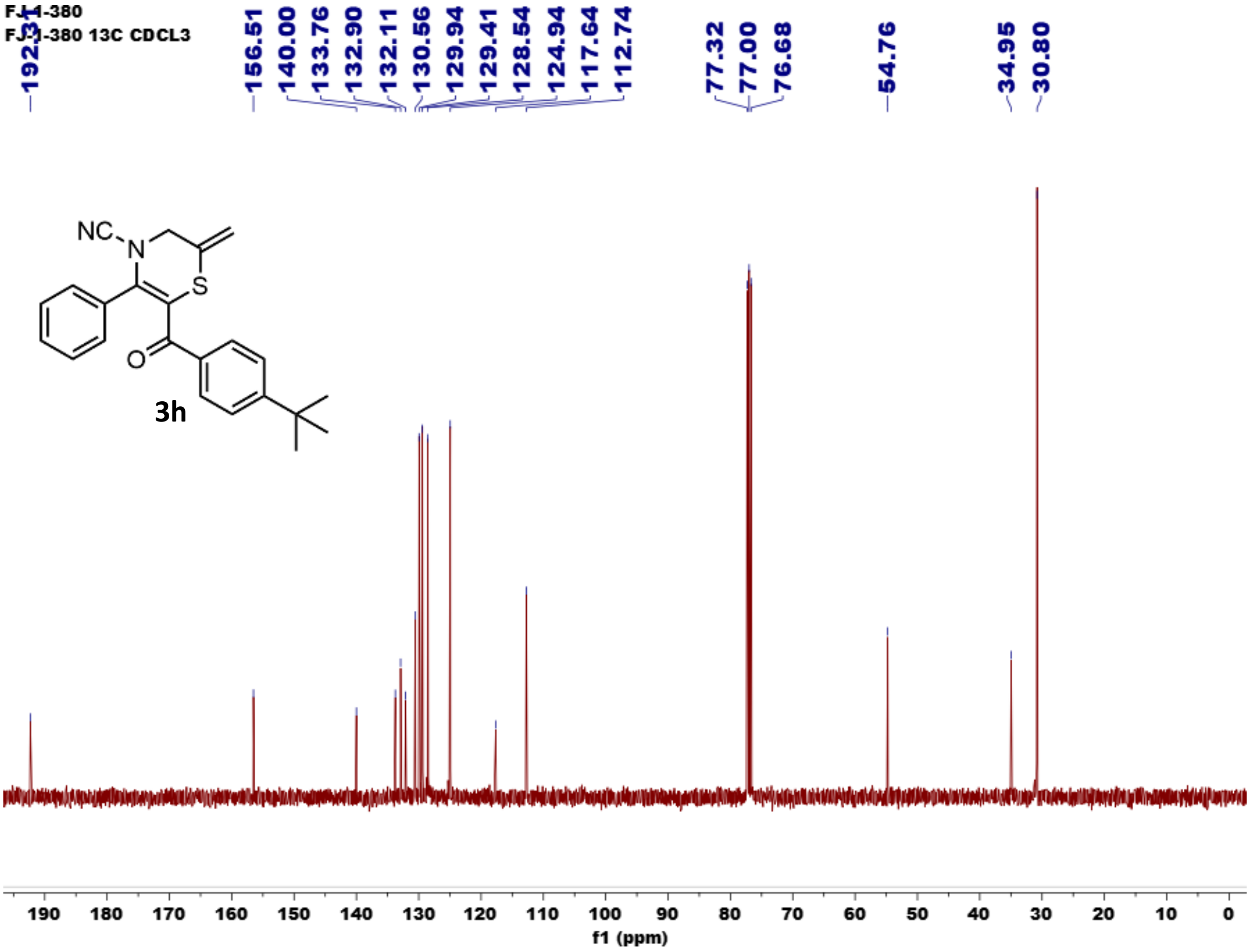

^{1}H NMR spectrum of compound 3i (400 MHz, $CDCl_3$)

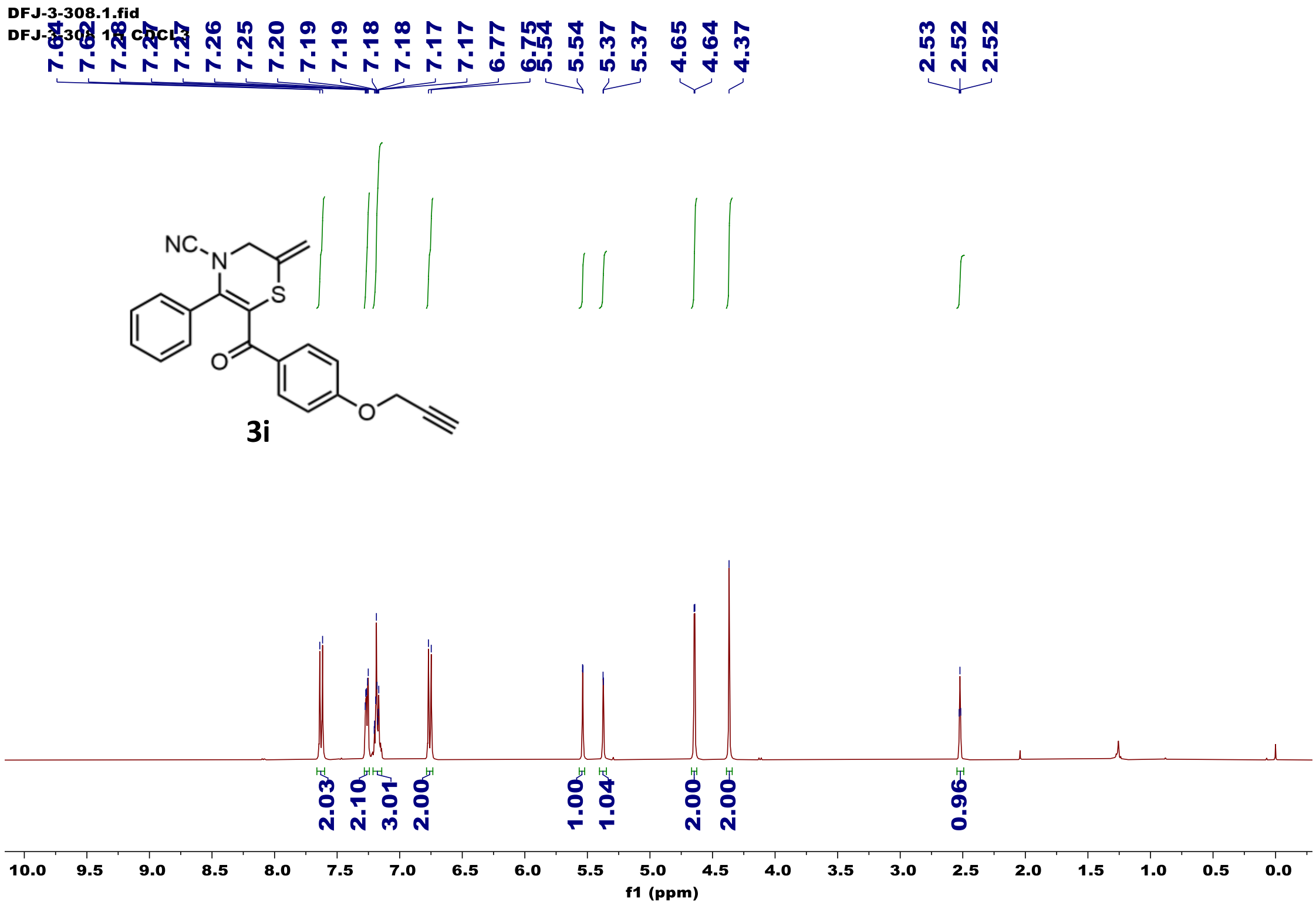

^{13}C NMR spectrum of compound 3i (101 MHz, $CDCl_3$)

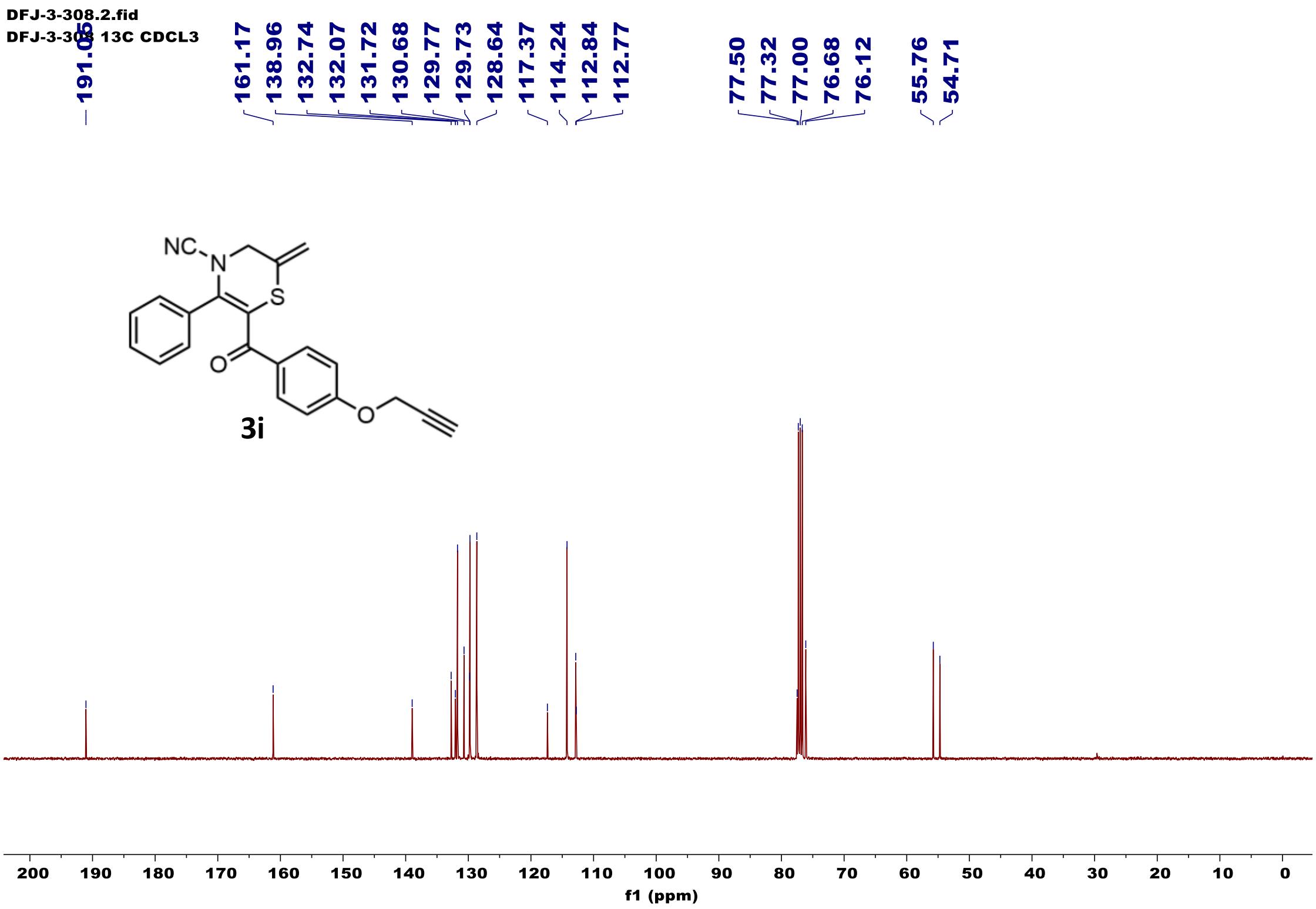

^{1}H NMR spectrum of compound 3j (400 MHz, $CDCl_3$)

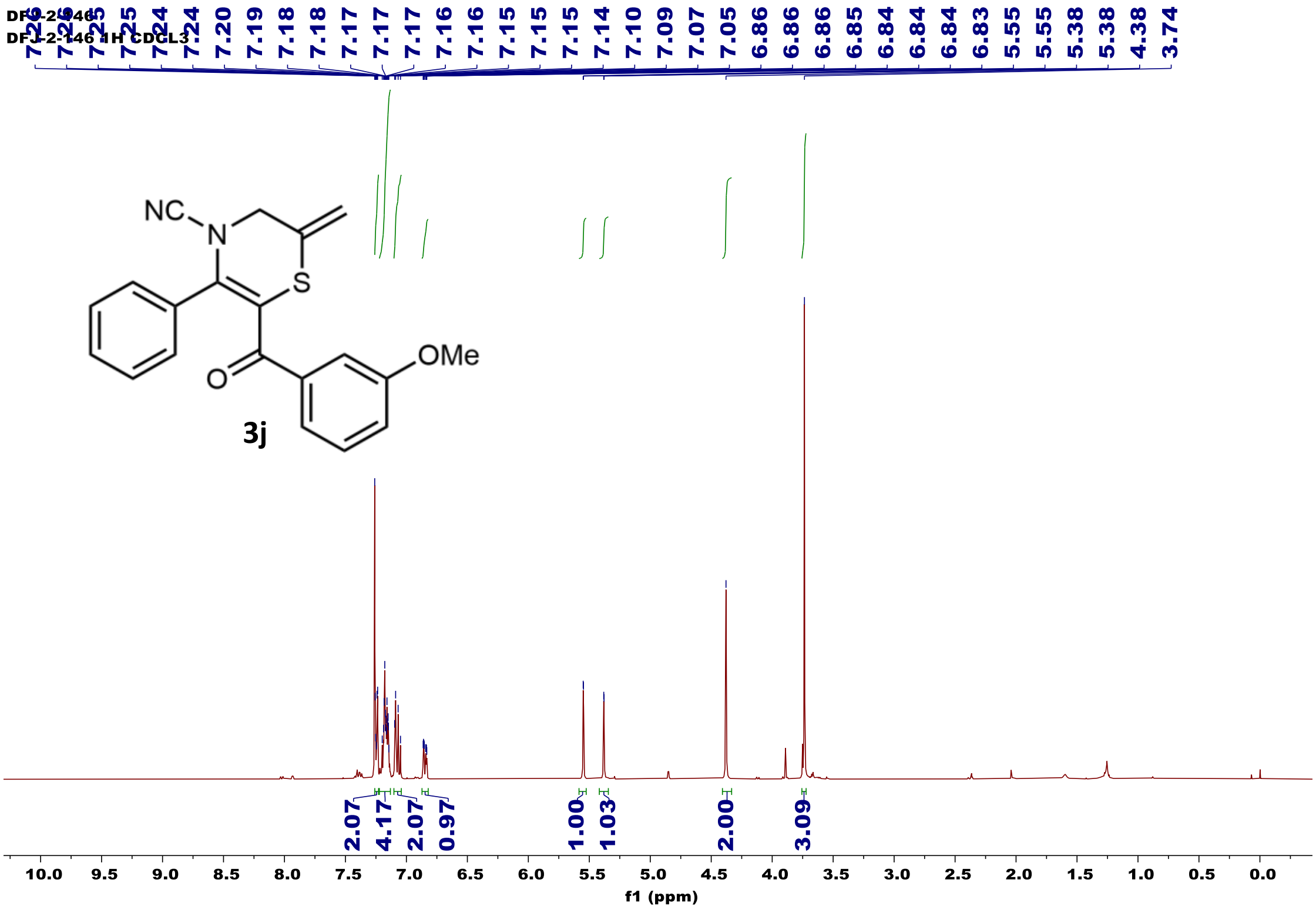

^{13}C NMR spectrum of compound 3j (101 MHz, $CDCl_3$)

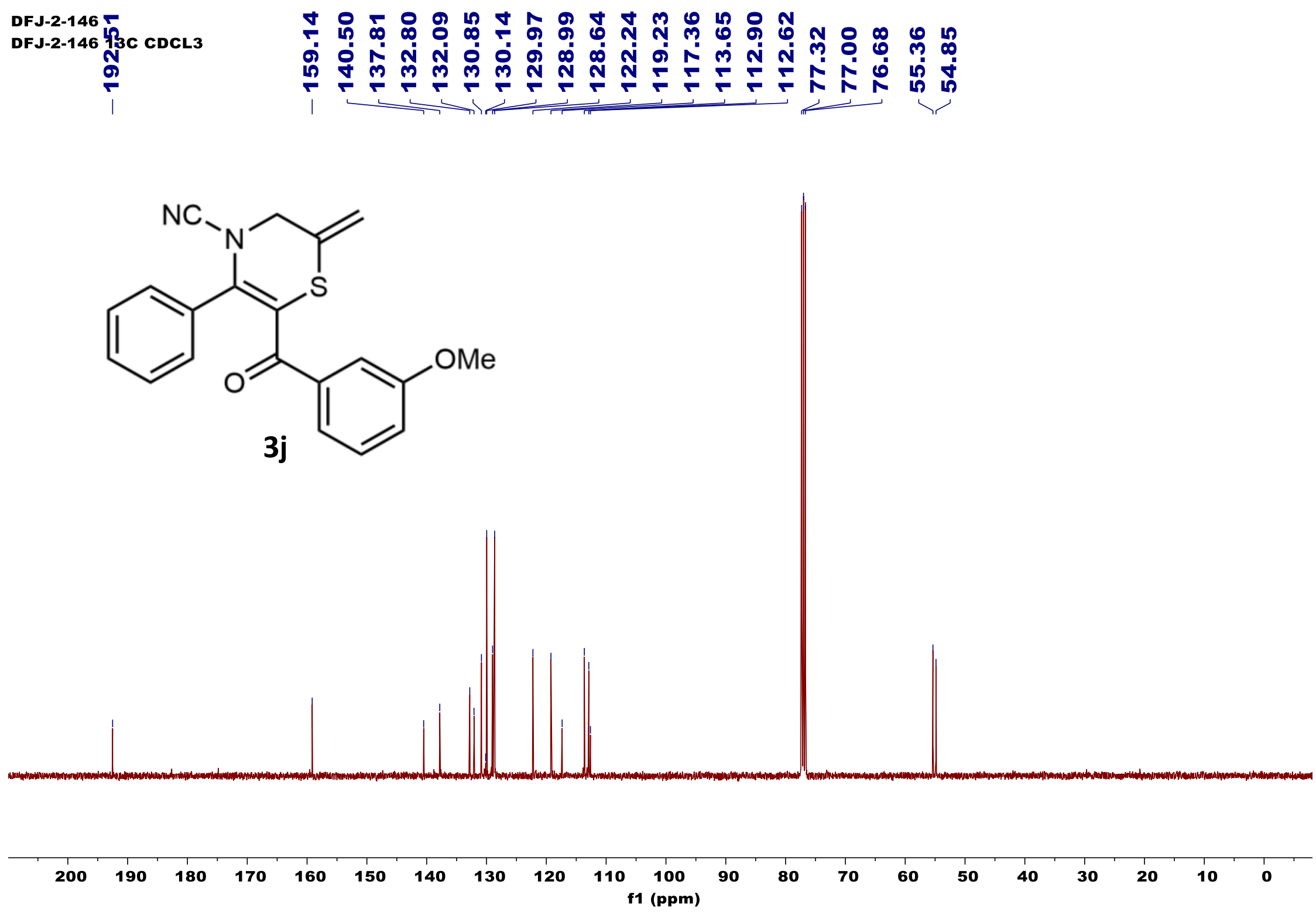

^{1}H NMR spectrum of compound 3k (400 MHz, $CDCl_3$)

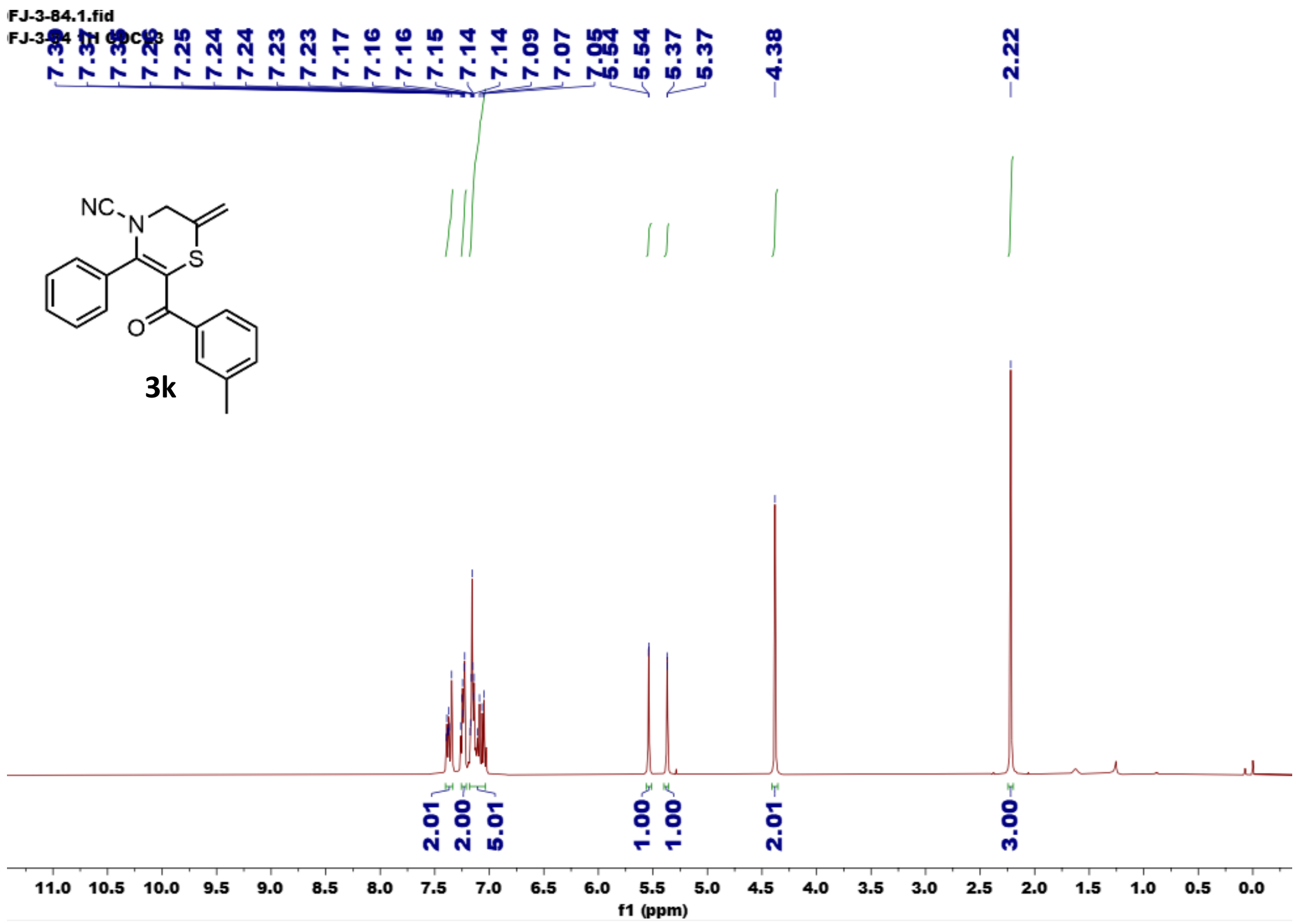

^{13}C NMR spectrum of compound 3k (101 MHz, $CDCl_3$)

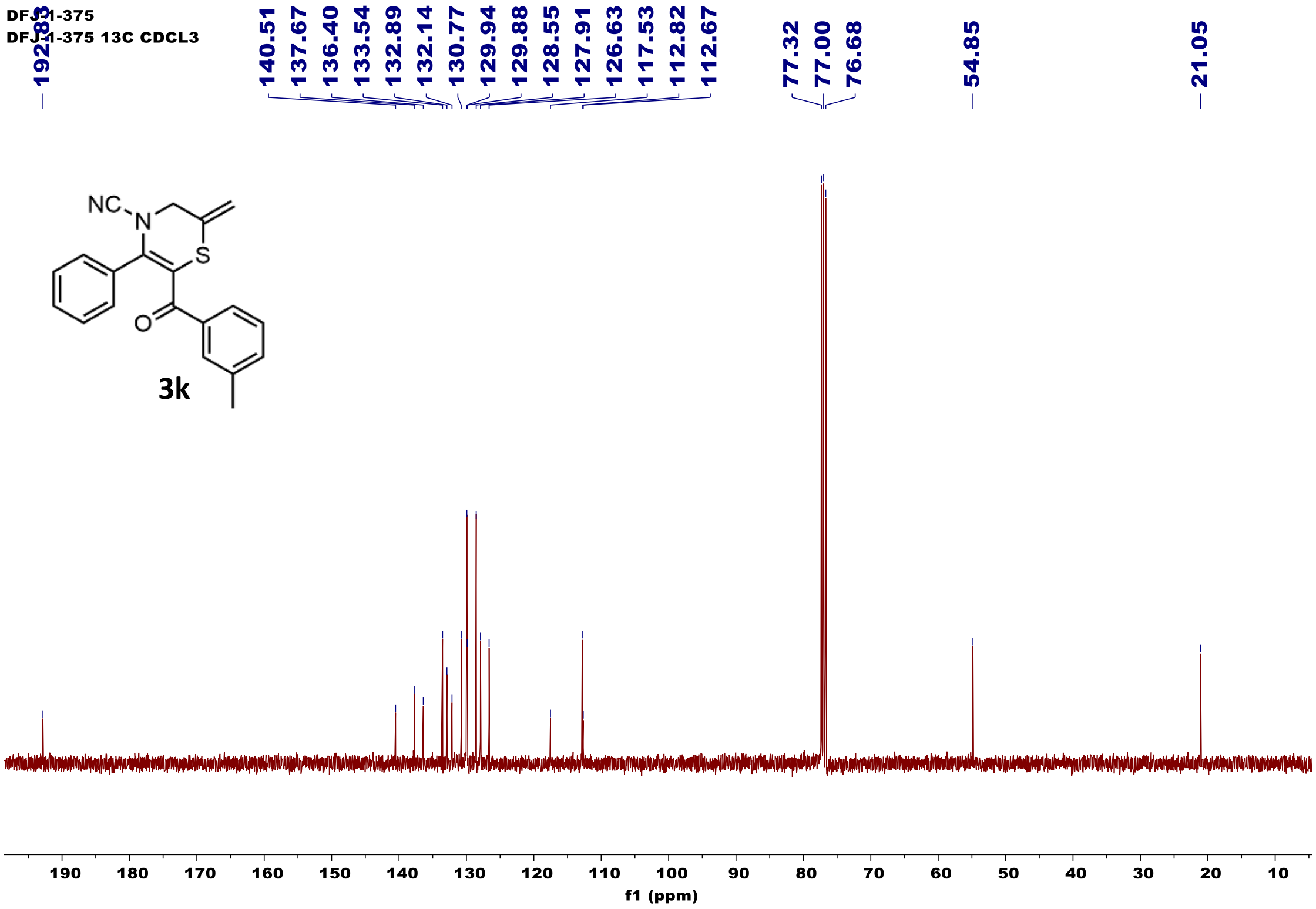

^{1}H NMR spectrum of compound 3l (400 MHz, $CDCl_3$)

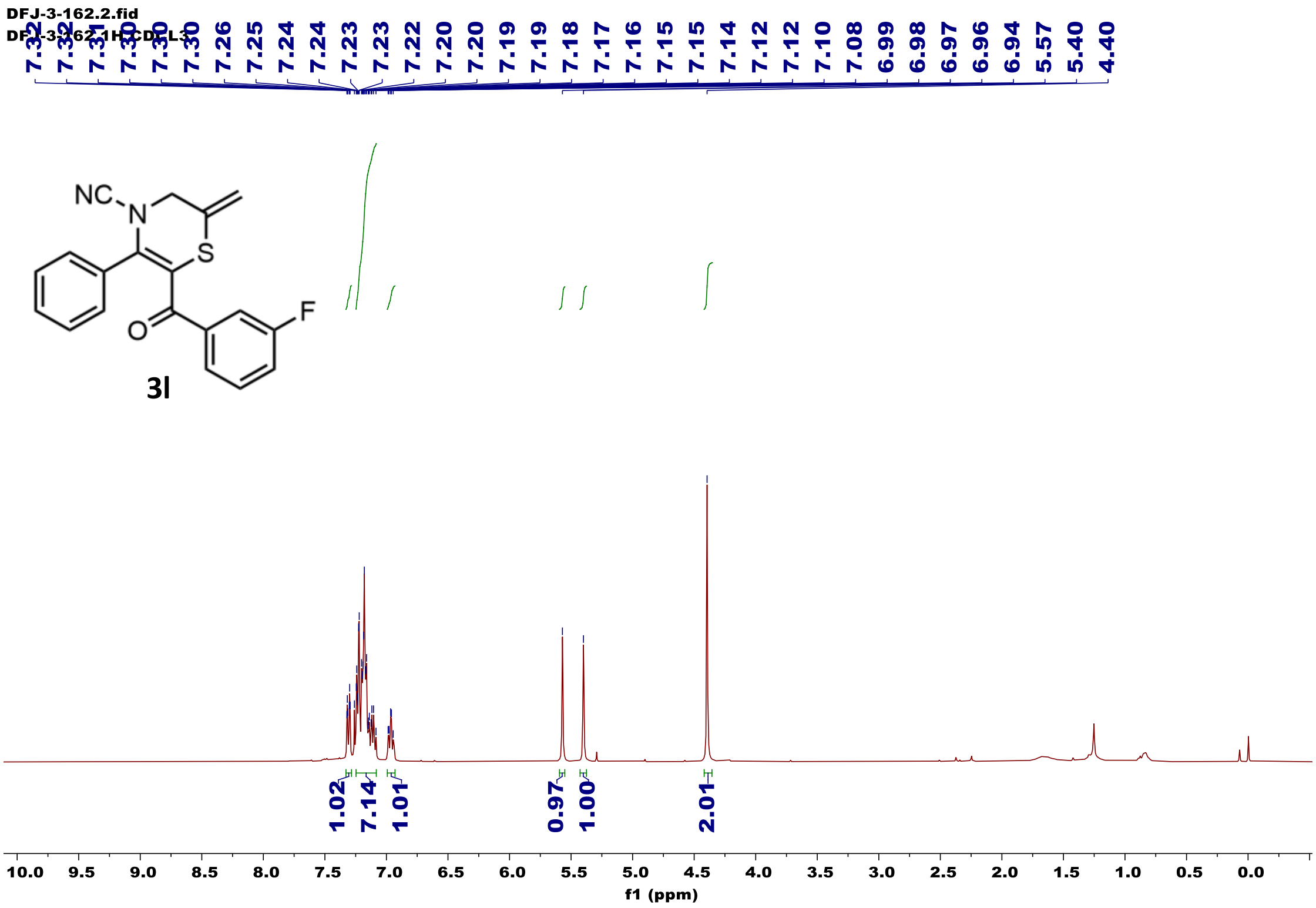

^{13}C NMR spectrum of compound 3l (101 MHz, $CDCl_3$)

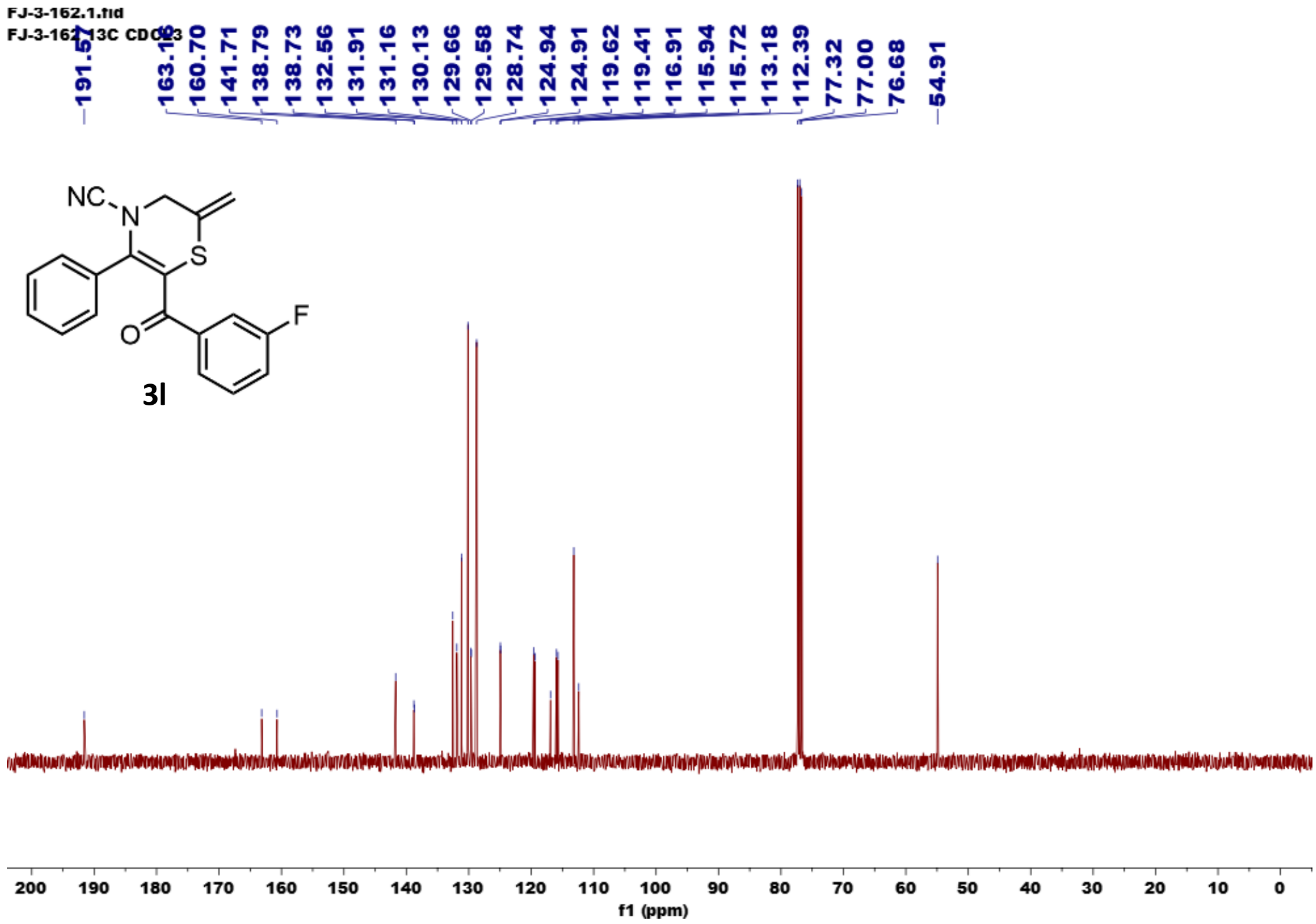

^{19}F NMR spectrum of compound 3l (376 MHz, $CDCl_3$)

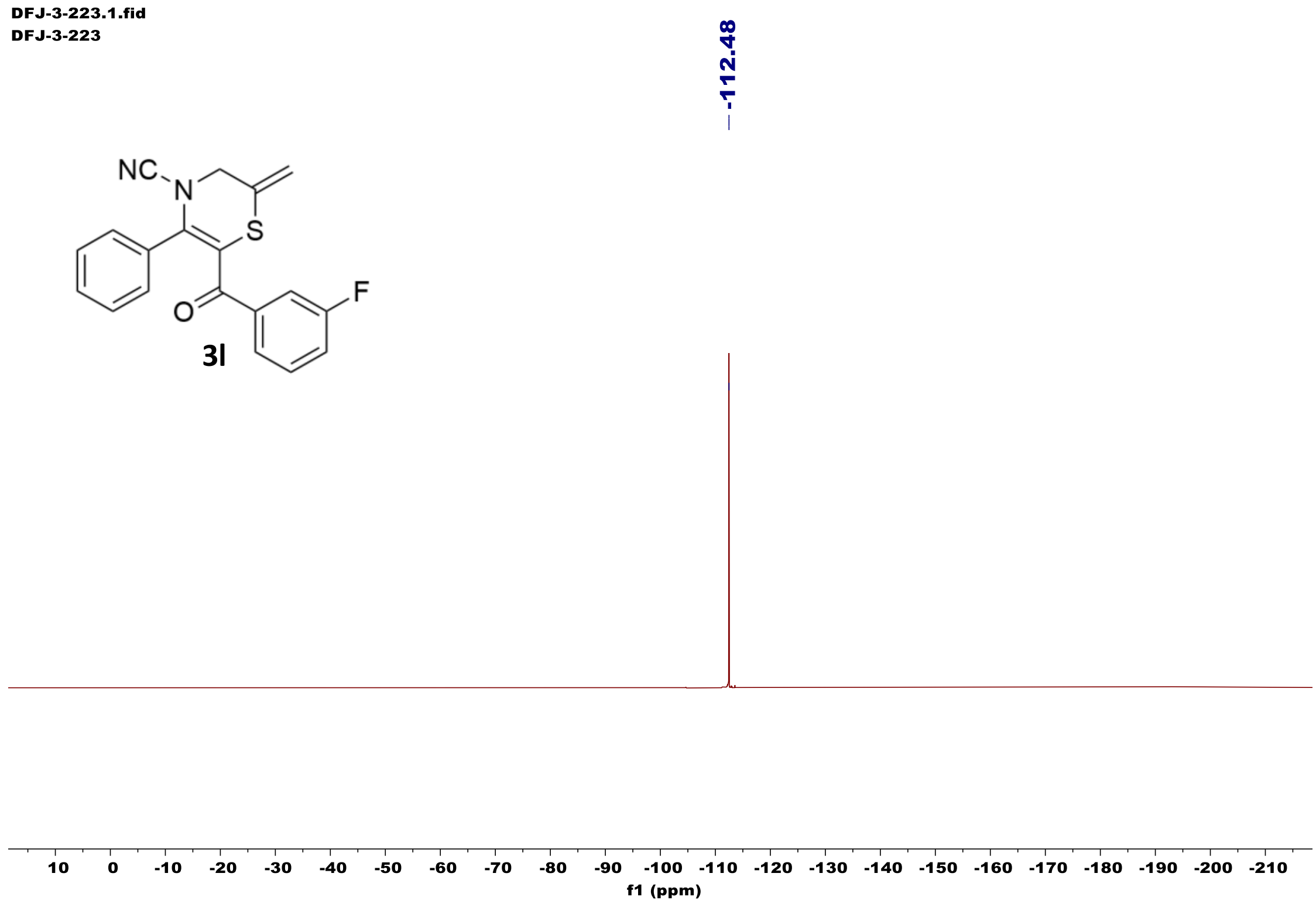

^{1}H NMR spectrum of compound 3m (400 MHz, $CDCl_3$)

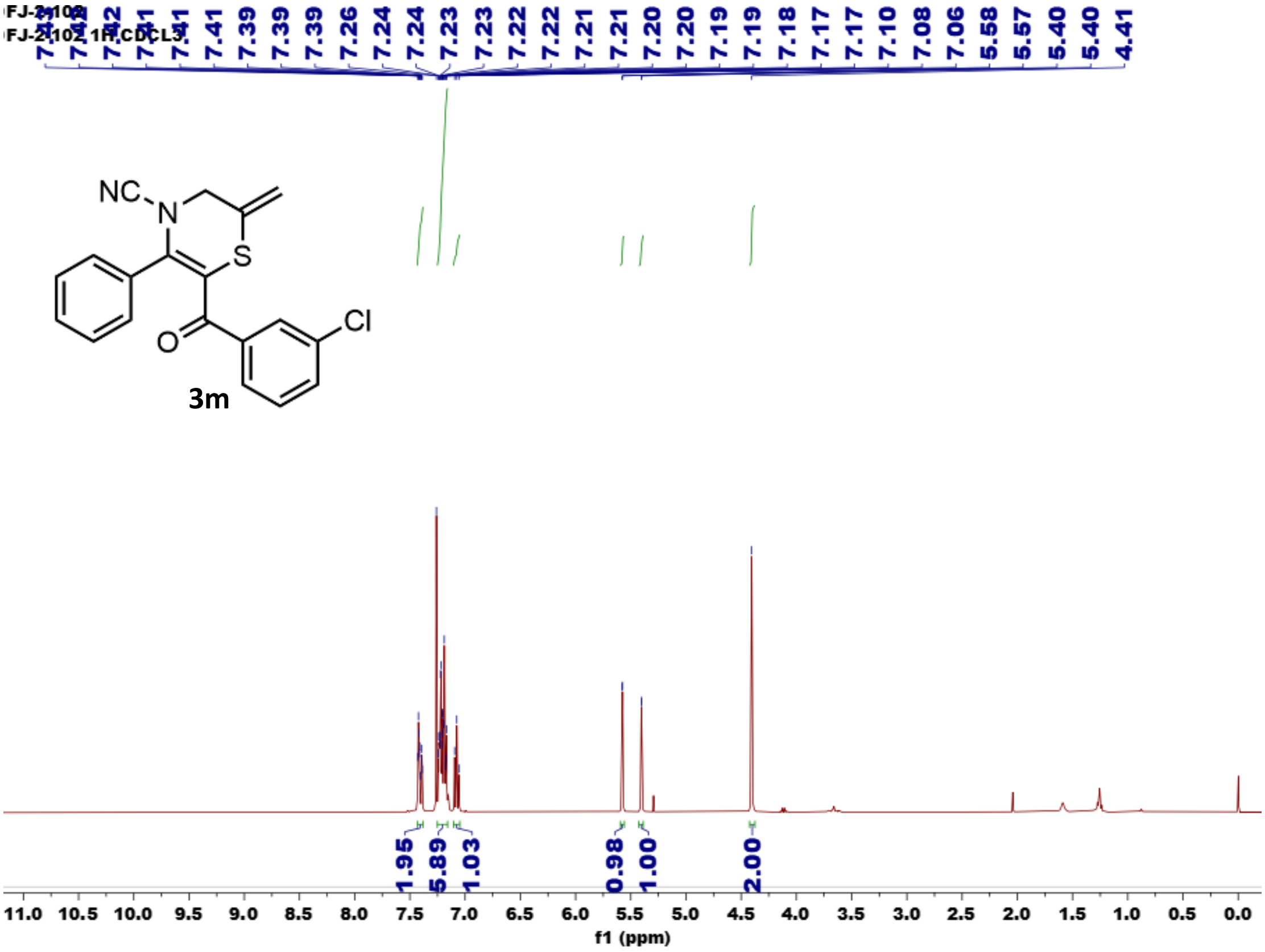

^{13}C NMR spectrum of compound 3m (101 MHz, $CDCl_3$)

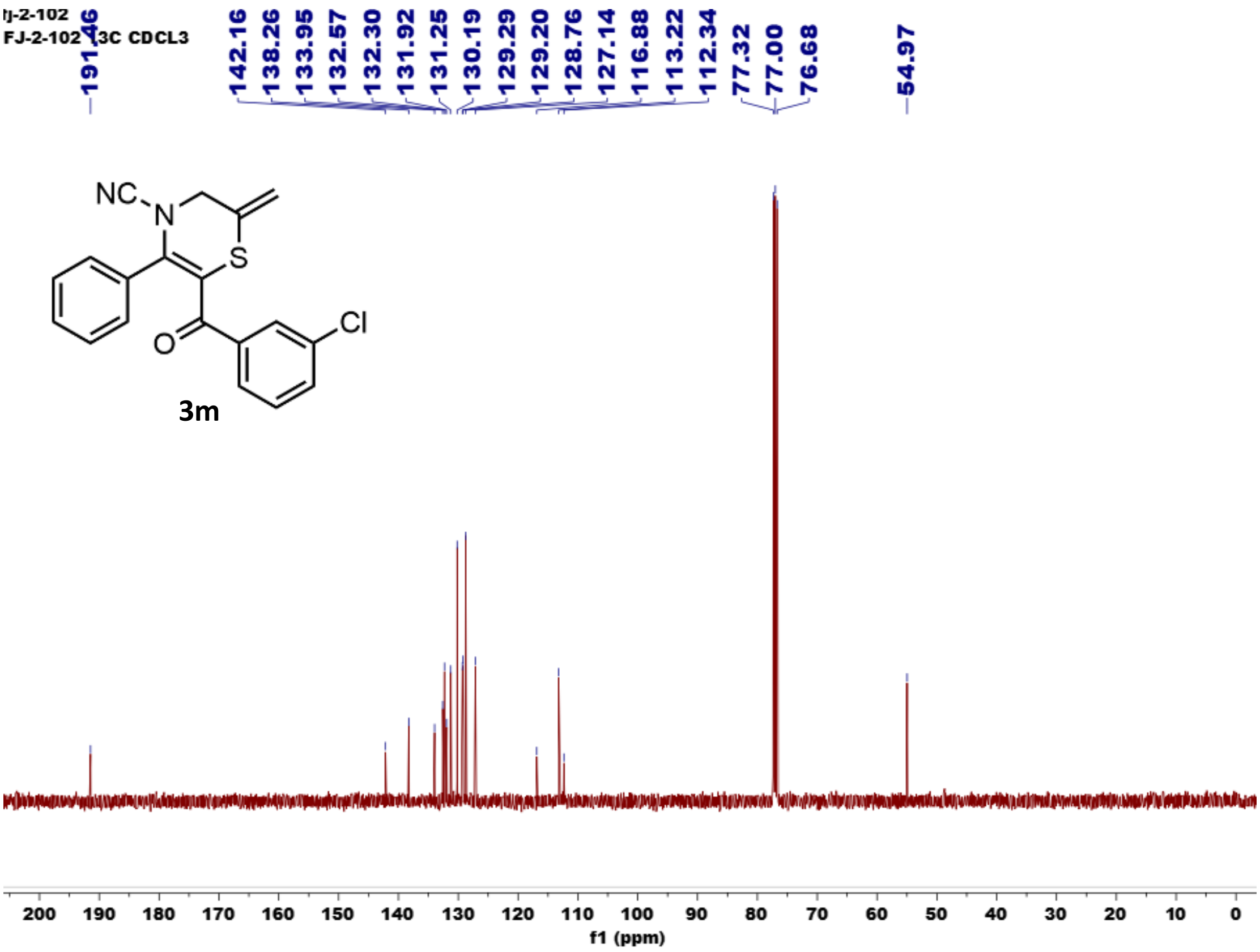

^{1}H NMR spectrum of compound 3n (400 MHz, $CDCl_3$)

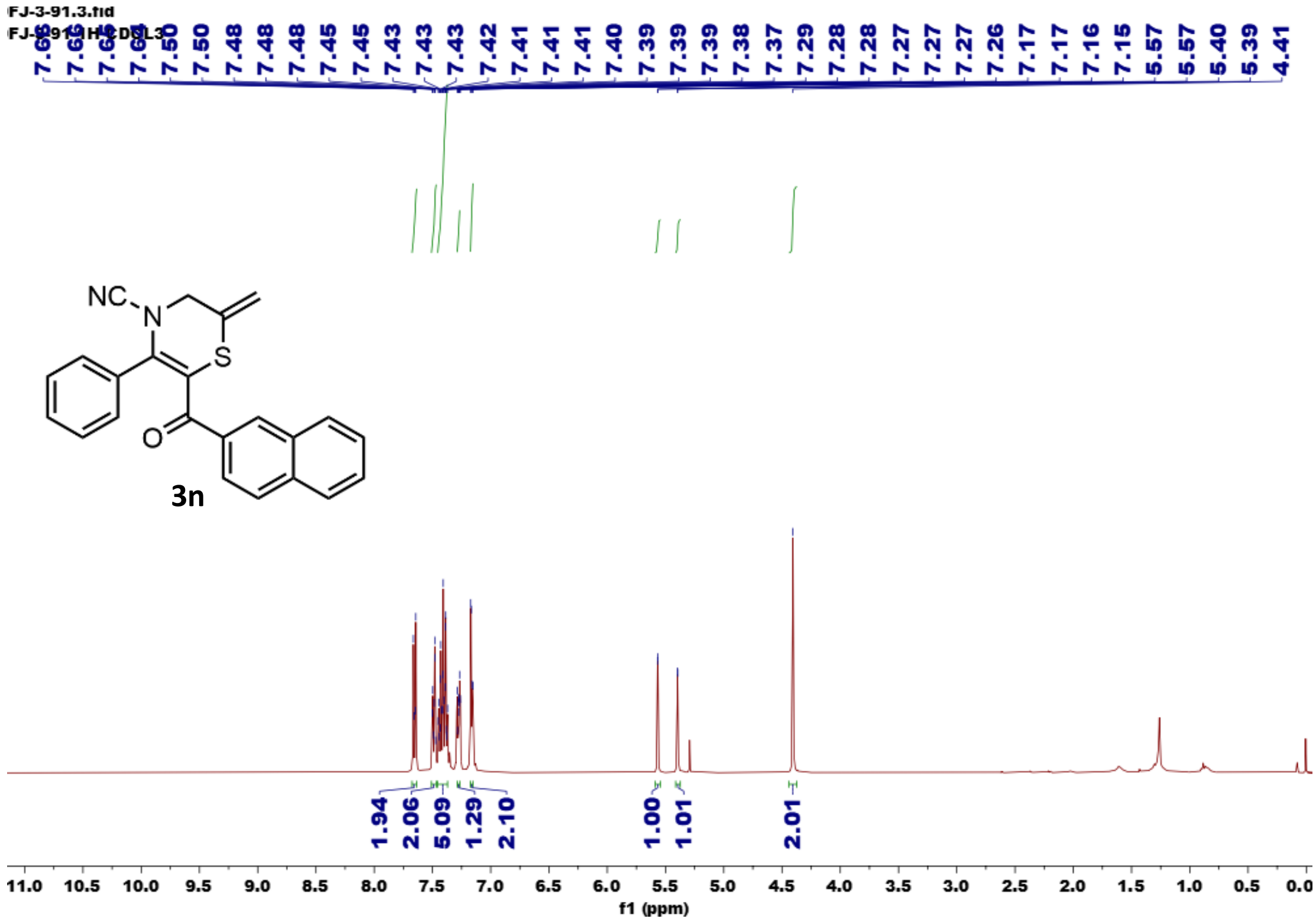

^{13}C NMR spectrum of compound 3n (101 MHz, $CDCl_3$)

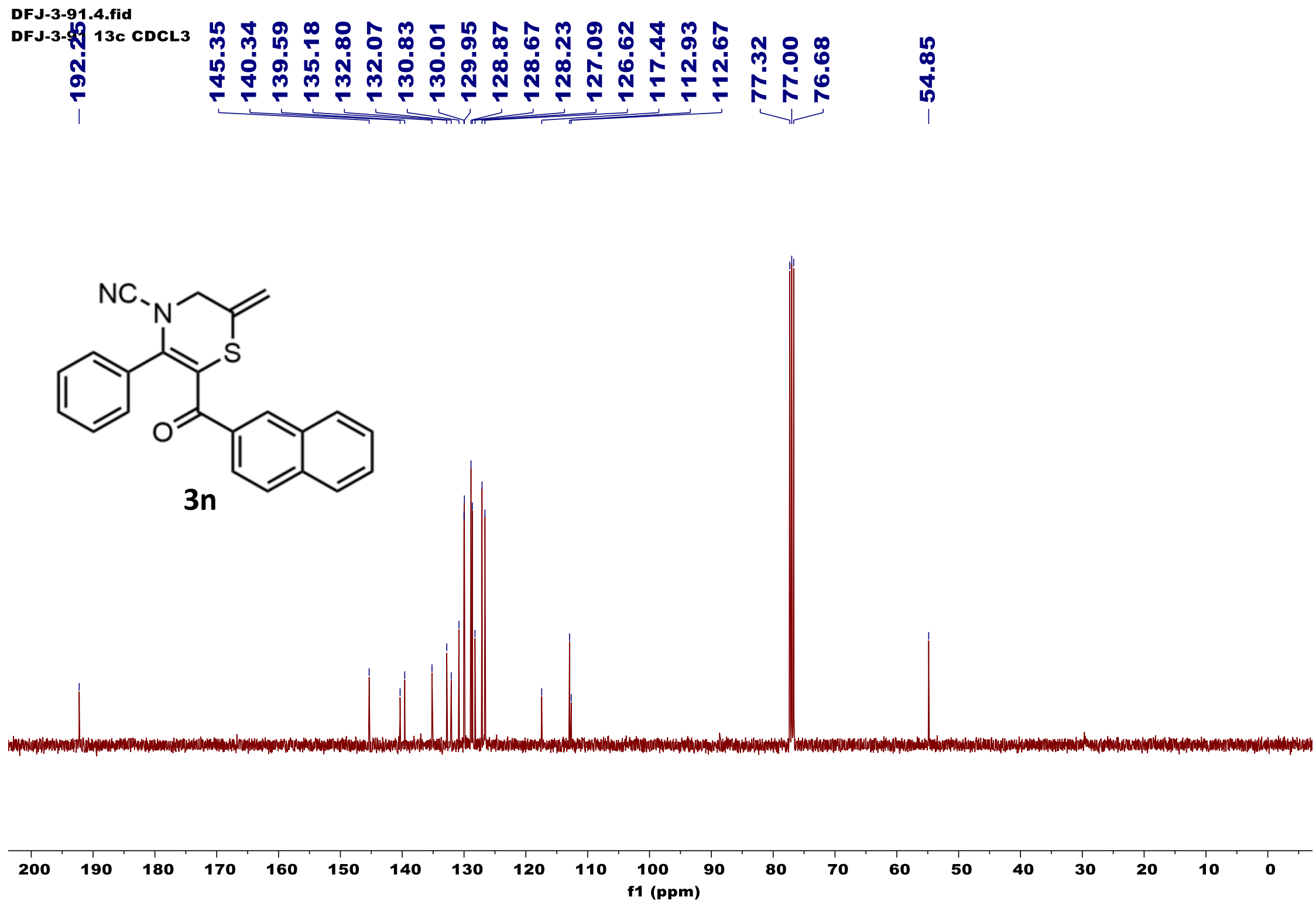

¹H NMR spectrum of compound 3o (400 MHz, $CDCl_3$)

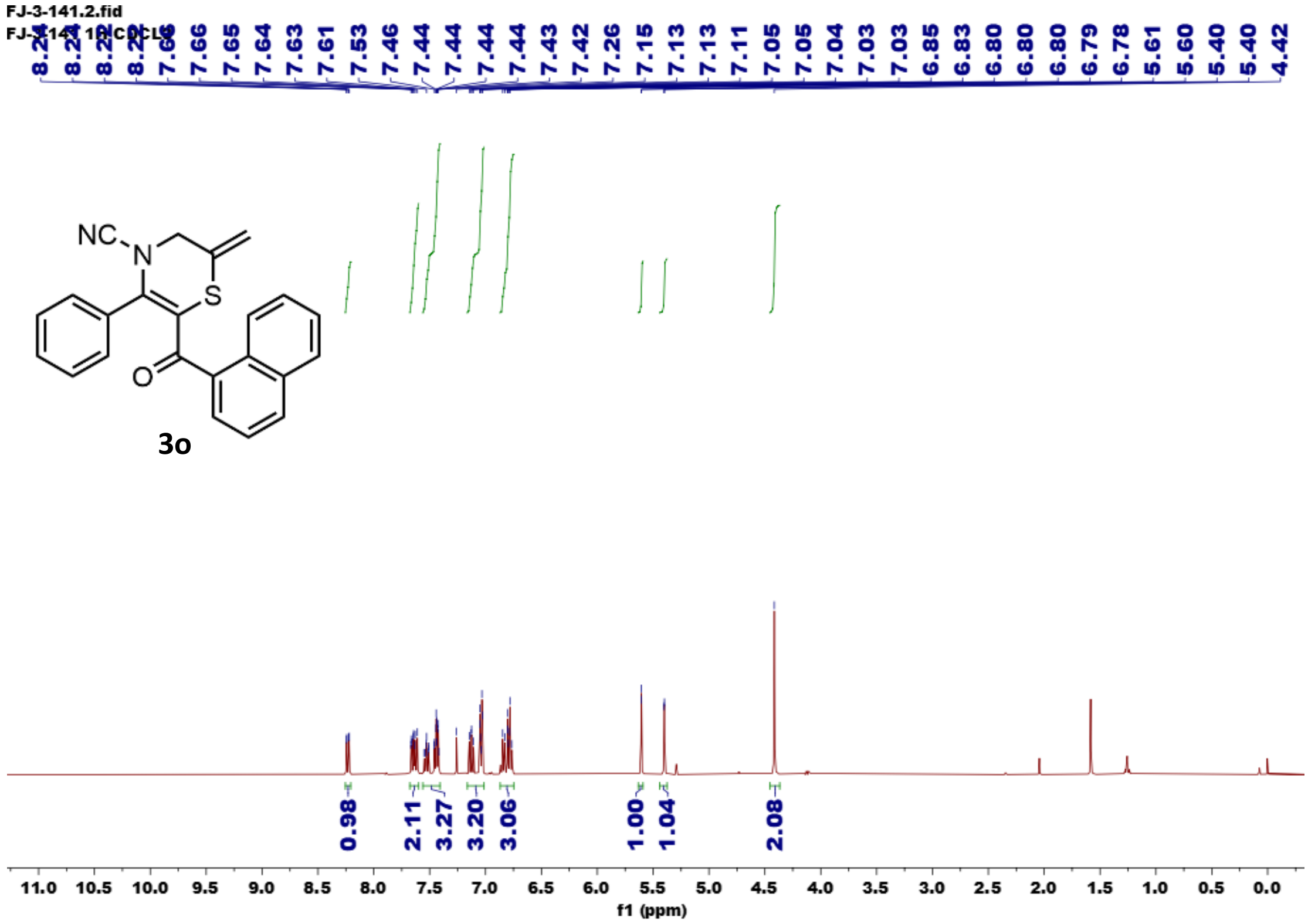

¹³C NMR spectrum of compound 3o (101 MHz, $CDCl_3$)

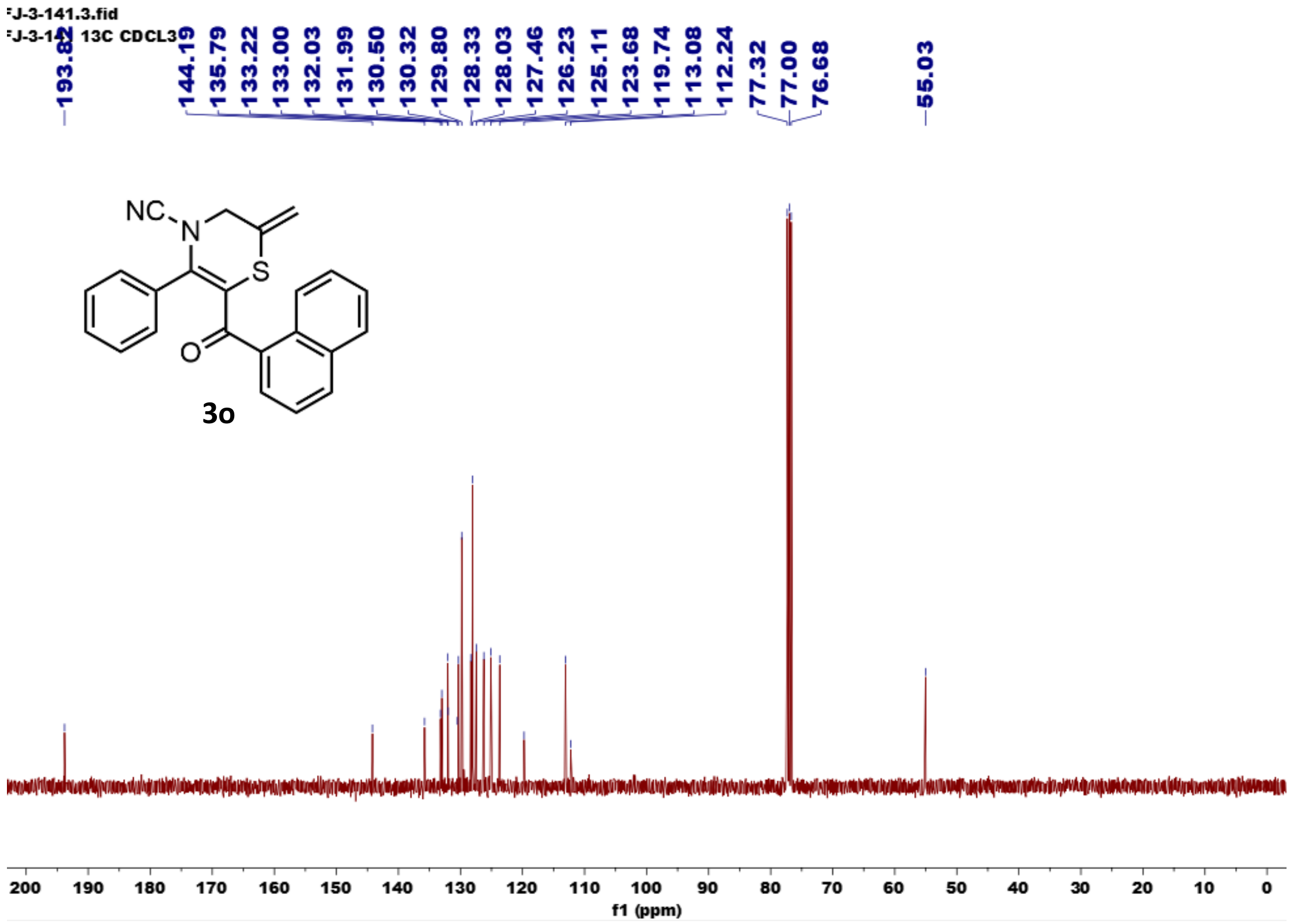

^{1}H NMR spectrum of compound 3p (400 MHz, $CDCl_3$)

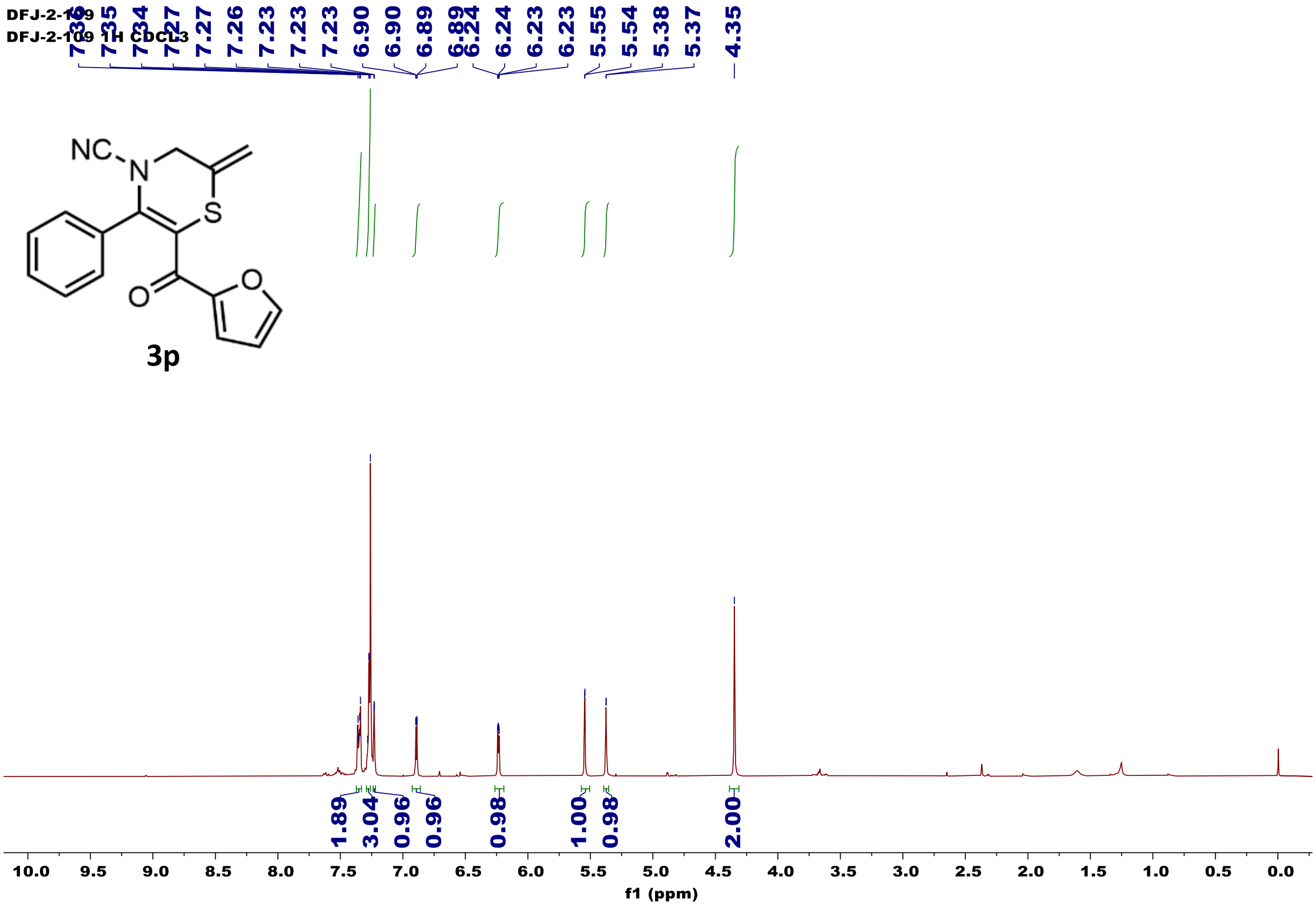

^{13}C NMR spectrum of compound 3p (101 MHz, $CDCl_3$)

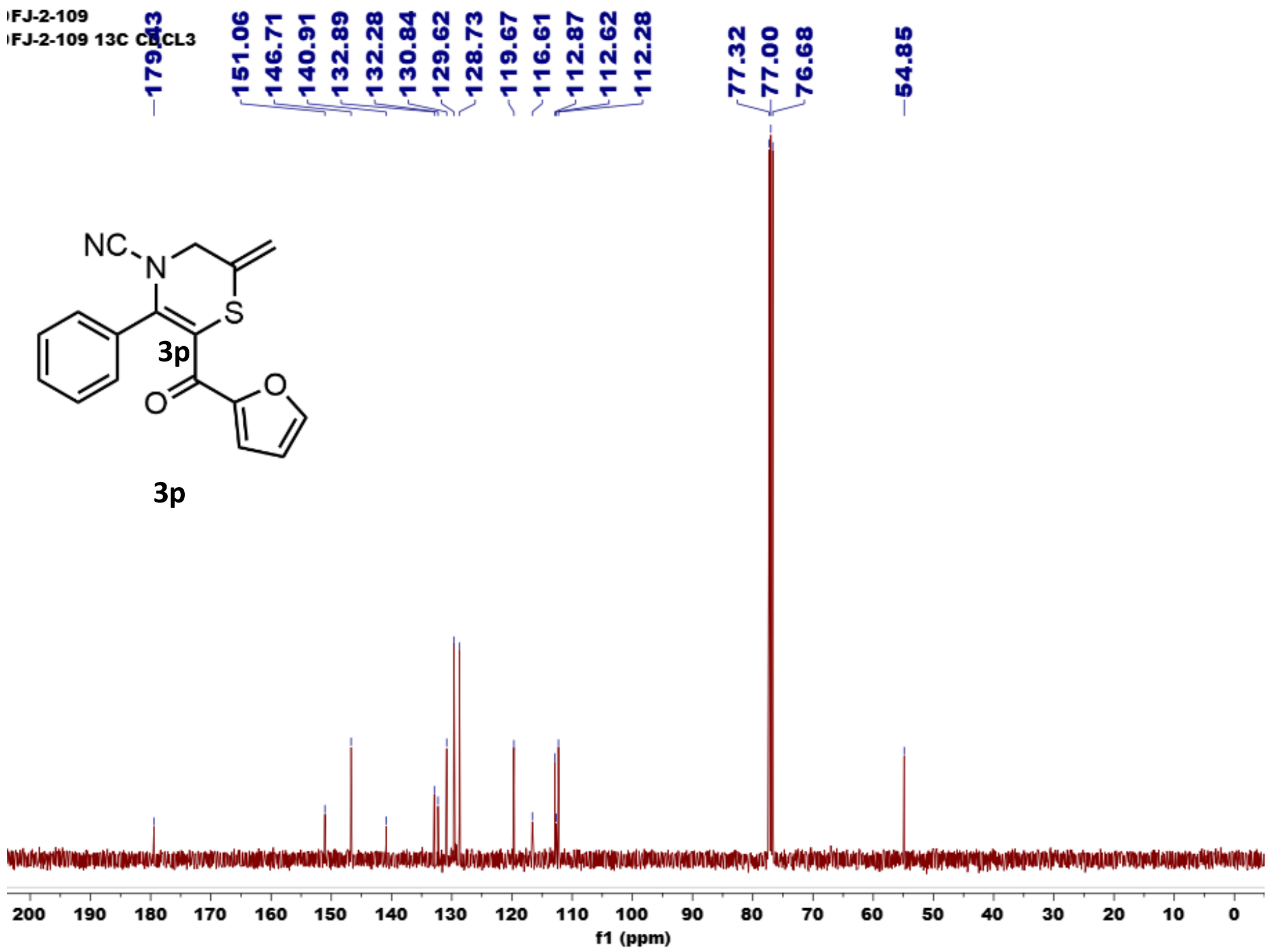

^{1}H NMR spectrum of compound 3q (400 MHz, $CDCl_3$)

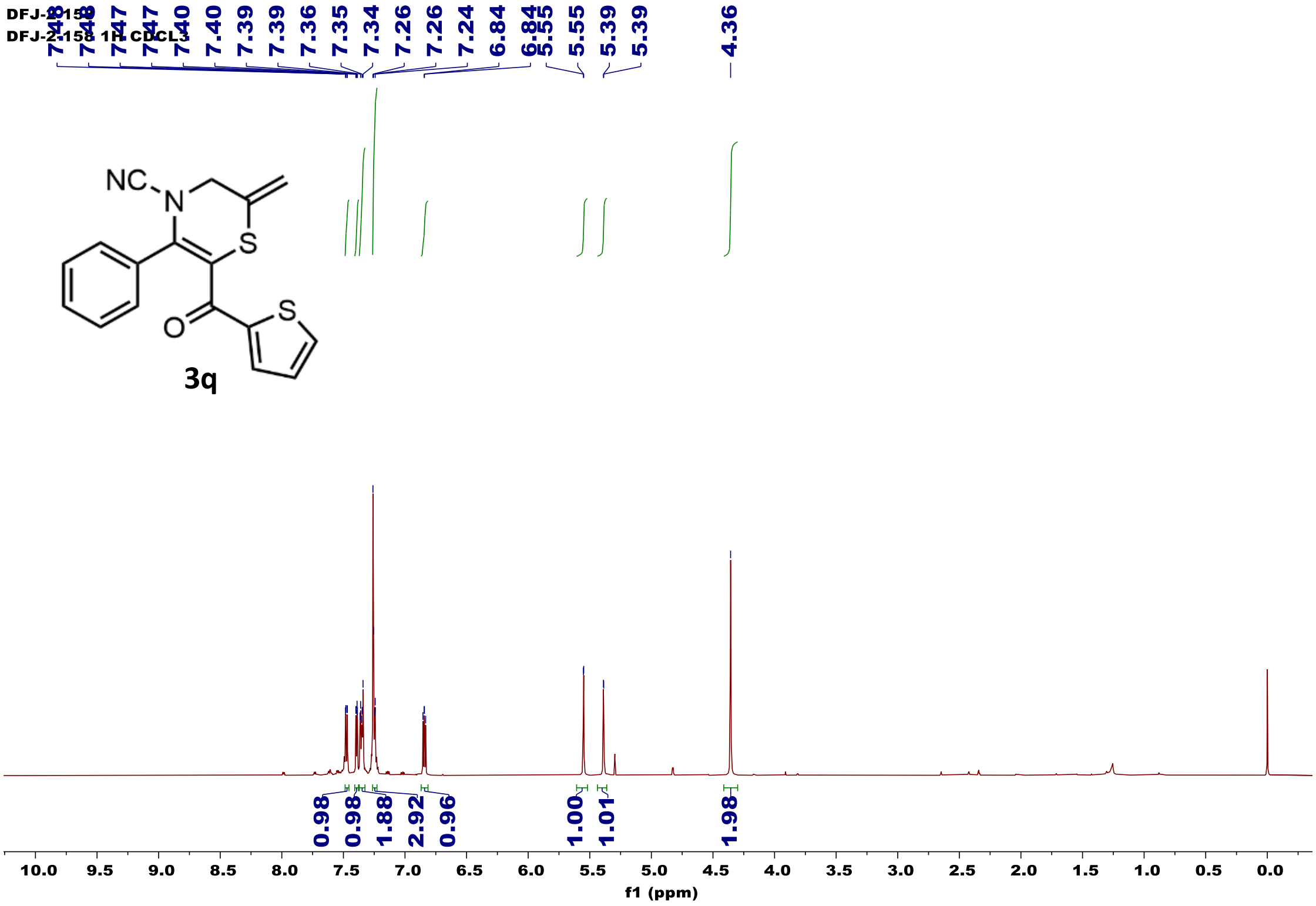

^{13}C NMR spectrum of compound 3q (101 MHz, $CDCl_3$)

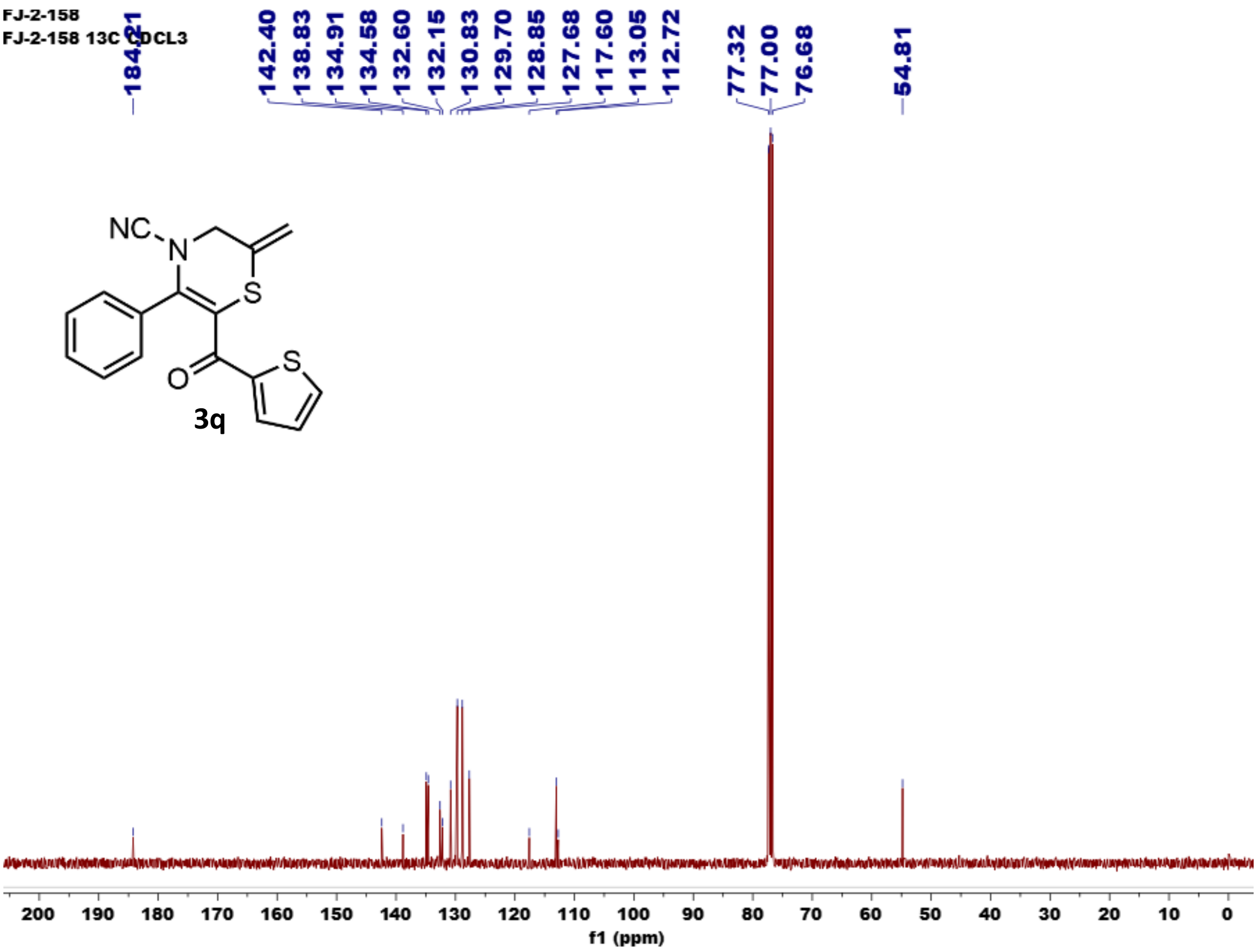

^{1}H NMR spectrum of compound 3r (400 MHz, $CDCl_3$)

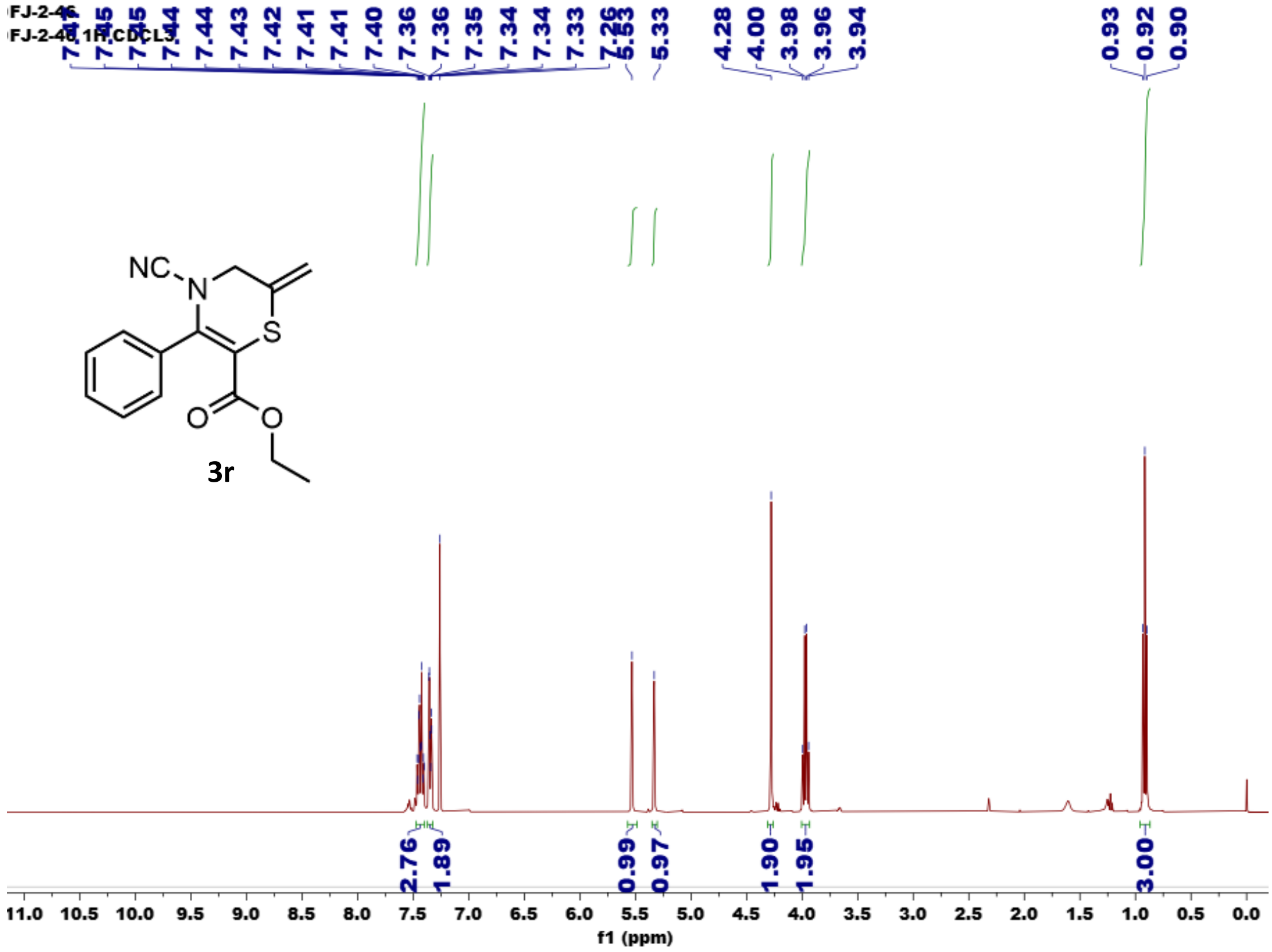

^{13}C NMR spectrum of compound 3r (101 MHz, $CDCl_3$)

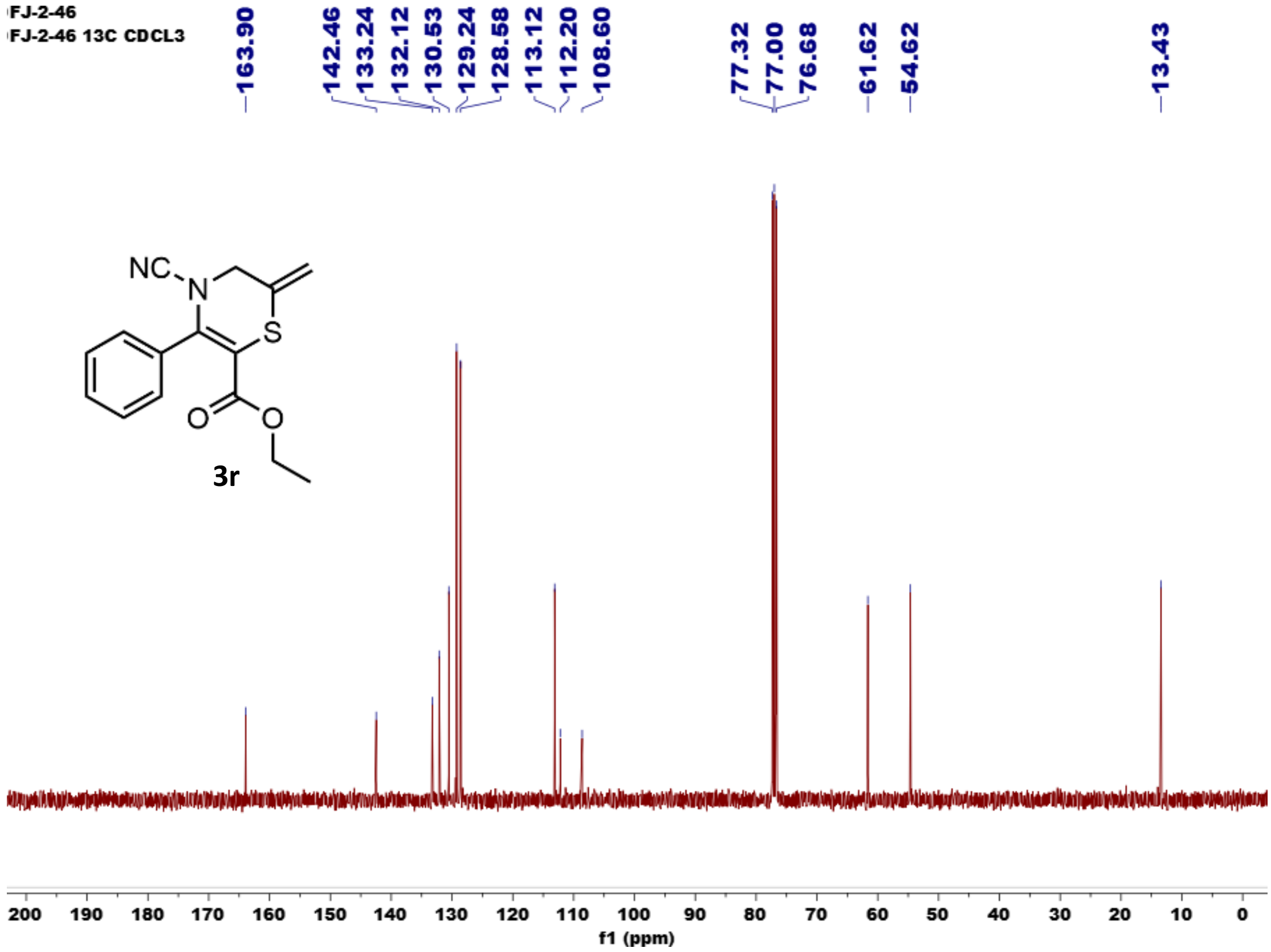

^{1}H NMR spectrum of compound 3s (400 MHz, $CDCl_3$)

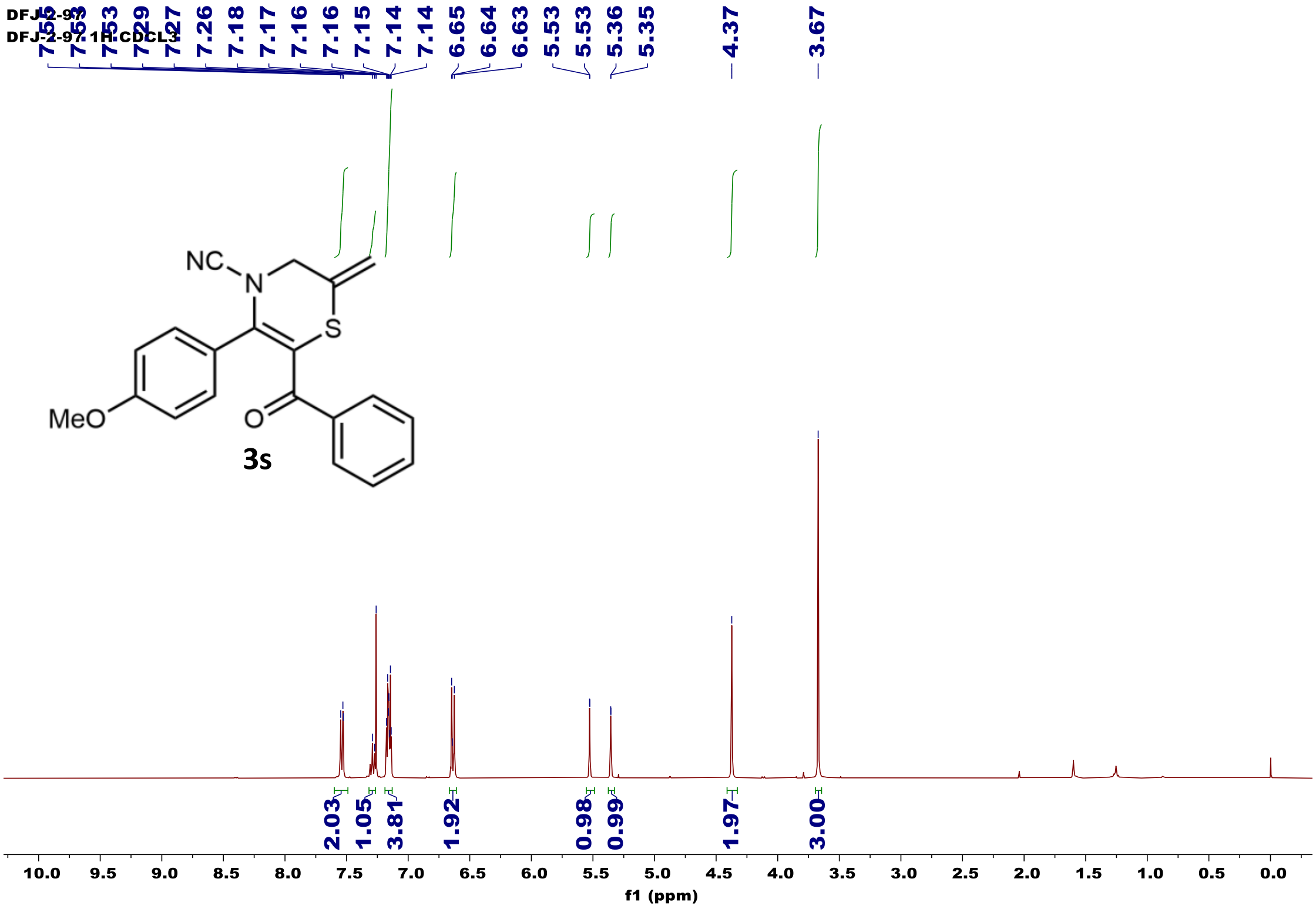

^{13}C NMR spectrum of compound 3s (101 MHz, $CDCl_3$)

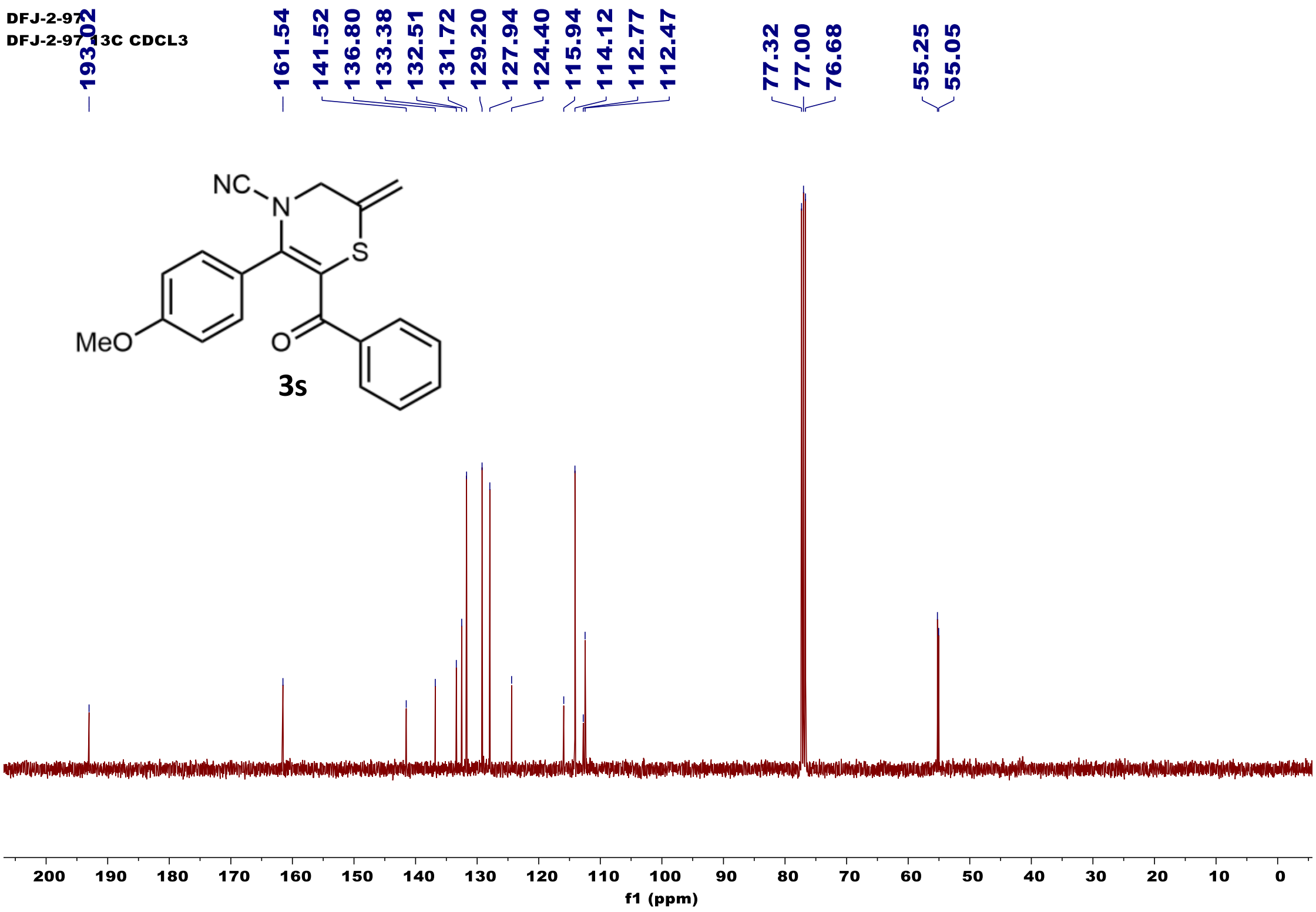

^{1}H NMR spectrum of compound 3t (400 MHz, $CDCl_3$)

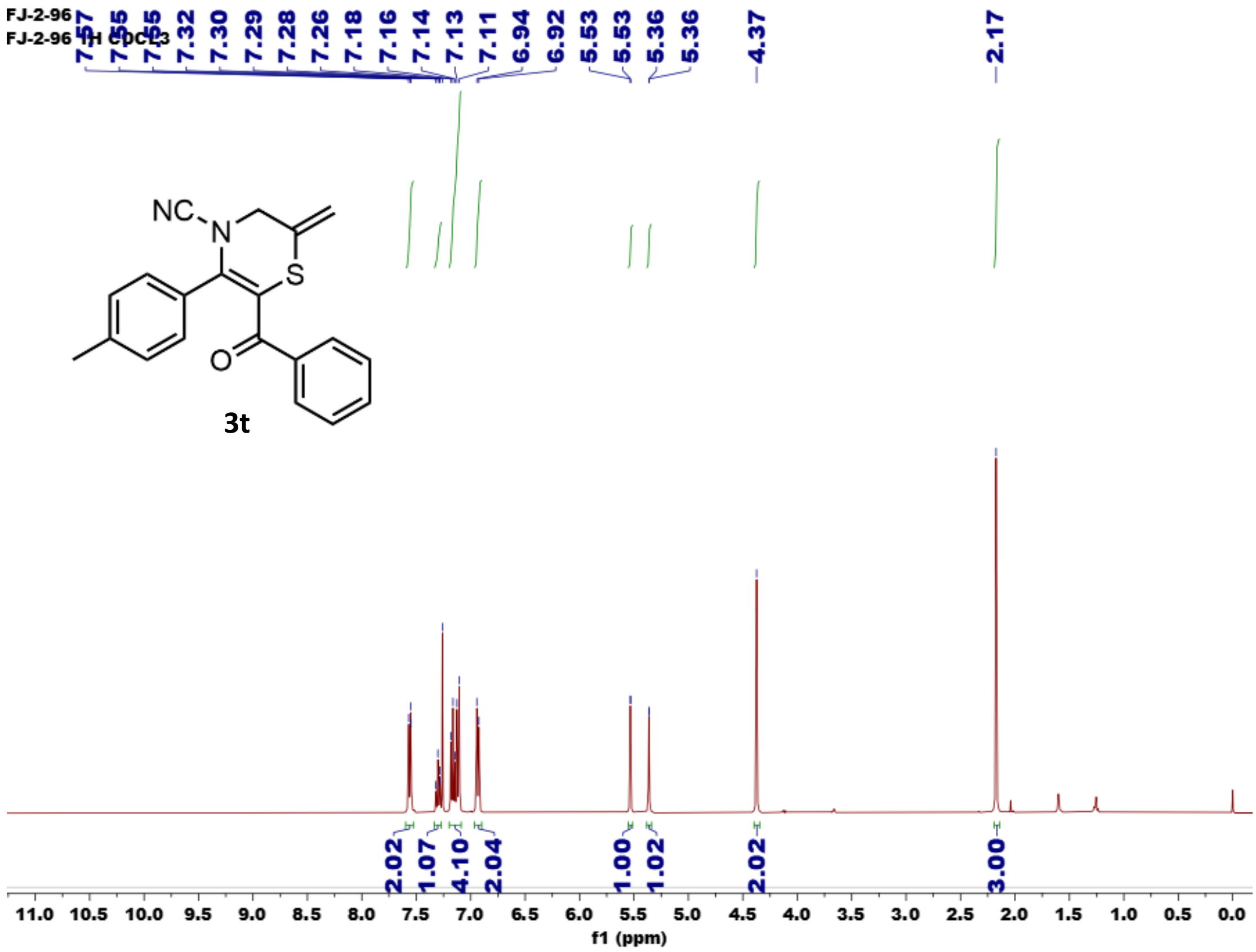

^{13}C NMR spectrum of compound 3t (101 MHz, $CDCl_3$)

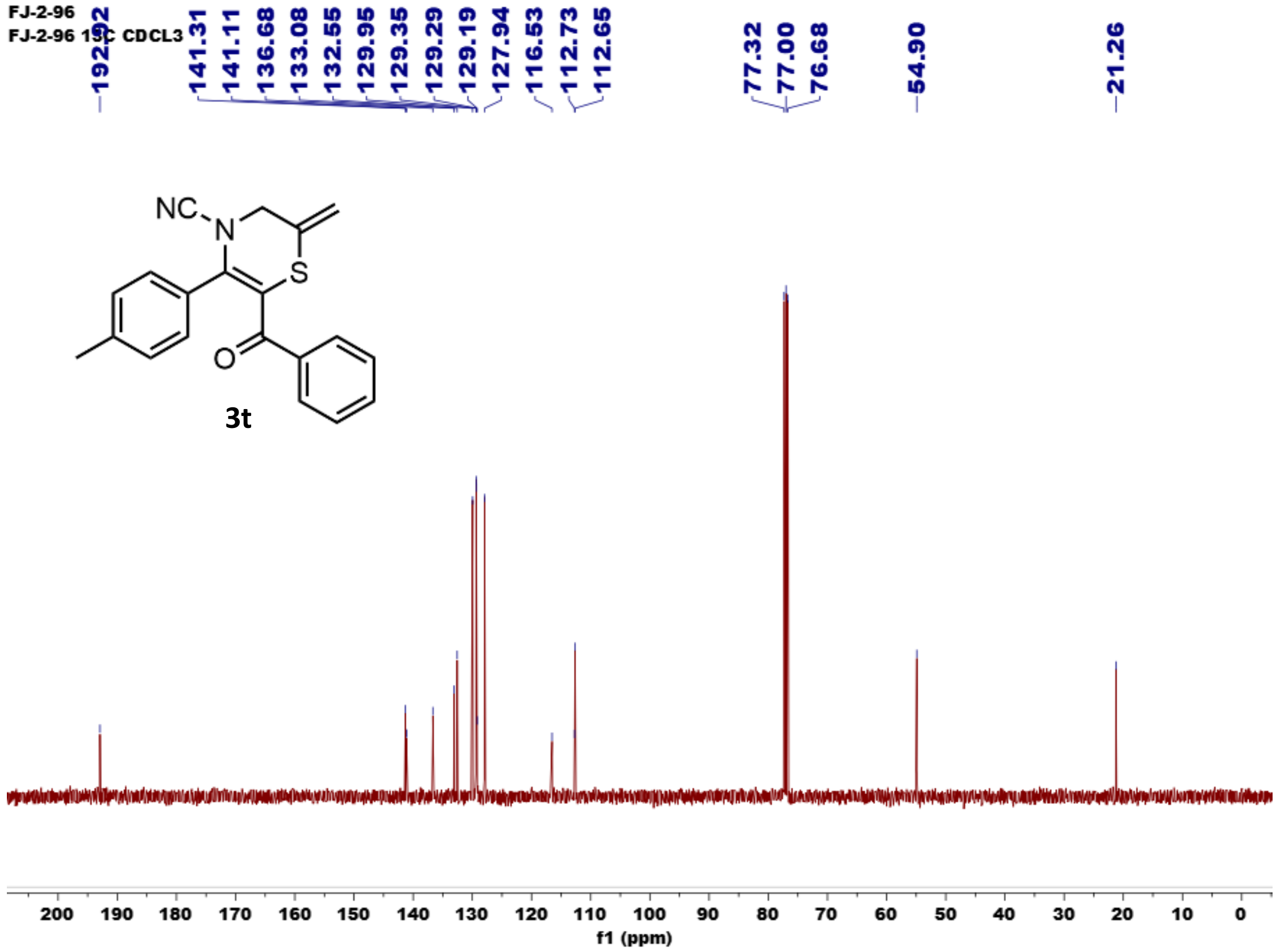

^{1}H NMR spectrum of compound 3u (400 MHz, $CDCl_3$)

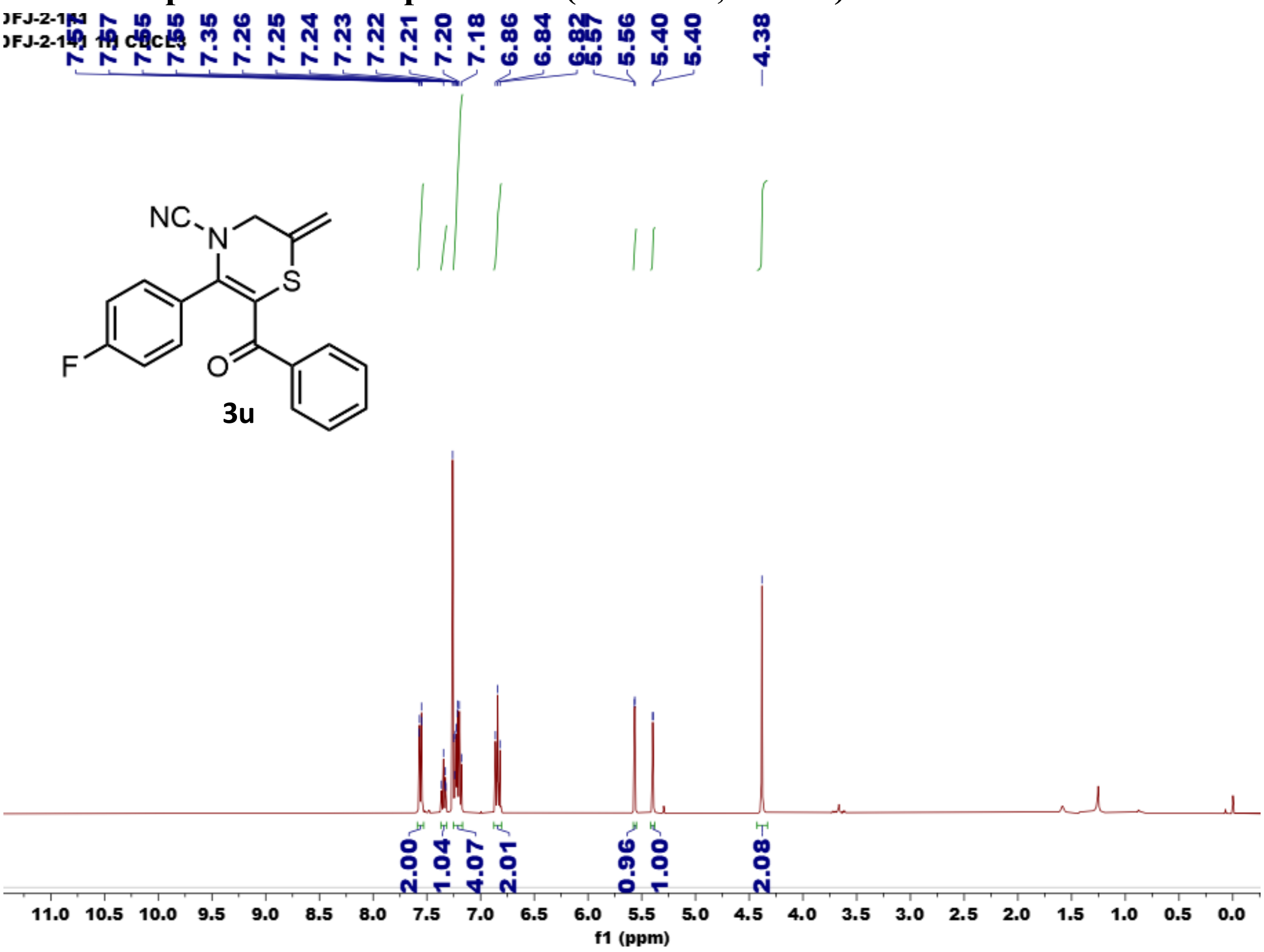

^{13}C NMR spectrum of compound 3u (101 MHz, $CDCl_3$)

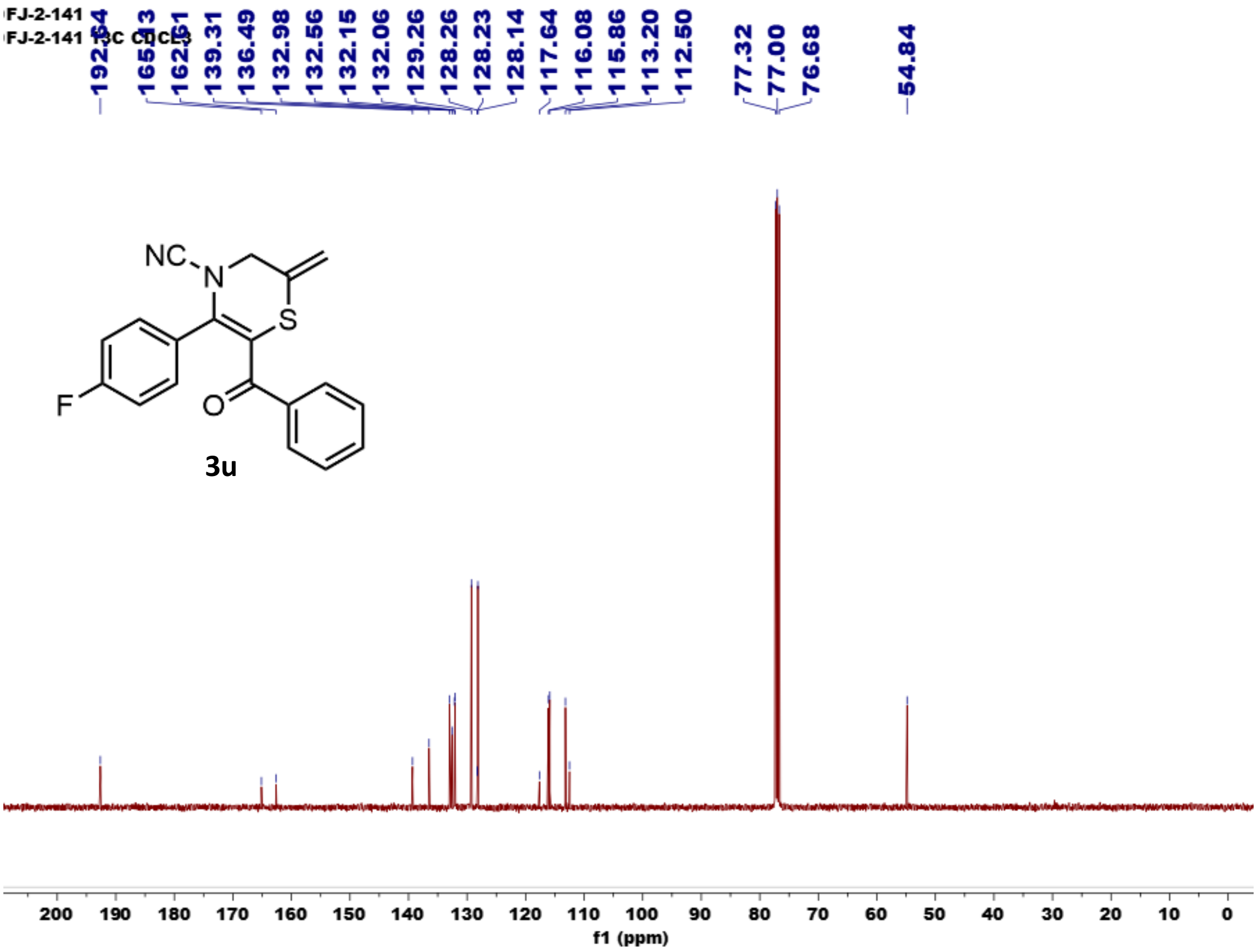

^{19}F NMR spectrum of compound 3u (376 MHz, $CDCl_3$)

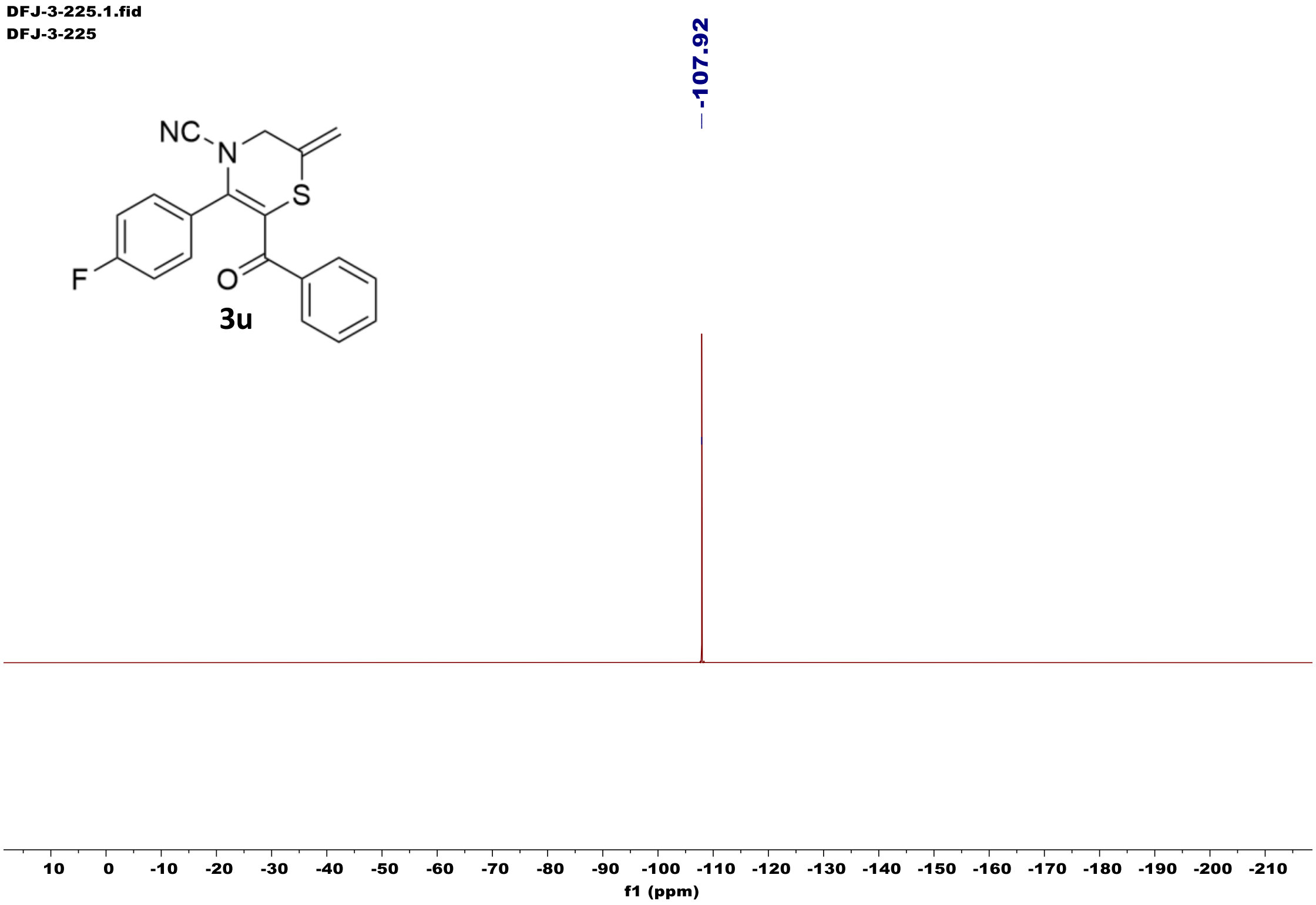

^{1}H NMR spectrum of compound 3v (400 MHz, $CDCl_3$)

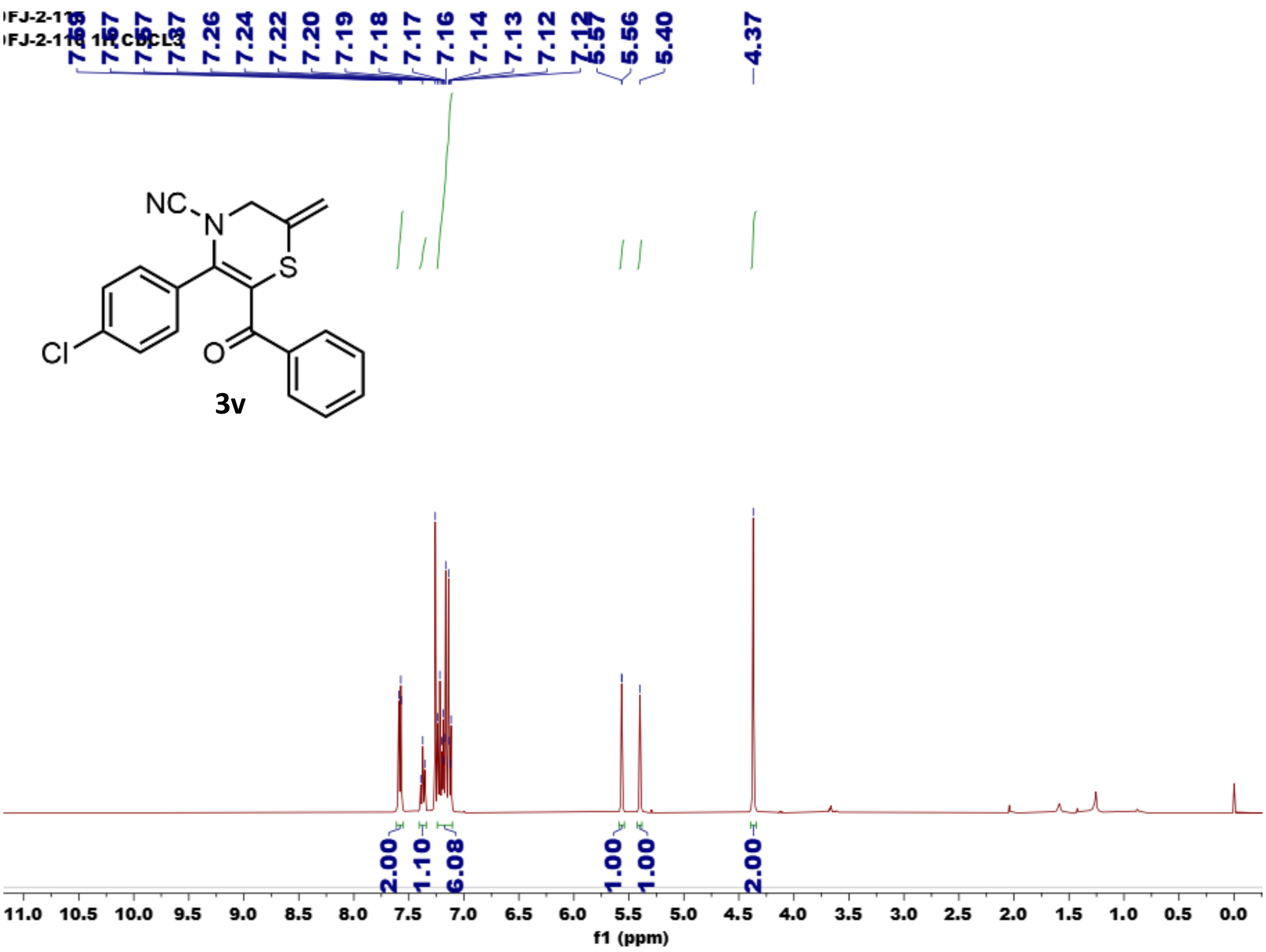

^{13}C NMR spectrum of compound 3v (101 MHz, $CDCl_3$)

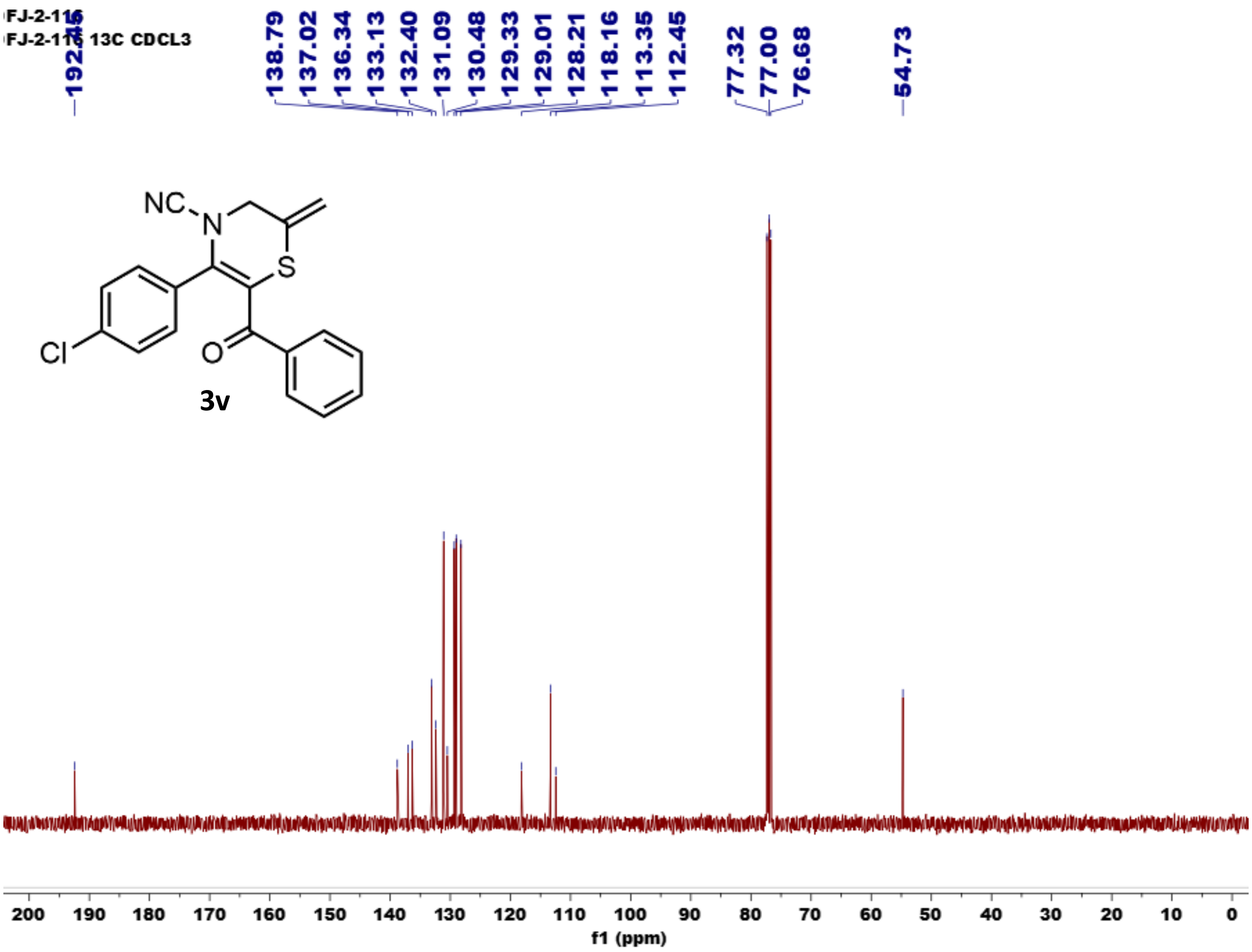

^{1}H NMR spectrum of compound 3w (400 MHz, $CDCl_3$)

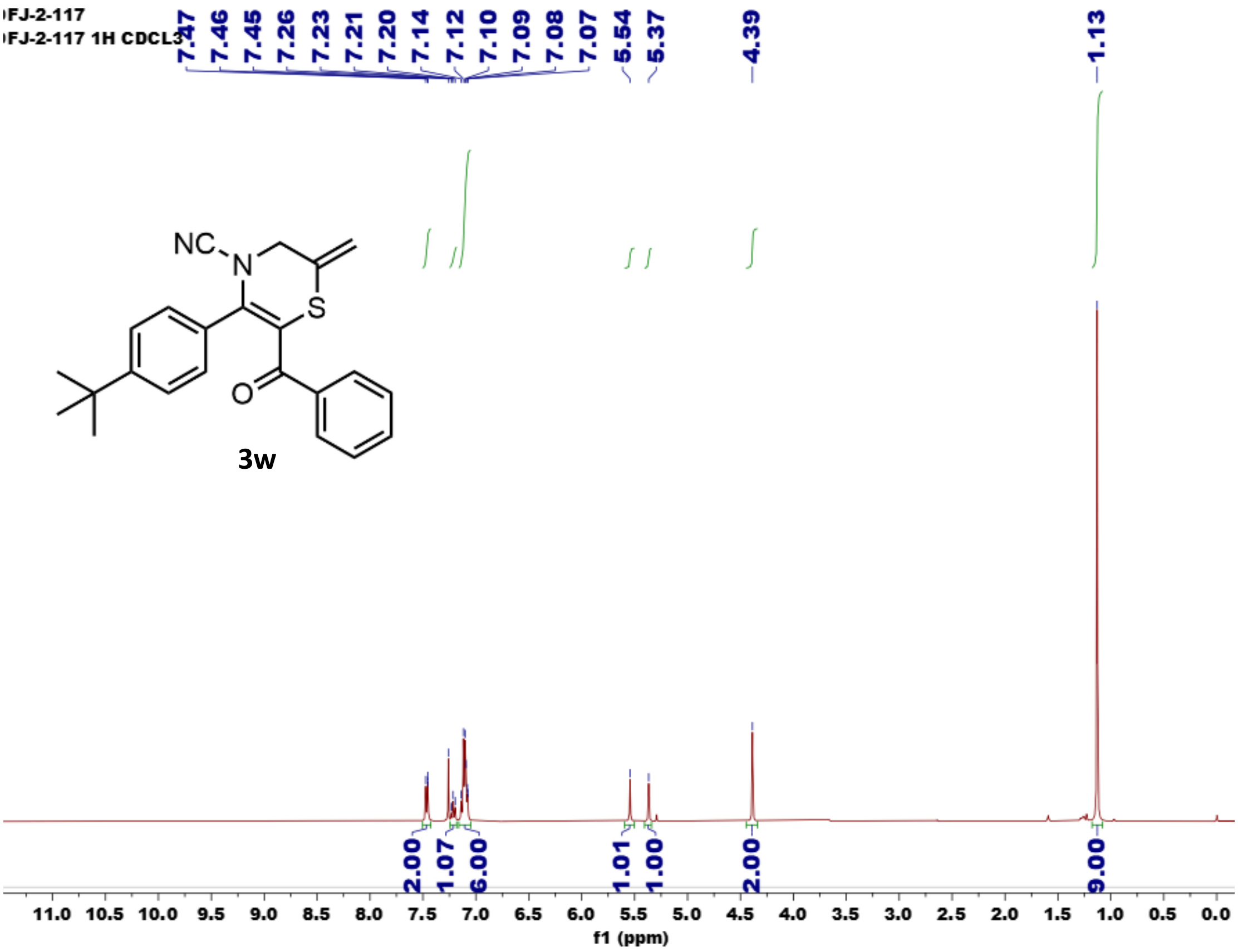

^{13}C NMR spectrum of compound 3w (101 MHz, $CDCl_3$)

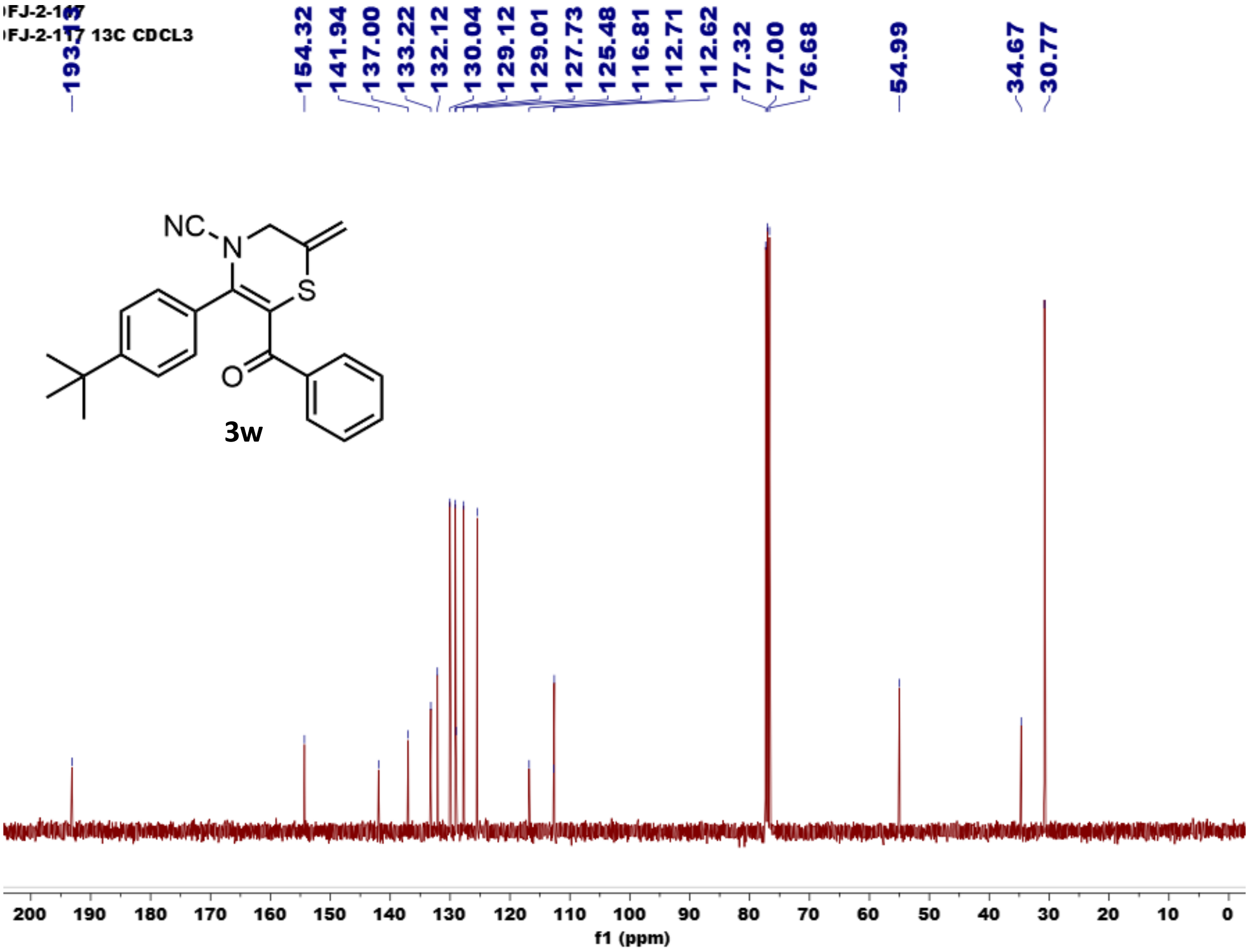

^{1}H NMR spectrum of compound 3x (400 MHz, $CDCl_3$)

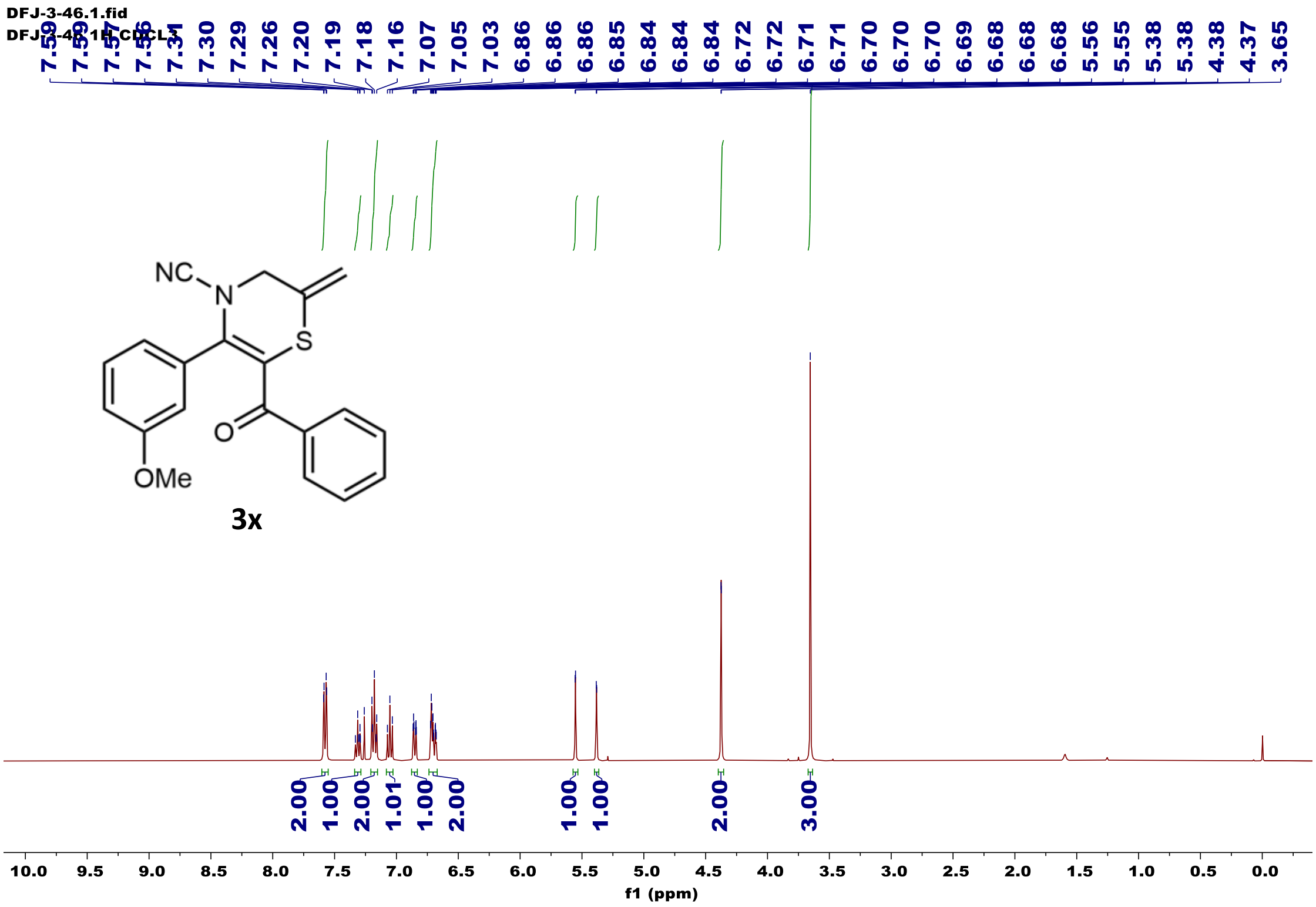

^{13}C NMR spectrum of compound 3x (101 MHz, $CDCl_3$)

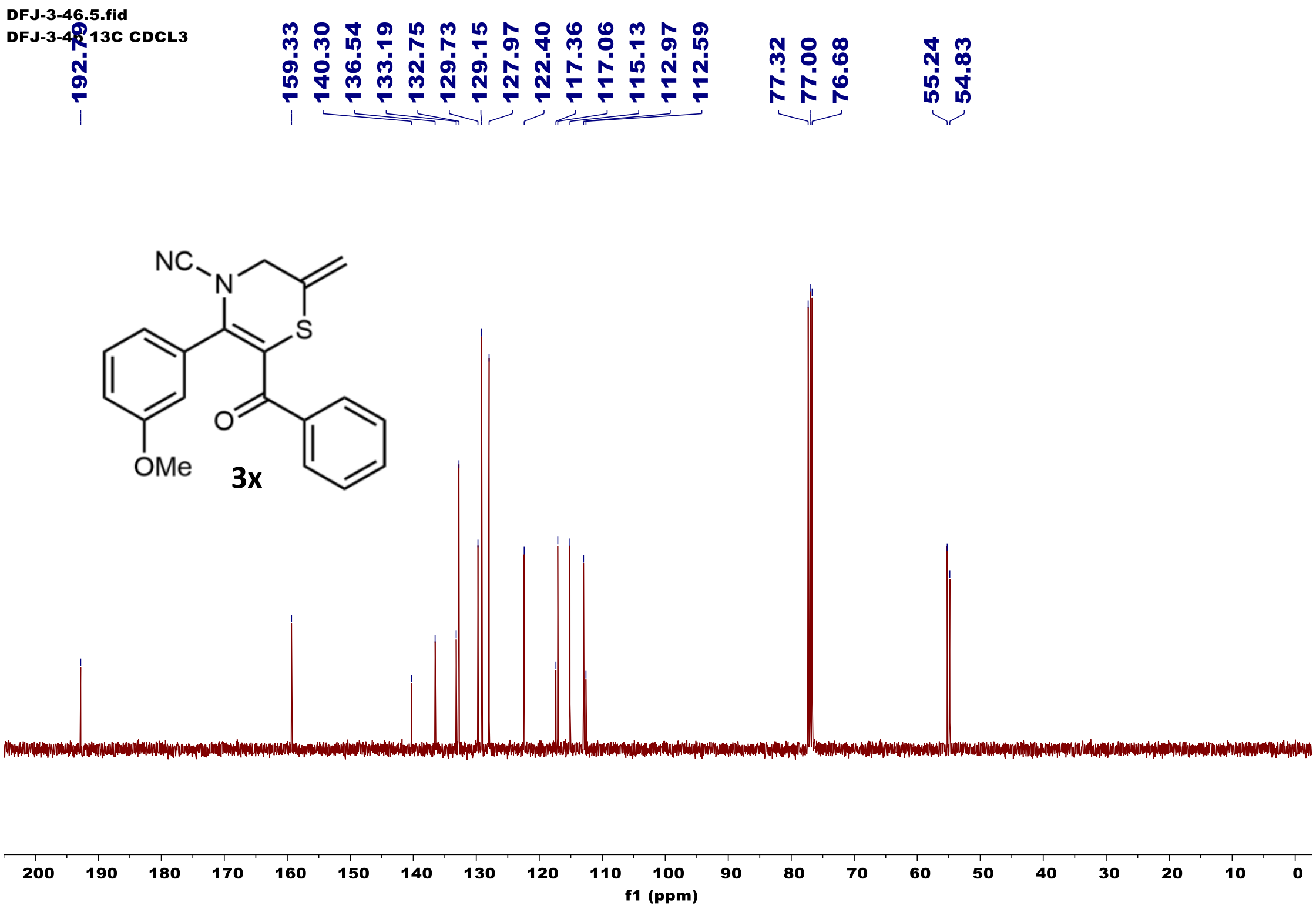

^{1}H NMR spectrum of compound 3y (400 MHz, $CDCl_3$)

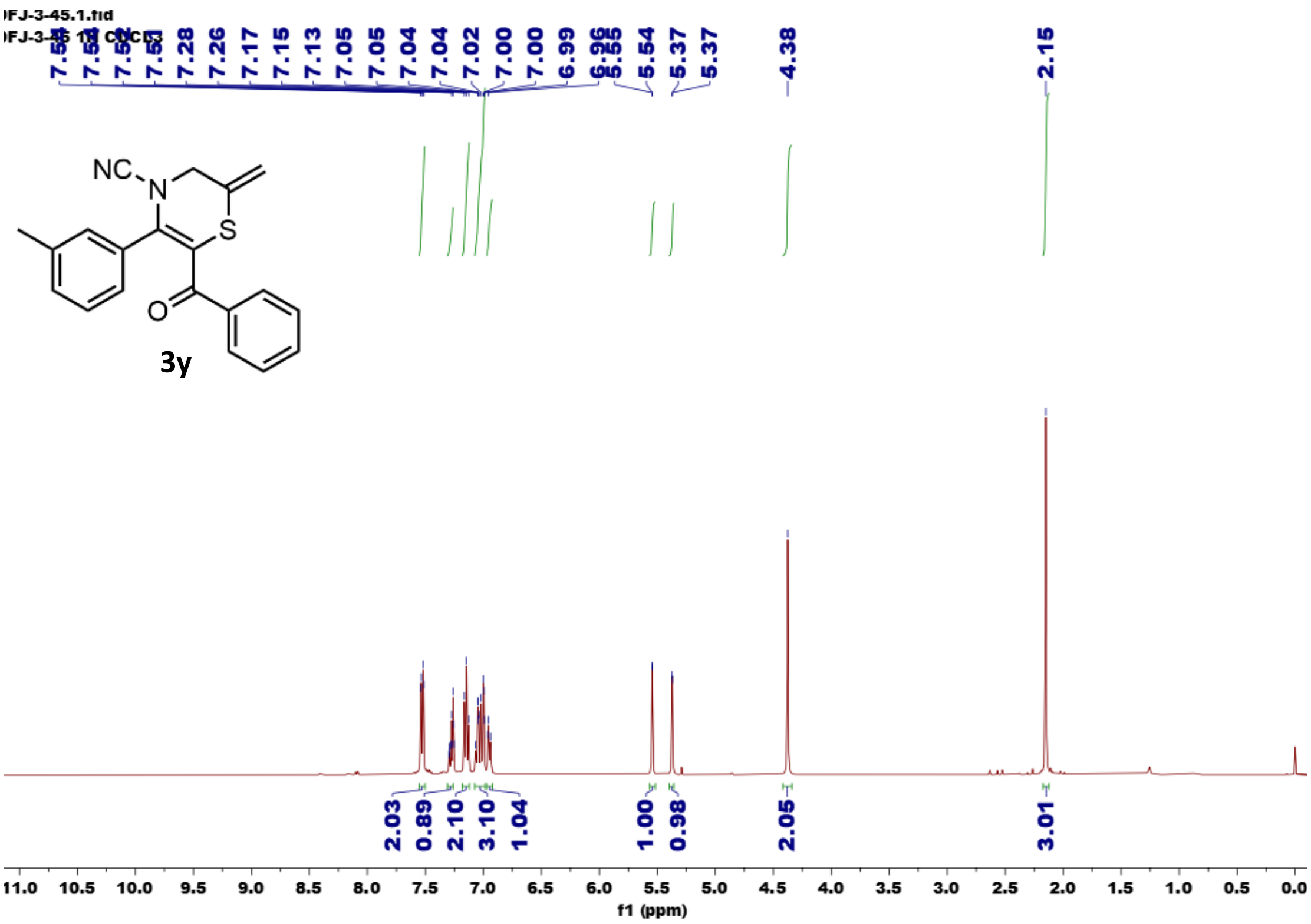

^{13}C NMR spectrum of compound 3y (101 MHz, $CDCl_3$)

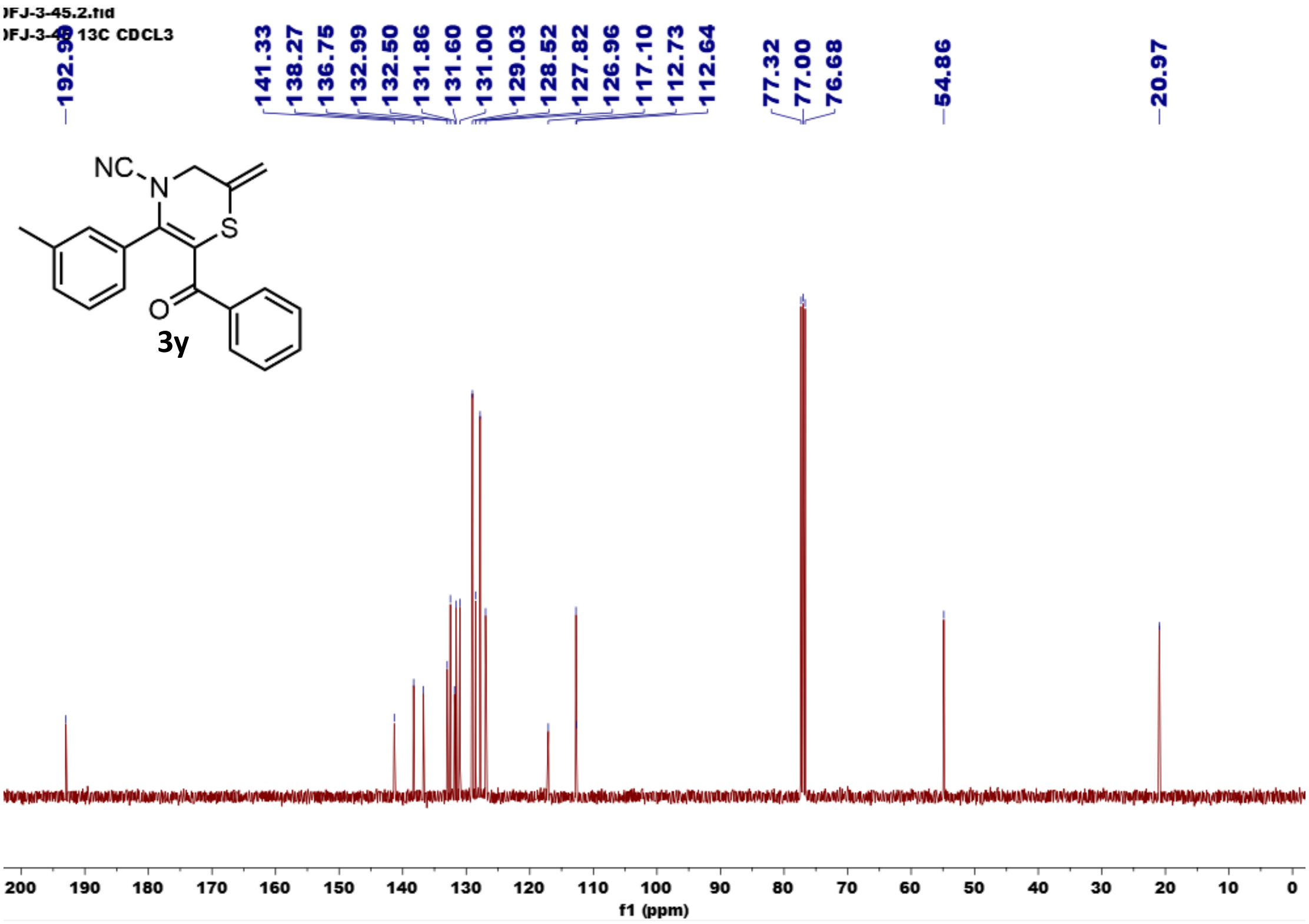

^{1}H NMR spectrum of compound 3z (400 MHz, $CDCl_3$)

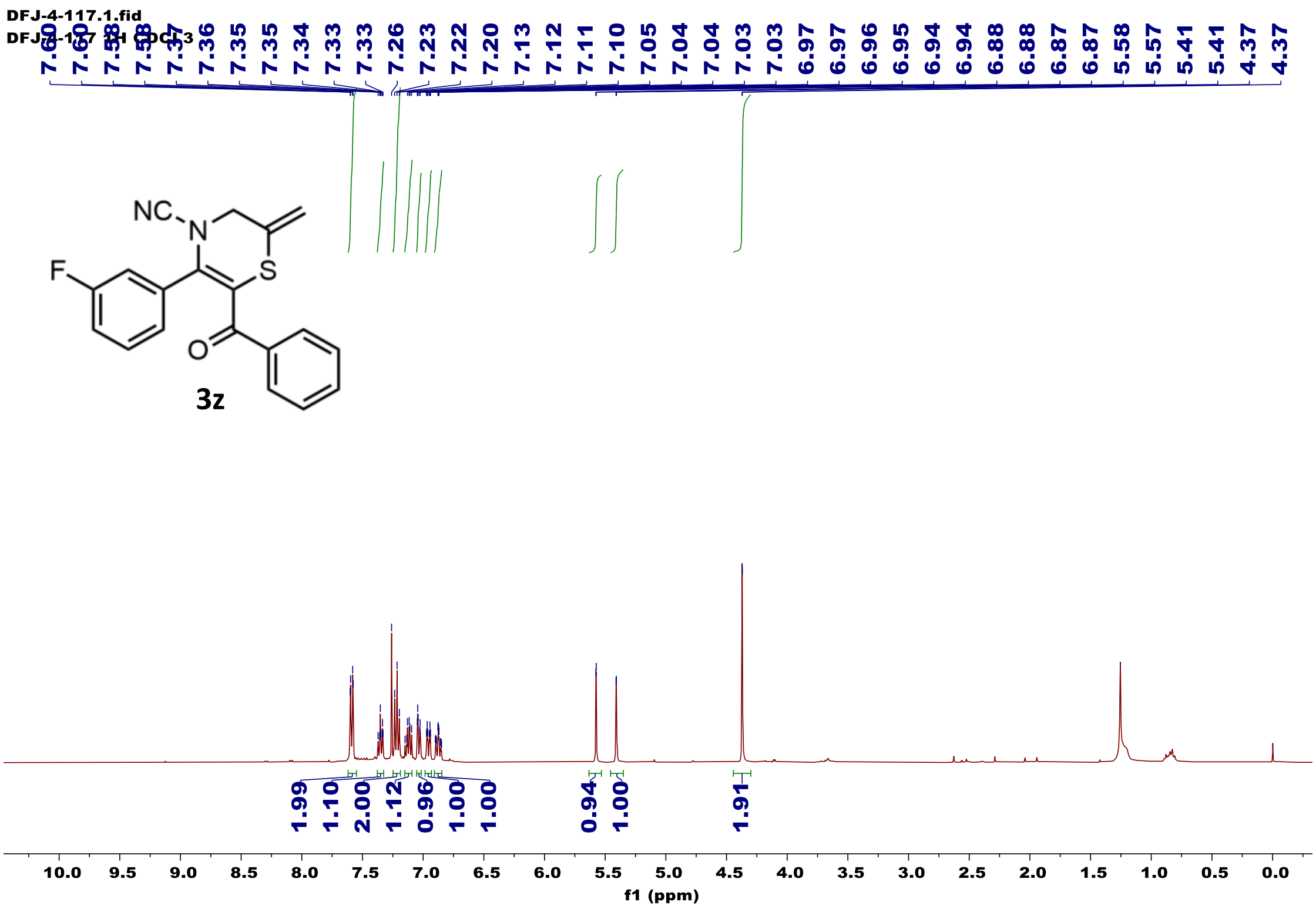

^{13}C NMR spectrum of compound 3z (101 MHz, $CDCl_3$)

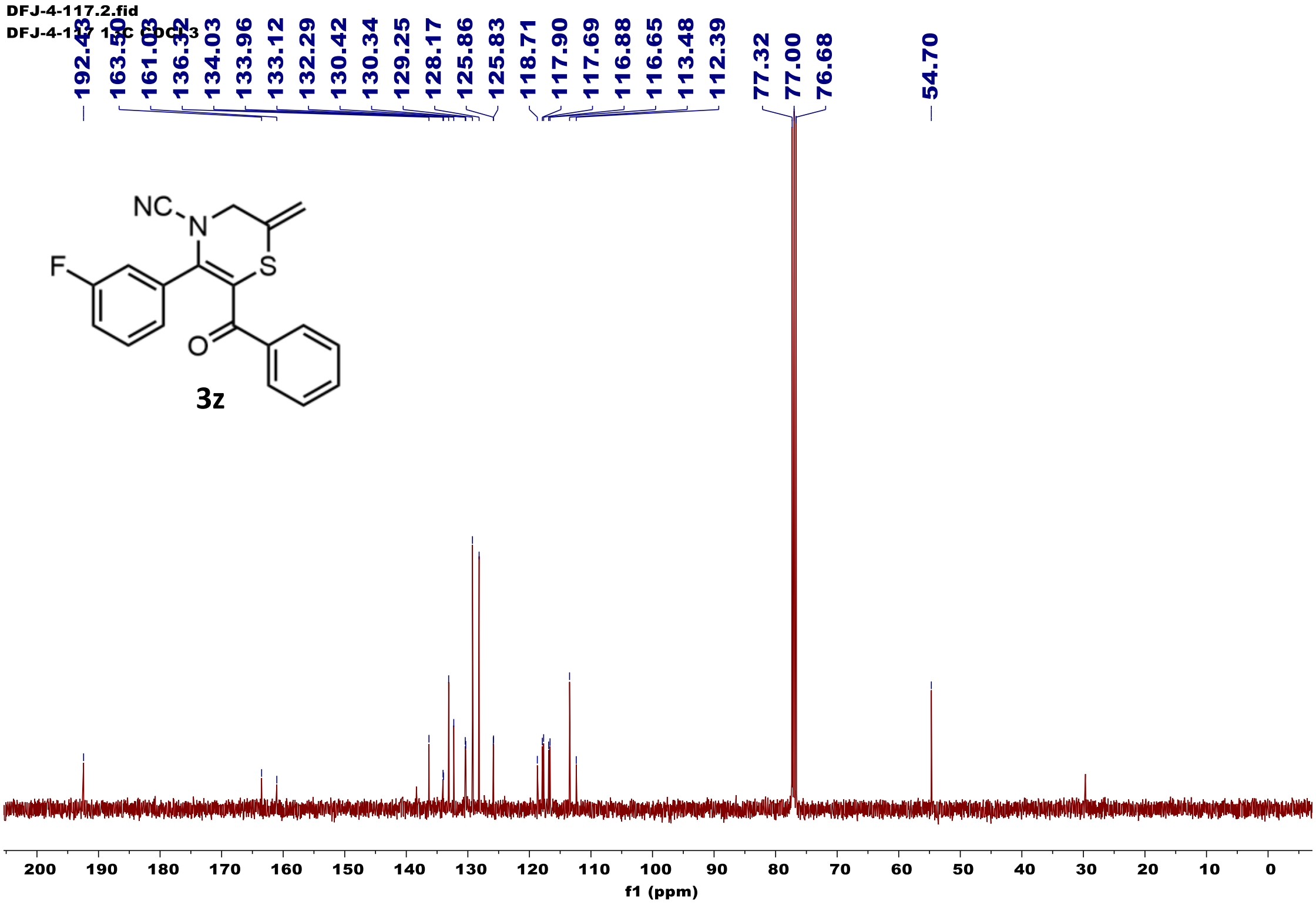

^{19}F NMR spectrum of compound 3u (376 MHz, $CDCl_3$)

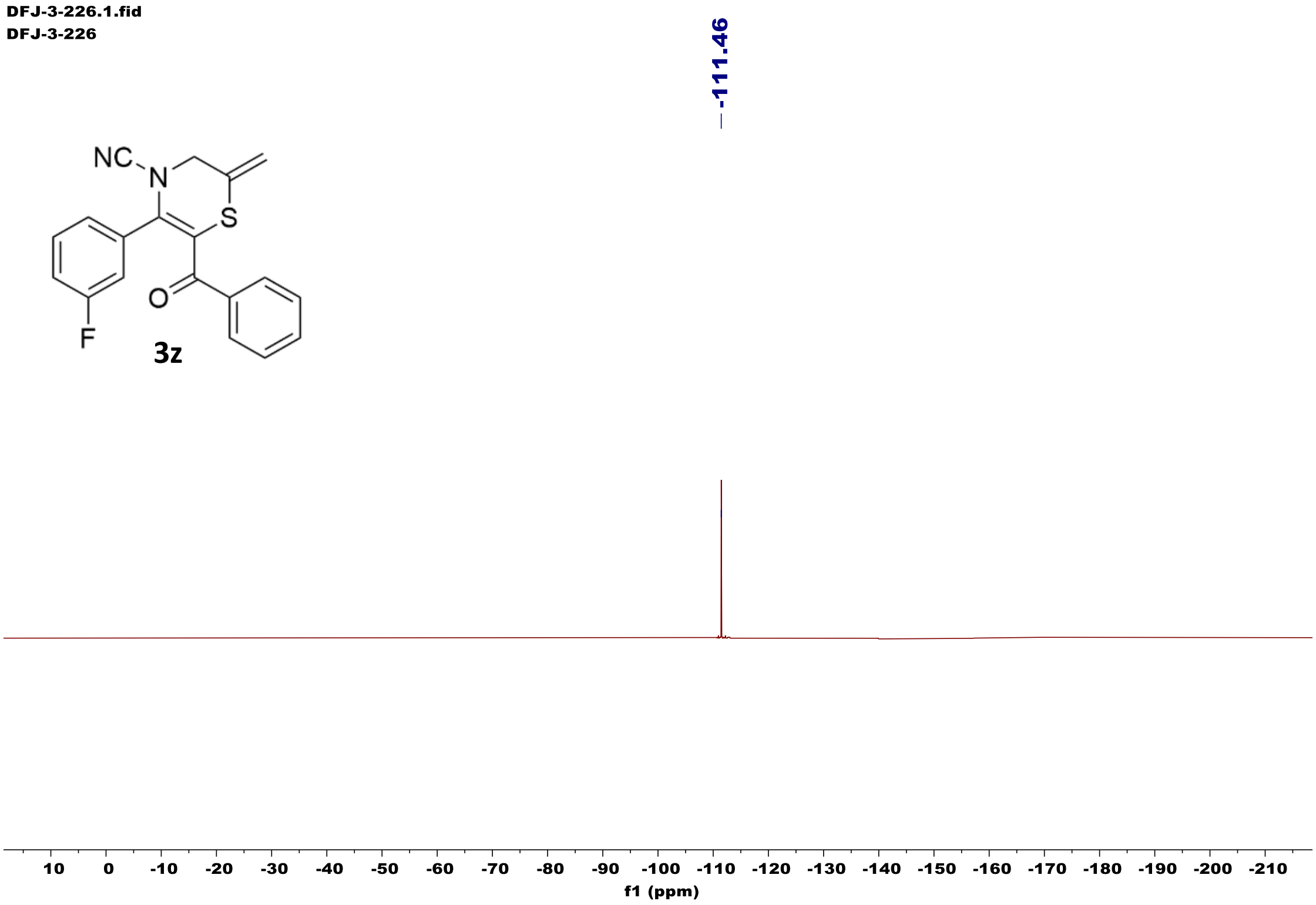

^1H NMR spectrum of compound 3aa (400 MHz, $CDCl_3$)

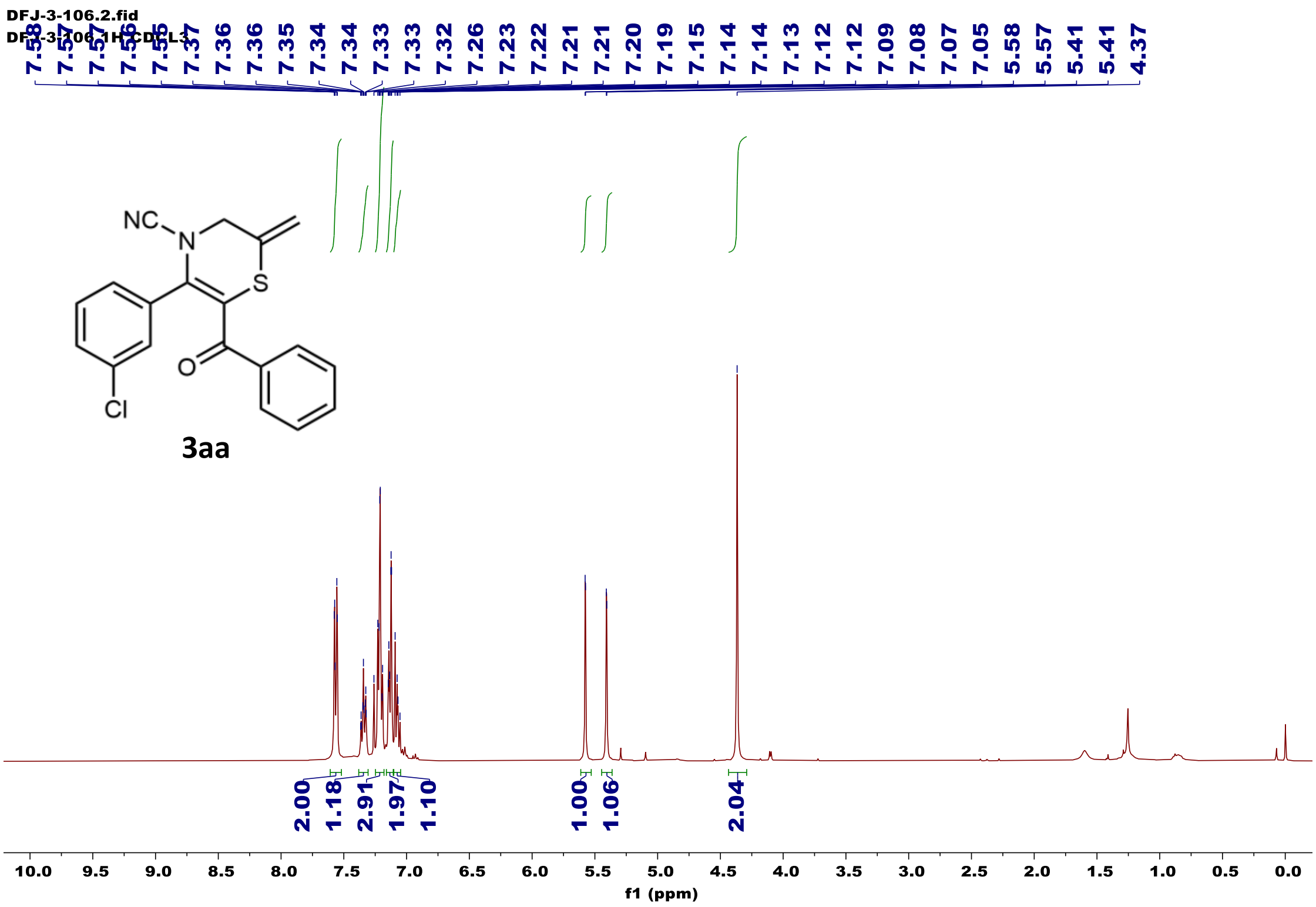

^{13}C NMR spectrum of compound 3aa (101 MHz, $CDCl_3$)

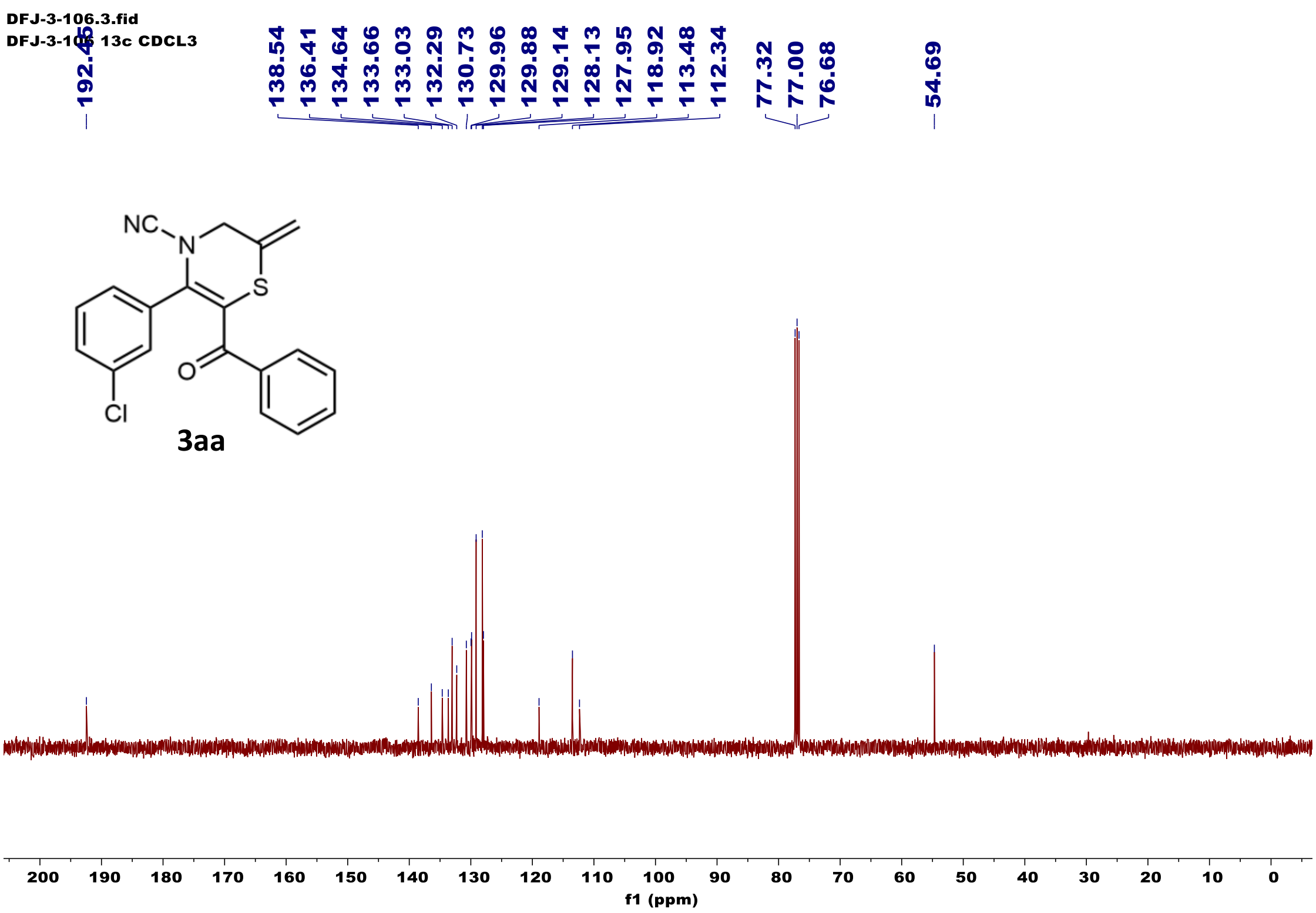

^{1}H NMR spectrum of compound 3ab (400 MHz, $CDCl_3$)

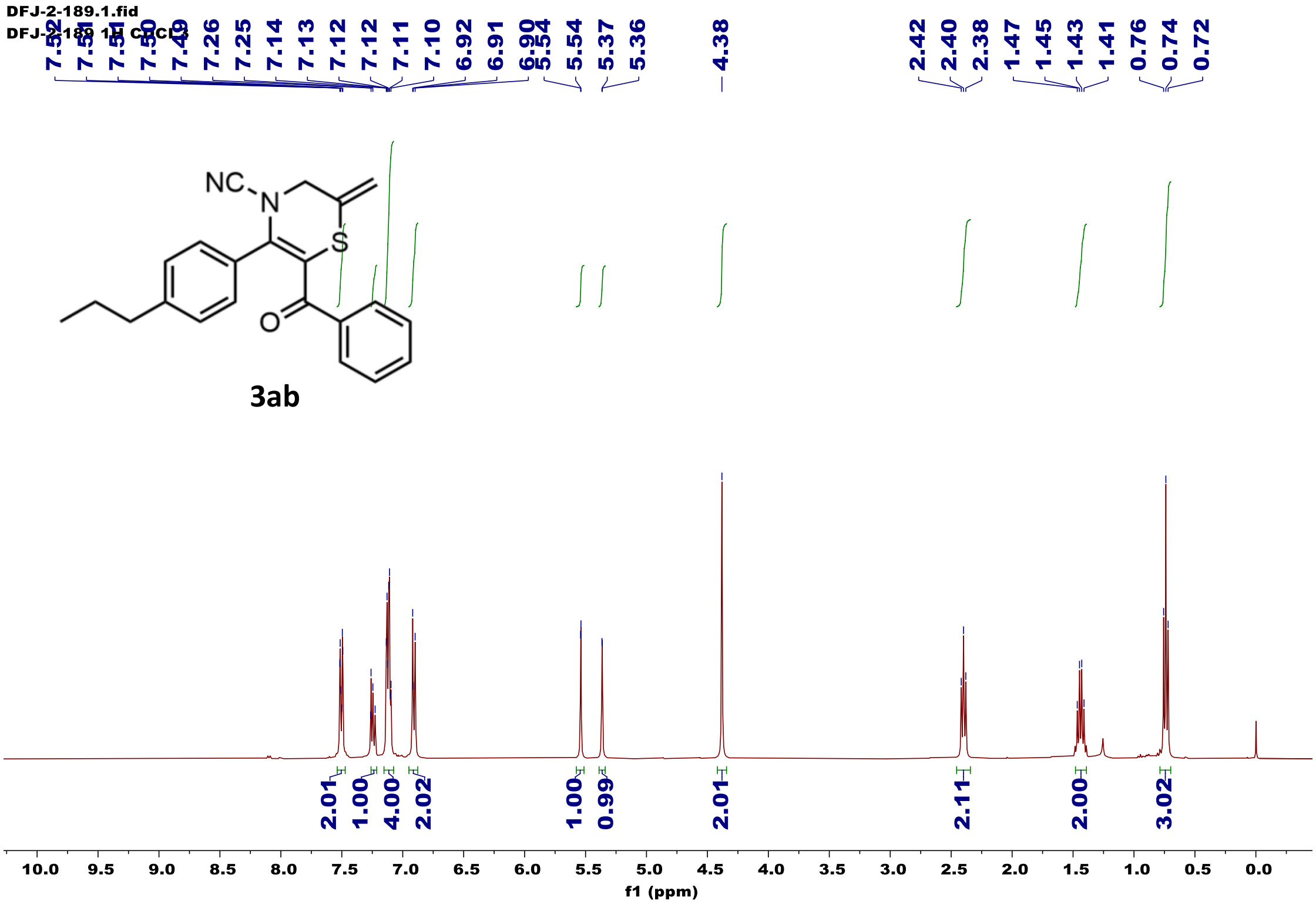

^{13}C NMR spectrum of compound 3ab (101 MHz, $CDCl_3$)

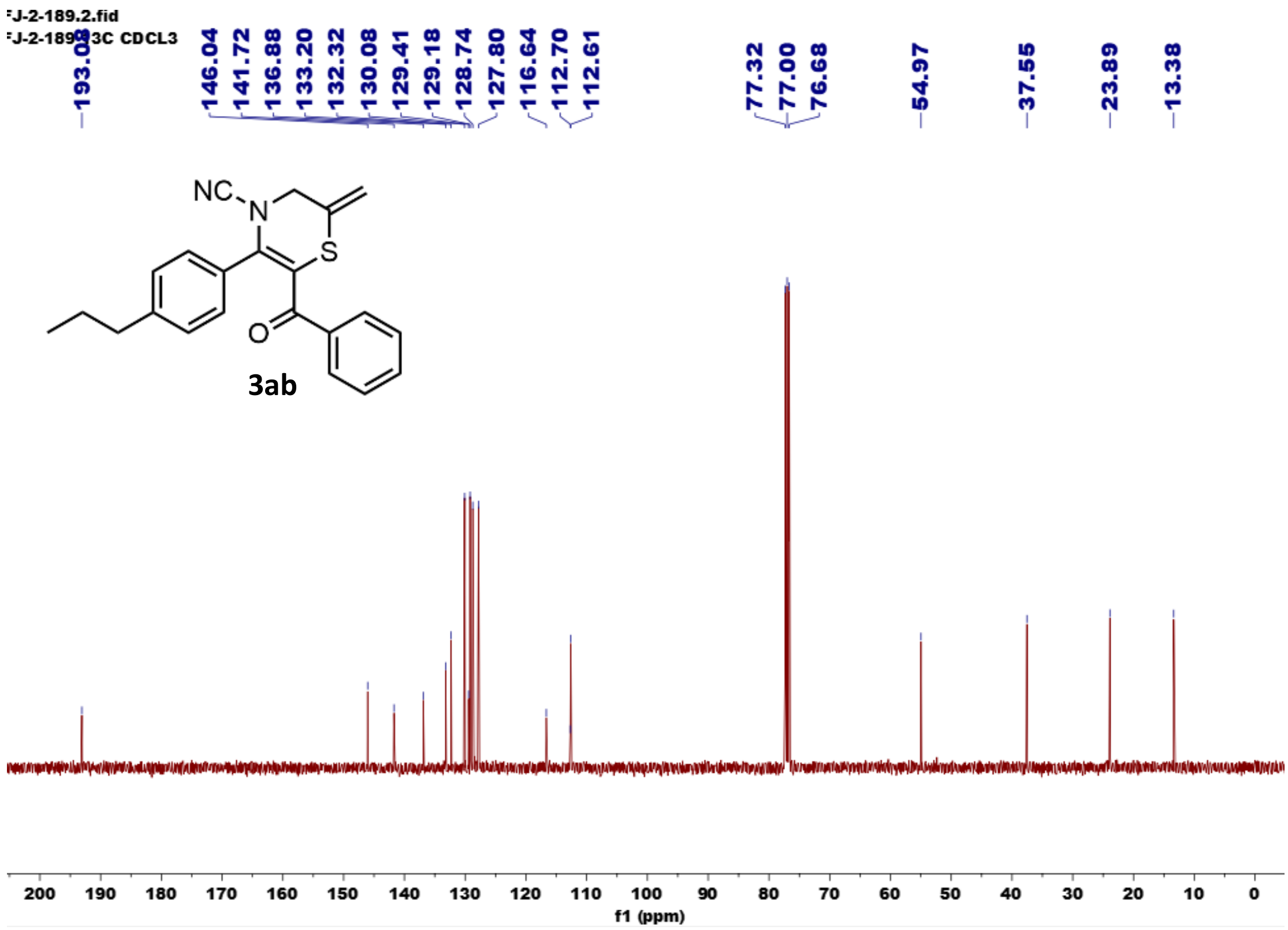

^{1}H NMR spectrum of compound 3ac (400 MHz, $CDCl_3$)

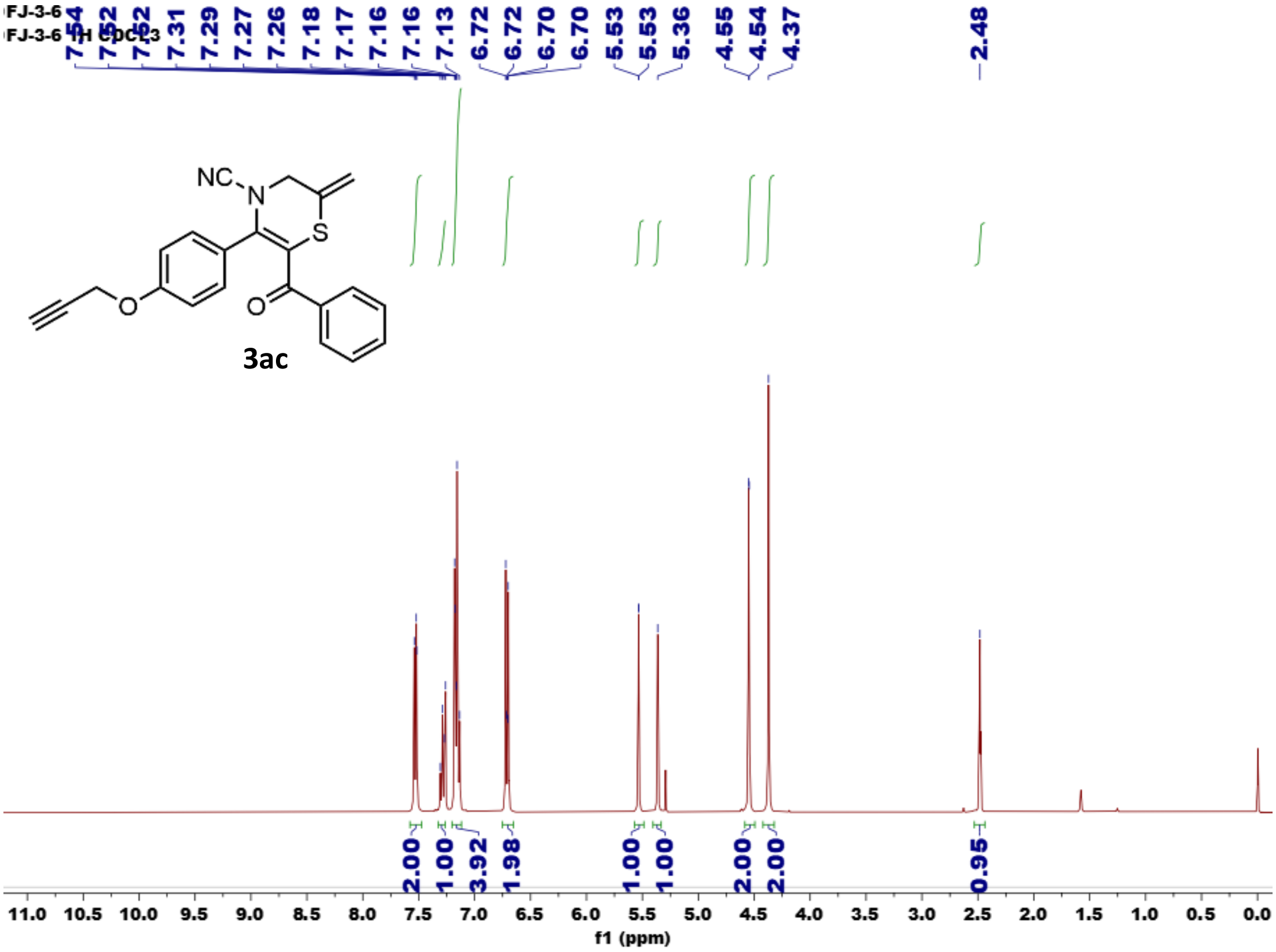

^{13}C NMR spectrum of compound 3ac (101 MHz, $CDCl_3$)

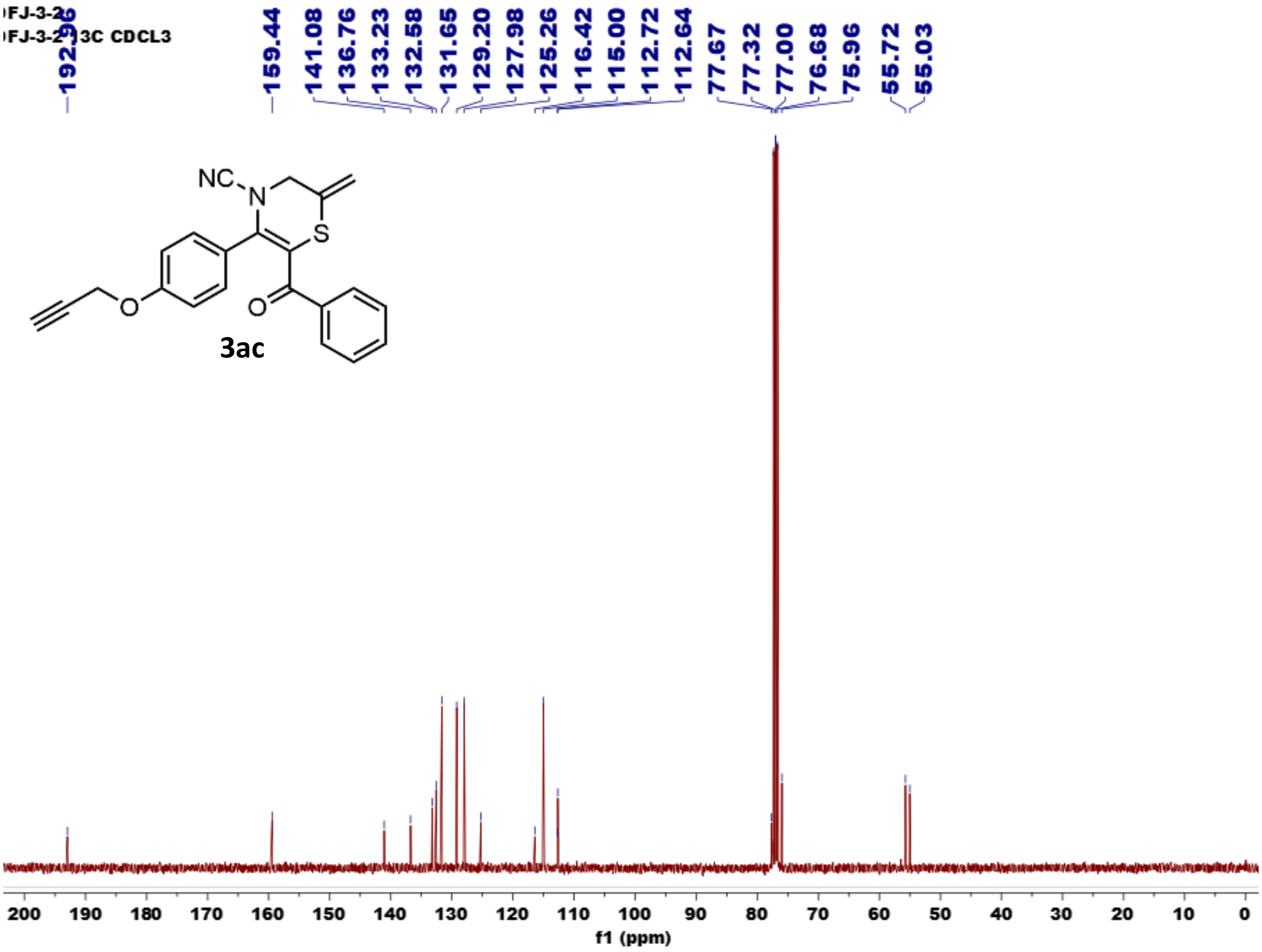

^{1}H NMR spectrum of compound 3ad (400 MHz, $CDCl_3$)

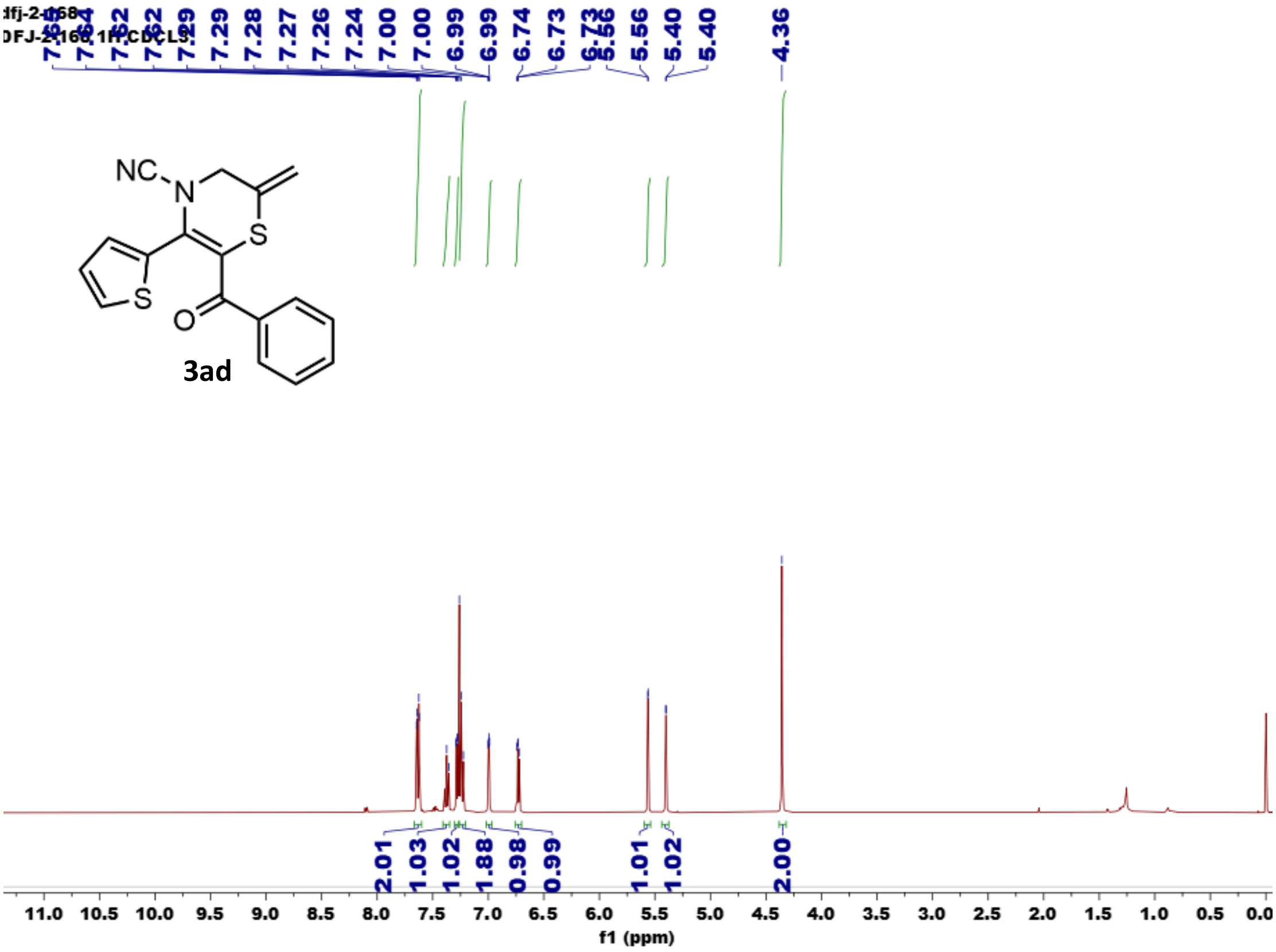

^{13}C NMR spectrum of compound 3ad (101 MHz, $CDCl_3$)

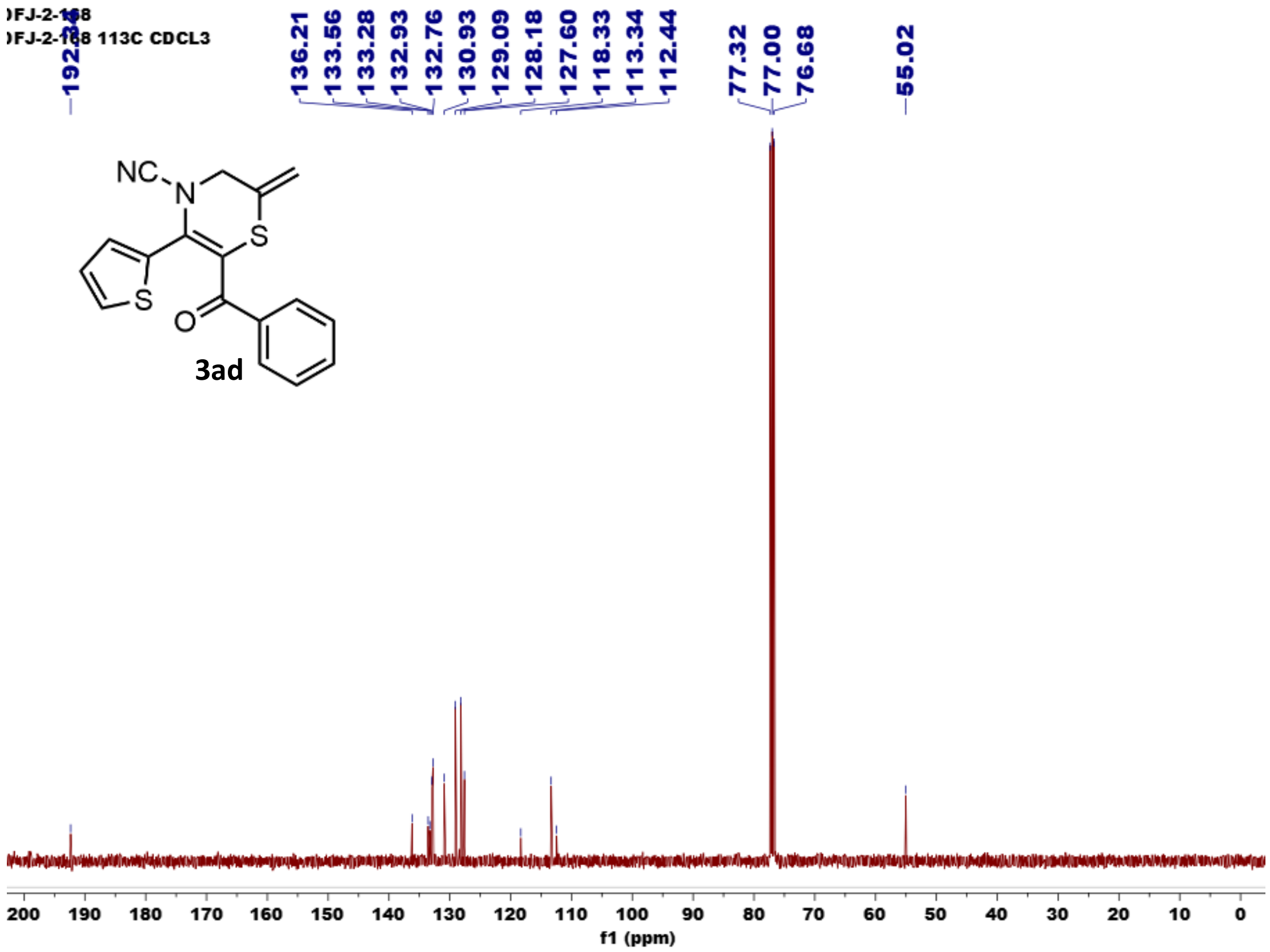

^{1}H NMR spectrum of compound 3ae (400 MHz, $CDCl_3$)

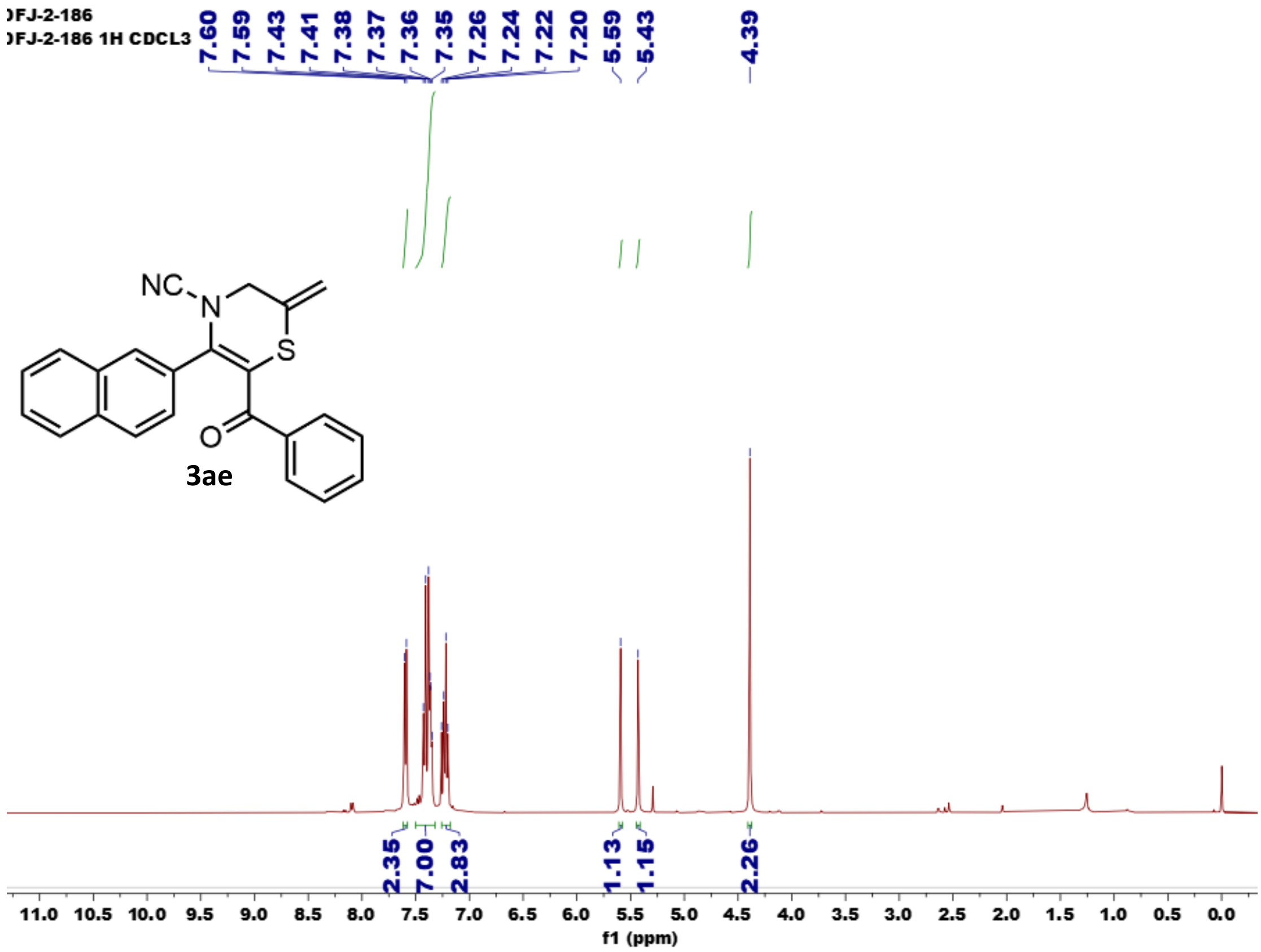

^{13}C NMR spectrum of compound 3ae (101 MHz, $CDCl_3$)

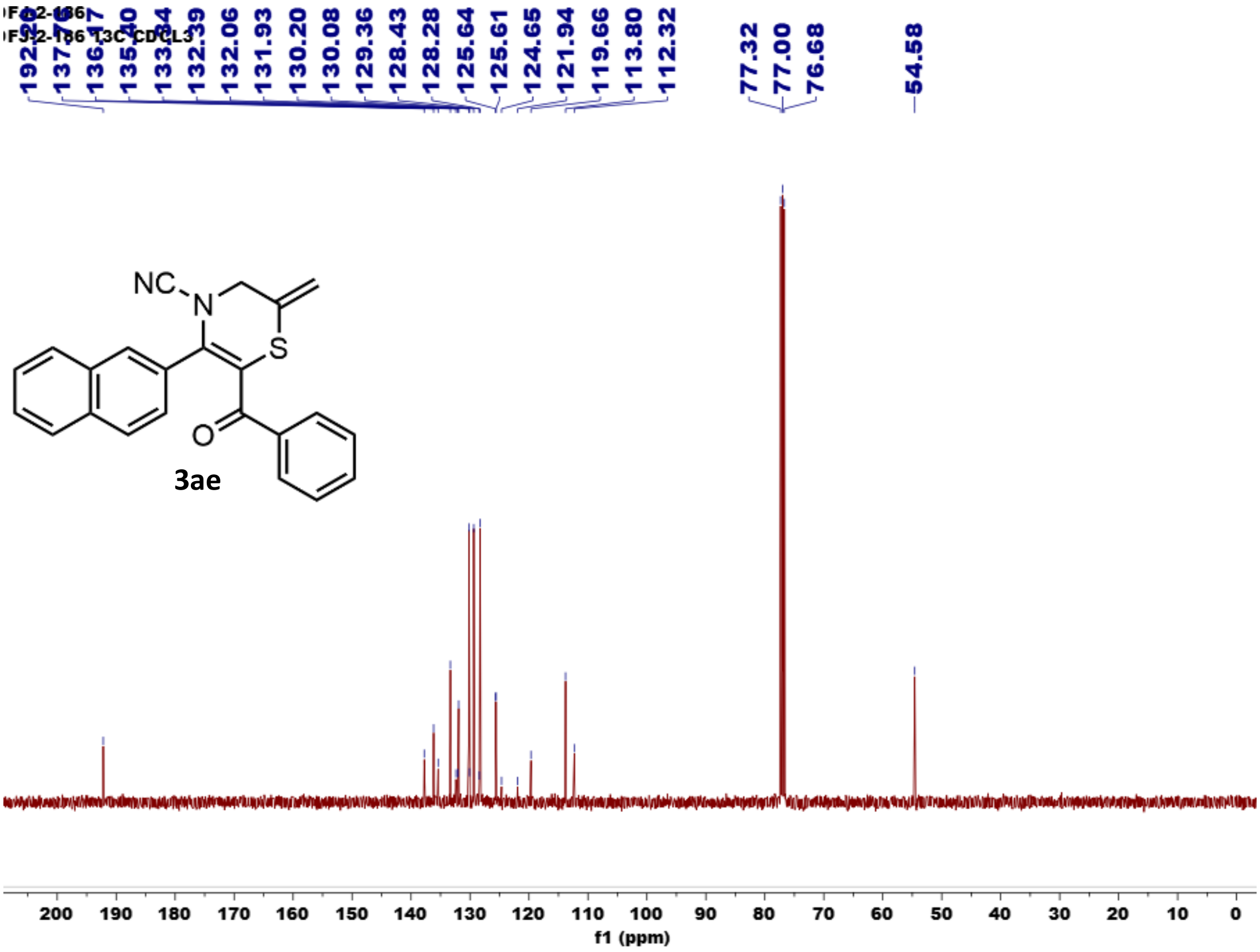

^{1}H NMR spectrum of compound 3af (400 MHz, $CDCl_3$)

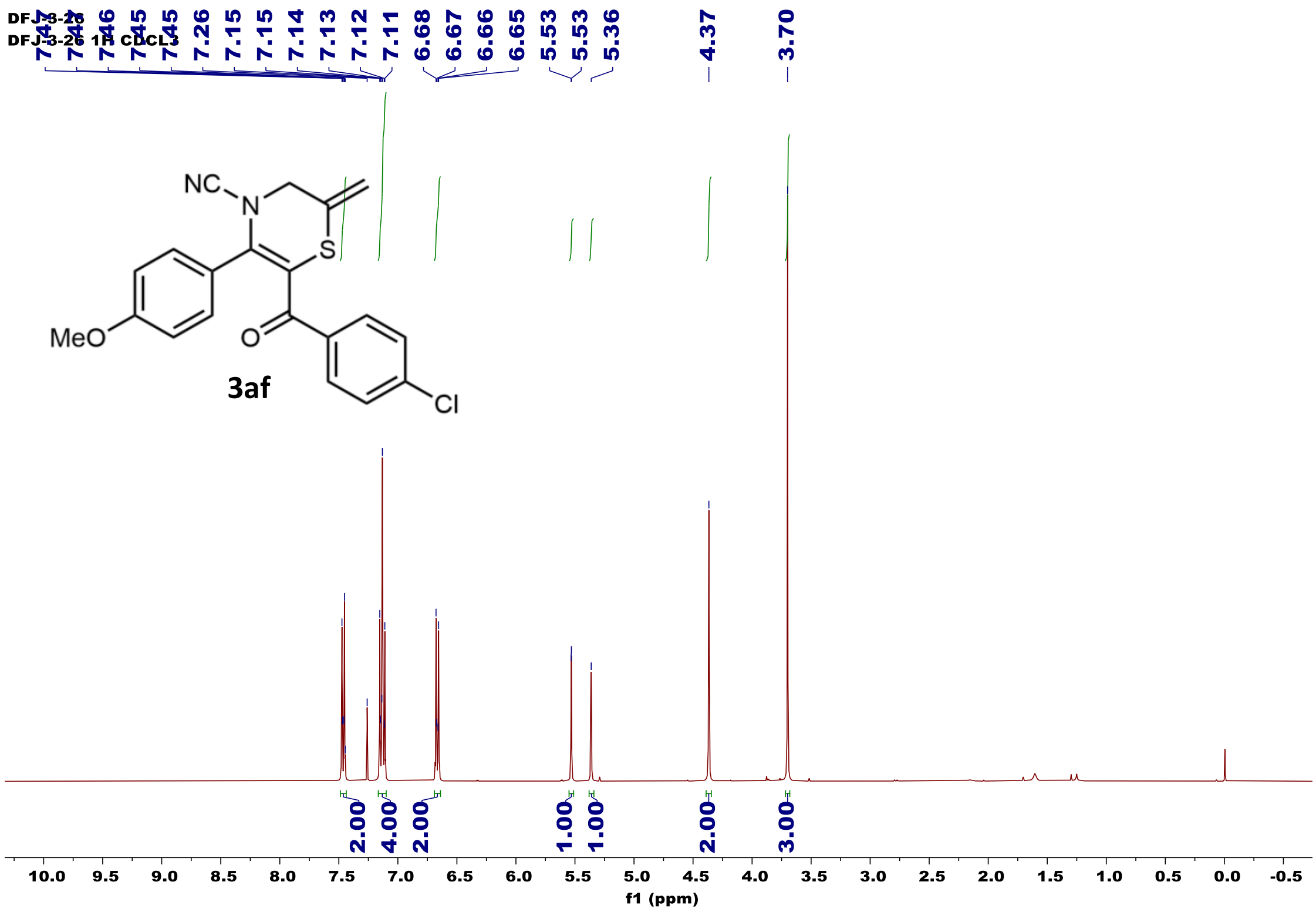

^{13}C NMR spectrum of compound 3af (101 MHz, $CDCl_3$)

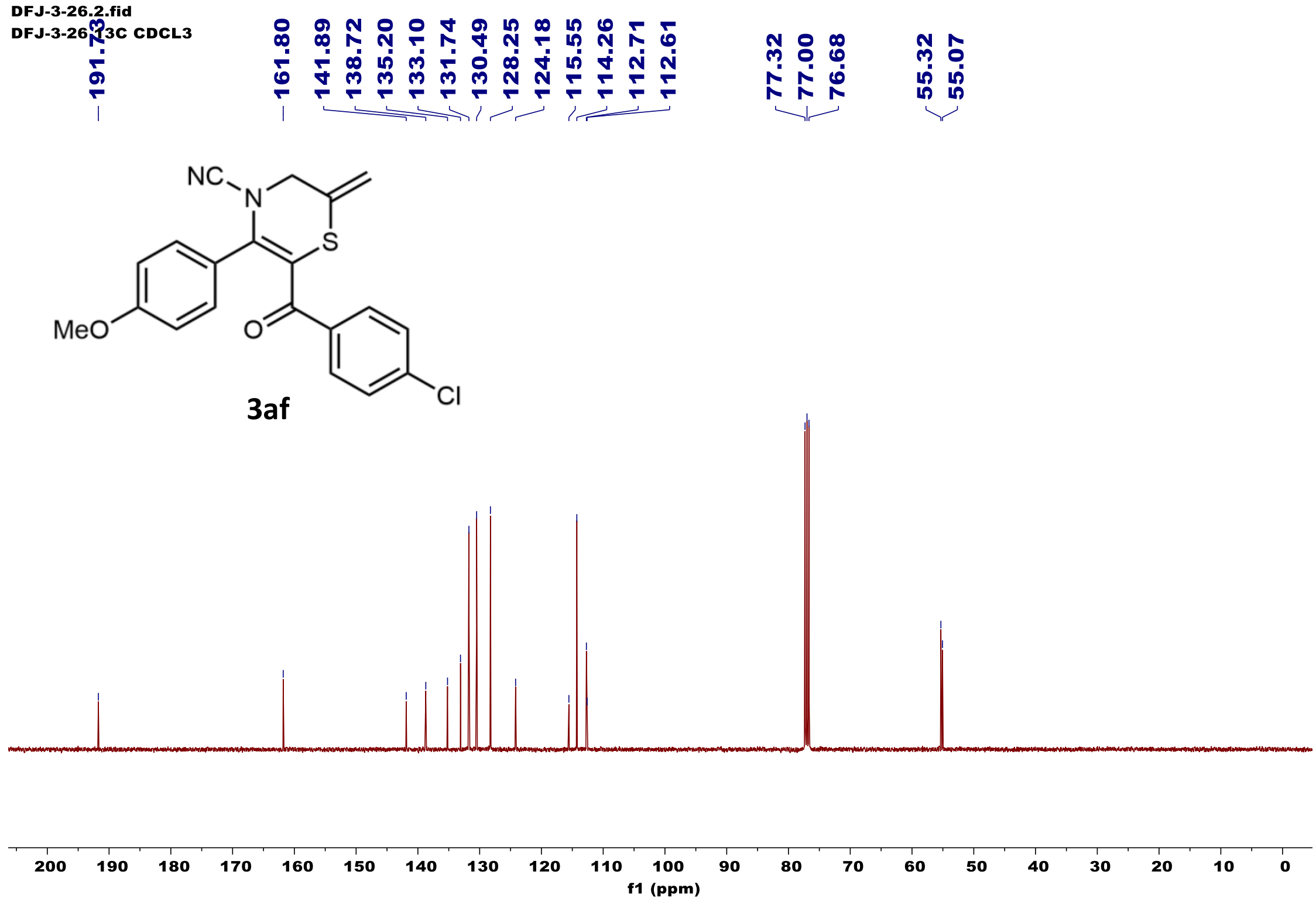

^{1}H NMR spectrum of compound 3ag (400 MHz, $CDCl_3$)

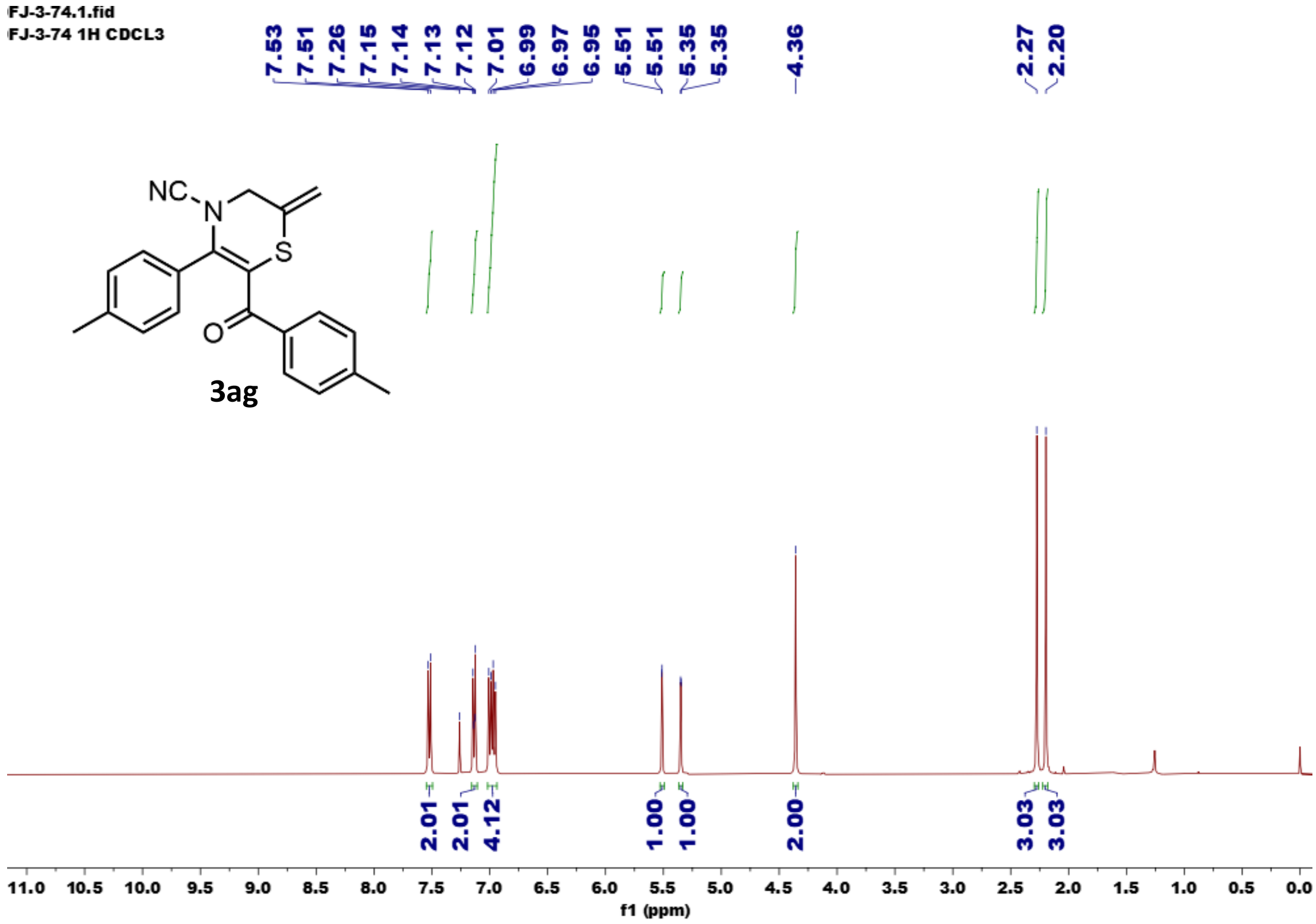

^{13}C NMR spectrum of compound 3ag (101 MHz, $CDCl_3$)

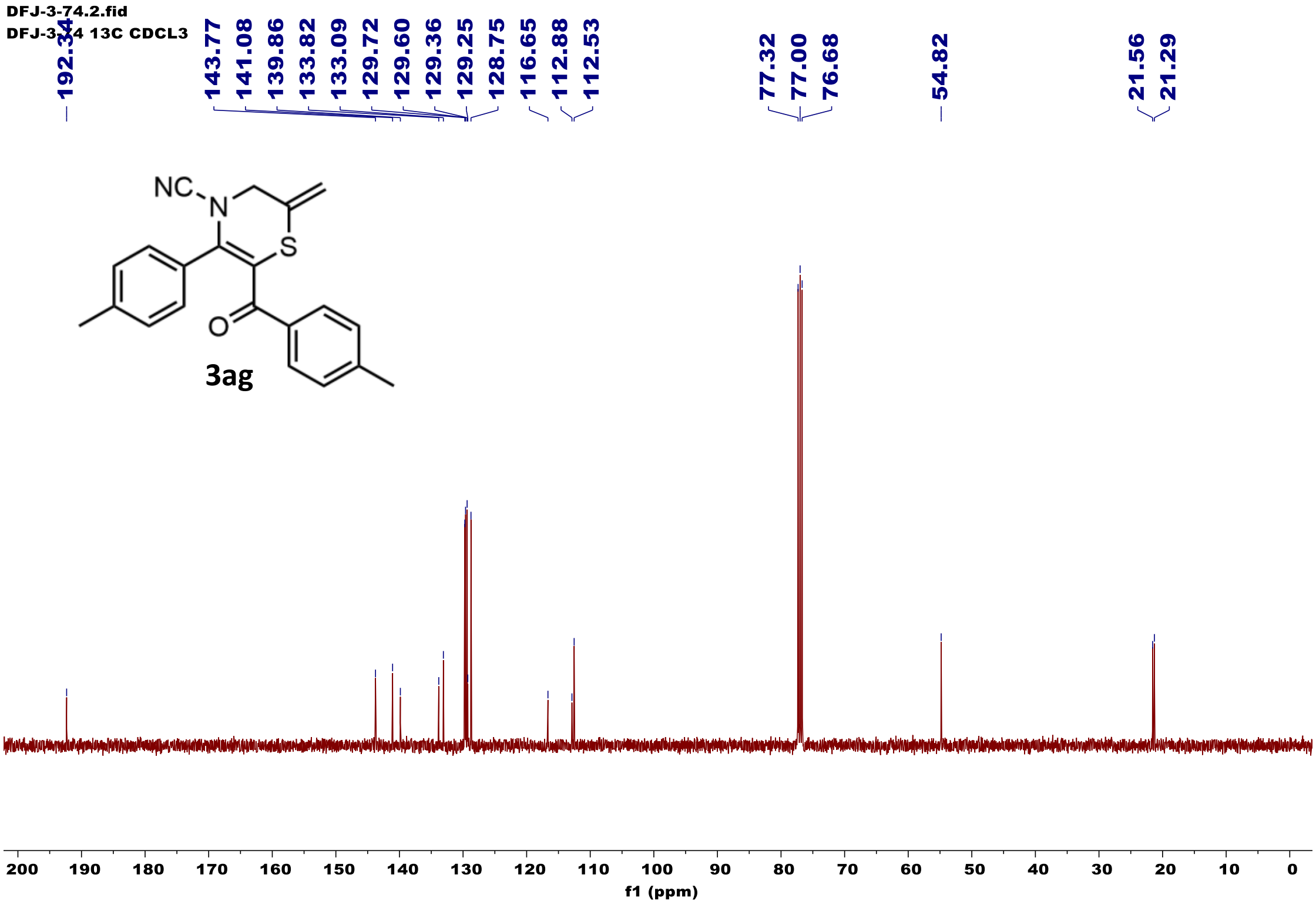

^{1}H NMR spectrum of compound 3ah (400 MHz, $CDCl_3$)

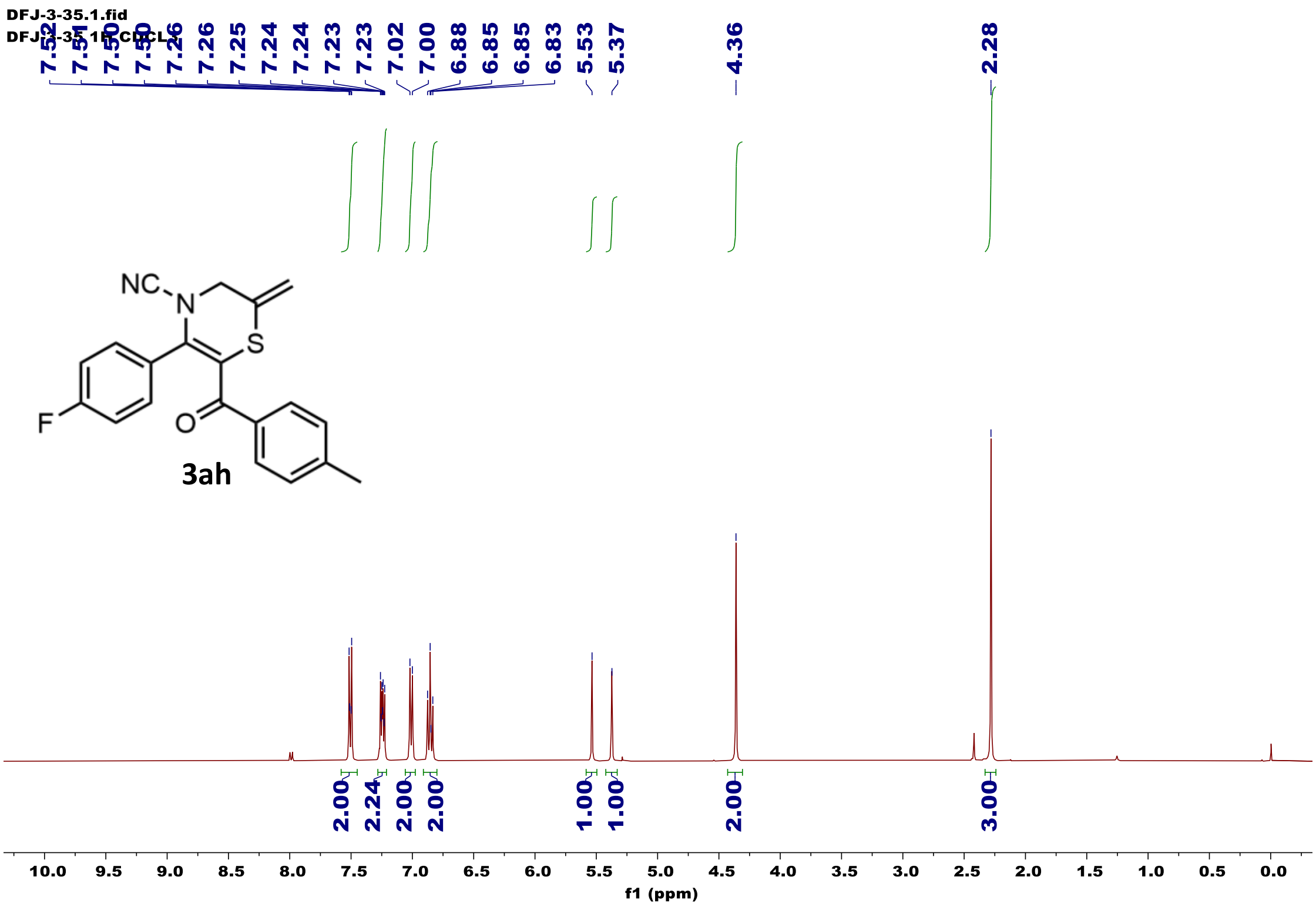

^{13}C NMR spectrum of compound 3ah (101 MHz, $CDCl_3$)

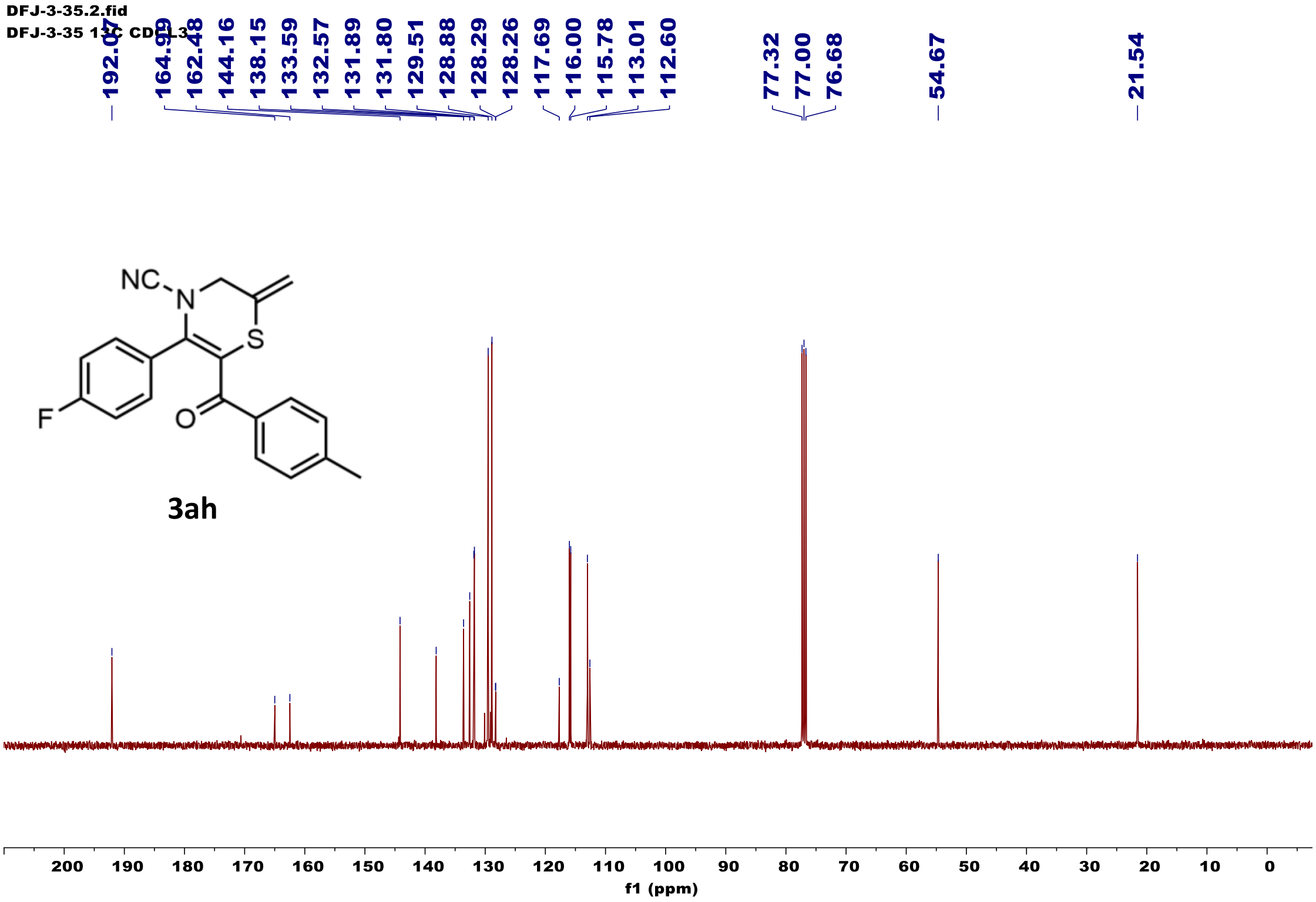

^{19}F NMR spectrum of compound 3ah (376 MHz, $CDCl_3$)

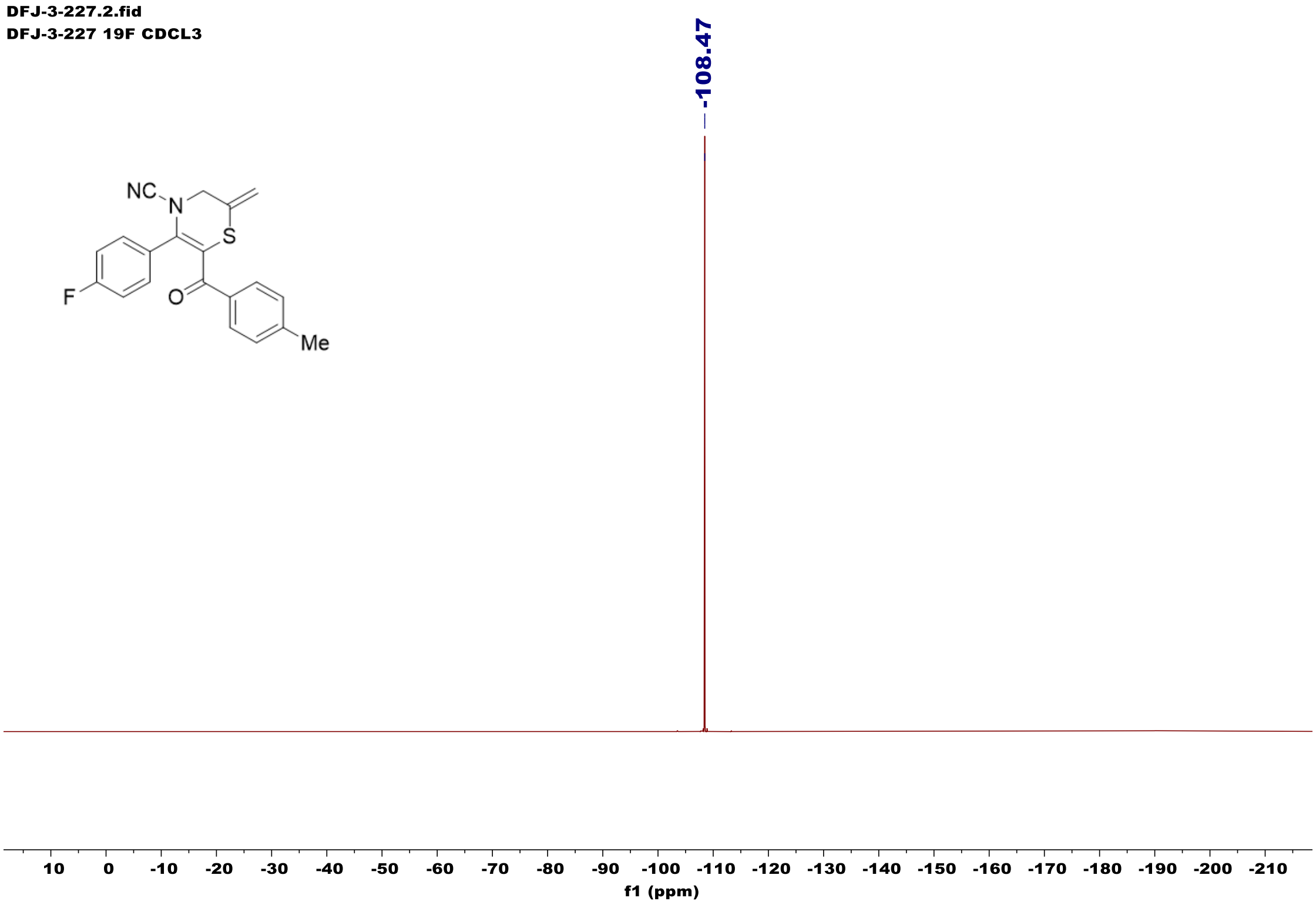

^{1}H NMR spectrum of compound 3ai (400 MHz, $CDCl_3$)

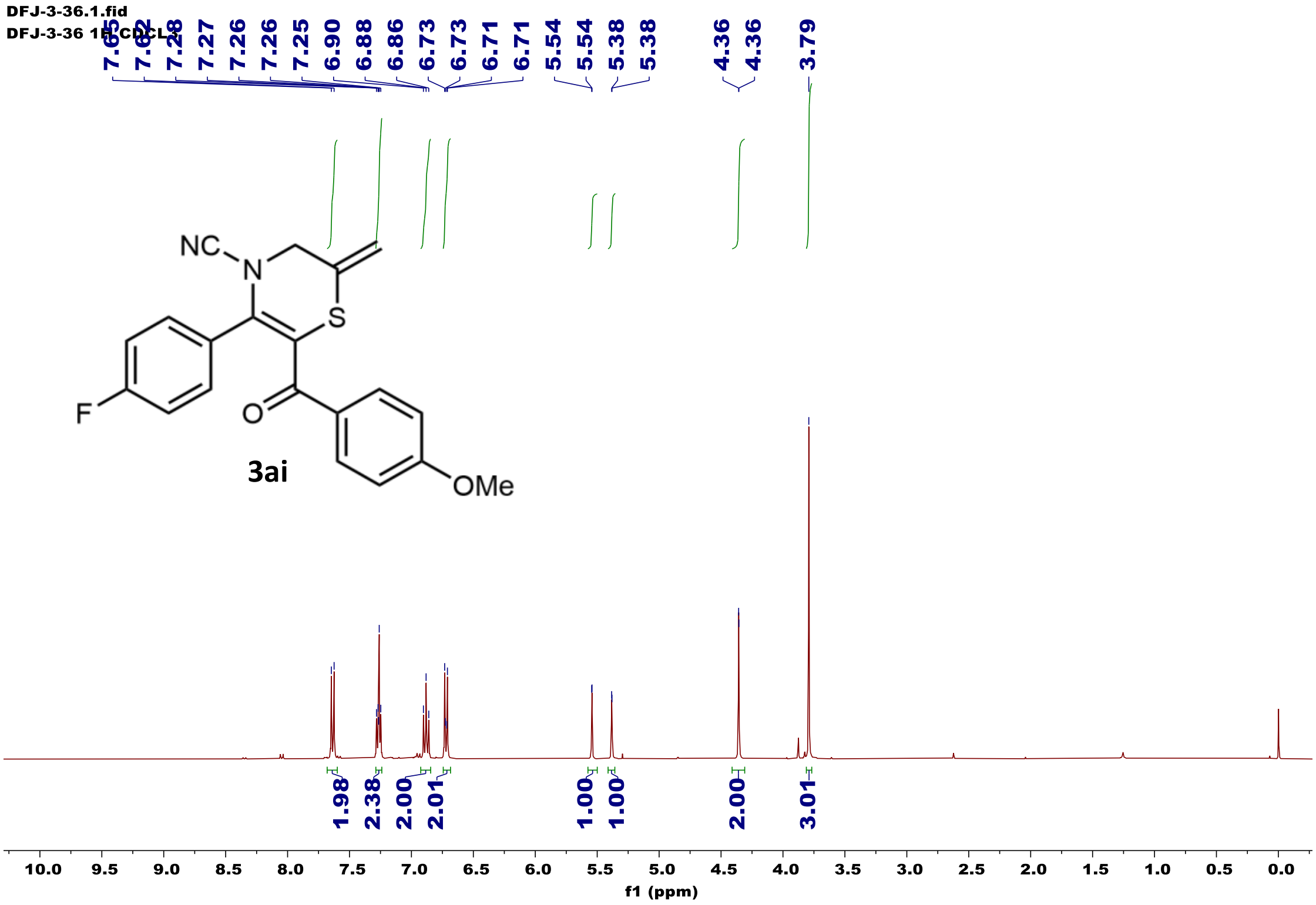

^{13}C NMR spectrum of compound 3ai (101 MHz, $CDCl_3$)

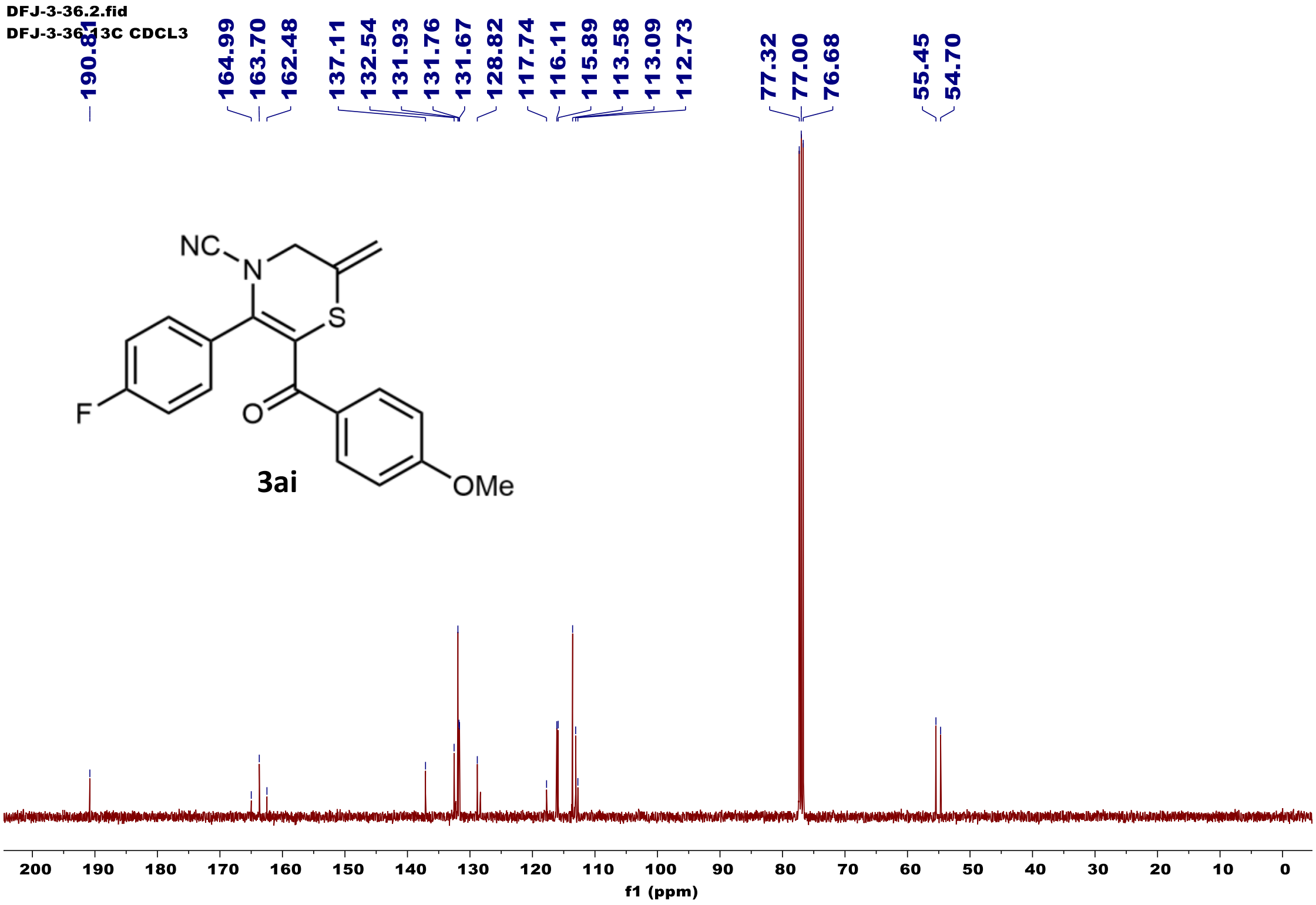

^{19}F NMR spectrum of compound 3ai (376 MHz, $CDCl_3$)

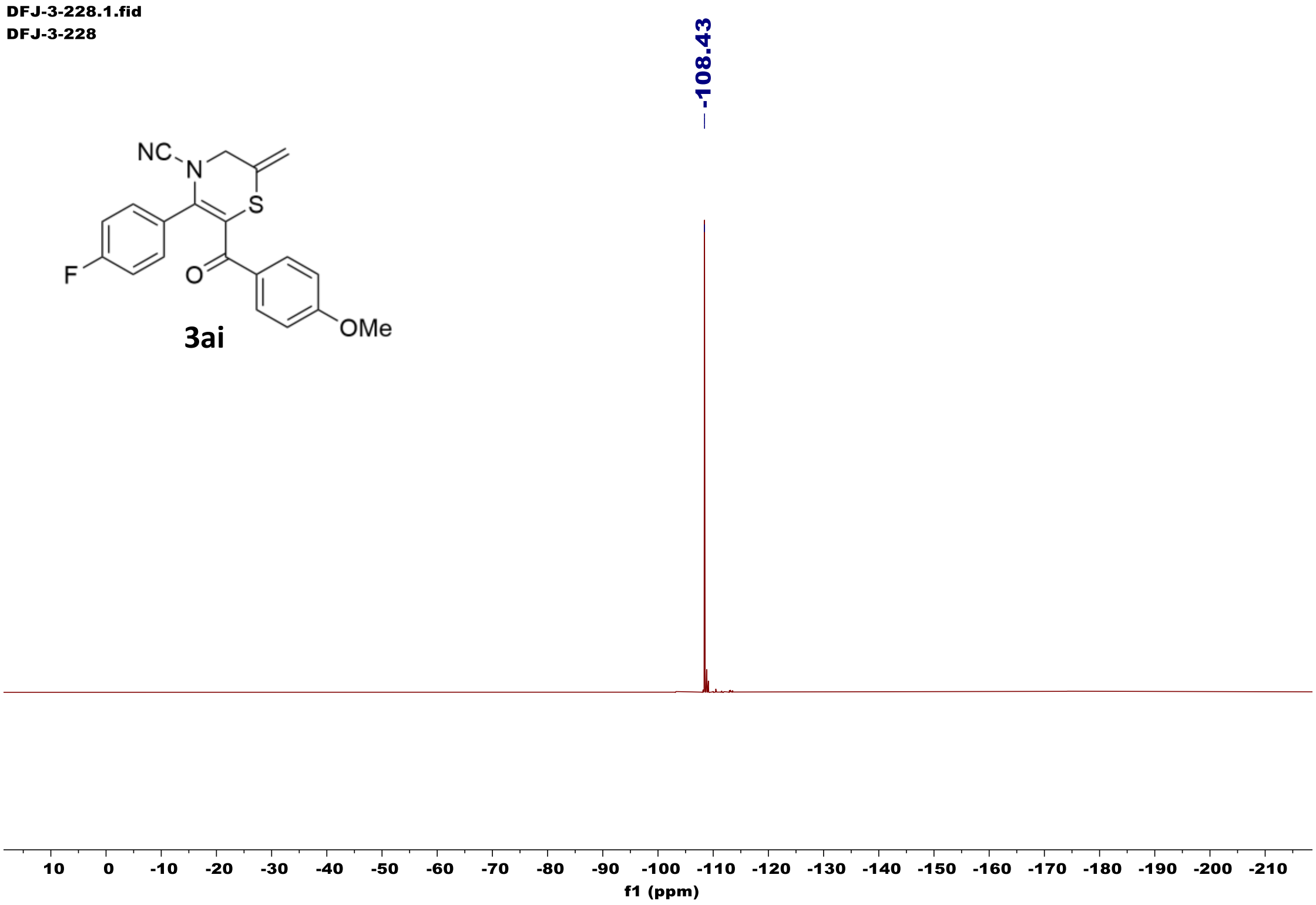

^{1}H NMR spectrum of compound 3aj (400 MHz, $CDCl_3$)

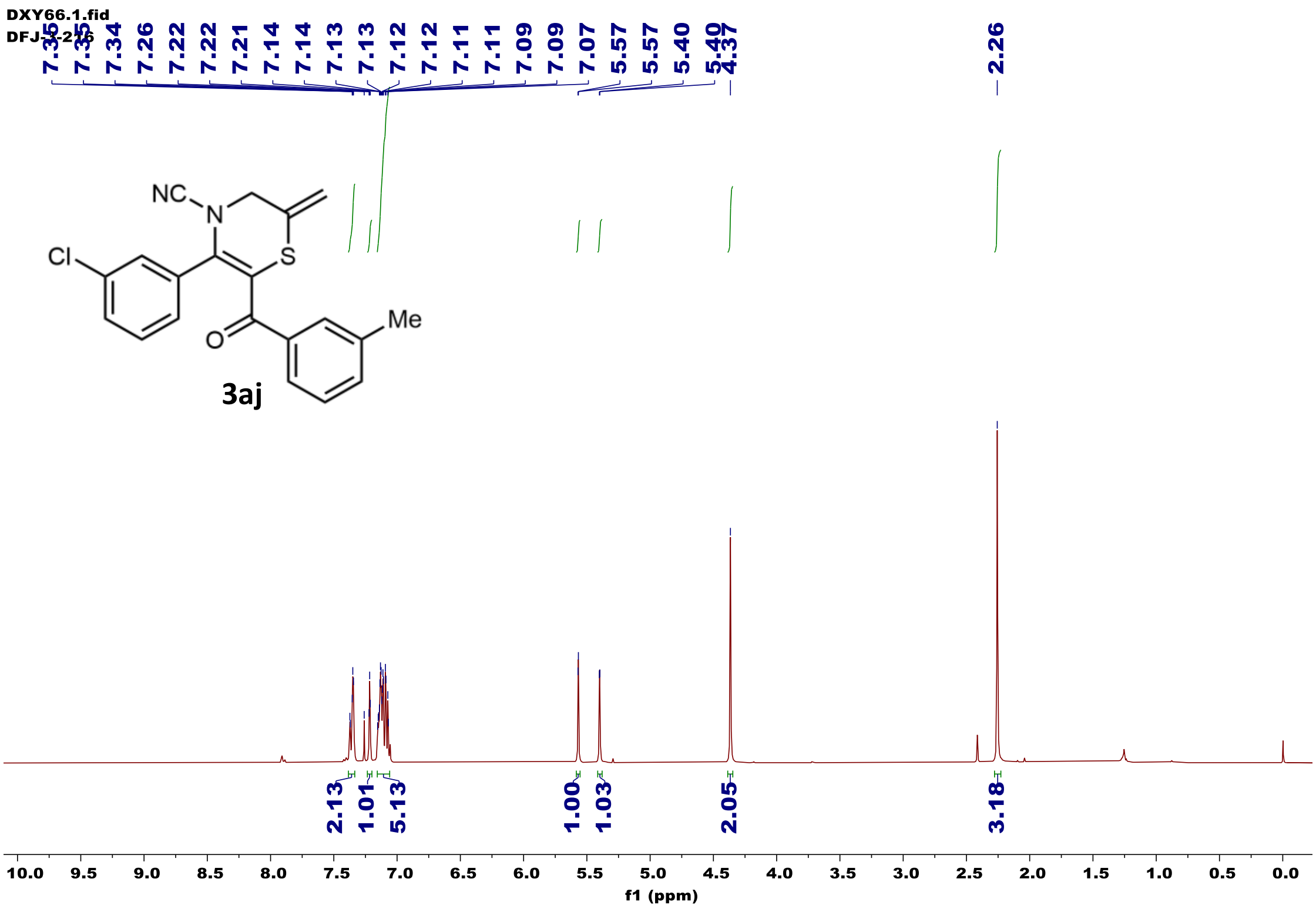

^{13}C NMR spectrum of compound 3aj (101 MHz, $CDCl_3$)

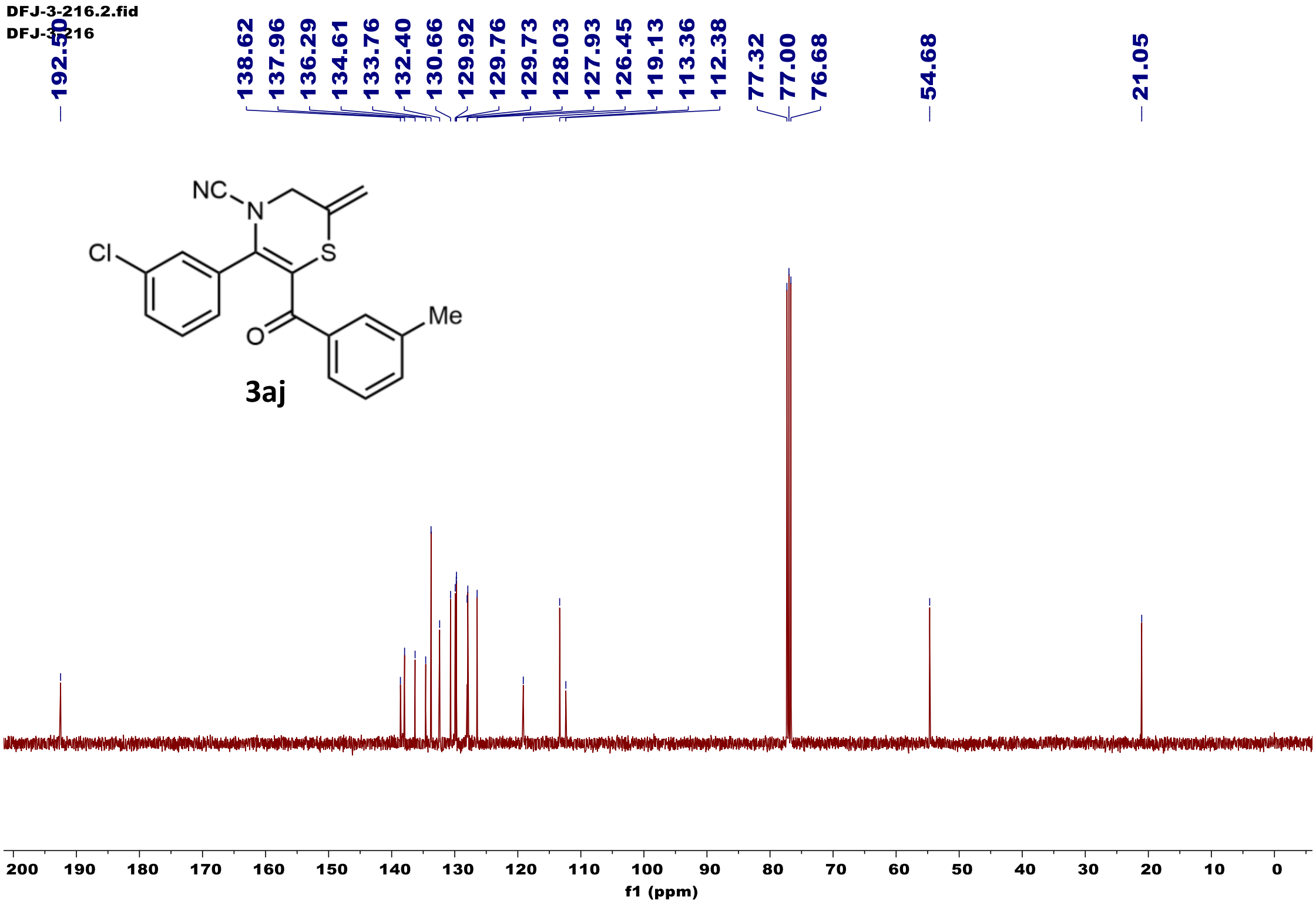

^{1}H NMR spectrum of compound 3ak (400 MHz, $CDCl_3$)

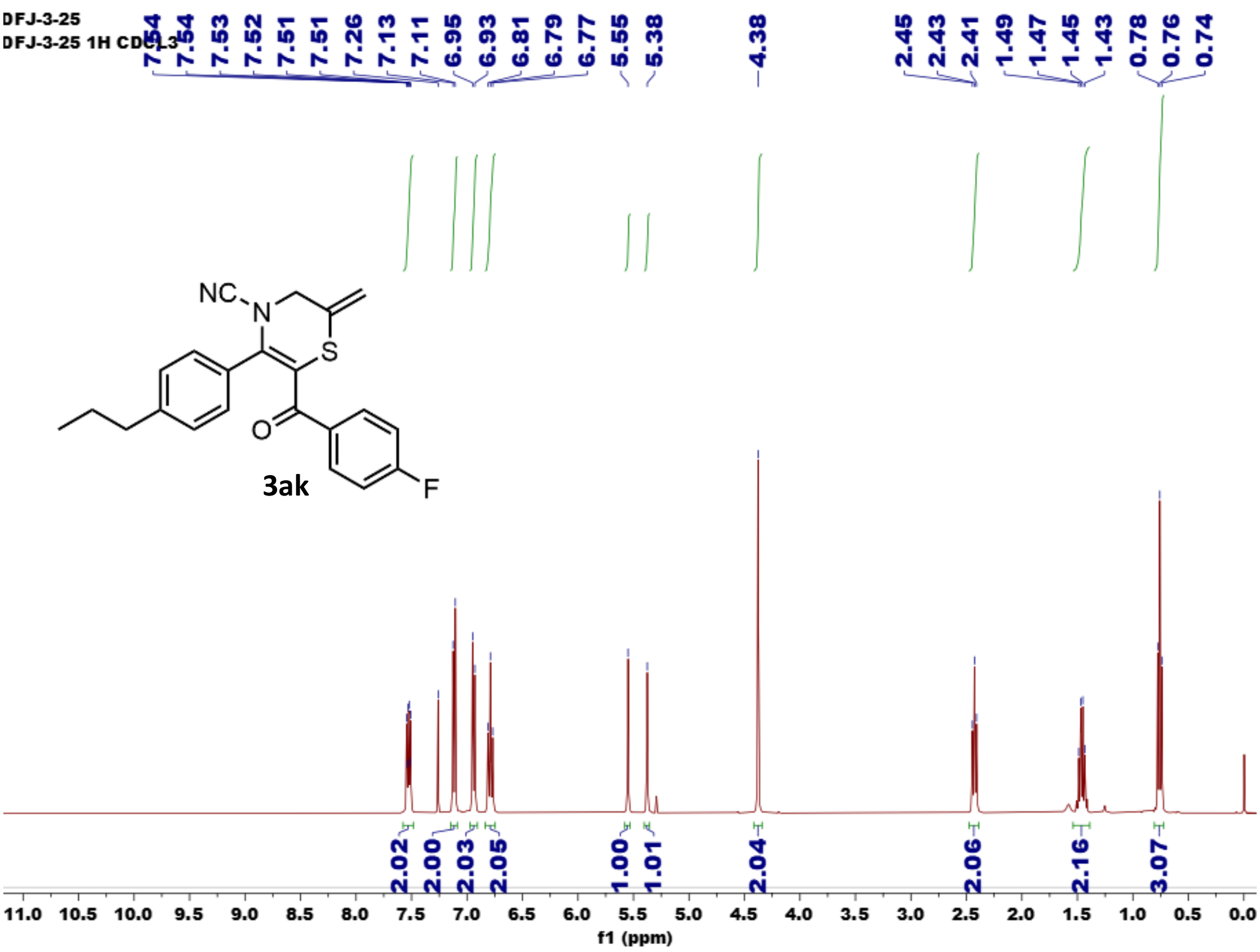

^{13}C NMR spectrum of compound 3ak (101 MHz, $CDCl_3$)

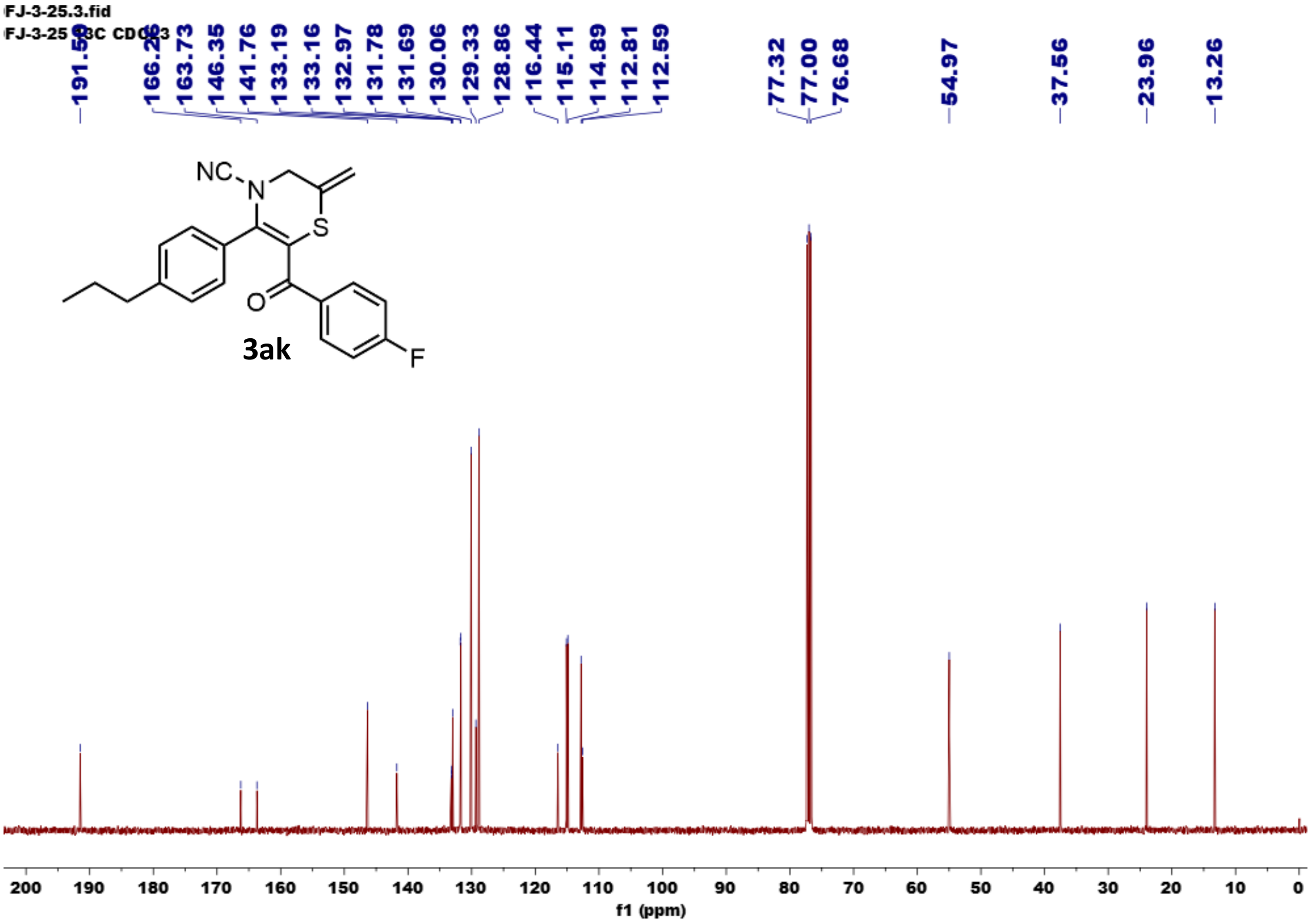

^{19}F NMR spectrum of compound 3ak (376 MHz, $CDCl_3$)

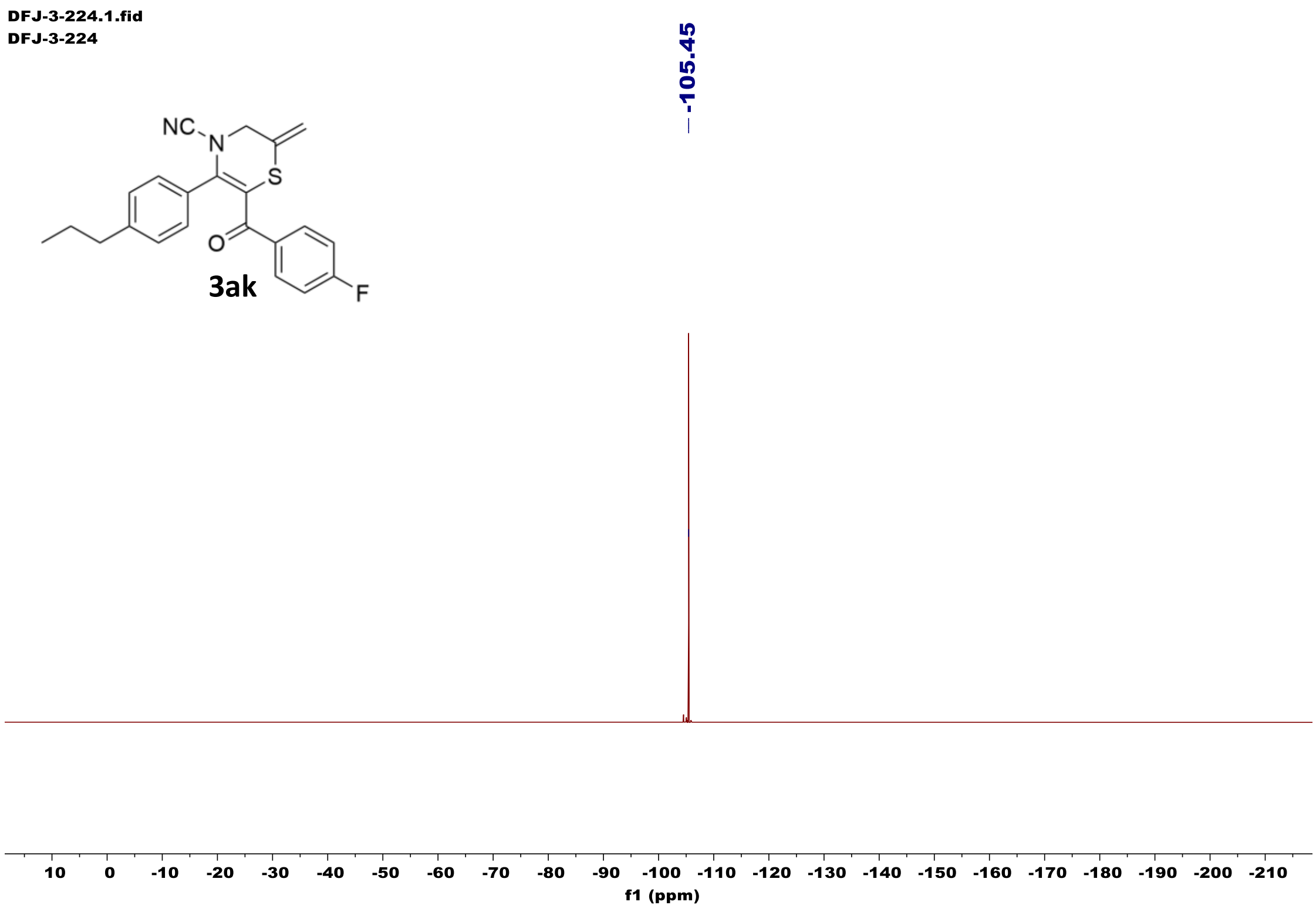

^{1}H NMR spectrum of compound 3al (400 MHz, $CDCl_3$)

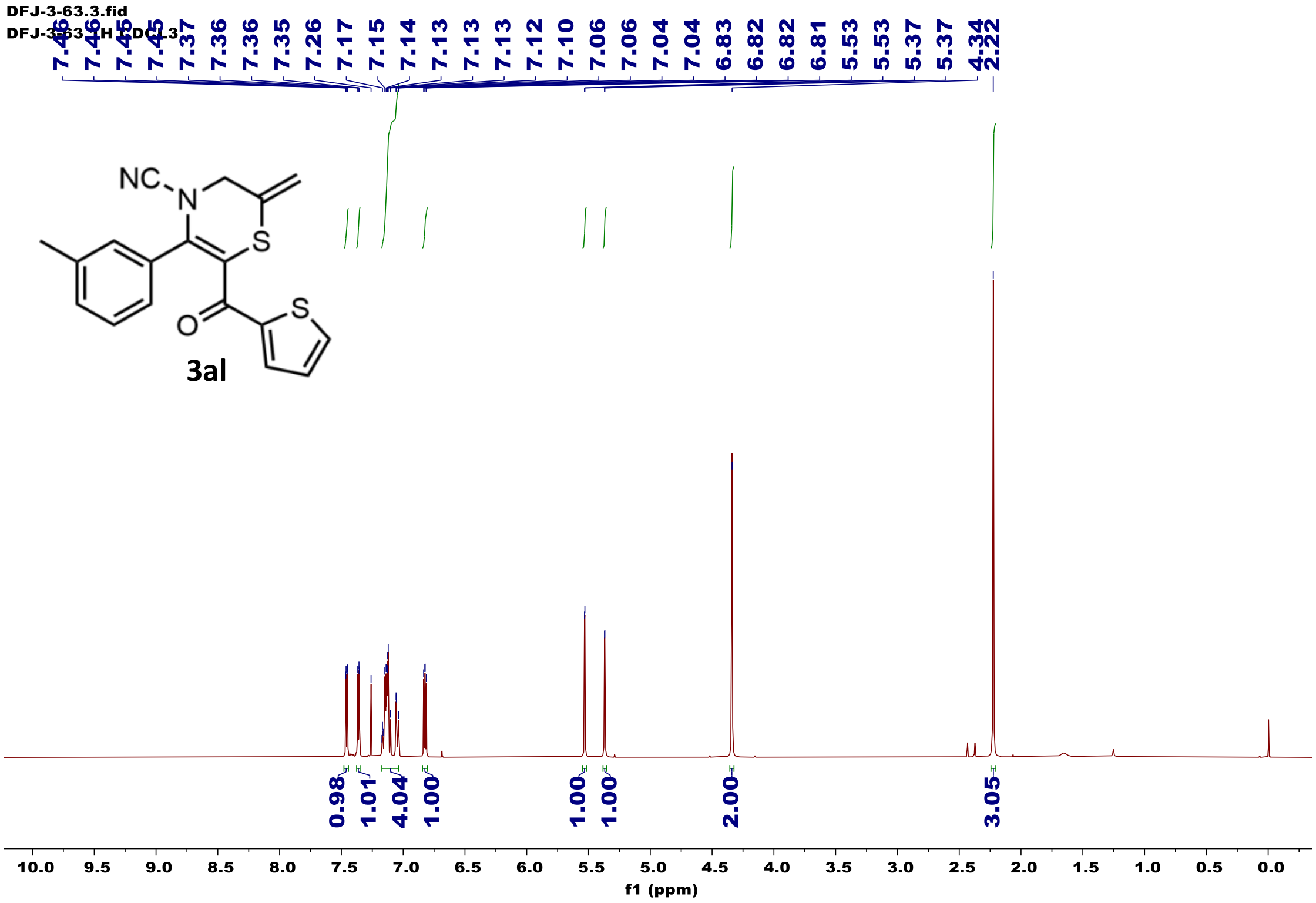

^{13}C NMR spectrum of compound 3al (101 MHz, $CDCl_3$)

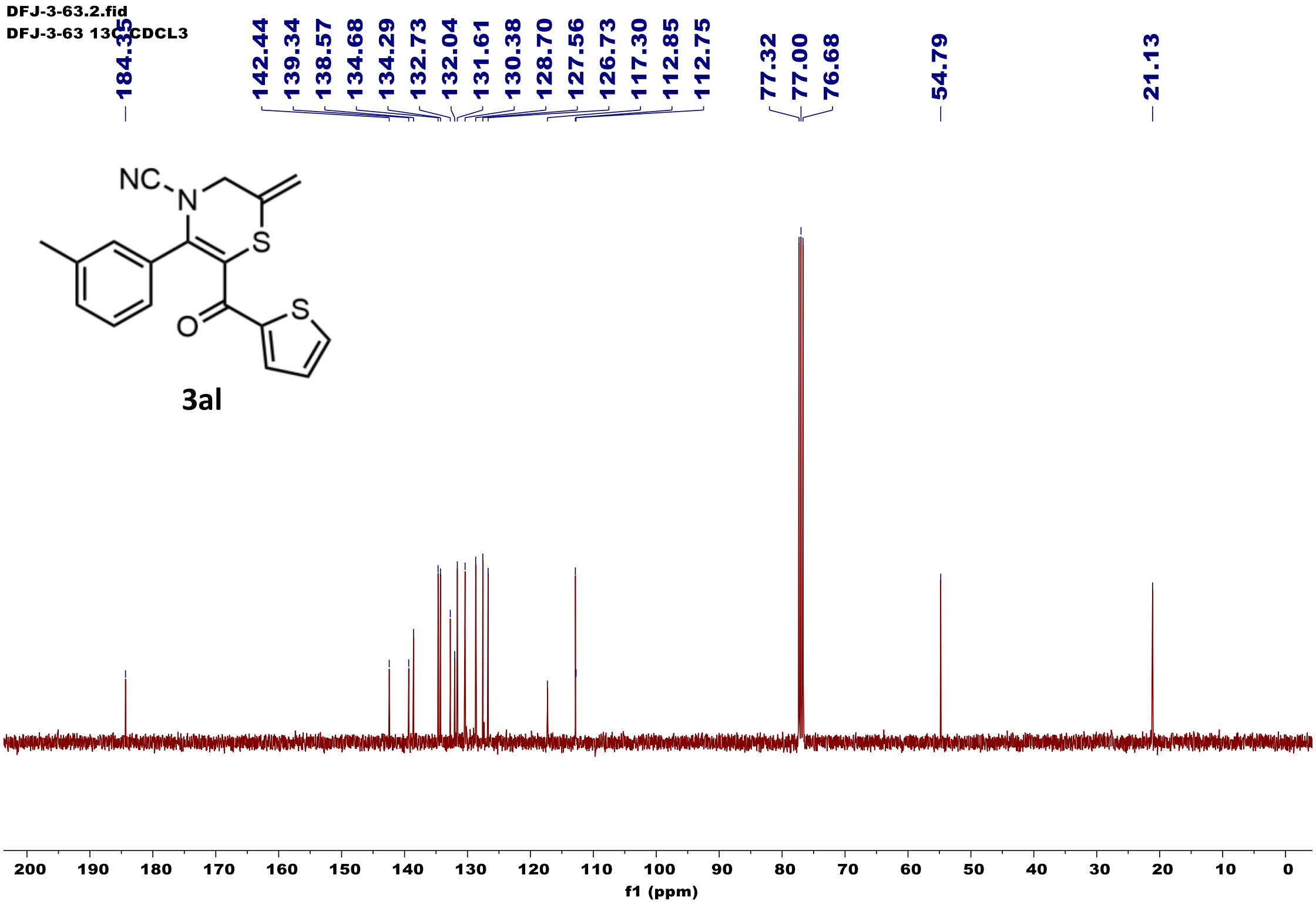

^{1}H NMR spectrum of compound 3am (400 MHz, $CDCl_3$)

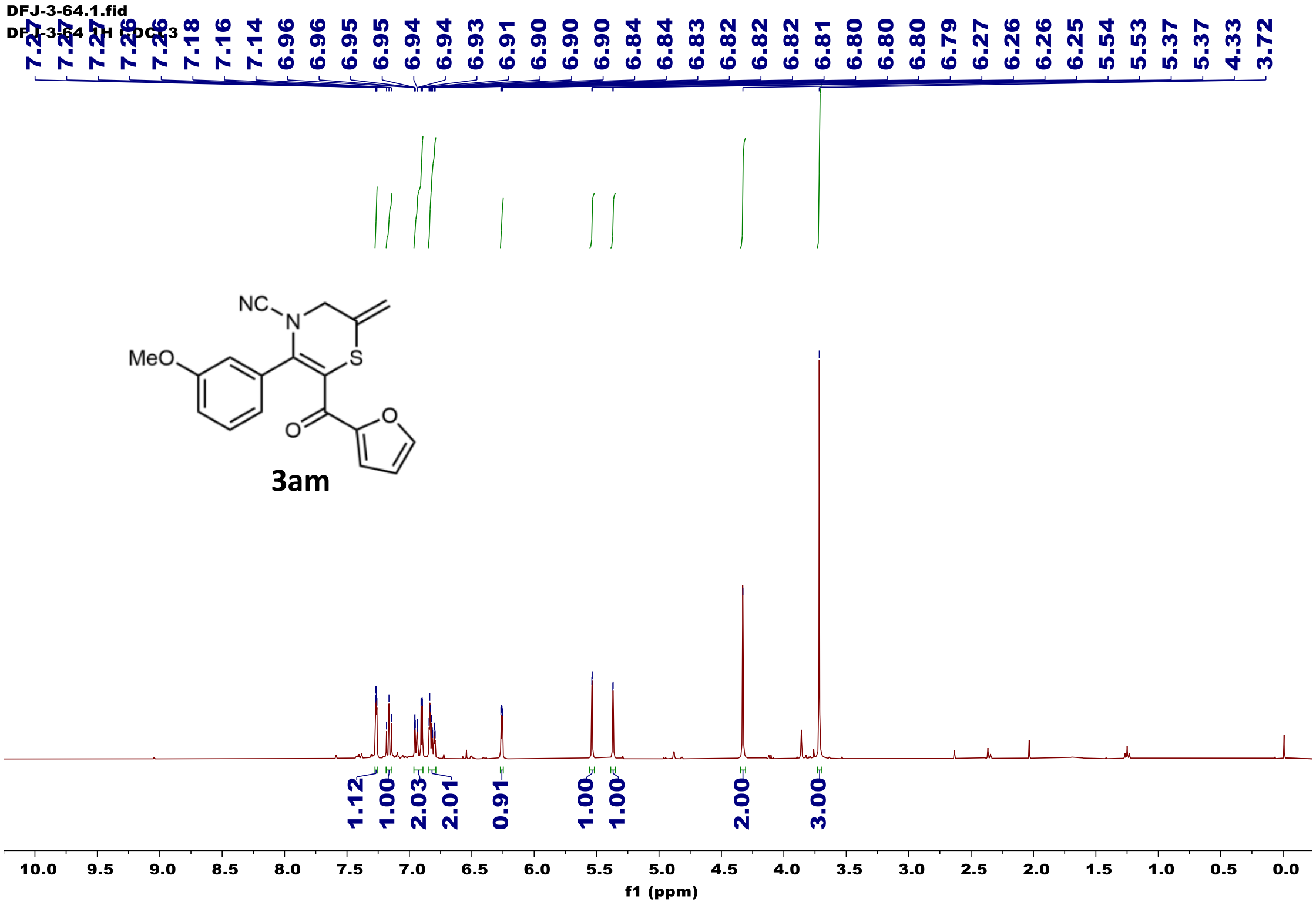

^{13}C NMR spectrum of compound 3am (101 MHz, $CDCl_3$)

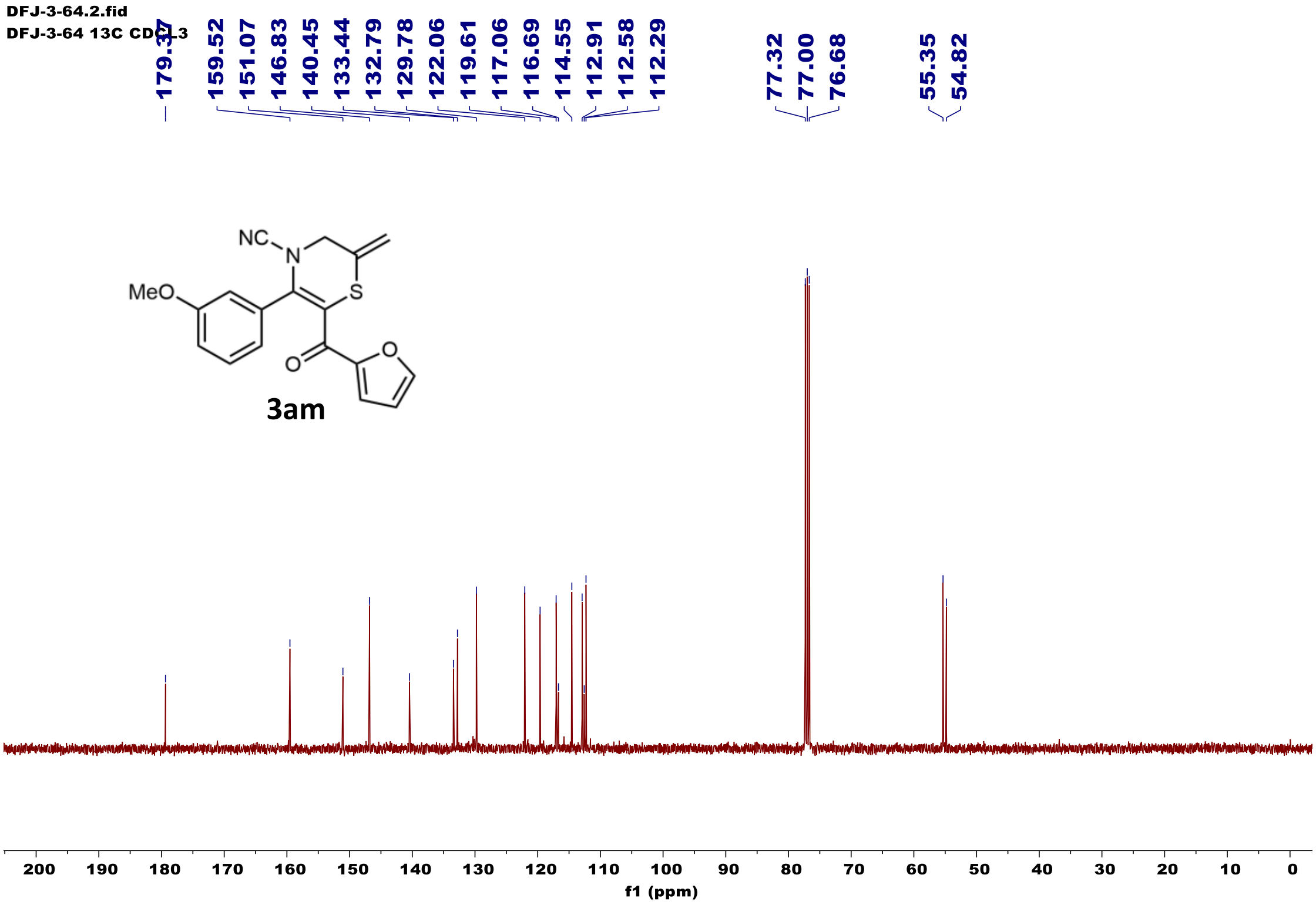

^{1}H NMR spectrum of compound 3an (400 MHz, $CDCl_3$)

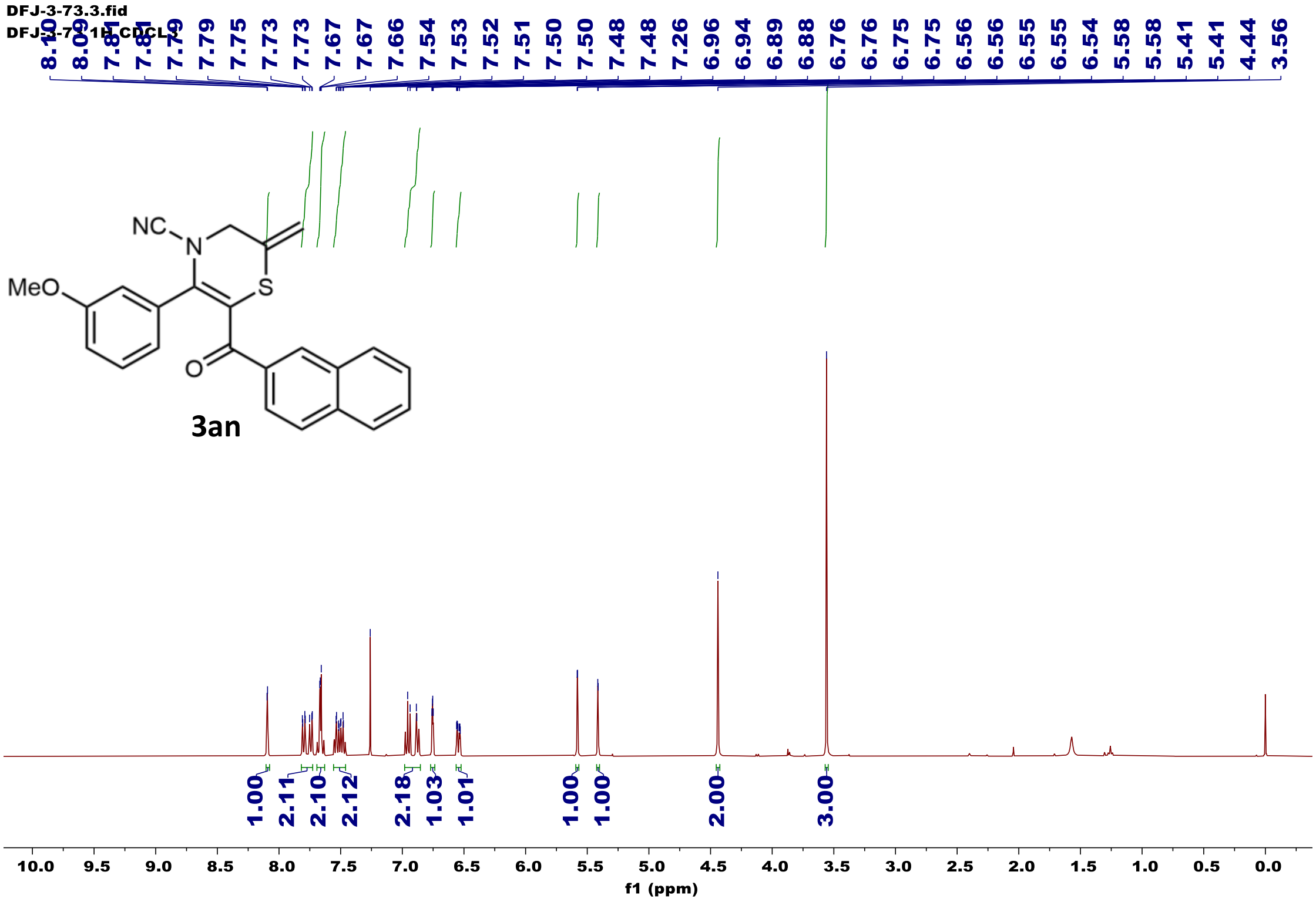

^{13}C NMR spectrum of compound 3an (101 MHz, $CDCl_3$)

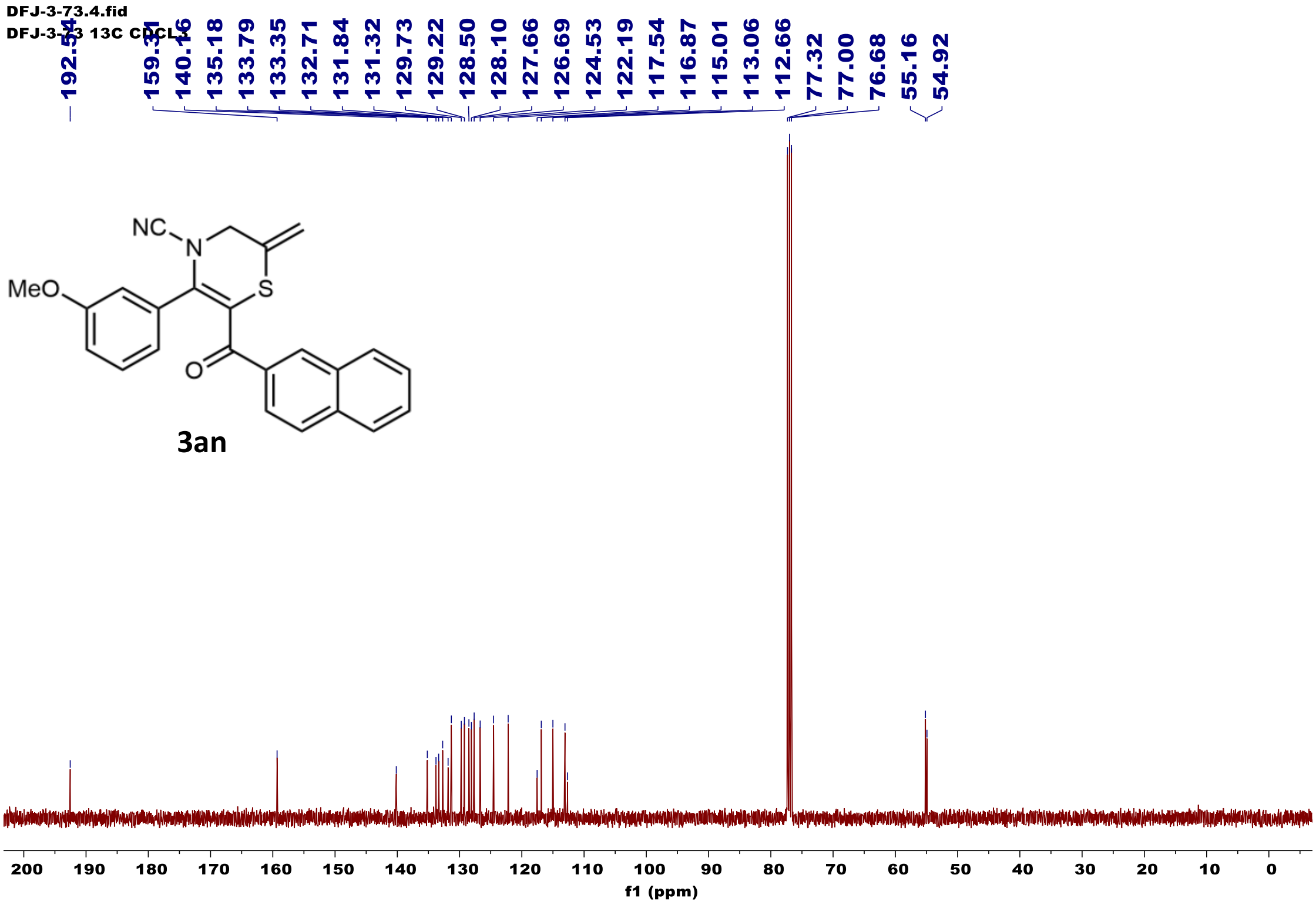

Supplement: Supplementary file 1 [file oc6c00645_si_001.pdf]
